# Multiomics integration prioritizes potential drug targets for multiple sclerosis

**DOI:** 10.1073/pnas.2425537122

**Published:** 2025-06-27

**Authors:** Yuan Jiang, Qianwen Liu, Pernilla Stridh, Ingrid Kockum, Tomas Olsson, Lars Alfredsson, Lina-Marcela Diaz-Gallo, Xia Jiang

**Affiliations:** ^a^Department of Clinical Neuroscience, The Karolinska Neuroimmunology & Multiple Sclerosis Centre, Centre for Molecular Medicine, Karolinska Institutet, Stockholm 17177, Sweden; ^b^Institute of Environmental Medicine, Karolinska Institutet, Stockholm 17177, Sweden; ^c^Department of Medicine, Division of Rheumatology, Centre for Molecular Medicine, Karolinska Institutet, Stockholm 17177, Sweden; ^d^Department of Epidemiology and Health Statistics, West China School of Public Health and West China Fourth Hospital, Sichuan University, Chengdu, China

**Keywords:** causal, genomics, transcriptomics, proteomics, multiple sclerosis

## Abstract

Multiple sclerosis (MS) is an immune-mediated disease with no current cure. Drug discovery and repurposing are essential to enhance treatment efficacy and safety. We utilized summary statistics for protein quantitative trait loci (pQTL) of 2,004 plasma and 1,443 brain proteins, a genome-wide association study of MS susceptibility with 14,802 cases and 26,703 controls, both bulk and cell-type specific transcriptome data, and external pQTL data in blood and brain. Our integrative analysis included a proteome-wide association study to identify MS-associated proteins, followed by summary-data-based Mendelian randomization to determine potential causal associations. We used the HEIDI test and Bayesian colocalization analysis to distinguish pleiotropy from linkage. Proteins passing all analyses were prioritized as potential drug targets. We further conducted pathway annotations and protein–protein interaction network analysis (PPI) and verified our findings at mRNA and protein levels. We tested hundreds of MS-associated proteins and confirmed 18 potential causal proteins (nine in plasma and nine in brain). Among these, we found 78 annotated pathways and 16 existing non-MS drugs targeting six proteins. We also identified intricateAQ PPIs among seven potential drug targets and 19 existing MS drug targets, as well as PPIs of four targets across plasma and brain. We identified two targets using bulk mRNA expression data and four targets expressed in MS-related cell types. We finally verified 10 targets using external pQTL data. We prioritized 18 potential drug targets in plasma and brain, elucidating the underlying pathology and providing evidence for potential drug discovery and repurposing in MS.

## Significance

We tested hundreds of multiple sclerosis (MS)-associated proteins in plasma and brain and confirmed the potential causal role of 18 proteins (9 in plasma and 9 in brain) through comprehensive analytical strategies. We revealed intricate interactions between seven of these 18 proteins and 19 known MS drug targets, as well as interactions among 11 of these 18 proteins within and across plasma and brain. Furthermore, we identified 16 existing non-MS drugs targeting six of these 18 potential targets. These findings present significant potential for both the discovery of new drugs and the repurposing of existing ones. The reliability of our findings was further supported by 78 annotated pathways, transcriptional evidence on six targets, and external validation on 10 targets.

Multiple sclerosis (MS) is an immune-mediated inflammatory disease characterized by demyelination and degeneration in the central nervous system, and has caused nontraumatic disabilities in millions of individuals globally. Disease-modifying therapy (DMT) plays an important role in MS management by reducing the occurrence and severity of relapses and postponing disability accumulation (1). Nonetheless, current therapeutic options remain limited and lack targeted specificity, particularly for progressive MS (2). Moreover, side effects such as an increased risk of infection present serious concerns and can lead to nonadherence with long-term DMT treatment (3). Therefore, continuous exploration of drug discovery, development, and repurposing has become increasingly crucial for improving both the efficacy and the safety of MS treatment.

Proteins serve as the main molecular agents of cellular and biological processes and are, therefore, considered highly effective drug targets. Compared to genes and transcripts, proteins as end products play a more direct and influential role in determining phenotypes. Studies have demonstrated that although the human genome contains approximately 20,000 protein-coding genes (4), the number of proteoforms (distinct protein isoforms) could exceed a million (5). Furthermore, the difference between mRNA and protein levels can reach up to 20-30 fold (6). On the other hand, even in the absence of evidence at the genomic or transcriptomic levels, proteins can still contribute to diseases, such as through posttranslational modifications (7). Therefore, direct measurement of proteins not only helps elucidate disease pathology, but also provides potential targets for drug discovery and repurposing (8). Proteomic techniques have been used in MS research since the 2000s and have detected a substantial number of MS-associated proteins (8, 9). However, a challenge that is common to these studies is the limited sample size – the involvement of often less than 100 individuals for a panel of thousands of proteins usually yields a huge statistical burden and inconsistent results.

Integrating proteomic data with genetic information forms an effective strategy to unveil the association between protein expression levels and MS development. This approach has been proven promising, as investigational drugs with supportive evidence from genetics are almost twice as likely to succeed in phase II trials (73 vs. 43%) (10) and to achieve market approval (11). Transcriptomic data provide an additional layer of insights by revealing the effect of mRNA expressions on MS. Transcriptome profile studies have identified candidate drugs across a wide range of diseases (12, 13). Integrating multiomics data, including genomics, transcriptomics, and proteomics with phenotypic information, allows us to prioritize potential drug targets and uncover underlying biological mechanisms.

Complex diseases often manifest in specific tissues and cell types, despite genetic risk variants being present in all cells. By targeting relevant and functional tissues and cell types, trait-associated molecular agents can be more accurately prioritized with the revealed underlying pathological mechanisms. For MS, while plasma proteins reflect circulatory function and are easily accessed in clinical practice, brain proteins inform about MS pathophysiology. Therefore, incorporating plasma and brain proteomes and detecting gene expression of prioritized proteins would aid in identifying disease-relevant signals.

**Fig. 1.**
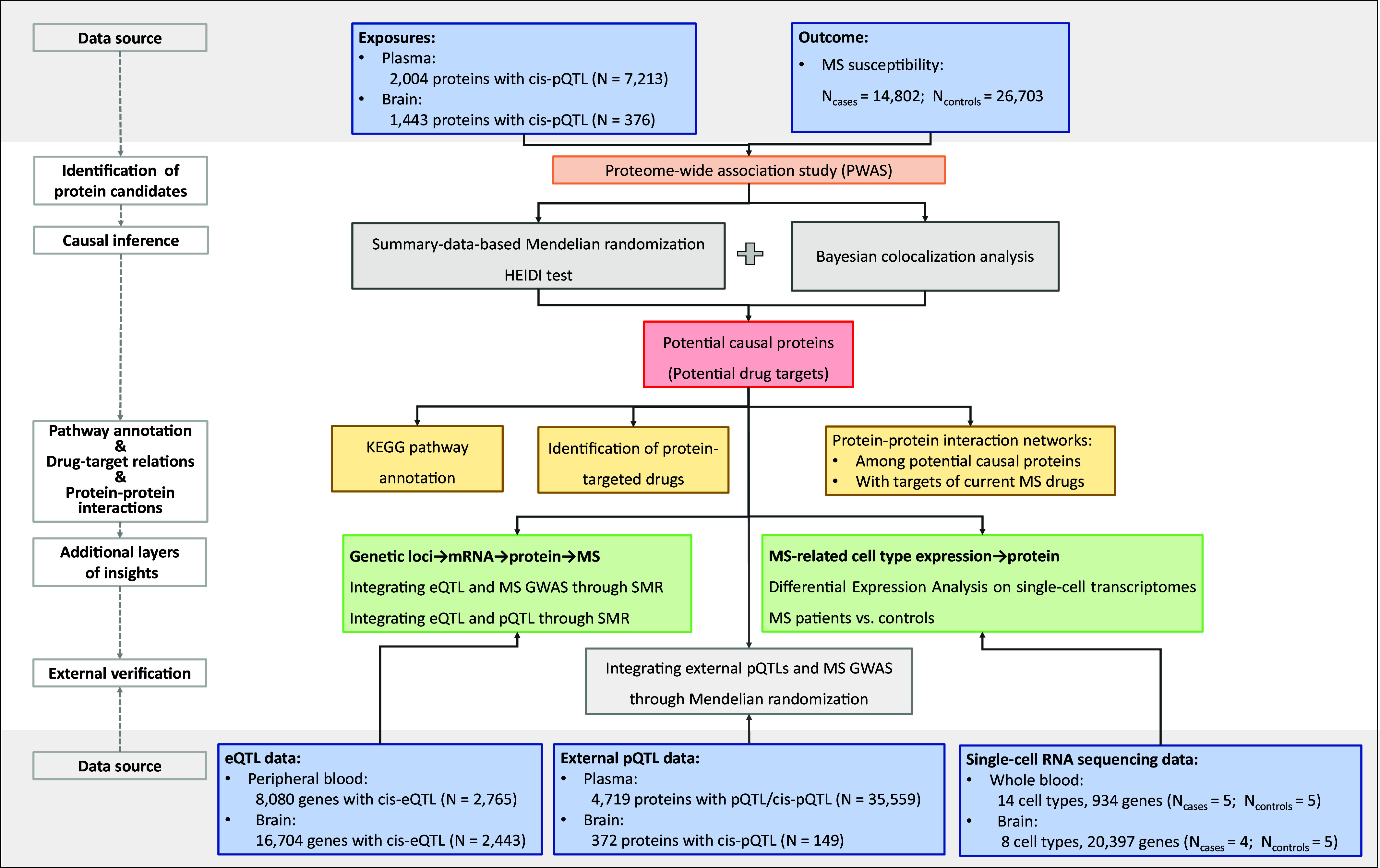
Flowchart of the overall study design. To identify proteins as potential drug targets for multiple sclerosis (MS) treatment, we first conducted a proteome-wide association study (PWAS) to detect proteins associated with MS susceptibility. For candidate proteins identified by PWAS, we conducted a summary-data-based Mendelian randomization (SMR) to test for pleiotropic associations between protein levels and MS. We also performed the HEIDI test and Bayesian colocalization analysis to distinguish pleiotropy from genetic linkage. Proteins that passed all three analyses (SMR analysis + HEIDI test + colocalization analysis) were identified as potential causal proteins. For all detected potential causal proteins, we conducted protein pathway annotation, identified drugs targeting these proteins, and analyzed protein–protein interactions among themselves, as well as interactions with protein targets of existing MS drugs. To gain additional layers of insight, we further investigated mRNA levels of these proteins to assess adherence to the central dogma of molecular biology (genetic locus→mRNA transcription→protein translation→MS) through SMR. Additionally, we investigated whether the genes encoding prioritized proteins are expressed in MS-related cell types through differential expression analysis on single-cell transcriptomes. Finally, we verified our prioritized proteins using external pQTL data through the Mendelian randomization framework. pQTL: protein quantitative trait loci; MS: multiple sclerosis; HEIDI: heterogeneity in dependent instruments test; eQTL: expression quantitative trait loci. GWAS: genome-wide association study.

Previous studies using integrative strategies have identified several promising findings. In plasma, a study identified the proteins FCRL3, TYMP, and AHSG as potential drug targets for MS treatment (14). Other studies identified additional proteins, including CD40, TNFRSF1A, CD58 (15), PLEK, CR1, and CD59 (16, 17). To date, only one study has focused on brain tissue, the primary affected tissue in MS, identifying 34 associated proteins using proteome-wide association study (PWAS) (18). However, the causal relationship and the evaluation of pseudolinks due to SNPs in linkage disequilibrium (LD) remain to be examined in brain tissue. In our study, we conducted an integrative analysis using the hitherto largest protein panel of protein quantitative trait loci (pQTL), together with expression quantitative trait loci (eQTL), single-cell specific expression data, and the largest summary statistics genome-wide association study (GWAS) of MS susceptibility (19–27). We utilized cutting-edge statistical approaches to detect potential causal proteins from complex associations and to distinguish pleiotropy from linkage. By integrating tissue-specific pQTL and MS GWAS data, we prioritized 18 potential drug targets (nine in plasma and nine in brain). Integrating gene expression data, we identified two targets using bulk mRNA expression data and four targets expressed in MS-related cell types. We further found 78 annotated pathways and 16 existing non-MS drugs targeting six of the 18 identified potential targets. We also detected comprehensive protein–protein interactions (PPIs) between 7 of the 18 identified potential drug targets and 19 existing MS drug targets. Moreover, we identified PPIs among four potential drug targets across plasma and brain. Finally, we verified 10 targets using external data (24, 25) under a Mendelian randomization (MR) framework. (Fig. 1).

## Results

### Identifying Candidate Proteins Associated with MS Through PWAS.

**Fig. 2.**
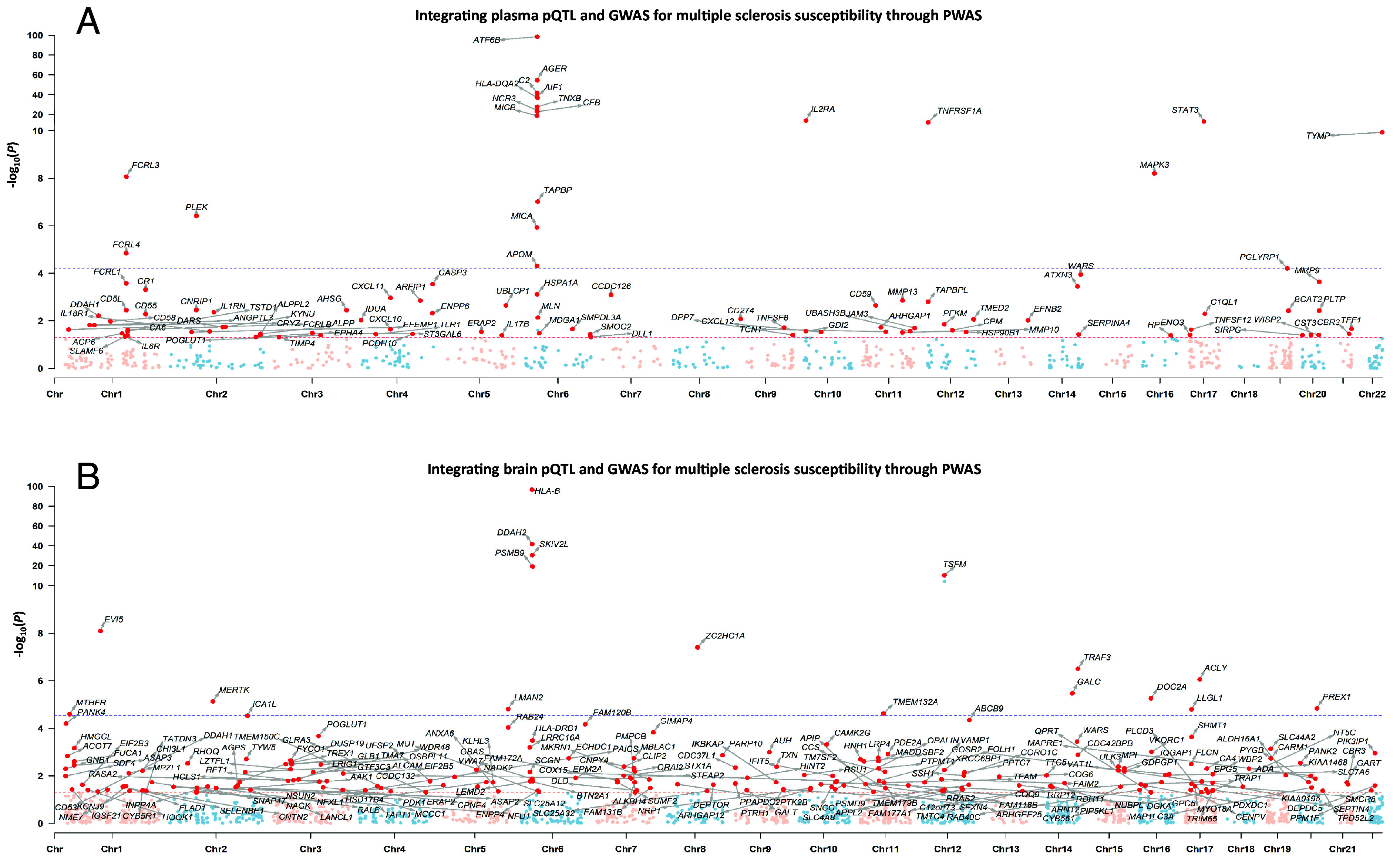
Identifying candidate proteins associated with multiple sclerosis by integrating pQTL and GWAS through PWAS (*A*) Identifying candidate proteins in plasma associated with multiple sclerosis by integrating pQTL and GWAS through PWAS. (*B*) Identifying candidate proteins in brain associated with multiple sclerosis by integrating pQTL and GWAS through PWAS. The *x*-axis represents chromosomes. The *y*-axis represents the negative logarithm of *P*-values. Each dot on the Manhattan plot represents a gene, whose cis-regulated protein expression level was tested in association with the multiple sclerosis risk. The red dashed line indicates a nominal *P*-value threshold of 0.05, and the blue dashed line indicates the Bonferroni-corrected *P*-value threshold of 6.63 × 10^−5^ in plasma and 2.86 × 10^−5^ in brain. Proteins exhibiting nominal significance in association with multiple sclerosis risk were identified as candidate proteins, and their corresponding encoding genes are highlighted with red dots. pQTL: protein quantitative trait loci; GWAS: genome-wide association study; PWAS: proteome-wide association study; Chr: chromosome.

To identify candidate proteins linked to MS, we employed PWAS to establish associations between protein abundances in plasma as well as in brain and MS susceptibility. In plasma, we identified 100 proteins whose expression levels were associated with MS susceptibility (*P*-values < 0.05). After Bonferroni correction, 21 out of these 100 proteins remained significant (Fig. 2*A* and *SI Appendix*, Table S1). Correspondingly, in brain, we identified 212 proteins (*P*-values < 0.05). After Bonferroni correction, 17 out of these 212 proteins remained significant (Fig. 2*B* and *SI Appendix,* Table S2). The 100 plasma proteins and 212 brain proteins under nominal significance were considered as candidates for subsequent causal inference analysis.

### Identifying Proteins Potentially Causally Associated with MS Through SMR and Colocalization.

**Fig. 3.**
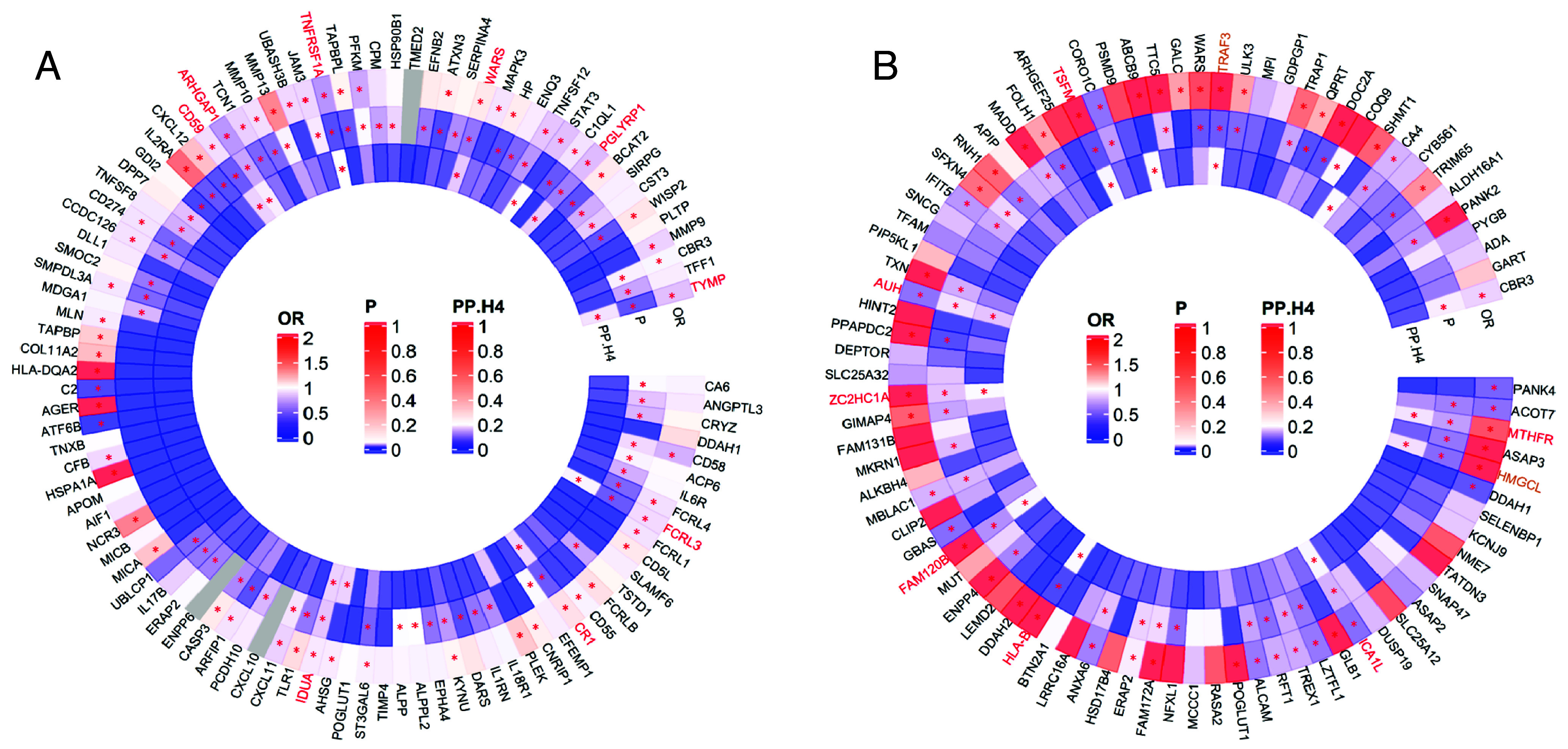
Identifying potential causal proteins for multiple sclerosis through SMR and colocalization analysis, based on candidate proteins determined by PWAS (*A*) Identifying potential causal proteins in plasma for multiple sclerosis through SMR and colocalization analysis, based on candidate proteins determined by PWAS. (*B*) Identifying potential causal proteins in brain for multiple sclerosis through SMR and colocalization analysis, based on candidate proteins determined by PWAS. The corresponding encoding genes for candidate proteins are listed outside the circle. Odds ratios from SMR analyses are presented in the outer circle. *P*-values for HEIDI tests are displayed in the middle circle. The posterior probabilities for colocalization analysis, under the hypotheses of one shared SNP associated with both protein quantitative traits and multiple sclerosis, are shown in the inner circle. The color of boxes in the circles represents the magnitude of each statistic, with warmer colors indicating stronger effects and gray indicating missing values. Significant results are highlighted with red asterisks, defined as Benjamini–Hochberg false discovery rate–adjusted *P* < 0.05 for SMR analysis, *P*_HEIDI_ > 0.05, and posterior probability for colocalization analysis > 0.75. Gene names in red indicate that their corresponding proteins passing all three analyses are identified as potential causal proteins. Gene names in brown indicate that their corresponding proteins are potential causal proteins and adhere to the central dogma of molecular biology. Fig. 3*B* excludes 127 out of 212 PWAS-identified candidate proteins from SMR analysis because their pQTLs had *P*-values > 5 × 10^−8^, not meeting the SMR instrument variable criterion (pQTLs with *P*-values < 5 × 10^−8^). The figure illustrates SMR and colocalization results for the remaining 85 out of 212 candidate proteins. SMR: summary-data-based Mendelian randomization; PWAS: proteome-wide association study; OR: odds ratio; HEIDI: heterogeneity in dependent instruments test. PP.H4: posterior probability for colocalization analysis under the hypotheses of one shared SNP associated with both traits.

We used SMR to infer the causal roles of candidate proteins identified by PWAS. In plasma, we identified 57 proteins whose expression levels were potentially causally associated with MS susceptibility (FDR-adjusted *P*-values < 0.05). Among these proteins, 34 exhibited *P*-value of heterogeneity in dependent instruments (HEIDI) - *P*_HEIDI_ > 0.05, indicating that the associations were likely to be pleiotropic rather than impacted by distinct variants in LD (Fig. 3*A* and *SI Appendix*, Table S3). Using colocalization analysis, we observed 11 proteins with a strong posterior probability of H4 (*PPH4* > 0.75), suggesting a shared causal variant responsible for both plasma protein expression and MS susceptibility, as opposed to distinct causal variants in proximity (Fig. 3*A* and *SI Appendix*, Table S4). Collectively, we identified nine potential causal proteins in plasma. Among these, highly expressed CR1 and WARS were associated with an increased risk of MS, while highly expressed TNFRSF1A, FCRL3, TYMP, PGLYRP1, CD59, IDUA, and ARHGAP1 were associated with a decreased risk of MS (Fig. 3*A* and *SI Appendix*, Fig. S1).

In brain, we first excluded 127 of 212 PWAS-identified candidate proteins from SMR analysis due to their pQTLs having *P-*values > 5 × 10^−8^, failing to meet the SMR instrument variable criterion (pQTLs with *P-*values < 5 × 10^−8^). Consequently, from the remaining 85 PWAS-identified candidate proteins, we identified 48 proteins whose expression levels were potentially causally associated with MS susceptibility (FDR-adjusted *P*-values < 0.05). Among these proteins, 39 successfully passed the HEIDI test with *P*_HEIDI_ > 0.05 (Fig. 3*B* and *SI Appendix*, Table S5), and 11 showed posterior probabilities of *PPH4* > 0.75 via colocalization analysis (Fig. 3*B* and *SI Appendix*, Table S6). Collectively, we identified nine potential causal proteins in brain. Among these, highly expressed HLA-B, ZC2HC1A, HMGCL, TSFM, FAM120B, TRAF3, and MTHFR were associated with an increased risk of MS, while highly expressed ICA1L and AUH were associated with a decreased risk of MS (Fig. 3*B* and *SI Appendix*, Fig. S2).

All prioritized proteins in plasma and brain were located outside of the major histocompatibility complex (MHC) region, with the exception of HLA-B. Utilizing another colocalization algorithm termed HyPrColoc, we verified 17 out of the 18 colocalized genes at a posterior probability > 0.75. The exception was AUH that exhibited only a slightly lower probability (PP.H4 = 0.753 via coloc, PP = 0.737 via HyPrColoc) (*SI Appendix*, Table S7). Flowchart depicting the number of proteins implicated in MS susceptibility at each step of our analytical strategy (PWAS —> SMR/HEIDI + Coloc) is presented in *SI Appendix*, Fig. S3.

To answer the question whether associations with MS at protein expression levels also exhibited similar evidence at gene expression levels, we further explored the causal role of mRNA levels of the 18 identified potential causal proteins. For the nine proteins identified in plasma, mRNA levels were profiled in seven. Among these, SMR identified mRNA levels of five genes potentially causally associated with MS susceptibility (*P_SMR_* < 0.05, *P*_HEIDI_ > 0.05, and exhibiting consistent direction of effects as observed at protein levels) (*SI Appendix*, Table S8). Unfortunately, despite these five genes showing evidence at transcriptional and translational levels for MS separately, SMR linking these two molecular traits failed to identify a relationship such that a genetic locus causally affects MS susceptibility through first modifying mRNA expression then protein translation (*SI Appendix*, Table S9).

For the nine potential causal proteins identified in brain, mRNA levels were profiled in eight. Among these, SMR identified mRNA levels of three genes (*TRAF3, AUH,* and *HMGCL*) to be causally associated with MS susceptibility (*P_SMR_* < 0.05, *P*_HEIDI_ > 0.05, and exhibiting consistent direction of effects as observed at protein levels) (*SI Appendix*, Table S10). Among these three genes with evidence at transcriptional and translational levels for MS respectively, SMR connecting these two molecular traits suggested that rs2076343 in *HMGCL* and rs7145882 in *TRAF3* were causally associated with MS through modifying mRNA expression and protein translation (*SI Appendix*, Fig. S4 and Table S11).

To explore the MS-related cell type origin of these 18 prioritized proteins, we conducted differential expression analysis (DEA) on single-cell transcriptomes measured in MS patients and controls. For plasma proteins, two encoding genes (*ARHGAP1* and *TNFRSF1A*) were detected in the single-cell RNA panel. They exhibited significantly lower levels of expression in three MS-related blood cell types (CD4 T cells, granulocytes, and CD8a T cells) among cases than controls (*SI Appendix*, Table S12). For brain proteins, eight candidate genes were detected in the white matter single-cell RNA panel, with two genes (*AUH* and *ICA1L*) exhibiting significantly lower levels of expression in five brain cell types (oligodendrocyte, oligodendrocyte precursor cell, astrocyte, endothelial cell, and neuron) (*SI Appendix*, Table S12). These findings reveal specific cell types, namely CD4 T cells, CD8 T cells, Granulocytes, and several brain-derived cells, where the prioritized proteins may have a relevant role in MS pathogenesis, validating their significance and highlighting their potential as therapeutic targets.

### Pathway Annotation, Protein-Targeted Drugs Identification, and PPI.

The 18 detected potential causal proteins were defined as potential drug targets, yet none are currently known drug targets for MS treatment. To verify our findings, we further performed comprehensive analyses on pathway annotation, protein-targeted drug identification, and PPI to investigate the underlying biological mechanisms, draggability, and interaction network.

**Fig. 4.**
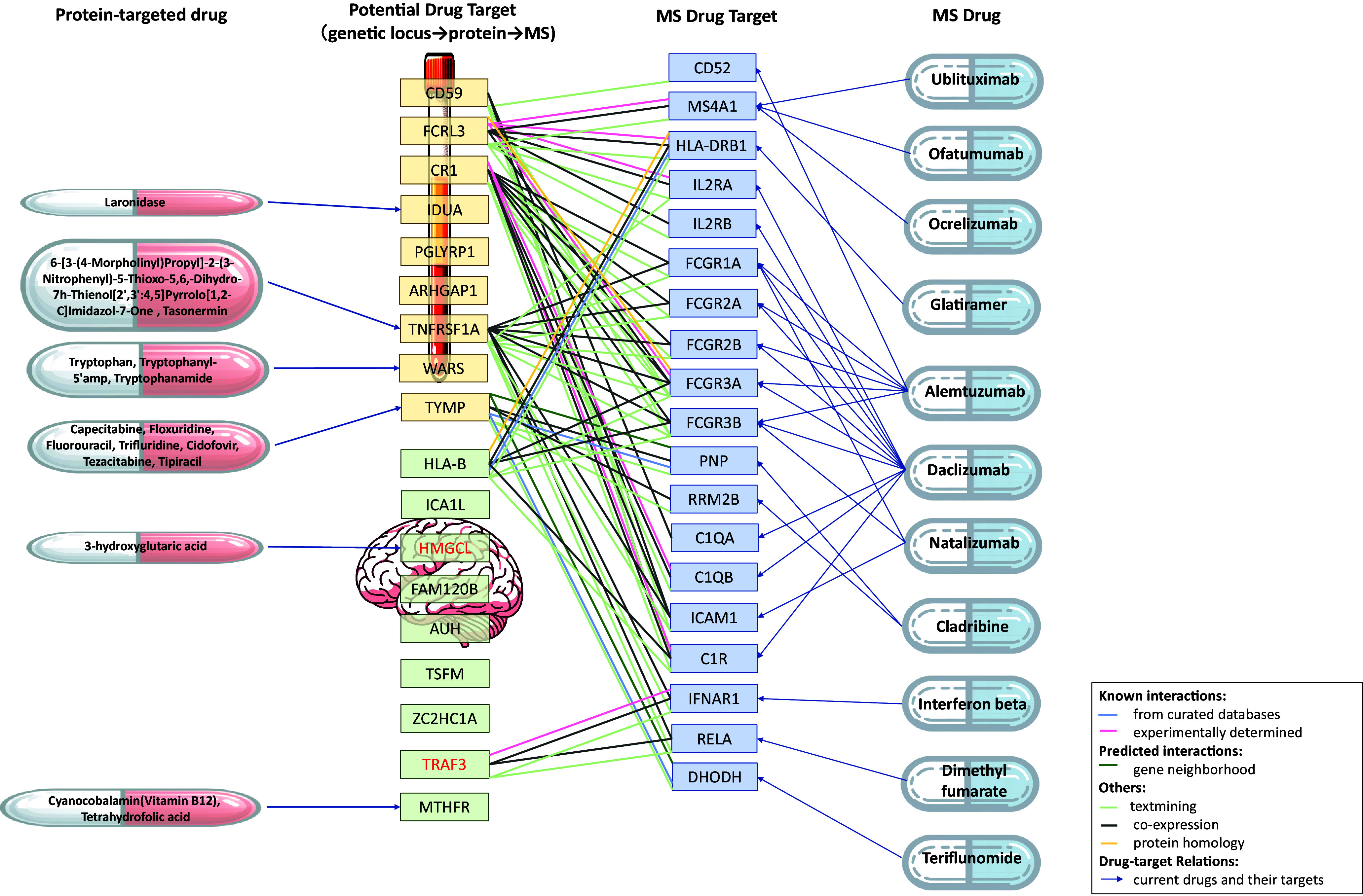
Potential drug targets, protein-targeted drugs, and protein–protein interaction network. The corresponding encoding genes for these potential causal proteins are presented. Gene names in red indicate that their corresponding proteins adhere to the central dogma of molecular biology. Potential drug targets highlighted in yellow boxes are identified in plasma, and those in green boxes are identified in brain. Drug relations refer to the existing drugs targeting the identified potential drug targets. MS drug targets refer to proteins targeted by existing MS drugs. The protein–protein interaction network is presented between potential drug targets and current MS drug targets, with lines of different colors representing various types of interaction. MS: multiple sclerosis; HEIDI: heterogeneity in dependent instruments test.

For the nine plasma drug targets, 56 pathways were annotated (*SI Appendix*, Table S13). An intricate interaction network was observed for five of these targets (CD59, FCRL3, CR1, TNFRSF1A, and TYMP) with 19 known targets of 10 existing MS drugs including *ublituximab, ofatumumab, ocrelizumab, glatiramer, alemtuzumab, natalizumab, cladribine, interferon beta, dimethyl fumarate,* and *teriflunomide*, as well as the previous MS drug *daclizumab*, which is not licensed due to severe liver toxicity (Fig. 4 and *SI Appendix*, Table S14). Moreover, four of these targets (IDUA, TNFRSF1A, WARS, and TYMP) were also targeted by 13 existing non-MS drugs, suggesting potential opportunities for drug repurposing (Fig. 4 and *SI Appendix*, Table S13).

For the nine brain drug targets, 36 pathways were annotated (*SI Appendix*, Table S15). An intricate interaction network was observed for two of these targets (HLA-B and TRAF3) with five known drug targets of six MS drugs, including *glatiramer*, *alemtuzumab*, *interferon beta*, *daclizumab* (not licensed), and *dimethyl fumarate* (Fig. 4 and *SI Appendix*, Table S16). Moreover, two of these targets (HMGCL and MTHFR) were also targeted by three existing non-MS drugs, providing a source of drug repurposing (Fig. 4 and *SI Appendix*, Table S15).

### PPI Analysis Among Identified Potential Drug Targets in Plasma and Brain.

**Fig. 5.**
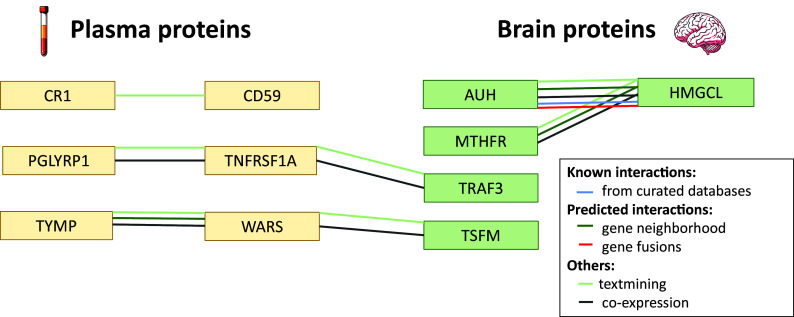
Protein–protein interaction network analysis among all identified potential drug targets in plasma and brain. Protein–protein interaction network analysis was conducted among all detected potential drug targets (nine in plasma and nine in brain). Only those with a minimum required interaction score of 0.4 (six in plasma and five in brain) are considered interactions and presented in this figure. The corresponding encoding genes for these potential causal proteins are presented. Potential drug targets in yellow boxes are identified in plasma, and those in green boxes are identified in brain. The different colored lines connecting these potential drug targets represent various types of interactions.

A PPI network analysis was conducted encompassing all 18 detected potential drug targets (Fig. 5 and *SI Appendix*, Table S17). Among these, six in plasma and five in brain met a minimum interaction score of 0.4. Comprehensive interactions were observed within plasma and brain. Particularly noteworthy were the interactions observed across plasma and brain (TNFRSF1A-TRAF3 and WARS-TSFM).

### Identifying Additional Targets Beyond MS GWAS.

We defined “novel targets” as potential drug targets that were distinct from those tagged by the 551 putative susceptibility genes prioritized in the latest and largest MS GWAS (22). In plasma, six out of nine potential targets were identified as novel targets (CD59, CR1, IDUA, PGLYRP1, ARHGAP1, and WARS), while in brain, three out of nine were novel (ICA1L, FAM120B, and AUH).

### Validating Prioritized Proteins Using MR.

To further strengthen our findings, we validated our results using external pQTL data through the MR approach. For plasma, we confirmed four of nine plasma proteins causally associated with MS (FCRL3, IDUA, TYMP, and WARS) utilizing instrumental variables (IVs) from genome-wide plasma pQTLs. Considering that proteins might exhibit effects in the opposite direction in cis-pQTLs and trans-pQTLs and given that trans-pQTLs act indirectly, we also performed MR analysis restricting IVs to the cis-region (transcription start site ± 500 KB). By involving multiple IVs in cis-region, this approach further verified the causal roles of three proteins (CD59, CR1, and PGLYRP1). Collectively, the causal roles of seven out of nine plasma proteins were verified (*SI Appendix*, Table S18). For brain, we did not find any appropriate genome-wide pQTL data. Instead, brain cis-pQTL profiling was available for three proteins (HMGCL, TSFM, and MTHFR), and MR analysis identified all associated with MS (*SI Appendix*, Table S19).

### Identifying Additional Druggable Proteins Through Relaxed Criteria.

As both HEIDI and colocalization analysis aimed to determine a shared causal variant responsible for multiple traits, to identify an enlarged number of potentially druggable proteins for MS, we relaxed the criteria, requiring passing SMR together with either HEIDI or colocalization test, rather than both HEIDI and colocalization test. A total of 78 proteins (37 in plasma and 41 in brain) successfully passed the relaxed criteria (*SI Appendix*, Tables S20 and S21). Among these, two proteins (CBR3, WARS) were identified in both plasma and brain. Half (39 of 78) of the identified proteins were confirmed by previous epidemiology or laboratory studies, proving the reliability of these findings (*SI Appendix*, Tables S20 and S21). Moreover, 13 of 78 (one in plasma and 12 in brain) proteins adhere to the central dogma of molecular biology (*SI Appendix*, Tables S22–S25).

Of the 37 proteins identified in plasma, 22 were annotated in 127 biological pathways. In total, 17 proteins were targeted on 62 existing non-MS drugs, and 18 proteins showed interaction with 23 known targets of existing MS drugs. Importantly, one of the identified proteins, *IL2RA*, was a known MS drug target (*SI Appendix*, Tables S20 and S26). For the 41 proteins identified in brain, 20 were annotated in 69 biological pathways. In total, 10 proteins were targeted on 45 existing non-MS drugs, and 11 proteins showed interaction with 15 known targets of existing MS drugs (*SI Appendix*, Tables S21 and S27). Intricate PPIs among the 78 proteins were identified (*SI Appendix*, Table S28).

For the 13 potential drug targets adhering to the central dogma of molecular biology, six were annotated in 30 biological pathways. In total, two proteins targeted four existing non-MS drugs, and three proteins interacted with three known targets of existing MS drugs (*SI Appendix*, Fig. S5 and Tables S17 and S18).

## Discussion

We tested hundreds of MS-associated proteins in plasma and brain and confirmed the potential causal role of 18 proteins through comprehensive analytical strategies. None of these are currently known MS drug targets and were, therefore, defined as potential novel drug targets. We revealed intricate interactions between seven of these 18 proteins and 19 known MS drug targets, as well as interactions among 11 of these 18 proteins within and across plasma and brain. Furthermore, we identified 16 existing non-MS drugs targeting six of these 18 potential targets. These findings present significant potential for both the discovery of new drugs and the repurposing of existing ones. The reliability of our findings was further supported by 78 annotated pathways, transcriptional evidence on six targets (two targets using bulk mRNA expression data and four targets expressed in MS-related cell types), and external validation on 10 targets. In the contemporary pharmaceutical industry, the development of new drugs is time-consuming, costly, and highly vulnerable to termination. On average, it takes about 13 y, with costs from hundreds of thousands to over a billion US dollars and a failure rate exceeding 90% before reaching the market (28). Our study integrating large-scale omics data using cutting-edge statistical approaches helps prioritize drug targets by elucidating pathological mechanisms, thus may improve the effectiveness of new drug development (11).

Proteins, as the most common and highly effective drug targets, have been investigated in MS field in two previous integrative studies. Our study largely replicated these findings, successfully confirming five out of the nine previously identified proteins (FCRL3, TYMP, CR1, CD59, and TNFRSF1A). By incorporating the hitherto largest number of proteins (2,004 in plasma and 1,443 in brain) and utilizing PWAS in combination with SMR to reduce statistical testing burden, our study extends previous work by identifying the largest numbers of MS drug targets to date (18 proteins passed stringent criteria and 78 proteins passed relaxed criteria). This significantly advances our understanding of MS pathology and enhances the potential for future drug development and functional studies. Considering the pathogenesis of MS in the central nervous system, our study also identified proteins in brain tissue. These potential causal brain proteins advance the associative findings of the previous study (18), thereby expanding the treatment perspective directly to the most relevant tissue. Another advantage of our research is the involvement of bulk and single-cell transcriptional evidence for the identified potential drug targets, providing opportunities for further studies to enhance the understanding of the MS molecular mechanisms. Notably, two targets showed altered expression levels through bulk mRNA expression, and four targets were differentially expressed in MS-related cell types, greatly strengthening their credibility for further clinical applications.

We prioritized seven potential drug targets with identified biological relevance connected to known MS drug targets. As FCRL3, TNFRSF1A, CR1, CD59, and TYMP have already been discovered and confirmed by the previous integrative studies (14, 15), we, therefore, emphasize two novel findings. **HLA-B** is an MHC class I molecule presenting antigens to CD8+ T cells. In alignment with our findings, protective effects of *HLA-B* alleles against MS susceptibility have been observed with HLA-B*44:02, HLA-B*38:01, and HLA-B*55:01(29). *HLA-B* has been annotated to several pathways, such as natural killer cell mediated cytotoxicity (30), human cytomegalovirus infection (31), and Epstein–Barr virus infection (32), all of which have been implicated in MS development. Our study identified strong interactions between HLA-B and HLA-DRB1, the major MS susceptibility gene and a known target of *Glatiramer*, supporting the complex involvement of both proteins in relevant MS processes and suggesting HLA-B as a potential target. **TRAF3** regulates the NF-kappa-B pathway (33) which mediates inflammation in MS (34). TRAF3 is part of other annotated pathways, such as NOD-like receptor signaling, RIG-I-like receptor signaling, IL-17 signaling, and TNF signaling, all known to be altered in autoimmune diseases and MS development (35–37). Previous studies have shown that TRAF3 degradation promotes microglia-mediated CNS inflammation (38), and TRAF3 haploinsufficiency syndrome exhibits B cell hyperactivity leading to hypergammaglobulinemia and autoimmunity (39).

The 11 novel potential drug targets that do not interact with known MS drug targets represent opportunities for developing new therapeutic strategies. Here, we highlight two potential drug targets with strong supporting evidence. **IDUA** is responsible for the degradation of glycosaminoglycans heparan sulfate and dermatan sulfate, and, therefore, it is involved in pathways of glycosaminoglycan degradation (40), metabolic pathways (41), and lysosome function (42), which have been proven to play a pivotal role in MS development, underscoring IDUA as a promising potential target. **PGLYRP1** is an innate immunity protein that functions in antimicrobial and antitumor defense systems. Although no pathway is currently annotated to *PGLYRP1*, a study demonstrating PGLYRP1 as a proinflammatory molecule in myeloid cells during autoimmune conditions (43) has proven its potential in immunotherapy. Additionally, elevated levels of PGLYRP1 protein have been observed in the white and gray matter in the cerebellum and spinal cord of patients with MS (44).

Drug repurposing accelerates the translation of scientific discoveries into clinically beneficial treatments. Among the 16 identified non-MS drugs, we first would like to highlight eight existing non-MS drugs including *Tasonermin*, *Tipiracil*, *Capecitabine*, *Floxuridine*, *Fluorouracil*, *Tezacitabine*, *Trifluridine*, and *Cidofovir*, the targets of which interact with known MS drug targets, suggesting the possibility for these non-MS drugs to benefit or assist the treatment of MS. Among these non-MS drugs, the first six possess cancer-therapeutic properties, and the last two are antiviral agents. However, these non-MS drugs need prescriptions and usually associated with higher toxicity or stronger side effects compared to over-the-counter drugs, challenging their repurposing as MS treatments. Next, we would like to highlight vitamin B12 and tetrahydrofolic acid (a folic acid derivative), both of which are easily accessible and cost-effective vitamin supplements, targeting the protein **MTHFR** (a potential MS drug target). Studies have demonstrated that both vitamin B12 and folate improve myelin regeneration and possess neuroprotective and immunomodulatory properties (45, 46). Moreover, supplementing vitamin B12 and folate may help reduce homocysteine levels (47, 48), a neuro- and vascular-toxic sulfur-containing intermediary product that has been proven to be elevated in MS patients(49). Although some studies have provided inconsistent findings (50, 51), a clinical study has demonstrated that supplementation with folic acid and vitamin B12 improves the quality of life in MS patients (47). Considering the outcome of our study on MS susceptibility, supplementation with vitamin B12 and folic acid may serve as prophylactic agents against the onset of MS. Additionally, combining vitamin B12 and folic acid with DMTs may offer a more effective approach for MS management.

The interactions of potential drug targets across plasma and brain suggest the possibility of substance transport or signaling molecules across blood and brain, which could indicate that targets identified in plasma may influence MS pathology in the brain. Based on relaxed criteria, we identified two potential proteins (**CBR3** and **WARS**) present in both plasma and brain tissue, suggesting the possibility of a link between systemic circulation and neurological processes.

The interpretation of our study should be approached with caution. First, not every protein has pQTLs. For example, in our referenced plasma pQTL study, less than half of the proteins (45%, 2,004 out of 4,435) possessed significant cis-pQTLs (19). Consequently, using pQTLs in integrative analysis, rather than relying on quantitative proteomic analysis, results in the omission of proteins. Nevertheless, integrative analysis allows for causal inferences, representing an improvement over observational association design. Furthermore, our study has identified the hitherto largest number of potential MS drug targets, owing to the large protein panel of pQTL data and large sample size, representing an advantage over the small sample sizes that usually characterize the current quantitative proteomic analysis. For example, our study identified more MS-determined proteins than a previous proteomics study, which quantified proteins in cerebrospinal fluid and plasma from 143 MS patients and 43 healthy controls using the Olink technique (52). Alternatively, these proteomics studies, which directly assay proteins in MS patients, could strongly strengthen our computational findings. For instance, FCRL3 decreased in plasma (*P* = 0.04) (52). Second, six of the 18 potential drug targets are also supported by transcriptional levels. The absence of transcriptional evidence for most drug targets could be due to posttranscriptional regulation and modification, which, from another aspect, highlights the importance and superiority of a direct proteome investigation. Third, while SMR analysis with the HEIDI test and colocalization can distinguish linkage from pleiotropy, differentiating between vertical (causality) and horizontal pleiotropy remains challenging. We, therefore, leveraged genome-wide pQTLs data in plasma and performed MR analysis with multiple independent genetic instruments. After accounting for directional pleiotropy using the MR-Egger intercept test, we confirmed the putative causal roles of 7 out of the 9 plasma proteins. Nonetheless, causal inference should be interpreted with caution. Therefore, we describe these prioritized proteins as “potential causal proteins” that require functional validation in future studies. Fourth, the prioritized MS-linked proteins in plasma, such as FCRL3 and CD59, are most likely in soluble form rather than membrane-bound form. To determine whether disease pathology is driven by an overall increase in these proteins or a shift toward their secreted forms and how this impacts drug targeting, it is important to uncover the complexity and cellular process leading to their differential expression using single-cell proteomics and functional experiments in both in vivo and in vitro approaches.

## Materials and Methods

### Plasma pQTL Summary Data.

Summary statistics of plasma pQTL were obtained from a large-scale community-based cohort study involving 7,213 participants of European ancestry (19). Relative plasma protein concentrations were measured using modified aptamers (SOMAmer). Following quality control for protein–gene mapping, 4,657 SOMAmers with tagged proteins encoded by 4,435 genes were identified. Genotyping was conducted using the Affymetrix 6.0 DNA microarray and subsequently imputed with the TOPMed reference panel. After quality control for imputation quality, Hardy–Weinberg equilibrium, and minor allele frequencies (MAF), 6,181,856 SNPs were identified.

To identify pQTL, a linear regression model was performed, adjusting for age, sex, study site, and ten genetic principal components (PCs). Cis-regions were defined as ±500 KB of the transcription start site. In total, 2,004 significant proteins with cis-pQTLs were identified.

In addition, summary statistics of plasma pQTL for 4,719 proteins (including 1,881 cis-pQTLs and 16,203 trans-pQTLs, N = 35,559) were used for validation (24).

### Brain pQTL Summary Data.

Summary statistics of brain pQTL were obtained from two clinical-pathologic cohort studies using the dorsolateral prefrontal cortex of postmortem brain samples donated by 400 participants of European ancestry (21). The brain proteome was profiled using liquid chromatography coupled to mass spectrometry analysis. Following quality control for outliers, missing values, and protein loadings, 8,356 brain proteins with corresponding quantitation were detected. Genotyping was conducted either by whole-genome sequencing or by genome-wide genotyping using Illumina OmniQuad Express or Affymetrix GeneChip platforms. Imputation was performed using the 1,000 Genomes (1 KG) reference panel. After quality control on variants missing rate, Hardy–Weinberg equilibrium, imputation quality, MAF, and degree of kinship, 1,190,321 SNPs on autosomes matching the HapMap LD reference panel were identified.

To determine pQTL, a linear regression model was performed. Cis-regions were defined as a genomic window of 1 MB (±500 KB) around genes. In total, 1,443 significant proteins with cis-pQTL were identified.

In addition, summary statistics of brain cis-pQTLs for 372 proteins (N = 149) were used for validation (25).

### GWAS of MS Susceptibility.

The largest MS susceptibility GWAS was performed on 47,429 MS and 68,374 controls (22). The overall sample was divided into discovery and replication stages. In the discovery stage, 14,802 MS cases and 26,703 non-MS controls of individuals of European ancestry were involved. Each of the 15 participating datasets underwent quality checks, followed by imputation using BEAGLE or 1 KG reference panel. Subsequently, the association between genetic variants and MS risk was estimated for each dataset through logistic regression, adjusting for the first five PCs. A fixed-effect meta-analysis was conducted to pool the results. Replication was performed on 4,842 prioritized SNPs (from the discovery stage) using the designed MS Chip SNPs among 20,360 MS and 19,047 controls, as well as targeted genotyping using the ImmunoChip platform among 12,257 MS and 22,625 controls. Only the summary statistics of MS susceptibility from the discovery stage were genome-wide as well as were accessible (https://imsgc.net/), which were utilized in our study.

### Blood eQTL Summary Data.

Summary statistics of peripheral blood eQTL were obtained from the Consortium for the Architecture of Gene Expression, using whole blood of 2,765 individuals of European ancestry (23). Gene expression quantification was conducted using Illumina Whole-Genome Expression BeadChips. After quality control on standardizing the expression levels across samples and adjusting for covariates, the mRNA levels for 36,778 transcript expression traits (probes) were investigated. Genotyping was conducted using various platforms and subsequently imputed using the 1 KG reference panel. After standard quality control on variants missing rate, Hardy–Weinberg equilibrium, imputation quality, and MAF, 7,763,174 autosomal SNPs were detected.

To identify SNP-probe associations, a linear mixed regression model was performed, adjusting for population structure. Subsequently, Conditional & joint association analysis was conducted to refine the extensive set of identified SNP-probe association results. In total, 11,204 cis-eQTLs for 8,080 genes were identified.

### Brain eQTL Summary Data.

Summary statistics of brain eQTL were accessed from seven cohorts using brain cortex samples donated by 2,443 individuals of European ancestry (20). Gene-level transcriptional abundances were quantified using RNA-SeQC. After quality control, retaining individuals with over 10 million reads and an RNA integrity number > 5.5, RNA-seq data from 2,865 brain cortex samples were retained. Genotyping was conducted using various platforms and imputed using the 1 KG reference panel and then filtered with standard quality control. In total, 11,631,763 SNPs were detected.

To identify brain eQTL, a linear regression model was performed, adjusting for five genetic PCs. A meta-analysis was then conducted to pool the results across cohorts. In total, 1,962,048 cis-eQTLs for 16,704 genes were identified.

To the best of our knowledge, participants in MS GWAS did not overlap with those involved in the eQTL/pQTL data we introduced.

### Statistical Analysis.

#### PWAS identifying proteins associated with MS, by integrating pQTL and MS GWAS.

We first conducted a PWAS to identify candidate proteins associated with MS. FUSION was performed using precomputed elastic net model-based weights for both plasma and brain protein expressions (19, 21). Briefly, the 1 KG reference panel was used to reduce the impact of LD on MS GWAS. Subsequently, SNP effect sizes (z-scores) of MS GWAS were imputed using the ImpG-Summary algorithm (53). Finally, the PWAS effect size for each protein was computed as a weighted linear combination of GWAS SNP-level Z-scores while accounting for LD among SNPs (54). To identify the largest possible number of candidates, a nominal *P*-value < 0.05 was utilized as a significance threshold. Moreover, Bonferroni correction was also applied, with a corrected threshold of 6.63×10^-5^ in plasma and 2.86×10^-5^ in brain.

#### SMR identifying proteins potentially causally associated with MS.

To confirm whether PWAS-identified protein candidates potentially causally influence MS, SMR analysis was performed (55). This method tests the pleiotropic association between genetically predicted protein levels and MS onset. A key assumption of this approach is that an identical underlying causal variant determines both protein expression and disease phenotype. However, due to LD, it is possible that the SMR effect could be nonzero even when this assumption is violated. To distinguish pleiotropy from linkage, HEIDI test was performed, under the assumption that if protein expression and disease phenotype are affected by a shared causal variant, β_SMR_ would be the same for any variant in LD with that causal variant. Thus, greater heterogeneity among β_SMR_ statistics calculated for all cis-pQTLs implies a greater likelihood of linkage, rather than pleiotropy.

SMR and HEIDI analyses were performed using SMR software (version 1.3.1). The 1 KG reference panel was used for LD estimation. Common variants with MAF > 0.01 were involved in the analysis. Briefly, for the SMR test, only pQTLs that met the significance threshold of *P*-value < 5 × 10^−8^ were involved. For HEIDI test, the significance threshold was set to a *P*-value < 1.57×10^−3^; pQTLs were required to correlate with the top-associated cis-pQTL with an *R^2^* of 0.05-0.90; and the number of involved SNPs was restricted to 3-20. The statistical significance threshold for putative causal proteins was defined using FDR-adjusted *P_SMR_* < 0.05 and *P_HEIDI_* > 0.05 (55).

#### Bayesian colocalization analysis examining shared causal variant influence on both protein expression and MS.

To further address the potential effects of LD on the pleiotropic associations, Bayesian colocalization analysis was performed to assess the posterior probability of shared genetic variants being responsible for both protein expression and MS development using the coloc R package (version 5.2.3). In this approach, the posterior probability is generated for the following hypotheses: H0: neither protein expression level nor MS development has a genetic association in the region; H1: only protein expression level has a genetic association in the region; H2: only MS development has a genetic association in the region; H3: both protein expression level and MS development have genetic association but with different causal variants; H4: both protein expression level and MS development have genetic association in the region and share a single causal variant. Conventionally, a *PPH4* ranging from 0.5 to 0.8 was considered appropriate. In this study, a *PPH4* > 0.75 was considered colocalized (56, 57).

To validate the findings of colocalization, we applied HyPrColoc (R package, Version 0.0.2) using the same priors as in coloc analysis (prior.1 = 1e-04, prior.c = 1e-04, prior.12 = 1e-05). Colocalization was considered significant at posterior probability > 0.75.

#### Definition of potential causal proteins.

Potential causal proteins were defined as those that successfully passed all three analyses: the SMR analysis (FDR-adjusted *P_SMR_* < 0.05), the HEIDI test (*P_HEIDI_* > 0.05), and the colocalization analysis (*PPH4* > 0.75). These potential causal proteins were recognized as potential drug targets and underwent further comprehensive functional investigations. Regional visualization plots of these potential causal proteins were performed through SMR portal (https://yanglab.westlake.edu.cn/smr-portal/viewer) to integrate QTL and GWAS signals with neighboring gene annotations.

#### Identifying novel targets beyond MS GWAS.

To provide additional intriguing insights beyond the previously known signals, we defined novel targets as those distinct from the 551 putative susceptibility genes prioritized by the largest MS GWAS (22).

#### KEGG pathway annotation, protein-targeted drugs identification, and PPI network analysis.

To interpret functional implications, KEGG pathway annotation was performed for all detected potential causal proteins (https://www.genome.jp/kegg/pathway.html). Existing drugs targeting each of the identified potential causal proteins as well as existing MS drug targets were determined by reviewing the Drugbank database (https://go.drugbank.com/). To investigate the interactions between potential drug targets and the known MS drug targets, and to explore the interactions of potential drug targets within and across plasma and brain, PPI network analysis was conducted using the Search Tool for the Retrieval of Interacting Genes database (https://string-db.org/). Only interaction scores greater than 0.4 were shown.

#### SMR identifying mRNA level of detected proteins.

To increase the biological interpretability of the identified potential causal proteins, we further examined their mRNA expression levels, investigating compliance with the central dogma of molecular biology. First, for identified potential causal proteins, the corresponding mRNA levels were tested in relation to MS through SMR analysis (integrating cis-eQTL and MS GWAS). Subsequently, for significant MS-associated mRNA expressions, the links between mRNA expressions and protein expressions were investigated through SMR analysis (integrating cis-eQTL and cis-pQTL). The significance threshold was set to *P_SMR_* < 0.05, and probes with *P_HEIDI_* < 0.05 were excluded.

#### DEA identifying cell-type specific gene expression of prioritized proteins.

To investigate whether the genes encoding prioritized proteins are expressed in MS-related cell types, we performed DEA between MS patients and controls in single-cell transcriptomes. For plasma proteins, single-cell transcriptomics data from whole blood samples of five MS patients and five controls were utilized (27). DEA was originally performed in this study across 14 blood cell types using the Bayes factor method and the EdgeR method. For brain proteins, we utilized single-nucleus RNA sequencing data from the white matter area in the brain of four progressive MS patients and five controls (26). After quality control and normalization, we performed DEA across eight brain cell types using the Wilcoxon Rank Sum test. Multiple testing correction was applied using the FDR, adjusting for all genes within the specific cell type following a similar approach as in the blood DEA. Significance threshold was set at FDR-adjusted *P* < 0.05. To ensure biologically meaningful results, we applied an absolute average log2 fold change > 0.10, indicating at least a 10% differential expression of a gene in the target cell type compared to the reference group. Only genes exhibiting significant differential expression with a consistent direction of effect at both transcriptomic and proteomic levels were retained.

#### Validating prioritized proteins using MR.

To ensure the robustness of the results, we verified our prioritized pleiotropy proteins using external pQTL data through MR analysis. Independent IVs were identified based on genome-wide significance and subsequently clumped with an *r^2^* threshold of 0.1 within a ± 500 KB window. The inverse variance weighted (IVW) approach and weighted median (WM) approach were performed. Additionally, the MR-Egger intercept test was used to reflect directional pleiotropy. For proteins incorporating only one independent instrument, the Wald ratio and robust adjusted profile score (MR-RAPS) were applied. All analyses were conducted with packages “TwoSampleMR”, “MRInstruments”, “MendelianRandomization”, and “mr.raps” in R v3.6.3. Significance was defined as a *P*-value < 0.05 in either IVW or WM (for single instrument: either Wald ratio or MS-RAPS) with a consistent direction of effect across both approaches. The MR-Egger intercept with a *P*-value < 0.05 was used to indicate directional pleiotropy.

### Ethics.

This study was a secondary analysis of existing, publicly available summary-level GWAS and QTL data. The statement of ethics for each research can be found elsewhere, approved by the relevant institutional review board or an equivalent committee (19–23). Consent to participate: not applicable.

## Supplementary Material

Appendix 01 (PDF)

## Data Availability

All the genetic and QTL data used in this study were from the publicly accessible summary statistics and can be accessed through the corresponding references presented in the main text (19–25). All other data are included in the manuscript and/or *SI Appendix*.
