# Supplementary material for: Multiomics integration prioritizes potential drug targets for multiple sclerosis
Source: Proc Natl Acad Sci U S A. 2025 Jun 27;122(26):e2425537122. doi: 10.1073/pnas.2425537122 (PMC12232717; doi:10.1073/pnas.2425537122)
Supplement: Supplementary file 1 — Appendix 01 (PDF) [file pnas.2425537122.sapp.pdf]

PNAS

**Supporting Information for**

# Multi-omics integration prioritizes potential drug targets for multiple sclerosis

Yuan Jiang, Qianwen Liu , Pernilla Stridh, Ingrid Kockum, Tomas Olsson, Lars Alfredsson, Lina-Marcela Diaz-Gallo, Xia Jiang

Xia Jiang
Email: xia.jiang@ki.se

**This PDF file includes:**

Tables S1 to S28
Figures S1 to S5

| Supplementary Tables |
|---|
| Table S1. PWAS identifying plasma proteins associated with multiple sclerosis, through integrating pQTL and MS GWAS |
| Table S2. PWAS identifying brain proteins associated with multiple sclerosis, through integrating pQTL and MS GWAS |
| Table S3. SMR analysis identifying plasma proteins causally associated with multiple sclerosis, based on candidate proteins determined by PWAS |
| Table S4. Colocalization analysis investigating whether the same causal variant is responsible for both plasma protein expression and multiple sclerosis |
| Table S5. SMR analysis identifying brain proteins causally associated with multiple sclerosis, based on candidate proteins determined by PWAS |
| Table S6. Colocalization analysis investigating whether the same causal variant is responsible for both brain protein expression and multiple sclerosis |
| Table S7. Evaluating posterior probability in different colocalization approaches |
| Table S8. SMR analysis identifying gene expression of potential causal proteins in plasma, through integrating eQTL and MS GWAS |
| Table S9. SMR analysis examining if both gene expression and protein expression in plasma are causally affected by a single variant, through integrating eQTL and plasma pQTL |
| Table S10. SMR analysis identifying gene expression of potential causal proteins in brain, through integrating eQTL and MS GWAS |
| Table S11. SMR analysis examining if both gene expression and protein expression in brain are causally affected by a single variant, through Integrating eQTL and brain pQTL |
| Table S12. Single cell-specific differential gene expression of prioritized proteins |
| Table S13. For all detected potential causal proteins in plasma: pathway annotation, protein-targeted drugs, and evidence in previous studies |
| Table S14. Protein-protein interaction analysis of all detected potential causal proteins in plasma with targets of current multiple sclerosis drugs (minimum combined score of 0.4) |
| Table S15. For all detected potential causal proteins in brain: pathway annotation, protein-targeted drugs, and evidence in previous studies |
| Table S16. Protein-protein interaction analysis of all detected potential causal proteins in brain with targets of current multiple sclerosis drugs (minimum combined score of 0.4) |
| Table S17. Protein-protein interaction analysis among all detected potential causal proteins (minimum combined score of 0.4) |
| Table S18. Associations between genetically predicted plasma protein levels and MS risk: a Mendelian randomization study |
| Table S19. Associations between genetically predicted brain protein levels and multiple sclerosis risk: a Mendelian randomization study |
| Table S20. For all relaxed-criteria-based potential causal proteins in plasma: pathway annotation, protein-targeted drugs, evidence in previous studies |
| Table S21. For all relaxed-criteria-based potential causal proteins in brain: pathway annotation, targeted existing drugs, and evidence in previous studies |
| Table S22. SMR analysis identifying gene expression of relaxed-criteria-based plasma proteins, through integrating eQTL and MS GWAS |
| Table S23. SMR analysis examining if both gene expression and relaxed-criteria-based plasma protein expression are causally affected by a single variant, through integrating eQTL and plasma pQTL |
| Table S24. SMR analysis identifying gene expression of relaxed-criteria-based brain proteins, through integrating eQTL and MS GWAS |
| Table S25. SMR analysis examining if both gene expression and relaxed-criteria-based brain protein expression are causally affected by a single variant, through integrating eQTL and plasma pQTL |
| Table S26. Protein-protein interaction analysis of relaxed-criteria-based plasma proteins with multiple sclerosis drug targets (minimum combined score of 0.4) |
| Table S27. Protein-protein interaction analysis of relaxed-criteria-based brain proteins with multiple sclerosis drug targets (minimum combined score of 0.4) |
| Table S28. Protein-protein interaction analysis among all relaxed-criteria-based potential causal proteins (minimum combined score of 0.4) |

**Table S1. PWAS identifying plasma proteins associated with multiple sclerosis, through integrating pQTL and MS GWAS**

| Gene | Chr | P0 | P1 | HSQ | BEST.GWAS.ID | BEST.GWAS.Z | PQTL.ID | PQTL.R2 | PQTL.Z | PQTL.GWAS.Z | $N_{SNP}$ | NWGT | MODEL | MODELCV.R2 | MODELCV.PV | PWAS.Z | PWAS.P |
|---|---|---|---|---|---|---|---|---|---|---|---|---|---|---|---|---|---|
| ATF6B | 6 | 32083041 | 32096030 | 0.174 | rs182547608 | 33.920 | rs6449 | 0.030 | 14.900 | -27.995 | 86 | 86 | enet | 0.054 | 9.80E-90 | -21.139 | 3.46E-99 |
| AGER | 6 | 32148745 | 32152099 | 0.113 | rs7197 | -37.070 | rs2070600 | 0.049 | -18.860 | -13.508 | 17 | 17 | enet | 0.049 | 3.40E-81 | 15.661 | 2.78E-55 |
| C2 | 6 | 31865562 | 31913449 | 0.221 | rs9268149 | -37.070 | rs115204832 | 0.031 | -16.560 | 3.737 | 137 | 137 | enet | 0.120 | 2.10E-204 | -13.655 | 1.89E-42 |
| AIF1 | 6 | 31582994 | 31584798 | 0.045 | rs6937318 | -27.370 | rs3130623 | 0.009 | 9.370 | -1.217 | 50 | 50 | enet | 0.027 | 8.00E-46 | 12.933 | 2.93E-38 |
| HLA-DQA2 | 6 | 32709168 | 32714975 | 0.177 | rs3129880 | -37.070 | rs9272014 | 0.149 | -32.900 | -14.987 | 83 | 83 | enet | 0.240 | 0.00E+00 | -12.797 | 1.70E-37 |
| TNXB | 6 | 32008930 | 32083111 | 0.391 | rs9267573 | -33.840 | rs116298992 | 0.125 | -30.110 | 0.108 | 100 | 100 | enet | 0.250 | 0.00E+00 | -11.066 | 1.82E-28 |
| NCR3 | 6 | 31556672 | 31560783 | 0.276 | rs2523508 | -24.220 | rs986475 | 0.066 | -21.800 | -8.424 | 107 | 107 | enet | 0.110 | 5.60E-188 | 10.326 | 5.39E-25 |
| CFB | 6 | 31913427 | 31919861 | 0.232 | rs9267658 | -34.470 | rs541862 | 0.113 | 28.590 | -4.703 | 80 | 80 | enet | 0.210 | 0.00E+00 | -10.009 | 1.39E-23 |
| MICB | 6 | 31462658 | 31478901 | 0.433 | rs389883 | -29.600 | rs3094011 | 0.163 | -34.420 | 1.625 | 159 | 159 | enet | 0.360 | 0.00E+00 | -8.999 | 2.28E-19 |
| TAPBP | 6 | 33267471 | 33282164 | 0.331 | rs3132131 | -19.090 | rs1014779 | 0.100 | -27.110 | -5.428 | 122 | 122 | enet | 0.170 | 4.70E-303 | 5.333 | 9.64E-08 |
| MICA | 6 | 31367561 | 31383092 | 0.412 | rs2855811 | -23.790 | rs2523496 | 0.334 | -49.080 | -10.551 | 158 | 158 | enet | 0.500 | 0.00E+00 | 4.858 | 1.18E-06 |
| APOM | 6 | 31620193 | 31625987 | 0.100 | rs2856448 | -27.570 | rs805264 | 0.044 | -18.050 | -0.232 | 40 | 40 | enet | 0.051 | 1.20E-83 | 4.061 | 4.89E-05 |
| HSPA1A | 6 | 31783320 | 31785723 | 0.018 | rs35609644 | 34.220 | rs74434374 | 0.006 | -7.140 | -5.364 | 56 | 56 | enet | 0.009 | 4.10E-16 | 3.364 | 7.68E-04 |
| MLN | 6 | 33762450 | 33771780 | 0.074 | rs62405888 | 5.510 | rs9394169 | 0.064 | -23.050 | 2.331 | 51 | 51 | enet | 0.100 | 8.70E-168 | -2.682 | 7.32E-03 |
| SMPDL3A | 6 | 123110194 | 123130865 | 0.106 | rs13192569 | 2.341 | rs28385609 | 0.058 | -20.540 | 2.336 | 11 | 11 | enet | 0.063 | 2.00E-104 | -2.290 | 2.20E-02 |
| COL11A2 | 6 | 33130458 | 33160276 | 0.211 | rs7767167 | -37.070 | rs3129205 | 0.065 | 21.990 | 5.638 | 75 | 75 | enet | 0.160 | 2.20E-282 | 2.238 | 2.52E-02 |
| MDGA1 | 6 | 37598455 | 37667082 | 0.574 | rs9296221 | 2.979 | rs9349050 | 0.500 | -60.110 | 1.840 | 173 | 173 | enet | 0.550 | 0.00E+00 | -2.126 | 3.35E-02 |
| SMOC2 | 6 | 168841831 | 169073984 | 0.164 | rs4708637 | 2.440 | rs56296467 | 0.088 | -25.220 | -1.186 | 122 | 122 | enet | 0.160 | 1.00E-277 | 2.089 | 3.67E-02 |
| DLL1 | 6 | 170591294 | 170615653 | 0.054 | rs78154159 | -4.089 | rs4710790 | 0.014 | 10.450 | -1.069 | 51 | 51 | enet | 0.033 | 2.20E-54 | -1.979 | 4.79E-02 |
| TNFRSF21 | 6 | 47199268 | 47277641 | 0.108 | rs9473022 | 2.045 | rs6458555 | 0.036 | 16.250 | -0.361 | 92 | 92 | enet | 0.071 | 1.90E-118 | -1.555 | 1.20E-01 |
| GFRAL | 6 | 55192267 | 55267291 | 0.096 | rs9475311 | 2.681 | rs73439277 | 0.014 | 12.130 | 0.458 | 127 | 127 | enet | 0.069 | 7.00E-115 | 1.503 | 1.33E-01 |
| BTN3A3 | 6 | 26440700 | 26453643 | 0.458 | rs111884694 | -3.191 | rs9393711 | 0.258 | -43.200 | 2.944 | 116 | 116 | enet | 0.470 | 0.00E+00 | -1.482 | 1.38E-01 |
| ENPP5 | 6 | 46126922 | 46138717 | 0.335 | rs74918287 | 3.409 | rs1047153 | 0.293 | -46.000 | -2.506 | 74 | 74 | enet | 0.460 | 0.00E+00 | 1.416 | 1.57E-01 |
| CRISP2 | 6 | 49660071 | 49681303 | 0.220 | rs62413604 | -2.509 | rs555247 | 0.124 | 29.920 | -1.386 | 82 | 82 | enet | 0.240 | 0.00E+00 | -1.348 | 1.78E-01 |
| NT5E | 6 | 86159302 | 86205509 | 0.131 | rs12530242 | -2.573 | rs6903114 | 0.092 | -25.820 | -1.284 | 51 | 51 | enet | 0.150 | 2.40E-259 | 1.339 | 1.81E-01 |
| VTA1 | 6 | 142468299 | 142545822 | 0.046 | rs17071756 | -2.287 | rs225628 | 0.034 | -15.870 | 0.319 | 43 | 43 | enet | 0.061 | 2.10E-101 | 1.132 | 2.58E-01 |
| PSMB1 | 6 | 170844208 | 170862395 | 0.065 | rs36058340 | -1.645 | rs3734763 | 0.132 | -31.050 | -0.753 | 26 | 26 | enet | 0.130 | 3.60E-228 | 0.958 | 3.38E-01 |
| VEGFA | 6 | 43737921 | 43754224 | 0.018 | rs9369434 | 1.567 | rs6921438 | 0.014 | -10.520 | 0.810 | 13 | 13 | enet | 0.013 | 1.10E-22 | -0.928 | 3.54E-01 |
| THBS2 | 6 | 169615875 | 169654145 | 0.234 | rs140033265 | -3.673 | rs74507247 | 0.159 | 33.890 | 0.834 | 97 | 97 | enet | 0.200 | 0.00E+00 | 0.907 | 3.64E-01 |
| NQO2 | 6 | 2988221 | 3019989 | 0.436 | rs116630977 | -2.190 | rs9378755 | 0.180 | -35.350 | -1.530 | 95 | 95 | enet | 0.410 | 0.00E+00 | 0.884 | 3.77E-01 |
| ADGRF5 | 6 | 46820259 | 46922680 | 0.150 | rs7741899 | -1.800 | rs586024 | 0.232 | 40.970 | 0.540 | 59 | 59 | enet | 0.260 | 0.00E+00 | 0.863 | 3.88E-01 |
| STX7 | 6 | 132767006 | 132834337 | 0.046 | rs73000806 | -1.418 | rs3813356 | 0.022 | -13.250 | 0.752 | 11 | 11 | enet | 0.028 | 3.40E-47 | -0.806 | 4.20E-01 |
| IGF2R | 6 | 160390114 | 160534539 | 0.347 | rs138990898 | 3.435 | rs2282140 | 0.175 | 35.590 | 0.801 | 82 | 82 | enet | 0.290 | 0.00E+00 | 0.752 | 4.52E-01 |
| ACOT13 | 6 | 24667263 | 24705293 | 0.086 | rs303019 | 1.656 | rs4544900 | 0.026 | 14.350 | -0.742 | 33 | 33 | enet | 0.054 | 1.10E-88 | -0.626 | 5.31E-01 |
| GSTA1 | 6 | 52656169 | 52668658 | 0.112 | rs72927453 | -1.823 | rs4715316 | 0.261 | 43.600 | -0.683 | 64 | 64 | enet | 0.280 | 0.00E+00 | -0.597 | 5.51E-01 |
| LRP11 | 6 | 150139893 | 150185495 | 0.242 | rs55661101 | -2.014 | rs3805753 | 0.383 | 52.630 | -0.306 | 146 | 146 | enet | 0.420 | 0.00E+00 | -0.594 | 5.53E-01 |
| PPIL1 | 6 | 36822603 | 36842579 | 0.238 | rs9394409 | 1.262 | rs10440825 | 0.103 | -27.210 | 0.573 | 17 | 17 | enet | 0.100 | 2.00E-172 | -0.571 | 5.68E-01 |
| SERPINB1 | 6 | 2832566 | 2842193 | 0.061 | rs140905415 | 1.580 | rs316343 | 0.043 | 18.140 | -0.165 | 25 | 25 | enet | 0.051 | 6.60E-85 | -0.474 | 6.35E-01 |
| IL20RA | 6 | 137321108 | 137366317 | 0.018 | rs62420816 | 6.651 | rs1775296 | 0.006 | -6.810 | 0.255 | 45 | 45 | enet | 0.010 | 2.40E-17 | 0.465 | 6.42E-01 |
| RSPO3 | 6 | 127439816 | 127520626 | 0.081 | rs74806437 | -2.147 | rs2154167 | 0.098 | 26.980 | 0.026 | 45 | 45 | enet | 0.110 | 2.80E-180 | -0.389 | 6.97E-01 |
| TREM1 | 6 | 41235664 | 41254420 | 0.180 | rs9394779 | -2.270 | rs2234243 | 0.123 | -29.820 | 0.736 | 107 | 107 | enet | 0.230 | 0.00E+00 | -0.371 | 7.11E-01 |
| PREP | 6 | 105721093 | 105901937 | 0.205 | rs2400135 | 3.379 | rs1051484 | 0.164 | 34.470 | 0.243 | 93 | 93 | enet | 0.260 | 0.00E+00 | 0.341 | 7.33E-01 |

| | | | | | | | | | | | | | | | | | |
|---|---|---|---|---|---|---|---|---|---|---|---|---|---|---|---|---|---|
| RSPO3 | 6 | 127439816 | 127520626 | 0.084 | rs111992865 | -2.855 | rs1892172 | 0.124 | 29.940 | 0.058 | 49 | 49 | enet | 0.130 | 1.60E-229 | -0.330 | 7.42E-01 |
| UST | 6 | 149068166 | 149398126 | 0.047 | rs28592230 | -2.235 | rs7764228 | 0.009 | -9.260 | 0.493 | 86 | 86 | enet | 0.033 | 2.50E-55 | -0.275 | 7.84E-01 |
| ECI2 | 6 | 4115927 | 4135831 | 0.065 | rs2208626 | 0.936 | rs7757606 | 0.084 | -24.810 | 0.315 | 36 | 36 | enet | 0.083 | 1.20E-138 | -0.272 | 7.86E-01 |
| TREM2 | 6 | 41126244 | 41130924 | 0.108 | rs56246713 | 2.479 | rs143332484 | 0.048 | -18.740 | -0.159 | 39 | 39 | enet | 0.053 | 2.30E-87 | 0.256 | 7.98E-01 |
| TXNDC5 | 6 | 7881750 | 7911021 | 0.083 | rs17398289 | 2.749 | rs111331197 | 0.034 | 15.780 | -0.677 | 28 | 28 | enet | 0.060 | 1.10E-98 | -0.250 | 8.03E-01 |
| LRP11 | 6 | 150139893 | 150185495 | 0.214 | rs6939385 | 1.863 | rs1889471 | 0.499 | 60.280 | -0.072 | 88 | 88 | enet | 0.560 | 0.00E+00 | -0.164 | 8.70E-01 |
| CLIC5 | 6 | 45848564 | 46048085 | 0.265 | rs79580941 | 3.596 | rs35822882 | 0.073 | -23.020 | -0.941 | 75 | 75 | enet | 0.088 | 2.30E-147 | -0.100 | 9.21E-01 |
| GMPR | 6 | 16238818 | 16295780 | 0.273 | rs138539729 | -2.429 | rs6459467 | 0.428 | 55.590 | 0.051 | 29 | 29 | enet | 0.430 | 0.00E+00 | -0.049 | 9.61E-01 |
| SMAP1 | 6 | 71377479 | 71571714 | 0.270 | NA | NA | NA | NA | NA | NA | 89 | 0 | enet | 0.320 | 0.00E+00 | NA | NA |
| COL9A1 | 6 | 70925743 | 71012787 | 0.024 | NA | NA | NA | NA | NA | NA | 51 | 0 | enet | 0.014 | 2.40E-24 | NA | NA |
| VNN2 | 6 | 133065009 | 133084598 | 0.398 | NA | NA | NA | NA | NA | NA | 86 | 0 | enet | 0.330 | 0.00E+00 | NA | NA |
| RNASET2 | 6 | 167335601 | 167370679 | 0.196 | NA | NA | NA | NA | NA | NA | 66 | 0 | enet | 0.160 | 2.00E-270 | NA | NA |
| ASF1A | 6 | 119215316 | 119230336 | 0.007 | NA | NA | NA | NA | NA | NA | 23 | 0 | enet | 0.004 | 5.30E-08 | NA | NA |
| SH3BGRL2 | 6 | 80341046 | 80413372 | 0.104 | NA | NA | NA | NA | NA | NA | 44 | 0 | enet | 0.100 | 4.20E-169 | NA | NA |
| CD109 | 6 | 74405508 | 74538040 | 0.345 | NA | NA | NA | NA | NA | NA | 179 | 0 | enet | 0.340 | 0.00E+00 | NA | NA |
| PLG | 6 | 161123270 | 161175129 | 0.546 | NA | NA | NA | NA | NA | NA | 166 | 0 | enet | 0.230 | 0.00E+00 | NA | NA |
| PLG | 6 | 161123270 | 161175129 | 0.425 | NA | NA | NA | NA | NA | NA | 119 | 0 | enet | 0.170 | 7.20E-293 | NA | NA |
| IL22RA2 | 6 | 137464957 | 137494785 | 0.048 | NA | NA | NA | NA | NA | NA | 46 | 0 | enet | 0.032 | 2.60E-53 | NA | NA |
| TREML2 | 6 | 41157487 | 41168887 | 0.189 | NA | NA | NA | NA | NA | NA | 64 | 0 | enet | 0.180 | 1.30E-306 | NA | NA |
| CLPS | 6 | 35762759 | 35765121 | 0.276 | NA | NA | NA | NA | NA | NA | 147 | 0 | enet | 0.150 | 1.90E-252 | NA | NA |
| MANEA | 6 | 96025361 | 96057346 | 0.333 | NA | NA | NA | NA | NA | NA | 92 | 0 | enet | 0.420 | 0.00E+00 | NA | NA |
| DLK2 | 6 | 43418090 | 43424370 | 0.112 | NA | NA | NA | NA | NA | NA | 56 | 0 | enet | 0.081 | 2.20E-134 | NA | NA |
| METTL24 | 6 | 110565143 | 110679552 | 0.052 | NA | NA | NA | NA | NA | NA | 38 | 0 | enet | 0.028 | 5.70E-47 | NA | NA |
| PTK7 | 6 | 43044045 | 43129457 | 0.059 | NA | NA | NA | NA | NA | NA | 124 | 0 | enet | 0.033 | 2.70E-54 | NA | NA |
| MYO6 | 6 | 76458909 | 76629254 | 0.036 | NA | NA | NA | NA | NA | NA | 18 | 0 | enet | 0.017 | 2.00E-28 | NA | NA |

**Table S2. PWAS identifying brain proteins associated with multiple sclerosis, through integrating pQTL and MS GWAS**

| Gene | Chr | P0 | P1 | HSQ | BEST.GWAS.ID | BEST.GWAS.Z | PQTL.ID | PQTL.R2 | PQTL.Z | PQTL.GWAS.Z | $N_{SNP}$ | $N_{WGT}$ | MODEL | MODELCV.R2 | MODELCV.PV | PWAS.Z | PWAS.P |
|---|---|---|---|---|---|---|---|---|---|---|---|---|---|---|---|---|---|
| HLA-B | 6 | 31321649 | 31324965 | 0.1950 | rs2523612 | 23.440 | rs2523589 | 0.056 | 5.770 | 16.626 | 19 | 14 | enet | 0.200 | 2.50E-18 | 2.09E+01 | 2.32E-97 |
| DDAH2 | 6 | 31694815 | 31698394 | 0.2217 | rs2227956 | 28.260 | rs1144708 | 0.223 | -9.210 | -6.250 | 40 | 9 | enet | 0.200 | 1.70E-20 | 1.37E+01 | 1.78E-42 |
| SKIV2L | 6 | 31926857 | 31937532 | 0.1027 | rs9267658 | 34.470 | rs391165 | 0.009 | -4.410 | -4.682 | 70 | 18 | enet | 0.039 | 6.20E-05 | -1.16E+01 | 2.97E-31 |
| PSMB9 | 6 | 32811913 | 32827362 | 0.1739 | rs3763354 | 29.090 | rs17587 | 0.059 | -4.980 | -7.634 | 144 | 29 | enet | 0.044 | 3.00E-05 | 9.11E+00 | 8.17E-20 |
| TSFM | 12 | 58176372 | 58201854 | 0.1111 | rs701006 | -6.760 | rs10877019 | 0.231 | -9.590 | -6.418 | 83 | 16 | enet | 0.210 | 4.30E-21 | 6.53E+00 | 6.52E-11 |
| EVI5 | 1 | 92974253 | 93257961 | 0.0388 | rs11164820 | 7.636 | rs4847221 | 0.007 | 3.310 | -1.385 | 117 | 10 | enet | 0.011 | 2.50E-02 | -5.77E+00 | 8.10E-09 |
| ZC2HC1A | 8 | 79578282 | 79632000 | 0.0544 | rs3736169 | 5.700 | rs2369440 | 0.094 | 6.100 | 5.649 | 82 | 10 | enet | 0.086 | 3.90E-09 | 5.49E+00 | 3.93E-08 |
| TRAF3 | 14 | 103243813 | 103377837 | 0.0870 | rs12148050 | 5.870 | rs3803286 | 0.071 | 5.500 | 5.545 | 102 | 15 | enet | 0.057 | 1.50E-06 | 5.11E+00 | 3.16E-07 |
| ACLY | 17 | 40023161 | 40086795 | 0.0400 | rs13412 | -4.180 | rs13412 | 0.006 | -3.520 | -4.181 | 103 | 6 | enet | 0.002 | 2.00E-01 | 4.92E+00 | 8.79E-07 |
| GALC | 14 | 88304164 | 88460009 | 0.4590 | rs17124032 | -6.860 | rs365448 | 0.436 | -13.020 | -5.392 | 188 | 29 | enet | 0.510 | 7.90E-60 | 4.65E+00 | 3.39E-06 |
| DOC2A | 16 | 30016830 | 30034591 | 0.0623 | rs11865086 | -5.884 | rs11642612 | 0.080 | 6.120 | 4.047 | 76 | 15 | enet | 0.070 | 1.20E-07 | 4.55E+00 | 5.48E-06 |
| MERTK | 2 | 112656056 | 112787138 | 0.0307 | rs6737989 | -5.496 | rs6713344 | 0.004 | 3.870 | -5.055 | 147 | 12 | enet | 0.002 | 1.80E-01 | -4.48E+00 | 7.43E-06 |
| PREX1 | 20 | 47240790 | 47444420 | 0.0981 | rs6019349 | 5.311 | rs11698214 | 0.009 | 4.420 | 2.643 | 229 | 35 | enet | 0.053 | 4.10E-06 | 4.34E+00 | 1.45E-05 |
| LMAN2 | 5 | 176758563 | 176778853 | 0.1922 | rs4976646 | 5.025 | rs2731663 | 0.008 | -4.430 | -2.113 | 76 | 16 | enet | 0.039 | 6.40E-05 | 4.32E+00 | 1.54E-05 |
| LLGL1 | 17 | 18128901 | 18148189 | 0.0440 | rs12945597 | 4.540 | rs4925159 | 0.041 | 4.150 | 4.139 | 101 | 4 | enet | 0.002 | 1.90E-01 | 4.32E+00 | 1.59E-05 |
| TMEM132A | 11 | 60691935 | 60704631 | 0.0854 | rs2074230 | 6.930 | rs11230548 | 0.010 | 3.830 | -1.458 | 120 | 15 | enet | 0.025 | 1.20E-03 | -4.23E+00 | 2.39E-05 |
| MTHFR | 1 | 11845780 | 11866977 | 0.6112 | rs1801133 | -3.641 | rs1801133 | 0.271 | -10.210 | -3.641 | 144 | 22 | enet | 0.370 | 3.20E-39 | 4.21E+00 | 2.56E-05 |
| ICA1L | 2 | 203640690 | 203736708 | 0.1511 | rs1541853 | -3.965 | rs1541853 | 0.163 | 8.210 | -3.965 | 81 | 8 | enet | 0.180 | 7.50E-18 | -4.18E+00 | 2.94E-05 |
| ABCB9 | 12 | 123405498 | 123466196 | 0.1069 | rs585522 | 5.570 | rs7305511 | 0.090 | 6.050 | 5.332 | 55 | 13 | enet | 0.074 | 5.20E-08 | 4.08E+00 | 4.50E-05 |
| PANK4 | 1 | 2439972 | 2458039 | 0.0865 | rs10797440 | -8.141 | rs10910082 | 0.037 | 6.140 | -2.629 | 96 | 13 | enet | 0.068 | 1.80E-07 | -4.00E+00 | 6.24E-05 |
| FAM120B | 6 | 170599791 | 170716153 | 0.0743 | rs9366200 | -4.054 | rs9366199 | 0.088 | -5.860 | -4.051 | 159 | 16 | enet | 0.075 | 5.70E-08 | 3.99E+00 | 6.69E-05 |
| RAB24 | 5 | 176728199 | 176730745 | 0.0627 | rs4976646 | 5.025 | rs28362590 | 0.061 | 5.360 | -4.088 | 73 | 6 | enet | 0.037 | 9.90E-05 | -3.91E+00 | 9.17E-05 |
| GIMAP4 | 7 | 150264365 | 150271041 | 0.1878 | rs6944136 | -4.080 | rs12531207 | 0.164 | 8.560 | 3.472 | 114 | 16 | enet | 0.220 | 1.90E-22 | 3.79E+00 | 1.50E-04 |
| POGLUT1 | 3 | 119187785 | 119213555 | 0.1133 | rs2293370 | -6.220 | rs9872589 | 0.071 | -5.540 | -4.755 | 143 | 15 | enet | 0.077 | 4.30E-08 | 3.71E+00 | 2.10E-04 |
| SHMT1 | 17 | 18231187 | 18266856 | 0.3867 | rs12945597 | 4.540 | rs2461838 | 0.369 | 12.100 | 4.378 | 58 | 17 | enet | 0.460 | 4.80E-52 | 3.68E+00 | 2.31E-04 |
| HLA-DRB1 | 6 | 32546546 | 32557625 | 0.3845 | rs9271055 | 37.070 | rs2858870 | -0.005 | -4.510 | -7.845 | 13 | 6 | enet | 0.210 | 5.10E-12 | 3.59E+00 | 3.28E-04 |
| WARS | 14 | 100800125 | 100843142 | 0.0829 | rs4900463 | -4.060 | rs12897338 | 0.079 | 6.710 | 2.356 | 91 | 6 | enet | 0.110 | 2.30E-11 | 3.56E+00 | 3.68E-04 |
| CAMK2G | 10 | 75572259 | 75634343 | 0.0614 | rs17741873 | -4.250 | rs2688623 | 0.017 | -3.780 | -2.694 | 78 | 10 | enet | 0.021 | 3.00E-03 | 3.49E+00 | 4.77E-04 |
| LRRC16A | 6 | 25279306 | 25620758 | 0.1452 | rs1980449 | 4.446 | rs1543603 | 0.024 | 5.820 | 1.754 | 332 | 25 | enet | 0.059 | 1.10E-06 | 3.42E+00 | 6.32E-04 |
| HMGCL | 1 | 24128375 | 24165110 | 0.0745 | rs12733278 | -3.824 | rs11591202 | 0.118 | 7.170 | 3.714 | 55 | 12 | enet | 0.099 | 2.50E-10 | 3.40E+00 | 6.85E-04 |
| SLC44A2 | 19 | 10713133 | 10755235 | 0.0238 | rs11668429 | -5.230 | rs3810154 | 0.017 | 4.000 | -3.036 | 96 | 13 | enet | 0.008 | 4.30E-02 | -3.38E+00 | 7.26E-04 |
| VKORC1 | 16 | 31102163 | 31107301 | 0.0644 | rs1978487 | 3.041 | rs2855475 | 0.040 | 4.090 | 2.854 | 51 | 14 | enet | 0.019 | 5.50E-03 | 3.30E+00 | 9.72E-04 |
| AUH | 9 | 93976097 | 94124195 | 0.1186 | rs2457715 | 3.610 | rs4743820 | 0.130 | 7.920 | -3.433 | 130 | 11 | enet | 0.170 | 1.80E-17 | -3.28E+00 | 1.02E-03 |
| PIK3IP1 | 22 | 31677579 | 31688520 | 0.0278 | rs9609263 | -4.040 | rs7287267 | 0.023 | -3.600 | -3.282 | 64 | 4 | enet | 0.000 | 2.80E-01 | 3.26E+00 | 1.10E-03 |
| PDE2A | 11 | 72287185 | 72385635 | 0.0672 | rs900613 | 3.810 | rs1864323 | 0.005 | -3.780 | 1.513 | 149 | 14 | enet | 0.009 | 3.70E-02 | -3.23E+00 | 1.22E-03 |
| CDC42BPB | 14 | 103398716 | 103523799 | 0.0648 | rs7147531 | 3.980 | rs1190234 | 0.031 | 4.970 | 3.178 | 99 | 10 | enet | 0.051 | 5.20E-06 | 3.21E+00 | 1.32E-03 |
| PARP10 | 8 | 145051321 | 145086940 | 0.0560 | rs4077669 | 4.170 | rs11989052 | 0.045 | 4.250 | 3.078 | 67 | 7 | enet | 0.058 | 6.40E-05 | 3.20E+00 | 1.37E-03 |
| ACOT7 | 1 | 6324329 | 6454451 | 0.0666 | rs14708 | 3.999 | rs3789498 | 0.122 | -6.840 | 2.985 | 117 | 5 | enet | 0.082 | 8.30E-09 | -3.19E+00 | 1.45E-03 |
| LRP4 | 11 | 46878419 | 46940193 | 0.0708 | rs11039035 | 4.360 | rs6485702 | 0.015 | 4.000 | 2.275 | 66 | 13 | enet | 0.030 | 4.50E-04 | 3.13E+00 | 1.74E-03 |
| MBLAC1 | 7 | 99724317 | 99726118 | 0.4233 | rs4134891 | 3.140 | rs2307355 | 0.421 | -12.910 | 3.056 | 72 | 10 | enet | 0.440 | 5.30E-48 | -3.12E+00 | 1.81E-03 |
| ECHDC1 | 6 | 127609855 | 127664754 | 0.0989 | rs3823043 | -2.872 | rs11556354 | 0.007 | 3.970 | -1.492 | 65 | 11 | enet | 0.050 | 7.50E-06 | -3.12E+00 | 1.82E-03 |
| CARM1 | 19 | 10982189 | 11033453 | 0.0840 | rs11878377 | -3.660 | rs12460421 | 0.065 | 5.240 | -3.135 | 70 | 5 | enet | 0.044 | 2.60E-05 | -3.11E+00 | 1.86E-03 |

| | | | | | | | | | | | | | | | | | |
|---|---|---|---|---|---|---|---|---|---|---|---|---|---|---|---|---|---|
| TYW5 | 2 | 200794698 | 200820459 | 0.0250 | rs11694369 | -4.043 | rs7569510 | 0.019 | -3.790 | -3.170 | 75 | 9 | enet | 0.005 | 1.00E-01 | 3.10E+00 | 1.96E-03 |
| RNH1 | 11 | 494512 | 507300 | 0.1812 | rs7935013 | -2.990 | rs7121599 | 0.263 | -10.140 | -2.977 | 82 | 14 | enet | 0.240 | 6.80E-24 | 3.09E+00 | 2.02E-03 |
| FYCO1 | 3 | 45959396 | 46037316 | 0.0322 | rs6441929 | 3.170 | rs13074382 | 0.032 | 3.780 | -2.946 | 130 | 7 | enet | 0.014 | 1.60E-02 | -3.06E+00 | 2.23E-03 |
| MADD | 11 | 47290712 | 47351582 | 0.3678 | rs10838692 | 4.710 | rs11570115 | 0.169 | -8.240 | -3.477 | 99 | 18 | enet | 0.200 | 1.80E-20 | 3.04E+00 | 2.39E-03 |
| FUCA1 | 1 | 24171567 | 24194784 | 0.0283 | rs12733278 | -3.824 | rs2473375 | 0.013 | -3.540 | 2.222 | 48 | 9 | enet | 0.013 | 1.70E-02 | -3.03E+00 | 2.48E-03 |
| SBF2 | 11 | 9800214 | 10315754 | 0.0652 | rs4910089 | -3.620 | rs7944706 | -0.001 | 3.440 | -3.118 | 289 | 33 | enet | 0.010 | 2.80E-02 | -3.02E+00 | 2.51E-03 |
| TREX1 | 3 | 48506445 | 48509044 | 0.0857 | rs12491849 | 3.230 | rs11797 | 0.153 | -7.380 | 2.915 | 62 | 8 | enet | 0.120 | 3.80E-11 | -2.99E+00 | 2.75E-03 |
| PANK2 | 20 | 3869486 | 3907605 | 0.1856 | rs16989000 | 2.981 | rs16989000 | 0.168 | 8.120 | 2.981 | 105 | 14 | enet | 0.190 | 3.00E-19 | 2.98E+00 | 2.87E-03 |
| RHOQ | 2 | 46768945 | 46810260 | 0.0547 | rs6713990 | 3.231 | rs1901263 | 0.004 | 3.620 | 0.062 | 139 | 10 | enet | 0.020 | 4.70E-03 | -2.97E+00 | 2.99E-03 |
| FLCN | 17 | 17115526 | 17140502 | 0.0668 | rs12600635 | 4.030 | rs16961474 | 0.047 | 4.300 | 3.165 | 92 | 5 | enet | 0.022 | 2.50E-03 | 2.95E+00 | 3.22E-03 |
| GLB1 | 3 | 33038100 | 33138722 | 0.0845 | rs11919880 | -4.270 | rs7637099 | 0.104 | -6.650 | -2.531 | 121 | 12 | enet | 0.082 | 8.70E-09 | 2.94E+00 | 3.24E-03 |
| TMA7 | 3 | 48481667 | 48485616 | 0.0215 | rs12491849 | 3.230 | rs1563736 | 0.023 | -3.680 | 2.869 | 67 | 10 | enet | 0.003 | 1.60E-01 | -2.94E+00 | 3.28E-03 |
| OSBPL11 | 3 | 125247702 | 125313934 | 0.0393 | rs2979306 | 3.180 | rs9838753 | 0.047 | -4.260 | -2.274 | 111 | 5 | enet | 0.023 | 2.40E-03 | 2.93E+00 | 3.36E-03 |
| ASAP3 | 1 | 23755056 | 23811061 | 0.1392 | rs6667575 | 2.969 | rs1555024 | 0.078 | -5.610 | -2.524 | 89 | 32 | enet | 0.070 | 1.60E-07 | 2.91E+00 | 3.58E-03 |
| CLIP2 | 7 | 73703805 | 73820273 | 0.2989 | rs10279483 | 2.270 | rs10949838 | 0.086 | -5.870 | -1.925 | 79 | 38 | enet | 0.110 | 8.90E-12 | 2.87E+00 | 4.08E-03 |
| MPI | 15 | 75182346 | 75191798 | 0.1750 | rs6495126 | 3.230 | rs1130741 | 0.229 | -10.370 | 2.087 | 79 | 15 | enet | 0.300 | 6.60E-31 | -2.87E+00 | 4.09E-03 |
| TXN | 9 | 113006091 | 113018920 | 0.3200 | rs4135168 | -2.870 | rs1049927 | 0.219 | -9.240 | -2.833 | 142 | 15 | enet | 0.240 | 2.40E-24 | 2.87E+00 | 4.14E-03 |
| CDC37L1 | 9 | 4679559 | 4708398 | 0.0822 | rs10815051 | -2.880 | rs445684 | 0.064 | -5.210 | -2.679 | 140 | 10 | enet | 0.043 | 2.80E-05 | 2.84E+00 | 4.50E-03 |
| CNPY4 | 7 | 99717236 | 99723134 | 0.1255 | rs4134891 | 3.140 | rs4134891 | 0.034 | -4.420 | 3.139 | 73 | 12 | enet | 0.070 | 1.50E-07 | -2.82E+00 | 4.84E-03 |
| IQGAP1 | 15 | 90931450 | 91045475 | 0.0576 | rs7183707 | 5.540 | rs17176602 | 0.008 | 3.590 | -0.850 | 141 | 22 | enet | 0.011 | 2.10E-02 | -2.81E+00 | 4.89E-03 |
| PTPMT1 | 11 | 47586982 | 47595013 | 0.0574 | rs4752783 | 3.290 | rs1044269 | 0.041 | 4.200 | 3.173 | 38 | 12 | enet | 0.051 | 6.50E-06 | 2.81E+00 | 4.90E-03 |
| CA4 | 17 | 58227297 | 58248260 | 0.1572 | rs345187 | 3.250 | rs345189 | 0.124 | -7.370 | 2.734 | 45 | 13 | enet | 0.130 | 3.20E-13 | -2.81E+00 | 4.99E-03 |
| KIAA1468 | 18 | 59854491 | 59974355 | 0.0645 | rs11663647 | 3.850 | rs9320002 | 0.055 | -4.900 | 2.314 | 151 | 13 | enet | 0.037 | 9.40E-05 | -2.80E+00 | 5.08E-03 |
| GNB1 | 1 | 1716729 | 1822495 | 0.0353 | rs1014988 | -3.415 | rs12044597 | 0.024 | 3.650 | 3.337 | 74 | 8 | enet | 0.018 | 5.70E-03 | 2.80E+00 | 5.09E-03 |
| LZTFL1 | 3 | 45864808 | 45957534 | 0.2540 | rs6441929 | 3.170 | rs1129183 | 0.135 | -7.370 | 3.071 | 144 | 28 | enet | 0.120 | 6.60E-12 | -2.79E+00 | 5.32E-03 |
| FAM172A | 5 | 92953775 | 93447404 | 0.0682 | rs10061080 | 2.984 | rs9314093 | 0.092 | 6.380 | 2.716 | 162 | 13 | enet | 0.100 | 1.80E-10 | 2.77E+00 | 5.54E-03 |
| XRCC6BP1 | 12 | 58335324 | 58351052 | 0.0540 | rs1599751 | -5.290 | rs10877041 | 0.050 | -4.780 | -2.570 | 72 | 5 | enet | 0.048 | 2.90E-04 | 2.77E+00 | 5.66E-03 |
| ULK3 | 15 | 75128457 | 75135687 | 0.3465 | rs6495126 | 3.230 | rs936227 | 0.437 | 12.870 | 2.310 | 87 | 11 | enet | 0.450 | 1.10E-50 | 2.75E+00 | 5.93E-03 |
| CHI3L1 | 1 | 203148059 | 203155877 | 0.0964 | rs2486934 | 3.504 | rs946263 | 0.045 | -4.270 | 1.068 | 131 | 27 | enet | 0.037 | 1.00E-03 | -2.74E+00 | 6.16E-03 |
| ORAI2 | 7 | 102073553 | 102097268 | 0.0644 | rs6976282 | 3.040 | rs7801498 | 0.026 | -4.170 | -2.826 | 41 | 9 | enet | 0.030 | 6.90E-03 | 2.72E+00 | 6.52E-03 |
| GDPGP1 | 15 | 90777040 | 90785315 | 0.6311 | rs7183707 | 5.540 | rs10152994 | 0.396 | -12.070 | 2.062 | 95 | 20 | enet | 0.450 | 3.10E-49 | -2.72E+00 | 6.55E-03 |
| TM7SF2 | 11 | 64879317 | 64883856 | 0.0625 | rs1129195 | 2.470 | rs9971407 | 0.008 | -3.970 | 2.274 | 87 | 7 | enet | -0.002 | 5.20E-01 | -2.71E+00 | 6.76E-03 |
| SCGN | 6 | 25652464 | 25702011 | 0.0726 | rs1980449 | 4.446 | rs12213891 | 0.013 | 4.110 | -3.301 | 168 | 4 | enet | 0.005 | 1.00E-01 | -2.70E+00 | 6.86E-03 |
| GTF3C3 | 2 | 197627756 | 197664449 | 0.0536 | rs16857304 | -2.954 | rs3806507 | 0.051 | -4.420 | -2.592 | 63 | 12 | enet | 0.030 | 2.00E-03 | 2.69E+00 | 7.05E-03 |
| ALCAM | 3 | 105085753 | 105295744 | 0.1930 | rs4361309 | 3.230 | rs6771987 | 0.064 | -6.190 | 2.461 | 171 | 21 | enet | 0.130 | 3.70E-13 | -2.69E+00 | 7.06E-03 |
| PPTC7 | 12 | 110969120 | 111021125 | 0.0441 | rs3864937 | 2.680 | rs4766430 | 0.045 | -4.740 | 2.517 | 50 | 6 | enet | 0.041 | 4.60E-05 | -2.69E+00 | 7.09E-03 |
| MPZL1 | 1 | 167690429 | 167761156 | 0.0742 | rs10918754 | 2.389 | rs6671664 | 0.024 | 4.220 | -2.200 | 165 | 21 | enet | 0.020 | 5.50E-03 | -2.66E+00 | 7.75E-03 |
| EIF2B5 | 3 | 183852826 | 184402546 | 0.0773 | rs4912537 | 3.110 | rs4912474 | 0.010 | -4.310 | 0.368 | 369 | 16 | enet | 0.030 | 4.20E-04 | 2.65E+00 | 7.98E-03 |
| WBP2 | 17 | 73841780 | 73852588 | 0.0619 | rs2290771 | 3.630 | rs7370 | 0.059 | -5.100 | 2.604 | 60 | 12 | enet | 0.044 | 2.50E-05 | -2.64E+00 | 8.36E-03 |
| VAT1L | 16 | 77822427 | 78014004 | 0.0738 | rs437557 | -2.644 | rs640605 | 0.018 | 4.190 | 1.631 | 350 | 15 | enet | 0.023 | 1.80E-03 | 2.62E+00 | 8.67E-03 |
| CORO1C | 12 | 109038885 | 109125372 | 0.1745 | rs11114028 | -3.410 | rs1861642 | 0.123 | -7.970 | 2.530 | 129 | 30 | enet | 0.170 | 7.80E-17 | -2.62E+00 | 8.73E-03 |
| RSU1 | 10 | 16632610 | 16859527 | 0.1235 | rs10904820 | 3.110 | rs1797076 | 0.049 | 5.280 | 3.049 | 249 | 12 | enet | 0.043 | 3.20E-05 | 2.60E+00 | 9.22E-03 |
| SLC7A6 | 16 | 68298433 | 68335722 | 0.0534 | rs4359427 | 3.018 | rs4783553 | 0.034 | -3.480 | -1.855 | 87 | 10 | enet | 0.022 | 1.70E-02 | 2.60E+00 | 9.43E-03 |
| ALDH16A1 | 19 | 49956426 | 49974305 | 0.2329 | rs4802597 | 6.430 | rs1578944 | 0.266 | 10.140 | -1.530 | 78 | 17 | enet | 0.260 | 2.30E-26 | -2.59E+00 | 9.53E-03 |
| TTC5 | 14 | 20724717 | 20774153 | 0.1313 | rs3093872 | -3.240 | rs2318864 | 0.160 | -8.060 | -2.475 | 174 | 10 | enet | 0.170 | 6.70E-17 | 2.59E+00 | 9.65E-03 |
| SSH1 | 12 | 109176466 | 109251366 | 0.0243 | rs11114028 | -3.410 | rs3741782 | 0.003 | 3.590 | 2.229 | 112 | 11 | enet | 0.002 | 2.10E-01 | 2.58E+00 | 9.88E-03 |

| | | | | | | | | | | | | | | | | | |
|---|---|---|---|---|---|---|---|---|---|---|---|---|---|---|---|---|---|
| STX1A | 7 | 73113536 | 73134002 | 0.0389 | rs4717803 | -3.400 | rs941297 | 0.005 | 3.800 | -2.879 | 74 | 17 | enet | 0.023 | 1.80E-03 | -2.58E+00 | 9.90E-03 |
| MAPRE1 | 20 | 31407699 | 31438211 | 0.0889 | rs6057638 | 3.027 | rs6119954 | 0.024 | -4.290 | 2.689 | 109 | 17 | enet | 0.022 | 2.00E-03 | -2.57E+00 | 1.00E-02 |
| IFIT5 | 10 | 91174343 | 91180758 | 0.1558 | rs304500 | 2.570 | rs304500 | 0.199 | -8.990 | 2.574 | 98 | 11 | enet | 0.200 | 1.70E-20 | -2.57E+00 | 1.01E-02 |
| SDF4 | 1 | 1152288 | 1167411 | 0.0476 | rs11721 | 3.552 | rs6697886 | 0.060 | -4.910 | 2.786 | 53 | 3 | enet | 0.054 | 9.60E-06 | -2.56E+00 | 1.03E-02 |
| COG6 | 13 | 40229764 | 40365802 | 0.0722 | rs17591266 | 4.400 | rs9548846 | 0.016 | 3.400 | 1.962 | 153 | 24 | enet | 0.018 | 4.90E-03 | 2.54E+00 | 1.10E-02 |
| NADK2 | 5 | 36192694 | 36242381 | 0.2071 | rs11741164 | 2.414 | rs10512640 | 0.002 | -3.710 | 1.903 | 79 | 39 | enet | 0.083 | 7.50E-09 | -2.54E+00 | 1.12E-02 |
| STEAP2 | 7 | 89796904 | 89867451 | 0.1021 | rs6465247 | 2.640 | rs6465243 | 0.015 | 3.730 | -0.653 | 106 | 10 | enet | 0.053 | 3.70E-06 | -2.52E+00 | 1.19E-02 |
| HINT2 | 9 | 35812957 | 35815351 | 0.0946 | rs10758328 | -3.710 | rs4879929 | 0.083 | -5.970 | -1.996 | 85 | 9 | enet | 0.068 | 1.80E-07 | 2.51E+00 | 1.21E-02 |
| EPM2A | 6 | 145822719 | 146057160 | 0.1631 | rs9399547 | 3.094 | rs12197582 | 0.022 | 4.110 | 3.004 | 120 | 14 | enet | 0.047 | 1.20E-05 | 2.51E+00 | 1.22E-02 |
| CCDC132 | 7 | 92861653 | 92988338 | 0.0407 | rs28662 | -2.970 | rs13224723 | 0.024 | 4.290 | -2.689 | 113 | 7 | enet | 0.017 | 6.30E-03 | -2.49E+00 | 1.29E-02 |
| ADA | 20 | 43248163 | 43280874 | 0.2817 | rs8121830 | -3.005 | rs2007720 | 0.192 | 8.560 | -1.532 | 91 | 18 | enet | 0.190 | 7.60E-19 | -2.48E+00 | 1.31E-02 |
| VWA7 | 6 | 31733367 | 31745108 | 0.6826 | rs9267576 | 33.890 | rs17201144 | 0.022 | -5.370 | -4.217 | 56 | 53 | enet | 0.250 | 6.80E-26 | 2.47E+00 | 1.34E-02 |
| FAIM2 | 12 | 50260679 | 50298000 | 0.0679 | rs10875982 | 3.000 | rs638515 | 0.047 | 4.710 | 1.698 | 105 | 11 | enet | 0.034 | 1.90E-04 | 2.45E+00 | 1.42E-02 |
| LRIG1 | 3 | 66429221 | 66551687 | 0.0277 | rs231276 | -2.970 | rs13434228 | 0.012 | -4.010 | 1.820 | 119 | 4 | enet | 0.002 | 2.00E-01 | -2.45E+00 | 1.43E-02 |
| MKRN1 | 7 | 140152840 | 140179369 | 0.2001 | rs11971702 | -2.380 | rs12155423 | 0.115 | -6.630 | -2.166 | 96 | 14 | enet | 0.100 | 1.20E-10 | 2.45E+00 | 1.43E-02 |
| RFT1 | 3 | 53122499 | 53164478 | 0.1446 | rs2253675 | -3.120 | rs2336725 | 0.237 | 9.010 | -2.785 | 88 | 16 | enet | 0.220 | 2.40E-20 | -2.42E+00 | 1.54E-02 |
| GBAS | 7 | 56019486 | 56067874 | 0.2559 | rs2242508 | -5.130 | rs7793921 | 0.150 | 7.650 | -3.650 | 87 | 14 | enet | 0.200 | 6.20E-20 | -2.42E+00 | 1.56E-02 |
| FOLH1 | 11 | 49168187 | 49230222 | 0.0862 | rs1164677 | 3.330 | rs10839239 | 0.097 | 6.130 | 2.860 | 59 | 12 | enet | 0.096 | 6.60E-10 | 2.41E+00 | 1.60E-02 |
| WDR48 | 3 | 39093489 | 39138155 | 0.0259 | rs1298712 | 3.730 | rs811971 | 0.012 | -3.680 | 2.460 | 97 | 9 | enet | 0.011 | 2.30E-02 | -2.40E+00 | 1.62E-02 |
| COX15 | 10 | 101471601 | 101491857 | 0.0724 | rs2002042 | 4.330 | rs11190245 | 0.064 | -5.080 | 2.755 | 81 | 6 | enet | 0.050 | 6.70E-06 | -2.40E+00 | 1.63E-02 |
| RASA2 | 3 | 141205889 | 141334184 | 0.0950 | rs6785073 | -3.560 | rs16851483 | 0.071 | 5.970 | 1.328 | 138 | 14 | enet | 0.039 | 8.00E-05 | 2.40E+00 | 1.64E-02 |
| LEMD2 | 6 | 33738979 | 33756913 | 0.0735 | rs471942 | 5.470 | rs2281918 | 0.071 | -5.780 | -1.450 | 213 | 11 | enet | 0.083 | 1.20E-08 | 2.40E+00 | 1.66E-02 |
| UFSP2 | 4 | 186320694 | 186347139 | 0.0374 | rs3733645 | 2.730 | rs6845326 | 0.014 | -3.930 | 2.620 | 99 | 9 | enet | 0.006 | 7.20E-02 | -2.39E+00 | 1.69E-02 |
| BTN2A1 | 6 | 26458150 | 26476849 | 0.6557 | rs13194984 | 4.790 | rs10456045 | 0.331 | -11.250 | 0.201 | 184 | 18 | enet | 0.560 | 8.60E-69 | -2.39E+00 | 1.69E-02 |
| GOSR2 | 17 | 45000483 | 45105003 | 0.0427 | rs1373089 | -3.490 | rs17676978 | 0.015 | -4.170 | 1.083 | 113 | 15 | enet | 0.027 | 8.00E-04 | -2.38E+00 | 1.73E-02 |
| HCLS1 | 3 | 121350246 | 121379774 | 0.0324 | rs4472078 | 4.420 | rs2070179 | 0.023 | 4.600 | 2.486 | 93 | 6 | enet | 0.028 | 7.10E-04 | 2.38E+00 | 1.74E-02 |
| DUSP19 | 2 | 183943287 | 183964733 | 0.1930 | rs11883456 | 2.902 | rs16824018 | 0.129 | -6.390 | 2.003 | 76 | 25 | enet | 0.140 | 3.50E-11 | -2.37E+00 | 1.78E-02 |
| HSD17B4 | 5 | 118788138 | 118972894 | 0.2042 | rs10478421 | -4.132 | rs2678070 | 0.230 | -9.530 | -2.119 | 151 | 23 | enet | 0.220 | 1.70E-22 | 2.36E+00 | 1.82E-02 |
| PYGB | 20 | 25228705 | 25278650 | 0.2735 | rs3787081 | -2.159 | rs11699316 | 0.082 | -5.870 | 1.506 | 113 | 12 | enet | 0.088 | 2.40E-09 | -2.36E+00 | 1.83E-02 |
| COQ9 | 16 | 57481337 | 57495187 | 0.0694 | rs223881 | 2.343 | rs223877 | 0.075 | 6.130 | 2.302 | 105 | 15 | enet | 0.089 | 2.80E-09 | 2.36E+00 | 1.84E-02 |
| SNAP47 | 1 | 227916240 | 227968927 | 0.1071 | rs6671682 | 2.417 | rs10916203 | 0.083 | 6.100 | -0.858 | 95 | 32 | enet | 0.077 | 2.50E-08 | -2.36E+00 | 1.85E-02 |
| IKBKAP | 9 | 111629797 | 111696396 | 0.1241 | rs2039923 | 3.890 | rs1759842 | 0.024 | 4.970 | -1.647 | 188 | 26 | enet | 0.074 | 4.90E-08 | -2.35E+00 | 1.86E-02 |
| ALKBH4 | 7 | 102096685 | 102105323 | 0.1029 | rs6976282 | 3.040 | rs4989124 | 0.044 | -5.430 | -1.063 | 40 | 10 | enet | 0.066 | 4.10E-07 | 2.35E+00 | 1.86E-02 |
| RRP12 | 10 | 99116115 | 99161127 | 0.0245 | rs3814553 | 4.130 | rs10882937 | 0.000 | 3.550 | 1.732 | 90 | 5 | enet | 0.001 | 2.30E-01 | 2.35E+00 | 1.87E-02 |
| KLHL3 | 5 | 136953189 | 137071779 | 0.0388 | rs17797119 | -3.038 | rs17797119 | 0.013 | -3.720 | -3.038 | 106 | 9 | enet | 0.010 | 3.80E-02 | 2.35E+00 | 1.87E-02 |
| SUMF2 | 7 | 56131695 | 56148363 | 0.0464 | rs2242508 | -5.130 | rs7793921 | 0.018 | -3.780 | -3.650 | 75 | 10 | enet | 0.023 | 2.30E-03 | 2.35E+00 | 1.88E-02 |
| VAMP1 | 12 | 6571403 | 6580153 | 0.0619 | rs11064153 | 4.200 | rs2534715 | 0.032 | -4.800 | 3.087 | 104 | 12 | enet | 0.018 | 5.30E-03 | -2.35E+00 | 1.89E-02 |
| GART | 21 | 34876238 | 34915797 | 0.4783 | rs9808753 | 3.490 | rs2834234 | 0.472 | -13.330 | -2.056 | 74 | 17 | enet | 0.490 | 3.50E-57 | 2.34E+00 | 1.90E-02 |
| TMEM179B | 11 | 62554887 | 62557877 | 0.0515 | rs3809075 | -2.020 | rs2584916 | 0.012 | -3.650 | -1.538 | 58 | 15 | enet | 0.038 | 3.30E-03 | 2.34E+00 | 1.91E-02 |
| KIAA0195 | 17 | 73437240 | 73496171 | 0.0746 | rs9900586 | 4.860 | rs8079373 | 0.028 | 3.780 | 1.959 | 103 | 15 | enet | 0.033 | 2.40E-04 | 2.33E+00 | 1.96E-02 |
| RDH11 | 14 | 68143518 | 68162531 | 0.2718 | rs12880908 | 1.780 | rs11628035 | 0.029 | -4.740 | -1.533 | 96 | 32 | enet | 0.140 | 3.50E-14 | 2.32E+00 | 2.04E-02 |
| PPAPDC2 | 9 | 4662298 | 4665256 | 0.2112 | rs10815051 | -2.880 | rs445684 | 0.077 | -6.280 | -2.679 | 137 | 24 | enet | 0.120 | 2.30E-10 | 2.31E+00 | 2.10E-02 |
| SMCR8 | 17 | 18218624 | 18226517 | 0.0289 | rs12945597 | 4.540 | rs2273027 | 0.020 | -3.760 | -2.741 | 63 | 8 | enet | 0.010 | 2.80E-02 | 2.29E+00 | 2.18E-02 |
| PTK2B | 8 | 27168999 | 27316903 | 0.0907 | rs12334538 | -3.250 | rs6557999 | 0.004 | -3.440 | 2.347 | 220 | 21 | enet | 0.016 | 7.20E-03 | -2.28E+00 | 2.27E-02 |
| RRAS2 | 11 | 14299472 | 14386052 | 0.0404 | rs1003077 | -4.570 | rs11023170 | -0.003 | -3.350 | 0.835 | 100 | 11 | enet | 0.004 | 1.20E-01 | -2.28E+00 | 2.27E-02 |
| TRAP1 | 16 | 3701640 | 3767598 | 0.2062 | rs2791 | -2.562 | rs2791 | 0.132 | -8.660 | -2.562 | 72 | 18 | enet | 0.240 | 3.90E-24 | 2.28E+00 | 2.28E-02 |

| | | | | | | | | | | | | | | | | | |
|---|---|---|---|---|---|---|---|---|---|---|---|---|---|---|---|---|---|
| CBR3 | 21 | 37507210 | 37518864 | 0.4982 | rs4817788 | -2.420 | rs879894 | 0.589 | -14.890 | 2.259 | 122 | 15 | enet | 0.620 | 5.60E-80 | -2.27E+00 | 2.29E-02 |
| TFAM | 10 | 60144782 | 60158981 | 0.0918 | rs11006132 | 2.670 | rs9971104 | 0.101 | -6.280 | 2.064 | 86 | 5 | enet | 0.066 | 2.30E-07 | -2.28E+00 | 2.29E-02 |
| DEPTOR | 8 | 120885957 | 121063152 | 0.0936 | rs11781657 | 3.290 | rs4871827 | 0.124 | 6.390 | -1.966 | 178 | 10 | enet | 0.082 | 1.10E-07 | -2.26E+00 | 2.36E-02 |
| PSMD9 | 12 | 122326637 | 122356203 | 0.1771 | rs1795963 | -2.920 | rs1169081 | 0.126 | -7.490 | -1.571 | 89 | 14 | enet | 0.160 | 9.30E-16 | 2.26E+00 | 2.38E-02 |
| EIF2B3 | 1 | 45316450 | 45452282 | 0.0331 | rs10465818 | 3.438 | rs264011 | 0.029 | 4.460 | 2.283 | 102 | 11 | enet | 0.024 | 1.60E-03 | 2.26E+00 | 2.39E-02 |
| NSUN2 | 5 | 6599352 | 6633404 | 0.0812 | rs274713 | -4.327 | rs11741091 | 0.010 | 4.050 | 1.874 | 178 | 15 | enet | 0.015 | 1.00E-02 | 2.25E+00 | 2.47E-02 |
| AGPS | 2 | 178257372 | 178408564 | 0.0421 | rs12618139 | 2.136 | rs3769996 | 0.029 | 4.370 | 2.045 | 136 | 10 | enet | 0.010 | 3.30E-02 | 2.24E+00 | 2.49E-02 |
| FAM118B | 11 | 126081309 | 126132881 | 0.0572 | rs4937122 | -3.430 | rs527051 | 0.021 | -4.360 | -1.511 | 173 | 9 | enet | 0.038 | 1.30E-04 | 2.24E+00 | 2.51E-02 |
| GPC5 | 13 | 92050929 | 93519490 | 0.1269 | rs9556084 | 2.740 | rs9523366 | 0.024 | -5.130 | 1.495 | 725 | 14 | enet | 0.066 | 2.60E-07 | -2.24E+00 | 2.51E-02 |
| SFXN4 | 10 | 120900279 | 120925179 | 0.2602 | rs10886406 | -2.500 | rs10749291 | 0.251 | -9.790 | -2.348 | 82 | 18 | enet | 0.260 | 2.60E-26 | 2.24E+00 | 2.53E-02 |
| PDXDC1 | 16 | 15068448 | 15233196 | 0.0320 | rs4985155 | -2.242 | rs34955778 | 0.002 | -3.570 | -1.340 | 31 | 7 | enet | 0.007 | 6.10E-02 | 2.23E+00 | 2.56E-02 |
| DEPDC5 | 22 | 32149944 | 32303012 | 0.0543 | rs11703079 | 3.470 | rs7286557 | 0.039 | -4.540 | -2.818 | 113 | 7 | enet | 0.038 | 8.70E-05 | 2.23E+00 | 2.56E-02 |
| NUBPL | 14 | 31959162 | 32330430 | 0.1714 | rs17098406 | -2.060 | rs12101002 | 0.026 | -5.220 | -1.479 | 202 | 20 | enet | 0.110 | 3.10E-11 | 2.22E+00 | 2.63E-02 |
| NFXL1 | 4 | 47849257 | 47916653 | 0.0994 | rs12508460 | 3.670 | rs6823698 | 0.103 | -6.510 | -2.237 | 79 | 12 | enet | 0.097 | 1.20E-09 | 2.21E+00 | 2.69E-02 |
| KCNJ9 | 1 | 160051360 | 160060353 | 0.1397 | rs16831518 | -3.158 | rs2737703 | 0.207 | -8.430 | 2.021 | 139 | 7 | enet | 0.190 | 2.40E-16 | -2.21E+00 | 2.74E-02 |
| MYO18A | 17 | 27400528 | 27507430 | 0.0808 | rs8076604 | 3.120 | rs1517623 | 0.000 | 3.300 | -0.826 | 112 | 21 | enet | 0.022 | 2.40E-03 | -2.20E+00 | 2.79E-02 |
| INPP4A | 2 | 99061317 | 99210853 | 0.0298 | rs10201079 | 2.456 | rs17031139 | 0.043 | -4.720 | -1.535 | 108 | 24 | enet | 0.045 | 1.80E-05 | 2.19E+00 | 2.82E-02 |
| FLAD1 | 1 | 154955814 | 154965587 | 0.0313 | rs4845710 | 2.245 | rs6696397 | 0.019 | 3.870 | 2.028 | 75 | 6 | enet | 0.014 | 1.40E-02 | 2.19E+00 | 2.83E-02 |
| ERAP2 | 5 | 96211643 | 96255420 | 0.3126 | rs12054720 | 2.684 | rs7716222 | 0.489 | 10.100 | -2.393 | 132 | 41 | enet | 0.490 | 2.00E-32 | -2.19E+00 | 2.83E-02 |
| SNCG | 10 | 88718375 | 88723017 | 0.1877 | rs1240386 | 2.250 | rs1240390 | 0.093 | -6.700 | 1.228 | 58 | 11 | enet | 0.140 | 2.40E-14 | -2.19E+00 | 2.86E-02 |
| ARNT2 | 15 | 80696692 | 80890278 | 0.0583 | rs1446340 | -3.050 | rs3901896 | 0.067 | 5.190 | -2.084 | 200 | 4 | enet | 0.062 | 7.60E-07 | -2.19E+00 | 2.87E-02 |
| SELENBP1 | 1 | 151336778 | 151345209 | 0.2405 | rs6684085 | -2.415 | rs17564294 | 0.160 | -8.120 | 1.486 | 58 | 6 | enet | 0.180 | 8.50E-18 | -2.19E+00 | 2.88E-02 |
| EPG5 | 18 | 43427574 | 43547240 | 0.0557 | rs894050 | -3.910 | rs1075906 | 0.030 | 4.400 | -1.690 | 127 | 6 | enet | 0.013 | 1.90E-02 | -2.19E+00 | 2.89E-02 |
| ASAP2 | 2 | 9346894 | 9545812 | 0.0982 | rs11690126 | -2.446 | rs4392227 | 0.037 | -5.430 | 1.413 | 159 | 24 | enet | 0.051 | 5.70E-06 | -2.19E+00 | 2.89E-02 |
| PDK1 | 2 | 173420101 | 173489823 | 0.0260 | rs7566884 | 2.712 | rs11888564 | 0.024 | -4.200 | 1.907 | 116 | 9 | enet | 0.026 | 9.90E-04 | -2.18E+00 | 2.93E-02 |
| FAM177A1 | 14 | 35514113 | 35582336 | 0.0443 | rs12891473 | 3.860 | rs11156867 | 0.008 | -3.410 | -2.711 | 102 | 10 | enet | 0.015 | 9.20E-03 | 2.17E+00 | 2.97E-02 |
| SLC25A12 | 2 | 172640880 | 172864766 | 0.1035 | rs11677573 | -2.989 | rs7608403 | 0.075 | 6.500 | 1.690 | 142 | 12 | enet | 0.079 | 1.80E-08 | 2.17E+00 | 2.99E-02 |
| CPNE4 | 3 | 131252399 | 132004254 | 0.1133 | rs6766639 | -3.070 | rs1320900 | 0.043 | -4.920 | -2.221 | 537 | 22 | enet | 0.041 | 4.30E-05 | 2.17E+00 | 3.04E-02 |
| MAP1LC3A | 20 | 33134658 | 33148149 | 0.0546 | rs6088520 | 2.521 | rs6088521 | 0.037 | 4.350 | 2.266 | 72 | 3 | enet | 0.057 | 1.10E-05 | 2.16E+00 | 3.09E-02 |
| NAGK | 2 | 71291474 | 71306935 | 0.1489 | rs6724815 | -2.964 | rs10202354 | 0.047 | -4.590 | -0.624 | 105 | 25 | enet | 0.045 | 1.80E-05 | 2.15E+00 | 3.14E-02 |
| RALB | 2 | 120997640 | 121052289 | 0.0483 | rs17050073 | -2.378 | rs4849849 | 0.035 | -4.420 | 2.020 | 69 | 5 | enet | 0.029 | 5.60E-04 | -2.14E+00 | 3.22E-02 |
| FAM131B | 7 | 143050493 | 143059863 | 0.1351 | rs10233736 | -2.060 | rs4236482 | 0.109 | -6.520 | -1.964 | 94 | 4 | enet | 0.110 | 5.80E-11 | 2.14E+00 | 3.24E-02 |
| PAICS | 4 | 57301907 | 57327534 | 0.1144 | rs4865115 | 2.100 | rs6554348 | 0.033 | -4.460 | 1.404 | 81 | 23 | enet | 0.066 | 2.70E-07 | -2.14E+00 | 3.25E-02 |
| C12orf73 | 12 | 104343980 | 104359486 | 0.0883 | rs2583237 | -3.360 | rs1165694 | 0.074 | -5.330 | 2.250 | 162 | 6 | enet | 0.054 | 9.70E-06 | -2.14E+00 | 3.26E-02 |
| NRP1 | 10 | 33466420 | 33625190 | 0.1164 | rs11009265 | 2.580 | rs2228638 | 0.063 | -4.970 | -2.100 | 261 | 32 | enet | 0.037 | 1.10E-04 | 2.13E+00 | 3.31E-02 |
| CCS | 11 | 66360292 | 66373490 | 0.1588 | rs10791881 | 1.890 | rs3867132 | 0.035 | -4.520 | -1.037 | 64 | 15 | enet | 0.053 | 3.80E-06 | 2.12E+00 | 3.37E-02 |
| PTRH1 | 9 | 130455257 | 130487152 | 0.0427 | rs534652 | 2.080 | rs504434 | 0.017 | 3.660 | 1.487 | 84 | 5 | enet | 0.019 | 8.50E-03 | 2.09E+00 | 3.65E-02 |
| OPALIN | 10 | 98102973 | 98119092 | 0.0560 | rs10748668 | -3.650 | rs1923702 | 0.026 | -4.200 | -2.370 | 127 | 13 | enet | 0.036 | 1.20E-04 | 2.09E+00 | 3.66E-02 |
| IGSF21 | 1 | 18434240 | 18704977 | 0.0813 | rs11801569 | 2.703 | rs2355999 | 0.036 | -4.270 | -2.032 | 347 | 3 | enet | 0.020 | 3.30E-03 | 2.09E+00 | 3.67E-02 |
| SEPTIN4 | 17 | 56597611 | 56618179 | 0.1255 | rs2680710 | -2.960 | rs11650710 | 0.016 | -3.630 | 0.466 | 63 | 16 | enet | 0.038 | 8.80E-05 | -2.09E+00 | 3.68E-02 |
| ARHGAP12 | 10 | 32094365 | 32217742 | 0.0418 | rs2808099 | 2.440 | rs12256714 | 0.040 | -4.180 | -1.819 | 122 | 10 | enet | 0.027 | 8.70E-04 | 2.08E+00 | 3.72E-02 |
| ARHGEF25 | 12 | 58003963 | 58013162 | 0.1082 | rs701006 | -6.760 | rs1806652 | 0.081 | 6.120 | 1.975 | 85 | 5 | enet | 0.087 | 7.00E-09 | 2.08E+00 | 3.72E-02 |
| CD53 | 1 | 111415775 | 111442550 | 0.0968 | rs4838884 | 2.423 | rs343810 | 0.062 | 5.010 | -1.843 | 99 | 31 | enet | 0.080 | 1.30E-05 | -2.08E+00 | 3.75E-02 |
| PIP5KL1 | 9 | 130683158 | 130693076 | 0.2424 | rs10819317 | 3.260 | rs7865476 | 0.212 | -9.130 | -1.353 | 73 | 14 | enet | 0.200 | 2.90E-20 | 2.08E+00 | 3.78E-02 |
| APPL2 | 12 | 105567074 | 105630016 | 0.0730 | rs11112453 | 3.600 | rs10861369 | -0.002 | 3.240 | 2.143 | 136 | 12 | enet | 0.018 | 5.60E-03 | 2.08E+00 | 3.78E-02 |
| HOOK1 | 1 | 60280458 | 60342050 | 0.0532 | rs11207530 | -1.749 | rs664762 | 0.013 | 3.560 | -1.219 | 115 | 14 | enet | 0.020 | 3.50E-03 | -2.07E+00 | 3.87E-02 |

| | | | | | | | | | | | | | | | | | |
|---|---|---|---|---|---|---|---|---|---|---|---|---|---|---|---|---|---|
| MCCC1 | 3 | 182733006 | 182833863 | 0.3703 | rs1547910 | 1.770 | rs4859156 | 0.290 | -10.520 | 1.388 | 112 | 34 | enet | 0.350 | 3.20E-37 | -2.07E+00 | 3.88E-02 |
| CENPV | 17 | 16245848 | 16256970 | 0.0441 | rs17779879 | 1.900 | rs3112526 | 0.048 | 5.260 | 1.603 | 73 | 9 | enet | 0.074 | 4.80E-08 | 2.07E+00 | 3.88E-02 |
| RAB40C | 16 | 639357 | 679272 | 0.0304 | rs4984902 | -2.593 | rs1139897 | 0.025 | 3.880 | -2.000 | 104 | 6 | enet | 0.010 | 4.00E-02 | -2.06E+00 | 3.91E-02 |
| LANCL1 | 2 | 211295973 | 211342376 | 0.0393 | rs17770211 | 3.061 | rs2287420 | 0.044 | 4.450 | 2.167 | 91 | 13 | enet | 0.038 | 7.70E-05 | 2.06E+00 | 3.94E-02 |
| TRIM65 | 17 | 73876416 | 73893084 | 0.1934 | rs2290771 | 3.630 | rs8066711 | 0.180 | 6.930 | 2.839 | 61 | 10 | enet | 0.210 | 1.30E-14 | 2.05E+00 | 4.03E-02 |
| GALT | 9 | 34638130 | 34651032 | 0.1037 | rs10758267 | -3.420 | rs7037232 | 0.036 | -4.920 | -1.558 | 90 | 4 | enet | 0.040 | 6.90E-05 | 2.05E+00 | 4.05E-02 |
| TAPT1 | 4 | 16162128 | 16229033 | 0.0387 | rs10028796 | -3.070 | rs16893162 | -0.001 | 3.540 | 1.345 | 143 | 7 | enet | -0.002 | 7.20E-01 | 2.04E+00 | 4.11E-02 |
| CNTN2 | 1 | 205012325 | 205047627 | 0.0735 | rs12067709 | -3.083 | rs4951162 | 0.057 | -5.150 | -2.187 | 159 | 7 | enet | 0.048 | 1.10E-05 | 2.04E+00 | 4.15E-02 |
| PPM1F | 22 | 22273793 | 22307209 | 0.0526 | rs5755694 | -6.880 | rs240054 | 0.034 | -4.830 | 2.732 | 115 | 15 | enet | 0.036 | 1.30E-04 | -2.04E+00 | 4.18E-02 |
| DLD | 7 | 107531415 | 107572175 | 0.0471 | rs2237686 | -3.020 | rs6976266 | 0.056 | 5.260 | -2.151 | 157 | 10 | enet | 0.039 | 6.40E-05 | -2.03E+00 | 4.19E-02 |
| CYB5R1 | 1 | 202930997 | 202936408 | 0.0802 | rs2232852 | 2.233 | rs2363768 | 0.037 | -4.860 | 0.792 | 103 | 12 | enet | 0.052 | 4.40E-06 | -2.03E+00 | 4.19E-02 |
| TPD52L2 | 20 | 62496596 | 62522898 | 0.0492 | rs6062314 | -4.702 | rs7000 | 0.023 | -4.380 | -0.435 | 79 | 17 | enet | 0.028 | 6.70E-04 | 2.03E+00 | 4.24E-02 |
| ENPP4 | 6 | 46097730 | 46114436 | 0.0757 | rs4714902 | 2.921 | rs7754915 | 0.067 | 6.200 | 2.375 | 109 | 13 | enet | 0.065 | 6.60E-07 | 2.03E+00 | 4.26E-02 |
| SLC4A8 | 12 | 51785101 | 51902980 | 0.0365 | rs12369067 | 3.470 | rs12366428 | 0.031 | 4.220 | -2.036 | 138 | 7 | enet | 0.029 | 5.10E-04 | -2.03E+00 | 4.28E-02 |
| NME7 | 1 | 169101769 | 169337205 | 0.0567 | rs3007364 | 3.222 | rs2157597 | 0.075 | -5.960 | -1.729 | 201 | 20 | enet | 0.066 | 4.30E-07 | 2.02E+00 | 4.31E-02 |
| NT5C | 17 | 73126320 | 73127890 | 0.1356 | rs9909306 | 3.120 | rs729405 | 0.003 | 3.990 | 0.455 | 66 | 17 | enet | 0.029 | 5.60E-04 | 2.02E+00 | 4.33E-02 |
| TATDN3 | 1 | 212965170 | 212989968 | 0.2057 | rs1156058 | 3.953 | rs12753524 | 0.041 | 5.900 | 2.283 | 94 | 15 | enet | 0.130 | 2.20E-13 | 2.02E+00 | 4.34E-02 |
| SLC25A32 | 8 | 104410863 | 104427417 | 0.1474 | rs17802089 | 2.100 | rs17803441 | 0.078 | -5.420 | 1.781 | 96 | 12 | enet | 0.082 | 3.50E-08 | -2.02E+00 | 4.36E-02 |
| DGKA | 12 | 56321103 | 56347811 | 0.0665 | rs1056784 | -2.100 | rs1052206 | 0.013 | -4.600 | -1.764 | 45 | 12 | enet | 0.056 | 1.90E-06 | 2.01E+00 | 4.39E-02 |
| TMTC4 | 13 | 101256181 | 101327347 | 0.1089 | rs17475612 | 2.190 | rs1283210 | 0.070 | 4.680 | -1.584 | 124 | 22 | enet | 0.056 | 9.60E-05 | -2.02E+00 | 4.39E-02 |
| TMEM150C | 4 | 83404323 | 83483510 | 0.1074 | rs10014422 | 1.870 | rs3796472 | -0.005 | -3.310 | 0.953 | 133 | 15 | enet | -0.002 | 4.40E-01 | -2.01E+00 | 4.42E-02 |
| ANXA6 | 5 | 150480273 | 150537443 | 0.1385 | rs1004595 | 2.937 | rs6859236 | 0.071 | -6.460 | 2.409 | 212 | 16 | enet | 0.086 | 4.20E-09 | -2.00E+00 | 4.51E-02 |
| DDAH1 | 1 | 85784164 | 86043933 | 0.0962 | rs9887812 | 6.337 | rs648310 | 0.046 | -5.560 | 3.200 | 255 | 22 | enet | 0.092 | 1.10E-09 | -2.00E+00 | 4.58E-02 |
| APIP | 11 | 34874641 | 34938046 | 0.4192 | rs7124428 | 2.750 | rs1571134 | 0.461 | 13.080 | 1.620 | 163 | 35 | enet | 0.590 | 1.40E-72 | 1.99E+00 | 4.66E-02 |
| AAK1 | 2 | 69688532 | 69901481 | 0.0555 | rs5017686 | -2.716 | rs2312207 | 0.043 | 4.490 | 1.885 | 130 | 6 | enet | 0.032 | 2.90E-04 | 1.99E+00 | 4.70E-02 |
| MUT | 6 | 49398073 | 49430904 | 0.1072 | rs9463475 | -1.946 | rs2501976 | 0.102 | -7.020 | -1.803 | 84 | 9 | enet | 0.140 | 1.70E-13 | 1.98E+00 | 4.76E-02 |
| PLCD3 | 17 | 43186335 | 43210721 | 0.0455 | rs8077692 | 3.690 | rs12944434 | 0.007 | 3.990 | 1.551 | 96 | 10 | enet | 0.032 | 3.00E-04 | 1.97E+00 | 4.83E-02 |
| PMPCB | 7 | 102937869 | 102969958 | 0.0636 | rs17136728 | -1.420 | rs17680523 | 0.043 | -4.610 | -0.967 | 35 | 23 | enet | 0.020 | 3.50E-03 | 1.97E+00 | 4.89E-02 |
| NFU1 | 2 | 69622882 | 69664760 | 0.0740 | rs5017686 | -2.716 | rs6724567 | 0.033 | 5.270 | -2.521 | 92 | 10 | enet | 0.058 | 1.30E-06 | -1.97E+00 | 4.91E-02 |
| QPRT | 16 | 29674600 | 29710020 | 0.4857 | rs4563056 | 3.577 | rs9933310 | 0.362 | -11.820 | -3.217 | 29 | 8 | enet | 0.460 | 9.00E-52 | 1.97E+00 | 4.91E-02 |
| GLRA3 | 4 | 175558065 | 175750465 | 0.0912 | rs1426935 | 3.120 | rs13116721 | 0.002 | 3.720 | 0.973 | 202 | 32 | enet | 0.026 | 2.70E-03 | 1.97E+00 | 4.93E-02 |
| CYB561 | 17 | 61509665 | 61523739 | 0.1052 | rs3809721 | 2.140 | rs3809721 | 0.113 | -6.870 | 2.143 | 68 | 7 | enet | 0.110 | 8.20E-10 | -1.96E+00 | 4.95E-02 |
| ABHD6 | 3 | 58223233 | 58281420 | 0.1083 | rs7622074 | -2.350 | rs6802482 | 0.016 | 4.400 | 1.340 | 129 | 26 | enet | 0.075 | 4.10E-08 | 1.96E+00 | 5.06E-02 |
| THRAP3 | 1 | 36690017 | 36770958 | 0.0536 | rs6690378 | 2.743 | rs6690378 | 0.055 | 4.930 | 2.743 | 71 | 12 | enet | 0.043 | 2.90E-05 | 1.95E+00 | 5.07E-02 |
| RCN1 | 11 | 31833939 | 32127301 | 0.0776 | rs10734409 | 2.350 | rs224633 | 0.093 | -6.300 | -1.757 | 216 | 8 | enet | 0.081 | 1.10E-08 | 1.95E+00 | 5.07E-02 |
| SPRED1 | 15 | 38544527 | 38649450 | 0.0481 | rs12440278 | 2.130 | rs4924250 | 0.032 | 4.160 | -1.712 | 143 | 13 | enet | 0.031 | 4.40E-04 | -1.94E+00 | 5.23E-02 |
| ANGEL1 | 14 | 77253588 | 77292589 | 0.0695 | rs12886982 | 2.840 | rs2075772 | 0.057 | 4.870 | -2.052 | 118 | 3 | enet | 0.038 | 2.00E-04 | -1.93E+00 | 5.34E-02 |
| EIF2AK4 | 15 | 40226347 | 40327797 | 0.1007 | rs8031319 | -3.330 | rs8041057 | 0.089 | -6.070 | -1.725 | 145 | 14 | enet | 0.100 | 2.90E-10 | 1.93E+00 | 5.35E-02 |
| STAT6 | 12 | 57489191 | 57525922 | 0.1373 | rs12298170 | -2.720 | rs12322902 | 0.086 | 6.430 | -2.718 | 86 | 18 | enet | 0.097 | 3.70E-10 | -1.93E+00 | 5.36E-02 |
| HDDC2 | 6 | 125541108 | 125623282 | 0.5247 | rs2243389 | -2.206 | rs987168 | 0.534 | -14.190 | -2.190 | 136 | 34 | enet | 0.560 | 4.00E-69 | 1.93E+00 | 5.37E-02 |
| IQCB1 | 3 | 121488610 | 121553926 | 0.0801 | rs2331964 | -5.860 | rs6767409 | 0.040 | 4.820 | 3.117 | 105 | 3 | enet | 0.030 | 3.30E-03 | 1.92E+00 | 5.54E-02 |
| SLC9A3R2 | 16 | 2075357 | 2089027 | 0.0940 | rs12929367 | -2.203 | rs34123233 | 0.041 | -4.730 | -1.660 | 65 | 14 | enet | 0.046 | 1.60E-05 | 1.91E+00 | 5.58E-02 |
| LRPPRC | 2 | 44113647 | 44223144 | 0.0713 | rs4148210 | -2.460 | rs13384238 | 0.040 | 5.090 | -1.606 | 177 | 20 | enet | 0.057 | 1.60E-06 | -1.91E+00 | 5.59E-02 |
| ACTN2 | 1 | 236849754 | 236927931 | 0.0478 | rs4659713 | 2.450 | rs1652226 | 0.011 | -4.060 | 2.122 | 164 | 6 | enet | 0.018 | 4.80E-03 | -1.91E+00 | 5.60E-02 |
| NNT | 5 | 43602794 | 43707507 | 0.0650 | rs6896527 | -1.831 | rs6896527 | 0.029 | 3.740 | -1.831 | 71 | 4 | enet | 0.015 | 1.10E-02 | -1.91E+00 | 5.60E-02 |
| CTPS1 | 1 | 41445007 | 41478235 | 0.0758 | rs2300650 | 2.106 | rs4453027 | 0.064 | -5.510 | -1.717 | 102 | 16 | enet | 0.048 | 1.00E-05 | 1.90E+00 | 5.69E-02 |

| | | | | | | | | | | | | | | | | | | |
|---|---|---|---|---|---|---|---|---|---|---|---|---|---|---|---|---|---|---|
| APOL2 | 22 | 36622256 | 36636000 | 0.3779 | rs132649 | 3.880 | rs7289037 | 0.132 | -7.340 | -2.792 | 180 | 35 | enet | 0.190 | 1.40E-19 | 1.90E+00 | 5.71E-02 |
| PCYT1A | 3 | 195941093 | 196014828 | 0.0865 | rs4916519 | 3.530 | rs12637288 | 0.043 | -5.340 | 1.407 | 108 | 7 | enet | 0.046 | 1.50E-05 | -1.90E+00 | 5.72E-02 |
| PSMB4 | 1 | 151372010 | 151374420 | 0.3156 | rs6684085 | -2.415 | rs4603 | 0.569 | -14.610 | -1.928 | 52 | 14 | enet | 0.560 | 6.90E-70 | 1.90E+00 | 5.76E-02 |
| IDUA | 4 | 980785 | 998316 | 0.2860 | rs3822020 | 3.680 | rs4647939 | 0.115 | 6.290 | 0.040 | 110 | 27 | enet | 0.190 | 5.00E-13 | -1.90E+00 | 5.76E-02 |
| ADHFE1 | 8 | 67342420 | 67383836 | 0.5363 | rs957655 | 2.800 | rs1030420 | 0.412 | 12.460 | 1.842 | 73 | 15 | enet | 0.540 | 3.20E-65 | 1.90E+00 | 5.81E-02 |
| TPD52 | 8 | 80870571 | 81143467 | 0.1139 | rs7014791 | 2.390 | rs9643750 | 0.050 | 5.240 | 1.673 | 152 | 35 | enet | 0.057 | 1.70E-06 | 1.89E+00 | 5.86E-02 |
| BLVRB | 19 | 40953696 | 40971747 | 0.1385 | rs1061197 | -2.450 | rs268672 | -0.001 | -3.680 | 0.435 | 84 | 16 | enet | 0.062 | 5.50E-07 | -1.89E+00 | 5.87E-02 |
| LRRC57 | 15 | 42834720 | 42841000 | 0.0679 | rs12911585 | -2.470 | rs34359922 | 0.052 | 4.670 | -1.732 | 44 | 5 | enet | 0.047 | 1.40E-05 | -1.89E+00 | 5.90E-02 |
| CYB5R2 | 11 | 7686331 | 7698453 | 0.0801 | rs1600970 | 2.470 | rs1073484 | 0.004 | -3.300 | 1.816 | 162 | 31 | enet | -0.002 | 7.10E-01 | -1.88E+00 | 5.96E-02 |
| GSTM3 | 1 | 110276554 | 110284384 | 0.6359 | rs11102002 | -2.673 | rs11807 | 0.540 | 14.400 | -0.973 | 93 | 26 | enet | 0.670 | 2.60E-91 | -1.88E+00 | 6.02E-02 |
| NAT6 | 3 | 50333833 | 50336852 | 0.0969 | rs9852677 | -2.490 | rs13100173 | 0.100 | 6.090 | 1.996 | 54 | 2 | enet | 0.090 | 7.10E-09 | 1.88E+00 | 6.02E-02 |
| ARMC10 | 7 | 102715328 | 102740205 | 0.1988 | rs17605251 | 2.380 | rs4729873 | 0.145 | -8.200 | -1.940 | 32 | 6 | enet | 0.180 | 4.20E-18 | 1.87E+00 | 6.10E-02 |
| SMUG1 | 12 | 54558529 | 54582778 | 0.0864 | rs4759345 | 2.570 | rs7311216 | 0.076 | 5.030 | 1.600 | 71 | 7 | enet | 0.066 | 6.80E-05 | 1.87E+00 | 6.14E-02 |
| SAT2 | 17 | 7529552 | 7531194 | 0.3647 | rs9901675 | 2.970 | rs9913778 | 0.334 | -11.320 | 1.597 | 102 | 18 | enet | 0.380 | 2.90E-41 | -1.87E+00 | 6.14E-02 |
| B3GAT3 | 11 | 62382768 | 62389647 | 0.0833 | rs35587183 | -2.140 | rs2516633 | 0.056 | -5.390 | -1.907 | 51 | 10 | enet | 0.052 | 5.00E-06 | 1.87E+00 | 6.16E-02 |
| TBCC | 6 | 42712219 | 42714558 | 0.3328 | rs2234026 | -2.099 | rs2234026 | 0.151 | -7.650 | -2.099 | 72 | 3 | enet | 0.140 | 1.00E-13 | 1.85E+00 | 6.38E-02 |
| PDE4C | 19 | 18318771 | 18366229 | 0.1002 | rs874628 | -7.220 | rs172043 | 0.086 | 5.330 | 1.292 | 133 | 7 | enet | 0.055 | 2.10E-04 | 1.85E+00 | 6.40E-02 |
| ABHD12 | 20 | 25275379 | 25371619 | 0.3957 | rs3787081 | -2.159 | rs746748 | 0.144 | -7.500 | -1.975 | 140 | 14 | enet | 0.140 | 2.00E-14 | 1.85E+00 | 6.49E-02 |
| AKR1C3 | 10 | 5077546 | 5149878 | 0.1172 | rs11252861 | -2.360 | rs4143631 | 0.052 | 4.750 | -1.731 | 155 | 23 | enet | 0.021 | 5.70E-03 | -1.85E+00 | 6.49E-02 |
| TLDC1 | 16 | 84511681 | 84587639 | 0.1844 | rs394035 | 3.764 | rs9630644 | 0.132 | 7.960 | -2.278 | 291 | 18 | enet | 0.190 | 1.30E-19 | -1.84E+00 | 6.52E-02 |
| ACAD9 | 3 | 128598439 | 128634910 | 0.1435 | rs9853945 | -2.050 | rs7637352 | 0.005 | -4.330 | 1.080 | 77 | 9 | enet | 0.050 | 6.60E-06 | -1.84E+00 | 6.53E-02 |
| RHPN2 | 19 | 33469499 | 33555794 | 0.0544 | rs2304103 | -2.610 | rs1981818 | 0.062 | -5.020 | -1.838 | 118 | 7 | enet | 0.039 | 9.40E-04 | 1.84E+00 | 6.55E-02 |
| TYK2 | 19 | 10461209 | 10491352 | 0.4287 | rs11666263 | -5.700 | rs12720356 | 0.184 | -9.110 | 1.246 | 80 | 8 | enet | 0.260 | 4.90E-25 | 1.84E+00 | 6.55E-02 |
| ANKRD36C | 2 | 96514587 | 96657541 | 0.0691 | rs2579552 | 3.234 | rs11689832 | 0.043 | 3.610 | 2.954 | 22 | 12 | enet | 0.021 | 2.30E-02 | 1.84E+00 | 6.57E-02 |
| ESYT2 | 7 | 158523686 | 158622944 | 0.1311 | rs12670677 | 1.840 | rs1061733 | 0.045 | -5.040 | 0.907 | 144 | 29 | enet | 0.087 | 3.30E-09 | -1.84E+00 | 6.59E-02 |
| LUZP1 | 1 | 23410516 | 23504301 | 0.0879 | rs10917347 | 2.677 | rs683893 | 0.025 | 4.600 | -1.158 | 97 | 13 | enet | 0.068 | 1.50E-07 | -1.84E+00 | 6.62E-02 |
| CARKD | 13 | 111267881 | 111292340 | 0.4268 | rs11840245 | -2.040 | rs179356 | 0.385 | 12.540 | 1.632 | 140 | 23 | enet | 0.410 | 3.30E-45 | 1.84E+00 | 6.65E-02 |
| SOD2 | 6 | 160090089 | 160183561 | 0.1537 | rs11965350 | 2.148 | rs9365085 | 0.160 | 8.030 | -1.635 | 130 | 8 | enet | 0.160 | 2.90E-16 | -1.83E+00 | 6.76E-02 |
| PSAT1 | 9 | 80912059 | 80945009 | 0.2401 | rs10429454 | 2.270 | rs1929402 | 0.160 | -8.270 | -1.809 | 99 | 17 | enet | 0.210 | 4.40E-21 | 1.82E+00 | 6.89E-02 |
| RPE | 2 | 210867289 | 210886300 | 0.0840 | rs12470687 | -2.334 | rs7570090 | 0.082 | -6.250 | -1.428 | 46 | 9 | enet | 0.110 | 4.70E-11 | 1.82E+00 | 6.89E-02 |
| ACOT8 | 20 | 44470360 | 44486045 | 0.1861 | rs6065904 | -3.558 | rs7270170 | 0.098 | 6.860 | 2.446 | 103 | 26 | enet | 0.140 | 2.50E-14 | 1.82E+00 | 6.89E-02 |
| VWA1 | 1 | 1370241 | 1378262 | 0.0483 | rs12089560 | 2.351 | rs1695824 | 0.048 | -4.430 | 2.042 | 33 | 4 | enet | 0.018 | 5.40E-03 | -1.81E+00 | 7.08E-02 |
| GPR56 | 16 | 57644564 | 57698944 | 0.0800 | rs935746 | 2.217 | rs1466134 | -0.003 | -3.420 | -0.501 | 168 | 19 | enet | 0.005 | 9.40E-02 | 1.80E+00 | 7.12E-02 |
| ICA1 | 7 | 8152814 | 8302317 | 0.0576 | rs17142727 | -3.690 | rs12532271 | -0.003 | 3.590 | 2.152 | 233 | 4 | enet | 0.000 | 3.20E-01 | 1.80E+00 | 7.13E-02 |
| ENO1 | 1 | 8921061 | 8939308 | 0.0918 | rs6682376 | -3.057 | rs11582339 | 0.007 | -4.150 | 0.887 | 98 | 11 | enet | 0.059 | 9.80E-07 | -1.80E+00 | 7.13E-02 |
| CPS1 | 2 | 211342406 | 211543831 | 0.0683 | rs7583500 | -3.732 | rs759688 | 0.029 | -4.410 | -0.839 | 176 | 19 | enet | 0.056 | 2.30E-06 | 1.80E+00 | 7.21E-02 |
| LSP1 | 11 | 1874200 | 1913497 | 0.2142 | rs10839670 | -2.480 | rs907611 | 0.140 | 6.730 | 2.242 | 94 | 7 | enet | 0.130 | 1.10E-10 | 1.79E+00 | 7.27E-02 |
| COG7 | 16 | 23399814 | 23464501 | 0.0404 | rs12446463 | -2.281 | rs181835 | 0.043 | 5.370 | 1.717 | 103 | 10 | enet | 0.045 | 1.90E-05 | 1.79E+00 | 7.27E-02 |
| SDHA | 5 | 218356 | 256815 | 0.1072 | rs6883247 | 2.129 | rs9312983 | 0.096 | -6.140 | 1.895 | 112 | 17 | enet | 0.090 | 1.80E-09 | -1.79E+00 | 7.34E-02 |
| PHYHD1 | 9 | 131683174 | 131704320 | 0.2729 | rs3750340 | 2.560 | rs10113912 | 0.434 | -13.200 | -1.861 | 63 | 21 | enet | 0.450 | 2.90E-50 | 1.79E+00 | 7.35E-02 |
| RABGGTB | 1 | 76251879 | 76260764 | 0.0279 | rs3818855 | -2.049 | rs2275378 | 0.024 | -4.050 | -2.038 | 90 | 9 | enet | 0.005 | 8.80E-02 | 1.79E+00 | 7.42E-02 |
| HARS2 | 5 | 140071011 | 140078889 | 0.0273 | rs34282994 | -2.934 | rs778596 | 0.000 | 3.360 | -1.958 | 77 | 14 | enet | 0.001 | 2.40E-01 | -1.78E+00 | 7.45E-02 |
| FAS | 10 | 90750414 | 90775542 | 0.5130 | rs1468063 | -2.180 | rs7097572 | 0.619 | 15.010 | -1.529 | 144 | 17 | enet | 0.630 | 2.30E-80 | -1.78E+00 | 7.51E-02 |
| NT5C1A | 1 | 40124793 | 40137710 | 0.2010 | rs2242500 | 2.169 | rs9662128 | 0.165 | 7.810 | -1.104 | 91 | 22 | enet | 0.170 | 1.80E-16 | -1.78E+00 | 7.53E-02 |
| SELO | 22 | 50639408 | 50656045 | 0.1107 | rs11703226 | 3.740 | rs738338 | 0.008 | -4.090 | 0.094 | 95 | 15 | enet | 0.055 | 2.90E-06 | -1.78E+00 | 7.56E-02 |
| HBS1L | 6 | 135281516 | 135424194 | 0.1464 | rs9494167 | 5.797 | rs13214669 | 0.179 | 8.240 | -1.372 | 130 | 11 | enet | 0.170 | 4.10E-17 | -1.78E+00 | 7.57E-02 |

| | | | | | | | | | | | | | | | | | |
|---|---|---|---|---|---|---|---|---|---|---|---|---|---|---|---|---|---|
| ADPRHL1 | 13 | 114076260 | 114107839 | 0.3214 | rs9549757 | -2.330 | rs8001629 | 0.055 | -6.030 | -2.185 | 125 | 22 | enet | 0.190 | 3.70E-19 | 1.78E+00 | 7.57E-02 |
| LYZ | 12 | 69742121 | 69748014 | 0.0344 | rs11177594 | 3.000 | rs2168029 | 0.030 | 4.150 | 2.003 | 109 | 6 | enet | 0.009 | 4.40E-02 | 1.77E+00 | 7.59E-02 |
| STMN4 | 8 | 27092840 | 27115937 | 0.0780 | rs1345134 | -2.820 | rs11786687 | 0.059 | 5.590 | 0.790 | 93 | 8 | enet | 0.060 | 1.20E-06 | 1.77E+00 | 7.67E-02 |
| CSTB | 21 | 45192393 | 45196326 | 0.0485 | rs9941787 | 3.010 | rs6518323 | 0.003 | -4.200 | -1.625 | 99 | 13 | enet | 0.023 | 1.70E-03 | 1.77E+00 | 7.67E-02 |
| RPS9 | 19 | 54704610 | 54752862 | 0.1035 | rs3865475 | -2.430 | rs1205315 | -0.002 | 3.380 | 1.439 | 112 | 22 | enet | 0.008 | 4.70E-02 | 1.77E+00 | 7.68E-02 |
| BLMH | 17 | 28575218 | 28619074 | 0.0628 | rs12947084 | -2.810 | rs3110095 | 0.001 | 3.380 | -1.130 | 93 | 16 | enet | 0.037 | 9.50E-05 | 1.76E+00 | 7.90E-02 |
| C14orf159 | 14 | 91526677 | 91691976 | 0.7512 | rs1286086 | -3.230 | rs4900072 | 0.733 | -16.670 | -1.414 | 122 | 18 | enet | 0.740 | 8.90E-111 | 1.76E+00 | 7.91E-02 |
| PLA2G4C | 19 | 48551100 | 48614074 | 0.3645 | rs12611226 | -2.700 | rs16981247 | 0.161 | 6.660 | 1.248 | 171 | 18 | enet | 0.260 | 3.10E-17 | 1.75E+00 | 8.05E-02 |
| ALG11 | 13 | 52586534 | 52603800 | 0.0734 | rs9526816 | -3.040 | rs9535825 | 0.070 | -5.400 | -1.906 | 100 | 3 | enet | 0.031 | 3.60E-04 | 1.75E+00 | 8.08E-02 |
| STXBP6 | 14 | 25278862 | 25519503 | 0.4347 | rs17185348 | 2.360 | rs4983037 | 0.095 | 7.750 | -1.398 | 285 | 34 | enet | 0.410 | 1.20E-44 | -1.75E+00 | 8.09E-02 |
| ATG2B | 14 | 96747595 | 96830207 | 0.0711 | rs2275554 | 2.600 | rs4905480 | 0.055 | 5.160 | -1.502 | 162 | 14 | enet | 0.042 | 3.60E-05 | -1.75E+00 | 8.10E-02 |
| EPHB2 | 1 | 23037332 | 23241818 | 0.0680 | rs10917317 | 2.875 | rs12723359 | 0.007 | -4.730 | 0.115 | 172 | 17 | enet | 0.015 | 1.10E-02 | -1.74E+00 | 8.13E-02 |
| NDUFS1 | 2 | 206979541 | 207024327 | 0.0285 | rs2010572 | 3.335 | rs1024809 | 0.000 | 3.710 | 0.615 | 91 | 7 | enet | 0.010 | 2.80E-02 | 1.74E+00 | 8.14E-02 |
| PARL | 3 | 183547173 | 183602721 | 0.1740 | rs12634358 | 2.610 | rs3811724 | 0.078 | -6.010 | -0.795 | 143 | 21 | enet | 0.100 | 2.20E-10 | 1.74E+00 | 8.26E-02 |
| ARHGAP25 | 2 | 68906733 | 69053965 | 0.4526 | rs10193109 | -3.007 | rs2280244 | 0.356 | -11.170 | -1.711 | 231 | 20 | enet | 0.420 | 6.80E-43 | 1.73E+00 | 8.36E-02 |
| PRKG1 | 10 | 52750945 | 54058110 | 0.2694 | rs11594206 | -3.220 | rs1871631 | 0.172 | 8.680 | 1.440 | 816 | 28 | enet | 0.200 | 1.50E-20 | 1.72E+00 | 8.47E-02 |
| QPCT | 2 | 37571717 | 37600465 | 0.1262 | rs6734118 | -1.894 | rs10179447 | 0.105 | 6.230 | 1.758 | 121 | 12 | enet | 0.088 | 2.60E-08 | 1.72E+00 | 8.48E-02 |
| P4HB | 17 | 79801035 | 79818570 | 0.0233 | rs1130674 | -1.810 | rs35170948 | 0.018 | -3.320 | -1.793 | 14 | 10 | enet | 0.016 | 7.70E-03 | 1.72E+00 | 8.63E-02 |
| LIG3 | 17 | 33307513 | 33332083 | 0.0754 | rs17546473 | 2.540 | rs3744357 | 0.028 | 4.500 | 1.734 | 53 | 12 | enet | 0.037 | 1.40E-04 | 1.70E+00 | 8.84E-02 |
| LHPP | 10 | 126150403 | 126306457 | 0.2560 | rs4246200 | 2.440 | rs4962386 | 0.131 | 7.370 | 0.718 | 219 | 40 | enet | 0.160 | 1.90E-16 | 1.70E+00 | 8.91E-02 |
| BID | 22 | 18216906 | 18257536 | 0.0488 | rs5747338 | 3.320 | rs181399 | 0.003 | -3.850 | 0.362 | 132 | 11 | enet | 0.002 | 2.20E-01 | 1.70E+00 | 8.94E-02 |
| ATL3 | 11 | 63391559 | 63439393 | 0.0618 | rs7928514 | 2.070 | rs17158453 | 0.069 | -5.400 | 1.436 | 51 | 12 | enet | 0.071 | 8.50E-08 | -1.70E+00 | 8.96E-02 |
| ERAP1 | 5 | 96096521 | 96143803 | 0.5707 | rs26498 | 2.200 | rs17482078 | 0.366 | -12.170 | -0.411 | 184 | 26 | enet | 0.630 | 2.60E-83 | 1.70E+00 | 8.97E-02 |
| NUB1 | 7 | 151038785 | 151075535 | 0.0394 | rs11761588 | 2.500 | rs394259 | 0.017 | 4.300 | 2.106 | 140 | 6 | enet | 0.023 | 1.90E-03 | 1.69E+00 | 9.04E-02 |
| PPAT | 4 | 57259528 | 57301781 | 0.1417 | rs6854916 | 2.490 | rs6836118 | 0.150 | 7.810 | -1.147 | 103 | 17 | enet | 0.130 | 1.70E-13 | -1.69E+00 | 9.12E-02 |
| MCAT | 22 | 43528212 | 43539400 | 0.0550 | rs998409 | 2.410 | rs12484656 | 0.030 | -4.270 | 1.900 | 141 | 12 | enet | 0.017 | 6.10E-03 | -1.69E+00 | 9.16E-02 |
| AVEN | 15 | 34158428 | 34331377 | 0.0653 | rs10519874 | -3.050 | rs613479 | -0.001 | -3.330 | 1.193 | 196 | 12 | enet | 0.004 | 1.30E-01 | -1.69E+00 | 9.19E-02 |
| TK2 | 16 | 66541906 | 66586447 | 0.0510 | rs2232725 | 2.452 | rs16956585 | 0.044 | -5.040 | 1.731 | 108 | 23 | enet | 0.048 | 1.00E-05 | -1.68E+00 | 9.20E-02 |
| CD200 | 3 | 112051194 | 112081659 | 0.3915 | rs6438068 | 3.520 | rs7647411 | 0.544 | -14.150 | -1.681 | 122 | 14 | enet | 0.540 | 3.50E-64 | 1.68E+00 | 9.27E-02 |
| SACM1L | 3 | 45730548 | 45786916 | 0.0606 | rs2005227 | -3.360 | rs17279437 | 0.012 | 3.720 | -0.605 | 140 | 11 | enet | 0.002 | 2.10E-01 | -1.68E+00 | 9.32E-02 |
| ECI1 | 16 | 2289396 | 2302301 | 0.4603 | rs161426 | -2.342 | rs11645942 | 0.405 | -12.460 | -1.638 | 60 | 18 | enet | 0.390 | 9.00E-43 | 1.68E+00 | 9.35E-02 |
| CRELD2 | 22 | 50311815 | 50321188 | 0.3293 | rs28681372 | 1.990 | rs7410773 | 0.048 | -5.020 | -1.618 | 46 | 5 | enet | 0.073 | 2.60E-07 | 1.68E+00 | 9.35E-02 |
| ARL2BP | 16 | 57279010 | 57287516 | 0.2710 | rs591940 | 2.895 | rs7198865 | 0.097 | -6.150 | 0.793 | 76 | 20 | enet | 0.120 | 5.70E-12 | -1.67E+00 | 9.46E-02 |
| SMCHD1 | 18 | 2655737 | 2805015 | 0.0532 | rs12953815 | -2.410 | rs506739 | -0.001 | 3.420 | -0.690 | 152 | 11 | enet | 0.017 | 7.80E-03 | -1.67E+00 | 9.51E-02 |
| LIPT2 | 11 | 74202757 | 74204778 | 0.0812 | rs11236164 | 2.580 | rs1280088 | 0.104 | -5.610 | 1.828 | 108 | 7 | enet | 0.091 | 3.00E-07 | -1.66E+00 | 9.60E-02 |
| PNPO | 17 | 46018872 | 46025654 | 0.7245 | rs11654010 | 3.070 | rs17679445 | 0.571 | -14.970 | -1.221 | 104 | 14 | enet | 0.610 | 6.10E-77 | 1.66E+00 | 9.69E-02 |
| NEK1 | 4 | 170314426 | 170533780 | 0.1636 | rs10023181 | -3.200 | rs7678428 | 0.045 | 4.720 | -0.991 | 131 | 14 | enet | 0.046 | 5.80E-05 | -1.66E+00 | 9.76E-02 |
| CPT2 | 1 | 53662101 | 53679869 | 0.4099 | rs1288502 | 1.880 | rs11578832 | 0.433 | -12.810 | -1.630 | 127 | 11 | enet | 0.440 | 1.80E-49 | 1.65E+00 | 9.81E-02 |
| ROCK2 | 2 | 11319887 | 11488456 | 0.0674 | rs6432182 | -2.700 | rs10929728 | 0.039 | 5.050 | -0.916 | 139 | 10 | enet | 0.021 | 2.70E-03 | -1.65E+00 | 9.90E-02 |
| RHOT2 | 16 | 718086 | 724174 | 0.2166 | rs4984902 | -2.593 | rs1128550 | 0.342 | -11.280 | -1.155 | 85 | 10 | enet | 0.370 | 1.80E-38 | 1.65E+00 | 9.93E-02 |
| RAB8A | 19 | 16222439 | 16245044 | 0.1160 | rs12980138 | 2.090 | rs2233143 | 0.060 | -4.900 | 2.003 | 95 | 22 | enet | 0.052 | 4.50E-06 | -1.65E+00 | 9.97E-02 |
| FAM69A | 1 | 93307724 | 93427057 | 0.0395 | rs11164820 | 7.636 | rs2811602 | 0.005 | 3.380 | -1.626 | 94 | 7 | enet | 0.010 | 4.40E-02 | -1.64E+00 | 1.01E-01 |
| NUCB2 | 11 | 17229700 | 17371521 | 0.1530 | rs10741723 | 2.280 | rs214105 | 0.050 | -5.940 | -1.573 | 145 | 33 | enet | 0.130 | 8.40E-13 | 1.64E+00 | 1.01E-01 |
| EFNA3 | 1 | 155036224 | 155060014 | 0.0742 | rs4845710 | 2.245 | rs4045192 | 0.021 | 4.520 | -1.210 | 95 | 12 | enet | 0.038 | 9.10E-05 | -1.64E+00 | 1.02E-01 |
| TFB1M | 6 | 155578643 | 155635627 | 0.0855 | rs950994 | -3.057 | rs1334687 | 0.065 | 4.750 | 1.681 | 160 | 4 | enet | 0.027 | 2.30E-03 | 1.64E+00 | 1.02E-01 |
| CBR1 | 21 | 37442239 | 37445464 | 0.5132 | rs1028997 | 2.340 | rs2835267 | 0.130 | 7.260 | -2.045 | 115 | 20 | enet | 0.200 | 9.70E-20 | -1.63E+00 | 1.02E-01 |

| | | | | | | | | | | | | | | | | | |
|---|---|---|---|---|---|---|---|---|---|---|---|---|---|---|---|---|---|
| ACAP2 | 3 | 194995465 | 195163807 | 0.0594 | rs9825185 | -2.180 | rs6791943 | 0.013 | 4.530 | -1.238 | 115 | 7 | enet | 0.029 | 4.90E-04 | -1.63E+00 | 1.02E-01 |
| DPP7 | 9 | 140004994 | 140009629 | 0.4339 | rs3829107 | 2.290 | rs4880091 | 0.468 | -13.320 | -1.761 | 76 | 26 | enet | 0.460 | 1.80E-52 | 1.63E+00 | 1.03E-01 |
| CHCHD6 | 3 | 126423063 | 126679249 | 0.0601 | rs13326736 | 2.030 | rs9852440 | 0.046 | -5.680 | 0.774 | 174 | 17 | enet | 0.073 | 6.30E-08 | -1.63E+00 | 1.03E-01 |
| TEX264 | 3 | 51696709 | 51738339 | 0.0454 | rs12330652 | -2.470 | rs12330652 | 0.024 | -4.390 | -2.474 | 25 | 4 | enet | 0.029 | 6.30E-04 | 1.63E+00 | 1.03E-01 |
| TTC38 | 22 | 46663858 | 46689905 | 0.2680 | rs9627351 | -1.970 | rs6008495 | 0.231 | 9.280 | -1.609 | 86 | 21 | enet | 0.320 | 4.30E-33 | -1.63E+00 | 1.04E-01 |
| KIAA1211L | 2 | 99410309 | 99552722 | 0.0418 | rs6542847 | -2.503 | rs4851165 | 0.008 | 3.740 | -1.477 | 97 | 6 | enet | 0.007 | 5.90E-02 | -1.62E+00 | 1.04E-01 |
| PDLIM5 | 4 | 95373037 | 95589377 | 0.1234 | rs17336401 | 2.130 | rs2452600 | 0.080 | -5.810 | 1.687 | 148 | 19 | enet | 0.065 | 3.00E-07 | -1.62E+00 | 1.05E-01 |
| NAPB | 20 | 23355159 | 23402125 | 0.0463 | rs8615 | 1.892 | rs8615 | 0.026 | 4.450 | 1.892 | 157 | 10 | enet | 0.040 | 5.80E-05 | 1.62E+00 | 1.05E-01 |
| ACSS1 | 20 | 24986868 | 25039616 | 0.1216 | rs3746332 | 2.366 | rs7266451 | 0.019 | 4.890 | 0.311 | 128 | 18 | enet | 0.038 | 8.00E-05 | -1.62E+00 | 1.05E-01 |
| CRYL1 | 13 | 20977806 | 21099996 | 0.1225 | rs944018 | 2.840 | rs9506497 | 0.000 | -4.290 | 1.380 | 217 | 6 | enet | 0.000 | 3.40E-01 | -1.62E+00 | 1.05E-01 |
| MTPAP | 10 | 30598730 | 30663377 | 0.1349 | rs531707 | -2.180 | rs11818836 | 0.188 | -8.540 | -0.990 | 117 | 14 | enet | 0.180 | 1.70E-18 | 1.62E+00 | 1.06E-01 |
| TMEM51 | 1 | 15479028 | 15546976 | 0.1145 | rs4661594 | 2.933 | rs742668 | 0.084 | -5.660 | -1.150 | 189 | 11 | enet | 0.078 | 9.30E-08 | 1.62E+00 | 1.06E-01 |
| CDC42BPA | 1 | 227177566 | 227506175 | 0.0744 | rs4653809 | 2.481 | rs11804613 | 0.032 | 5.410 | 1.629 | 280 | 16 | enet | 0.046 | 1.70E-05 | 1.61E+00 | 1.07E-01 |
| IMPA1 | 8 | 82570196 | 82598928 | 0.1588 | rs17633897 | -2.200 | rs1967328 | 0.072 | -5.950 | -0.263 | 88 | 22 | enet | 0.096 | 4.70E-10 | 1.61E+00 | 1.07E-01 |
| ASPH | 8 | 62413116 | 62627155 | 0.0545 | rs13256118 | -2.710 | rs6471960 | 0.013 | 4.340 | -1.948 | 195 | 12 | enet | 0.025 | 1.10E-03 | -1.61E+00 | 1.07E-01 |
| DCC | 18 | 49866542 | 51057784 | 0.1040 | rs1222938 | 3.060 | rs1394466 | 0.058 | -6.590 | 0.967 | 648 | 34 | enet | 0.120 | 3.10E-12 | -1.61E+00 | 1.08E-01 |
| MAP2K2 | 19 | 4090319 | 4124126 | 0.0476 | rs7251928 | -2.750 | rs350832 | 0.020 | 4.110 | -1.516 | 37 | 6 | enet | 0.013 | 1.40E-02 | -1.61E+00 | 1.08E-01 |
| ACOT13 | 6 | 24667263 | 24705293 | 0.0518 | rs1803005 | 2.337 | rs12193165 | 0.026 | 3.720 | 1.602 | 117 | 3 | enet | 0.025 | 1.30E-03 | 1.60E+00 | 1.09E-01 |
| COQ5 | 12 | 120941077 | 120972237 | 0.1207 | rs17431717 | 4.040 | rs10774555 | 0.124 | 7.020 | -0.859 | 92 | 23 | enet | 0.110 | 1.50E-11 | -1.60E+00 | 1.09E-01 |
| UAP1L1 | 9 | 139971953 | 139978991 | 0.1739 | rs6926 | -2.380 | rs3750518 | 0.241 | -9.630 | 1.408 | 85 | 8 | enet | 0.240 | 9.20E-24 | -1.59E+00 | 1.11E-01 |
| UQCC2 | 6 | 33662070 | 33679504 | 0.0420 | rs471942 | 5.470 | rs2296748 | 0.001 | -3.790 | -0.741 | 185 | 11 | enet | 0.011 | 2.40E-02 | 1.59E+00 | 1.11E-01 |
| RDX | 11 | 110045605 | 110167447 | 0.0401 | rs2051466 | -3.420 | rs7936488 | 0.028 | 3.830 | -1.425 | 127 | 6 | enet | 0.011 | 2.30E-02 | -1.59E+00 | 1.12E-01 |
| EARS2 | 16 | 23533335 | 23569052 | 0.2385 | rs3760109 | 2.024 | rs375237 | 0.466 | -13.750 | 1.605 | 95 | 22 | enet | 0.500 | 4.80E-58 | -1.59E+00 | 1.12E-01 |
| FTSJ3 | 17 | 61896793 | 61907372 | 0.0649 | rs8067064 | 1.970 | rs28386778 | 0.120 | -5.860 | -1.441 | 68 | 11 | enet | 0.100 | 4.20E-08 | 1.59E+00 | 1.12E-01 |
| CDH19 | 18 | 64168320 | 64271375 | 0.0563 | rs11151296 | -2.640 | rs4544336 | 0.031 | -3.670 | -0.357 | 100 | 3 | enet | 0.024 | 9.10E-03 | 1.59E+00 | 1.12E-01 |
| SGIP1 | 1 | 66999066 | 67213982 | 0.0671 | rs2483704 | -2.475 | rs539014 | 0.038 | 4.700 | -1.905 | 300 | 15 | enet | 0.055 | 2.40E-06 | -1.58E+00 | 1.14E-01 |
| DNM3 | 1 | 171810621 | 172387606 | 0.1245 | rs7539972 | -3.224 | rs4916263 | 0.059 | 5.720 | -0.684 | 389 | 27 | enet | 0.086 | 3.60E-09 | -1.58E+00 | 1.14E-01 |
| GRN | 17 | 42422614 | 42430470 | 0.0596 | rs7222501 | 2.090 | rs4793105 | 0.045 | -3.580 | -1.186 | 62 | 22 | enet | 0.015 | 2.90E-02 | 1.58E+00 | 1.15E-01 |
| NDUFAF1 | 15 | 41679551 | 41694717 | 0.2612 | rs11635906 | -2.380 | rs17730589 | 0.195 | -8.690 | -1.051 | 66 | 15 | enet | 0.230 | 4.20E-23 | 1.57E+00 | 1.15E-01 |
| PDLIM2 | 8 | 22435792 | 22455538 | 0.3407 | rs2469749 | 2.130 | rs3735894 | 0.114 | -7.130 | -0.959 | 126 | 31 | enet | 0.150 | 1.50E-15 | 1.57E+00 | 1.16E-01 |
| ENDOD1 | 11 | 94822974 | 94865809 | 0.0853 | rs626481 | 2.520 | rs641330 | 0.006 | -3.630 | 0.112 | 132 | 27 | enet | -0.002 | 5.60E-01 | -1.57E+00 | 1.16E-01 |
| ALAD | 9 | 116148597 | 116163613 | 0.4486 | rs4979240 | 3.060 | rs818702 | 0.032 | -5.270 | 0.168 | 119 | 18 | enet | 0.190 | 1.20E-18 | -1.57E+00 | 1.16E-01 |
| L2HGDH | 14 | 50704281 | 50779266 | 0.1047 | rs4901030 | 2.060 | rs12894758 | 0.093 | -6.250 | 1.673 | 109 | 13 | enet | 0.090 | 1.60E-09 | -1.57E+00 | 1.17E-01 |
| ALG5 | 13 | 37523912 | 37574398 | 0.0664 | rs515397 | -2.470 | rs2243710 | 0.048 | 4.610 | 1.430 | 108 | 15 | enet | 0.033 | 3.10E-04 | 1.57E+00 | 1.17E-01 |
| CD14 | 5 | 140011313 | 140013286 | 0.0426 | rs34282994 | -2.537 | rs778587 | 0.035 | 3.800 | -1.429 | 75 | 13 | enet | 0.011 | 2.20E-02 | -1.57E+00 | 1.18E-01 |
| ETFB | 19 | 51848423 | 51869672 | 0.0638 | rs11084071 | 2.410 | rs8102334 | 0.023 | -4.430 | -1.273 | 106 | 7 | enet | 0.021 | 2.60E-03 | 1.56E+00 | 1.18E-01 |
| COX17 | 3 | 119373360 | 119396301 | 0.0705 | rs4688029 | -2.490 | rs34775086 | 0.043 | 4.020 | -1.639 | 119 | 5 | enet | 0.008 | 4.90E-02 | -1.56E+00 | 1.18E-01 |
| LRRC40 | 1 | 70610488 | 70671303 | 0.0796 | rs12033206 | -3.091 | rs476591 | 0.088 | -6.030 | 1.219 | 98 | 21 | enet | 0.088 | 2.80E-09 | -1.56E+00 | 1.19E-01 |
| AGL | 1 | 100315640 | 100389579 | 0.6233 | rs1541042 | 2.012 | rs3818568 | 0.429 | -13.100 | 1.054 | 138 | 33 | enet | 0.560 | 6.80E-70 | -1.56E+00 | 1.19E-01 |
| TMEM65 | 8 | 125324231 | 125384933 | 0.0850 | rs9886564 | 3.180 | rs10089507 | 0.003 | 4.510 | -0.710 | 115 | 28 | enet | 0.006 | 7.00E-02 | -1.55E+00 | 1.20E-01 |
| RNMTL1 | 17 | 685513 | 695749 | 0.1557 | rs9892880 | 2.430 | rs3087833 | 0.097 | -6.260 | -0.561 | 101 | 14 | enet | 0.120 | 7.30E-12 | 1.56E+00 | 1.20E-01 |
| TXNL4A | 18 | 77732867 | 77793949 | 0.0617 | rs7505298 | 2.160 | rs4799113 | 0.034 | 4.440 | -1.208 | 109 | 22 | enet | 0.028 | 1.00E-03 | -1.55E+00 | 1.20E-01 |
| CNNM2 | 10 | 104678050 | 104849978 | 0.1453 | rs1163249 | -2.410 | rs11191454 | 0.089 | 6.110 | -1.341 | 132 | 15 | enet | 0.096 | 7.30E-10 | -1.55E+00 | 1.20E-01 |
| THBS4 | 5 | 79287134 | 79379110 | 0.0764 | rs4703803 | -3.080 | rs4703797 | 0.105 | 6.110 | 1.586 | 143 | 5 | enet | 0.083 | 2.70E-07 | 1.55E+00 | 1.21E-01 |
| DBNL | 7 | 44084239 | 44109055 | 0.3936 | rs2527813 | -2.140 | rs1894752 | 0.180 | 8.380 | -1.776 | 71 | 23 | enet | 0.240 | 3.80E-24 | -1.55E+00 | 1.21E-01 |
| NDUFB2 | 7 | 140390577 | 140422590 | 0.0503 | rs269267 | -2.650 | rs6464036 | 0.001 | 4.320 | -0.372 | 61 | 36 | enet | 0.002 | 2.20E-01 | -1.55E+00 | 1.21E-01 |

| | | | | | | | | | | | | | | | | | |
|---|---|---|---|---|---|---|---|---|---|---|---|---|---|---|---|---|---|
| THUMPD3 | 3 | 9404526 | 9428475 | 0.2425 | rs7642020 | -2.800 | rs3898760 | 0.033 | -5.140 | -2.106 | 98 | 30 | enet | 0.140 | 1.80E-13 | 1.55E+00 | 1.22E-01 |
| PGS1 | 17 | 76374721 | 76421195 | 0.1062 | rs6501192 | -2.710 | rs2292645 | 0.092 | 5.960 | -1.582 | 140 | 23 | enet | 0.100 | 2.90E-10 | -1.55E+00 | 1.22E-01 |
| GGA3 | 17 | 73232694 | 73258444 | 0.1302 | rs9900586 | 4.860 | rs4789161 | 0.038 | -4.100 | 2.792 | 50 | 8 | enet | 0.049 | 8.60E-06 | -1.54E+00 | 1.22E-01 |
| MPPED2 | 11 | 30406040 | 30608419 | 0.0914 | rs7952660 | -2.340 | rs11031118 | 0.021 | -3.840 | 0.758 | 191 | 31 | enet | 0.038 | 1.00E-03 | 1.54E+00 | 1.23E-01 |
| NPEPL1 | 20 | 57264187 | 57294294 | 0.0729 | rs4812024 | 1.954 | rs6015338 | 0.016 | -3.840 | 1.507 | 156 | 12 | enet | 0.029 | 5.10E-04 | -1.54E+00 | 1.23E-01 |
| SEC14L2 | 22 | 30792846 | 30821305 | 0.1641 | rs4339043 | 2.870 | rs2240422 | 0.055 | -5.850 | -0.949 | 130 | 17 | enet | 0.074 | 5.00E-08 | -1.54E+00 | 1.23E-01 |
| DUT | 15 | 48623208 | 48635570 | 0.0273 | rs971952 | 1.820 | rs3784619 | 0.026 | -3.710 | 1.788 | 85 | 13 | enet | 0.013 | 1.60E-02 | -1.54E+00 | 1.23E-01 |
| C9orf78 | 9 | 132589569 | 132598142 | 0.0611 | rs7857431 | 2.990 | rs3844048 | 0.002 | 3.470 | -1.499 | 116 | 22 | enet | 0.016 | 1.00E-02 | -1.54E+00 | 1.24E-01 |
| GFM2 | 5 | 74017029 | 74063196 | 0.3732 | rs17561000 | 1.848 | rs6453075 | 0.459 | 13.280 | -1.284 | 119 | 25 | enet | 0.530 | 2.90E-64 | -1.54E+00 | 1.24E-01 |
| STX2 | 12 | 131274145 | 131323811 | 0.0468 | rs4412792 | 2.560 | rs10773819 | 0.034 | 4.710 | -1.624 | 121 | 7 | enet | 0.017 | 7.00E-03 | -1.54E+00 | 1.24E-01 |
| LNPEP | 5 | 96271098 | 96373219 | 0.0943 | rs12054720 | 2.684 | rs18059 | 0.068 | 6.340 | 1.953 | 123 | 21 | enet | 0.076 | 3.40E-08 | 1.53E+00 | 1.25E-01 |
| ANO5 | 11 | 22214722 | 22304903 | 0.3133 | rs11026477 | -2.390 | rs4275631 | 0.267 | 7.970 | 0.012 | 89 | 33 | enet | 0.340 | 9.30E-21 | 1.53E+00 | 1.25E-01 |
| SLC25A22 | 11 | 790475 | 798316 | 0.1364 | rs12796837 | -1.590 | rs7946354 | 0.038 | -4.900 | 0.625 | 101 | 12 | enet | 0.061 | 6.80E-07 | -1.54E+00 | 1.25E-01 |
| ERCC2 | 19 | 45853095 | 45874176 | 0.0406 | rs238406 | 2.050 | rs1793978 | 0.018 | -4.060 | -1.176 | 65 | 12 | enet | 0.024 | 5.60E-03 | 1.53E+00 | 1.27E-01 |
| PFKP | 10 | 3108525 | 3179904 | 0.1515 | rs7073415 | -2.160 | rs2306302 | 0.001 | -3.550 | 1.223 | 229 | 31 | enet | 0.009 | 3.90E-02 | -1.53E+00 | 1.27E-01 |
| ADO | 10 | 64564516 | 64568238 | 0.1837 | rs10509174 | -3.560 | rs1848797 | 0.231 | -9.410 | -1.159 | 104 | 21 | enet | 0.210 | 1.90E-21 | 1.53E+00 | 1.27E-01 |
| BCL2L13 | 22 | 18111621 | 18213388 | 0.2041 | rs5747338 | 3.320 | rs2535708 | 0.223 | -9.320 | -2.192 | 161 | 24 | enet | 0.240 | 8.20E-25 | 1.53E+00 | 1.27E-01 |
| PPID | 4 | 159630286 | 159644548 | 0.9196 | rs6815665 | -2.480 | rs17843966 | 0.341 | 11.360 | 0.608 | 29 | 11 | enet | 0.600 | 4.40E-76 | 1.52E+00 | 1.28E-01 |
| SCRN3 | 2 | 175260458 | 175294303 | 0.4427 | rs12151707 | -2.179 | rs7570614 | 0.194 | -9.640 | 1.457 | 71 | 28 | enet | 0.460 | 4.40E-53 | -1.52E+00 | 1.28E-01 |
| NRIP2 | 12 | 2934514 | 2944710 | 0.0556 | rs2109108 | -2.080 | rs6489402 | 0.005 | -3.590 | -1.577 | 83 | 17 | enet | 0.022 | 2.30E-03 | 1.52E+00 | 1.28E-01 |
| MSMO1 | 4 | 166248775 | 166264312 | 0.0415 | rs12330937 | -2.690 | rs12513198 | -0.001 | 3.520 | 0.613 | 100 | 8 | enet | 0.012 | 2.60E-02 | 1.52E+00 | 1.29E-01 |
| VWA5A | 11 | 123986069 | 124018428 | 0.2294 | rs1939852 | 2.840 | rs2508888 | 0.150 | -7.580 | 1.638 | 140 | 14 | enet | 0.200 | 6.80E-20 | -1.52E+00 | 1.29E-01 |
| PTGR2 | 14 | 74318547 | 74353530 | 0.7097 | rs2336547 | 1.870 | rs8020267 | 0.310 | 11.250 | 1.136 | 106 | 33 | enet | 0.440 | 7.40E-50 | 1.51E+00 | 1.30E-01 |
| STX4 | 16 | 31044210 | 31054296 | 0.0469 | rs4889603 | 3.086 | rs729482 | 0.018 | -4.020 | -2.640 | 66 | 8 | enet | 0.034 | 1.80E-04 | 1.52E+00 | 1.30E-01 |
| MLF1 | 3 | 158288952 | 158325041 | 0.0610 | rs6441205 | 2.610 | rs7631310 | 0.027 | -4.870 | -0.695 | 85 | 22 | enet | 0.035 | 6.50E-04 | 1.51E+00 | 1.30E-01 |
| GAK | 4 | 843064 | 926161 | 0.0612 | rs11946340 | 3.840 | rs6843423 | 0.035 | -4.900 | -1.720 | 148 | 15 | enet | 0.026 | 1.00E-03 | 1.51E+00 | 1.30E-01 |
| EGFR | 7 | 55086714 | 55324313 | 0.0680 | rs10244108 | -3.320 | rs763317 | 0.005 | -4.300 | -2.663 | 216 | 10 | enet | 0.038 | 8.80E-05 | 1.51E+00 | 1.30E-01 |
| BCS1L | 2 | 219523487 | 219528166 | 0.0819 | rs11896209 | 2.010 | rs3845836 | 0.016 | -3.940 | 1.449 | 87 | 24 | enet | 0.041 | 4.80E-05 | -1.51E+00 | 1.31E-01 |
| GDI2 | 10 | 5807186 | 5884095 | 0.1034 | rs2380204 | 3.180 | rs10795547 | 0.065 | -5.680 | -0.914 | 165 | 12 | enet | 0.063 | 4.80E-07 | 1.51E+00 | 1.32E-01 |
| TECPR1 | 7 | 97843936 | 97881563 | 0.2667 | rs12531245 | 1.880 | rs6969321 | 0.096 | -6.210 | 1.577 | 90 | 9 | enet | 0.110 | 3.20E-11 | -1.51E+00 | 1.32E-01 |
| DIP2B | 12 | 50898768 | 51142450 | 0.0768 | rs1047912 | -3.310 | rs2251603 | 0.063 | -5.550 | 1.602 | 114 | 15 | enet | 0.063 | 5.20E-07 | -1.51E+00 | 1.32E-01 |
| GLUD1 | 10 | 88810243 | 88854623 | 0.0344 | rs1240386 | 2.250 | rs4933426 | 0.028 | -4.010 | -1.473 | 61 | 5 | enet | 0.018 | 5.30E-03 | 1.51E+00 | 1.32E-01 |
| SND1 | 7 | 127292234 | 127732661 | 0.0316 | rs10954160 | 2.930 | rs17151739 | 0.011 | -3.640 | 1.943 | 244 | 16 | enet | 0.003 | 1.50E-01 | -1.51E+00 | 1.32E-01 |
| DHRS7 | 14 | 60610838 | 60636574 | 0.0658 | rs12431948 | -1.740 | rs441282 | 0.104 | -6.790 | -1.550 | 79 | 25 | enet | 0.110 | 1.50E-11 | 1.50E+00 | 1.33E-01 |
| ADH5 | 4 | 99992132 | 100009952 | 0.1864 | rs2602884 | -1.980 | rs6827292 | 0.066 | -6.210 | -1.051 | 132 | 20 | enet | 0.099 | 2.30E-10 | 1.50E+00 | 1.34E-01 |
| GOPC | 6 | 117639374 | 117923691 | 0.2337 | rs2243380 | 2.695 | rs210643 | 0.199 | 8.820 | -1.078 | 241 | 29 | enet | 0.200 | 2.20E-20 | -1.50E+00 | 1.34E-01 |
| SLC7A8 | 14 | 23594504 | 23652883 | 0.3400 | rs10132368 | 1.920 | rs3783436 | 0.255 | -9.880 | -1.479 | 112 | 12 | enet | 0.230 | 2.70E-23 | 1.50E+00 | 1.34E-01 |
| NOSTRIN | 2 | 169643049 | 169722024 | 0.0820 | rs506273 | -2.999 | rs540652 | 0.025 | 4.570 | -2.016 | 180 | 14 | enet | 0.025 | 8.90E-03 | -1.49E+00 | 1.35E-01 |
| CHKB | 22 | 51017378 | 51039884 | 0.0847 | rs140522 | 6.310 | rs131749 | 0.000 | -3.400 | 1.782 | 102 | 28 | enet | 0.007 | 5.30E-02 | -1.49E+00 | 1.36E-01 |
| OPA3 | 19 | 46030685 | 46105470 | 0.0302 | rs2287019 | -3.450 | rs8112209 | -0.001 | -3.550 | -0.642 | 105 | 9 | enet | 0.009 | 4.10E-02 | -1.49E+00 | 1.37E-01 |
| LXN | 3 | 158363611 | 158390482 | 0.1904 | rs7617304 | 3.030 | rs3867391 | 0.228 | 9.690 | 0.783 | 93 | 20 | enet | 0.280 | 5.20E-27 | 1.48E+00 | 1.38E-01 |
| GDAP1 | 8 | 75233365 | 75401107 | 0.1021 | rs4464961 | -3.450 | rs16938888 | 0.067 | 5.490 | 0.875 | 146 | 22 | enet | 0.062 | 6.10E-07 | 1.48E+00 | 1.38E-01 |
| N6AMT1 | 21 | 30244513 | 30257693 | 0.4108 | rs2254796 | 2.340 | rs7282280 | 0.344 | -10.260 | -1.145 | 95 | 34 | enet | 0.390 | 1.00E-32 | 1.48E+00 | 1.39E-01 |
| ETHE1 | 19 | 44010871 | 44031396 | 0.2157 | rs11083711 | -2.810 | rs2102954 | 0.168 | -8.140 | -1.224 | 111 | 15 | enet | 0.210 | 1.30E-21 | 1.48E+00 | 1.40E-01 |
| FARSB | 2 | 223435255 | 223521056 | 0.1188 | rs6436320 | 3.025 | rs12694580 | 0.060 | -7.000 | 1.123 | 140 | 17 | enet | 0.140 | 1.40E-14 | -1.48E+00 | 1.40E-01 |
| LPCAT2 | 16 | 55542910 | 55620582 | 0.1842 | rs17841329 | -3.029 | rs1393257 | 0.243 | 9.540 | -1.329 | 191 | 40 | enet | 0.210 | 3.50E-21 | -1.48E+00 | 1.40E-01 |

| | | | | | | | | | | | | | | | | | | |
|---|---|---|---|---|---|---|---|---|---|---|---|---|---|---|---|---|---|---|
| SNUPN | 15 | 75890424 | 75918810 | 0.0860 | rs8041033 | -1.690 | rs8027749 | 0.037 | -4.160 | 0.904 | 68 | 21 | enet | 0.021 | 7.00E-03 | -1.47E+00 | 1.41E-01 |
| RASA4B | 7 | 102122892 | 102158228 | 0.0878 | rs6976282 | 3.040 | rs4342524 | 0.032 | 4.680 | -2.355 | 39 | 18 | enet | 0.055 | 4.20E-06 | -1.47E+00 | 1.41E-01 |
| PPP3CC | 8 | 22298332 | 22398652 | 0.0792 | rs7014360 | 2.770 | rs2469747 | 0.142 | -7.870 | -1.369 | 160 | 9 | enet | 0.140 | 8.40E-14 | 1.46E+00 | 1.43E-01 |
| MLYCD | 16 | 83932731 | 83949787 | 0.1437 | rs11641198 | 2.210 | rs9923900 | 0.135 | -7.320 | 1.768 | 165 | 6 | enet | 0.120 | 1.00E-12 | -1.46E+00 | 1.44E-01 |
| RILPL1 | 12 | 123955925 | 124018265 | 0.0813 | rs4930718 | 5.130 | rs28647680 | -0.001 | 3.440 | 2.759 | 90 | 21 | enet | 0.008 | 4.60E-02 | 1.46E+00 | 1.45E-01 |
| TXNRD1 | 12 | 104609557 | 104744061 | 0.1152 | rs7978310 | 2.240 | rs10861197 | 0.101 | 6.510 | 1.751 | 151 | 14 | enet | 0.110 | 5.90E-11 | 1.46E+00 | 1.45E-01 |
| NUDT12 | 5 | 102884556 | 102898494 | 0.0983 | rs10900867 | 3.299 | rs7723689 | 0.016 | -4.420 | -0.473 | 96 | 13 | enet | 0.013 | 1.70E-02 | 1.45E+00 | 1.46E-01 |
| OPA1 | 3 | 193310933 | 193415612 | 0.0807 | rs6764269 | -1.810 | rs1056392 | 0.019 | -3.830 | 1.407 | 135 | 11 | enet | 0.023 | 1.80E-03 | -1.45E+00 | 1.48E-01 |
| ITGA6 | 2 | 173292082 | 173371181 | 0.1520 | rs11904366 | 2.706 | rs6433355 | 0.041 | 5.400 | 0.415 | 157 | 27 | enet | 0.066 | 2.30E-07 | 1.44E+00 | 1.49E-01 |
| AKAP12 | 6 | 151561134 | 151679692 | 0.1262 | rs1535831 | -2.550 | rs10499265 | 0.016 | -4.470 | 0.777 | 192 | 26 | enet | 0.054 | 3.00E-06 | -1.44E+00 | 1.49E-01 |
| REXO2 | 11 | 114310108 | 114321001 | 0.0890 | rs4938094 | -1.910 | rs11824249 | 0.007 | 3.890 | -1.443 | 102 | 12 | enet | 0.023 | 1.70E-03 | -1.44E+00 | 1.49E-01 |
| CHORDC1 | 11 | 89934328 | 89956532 | 0.0797 | rs7122533 | -2.610 | rs647248 | 0.006 | 4.130 | 1.417 | 85 | 9 | enet | 0.034 | 2.00E-04 | 1.44E+00 | 1.50E-01 |
| GSKIP | 14 | 96829814 | 96853625 | 0.0295 | rs2275554 | 2.600 | rs1545280 | 0.038 | 4.440 | -1.554 | 91 | 5 | enet | 0.027 | 8.20E-04 | -1.44E+00 | 1.51E-01 |
| LIMK1 | 7 | 73497263 | 73536855 | 0.0519 | rs810531 | 2.520 | rs6460071 | 0.000 | 3.790 | -0.739 | 87 | 9 | enet | -0.001 | 4.20E-01 | -1.43E+00 | 1.51E-01 |
| PPCDC | 15 | 75315896 | 75409803 | 0.0714 | rs11072517 | 2.990 | rs4886649 | 0.019 | -4.480 | -1.712 | 80 | 11 | enet | 0.050 | 8.10E-05 | 1.43E+00 | 1.52E-01 |
| AAGAB | 15 | 67493371 | 67547533 | 0.0523 | rs11635391 | -2.250 | rs8025774 | 0.029 | -4.970 | 1.532 | 121 | 8 | enet | 0.026 | 1.50E-03 | -1.43E+00 | 1.53E-01 |
| KCNK15 | 20 | 43374421 | 43379675 | 0.0559 | rs8121830 | -3.005 | rs967689 | 0.008 | -3.590 | -1.345 | 70 | 8 | enet | 0.018 | 1.70E-02 | 1.43E+00 | 1.53E-01 |
| PVRL1 | 11 | 119494120 | 119599794 | 0.0484 | rs12421432 | 3.270 | rs931953 | 0.044 | -4.680 | 0.861 | 136 | 6 | enet | 0.044 | 2.20E-05 | -1.42E+00 | 1.54E-01 |
| CWF19L1 | 10 | 101992055 | 102027437 | 0.1539 | rs677603 | 2.080 | rs11597086 | 0.199 | 8.490 | -1.376 | 107 | 8 | enet | 0.170 | 1.10E-16 | -1.42E+00 | 1.55E-01 |
| SQRDL | 15 | 45923346 | 45983492 | 0.2127 | rs4774709 | -2.440 | rs1044032 | 0.170 | -8.100 | 1.093 | 109 | 24 | enet | 0.150 | 1.30E-15 | -1.42E+00 | 1.55E-01 |
| YWHAB | 20 | 43514317 | 43537173 | 0.0594 | rs6876 | 3.133 | rs2425684 | 0.033 | -3.910 | 2.009 | 86 | 17 | enet | 0.025 | 1.40E-03 | -1.42E+00 | 1.57E-01 |
| TRAPPC5 | 19 | 7745729 | 7747744 | 0.1003 | rs1010046 | -2.460 | rs17159840 | 0.032 | -4.180 | 1.054 | 103 | 21 | enet | 0.029 | 4.80E-04 | -1.41E+00 | 1.59E-01 |
| ACSF3 | 16 | 89154783 | 89222254 | 0.5069 | rs7198060 | 3.958 | rs4785602 | 0.048 | 5.560 | 2.198 | 79 | 31 | enet | 0.290 | 1.40E-29 | 1.41E+00 | 1.59E-01 |
| MKRN2 | 3 | 12598513 | 12625212 | 0.1802 | rs1532534 | 2.420 | rs11710163 | 0.069 | 5.880 | -1.636 | 84 | 31 | enet | 0.110 | 2.70E-11 | -1.41E+00 | 1.59E-01 |
| GALM | 2 | 38893052 | 38968379 | 0.5354 | rs7578356 | 1.864 | rs3821023 | 0.566 | 14.850 | -1.394 | 83 | 13 | enet | 0.660 | 7.00E-90 | -1.40E+00 | 1.61E-01 |
| CCDC127 | 5 | 196986 | 218330 | 0.3373 | rs6883247 | 2.129 | rs9763161 | 0.201 | 8.750 | -0.877 | 110 | 24 | enet | 0.220 | 3.10E-22 | -1.40E+00 | 1.61E-01 |
| LACE1 | 6 | 108616098 | 108847999 | 0.0873 | rs17598747 | -2.277 | rs9372187 | 0.011 | -3.630 | 1.257 | 115 | 22 | enet | 0.024 | 1.50E-03 | -1.40E+00 | 1.62E-01 |
| ARHGAP32 | 11 | 128834955 | 129149219 | 0.0352 | rs12293164 | 3.060 | rs10893942 | 0.034 | -4.600 | 1.461 | 235 | 8 | enet | 0.017 | 6.30E-03 | -1.40E+00 | 1.63E-01 |
| ANPEP | 15 | 90328120 | 90358633 | 0.0783 | rs10852122 | -2.550 | rs6496609 | 0.011 | 3.760 | 1.835 | 104 | 13 | enet | 0.067 | 3.40E-07 | 1.39E+00 | 1.63E-01 |
| PON1 | 7 | 94926988 | 95025673 | 0.1563 | rs11762113 | 2.260 | rs757158 | 0.141 | 7.210 | 1.689 | 171 | 10 | enet | 0.120 | 8.80E-11 | 1.39E+00 | 1.63E-01 |
| DHRS4 | 14 | 24422795 | 24438488 | 0.4422 | rs10142648 | -2.540 | rs8022613 | 0.366 | -12.110 | -1.145 | 62 | 18 | enet | 0.500 | 6.20E-57 | 1.39E+00 | 1.64E-01 |
| HIBADH | 7 | 27565061 | 27702614 | 0.3514 | rs2110524 | 2.450 | rs1404282 | 0.244 | 9.970 | 1.590 | 120 | 29 | enet | 0.320 | 2.00E-33 | 1.39E+00 | 1.65E-01 |
| TSTD1 | 1 | 161007421 | 161008780 | 0.2177 | rs4656971 | -3.411 | rs11265548 | 0.065 | 7.140 | 2.449 | 88 | 17 | enet | 0.190 | 1.70E-19 | 1.39E+00 | 1.65E-01 |
| MVD | 16 | 88718343 | 88729569 | 0.3394 | rs3794624 | -3.594 | rs4782395 | 0.067 | -6.870 | 2.747 | 130 | 11 | enet | 0.190 | 7.10E-19 | -1.39E+00 | 1.65E-01 |
| GYS1 | 19 | 49471382 | 49496567 | 0.1376 | rs4347731 | -3.420 | rs1039442 | 0.067 | 5.340 | 1.450 | 86 | 10 | enet | 0.072 | 7.50E-08 | 1.39E+00 | 1.66E-01 |
| DSTYK | 1 | 205111632 | 205180727 | 0.0437 | rs12067709 | -3.083 | rs2242000 | 0.013 | -4.080 | -1.775 | 146 | 8 | enet | 0.019 | 4.50E-03 | 1.39E+00 | 1.66E-01 |
| SOD3 | 4 | 24791534 | 24802464 | 0.1728 | rs8192287 | -2.970 | rs2284659 | 0.127 | 7.110 | -1.312 | 104 | 26 | enet | 0.110 | 3.60E-11 | -1.38E+00 | 1.66E-01 |
| MTFMT | 15 | 65294845 | 65321977 | 0.1239 | rs1631677 | 2.660 | rs4776654 | 0.011 | 3.560 | -0.720 | 75 | 8 | enet | 0.051 | 1.50E-05 | -1.38E+00 | 1.67E-01 |
| MRS2 | 6 | 24403153 | 24425810 | 0.3311 | rs1321820 | 2.125 | rs1321820 | 0.140 | 7.810 | 2.125 | 141 | 20 | enet | 0.260 | 3.00E-24 | 1.38E+00 | 1.67E-01 |
| AP4E1 | 15 | 51200869 | 51298097 | 0.0991 | rs10744956 | -2.790 | rs11630203 | 0.062 | 5.430 | 1.367 | 87 | 4 | enet | 0.048 | 6.80E-05 | 1.38E+00 | 1.67E-01 |
| ALDH2 | 12 | 112204691 | 112247782 | 0.0701 | rs11066028 | -2.340 | rs4648328 | 0.146 | -7.990 | -1.380 | 61 | 12 | enet | 0.140 | 5.00E-14 | 1.38E+00 | 1.68E-01 |
| ERO1L | 14 | 53106634 | 53162618 | 0.0327 | rs11620764 | 1.930 | rs12892461 | 0.004 | 3.660 | -1.230 | 77 | 13 | enet | 0.016 | 8.10E-03 | -1.38E+00 | 1.69E-01 |
| C20orf194 | 20 | 3229951 | 3388272 | 0.0942 | rs6051812 | 2.157 | rs910768 | 0.002 | -3.430 | -0.415 | 140 | 13 | enet | 0.005 | 1.10E-01 | 1.38E+00 | 1.69E-01 |
| RECQL | 12 | 21621845 | 21654603 | 0.3894 | rs11046127 | 2.540 | rs7295965 | 0.088 | -6.450 | 0.467 | 166 | 33 | enet | 0.190 | 1.20E-18 | -1.37E+00 | 1.71E-01 |
| EPN2 | 17 | 19118928 | 19240028 | 0.0293 | rs2048230 | -1.790 | rs2233072 | 0.031 | 3.630 | -1.465 | 37 | 4 | enet | 0.004 | 1.20E-01 | -1.37E+00 | 1.71E-01 |
| HMBS | 11 | 118955576 | 118964259 | 0.1422 | rs4936459 | -3.250 | rs673768 | 0.048 | -5.540 | -0.215 | 96 | 29 | enet | 0.056 | 2.20E-06 | 1.37E+00 | 1.72E-01 |

| | | | | | | | | | | | | | | | | | |
|---|---|---|---|---|---|---|---|---|---|---|---|---|---|---|---|---|---|
| SUSD5 | 3 | 33191537 | 33260707 | 0.2671 | rs4392367 | -3.850 | rs10222597 | 0.145 | 7.430 | -0.340 | 106 | 15 | enet | 0.200 | 4.00E-20 | 1.37E+00 | 1.72E-01 |
| ARSA | 22 | 51061182 | 51066607 | 0.3284 | rs140522 | 6.310 | rs6151429 | 0.183 | -8.760 | 0.794 | 99 | 17 | enet | 0.220 | 5.30E-21 | -1.36E+00 | 1.73E-01 |
| GMFB | 14 | 54941202 | 54955914 | 0.0473 | rs8015391 | -2.500 | rs9323267 | 0.036 | 4.540 | -1.831 | 66 | 12 | enet | 0.016 | 7.90E-03 | -1.36E+00 | 1.73E-01 |
| CTBS | 1 | 85015289 | 85040163 | 0.1457 | rs10874453 | -2.721 | rs2911602 | 0.040 | 5.040 | 0.867 | 114 | 14 | enet | 0.092 | 1.30E-07 | 1.36E+00 | 1.74E-01 |
| LMCD1 | 3 | 8543393 | 8609805 | 0.0837 | rs12637725 | -2.560 | rs9810512 | 0.049 | -5.530 | -1.631 | 202 | 11 | enet | 0.051 | 6.90E-06 | 1.36E+00 | 1.75E-01 |
| SDR39U1 | 14 | 24908972 | 24912111 | 0.2547 | rs11846223 | -2.880 | rs10140911 | 0.085 | -6.220 | 0.544 | 152 | 28 | enet | 0.100 | 1.70E-10 | -1.36E+00 | 1.75E-01 |
| S100A4 | 1 | 153516089 | 153522612 | 0.0387 | rs7549308 | -2.244 | rs8401 | 0.034 | 4.720 | -1.335 | 72 | 7 | enet | 0.028 | 6.90E-04 | -1.35E+00 | 1.76E-01 |
| ADPGK | 15 | 73043710 | 73078187 | 0.1032 | rs11637927 | -3.530 | rs11072380 | 0.063 | -5.410 | 1.088 | 67 | 5 | enet | 0.054 | 7.00E-06 | -1.35E+00 | 1.77E-01 |
| FAM160B2 | 8 | 21946670 | 21962409 | 0.0470 | rs1552286 | -3.440 | rs7823952 | 0.015 | -4.210 | 0.905 | 91 | 8 | enet | 0.005 | 9.10E-02 | -1.35E+00 | 1.77E-01 |
| CPNE2 | 16 | 57126449 | 57181878 | 0.0778 | rs7192652 | -4.584 | rs291053 | 0.044 | -4.840 | -0.598 | 145 | 13 | enet | 0.039 | 6.90E-05 | 1.35E+00 | 1.77E-01 |
| COQ7 | 16 | 19078921 | 19091417 | 0.3162 | rs12918623 | 2.235 | rs12935719 | 0.311 | 10.940 | 1.475 | 84 | 14 | enet | 0.320 | 1.10E-32 | 1.35E+00 | 1.77E-01 |
| LONP1 | 19 | 5691845 | 5720583 | 0.1985 | rs17205911 | -3.000 | rs3892355 | 0.107 | -7.560 | 1.069 | 80 | 20 | enet | 0.160 | 3.60E-16 | -1.35E+00 | 1.77E-01 |
| NT5C3A | 7 | 33053742 | 33102409 | 0.1143 | rs9690597 | 2.660 | rs3915352 | 0.065 | 5.580 | -1.538 | 101 | 20 | enet | 0.092 | 1.10E-09 | -1.35E+00 | 1.78E-01 |
| RAB3B | 1 | 52373628 | 52456436 | 0.0858 | rs2795002 | -1.997 | rs2809949 | 0.030 | 4.580 | 0.016 | 67 | 42 | enet | 0.035 | 1.60E-04 | 1.35E+00 | 1.78E-01 |
| NUDCD1 | 8 | 110253148 | 110346614 | 0.4043 | rs16879302 | 2.840 | rs7012304 | 0.139 | 7.390 | 1.938 | 90 | 25 | enet | 0.170 | 1.70E-17 | 1.35E+00 | 1.78E-01 |
| CRYBG3 | 3 | 97595819 | 97663810 | 0.0911 | rs41527949 | -1.790 | rs1492030 | 0.061 | 4.390 | -1.698 | 104 | 18 | enet | 0.045 | 7.60E-04 | -1.35E+00 | 1.78E-01 |
| FAM21C | 10 | 46222648 | 46288409 | 0.0701 | rs3802542 | -1.700 | rs7091141 | 0.058 | -4.800 | 1.640 | 22 | 19 | enet | 0.041 | 1.10E-04 | -1.34E+00 | 1.80E-01 |
| KATNAL1 | 13 | 30776767 | 30881621 | 0.0917 | rs9506295 | -2.690 | rs756850 | 0.003 | -3.550 | -1.883 | 149 | 21 | enet | 0.030 | 4.00E-04 | 1.34E+00 | 1.80E-01 |
| FBP1 | 9 | 97365415 | 97402531 | 0.1784 | rs2274689 | 3.920 | rs16910850 | 0.110 | 5.990 | -0.678 | 112 | 27 | enet | 0.100 | 1.50E-08 | -1.34E+00 | 1.82E-01 |
| IGFBP2 | 2 | 217497551 | 217529159 | 0.0794 | rs12463731 | -1.896 | rs4674091 | 0.017 | 4.490 | 0.888 | 119 | 3 | enet | 0.018 | 6.20E-03 | 1.33E+00 | 1.82E-01 |
| FAM134A | 2 | 220040947 | 220050201 | 0.0951 | rs3210652 | 1.701 | rs3210652 | 0.103 | -6.360 | 1.701 | 89 | 11 | enet | 0.095 | 6.40E-10 | -1.33E+00 | 1.82E-01 |
| ACYP1 | 14 | 75519924 | 75536186 | 0.1371 | rs28756981 | 1.870 | rs10142770 | 0.156 | 8.050 | 1.220 | 70 | 20 | enet | 0.130 | 2.90E-13 | 1.33E+00 | 1.83E-01 |
| UPF3A | 13 | 115047059 | 115071283 | 0.1662 | rs7999630 | 1.830 | rs9525332 | 0.168 | -7.440 | -0.977 | 59 | 10 | enet | 0.170 | 9.80E-13 | 1.33E+00 | 1.84E-01 |
| C2CD2L | 11 | 118972908 | 118989252 | 0.1173 | rs4936459 | -3.250 | rs4938626 | 0.116 | -6.710 | -1.468 | 102 | 5 | enet | 0.120 | 3.40E-12 | 1.33E+00 | 1.85E-01 |
| MGST1 | 12 | 16500076 | 16762193 | 0.3535 | rs1042669 | 2.720 | rs2239675 | 0.063 | 6.290 | 2.483 | 184 | 29 | enet | 0.200 | 4.20E-20 | 1.33E+00 | 1.85E-01 |
| DOCK7 | 1 | 62920399 | 63153969 | 0.0689 | rs1168113 | 2.250 | rs10889325 | 0.039 | 4.260 | -0.186 | 123 | 11 | enet | 0.050 | 7.30E-06 | -1.32E+00 | 1.86E-01 |
| CAMK1G | 1 | 209757062 | 209787283 | 0.0586 | rs6673693 | 2.170 | rs926386 | 0.056 | -5.110 | 1.348 | 164 | 7 | enet | 0.047 | 2.00E-05 | -1.32E+00 | 1.87E-01 |
| CYP7B1 | 8 | 65500320 | 65711318 | 0.0279 | rs2218914 | -1.880 | rs4406437 | 0.028 | 4.390 | -1.517 | 145 | 14 | enet | 0.007 | 6.40E-02 | -1.32E+00 | 1.87E-01 |
| CTSD | 11 | 1773982 | 1785222 | 0.1213 | rs4752733 | -2.690 | rs2334411 | 0.001 | -3.660 | 0.383 | 88 | 19 | enet | 0.024 | 1.40E-03 | -1.32E+00 | 1.87E-01 |
| RPL13 | 16 | 89627065 | 89630950 | 0.0490 | rs154659 | -4.040 | rs3935627 | 0.056 | -5.320 | 1.063 | 84 | 3 | enet | 0.039 | 6.40E-05 | -1.32E+00 | 1.87E-01 |
| DPP10 | 2 | 115199876 | 116603328 | 0.1648 | rs17732349 | 2.693 | rs3738879 | 0.146 | -7.660 | 1.330 | 577 | 22 | enet | 0.140 | 6.00E-14 | -1.31E+00 | 1.89E-01 |
| MBOAT7 | 19 | 54677107 | 54693733 | 0.0632 | rs3865475 | -2.430 | rs36655 | 0.019 | 3.750 | 1.826 | 93 | 8 | enet | 0.011 | 2.30E-02 | 1.31E+00 | 1.90E-01 |
| SCYL1 | 11 | 65292548 | 65306175 | 0.0432 | rs2004649 | 3.970 | rs1152620 | 0.030 | 3.890 | -1.171 | 63 | 4 | enet | 0.012 | 1.70E-02 | -1.31E+00 | 1.90E-01 |
| GLRX5 | 14 | 95999840 | 96011061 | 0.7840 | rs10139467 | 3.330 | rs10484053 | 0.647 | -15.640 | -1.556 | 219 | 15 | enet | 0.660 | 1.80E-90 | 1.31E+00 | 1.90E-01 |
| SAMM50 | 22 | 44351301 | 44406411 | 0.0758 | rs5764434 | -1.800 | rs5764034 | 0.109 | 6.520 | -1.173 | 197 | 8 | enet | 0.093 | 8.00E-10 | -1.31E+00 | 1.92E-01 |
| GLRX | 5 | 95087023 | 95158709 | 0.0967 | rs34902 | -2.893 | rs9314158 | 0.031 | -5.010 | -0.522 | 155 | 16 | enet | 0.071 | 8.40E-08 | 1.31E+00 | 1.92E-01 |
| DPP3 | 11 | 66247484 | 66277130 | 0.2952 | rs10791881 | 1.890 | rs12421620 | 0.085 | -6.000 | -1.582 | 75 | 3 | enet | 0.077 | 2.40E-08 | 1.31E+00 | 1.92E-01 |
| PRDX3 | 10 | 120927215 | 120938345 | 0.0603 | rs10886406 | -2.500 | rs2297696 | 0.041 | -4.180 | 1.195 | 79 | 11 | enet | 0.023 | 1.90E-03 | -1.30E+00 | 1.93E-01 |
| FER | 5 | 108083523 | 108532542 | 0.0884 | rs892009 | 2.868 | rs6594341 | 0.024 | 4.250 | -1.697 | 222 | 23 | enet | 0.020 | 6.10E-03 | -1.30E+00 | 1.93E-01 |
| SLC25A24 | 1 | 108676658 | 108743471 | 0.6169 | rs7538977 | 2.408 | rs533233 | 0.486 | -13.550 | -1.113 | 118 | 11 | enet | 0.560 | 1.30E-68 | 1.30E+00 | 1.94E-01 |
| THG1L | 5 | 157158205 | 157168456 | 0.2061 | rs2270820 | -2.303 | rs2270818 | 0.122 | 6.010 | -1.397 | 102 | 13 | enet | 0.120 | 1.50E-08 | -1.30E+00 | 1.94E-01 |
| SIK3 | 11 | 116714118 | 116969153 | 0.0506 | rs632153 | 4.090 | rs11216164 | 0.046 | -4.440 | -1.068 | 204 | 7 | enet | 0.005 | 8.30E-02 | 1.30E+00 | 1.94E-01 |
| PRKCB | 16 | 23847322 | 24231932 | 0.0656 | rs780169 | -3.188 | rs120908 | 0.047 | -5.450 | 0.914 | 296 | 11 | enet | 0.066 | 2.80E-07 | -1.30E+00 | 1.94E-01 |
| APRT | 16 | 88875747 | 88878352 | 0.2667 | rs17176204 | 2.357 | rs1834065 | 0.152 | -7.890 | 0.731 | 105 | 11 | enet | 0.150 | 4.50E-15 | -1.30E+00 | 1.94E-01 |
| CCBL1 | 9 | 131595221 | 131644773 | 0.0754 | rs10739734 | 2.000 | rs10760581 | 0.004 | -4.010 | -0.238 | 65 | 14 | enet | 0.026 | 9.60E-04 | 1.30E+00 | 1.94E-01 |
| UMPS | 3 | 124449213 | 124464040 | 0.2497 | rs13314004 | 3.490 | rs6768588 | 0.357 | 11.640 | 1.015 | 148 | 47 | enet | 0.400 | 2.20E-42 | 1.29E+00 | 1.96E-01 |

| | | | | | | | | | | | | | | | | | |
|---|---|---|---|---|---|---|---|---|---|---|---|---|---|---|---|---|---|
| COA6 | 1 | 234509202 | 234519795 | 0.1995 | rs17517734 | 2.229 | rs6586384 | 0.166 | 8.520 | 1.394 | 157 | 13 | enet | 0.190 | 3.20E-18 | 1.29E+00 | 1.96E-01 |
| TELO2 | 16 | 1543345 | 1560458 | 0.0758 | rs2072950 | 2.009 | rs932391 | 0.003 | -3.480 | 1.062 | 110 | 12 | enet | 0.028 | 1.50E-03 | -1.29E+00 | 1.96E-01 |
| SIRPA | 20 | 1875154 | 1920543 | 0.6800 | rs4470399 | -3.045 | rs6075340 | 0.718 | -16.450 | -1.215 | 162 | 7 | enet | 0.710 | 2.00E-104 | 1.29E+00 | 1.96E-01 |
| LIMS1 | 2 | 109150857 | 109303702 | 0.0899 | rs4676040 | 2.265 | rs10196891 | 0.005 | -3.540 | 0.395 | 74 | 8 | enet | 0.032 | 2.90E-04 | 1.29E+00 | 1.97E-01 |
| EPHX2 | 8 | 27348296 | 27403081 | 0.4388 | rs12334538 | -3.250 | rs2741342 | 0.245 | -10.380 | -1.630 | 190 | 36 | enet | 0.500 | 6.20E-58 | 1.29E+00 | 1.97E-01 |
| TNFAIP2 | 14 | 103589779 | 103603776 | 0.2362 | rs944002 | 2.620 | rs2282037 | 0.076 | 5.130 | 0.286 | 72 | 13 | enet | 0.150 | 8.40E-12 | 1.29E+00 | 1.97E-01 |
| FTO | 16 | 53737875 | 54155853 | 0.1312 | rs2542671 | 2.163 | rs2072518 | 0.043 | -4.560 | 1.182 | 342 | 33 | enet | 0.014 | 1.20E-02 | -1.29E+00 | 1.97E-01 |
| HMGCLL1 | 6 | 55299167 | 55444012 | 0.1347 | rs6918642 | -4.026 | rs1546299 | 0.030 | -4.920 | 0.687 | 175 | 15 | enet | 0.073 | 7.80E-08 | -1.29E+00 | 1.98E-01 |
| TBRG4 | 7 | 45139699 | 45151646 | 0.1804 | rs2331209 | 2.570 | rs2304693 | 0.266 | -10.150 | 0.953 | 141 | 20 | enet | 0.250 | 1.80E-25 | -1.28E+00 | 1.99E-01 |
| CORO7 | 16 | 4404543 | 4475706 | 0.1861 | rs2283479 | -3.080 | rs6500596 | 0.077 | -5.600 | -1.757 | 85 | 18 | enet | 0.062 | 5.50E-07 | 1.28E+00 | 2.00E-01 |
| CCDC58 | 3 | 122078438 | 122102078 | 0.2160 | rs12496410 | 2.360 | rs6438726 | 0.139 | -7.270 | 1.884 | 116 | 30 | enet | 0.110 | 1.40E-11 | -1.28E+00 | 2.00E-01 |
| GCAT | 22 | 38203912 | 38213183 | 0.5526 | rs2285179 | -2.320 | rs5756825 | 0.486 | 13.280 | -1.517 | 60 | 13 | enet | 0.530 | 5.20E-61 | -1.28E+00 | 2.01E-01 |
| TKT | 3 | 53258723 | 53290068 | 0.1776 | rs4687570 | -2.510 | rs3736151 | 0.204 | 8.800 | -1.064 | 93 | 14 | enet | 0.210 | 3.30E-21 | -1.28E+00 | 2.01E-01 |
| TRPM3 | 9 | 73143979 | 74061820 | 0.1433 | rs17460690 | 3.710 | rs11142707 | 0.025 | -5.210 | -1.125 | 608 | 26 | enet | 0.073 | 1.20E-07 | 1.28E+00 | 2.01E-01 |
| ACAD11 | 3 | 132276982 | 132379567 | 0.2610 | rs4017724 | -2.620 | rs16839507 | 0.108 | -6.390 | 1.354 | 87 | 3 | enet | 0.079 | 5.70E-08 | -1.28E+00 | 2.01E-01 |
| DCUN1D1 | 3 | 182655862 | 182703741 | 0.0368 | rs1547910 | 1.770 | rs1002767 | 0.035 | -4.370 | 1.073 | 67 | 12 | enet | 0.018 | 4.90E-03 | -1.28E+00 | 2.02E-01 |
| ACAA1 | 3 | 38144620 | 38178733 | 0.0880 | rs11929529 | 2.470 | rs7625290 | 0.068 | -5.510 | 0.983 | 65 | 12 | enet | 0.066 | 2.40E-07 | -1.28E+00 | 2.02E-01 |
| SENP8 | 15 | 72406599 | 72433311 | 0.1260 | rs2957369 | -1.620 | rs7182776 | 0.086 | -5.700 | 0.426 | 57 | 25 | enet | 0.076 | 4.10E-08 | -1.27E+00 | 2.03E-01 |
| KALRN | 3 | 123798870 | 124445172 | 0.0597 | rs13314004 | 3.490 | rs1532668 | 0.017 | 4.230 | -0.726 | 450 | 12 | enet | 0.026 | 9.20E-04 | -1.27E+00 | 2.03E-01 |
| RARRES2 | 7 | 150035408 | 150038763 | 0.0623 | rs1524335 | 1.560 | rs3735167 | 0.015 | 3.650 | 0.667 | 65 | 31 | enet | 0.017 | 2.10E-02 | 1.27E+00 | 2.03E-01 |
| MFI2 | 3 | 196715492 | 196756687 | 0.1654 | rs4916453 | -2.790 | rs2280294 | 0.121 | 6.900 | 0.245 | 113 | 15 | enet | 0.150 | 2.60E-15 | 1.27E+00 | 2.04E-01 |
| DIAPH1 | 5 | 140894583 | 140998622 | 0.0679 | rs1059029 | 3.626 | rs6892185 | 0.034 | 4.960 | 1.365 | 151 | 16 | enet | 0.048 | 9.50E-06 | 1.27E+00 | 2.05E-01 |
| NAPRT1 | 8 | 144656955 | 144660819 | 0.6373 | rs13257849 | 1.690 | rs1809148 | 0.542 | -14.520 | 0.670 | 81 | 10 | enet | 0.650 | 1.00E-86 | -1.26E+00 | 2.06E-01 |
| LGALS3 | 14 | 55590828 | 55612126 | 0.2375 | rs8009181 | 2.940 | rs17672376 | 0.129 | -8.110 | 0.930 | 64 | 6 | enet | 0.200 | 7.30E-20 | -1.26E+00 | 2.06E-01 |
| RAB5B | 12 | 56367697 | 56388490 | 0.0309 | rs9739196 | -2.050 | rs10876864 | 0.012 | -3.850 | -0.692 | 49 | 8 | enet | 0.002 | 1.90E-01 | 1.26E+00 | 2.07E-01 |
| ACADS | 12 | 121163538 | 121177811 | 0.7651 | rs3809310 | -1.900 | rs2014355 | 0.414 | -12.530 | 1.624 | 116 | 19 | enet | 0.570 | 1.30E-70 | -1.26E+00 | 2.07E-01 |
| OSGEP | 14 | 20914570 | 20923264 | 0.0850 | rs2228036 | 2.800 | rs1760944 | 0.042 | -5.060 | 1.011 | 175 | 14 | enet | 0.054 | 5.40E-06 | -1.26E+00 | 2.07E-01 |
| NRSN1 | 6 | 24126350 | 24155128 | 0.0668 | rs13216359 | 2.090 | rs9791210 | 0.006 | -3.300 | -0.352 | 133 | 20 | enet | 0.007 | 5.90E-02 | 1.26E+00 | 2.08E-01 |
| PKIB | 6 | 122793076 | 123047518 | 0.0983 | rs9388133 | 2.601 | rs9490531 | -0.003 | 3.390 | -1.204 | 202 | 19 | enet | 0.007 | 1.30E-01 | -1.25E+00 | 2.10E-01 |
| ABHD11 | 7 | 73150424 | 73153197 | 0.2923 | rs4717803 | -3.400 | rs6460052 | 0.266 | 10.110 | -1.112 | 73 | 7 | enet | 0.240 | 8.50E-25 | -1.25E+00 | 2.10E-01 |
| GPNMB | 7 | 23275586 | 23314727 | 0.1904 | rs17740440 | 2.500 | rs858239 | 0.218 | -9.270 | 1.311 | 92 | 16 | enet | 0.220 | 6.40E-21 | -1.25E+00 | 2.12E-01 |
| FNDC3A | 13 | 49550048 | 49783915 | 0.0742 | rs623135 | -2.910 | rs9535179 | 0.031 | -4.350 | -0.989 | 151 | 6 | enet | 0.024 | 1.70E-03 | 1.25E+00 | 2.13E-01 |
| ETNPPL | 4 | 109663196 | 109684210 | 0.0473 | rs6837232 | 2.170 | rs10049520 | 0.013 | 4.320 | 1.269 | 94 | 9 | enet | 0.032 | 3.70E-04 | 1.25E+00 | 2.13E-01 |
| TMEM120A | 7 | 75616155 | 75623977 | 0.1010 | rs28737229 | 2.690 | rs7794040 | 0.078 | -6.470 | 1.643 | 73 | 10 | enet | 0.082 | 8.50E-09 | -1.25E+00 | 2.13E-01 |
| ALDH18A1 | 10 | 97365696 | 97416463 | 0.3445 | rs11188369 | 3.400 | rs2275272 | 0.227 | -9.470 | -1.259 | 157 | 29 | enet | 0.290 | 1.90E-29 | 1.24E+00 | 2.15E-01 |
| ADD1 | 4 | 2845584 | 2931803 | 0.2134 | rs12504990 | 3.310 | rs2285084 | 0.296 | -11.030 | -1.386 | 119 | 15 | enet | 0.310 | 2.20E-32 | 1.24E+00 | 2.15E-01 |
| AIFM3 | 22 | 21319396 | 21335649 | 0.0880 | rs2541953 | -2.480 | rs178280 | 0.032 | -4.240 | 0.872 | 135 | 20 | enet | 0.024 | 1.60E-03 | -1.24E+00 | 2.16E-01 |
| MMS19 | 10 | 99218081 | 99258551 | 0.0762 | rs3814553 | 4.130 | rs3740526 | 0.046 | -4.840 | 0.562 | 106 | 7 | enet | 0.045 | 2.10E-05 | -1.24E+00 | 2.16E-01 |
| WDR41 | 5 | 76721795 | 76916436 | 0.0627 | rs2241367 | 2.266 | rs4273585 | 0.061 | -6.080 | 1.061 | 224 | 9 | enet | 0.069 | 1.50E-07 | -1.24E+00 | 2.16E-01 |
| SIRT3 | 11 | 215458 | 236931 | 0.0794 | rs7944394 | 5.440 | rs1045288 | 0.003 | -4.270 | -0.387 | 106 | 12 | enet | 0.052 | 4.50E-06 | -1.24E+00 | 2.16E-01 |
| MICU1 | 10 | 74127098 | 74385899 | 0.0615 | rs7088870 | 2.100 | rs3009568 | 0.001 | -3.650 | 1.988 | 139 | 13 | enet | -0.003 | 9.20E-01 | -1.23E+00 | 2.18E-01 |
| HSP90B1 | 12 | 104323885 | 104347423 | 0.1436 | rs2583237 | -3.360 | rs1165679 | -0.001 | 3.910 | 0.585 | 170 | 40 | enet | 0.046 | 1.60E-05 | 1.23E+00 | 2.19E-01 |
| PDK2 | 17 | 48172101 | 48189516 | 0.1227 | rs1107439 | -3.650 | rs11868719 | 0.013 | 4.650 | 1.048 | 111 | 9 | enet | 0.079 | 1.80E-08 | 1.23E+00 | 2.19E-01 |
| EGLN1 | 1 | 231499497 | 231560790 | 0.0172 | rs2250704 | 1.901 | rs6541266 | 0.019 | -3.300 | 1.668 | 92 | 14 | enet | 0.019 | 4.00E-03 | -1.23E+00 | 2.20E-01 |
| TMEM143 | 19 | 48835613 | 48867494 | 0.1116 | rs6509368 | 1.580 | rs4801754 | -0.003 | -2.870 | -1.402 | 112 | 17 | enet | 0.011 | 2.40E-02 | 1.22E+00 | 2.21E-01 |
| STK39 | 2 | 168810530 | 169104651 | 0.0425 | rs1400641 | -3.101 | rs2138753 | -0.003 | -4.180 | -1.002 | 243 | 9 | enet | -0.003 | 9.30E-01 | 1.22E+00 | 2.21E-01 |

| | | | | | | | | | | | | | | | | |
|---|---|---|---|---|---|---|---|---|---|---|---|---|---|---|---|---|
| GSTP1 | 11 | 67351066 | 67354131 | 0.1225 | rs10896176 | -1.940 | rs7941648 | 0.222 | -9.580 | -1.405 | 76 | 10 | enet | 0.220 | 1.80E-22 | 1.22E+00 | 2.21E-01 |
| SURF1 | 9 | 136218610 | 136223552 | 0.5011 | rs630014 | 2.890 | rs3124768 | 0.210 | -9.600 | -1.259 | 143 | 47 | enet | 0.400 | 1.70E-43 | 1.22E+00 | 2.22E-01 |
| UHRF1BP1L | 12 | 100422233 | 100536626 | 0.1071 | rs12825841 | 3.200 | rs7962975 | -0.001 | 2.710 | -0.497 | 108 | 24 | enet | 0.009 | 3.70E-02 | -1.22E+00 | 2.22E-01 |
| GABRB1 | 4 | 46995740 | 47428461 | 0.0903 | rs7439087 | -2.630 | rs6289 | 0.032 | -4.740 | -0.040 | 260 | 9 | enet | 0.054 | 3.10E-06 | -1.22E+00 | 2.23E-01 |
| C9orf64 | 9 | 86553226 | 86571901 | 0.2515 | rs4535826 | 4.160 | rs1572030 | 0.196 | -8.810 | -1.640 | 55 | 6 | enet | 0.200 | 2.50E-20 | 1.21E+00 | 2.25E-01 |
| NANS | 9 | 100819021 | 100845357 | 0.3319 | rs7855251 | -5.480 | rs16912590 | 0.344 | -11.410 | 0.723 | 109 | 8 | enet | 0.350 | 4.40E-37 | -1.21E+00 | 2.25E-01 |
| DAGLB | 7 | 6448757 | 6523821 | 0.1608 | rs11980237 | 2.460 | rs836534 | 0.055 | -6.200 | -0.396 | 105 | 23 | enet | 0.098 | 3.00E-10 | 1.21E+00 | 2.27E-01 |
| KCNAB2 | 1 | 6051526 | 6161253 | 0.0323 | rs17436816 | -1.702 | rs12139627 | 0.039 | -4.520 | -1.212 | 143 | 3 | enet | 0.010 | 2.70E-02 | 1.21E+00 | 2.28E-01 |
| UBE2F | 2 | 238875469 | 238951236 | 0.1468 | rs6707140 | 3.053 | rs7578855 | 0.006 | -4.370 | 0.111 | 120 | 14 | enet | 0.014 | 1.40E-02 | -1.20E+00 | 2.29E-01 |
| SAMHD1 | 20 | 35518632 | 35580246 | 0.0996 | rs2425306 | 2.143 | rs6029941 | 0.019 | 3.930 | -0.387 | 61 | 15 | enet | 0.038 | 8.20E-05 | -1.20E+00 | 2.29E-01 |
| PLEKHA6 | 1 | 204187979 | 204346793 | 0.0966 | rs4951319 | 2.545 | rs4951056 | 0.002 | -4.080 | 1.910 | 249 | 8 | enet | 0.040 | 5.20E-05 | -1.20E+00 | 2.30E-01 |
| TNC | 9 | 117782806 | 117880536 | 0.0499 | rs2297181 | 3.400 | rs10817715 | 0.038 | -4.270 | -1.137 | 231 | 14 | enet | 0.018 | 4.90E-03 | 1.20E+00 | 2.31E-01 |
| SNX5 | 20 | 17922241 | 17949623 | 0.0534 | rs2618600 | 2.510 | rs6080918 | 0.007 | -3.800 | 1.473 | 131 | 9 | enet | 0.011 | 2.50E-02 | -1.20E+00 | 2.31E-01 |
| CRTAC1 | 10 | 99624757 | 99790585 | 0.1526 | rs1925556 | 3.040 | rs7922066 | 0.054 | -6.020 | -0.026 | 169 | 16 | enet | 0.096 | 5.00E-10 | -1.20E+00 | 2.32E-01 |
| MTERFD2 | 2 | 242011584 | 242041747 | 0.0945 | rs4497870 | 1.398 | rs2108485 | 0.125 | -6.420 | 1.110 | 119 | 7 | enet | 0.130 | 8.00E-10 | -1.19E+00 | 2.32E-01 |
| GGH | 8 | 63927638 | 63951730 | 0.1000 | rs4314646 | 1.760 | rs1031551 | 0.031 | 5.600 | -0.654 | 113 | 14 | enet | 0.072 | 8.00E-08 | -1.20E+00 | 2.32E-01 |
| MTG2 | 20 | 60758085 | 60778624 | 0.1442 | rs17812169 | 2.908 | rs6061457 | -0.001 | -4.140 | -0.161 | 104 | 5 | enet | 0.025 | 1.40E-02 | -1.19E+00 | 2.32E-01 |
| RIPK2 | 8 | 90769975 | 90803291 | 0.0529 | rs7841459 | 2.060 | rs39502 | 0.049 | -5.260 | 1.652 | 87 | 8 | enet | 0.036 | 1.70E-04 | -1.19E+00 | 2.33E-01 |
| NME1 | 17 | 49230897 | 49239789 | 0.4020 | rs17574235 | -3.270 | rs16949649 | 0.331 | 11.240 | -1.334 | 67 | 17 | enet | 0.330 | 7.80E-35 | -1.19E+00 | 2.33E-01 |
| TSPAN14 | 10 | 82213922 | 82292879 | 0.0475 | rs10785965 | 2.530 | rs7096348 | 0.018 | -3.930 | -0.388 | 172 | 19 | enet | 0.001 | 2.70E-01 | 1.19E+00 | 2.33E-01 |
| MAPK10 | 4 | 86936276 | 87515284 | 0.0583 | rs10029162 | -4.240 | rs2282598 | 0.031 | 4.360 | 0.491 | 253 | 16 | enet | 0.045 | 2.00E-05 | -1.19E+00 | 2.34E-01 |
| FBLN7 | 2 | 112895962 | 112945791 | 0.0979 | rs6713344 | -5.055 | rs13001162 | 0.033 | -4.760 | 1.142 | 104 | 19 | enet | 0.086 | 2.60E-08 | -1.19E+00 | 2.36E-01 |
| TFG | 3 | 100428205 | 100467810 | 0.0744 | rs560803 | 1.870 | rs4928081 | 0.123 | -7.330 | 1.074 | 102 | 10 | enet | 0.110 | 1.20E-11 | -1.18E+00 | 2.36E-01 |
| TMEM106B | 7 | 12250867 | 12282993 | 0.2378 | rs17165702 | 3.130 | rs5011432 | 0.277 | -10.260 | 1.253 | 139 | 19 | enet | 0.270 | 3.70E-28 | -1.18E+00 | 2.37E-01 |
| NOP9 | 14 | 24769068 | 24778330 | 0.0629 | rs11846223 | -2.880 | rs12895335 | 0.038 | -4.290 | 1.248 | 120 | 8 | enet | 0.030 | 2.50E-03 | -1.18E+00 | 2.37E-01 |
| LYRM5 | 12 | 25348150 | 25362579 | 0.0723 | rs11047889 | -1.870 | rs12813551 | 0.083 | 5.190 | 1.088 | 104 | 11 | enet | 0.047 | 3.00E-04 | 1.18E+00 | 2.38E-01 |
| CYB5R3 | 22 | 43013846 | 43045574 | 0.1699 | rs2071846 | 2.300 | rs137087 | 0.182 | -8.790 | 0.880 | 99 | 16 | enet | 0.230 | 8.10E-24 | -1.18E+00 | 2.38E-01 |
| EPB41L2 | 6 | 131160487 | 131384462 | 0.0702 | rs6933145 | -2.253 | rs7766505 | 0.013 | 3.850 | -0.315 | 213 | 25 | enet | 0.016 | 7.20E-03 | -1.18E+00 | 2.39E-01 |
| DDT | 22 | 24313554 | 24322660 | 0.6169 | rs12628766 | -1.380 | rs4822466 | 0.421 | -11.490 | 1.072 | 73 | 26 | enet | 0.620 | 1.20E-66 | -1.18E+00 | 2.40E-01 |
| GAN | 16 | 81348557 | 81424489 | 0.0741 | rs17700694 | 3.200 | rs2290948 | 0.002 | -4.140 | -0.046 | 190 | 18 | enet | 0.009 | 3.80E-02 | 1.18E+00 | 2.40E-01 |
| ZFYVE1 | 14 | 73436159 | 73493920 | 0.0329 | rs7161198 | 1.920 | rs1007934 | 0.032 | 4.190 | -1.229 | 111 | 4 | enet | 0.010 | 3.00E-02 | -1.17E+00 | 2.41E-01 |
| SLC25A29 | 14 | 100757448 | 100772884 | 0.0398 | rs4900463 | -4.060 | rs1059263 | 0.025 | 3.820 | 0.804 | 76 | 4 | enet | 0.006 | 8.70E-02 | 1.17E+00 | 2.41E-01 |
| PUSL1 | 1 | 1243947 | 1247057 | 0.1651 | rs11721 | 3.552 | rs1240708 | 0.006 | -3.950 | 1.928 | 56 | 5 | enet | 0.022 | 9.70E-03 | -1.17E+00 | 2.43E-01 |
| NSF | 17 | 44668035 | 44834830 | 0.1975 | rs1373089 | -3.490 | rs4968239 | 0.011 | 4.810 | -0.635 | 54 | 12 | enet | 0.099 | 2.30E-10 | -1.17E+00 | 2.43E-01 |
| SCRN2 | 17 | 45915058 | 45918699 | 0.3213 | rs16947058 | 4.250 | rs2325752 | 0.116 | 7.170 | -1.263 | 95 | 28 | enet | 0.170 | 2.10E-17 | -1.17E+00 | 2.43E-01 |
| YES1 | 18 | 721588 | 812547 | 0.0693 | rs11877057 | -1.990 | rs7240865 | 0.015 | -4.000 | 0.808 | 181 | 15 | enet | 0.025 | 1.20E-03 | -1.17E+00 | 2.43E-01 |
| NCAM2 | 21 | 22370633 | 22915650 | 0.0871 | rs2826621 | 2.110 | rs233803 | 0.000 | 3.330 | -0.589 | 366 | 33 | enet | -0.001 | 4.20E-01 | -1.17E+00 | 2.44E-01 |
| HEBP1 | 12 | 13127798 | 13153207 | 0.6877 | rs850938 | 2.760 | rs4763913 | 0.536 | -14.620 | -0.983 | 118 | 37 | enet | 0.540 | 2.40E-65 | 1.17E+00 | 2.44E-01 |
| AP3B2 | 15 | 83328033 | 83378666 | 0.0492 | rs3914699 | 1.450 | rs7163848 | 0.012 | 3.560 | 1.273 | 87 | 11 | enet | 0.033 | 2.10E-04 | 1.16E+00 | 2.44E-01 |
| XRCC1 | 19 | 44047192 | 44084625 | 0.0714 | rs11083711 | -2.810 | rs25489 | 0.008 | -4.000 | 0.654 | 128 | 11 | enet | -0.001 | 3.60E-01 | -1.16E+00 | 2.46E-01 |
| OSCP1 | 1 | 36881428 | 36916086 | 0.1584 | rs6690378 | 2.743 | rs12070076 | 0.161 | -8.530 | -1.361 | 113 | 18 | enet | 0.180 | 1.20E-17 | 1.16E+00 | 2.46E-01 |
| WWOX | 16 | 78133310 | 79246564 | 0.4247 | rs6564639 | -5.033 | rs7184686 | 0.184 | 8.390 | 1.956 | 1240 | 39 | enet | 0.300 | 9.10E-31 | 1.16E+00 | 2.46E-01 |
| ALG1 | 16 | 5083703 | 5137380 | 0.3285 | rs759191 | 2.465 | rs7190704 | 0.022 | -4.390 | -0.845 | 98 | 29 | enet | 0.098 | 4.20E-10 | 1.16E+00 | 2.47E-01 |
| CCDC136 | 7 | 128430811 | 128462186 | 0.0420 | rs17164371 | 1.570 | rs1006023 | 0.033 | -3.890 | 0.852 | 111 | 13 | enet | 0.008 | 4.20E-02 | -1.15E+00 | 2.51E-01 |
| RCHY1 | 4 | 76404247 | 76439974 | 0.0696 | rs1466441 | -2.750 | rs6535429 | 0.065 | 5.400 | -0.229 | 86 | 15 | enet | 0.070 | 1.70E-07 | -1.15E+00 | 2.51E-01 |
| DDX58 | 9 | 32455300 | 32526322 | 0.1514 | rs7042042 | 2.520 | rs7042042 | 0.018 | -3.560 | 2.518 | 128 | 14 | enet | 0.022 | 2.30E-03 | -1.15E+00 | 2.51E-01 |

| | | | | | | | | | | | | | | | | | |
|---|---|---|---|---|---|---|---|---|---|---|---|---|---|---|---|---|---|
| NDUFB9 | 8 | 125551344 | 125580751 | 0.1012 | rs16899928 | -1.300 | rs4527852 | 0.126 | -7.000 | -1.187 | 131 | 7 | enet | 0.095 | 6.30E-10 | 1.15E+00 | 2.51E-01 |
| SARM1 | 17 | 26691378 | 26728065 | 0.3080 | rs4795430 | -2.390 | rs739439 | 0.255 | 9.900 | 1.101 | 82 | 3 | enet | 0.250 | 2.50E-25 | 1.15E+00 | 2.51E-01 |
| TMEM230 | 20 | 5080486 | 5093749 | 0.0492 | rs13042903 | 2.093 | rs6053111 | 0.034 | -4.450 | 0.898 | 78 | 10 | enet | 0.019 | 4.70E-03 | -1.15E+00 | 2.51E-01 |
| CEP41 | 7 | 130033612 | 130082274 | 0.0889 | rs10253631 | 1.770 | rs1990790 | 0.124 | 6.920 | -1.113 | 129 | 3 | enet | 0.110 | 9.10E-12 | -1.15E+00 | 2.51E-01 |
| UROS | 10 | 127477146 | 127511817 | 0.1281 | rs12354855 | -1.980 | rs10794025 | 0.286 | 10.910 | 1.140 | 67 | 6 | enet | 0.280 | 3.30E-29 | 1.15E+00 | 2.52E-01 |
| EFHD2 | 1 | 15736391 | 15756839 | 0.1250 | rs16851662 | -2.395 | rs742361 | 0.024 | -4.500 | 1.273 | 99 | 9 | enet | 0.010 | 2.60E-02 | -1.15E+00 | 2.52E-01 |
| ISPD | 7 | 16130817 | 16460947 | 0.1702 | rs12669858 | -2.860 | rs7783810 | 0.035 | -4.850 | -1.245 | 273 | 34 | enet | 0.082 | 9.50E-09 | 1.14E+00 | 2.53E-01 |
| BAIAP3 | 16 | 1383602 | 1399439 | 0.2771 | rs3760122 | -1.843 | rs4984814 | 0.140 | 7.810 | -0.807 | 71 | 10 | enet | 0.170 | 9.90E-17 | -1.14E+00 | 2.55E-01 |
| ACADVL | 17 | 7120444 | 7128592 | 0.0707 | rs1215 | 2.770 | rs446994 | -0.002 | 3.400 | -1.187 | 77 | 19 | enet | -0.003 | 9.90E-01 | -1.14E+00 | 2.55E-01 |
| GMPS | 3 | 155588325 | 155661815 | 0.0555 | rs12487278 | 1.850 | rs11706359 | 0.028 | 4.160 | 1.105 | 90 | 13 | enet | 0.005 | 8.30E-02 | 1.14E+00 | 2.55E-01 |
| N6AMT2 | 13 | 21302870 | 21348097 | 0.2399 | rs9509372 | 3.070 | rs17318109 | 0.163 | -7.440 | -0.777 | 88 | 21 | enet | 0.150 | 6.50E-13 | 1.14E+00 | 2.56E-01 |
| IFT27 | 22 | 37154246 | 37172300 | 0.1451 | rs760517 | -4.510 | rs2009667 | 0.103 | 6.910 | 0.557 | 131 | 3 | enet | 0.130 | 2.20E-13 | 1.14E+00 | 2.56E-01 |
| INPP5D | 2 | 233924677 | 234116549 | 0.0586 | rs6720896 | 3.631 | rs13013293 | 0.013 | 3.880 | -1.326 | 192 | 7 | enet | -0.001 | 3.80E-01 | -1.13E+00 | 2.56E-01 |
| RTN3 | 11 | 63448918 | 63527363 | 0.0415 | rs7928514 | 2.070 | rs2509744 | -0.002 | -3.240 | 1.853 | 59 | 9 | enet | 0.003 | 1.60E-01 | -1.14E+00 | 2.56E-01 |
| LCMT1 | 16 | 25123050 | 25189552 | 0.0958 | rs12931780 | -2.661 | rs4238912 | 0.063 | -5.600 | 0.374 | 108 | 10 | enet | 0.081 | 1.20E-08 | -1.14E+00 | 2.56E-01 |
| APOH | 17 | 64208151 | 64252643 | 0.3899 | rs11869695 | -2.630 | rs1801689 | 0.140 | -7.400 | 0.153 | 109 | 11 | enet | 0.140 | 2.50E-14 | -1.14E+00 | 2.56E-01 |
| C17orf62 | 17 | 80400465 | 80408705 | 0.0479 | rs9894993 | 2.800 | rs9909476 | 0.020 | -3.700 | 1.590 | 83 | 8 | enet | 0.018 | 5.90E-03 | -1.14E+00 | 2.56E-01 |
| RWDD2B | 21 | 30376705 | 30391699 | 0.3290 | rs2297252 | 2.690 | rs2150403 | 0.388 | 8.910 | -1.210 | 73 | 9 | enet | 0.380 | 2.50E-22 | -1.13E+00 | 2.58E-01 |
| CACNA2D3 | 3 | 54156574 | 55108584 | 0.1330 | rs13062287 | 3.630 | rs750379 | 0.051 | 4.580 | -0.196 | 690 | 25 | enet | 0.022 | 2.10E-03 | -1.13E+00 | 2.59E-01 |
| BDH1 | 3 | 197236654 | 197300194 | 0.5851 | rs7649784 | 2.800 | rs8034 | 0.239 | 9.620 | -2.056 | 100 | 31 | enet | 0.340 | 1.10E-35 | -1.13E+00 | 2.59E-01 |
| ETFDH | 4 | 159593277 | 159630775 | 0.0836 | rs6815665 | -2.480 | rs11559290 | 0.040 | 4.230 | 0.014 | 30 | 25 | enet | 0.050 | 6.60E-06 | 1.13E+00 | 2.59E-01 |
| RAB7L1 | 1 | 205737114 | 205744588 | 0.1329 | rs4245718 | 2.579 | rs823114 | 0.072 | -5.360 | 1.665 | 92 | 18 | enet | 0.053 | 8.90E-06 | -1.13E+00 | 2.59E-01 |
| BTN3A3 | 6 | 26440700 | 26453643 | 0.2846 | rs13194984 | 4.790 | rs10946813 | 0.089 | 5.950 | 1.410 | 182 | 19 | enet | 0.150 | 1.00E-14 | 1.13E+00 | 2.60E-01 |
| TRIM4 | 7 | 99474581 | 99517223 | 0.1790 | rs6960542 | 3.530 | rs472660 | 0.013 | -4.290 | 0.950 | 81 | 23 | enet | 0.078 | 7.90E-07 | -1.13E+00 | 2.60E-01 |
| SGSH | 17 | 78180515 | 78194722 | 0.0602 | rs8068939 | 3.340 | rs7213900 | -0.003 | -3.280 | -1.418 | 116 | 17 | enet | 0.001 | 2.40E-01 | 1.12E+00 | 2.61E-01 |
| KCTD8 | 4 | 44175926 | 44450824 | 0.0642 | rs10009336 | 2.260 | rs11939023 | 0.007 | 3.920 | -1.253 | 136 | 2 | enet | 0.001 | 2.50E-01 | -1.12E+00 | 2.63E-01 |
| PLEKHG3 | 14 | 65170820 | 65213610 | 0.0873 | rs229621 | 2.590 | rs229677 | -0.001 | -2.940 | 1.947 | 156 | 23 | enet | 0.001 | 2.70E-01 | -1.12E+00 | 2.63E-01 |
| PENK | 8 | 57349233 | 57359293 | 0.0836 | rs6474069 | -1.760 | rs7002482 | 0.002 | -4.080 | 0.680 | 87 | 21 | enet | 0.042 | 2.10E-04 | -1.12E+00 | 2.64E-01 |
| FAM221A | 7 | 23719749 | 23742868 | 0.1606 | rs11762463 | -3.770 | rs6461725 | 0.133 | -7.340 | -0.073 | 92 | 15 | enet | 0.170 | 7.40E-16 | 1.12E+00 | 2.65E-01 |
| LIAS | 4 | 39460620 | 39479273 | 0.0955 | rs17510603 | 2.260 | rs17431867 | 0.058 | -4.660 | -1.934 | 96 | 16 | enet | 0.037 | 2.30E-04 | 1.11E+00 | 2.66E-01 |
| GRAMD3 | 5 | 125695824 | 125832186 | 0.0699 | rs7728158 | 2.647 | rs1156854 | 0.011 | -4.030 | -1.329 | 189 | 24 | enet | 0.030 | 4.80E-04 | 1.11E+00 | 2.66E-01 |
| SERGEF | 11 | 17809595 | 18034709 | 0.2485 | rs549485 | 2.110 | rs12292915 | 0.098 | 6.780 | 0.677 | 178 | 20 | enet | 0.210 | 1.70E-20 | 1.11E+00 | 2.66E-01 |
| ABCA5 | 17 | 67240452 | 67323385 | 0.2452 | rs10491178 | 3.390 | rs1550828 | 0.244 | 9.670 | 1.498 | 104 | 23 | enet | 0.310 | 9.00E-32 | 1.11E+00 | 2.66E-01 |
| COX20 | 1 | 244998624 | 245008359 | 0.0326 | rs2134271 | 1.819 | rs12146095 | 0.037 | -4.690 | 0.965 | 57 | 9 | enet | 0.026 | 1.00E-03 | -1.11E+00 | 2.67E-01 |
| CAPG | 2 | 85621871 | 85645555 | 0.3746 | rs2044475 | 2.448 | rs3770102 | 0.116 | 7.200 | 0.046 | 92 | 55 | enet | 0.230 | 8.60E-24 | -1.11E+00 | 2.67E-01 |
| PPIL3 | 2 | 201735630 | 201754026 | 0.6664 | rs11685853 | 2.787 | rs11892119 | 0.619 | -15.410 | -1.691 | 61 | 11 | enet | 0.730 | 1.40E-107 | 1.11E+00 | 2.67E-01 |
| RPA2 | 1 | 28218035 | 28241257 | 0.0902 | rs11399 | -1.934 | rs17257252 | 0.095 | 6.230 | 1.274 | 69 | 4 | enet | 0.087 | 3.50E-09 | 1.11E+00 | 2.68E-01 |
| SCN4B | 11 | 118004092 | 118023603 | 0.0331 | rs679327 | -3.730 | rs1715444 | 0.007 | -3.790 | 1.021 | 169 | 10 | enet | 0.010 | 2.80E-02 | -1.10E+00 | 2.70E-01 |
| ACSF2 | 17 | 48503519 | 48552206 | 0.1024 | rs4389179 | -1.620 | rs736911 | 0.069 | -5.310 | 1.499 | 113 | 11 | enet | 0.072 | 7.30E-08 | -1.10E+00 | 2.70E-01 |
| BHMT | 5 | 78407602 | 78428108 | 0.5924 | rs17823744 | -2.222 | rs7700970 | 0.310 | -10.470 | -0.204 | 82 | 17 | enet | 0.340 | 1.20E-32 | 1.10E+00 | 2.72E-01 |
| TBCB | 19 | 36605191 | 36616849 | 0.1055 | rs17884776 | 2.650 | rs2301736 | 0.042 | 4.430 | 0.297 | 79 | 14 | enet | 0.040 | 4.90E-05 | 1.10E+00 | 2.73E-01 |
| FABP3 | 1 | 31838472 | 31849697 | 0.0636 | rs7532845 | 1.608 | rs17495262 | 0.056 | -5.580 | -0.926 | 92 | 9 | enet | 0.053 | 3.60E-06 | 1.10E+00 | 2.73E-01 |
| NDUFAF2 | 5 | 60240956 | 60448853 | 0.1954 | rs4647128 | -4.214 | rs11951606 | 0.092 | 6.300 | -1.067 | 107 | 24 | enet | 0.160 | 1.10E-15 | 1.10E+00 | 2.73E-01 |
| VASN | 16 | 4421849 | 4433529 | 0.0450 | rs3888627 | -2.614 | rs11646063 | 0.011 | -4.090 | -1.490 | 65 | 11 | enet | 0.028 | 7.80E-04 | 1.10E+00 | 2.73E-01 |
| WDR61 | 15 | 78570177 | 78592136 | 0.0427 | rs16969711 | -2.620 | rs7183765 | 0.041 | 4.320 | 0.894 | 126 | 10 | enet | 0.028 | 6.50E-04 | 1.09E+00 | 2.74E-01 |
| RCSD1 | 1 | 167599330 | 167675486 | 0.2248 | rs10918754 | 2.389 | rs926521 | 0.127 | -6.990 | -1.794 | 198 | 28 | enet | 0.130 | 3.80E-13 | 1.09E+00 | 2.74E-01 |

| | | | | | | | | | | | | | | | | | |
|---|---|---|---|---|---|---|---|---|---|---|---|---|---|---|---|---|---|
| ATAD1 | 10 | 89511269 | 89601100 | 0.0801 | rs1234214 | 2.050 | rs12781556 | 0.002 | -3.070 | -0.466 | 109 | 31 | enet | 0.019 | 3.90E-03 | 1.09E+00 | 2.74E-01 |
| PGD | 1 | 10458649 | 10480201 | 0.1431 | rs11587326 | -2.699 | rs11121557 | 0.030 | 4.970 | 0.139 | 99 | 19 | enet | 0.063 | 5.20E-07 | 1.09E+00 | 2.75E-01 |
| RAB37 | 17 | 72666717 | 72743474 | 0.2570 | rs512038 | -2.140 | rs2280177 | 0.167 | -7.920 | 0.920 | 144 | 19 | enet | 0.150 | 7.30E-15 | -1.09E+00 | 2.75E-01 |
| CYBRD1 | 2 | 172378757 | 172414643 | 0.3245 | rs3806562 | -4.052 | rs2542941 | 0.328 | 11.180 | 1.340 | 113 | 18 | enet | 0.370 | 2.30E-40 | 1.09E+00 | 2.76E-01 |
| SLC1A4 | 2 | 65215611 | 65250999 | 0.1004 | rs6712664 | -3.093 | rs6754629 | 0.074 | 5.580 | -0.252 | 102 | 15 | enet | 0.055 | 3.00E-06 | -1.09E+00 | 2.77E-01 |
| KSR1 | 17 | 25783670 | 25953461 | 0.0713 | rs4794888 | -3.150 | rs940015 | -0.003 | -3.200 | -0.122 | 132 | 8 | enet | 0.006 | 7.00E-02 | 1.09E+00 | 2.77E-01 |
| RINT1 | 7 | 105172532 | 105208124 | 0.0345 | rs7799245 | 2.770 | rs711433 | 0.049 | -4.680 | 0.982 | 88 | 4 | enet | 0.018 | 5.70E-03 | -1.09E+00 | 2.77E-01 |
| C15orf38 | 15 | 90443159 | 90456188 | 0.0687 | rs10852122 | -2.550 | rs10520684 | 0.080 | -5.780 | 1.063 | 63 | 5 | enet | 0.069 | 1.50E-07 | -1.08E+00 | 2.81E-01 |
| PLEKHA2 | 8 | 38758753 | 38831428 | 0.3756 | rs7836041 | -2.830 | rs17513905 | 0.217 | 9.480 | 1.949 | 94 | 33 | enet | 0.340 | 1.60E-36 | 1.07E+00 | 2.83E-01 |
| UGDH | 4 | 39500375 | 39529931 | 0.0515 | rs17510603 | 2.260 | rs4453941 | 0.029 | -4.340 | -1.516 | 101 | 6 | enet | 0.023 | 3.60E-03 | 1.07E+00 | 2.84E-01 |
| CDH6 | 5 | 31193857 | 31329253 | 0.1774 | rs13172555 | 3.362 | rs7723322 | 0.083 | -5.880 | -1.315 | 234 | 12 | enet | 0.085 | 6.60E-09 | 1.07E+00 | 2.85E-01 |
| NAAA | 4 | 76831809 | 76862204 | 0.4383 | rs6532111 | -2.740 | rs10518142 | 0.238 | -7.780 | 1.671 | 118 | 27 | enet | 0.300 | 1.60E-21 | -1.07E+00 | 2.87E-01 |
| ACO1 | 9 | 32384618 | 32454767 | 0.1710 | rs7042042 | 2.520 | rs1133071 | 0.110 | 6.860 | -0.772 | 137 | 7 | enet | 0.099 | 2.50E-10 | -1.07E+00 | 2.87E-01 |
| LRP1B | 2 | 140988992 | 142889270 | 0.1976 | rs2177770 | 3.186 | rs11680286 | 0.047 | 4.860 | 1.487 | 1109 | 85 | enet | 0.022 | 2.60E-03 | 1.07E+00 | 2.87E-01 |
| PPP1R16A | 8 | 145703352 | 145727504 | 0.0330 | rs6989368 | 1.150 | rs6989368 | 0.024 | -4.120 | 1.146 | 58 | 6 | enet | 0.016 | 8.40E-03 | -1.06E+00 | 2.88E-01 |
| TOM1L2 | 17 | 17746828 | 17875736 | 0.1132 | rs17731766 | 2.040 | rs12941039 | 0.170 | -8.280 | 1.106 | 117 | 20 | enet | 0.160 | 1.70E-16 | -1.06E+00 | 2.88E-01 |
| CHMP1A | 16 | 89710839 | 89724253 | 0.0568 | rs154659 | -4.040 | rs164749 | 0.038 | -4.940 | -0.791 | 87 | 8 | enet | 0.024 | 1.60E-03 | 1.06E+00 | 2.89E-01 |
| ISLR2 | 15 | 74392652 | 74430881 | 0.1104 | rs17326524 | -1.370 | rs3888004 | 0.010 | -4.440 | 0.305 | 75 | 25 | enet | 0.053 | 5.80E-06 | -1.06E+00 | 2.90E-01 |
| BOLA1 | 1 | 149859440 | 149872351 | 0.2211 | rs1349532 | -1.204 | rs15931 | 0.397 | 12.370 | -1.194 | 24 | 10 | enet | 0.400 | 4.50E-43 | -1.06E+00 | 2.91E-01 |
| MRPL10 | 17 | 45900638 | 45908900 | 0.2458 | rs9910408 | 4.580 | rs3209 | 0.324 | -11.210 | -0.477 | 101 | 15 | enet | 0.330 | 5.50E-34 | 1.06E+00 | 2.91E-01 |
| GBA2 | 9 | 35736863 | 35749983 | 0.2790 | rs13284995 | -3.600 | rs1570249 | 0.333 | -11.310 | -0.899 | 104 | 11 | enet | 0.320 | 1.20E-33 | 1.06E+00 | 2.91E-01 |
| HSPA13 | 21 | 15743436 | 15755805 | 0.0560 | rs2159938 | 2.410 | rs2822648 | 0.007 | -4.050 | 1.072 | 155 | 12 | enet | 0.040 | 5.00E-05 | -1.05E+00 | 2.93E-01 |
| PDPR | 16 | 70147529 | 70195203 | 0.1323 | rs11075753 | -1.053 | rs4985520 | 0.034 | 4.030 | -0.618 | 15 | 8 | enet | 0.034 | 1.80E-04 | -1.05E+00 | 2.93E-01 |
| DGKQ | 4 | 952675 | 980683 | 0.1611 | rs11946340 | 3.840 | rs4583705 | 0.111 | -6.550 | -0.389 | 117 | 20 | enet | 0.110 | 4.00E-11 | 1.05E+00 | 2.94E-01 |
| TMED8 | 14 | 77801364 | 77843452 | 0.0798 | rs4899651 | -2.200 | rs17105724 | 0.121 | 7.310 | 1.389 | 144 | 12 | enet | 0.130 | 6.40E-13 | 1.05E+00 | 2.94E-01 |
| COMT | 22 | 19929130 | 19957498 | 0.4213 | rs756661 | 2.570 | rs4680 | 0.420 | 12.370 | -1.102 | 178 | 10 | enet | 0.400 | 2.40E-42 | -1.05E+00 | 2.94E-01 |
| HOMER2 | 15 | 83509838 | 83654661 | 0.0661 | rs17295457 | 1.680 | rs7178859 | 0.026 | 4.890 | 1.055 | 130 | 11 | enet | 0.027 | 8.40E-04 | 1.05E+00 | 2.96E-01 |
| BCAN | 1 | 156611182 | 156629324 | 0.1183 | rs6694580 | 1.997 | rs3795736 | 0.076 | -6.650 | 0.992 | 108 | 19 | enet | 0.100 | 1.20E-10 | -1.04E+00 | 2.96E-01 |
| AP5M1 | 14 | 57735627 | 57756797 | 0.2495 | rs2211582 | -0.990 | rs7143894 | 0.091 | -5.760 | -0.983 | 58 | 16 | enet | 0.084 | 1.10E-07 | 1.04E+00 | 2.96E-01 |
| NFX1 | 9 | 33290509 | 33371155 | 0.0252 | rs12683438 | 2.190 | rs626069 | 0.015 | -3.840 | 1.439 | 117 | 7 | enet | 0.016 | 1.10E-02 | -1.04E+00 | 2.97E-01 |
| KIAA1033 | 12 | 105501102 | 105562912 | 0.0437 | rs935251 | 2.300 | rs7298218 | 0.009 | 3.990 | -0.369 | 144 | 15 | enet | 0.011 | 2.20E-02 | -1.04E+00 | 2.98E-01 |
| CNDP2 | 18 | 72163051 | 72188366 | 0.1252 | rs1559806 | 2.540 | rs17089315 | 0.054 | 4.930 | -0.057 | 170 | 19 | enet | 0.059 | 1.10E-06 | 1.04E+00 | 2.99E-01 |
| FBXO6 | 1 | 11724181 | 11734411 | 0.0799 | rs12564559 | 3.552 | rs747862 | 0.028 | -4.610 | 0.884 | 96 | 5 | enet | 0.045 | 2.50E-05 | -1.04E+00 | 2.99E-01 |
| FAM65A | 16 | 67552321 | 67580691 | 0.0562 | rs34697097 | 1.847 | rs17686899 | 0.038 | -4.160 | 0.807 | 33 | 3 | enet | 0.011 | 2.60E-02 | -1.04E+00 | 2.99E-01 |
| COA7 | 1 | 53152508 | 53164038 | 0.1862 | rs681865 | 2.155 | rs443751 | 0.307 | -10.820 | 1.309 | 91 | 11 | enet | 0.300 | 2.20E-31 | -1.04E+00 | 3.00E-01 |
| CAMKK1 | 17 | 3763609 | 3798185 | 0.1711 | rs2074988 | 2.350 | rs2915546 | 0.074 | 5.570 | -0.809 | 101 | 20 | enet | 0.078 | 2.00E-08 | -1.04E+00 | 3.00E-01 |
| ZC3HAV1 | 7 | 138728266 | 138794465 | 0.0682 | rs10271373 | 4.310 | rs7801123 | 0.024 | 4.760 | 0.597 | 116 | 8 | enet | 0.020 | 3.60E-03 | 1.03E+00 | 3.01E-01 |
| FAM151B | 5 | 79783788 | 79838382 | 0.0888 | rs9687094 | 3.502 | rs17664442 | 0.069 | 4.530 | 1.276 | 100 | 12 | enet | 0.046 | 2.70E-04 | 1.03E+00 | 3.02E-01 |
| FUCA2 | 6 | 143815948 | 143832827 | 0.3450 | rs6911131 | 4.167 | rs11155297 | 0.222 | -8.310 | -0.955 | 140 | 22 | enet | 0.210 | 4.40E-17 | 1.03E+00 | 3.02E-01 |
| ITFG3 | 16 | 284545 | 319942 | 0.0959 | rs3213508 | -2.878 | rs11248914 | 0.010 | -4.060 | -0.229 | 113 | 17 | enet | 0.031 | 6.20E-04 | -1.03E+00 | 3.02E-01 |
| CORO2A | 9 | 100883257 | 100954922 | 0.1687 | rs7855251 | -5.480 | rs17810151 | 0.022 | -4.740 | 1.504 | 151 | 30 | enet | 0.098 | 3.20E-10 | 1.03E+00 | 3.02E-01 |
| COX7A2L | 2 | 42560686 | 42652228 | 0.0601 | rs13033481 | -1.498 | rs6544546 | 0.026 | 4.410 | -0.492 | 112 | 11 | enet | 0.019 | 4.30E-03 | -1.03E+00 | 3.03E-01 |
| ACY3 | 11 | 67410026 | 67418130 | 0.5463 | rs684928 | 1.700 | rs948445 | 0.238 | -8.870 | -0.984 | 67 | 7 | enet | 0.210 | 1.20E-17 | 1.03E+00 | 3.04E-01 |
| SVIP | 11 | 22835345 | 22851845 | 0.0736 | rs17570703 | 2.690 | rs10833823 | 0.015 | -4.080 | 0.427 | 84 | 18 | enet | 0.022 | 2.50E-03 | -1.03E+00 | 3.04E-01 |
| NT5C3B | 17 | 39981335 | 39992523 | 0.1276 | rs13412 | -4.180 | rs7212972 | 0.223 | 9.140 | -1.171 | 94 | 9 | enet | 0.200 | 4.10E-20 | -1.03E+00 | 3.04E-01 |
| ADK | 10 | 75910960 | 76469061 | 0.1029 | rs1874152 | 2.930 | rs10824092 | 0.110 | 6.630 | -1.190 | 164 | 12 | enet | 0.074 | 4.50E-08 | -1.03E+00 | 3.05E-01 |

| | | | | | | | | | | | | | | | | | |
|---|---|---|---|---|---|---|---|---|---|---|---|---|---|---|---|---|---|
| CANX | 5 | 179105629 | 179157926 | 0.0302 | rs12652934 | 2.197 | rs12515399 | 0.011 | -3.880 | -0.808 | 81 | 5 | enet | 0.010 | 3.20E-02 | 1.03E+00 | 3.05E-01 |
| KCTD5 | 16 | 2732476 | 2759031 | 0.0871 | rs9925824 | -1.690 | rs3112728 | 0.058 | 3.830 | 1.365 | 50 | 6 | enet | 0.043 | 1.70E-03 | 1.03E+00 | 3.05E-01 |
| GCA | 2 | 163175350 | 163228105 | 0.1890 | rs984971 | 2.845 | rs17783344 | 0.051 | -4.390 | 1.030 | 49 | 16 | enet | 0.063 | 1.90E-06 | -1.02E+00 | 3.06E-01 |
| SCP2 | 1 | 53392901 | 53517375 | 0.0309 | rs10437066 | 1.909 | rs536621 | 0.038 | -4.350 | -0.670 | 147 | 7 | enet | 0.017 | 7.10E-03 | 1.02E+00 | 3.07E-01 |
| LYPLAL1 | 1 | 219347186 | 219386207 | 0.1817 | rs7545152 | 1.725 | rs6699816 | 0.240 | 9.770 | 0.985 | 85 | 13 | enet | 0.220 | 2.30E-22 | 1.02E+00 | 3.07E-01 |
| SYNE1 | 6 | 152442819 | 152958936 | 0.0864 | rs6935362 | 2.674 | rs214987 | 0.035 | 4.500 | 0.598 | 466 | 28 | enet | 0.016 | 8.50E-03 | -1.02E+00 | 3.07E-01 |
| HDDC3 | 15 | 91474148 | 91475799 | 0.0575 | rs7171099 | 2.710 | rs6496749 | 0.004 | 3.320 | 0.920 | 108 | 37 | enet | 0.021 | 3.00E-03 | 1.02E+00 | 3.08E-01 |
| AGPAT3 | 21 | 45285067 | 45406417 | 0.0766 | rs4819312 | -2.360 | rs7276207 | 0.043 | 4.620 | 1.116 | 122 | 5 | enet | 0.033 | 2.10E-04 | 1.02E+00 | 3.09E-01 |
| PDE1B | 12 | 54943134 | 54973023 | 0.1529 | rs10747700 | -3.240 | rs10747699 | 0.093 | -6.080 | -2.246 | 131 | 18 | enet | 0.079 | 1.60E-08 | 1.02E+00 | 3.09E-01 |
| PGGT1B | 5 | 114546527 | 114598569 | 0.0310 | rs17137481 | 2.311 | rs265437 | 0.036 | -4.180 | -1.092 | 112 | 3 | enet | 0.005 | 9.00E-02 | 1.02E+00 | 3.10E-01 |
| GMPR | 6 | 16238811 | 16295780 | 0.0571 | rs2237217 | 2.140 | rs6459467 | 0.028 | 4.080 | 0.051 | 110 | 13 | enet | 0.021 | 3.00E-03 | -1.02E+00 | 3.10E-01 |
| TUBA8 | 22 | 18593097 | 18629321 | 0.1939 | rs5992185 | -2.680 | rs464541 | 0.047 | -5.010 | 1.329 | 94 | 26 | enet | 0.120 | 2.00E-12 | -1.01E+00 | 3.11E-01 |
| DOCK9 | 13 | 99445741 | 99738879 | 0.0544 | rs9557170 | -4.300 | rs9517505 | 0.008 | -4.010 | 1.233 | 273 | 19 | enet | 0.030 | 3.90E-04 | -1.01E+00 | 3.11E-01 |
| MTRR | 5 | 7851299 | 7906138 | 0.2010 | rs16879258 | -2.225 | rs3733784 | 0.114 | 6.470 | 0.352 | 194 | 36 | enet | 0.160 | 9.20E-13 | 1.01E+00 | 3.12E-01 |
| GOT2 | 16 | 58741035 | 58768261 | 0.0905 | rs4784975 | -3.174 | rs1616798 | 0.114 | 7.330 | 0.479 | 109 | 18 | enet | 0.130 | 2.10E-13 | 1.01E+00 | 3.12E-01 |
| AS3MT | 10 | 104629273 | 104661656 | 0.1281 | rs284859 | -2.520 | rs11191439 | 0.043 | -5.090 | 1.276 | 89 | 11 | enet | 0.062 | 7.30E-07 | -1.01E+00 | 3.13E-01 |
| ISLR | 15 | 74466012 | 74469213 | 0.0729 | rs7175533 | 1.410 | rs8033129 | -0.003 | 3.810 | -1.067 | 60 | 13 | enet | 0.005 | 1.20E-01 | -1.01E+00 | 3.13E-01 |
| MCTP1 | 5 | 94039446 | 94620279 | 0.0843 | rs6885525 | 3.477 | rs2112450 | 0.083 | -5.830 | -0.441 | 343 | 9 | enet | 0.100 | 9.50E-11 | 1.01E+00 | 3.14E-01 |
| TMEM2 | 9 | 74298282 | 74431606 | 0.0623 | rs923704 | -2.570 | rs25692 | 0.008 | -3.720 | 1.039 | 123 | 11 | enet | 0.019 | 6.00E-03 | -1.01E+00 | 3.15E-01 |
| PIP5K1B | 9 | 71320575 | 71624092 | 0.0566 | rs9314845 | 2.750 | rs10869403 | 0.013 | 3.780 | -1.340 | 276 | 17 | enet | 0.006 | 8.60E-02 | -1.00E+00 | 3.15E-01 |
| GSTM4 | 1 | 110198703 | 110208118 | 0.1517 | rs11102002 | -2.673 | rs524998 | 0.046 | -5.560 | -0.283 | 90 | 36 | enet | 0.110 | 8.20E-12 | 1.01E+00 | 3.15E-01 |
| NT5E | 6 | 86159809 | 86205500 | 0.2099 | rs7771366 | -2.375 | rs4373339 | 0.200 | 8.730 | -1.135 | 96 | 18 | enet | 0.260 | 1.00E-26 | -1.00E+00 | 3.15E-01 |
| SLC25A13 | 7 | 95749532 | 95951459 | 0.0728 | rs3757698 | 1.430 | rs1989825 | 0.081 | 5.840 | 1.329 | 162 | 34 | enet | 0.067 | 2.00E-07 | 1.00E+00 | 3.15E-01 |
| SYNPR | 3 | 63213991 | 63602597 | 0.0836 | rs41494245 | -1.730 | rs7647554 | 0.040 | 4.610 | -1.225 | 383 | 16 | enet | 0.040 | 5.20E-05 | -1.00E+00 | 3.17E-01 |
| RAB3IP | 12 | 70132461 | 70216984 | 0.0658 | rs11177793 | -2.710 | rs10506569 | 0.043 | 4.100 | -0.077 | 182 | 26 | enet | 0.011 | 2.80E-02 | 9.99E-01 | 3.18E-01 |
| VPS13A | 9 | 79792269 | 80036457 | 0.0734 | rs2377903 | -2.620 | rs11790663 | 0.013 | 3.830 | 1.353 | 189 | 16 | enet | 0.021 | 2.80E-03 | 9.97E-01 | 3.19E-01 |
| RAB3GAP2 | 1 | 220321635 | 220445796 | 0.0534 | rs2808027 | -2.190 | rs11805285 | 0.054 | -5.030 | 0.923 | 114 | 11 | enet | 0.042 | 3.80E-05 | -9.95E-01 | 3.20E-01 |
| MTIF2 | 2 | 55463731 | 55496483 | 0.0739 | rs13012766 | -2.190 | rs749221 | -0.002 | -2.990 | -0.563 | 102 | 7 | enet | 0.004 | 1.10E-01 | 9.92E-01 | 3.21E-01 |
| FAIM | 3 | 138327448 | 138352218 | 0.0653 | rs10513055 | 1.990 | rs1672928 | 0.063 | -4.450 | 0.992 | 68 | 1 | enet | 0.051 | 7.40E-05 | -9.92E-01 | 3.21E-01 |
| SORCS1 | 10 | 108333421 | 108924292 | 0.1225 | rs7895561 | 3.350 | rs1336621 | -0.002 | -3.650 | -2.038 | 350 | 44 | enet | 0.025 | 1.70E-03 | 9.92E-01 | 3.21E-01 |
| REEP6 | 19 | 1491165 | 1497926 | 0.1380 | rs2238591 | 1.920 | rs2656885 | 0.115 | -6.490 | -0.560 | 48 | 13 | enet | 0.100 | 7.80E-10 | 9.92E-01 | 3.21E-01 |
| PARK2 | 6 | 161768452 | 163148803 | 0.0988 | rs7747985 | -4.325 | rs2064419 | 0.049 | -4.530 | 1.069 | 946 | 11 | enet | 0.020 | 3.80E-03 | -9.91E-01 | 3.22E-01 |
| CAPN2 | 1 | 223889295 | 223963720 | 0.1233 | rs6693884 | 2.779 | rs10915917 | 0.046 | -5.420 | 0.245 | 118 | 18 | enet | 0.051 | 5.30E-06 | -9.87E-01 | 3.24E-01 |
| C2orf43 | 2 | 20883788 | 21022882 | 0.3498 | rs6531255 | 1.820 | rs13385191 | 0.378 | -11.170 | 1.085 | 159 | 10 | enet | 0.360 | 2.30E-33 | -9.87E-01 | 3.24E-01 |
| PACSIN3 | 11 | 47199076 | 47207994 | 0.1080 | rs901746 | 4.710 | rs1352307 | 0.068 | 5.940 | -0.738 | 83 | 15 | enet | 0.077 | 5.00E-08 | 9.85E-01 | 3.24E-01 |
| KIF21B | 1 | 200938520 | 200992828 | 0.2575 | rs7554511 | -6.591 | rs502658 | 0.140 | 8.760 | 0.233 | 151 | 13 | enet | 0.250 | 3.30E-25 | -9.85E-01 | 3.25E-01 |
| ACADM | 1 | 76190036 | 76253260 | 0.1896 | rs11163860 | 2.876 | rs11161521 | 0.242 | 9.680 | -1.384 | 110 | 23 | enet | 0.300 | 9.70E-31 | -9.82E-01 | 3.26E-01 |
| ACAD8 | 11 | 134123389 | 134135749 | 0.0698 | rs554874 | 2.070 | rs1146188 | 0.003 | 3.620 | -0.394 | 87 | 19 | enet | 0.007 | 6.20E-02 | 9.81E-01 | 3.26E-01 |
| FN3K | 17 | 80693451 | 80709073 | 0.2998 | rs3898431 | 1.390 | rs2451219 | 0.000 | -3.240 | -0.818 | 80 | 24 | enet | 0.033 | 2.40E-04 | -9.83E-01 | 3.26E-01 |
| UQCR10 | 22 | 30163358 | 30166402 | 0.2444 | rs9614040 | 3.770 | rs17711508 | 0.285 | -10.540 | 1.043 | 84 | 5 | enet | 0.270 | 3.20E-27 | -9.82E-01 | 3.26E-01 |
| PLD1 | 3 | 171318195 | 171528740 | 0.0371 | rs4378993 | 3.180 | rs3843979 | 0.049 | 4.300 | -1.441 | 192 | 17 | enet | 0.010 | 4.10E-02 | -9.80E-01 | 3.27E-01 |
| MESDC2 | 15 | 81239667 | 81282219 | 0.1010 | rs6495528 | -1.760 | rs3743041 | 0.100 | 6.550 | 1.076 | 102 | 19 | enet | 0.088 | 2.50E-09 | 9.77E-01 | 3.28E-01 |
| AACS | 12 | 125549925 | 125627873 | 0.0940 | rs10846807 | 2.630 | rs10846823 | 0.071 | 6.020 | -1.575 | 126 | 14 | enet | 0.100 | 1.00E-10 | -9.75E-01 | 3.29E-01 |
| CAB39L | 13 | 49882786 | 50018262 | 0.5688 | rs17072833 | 2.330 | rs4941638 | 0.371 | -11.850 | 1.328 | 142 | 30 | enet | 0.490 | 3.10E-56 | -9.76E-01 | 3.29E-01 |
| PPP2R5B | 11 | 64685025 | 64701945 | 0.0363 | rs11231903 | -2.460 | rs582580 | -0.003 | -3.620 | 0.854 | 73 | 11 | enet | 0.007 | 5.80E-02 | -9.74E-01 | 3.30E-01 |
| PPA2 | 4 | 106290234 | 106395238 | 0.3112 | rs2726507 | -4.500 | rs17617811 | 0.071 | -6.860 | 0.346 | 144 | 20 | enet | 0.190 | 1.90E-19 | -9.74E-01 | 3.30E-01 |

| | | | | | | | | | | | | | | | | | |
|---|---|---|---|---|---|---|---|---|---|---|---|---|---|---|---|---|---|
| HSD17B11 | 4 | 88257762 | 88312538 | 0.1066 | rs7674452 | -1.780 | rs3923442 | 0.014 | -4.810 | 0.378 | 87 | 12 | enet | 0.036 | 1.20E-04 | -9.72E-01 | 3.31E-01 |
| RGS6 | 14 | 72399156 | 73030654 | 0.1832 | rs11629255 | 3.020 | rs2238284 | 0.109 | -6.800 | 0.160 | 551 | 34 | enet | 0.130 | 1.20E-13 | 9.69E-01 | 3.32E-01 |
| FDXR | 17 | 72858619 | 72869156 | 0.3598 | rs7226145 | -1.840 | rs690514 | 0.550 | -14.410 | 0.970 | 102 | 19 | enet | 0.540 | 1.50E-65 | -9.68E-01 | 3.33E-01 |
| CAST | 5 | 95860971 | 96115299 | 0.2134 | rs155040 | 2.890 | rs152005 | 0.130 | 7.170 | -0.872 | 297 | 29 | enet | 0.130 | 1.20E-13 | -9.67E-01 | 3.34E-01 |
| PRODH | 22 | 18900294 | 18924066 | 0.4356 | rs2238732 | -1.900 | rs2005883 | 0.089 | -7.130 | -0.961 | 60 | 42 | enet | 0.290 | 9.00E-30 | 9.64E-01 | 3.35E-01 |
| CD2AP | 6 | 47445525 | 47594999 | 0.0598 | rs2184397 | -3.291 | rs9381563 | 0.008 | 3.650 | -3.116 | 146 | 15 | enet | 0.033 | 3.70E-04 | -9.62E-01 | 3.36E-01 |
| PEX6 | 6 | 42931608 | 42946958 | 0.0591 | rs6941129 | 1.614 | rs2395943 | 0.036 | -4.970 | 0.606 | 86 | 12 | enet | 0.050 | 3.60E-05 | -9.61E-01 | 3.37E-01 |
| RMND1 | 6 | 151725989 | 151773259 | 0.2950 | rs3734797 | -2.244 | rs11550103 | 0.247 | -10.160 | -0.529 | 133 | 22 | enet | 0.260 | 2.60E-26 | 9.60E-01 | 3.37E-01 |
| SHD | 19 | 4278598 | 4290721 | 0.0842 | rs12462529 | -3.690 | rs4740 | 0.074 | 6.120 | -0.062 | 74 | 19 | enet | 0.055 | 4.20E-06 | 9.60E-01 | 3.37E-01 |
| JMJD7 | 15 | 42120283 | 42129779 | 0.1073 | rs747779 | -2.080 | rs12440605 | 0.087 | -6.590 | -0.579 | 96 | 8 | enet | 0.140 | 4.30E-13 | 9.60E-01 | 3.37E-01 |
| TBC1D4 | 13 | 75858808 | 76056250 | 0.1278 | rs535468 | 1.940 | rs9565168 | 0.045 | -4.500 | 1.321 | 222 | 32 | enet | 0.024 | 1.40E-03 | -9.58E-01 | 3.38E-01 |
| FAM171A1 | 10 | 15253642 | 15413061 | 0.3488 | rs11259683 | 2.350 | rs3814165 | 0.298 | -10.790 | 1.365 | 198 | 16 | enet | 0.310 | 7.80E-32 | -9.58E-01 | 3.38E-01 |
| ATAD3A | 1 | 1447531 | 1470067 | 0.0273 | rs7517401 | 2.242 | rs3766169 | -0.002 | -2.740 | 1.900 | 26 | 14 | enet | 0.001 | 2.70E-01 | -9.56E-01 | 3.39E-01 |
| HDHD3 | 9 | 116135699 | 116139279 | 0.4942 | rs4979240 | 3.060 | rs1043836 | 0.167 | -9.030 | -1.585 | 121 | 19 | enet | 0.260 | 3.40E-26 | 9.55E-01 | 3.40E-01 |
| ANXA11 | 10 | 81910645 | 81965328 | 0.3596 | rs12146166 | -2.040 | rs12763624 | 0.187 | -8.470 | 0.716 | 136 | 14 | enet | 0.180 | 6.20E-18 | -9.54E-01 | 3.40E-01 |
| CALU | 7 | 128379346 | 128411861 | 0.0991 | rs17164371 | 1.570 | rs1043550 | 0.147 | 7.540 | 0.834 | 111 | 23 | enet | 0.130 | 1.10E-13 | 9.54E-01 | 3.40E-01 |
| SLC2A5 | 1 | 9095166 | 9148537 | 0.2191 | rs770041 | -2.330 | rs12239636 | -0.002 | 4.770 | 0.068 | 133 | 22 | enet | 0.110 | 1.30E-11 | 9.53E-01 | 3.41E-01 |
| SLC15A2 | 3 | 121612936 | 121662949 | 0.2293 | rs1962532 | 7.160 | rs11920521 | 0.261 | -9.980 | -0.538 | 105 | 26 | enet | 0.300 | 3.00E-31 | -9.50E-01 | 3.42E-01 |
| C3orf18 | 3 | 50595462 | 50608458 | 0.0582 | rs2040397 | 1.820 | rs13076850 | 0.093 | 5.140 | -1.035 | 49 | 8 | enet | 0.086 | 8.10E-06 | -9.48E-01 | 3.43E-01 |
| RUVBL1 | 3 | 127783621 | 127872757 | 0.0835 | rs16839675 | 1.620 | rs11717102 | 0.041 | -5.100 | -0.728 | 94 | 8 | enet | 0.044 | 2.40E-05 | 9.46E-01 | 3.44E-01 |
| STRN | 2 | 37070783 | 37193615 | 0.0869 | rs2160411 | 2.237 | rs10495870 | 0.043 | 4.610 | 0.707 | 129 | 5 | enet | 0.017 | 6.80E-03 | 9.44E-01 | 3.45E-01 |
| SLC27A2 | 15 | 50474393 | 50528592 | 0.0357 | rs17740607 | -2.560 | rs34146758 | 0.015 | -3.990 | -0.421 | 122 | 13 | enet | 0.003 | 1.70E-01 | 9.43E-01 | 3.46E-01 |
| BBOX1 | 11 | 27062272 | 27149356 | 0.2223 | rs6484268 | -2.320 | rs7106917 | 0.138 | 7.610 | -0.520 | 146 | 19 | enet | 0.190 | 6.30E-19 | -9.43E-01 | 3.46E-01 |
| PTER | 10 | 16478942 | 16555736 | 0.2226 | rs6602097 | 2.920 | rs1532104 | 0.356 | -11.280 | -0.547 | 150 | 22 | enet | 0.340 | 1.70E-33 | 9.42E-01 | 3.46E-01 |
| CFHR1 | 1 | 196788887 | 196801319 | 0.1962 | rs6657442 | 1.198 | rs16840522 | 0.208 | -8.010 | 0.720 | 29 | 6 | enet | 0.250 | 3.80E-20 | -9.39E-01 | 3.48E-01 |
| ABCF3 | 3 | 183903811 | 183911800 | 0.0430 | rs35637422 | -2.420 | rs6787471 | 0.024 | -4.340 | -0.903 | 89 | 8 | enet | 0.013 | 1.60E-02 | 9.38E-01 | 3.48E-01 |
| GTDC1 | 2 | 144695635 | 145090135 | 0.0615 | rs11888238 | 4.343 | rs3820755 | 0.019 | -4.100 | 1.049 | 150 | 18 | enet | 0.015 | 1.00E-02 | -9.36E-01 | 3.49E-01 |
| HDHD2 | 18 | 44633774 | 44676891 | 0.1293 | rs11876448 | 3.130 | rs2117373 | 0.037 | 4.780 | 1.985 | 91 | 24 | enet | 0.054 | 2.90E-06 | 9.35E-01 | 3.50E-01 |
| PRTFDC1 | 10 | 25137536 | 25241533 | 0.1698 | rs1572364 | -2.130 | rs274296 | 0.084 | -5.940 | 0.480 | 110 | 10 | enet | 0.100 | 2.00E-10 | -9.34E-01 | 3.50E-01 |
| IFIT2 | 10 | 91061712 | 91069033 | 0.1194 | rs304500 | 2.570 | rs1727 | 0.106 | -6.170 | 1.552 | 111 | 6 | enet | 0.089 | 1.50E-08 | -9.33E-01 | 3.51E-01 |
| H6PD | 1 | 9294834 | 9331396 | 0.1393 | rs4465231 | -4.116 | rs2268169 | 0.044 | 4.710 | 0.178 | 122 | 15 | enet | 0.120 | 1.50E-10 | 9.33E-01 | 3.51E-01 |
| VIPAS39 | 14 | 77893018 | 77924295 | 0.0975 | rs11846597 | 1.910 | rs17824094 | 0.087 | -6.430 | 0.994 | 107 | 10 | enet | 0.052 | 4.60E-06 | -9.32E-01 | 3.51E-01 |
| INPP5E | 9 | 139323071 | 139334274 | 0.1185 | rs2229971 | 1.750 | rs11146021 | 0.081 | -5.750 | -0.950 | 107 | 22 | enet | 0.065 | 1.50E-06 | 9.32E-01 | 3.51E-01 |
| SORD | 15 | 45315302 | 45369383 | 0.3405 | rs2470667 | -1.770 | rs17595239 | 0.005 | 3.510 | 1.277 | 71 | 25 | enet | 0.056 | 2.10E-06 | 9.30E-01 | 3.52E-01 |
| HDGF | 1 | 156711899 | 156736717 | 0.1053 | rs2298135 | 1.665 | rs11264534 | 0.138 | -7.450 | 1.042 | 88 | 13 | enet | 0.130 | 5.00E-13 | -9.29E-01 | 3.53E-01 |
| CAT | 11 | 34460472 | 34493609 | 0.1178 | rs2231823 | 2.050 | rs2284369 | 0.111 | -6.800 | 0.982 | 188 | 24 | enet | 0.110 | 1.50E-11 | -9.30E-01 | 3.53E-01 |
| CAMLG | 5 | 134074191 | 134087847 | 0.2035 | rs11747528 | -3.477 | rs2108665 | 0.172 | -8.140 | -0.865 | 29 | 17 | enet | 0.150 | 5.20E-15 | 9.22E-01 | 3.56E-01 |
| ELP3 | 8 | 27947190 | 28048673 | 0.0328 | rs1381114 | -3.450 | rs11784168 | 0.003 | 3.530 | -0.601 | 174 | 12 | enet | 0.015 | 1.00E-02 | 9.23E-01 | 3.56E-01 |
| IQSEC1 | 3 | 12938719 | 13114617 | 0.1690 | rs181722 | 2.700 | rs444944 | 0.106 | -6.640 | 0.936 | 212 | 10 | enet | 0.072 | 6.60E-08 | -9.19E-01 | 3.58E-01 |
| ACOX3 | 4 | 8368009 | 8442450 | 0.3896 | rs3796751 | 3.220 | rs3796751 | 0.111 | 7.760 | 3.221 | 182 | 52 | enet | 0.300 | 1.10E-30 | 9.18E-01 | 3.59E-01 |
| ANXA2 | 15 | 60639333 | 60695082 | 0.0913 | rs7176730 | -2.360 | rs11858787 | 0.002 | -3.420 | -0.784 | 124 | 19 | enet | 0.038 | 7.70E-05 | 9.14E-01 | 3.61E-01 |
| PDIA4 | 7 | 148700154 | 148725733 | 0.1564 | rs6464927 | -2.210 | rs12534095 | 0.226 | 9.440 | -0.503 | 64 | 13 | enet | 0.200 | 9.80E-20 | -9.14E-01 | 3.61E-01 |
| PLCL1 | 2 | 198669426 | 199437305 | 0.0653 | rs2529672 | 2.763 | rs1595823 | 0.039 | 5.270 | 1.307 | 302 | 12 | enet | 0.041 | 4.70E-05 | 9.14E-01 | 3.61E-01 |
| PNOC | 8 | 28174503 | 28200872 | 0.0673 | rs1381114 | -3.450 | rs6985192 | -0.004 | 3.180 | 1.707 | 119 | 17 | enet | 0.009 | 7.90E-02 | 9.13E-01 | 3.61E-01 |
| TTYH3 | 7 | 2671585 | 2704436 | 0.1006 | rs2969039 | -2.290 | rs1105421 | 0.048 | 4.600 | -1.274 | 121 | 12 | enet | 0.093 | 8.30E-10 | -9.13E-01 | 3.61E-01 |
| FDX1L | 19 | 10416103 | 10426691 | 0.1896 | rs12611227 | -5.230 | rs12720356 | 0.091 | -6.240 | 1.246 | 88 | 13 | enet | 0.092 | 1.10E-09 | 9.13E-01 | 3.61E-01 |

| | | | | | | | | | | | | | | | | | |
|---|---|---|---|---|---|---|---|---|---|---|---|---|---|---|---|---|---|
| VWA8 | 13 | 42140973 | 42535256 | 0.2714 | rs12429256 | 4.890 | rs9594643 | 0.242 | -9.610 | -1.731 | 250 | 23 | enet | 0.250 | 4.60E-25 | 9.12E-01 | 3.62E-01 |
| PLSCR4 | 3 | 145910126 | 145968966 | 0.2396 | rs9838866 | 1.410 | rs3762687 | 0.277 | 10.400 | -0.894 | 98 | 11 | enet | 0.270 | 1.30E-27 | -9.11E-01 | 3.63E-01 |
| TMEM141 | 9 | 139685807 | 139687709 | 0.2755 | rs4880136 | 3.250 | rs11145887 | 0.122 | 6.030 | 1.239 | 67 | 5 | enet | 0.075 | 2.50E-06 | 9.08E-01 | 3.64E-01 |
| PHF3 | 6 | 64345725 | 64489229 | 0.0518 | rs6922047 | -1.211 | rs1322416 | 0.040 | -3.600 | 1.088 | 60 | 4 | enet | 0.038 | 4.90E-04 | -9.07E-01 | 3.64E-01 |
| LYRM9 | 17 | 26205340 | 26221778 | 0.1974 | rs12709500 | 2.430 | rs3751972 | 0.338 | -11.520 | -0.722 | 76 | 11 | enet | 0.360 | 6.80E-39 | 9.07E-01 | 3.65E-01 |
| NRP2 | 2 | 206546714 | 206662857 | 0.1321 | rs2075208 | -2.475 | rs849523 | 0.036 | 3.920 | -0.926 | 150 | 33 | enet | 0.020 | 3.60E-03 | -9.03E-01 | 3.67E-01 |
| CA8 | 8 | 61099906 | 61193971 | 0.1657 | rs2681560 | -2.530 | rs16926000 | 0.067 | -6.140 | -0.206 | 141 | 18 | enet | 0.060 | 8.80E-07 | 8.99E-01 | 3.69E-01 |
| LARS2 | 3 | 45429998 | 45590913 | 0.3065 | rs2005227 | -3.360 | rs12638088 | 0.333 | -11.370 | 0.444 | 159 | 19 | enet | 0.350 | 1.50E-36 | -8.97E-01 | 3.70E-01 |
| CCDC90B | 11 | 82970139 | 82997450 | 0.0654 | rs17144746 | -2.640 | rs494791 | 0.042 | -4.490 | 0.562 | 71 | 15 | enet | 0.017 | 5.90E-03 | -8.96E-01 | 3.70E-01 |
| ITPA | 20 | 3189514 | 3204516 | 0.7651 | rs6084327 | 2.105 | rs6139030 | 0.522 | -14.110 | 0.860 | 96 | 20 | enet | 0.690 | 3.50E-97 | -8.97E-01 | 3.70E-01 |
| ARL2 | 11 | 64781585 | 64789656 | 0.0439 | rs1129195 | 2.470 | rs664226 | 0.028 | 4.410 | -0.696 | 89 | 4 | enet | 0.023 | 2.00E-03 | -8.94E-01 | 3.72E-01 |
| B3GALTL | 13 | 31774073 | 31906413 | 0.3036 | rs7997689 | 1.490 | rs7986755 | 0.267 | 10.550 | 0.441 | 177 | 21 | enet | 0.390 | 1.60E-41 | 8.90E-01 | 3.73E-01 |
| CPLX1 | 4 | 778745 | 819986 | 0.0365 | rs11946340 | 3.840 | rs4690322 | 0.022 | -4.100 | 0.115 | 111 | 9 | enet | 0.027 | 8.50E-04 | -8.90E-01 | 3.74E-01 |
| NT5C2 | 10 | 104845940 | 104953056 | 0.0391 | rs1163249 | -2.410 | rs12764154 | 0.002 | 3.540 | 0.469 | 95 | 6 | enet | 0.013 | 1.70E-02 | 8.90E-01 | 3.74E-01 |
| ATP13A1 | 19 | 19756007 | 19774502 | 0.1583 | rs6511047 | 2.490 | rs2304130 | 0.123 | 7.010 | -1.228 | 94 | 7 | enet | 0.130 | 5.50E-13 | -8.89E-01 | 3.74E-01 |
| LMAN1 | 18 | 56995055 | 57027194 | 0.0800 | rs10221365 | 1.720 | rs12604758 | 0.071 | -5.400 | -1.257 | 136 | 17 | enet | 0.066 | 2.50E-07 | 8.88E-01 | 3.75E-01 |
| LIN7A | 12 | 81186299 | 81331704 | 0.0731 | rs3782165 | -2.190 | rs7977901 | 0.048 | -5.170 | -0.875 | 150 | 9 | enet | 0.027 | 7.50E-04 | 8.86E-01 | 3.75E-01 |
| PGM2 | 4 | 37828255 | 37864558 | 0.2259 | rs6531596 | -1.900 | rs3775793 | 0.406 | 12.410 | 0.836 | 174 | 20 | enet | 0.400 | 4.40E-43 | 8.87E-01 | 3.75E-01 |
| FRRS1L | 9 | 111892573 | 111929571 | 0.0437 | rs614253 | -2.830 | rs10979694 | 0.039 | 4.870 | 1.092 | 127 | 7 | enet | 0.021 | 2.50E-03 | 8.87E-01 | 3.75E-01 |
| TUBA4A | 2 | 220114433 | 220142892 | 0.0240 | rs6709228 | -3.090 | rs4485556 | 0.019 | -3.460 | 0.267 | 99 | 17 | enet | 0.009 | 3.30E-02 | -8.86E-01 | 3.76E-01 |
| ITM2C | 2 | 231729354 | 231743963 | 0.0978 | rs3106301 | 2.301 | rs4973353 | 0.062 | -5.490 | 1.452 | 104 | 14 | enet | 0.053 | 4.70E-06 | -8.85E-01 | 3.76E-01 |
| SLC25A10 | 17 | 79670404 | 79688042 | 0.0343 | rs9895741 | 2.390 | rs12452251 | 0.033 | 4.050 | 1.092 | 63 | 6 | enet | 0.022 | 2.10E-03 | 8.85E-01 | 3.76E-01 |
| AMACR | 5 | 33986283 | 34008220 | 0.4115 | rs840381 | 2.117 | rs2278008 | 0.413 | -11.190 | 0.802 | 102 | 11 | enet | 0.460 | 4.50E-39 | -8.80E-01 | 3.79E-01 |
| RDH13 | 19 | 55550476 | 55582659 | 0.2091 | rs12610286 | -2.990 | rs12981732 | 0.022 | 5.080 | 1.959 | 123 | 41 | enet | 0.170 | 3.40E-17 | 8.77E-01 | 3.81E-01 |
| S100B | 21 | 48018875 | 48025121 | 0.1408 | rs9981272 | 2.610 | rs7282606 | 0.065 | -6.180 | 1.010 | 95 | 17 | enet | 0.150 | 2.30E-13 | -8.77E-01 | 3.81E-01 |
| THSD7A | 7 | 11409984 | 11871824 | 0.1222 | rs10232201 | -2.630 | rs1526554 | 0.007 | 4.230 | 0.162 | 465 | 32 | enet | 0.004 | 1.20E-01 | 8.76E-01 | 3.81E-01 |
| THNSL2 | 2 | 88469835 | 88486146 | 0.4757 | rs2919851 | -1.883 | rs10865489 | 0.396 | -10.400 | -1.195 | 126 | 18 | enet | 0.470 | 6.70E-39 | 8.75E-01 | 3.82E-01 |
| TMEM177 | 2 | 120436743 | 120444083 | 0.0385 | rs11123535 | -1.652 | rs4260253 | 0.022 | 4.450 | -0.573 | 52 | 8 | enet | 0.021 | 3.90E-03 | -8.72E-01 | 3.83E-01 |
| PLEKHB1 | 11 | 73357223 | 73373864 | 0.0297 | rs7940286 | -2.320 | rs1525749 | 0.010 | 3.520 | -1.775 | 73 | 7 | enet | 0.001 | 2.40E-01 | -8.72E-01 | 3.83E-01 |
| NIT2 | 3 | 100053545 | 100075710 | 0.6479 | rs1132022 | -2.570 | rs2289506 | 0.640 | -15.390 | -1.225 | 68 | 19 | enet | 0.620 | 1.40E-78 | 8.72E-01 | 3.83E-01 |
| ENPP6 | 4 | 185009859 | 185142383 | 0.1122 | rs7655396 | 3.040 | rs1882328 | 0.050 | -5.870 | -0.639 | 207 | 6 | enet | 0.076 | 3.40E-08 | 8.69E-01 | 3.85E-01 |
| DDHD1 | 14 | 53510686 | 53620000 | 0.0197 | rs12432010 | 2.300 | rs1255257 | 0.011 | -3.770 | -1.295 | 95 | 7 | enet | 0.011 | 2.60E-02 | 8.68E-01 | 3.85E-01 |
| NIF3L1 | 2 | 201754050 | 201768655 | 0.0463 | rs11685853 | 2.787 | rs11894842 | 0.028 | 4.080 | -0.138 | 53 | 12 | enet | -0.001 | 4.10E-01 | -8.65E-01 | 3.87E-01 |
| PLXNA2 | 1 | 208195587 | 208417665 | 0.0462 | rs6676233 | -2.597 | rs556625 | 0.003 | -4.160 | -0.494 | 263 | 9 | enet | 0.012 | 2.10E-02 | 8.63E-01 | 3.88E-01 |
| FAF1 | 1 | 50905150 | 51425935 | 0.1407 | rs1464081 | 1.988 | rs35491793 | 0.075 | 5.510 | 0.872 | 164 | 10 | enet | 0.043 | 2.80E-05 | 8.64E-01 | 3.88E-01 |
| STOM | 9 | 124101355 | 124132531 | 0.2800 | rs7857673 | -1.200 | rs306781 | 0.009 | -3.540 | 0.237 | 91 | 28 | enet | 0.077 | 2.40E-08 | -8.63E-01 | 3.88E-01 |
| PCK2 | 14 | 24563262 | 24579807 | 0.0559 | rs10142648 | -2.540 | rs2759409 | 0.008 | 3.760 | 1.334 | 71 | 8 | enet | 0.031 | 3.40E-04 | 8.61E-01 | 3.89E-01 |
| ECH1 | 19 | 39306062 | 39322645 | 0.2702 | rs3136646 | -3.080 | rs2229259 | 0.173 | -8.470 | -1.032 | 81 | 5 | enet | 0.150 | 9.80E-15 | 8.60E-01 | 3.90E-01 |
| CD109 | 6 | 74405508 | 74538040 | 0.0713 | rs10805984 | 1.793 | rs4708076 | 0.021 | 3.870 | 0.189 | 150 | 8 | enet | 0.013 | 1.50E-02 | 8.60E-01 | 3.90E-01 |
| ME3 | 11 | 86152150 | 86383678 | 0.1414 | rs2433409 | 2.720 | rs12362089 | 0.017 | 4.290 | 2.238 | 292 | 20 | enet | 0.020 | 3.30E-03 | 8.59E-01 | 3.90E-01 |
| DAB2IP | 9 | 124329336 | 124547809 | 0.0720 | rs10760198 | -3.380 | rs1571801 | 0.023 | -3.950 | -0.551 | 176 | 11 | enet | 0.045 | 2.20E-05 | 8.59E-01 | 3.90E-01 |
| RNF141 | 11 | 10533225 | 10562777 | 0.2570 | rs10840406 | 2.420 | rs12448 | 0.205 | 8.880 | -0.622 | 145 | 29 | enet | 0.220 | 3.90E-22 | -8.58E-01 | 3.91E-01 |
| NME1-NME2 | 17 | 49230920 | 49249108 | 0.1238 | rs17574235 | -3.270 | rs10514981 | 0.076 | -5.530 | -1.086 | 71 | 21 | enet | 0.076 | 3.40E-08 | 8.58E-01 | 3.91E-01 |
| MTAP | 9 | 21802542 | 21931646 | 0.1965 | rs2811711 | -4.160 | rs10757257 | 0.247 | -9.820 | 0.832 | 148 | 12 | enet | 0.200 | 2.80E-20 | -8.56E-01 | 3.92E-01 |
| AK3 | 9 | 4711155 | 4742043 | 0.4068 | rs10815051 | -2.880 | rs435100 | 0.067 | -6.980 | 0.038 | 153 | 35 | enet | 0.240 | 1.80E-24 | -8.54E-01 | 3.93E-01 |
| CECR5 | 22 | 17618401 | 17646177 | 0.1252 | rs741139 | 2.230 | rs1034859 | 0.080 | 5.590 | 0.065 | 131 | 20 | enet | 0.075 | 5.20E-08 | -8.52E-01 | 3.94E-01 |

| | | | | | | | | | | | | | | | | | |
|---|---|---|---|---|---|---|---|---|---|---|---|---|---|---|---|---|---|
| TTL | 2 | 113239731 | 113299316 | 0.0347 | rs4849067 | 2.113 | rs7581524 | 0.013 | 3.570 | 0.749 | 70 | 8 | enet | 0.010 | 3.50E-02 | 8.51E-01 | 3.95E-01 |
| DFNA5 | 7 | 24737972 | 24809244 | 0.3504 | rs2711132 | -2.020 | rs2237306 | 0.177 | 8.740 | 0.725 | 147 | 49 | enet | 0.270 | 1.70E-27 | 8.49E-01 | 3.96E-01 |
| MGARP | 4 | 140187317 | 140201492 | 0.1983 | rs6812491 | 2.650 | rs7673213 | 0.057 | 5.230 | -1.836 | 69 | 16 | enet | 0.070 | 7.80E-07 | -8.49E-01 | 3.96E-01 |
| SNAP91 | 6 | 84262599 | 84419410 | 0.0537 | rs910681 | 2.324 | rs2224195 | 0.014 | -4.090 | -0.915 | 137 | 14 | enet | 0.019 | 3.80E-03 | 8.49E-01 | 3.96E-01 |
| MANF | 3 | 51422478 | 51426828 | 0.0431 | rs7620081 | 2.180 | rs751075 | 0.043 | -4.710 | -0.863 | 73 | 12 | enet | 0.023 | 1.90E-03 | 8.44E-01 | 3.98E-01 |
| TSTA3 | 8 | 144694788 | 144700218 | 0.1296 | rs7829491 | -1.760 | rs7817301 | 0.033 | 4.680 | -0.158 | 85 | 25 | enet | 0.044 | 2.60E-05 | -8.43E-01 | 3.99E-01 |
| GPSM1 | 9 | 139221932 | 139254057 | 0.3046 | rs12684275 | 1.100 | rs28642252 | 0.136 | -7.630 | 0.763 | 77 | 15 | enet | 0.160 | 1.90E-16 | -8.42E-01 | 4.00E-01 |
| SLC14A1 | 18 | 43304092 | 43332485 | 0.3210 | rs894050 | -3.910 | rs10853535 | 0.186 | 8.740 | -1.549 | 170 | 23 | enet | 0.300 | 2.00E-29 | -8.42E-01 | 4.00E-01 |
| MACF1 | 1 | 39546988 | 39952849 | 0.0517 | rs3901310 | 2.726 | rs1539435 | 0.040 | -4.870 | 0.760 | 203 | 2 | enet | 0.027 | 7.20E-04 | -8.42E-01 | 4.00E-01 |
| RP11-139H15 | 15 | 55609382 | 55611359 | 0.1509 | rs549914 | -2.410 | rs8024695 | 0.049 | -4.720 | -0.146 | 77 | 23 | enet | 0.054 | 3.60E-06 | -8.36E-01 | 4.03E-01 |
| C21orf2 | 21 | 45748827 | 45759285 | 0.1151 | rs1055311 | 2.440 | rs2070573 | -0.003 | 3.350 | 0.378 | 70 | 27 | enet | 0.006 | 9.20E-02 | 8.29E-01 | 4.07E-01 |
| FAM136A | 2 | 70523107 | 70529222 | 0.3607 | rs11900489 | 2.711 | rs4852161 | 0.060 | -5.120 | -0.086 | 84 | 26 | enet | 0.039 | 6.50E-05 | -8.28E-01 | 4.08E-01 |
| CALB2 | 16 | 71392616 | 71424341 | 0.0496 | rs7500119 | -2.188 | rs2303257 | 0.023 | 3.750 | -0.716 | 114 | 3 | enet | 0.012 | 1.90E-02 | -8.27E-01 | 4.08E-01 |
| FLNC | 7 | 128470431 | 128499328 | 0.0827 | rs4728142 | 3.650 | rs7797420 | 0.003 | 3.630 | -0.079 | 96 | 30 | enet | 0.022 | 2.20E-03 | 8.26E-01 | 4.09E-01 |
| ANO6 | 12 | 45609770 | 45834187 | 0.1131 | rs11182857 | 2.900 | rs7977798 | 0.022 | -4.500 | 0.227 | 147 | 17 | enet | 0.028 | 6.50E-04 | 8.26E-01 | 4.09E-01 |
| MINA | 3 | 97660662 | 97691301 | 0.0673 | rs1492042 | 2.140 | rs1492030 | 0.086 | 4.830 | -1.698 | 107 | 8 | enet | 0.061 | 1.20E-04 | -8.24E-01 | 4.10E-01 |
| CASP9 | 1 | 15817327 | 15853029 | 0.1251 | rs16851662 | -2.395 | rs4233532 | 0.083 | 6.150 | -0.371 | 90 | 33 | enet | 0.140 | 1.80E-09 | -8.24E-01 | 4.10E-01 |
| MECR | 1 | 29519385 | 29557454 | 0.2984 | rs10799128 | -3.504 | rs547976 | 0.097 | 6.190 | -1.308 | 101 | 17 | enet | 0.140 | 3.50E-14 | -8.21E-01 | 4.11E-01 |
| TPRG1L | 1 | 3541566 | 3546691 | 0.0723 | rs6703610 | -3.097 | rs2821007 | 0.074 | -5.380 | 0.468 | 77 | 6 | enet | 0.061 | 7.30E-07 | -8.23E-01 | 4.11E-01 |
| NIT1 | 1 | 161087876 | 161095235 | 0.5796 | rs4559524 | 2.463 | rs1127525 | 0.295 | -10.730 | 1.282 | 97 | 21 | enet | 0.420 | 6.80E-46 | -8.22E-01 | 4.11E-01 |
| MTHFD1 | 14 | 64854749 | 64926722 | 0.0989 | rs8011839 | 2.530 | rs11158538 | 0.098 | -6.310 | 1.719 | 104 | 16 | enet | 0.130 | 7.20E-13 | -8.21E-01 | 4.11E-01 |
| ALDH7A1 | 5 | 125877533 | 125931110 | 0.1770 | rs4426911 | -2.514 | rs6870785 | 0.030 | 5.320 | -0.437 | 129 | 45 | enet | 0.098 | 3.00E-10 | -8.20E-01 | 4.12E-01 |
| SLC16A1 | 1 | 113454469 | 113499635 | 0.0460 | rs1216766 | 1.951 | rs1539624 | 0.033 | 4.230 | -0.938 | 56 | 3 | enet | 0.018 | 5.20E-03 | -8.19E-01 | 4.13E-01 |
| AGPAT5 | 8 | 6565878 | 6617184 | 0.0783 | rs11137053 | 2.640 | rs13281818 | 0.039 | -3.960 | -0.225 | 190 | 11 | enet | 0.040 | 5.50E-05 | 8.19E-01 | 4.13E-01 |
| CMC2 | 16 | 81009698 | 81053875 | 0.2191 | rs7188127 | -2.279 | rs9921914 | 0.303 | -11.270 | 0.304 | 147 | 13 | enet | 0.340 | 3.30E-36 | -8.17E-01 | 4.14E-01 |
| SPAG7 | 17 | 4862521 | 4871167 | 0.1226 | rs8078776 | -3.090 | rs16942615 | 0.117 | 6.740 | 0.855 | 92 | 4 | enet | 0.099 | 3.70E-10 | 8.16E-01 | 4.14E-01 |
| NCEH1 | 3 | 172348039 | 172429008 | 0.0426 | rs13093331 | -2.390 | rs6779265 | 0.001 | -3.290 | -0.046 | 145 | 22 | enet | 0.015 | 1.10E-02 | -8.16E-01 | 4.14E-01 |
| ADCK1 | 14 | 78266426 | 78401355 | 0.2701 | rs3861621 | -2.200 | rs734654 | 0.211 | -10.150 | 1.421 | 199 | 19 | enet | 0.300 | 3.70E-30 | -8.15E-01 | 4.15E-01 |
| OXNAD1 | 3 | 16306706 | 16391806 | 0.3263 | rs483682 | 4.070 | rs2292614 | 0.394 | -11.690 | -1.972 | 182 | 18 | enet | 0.460 | 6.80E-47 | 8.14E-01 | 4.16E-01 |
| SEC24C | 10 | 75504120 | 75531919 | 0.0331 | rs2675662 | -4.010 | rs10740418 | 0.025 | -3.720 | -1.238 | 56 | 10 | enet | 0.022 | 2.30E-03 | 8.12E-01 | 4.17E-01 |
| MRPL39 | 21 | 26957968 | 26979829 | 0.0516 | rs17407272 | -2.530 | rs2829867 | 0.000 | 3.400 | -0.215 | 84 | 9 | enet | -0.001 | 3.80E-01 | 8.11E-01 | 4.18E-01 |
| RUFY1 | 5 | 178977559 | 179037027 | 0.0922 | rs11249632 | -2.120 | rs4701140 | 0.033 | -4.550 | -0.130 | 100 | 8 | enet | 0.043 | 3.00E-05 | 8.09E-01 | 4.18E-01 |
| MYO6 | 6 | 76458909 | 76629254 | 0.2276 | rs2295935 | 1.636 | rs1280054 | 0.337 | -11.430 | 0.699 | 143 | 12 | enet | 0.340 | 2.10E-36 | -8.09E-01 | 4.19E-01 |
| GLS | 2 | 191745553 | 191830278 | 0.0314 | rs16833215 | -3.171 | rs3771308 | 0.013 | 4.090 | -1.745 | 106 | 14 | enet | 0.014 | 1.20E-02 | -8.07E-01 | 4.20E-01 |
| PGM3 | 6 | 83870869 | 83903655 | 0.1182 | rs9449589 | -1.558 | rs473267 | 0.225 | -9.370 | 0.899 | 60 | 15 | enet | 0.210 | 2.90E-21 | -8.04E-01 | 4.21E-01 |
| LMBRD1 | 6 | 70385694 | 70507003 | 0.0630 | rs2096060 | -2.603 | rs9185 | 0.055 | 5.850 | -0.960 | 132 | 7 | enet | 0.053 | 3.70E-06 | -8.04E-01 | 4.21E-01 |
| DECR2 | 16 | 451826 | 462487 | 0.1088 | rs1802752 | -2.400 | rs1698231 | 0.101 | 6.670 | -0.811 | 99 | 10 | enet | 0.110 | 2.70E-11 | -8.05E-01 | 4.21E-01 |
| STAM2 | 2 | 152973315 | 153032506 | 0.0831 | rs11883823 | 1.990 | rs4664089 | 0.088 | -6.690 | 0.464 | 82 | 10 | enet | 0.083 | 9.70E-09 | -8.04E-01 | 4.22E-01 |
| CD274 | 9 | 5450503 | 5470566 | 0.1703 | rs10975098 | -2.720 | rs1536926 | 0.172 | 7.120 | -0.569 | 142 | 8 | enet | 0.180 | 3.20E-13 | -8.02E-01 | 4.23E-01 |
| EHD4 | 15 | 42190950 | 42264776 | 0.1124 | rs2665208 | -2.790 | rs17739167 | 0.078 | -5.890 | 0.765 | 174 | 24 | enet | 0.068 | 1.60E-07 | -8.01E-01 | 4.23E-01 |
| GGPS1 | 1 | 235490665 | 235507847 | 0.0246 | rs12757197 | 4.190 | rs12035746 | 0.023 | 3.710 | -0.142 | 52 | 15 | enet | 0.011 | 2.50E-02 | 8.01E-01 | 4.23E-01 |
| TPD52L1 | 6 | 125440195 | 125585553 | 0.1361 | rs11154288 | 2.410 | rs3799732 | 0.059 | -6.540 | 0.976 | 179 | 18 | enet | 0.150 | 8.00E-15 | -7.97E-01 | 4.25E-01 |
| ANXA5 | 4 | 122589110 | 122618268 | 0.2848 | rs4283687 | 1.770 | rs2306420 | 0.338 | -11.440 | -0.332 | 124 | 26 | enet | 0.330 | 1.20E-34 | 7.97E-01 | 4.25E-01 |
| SNX32 | 11 | 65601112 | 65624367 | 0.2854 | rs624273 | -5.270 | rs2231884 | 0.222 | 9.680 | -1.616 | 87 | 29 | enet | 0.230 | 1.50E-23 | -7.96E-01 | 4.26E-01 |
| TRAF6 | 11 | 36508577 | 36531822 | 0.0430 | rs1365120 | 4.530 | rs5030482 | 0.058 | -4.980 | 0.760 | 118 | 8 | enet | 0.052 | 1.00E-05 | -7.95E-01 | 4.26E-01 |
| AARS | 16 | 70286198 | 70323446 | 0.0375 | rs8059245 | -1.588 | rs11642006 | 0.006 | -3.590 | 1.431 | 18 | 5 | enet | 0.014 | 1.20E-02 | -7.96E-01 | 4.26E-01 |

| | | | | | | | | | | | | | | | | | |
|---|---|---|---|---|---|---|---|---|---|---|---|---|---|---|---|---|---|
| HHATL | 3 | 42734155 | 42744319 | 0.2430 | rs17074669 | 2.570 | rs12635736 | 0.118 | -7.300 | 1.320 | 95 | 25 | enet | 0.180 | 3.20E-17 | -7.95E-01 | 4.26E-01 |
| PSMD5 | 9 | 123577774 | 123605262 | 0.0993 | rs1577001 | -1.380 | rs12343516 | 0.118 | 6.830 | 1.010 | 79 | 16 | enet | 0.110 | 2.30E-11 | 7.94E-01 | 4.27E-01 |
| GMPPB | 3 | 49754277 | 49761384 | 0.1884 | rs6446298 | -2.380 | rs6809879 | 0.226 | 9.310 | -0.440 | 80 | 10 | enet | 0.220 | 1.10E-22 | -7.92E-01 | 4.29E-01 |
| PAG1 | 8 | 81880045 | 82024303 | 0.0666 | rs7825859 | 3.110 | rs10958000 | 0.053 | 5.280 | -1.020 | 166 | 11 | enet | 0.021 | 2.90E-03 | -7.91E-01 | 4.29E-01 |
| CNTN5 | 11 | 98891683 | 100229616 | 0.2914 | rs12789951 | -3.940 | rs17660476 | 0.093 | -6.770 | 1.622 | 918 | 28 | enet | 0.130 | 8.10E-13 | -7.91E-01 | 4.29E-01 |
| TIPRL | 1 | 168148171 | 168169950 | 0.0924 | rs10800350 | -1.710 | rs10753766 | 0.092 | -6.410 | -0.874 | 87 | 12 | enet | 0.084 | 5.70E-09 | 7.90E-01 | 4.30E-01 |
| HIP1R | 12 | 123319000 | 123347507 | 0.1978 | rs2154 | 1.920 | rs11060180 | 0.077 | 5.510 | -0.380 | 65 | 11 | enet | 0.078 | 2.30E-08 | -7.89E-01 | 4.30E-01 |
| RCAN1 | 21 | 35885440 | 35987441 | 0.0867 | rs2243890 | 3.030 | rs13050712 | 0.004 | 3.900 | -1.162 | 175 | 19 | enet | 0.025 | 1.30E-03 | -7.88E-01 | 4.31E-01 |
| BLVRA | 7 | 43798279 | 43846939 | 0.1962 | rs2730610 | -2.760 | rs17246016 | 0.140 | -7.450 | 0.083 | 86 | 14 | enet | 0.190 | 1.20E-18 | -7.87E-01 | 4.31E-01 |
| CADPS2 | 7 | 121958481 | 122526813 | 0.0571 | rs4727939 | 3.460 | rs2189601 | 0.000 | -3.580 | 0.871 | 245 | 21 | enet | 0.000 | 3.50E-01 | -7.87E-01 | 4.31E-01 |
| TMEM38A | 19 | 16771938 | 16800840 | 0.0432 | rs754291 | 4.370 | rs7255269 | -0.001 | -3.770 | 1.093 | 67 | 11 | enet | -0.002 | 5.00E-01 | 7.85E-01 | 4.32E-01 |
| FXN | 9 | 71650175 | 71715094 | 0.2677 | rs9314845 | 2.750 | rs7857635 | 0.087 | -6.860 | 0.839 | 142 | 21 | enet | 0.210 | 6.00E-21 | -7.85E-01 | 4.33E-01 |
| CPT1C | 19 | 50194155 | 50216988 | 0.0583 | rs892149 | 2.400 | rs12981033 | 0.045 | -4.570 | 1.421 | 61 | 17 | enet | 0.019 | 4.10E-03 | -7.84E-01 | 4.33E-01 |
| VAPB | 20 | 56964178 | 57026157 | 0.0941 | rs17544336 | 2.438 | rs6026242 | 0.062 | 5.700 | 0.354 | 152 | 13 | enet | 0.096 | 5.10E-10 | 7.83E-01 | 4.34E-01 |
| SARS2 | 19 | 39405906 | 39440495 | 0.1075 | rs3136646 | -3.080 | rs4803027 | 0.029 | 4.630 | 1.053 | 104 | 38 | enet | 0.013 | 1.50E-02 | 7.79E-01 | 4.36E-01 |
| DAAM1 | 14 | 59655364 | 59838123 | 0.1606 | rs11626165 | -3.370 | rs1253035 | 0.016 | -4.980 | -0.960 | 218 | 15 | enet | 0.072 | 6.80E-08 | 7.78E-01 | 4.36E-01 |
| SEC23A | 14 | 39501123 | 39578850 | 0.1032 | rs17108797 | 2.980 | rs2277458 | 0.091 | 6.130 | -0.393 | 99 | 10 | enet | 0.088 | 2.70E-09 | -7.78E-01 | 4.36E-01 |
| LETMD1 | 12 | 51441745 | 51454207 | 0.0374 | rs2730648 | -2.450 | rs12582592 | 0.012 | -4.030 | -0.652 | 95 | 8 | enet | 0.018 | 5.30E-03 | 7.75E-01 | 4.38E-01 |
| USP8 | 15 | 50716577 | 50793280 | 0.0340 | rs12595633 | -2.420 | rs1060599 | 0.009 | -3.840 | 0.598 | 107 | 18 | enet | 0.008 | 4.50E-02 | -7.76E-01 | 4.38E-01 |
| ADH1B | 4 | 100226121 | 100242558 | 0.0658 | rs13145277 | -2.000 | rs4147545 | -0.002 | 3.310 | 1.195 | 137 | 9 | enet | 0.002 | 2.10E-01 | 7.75E-01 | 4.39E-01 |
| FAM114A2 | 5 | 153369688 | 153418496 | 0.1015 | rs4958354 | 1.467 | rs2560059 | 0.151 | -7.960 | 1.199 | 94 | 14 | enet | 0.150 | 5.40E-15 | -7.74E-01 | 4.39E-01 |
| TARS | 5 | 33440802 | 33469644 | 0.1035 | rs1595558 | 1.712 | rs3736393 | -0.003 | 3.460 | 1.264 | 91 | 16 | enet | 0.008 | 4.20E-02 | 7.74E-01 | 4.39E-01 |
| UHRF1BP1 | 6 | 34759857 | 34850915 | 0.1714 | rs3734266 | 1.673 | rs3734266 | 0.051 | 5.350 | 1.673 | 93 | 12 | enet | 0.099 | 6.40E-10 | 7.73E-01 | 4.40E-01 |
| PREP | 6 | 105725440 | 105850959 | 0.1075 | rs1210063 | 2.832 | rs717324 | 0.007 | -4.440 | -0.206 | 120 | 25 | enet | 0.024 | 1.50E-03 | -7.73E-01 | 4.40E-01 |
| ATIC | 2 | 216176540 | 216214487 | 0.3107 | rs4610054 | -2.956 | rs16853834 | 0.169 | 8.450 | 0.507 | 130 | 15 | enet | 0.220 | 7.70E-22 | 7.71E-01 | 4.41E-01 |
| EPDR1 | 7 | 37723446 | 37991543 | 0.1340 | rs17171184 | 2.700 | rs12671182 | 0.060 | 4.850 | 0.155 | 280 | 18 | enet | 0.049 | 8.20E-06 | 7.71E-01 | 4.41E-01 |
| CTSZ | 20 | 57570240 | 57582302 | 0.0829 | rs2021918 | 2.481 | rs9760 | 0.026 | -4.600 | 1.616 | 90 | 10 | enet | 0.014 | 1.30E-02 | -7.68E-01 | 4.42E-01 |
| STARD5 | 15 | 81601394 | 81616524 | 0.2170 | rs7162538 | 1.690 | rs4778893 | 0.159 | -8.110 | 0.611 | 97 | 15 | enet | 0.230 | 4.80E-21 | -7.69E-01 | 4.42E-01 |
| CTU2 | 16 | 88772871 | 88781794 | 0.1059 | rs3794624 | -3.594 | rs11076699 | 0.003 | -4.060 | 1.019 | 126 | 29 | enet | 0.000 | 2.90E-01 | -7.67E-01 | 4.43E-01 |
| SYNJ2 | 6 | 158402888 | 158520208 | 0.0825 | rs9295289 | -2.537 | rs756071 | 0.017 | -4.600 | 0.950 | 176 | 20 | enet | 0.048 | 1.60E-05 | -7.65E-01 | 4.44E-01 |
| ELP2 | 18 | 33709407 | 33757909 | 0.0748 | rs599457 | 1.880 | rs8299 | 0.090 | -6.210 | -0.176 | 131 | 25 | enet | 0.063 | 4.40E-07 | -7.65E-01 | 4.44E-01 |
| HIBCH | 2 | 191054461 | 191208919 | 0.5049 | rs7581986 | -2.646 | rs16832572 | 0.493 | -13.970 | 1.448 | 138 | 11 | enet | 0.590 | 8.10E-76 | -7.64E-01 | 4.45E-01 |
| EFEMP1 | 2 | 56093102 | 56151274 | 0.1121 | rs1346786 | -2.009 | rs11685113 | 0.047 | 5.240 | -0.328 | 134 | 21 | enet | 0.080 | 1.30E-08 | 7.63E-01 | 4.46E-01 |
| TRMT61B | 2 | 29072687 | 29093167 | 0.2494 | rs4233728 | 1.619 | rs4132617 | 0.327 | 11.110 | 0.829 | 74 | 12 | enet | 0.330 | 2.20E-34 | 7.62E-01 | 4.46E-01 |
| MRPS27 | 5 | 71515236 | 71616473 | 0.0398 | rs16876046 | -2.368 | rs10039706 | 0.031 | 4.210 | -0.717 | 113 | 5 | enet | 0.020 | 3.70E-03 | -7.60E-01 | 4.47E-01 |
| AADAT | 4 | 170981373 | 171012850 | 0.0319 | rs34752946 | -1.260 | rs7694808 | 0.012 | -3.600 | 0.173 | 101 | 9 | enet | 0.015 | 1.80E-02 | -7.59E-01 | 4.48E-01 |
| SEC23IP | 10 | 121652223 | 121702014 | 0.2800 | rs10788015 | -2.010 | rs2901218 | 0.243 | -9.700 | 1.024 | 103 | 21 | enet | 0.290 | 1.70E-30 | -7.57E-01 | 4.49E-01 |
| DNLZ | 9 | 139253932 | 139258241 | 0.1922 | rs12684275 | 1.100 | rs11145958 | 0.085 | -5.070 | -0.248 | 71 | 12 | enet | 0.067 | 4.80E-06 | 7.57E-01 | 4.49E-01 |
| PTPLB | 3 | 123209667 | 123304032 | 0.0524 | rs16834365 | -2.350 | rs820444 | 0.041 | 4.900 | 0.699 | 79 | 5 | enet | 0.034 | 2.00E-04 | 7.57E-01 | 4.49E-01 |
| COA4 | 11 | 73583712 | 73588033 | 0.0505 | rs1792174 | -2.230 | rs1792163 | 0.006 | -3.810 | -1.068 | 76 | 11 | enet | 0.004 | 1.30E-01 | 7.54E-01 | 4.51E-01 |
| DBT | 1 | 100652475 | 100715390 | 0.0847 | rs617153 | -2.652 | rs2810424 | 0.121 | -6.910 | -0.094 | 90 | 18 | enet | 0.100 | 1.80E-10 | -7.52E-01 | 4.52E-01 |
| ACTN1 | 14 | 69340860 | 69446157 | 0.0534 | rs4902647 | -6.330 | rs735986 | 0.014 | 4.100 | 1.389 | 186 | 10 | enet | 0.021 | 2.90E-03 | 7.52E-01 | 4.52E-01 |
| GPR37L1 | 1 | 202091986 | 202102720 | 0.0755 | rs3934648 | 2.492 | rs11588918 | 0.050 | 5.070 | -0.662 | 105 | 12 | enet | 0.051 | 6.20E-06 | -7.50E-01 | 4.53E-01 |
| BDH2 | 4 | 104000592 | 104021040 | 0.4613 | rs7685399 | 3.950 | rs6830624 | 0.113 | 6.690 | -0.927 | 87 | 10 | enet | 0.160 | 3.80E-16 | -7.49E-01 | 4.54E-01 |
| LYSMD1 | 1 | 151132224 | 151138424 | 0.0575 | rs11204787 | 2.200 | rs12089658 | 0.008 | -3.540 | -0.371 | 57 | 8 | enet | 0.013 | 1.60E-02 | 7.49E-01 | 4.54E-01 |
| CTNND1 | 11 | 57520715 | 57587018 | 0.0563 | rs11570210 | -1.080 | rs499188 | 0.049 | -4.630 | -0.771 | 66 | 7 | enet | 0.033 | 2.30E-04 | 7.49E-01 | 4.54E-01 |

| | | | | | | | | | | | | | | | | | |
|---|---|---|---|---|---|---|---|---|---|---|---|---|---|---|---|---|---|
| RNPEP | 1 | 201951500 | 201975275 | 0.2665 | rs7529387 | 2.405 | rs16849483 | 0.173 | -8.130 | -0.848 | 96 | 35 | enet | 0.210 | 6.30E-21 | -7.45E-01 | 4.56E-01 |
| LCLAT1 | 2 | 30670092 | 30867091 | 0.2263 | rs482528 | 3.060 | rs2602788 | 0.183 | -8.230 | -1.181 | 217 | 19 | enet | 0.200 | 3.10E-19 | 7.46E-01 | 4.56E-01 |
| PLEKHA1 | 10 | 124134212 | 124191867 | 0.0418 | rs7909454 | -2.920 | rs10510110 | 0.040 | -4.270 | 0.046 | 181 | 6 | enet | 0.009 | 3.40E-02 | -7.45E-01 | 4.57E-01 |
| PON2 | 7 | 95034175 | 95064510 | 0.2812 | rs6978425 | 2.110 | rs7785846 | 0.379 | -12.200 | 0.615 | 142 | 21 | enet | 0.390 | 2.30E-42 | -7.44E-01 | 4.57E-01 |
| AK5 | 1 | 77747736 | 78025651 | 0.1215 | rs6695572 | 2.561 | rs728741 | 0.105 | 6.910 | -0.420 | 265 | 22 | enet | 0.140 | 1.60E-14 | -7.44E-01 | 4.57E-01 |
| FARS2 | 6 | 5261277 | 5771813 | 0.1466 | rs9405272 | 2.372 | rs9504370 | 0.004 | -4.720 | 0.630 | 411 | 20 | enet | 0.013 | 1.60E-02 | -7.44E-01 | 4.57E-01 |
| DCUN1D2 | 13 | 114110134 | 114145267 | 0.0534 | rs9549757 | -2.330 | rs6577052 | 0.019 | -4.260 | 0.777 | 123 | 7 | enet | 0.024 | 3.20E-03 | -7.43E-01 | 4.58E-01 |
| C10orf32 | 10 | 104613980 | 104624718 | 0.0749 | rs284859 | -2.520 | rs9527 | 0.109 | -6.970 | 0.507 | 85 | 15 | enet | 0.085 | 5.30E-09 | -7.38E-01 | 4.61E-01 |
| HINT1 | 5 | 130494720 | 130507428 | 0.0641 | rs6875363 | 3.491 | rs1422661 | 0.076 | 5.870 | -0.681 | 71 | 10 | enet | 0.077 | 2.80E-08 | -7.37E-01 | 4.61E-01 |
| GSTO1 | 10 | 105995114 | 106027217 | 0.8144 | rs12769490 | -1.730 | rs11509438 | 0.653 | -15.640 | 0.692 | 94 | 24 | enet | 0.680 | 9.90E-95 | -7.37E-01 | 4.61E-01 |
| CCDC93 | 2 | 118673054 | 118771709 | 0.0678 | rs17584842 | -2.723 | rs2303331 | 0.049 | 4.720 | -1.412 | 134 | 10 | enet | 0.032 | 2.60E-04 | -7.36E-01 | 4.62E-01 |
| SWAP70 | 11 | 9685624 | 9774538 | 0.1370 | rs2289074 | -1.840 | rs373894 | 0.089 | -6.150 | -1.474 | 133 | 39 | enet | 0.100 | 1.10E-10 | 7.36E-01 | 4.62E-01 |
| PLOD2 | 3 | 145787227 | 145881440 | 0.0766 | rs9838866 | 1.410 | rs1449453 | 0.036 | -3.500 | -0.199 | 122 | 8 | enet | 0.063 | 1.50E-04 | 7.35E-01 | 4.62E-01 |
| PLGRKT | 9 | 5357973 | 5437878 | 0.2052 | rs2381215 | 3.360 | rs2890658 | 0.026 | -5.450 | -0.141 | 169 | 17 | enet | 0.140 | 5.70E-14 | 7.33E-01 | 4.63E-01 |
| RASGRF1 | 15 | 79252289 | 79383115 | 0.0321 | rs16970344 | -4.650 | rs12595749 | 0.024 | -4.000 | -0.098 | 164 | 11 | enet | 0.012 | 2.00E-02 | 7.33E-01 | 4.64E-01 |
| ITGAM | 16 | 31271311 | 31344213 | 0.0254 | rs9888739 | 4.887 | rs2359661 | 0.030 | -4.430 | 1.950 | 76 | 8 | enet | 0.039 | 6.90E-05 | -7.32E-01 | 4.64E-01 |
| FSD1 | 19 | 4304597 | 4323840 | 0.0779 | rs12462529 | -3.690 | rs8102860 | 0.019 | 4.250 | 1.230 | 82 | 18 | enet | 0.024 | 1.30E-03 | 7.32E-01 | 4.64E-01 |
| C1orf123 | 1 | 53679771 | 53686289 | 0.0968 | rs1288502 | 1.880 | rs2297656 | 0.156 | -7.770 | 0.723 | 109 | 12 | enet | 0.140 | 2.00E-14 | -7.31E-01 | 4.65E-01 |
| LACTB2 | 8 | 71547553 | 71581409 | 0.0996 | rs9886439 | -3.870 | rs2290702 | 0.134 | 7.220 | 1.051 | 67 | 8 | enet | 0.140 | 1.30E-14 | 7.29E-01 | 4.66E-01 |
| GPX1 | 3 | 49394609 | 49396033 | 0.4229 | rs9874474 | 1.870 | rs17080528 | 0.485 | -13.550 | 0.464 | 52 | 14 | enet | 0.490 | 5.50E-57 | -7.28E-01 | 4.67E-01 |
| KIAA1279 | 10 | 70748487 | 70776738 | 0.1642 | rs2429014 | -2.900 | rs2487707 | 0.170 | -8.320 | -0.432 | 93 | 8 | enet | 0.180 | 1.80E-18 | 7.28E-01 | 4.67E-01 |
| NHLRC2 | 10 | 115614420 | 115676953 | 0.0734 | rs7895609 | -2.310 | rs7913176 | 0.060 | -5.640 | 0.599 | 129 | 4 | enet | 0.040 | 5.60E-05 | -7.26E-01 | 4.68E-01 |
| SCCPDH | 1 | 246887349 | 246931439 | 0.2100 | rs6694274 | 3.006 | rs4926440 | 0.079 | -5.980 | -0.260 | 126 | 41 | enet | 0.130 | 1.20E-13 | -7.25E-01 | 4.68E-01 |
| IBA57 | 1 | 228353516 | 228369958 | 0.0331 | rs1188977 | -3.105 | rs3795782 | 0.016 | -4.140 | -1.736 | 68 | 17 | enet | 0.007 | 5.80E-02 | 7.26E-01 | 4.68E-01 |
| FAM47E-STBD1 | 4 | 77172886 | 77232752 | 0.0992 | rs6849069 | 2.370 | rs3733248 | 0.006 | -3.800 | 1.502 | 123 | 9 | enet | 0.017 | 7.60E-03 | -7.25E-01 | 4.69E-01 |
| LYN | 8 | 56792372 | 56923940 | 0.0723 | rs952784 | 2.360 | rs13277429 | 0.037 | 4.660 | 0.782 | 134 | 16 | enet | 0.025 | 1.20E-03 | 7.23E-01 | 4.69E-01 |
| LACTB | 15 | 63413999 | 63434260 | 0.2182 | rs17753220 | 2.270 | rs2729835 | 0.296 | -10.690 | 0.548 | 97 | 14 | enet | 0.320 | 5.10E-33 | -7.22E-01 | 4.70E-01 |
| ADCY3 | 2 | 25042038 | 25142708 | 0.0744 | rs1550115 | 3.916 | rs6726261 | 0.033 | -4.190 | 0.398 | 121 | 16 | enet | 0.014 | 1.40E-02 | -7.21E-01 | 4.71E-01 |
| GNG8 | 19 | 47137333 | 47137942 | 0.3763 | rs4803983 | 2.600 | rs3760759 | 0.131 | -6.510 | 0.309 | 85 | 17 | enet | 0.250 | 7.30E-22 | -7.20E-01 | 4.72E-01 |
| MRE11A | 11 | 94152895 | 94227074 | 0.2438 | rs7945777 | -3.160 | rs535801 | 0.056 | 7.400 | -0.425 | 141 | 27 | enet | 0.170 | 3.30E-17 | -7.19E-01 | 4.72E-01 |
| C12orf45 | 12 | 105380088 | 105443515 | 0.1120 | rs7134351 | 1.790 | rs7308568 | 0.040 | -5.920 | -0.040 | 114 | 13 | enet | 0.100 | 3.10E-09 | -7.20E-01 | 4.72E-01 |
| GTPBP10 | 7 | 89964537 | 90020769 | 0.2861 | rs12704539 | 2.740 | rs42659 | 0.320 | 10.830 | -0.075 | 116 | 14 | enet | 0.390 | 1.20E-40 | -7.19E-01 | 4.72E-01 |
| SYNM | 15 | 99638420 | 99675798 | 0.1069 | rs2623191 | -2.380 | rs1703770 | 0.052 | -5.870 | 1.093 | 164 | 17 | enet | 0.085 | 4.40E-09 | -7.18E-01 | 4.73E-01 |
| XYLB | 3 | 38388251 | 38462839 | 0.0451 | rs2070491 | 2.490 | rs196375 | 0.004 | -3.820 | 0.620 | 131 | 10 | enet | 0.017 | 2.40E-02 | -7.16E-01 | 4.74E-01 |
| PIK3CD | 1 | 9711790 | 9789172 | 0.0659 | rs3934934 | -2.167 | rs4240895 | 0.031 | 4.660 | -0.920 | 87 | 4 | enet | 0.040 | 9.80E-05 | -7.15E-01 | 4.74E-01 |
| CNPY2 | 12 | 56703626 | 56710120 | 0.0658 | rs4759218 | -2.460 | rs2306693 | 0.114 | 7.050 | -0.771 | 41 | 20 | enet | 0.094 | 1.10E-09 | -7.17E-01 | 4.74E-01 |
| MSRA | 8 | 9911778 | 10286401 | 0.1834 | rs17151244 | 2.540 | rs12675153 | -0.002 | 3.500 | -1.772 | 438 | 39 | enet | 0.018 | 5.70E-03 | -7.14E-01 | 4.75E-01 |
| NUDT16 | 3 | 131100515 | 131107674 | 0.2761 | rs17334851 | 1.970 | rs16836573 | 0.122 | -7.250 | 0.514 | 69 | 22 | enet | 0.130 | 3.90E-13 | -7.13E-01 | 4.76E-01 |
| WDFY1 | 2 | 224720433 | 224810104 | 0.0728 | rs2037755 | -1.912 | rs16865390 | 0.018 | 4.340 | -0.203 | 140 | 37 | enet | 0.037 | 1.00E-04 | 7.13E-01 | 4.76E-01 |
| UGT1A6 | 2 | 234600253 | 234681946 | 0.0834 | rs1823803 | -2.343 | rs11674078 | 0.001 | -3.150 | -0.925 | 317 | 22 | enet | 0.000 | 3.00E-01 | 7.12E-01 | 4.76E-01 |
| GM2A | 5 | 150591711 | 150650001 | 0.0484 | rs1004595 | 2.937 | rs152266 | 0.010 | 4.140 | 0.627 | 184 | 15 | enet | 0.027 | 7.80E-04 | 7.12E-01 | 4.76E-01 |
| AMPD3 | 11 | 10329860 | 10529126 | 0.1743 | rs11042727 | -3.150 | rs11604833 | 0.137 | 7.280 | -1.207 | 216 | 25 | enet | 0.140 | 8.90E-14 | -7.13E-01 | 4.76E-01 |
| NAPA | 19 | 47990894 | 48018497 | 0.2056 | rs4802352 | 2.450 | rs2974238 | 0.027 | 4.990 | -0.042 | 81 | 16 | enet | 0.080 | 1.30E-08 | 7.11E-01 | 4.77E-01 |
| CHN2 | 7 | 29161890 | 29553944 | 0.1233 | rs2286992 | 2.480 | rs1016726 | 0.000 | 3.620 | 0.211 | 470 | 33 | enet | 0.027 | 1.10E-03 | 7.10E-01 | 4.78E-01 |
| MYOM2 | 8 | 1993155 | 2113475 | 0.6377 | rs4876225 | -3.090 | rs2280817 | 0.307 | 8.620 | 1.075 | 287 | 26 | enet | 0.450 | 3.70E-28 | 7.07E-01 | 4.79E-01 |

| | | | | | | | | | | | | | | | | | |
|---|---|---|---|---|---|---|---|---|---|---|---|---|---|---|---|---|---|
| PAFAH1B2 | 11 | 117014983 | 117047610 | 0.0468 | rs17120293 | -2.650 | rs6589597 | 0.011 | -4.190 | 1.491 | 86 | 12 | enet | 0.010 | 2.80E-02 | -7.08E-01 | 4.79E-01 |
| DHRS1 | 14 | 24759804 | 24769039 | 0.7218 | rs11846223 | -2.880 | rs10134537 | 0.189 | -8.610 | 0.318 | 120 | 31 | enet | 0.530 | 2.20E-62 | 7.09E-01 | 4.79E-01 |
| PLXNA1 | 3 | 126707437 | 126756235 | 0.1232 | rs7633288 | -2.500 | rs1008175 | 0.037 | -3.960 | 0.847 | 130 | 21 | enet | 0.030 | 4.00E-04 | -7.08E-01 | 4.79E-01 |
| LRRFIP1 | 2 | 238536219 | 238722325 | 0.1139 | rs6725315 | 2.554 | rs734388 | 0.047 | -5.150 | 0.266 | 222 | 25 | enet | 0.047 | 1.30E-05 | -7.07E-01 | 4.80E-01 |
| C8orf47 | 8 | 99076539 | 99105838 | 0.1079 | rs2514344 | -2.920 | rs10098159 | 0.036 | 4.520 | 0.037 | 141 | 28 | enet | 0.077 | 3.80E-07 | 7.06E-01 | 4.80E-01 |
| BCKDHA | 19 | 41884215 | 41930910 | 0.2086 | rs8109627 | -3.490 | rs4803464 | 0.031 | 3.730 | -1.155 | 74 | 14 | enet | 0.062 | 5.30E-07 | 7.05E-01 | 4.81E-01 |
| MAN2C1 | 15 | 75648133 | 75660971 | 0.4343 | rs8028182 | -1.710 | rs8031650 | 0.121 | -7.550 | 1.002 | 30 | 8 | enet | 0.150 | 3.60E-15 | -7.05E-01 | 4.81E-01 |
| PPL | 16 | 4932508 | 5010742 | 0.0842 | rs17704487 | -2.872 | rs2939960 | 0.005 | -4.120 | -0.650 | 147 | 10 | enet | 0.029 | 9.60E-04 | 7.03E-01 | 4.82E-01 |
| ACSL1 | 4 | 185676749 | 185747972 | 0.1188 | rs1996546 | -3.460 | rs1554336 | 0.037 | -5.200 | -0.723 | 153 | 14 | enet | 0.062 | 8.80E-07 | 6.99E-01 | 4.85E-01 |
| QDPR | 4 | 17461884 | 17513857 | 0.0493 | rs4698184 | 2.210 | rs1868733 | -0.003 | -3.000 | -1.179 | 126 | 21 | enet | -0.001 | 4.80E-01 | 6.98E-01 | 4.85E-01 |
| SCRN1 | 7 | 29959719 | 30029905 | 0.2745 | rs174929 | 1.270 | rs17324153 | 0.032 | -5.150 | 0.485 | 117 | 29 | enet | 0.092 | 9.90E-10 | -6.97E-01 | 4.86E-01 |
| IGFBP7 | 4 | 57896939 | 57976551 | 0.0645 | rs11930475 | 3.120 | rs1714004 | 0.068 | -5.380 | 0.883 | 179 | 10 | enet | 0.062 | 8.40E-07 | -6.96E-01 | 4.87E-01 |
| CAPN5 | 11 | 76777979 | 76837201 | 0.1013 | rs7943716 | -2.600 | rs7926995 | 0.030 | 4.140 | 0.080 | 128 | 18 | enet | 0.036 | 1.10E-04 | -6.95E-01 | 4.87E-01 |
| PYROXD1 | 12 | 21590549 | 21623300 | 0.0776 | rs2192170 | 2.360 | rs2058463 | 0.000 | -3.210 | 0.989 | 173 | 19 | enet | 0.019 | 1.20E-02 | -6.95E-01 | 4.87E-01 |
| STX8 | 17 | 9153788 | 9479908 | 0.0753 | rs7224675 | 2.540 | rs6503202 | 0.000 | -3.140 | -0.673 | 262 | 40 | enet | 0.001 | 2.50E-01 | -6.96E-01 | 4.87E-01 |
| TBC1D10A | 22 | 30687979 | 30723035 | 0.0422 | rs9608859 | 3.580 | rs724055 | 0.015 | 3.670 | 0.544 | 129 | 19 | enet | 0.003 | 1.60E-01 | 6.93E-01 | 4.89E-01 |
| PYGM | 11 | 64513861 | 64527769 | 0.1662 | rs489192 | 1.840 | rs2073798 | 0.082 | -6.520 | 1.532 | 66 | 22 | enet | 0.120 | 4.00E-12 | -6.92E-01 | 4.89E-01 |
| ASL | 7 | 65540785 | 65558545 | 0.2225 | rs7811214 | 1.220 | rs908915 | 0.245 | -10.170 | 0.575 | 53 | 18 | enet | 0.290 | 2.80E-30 | -6.91E-01 | 4.89E-01 |
| FAAH | 1 | 46859937 | 46879520 | 0.2731 | rs17361749 | -2.899 | rs324420 | 0.235 | -9.510 | 0.483 | 80 | 20 | enet | 0.270 | 5.80E-27 | 6.91E-01 | 4.90E-01 |
| YOD1 | 1 | 207217194 | 207226325 | 0.0526 | rs12061578 | 2.876 | rs2883211 | 0.057 | -5.240 | -0.500 | 90 | 14 | enet | 0.067 | 2.80E-07 | 6.91E-01 | 4.90E-01 |
| NQO2 | 6 | 2988221 | 3019996 | 0.5860 | rs7743193 | -2.452 | rs17300141 | 0.207 | 10.390 | -0.072 | 134 | 22 | enet | 0.380 | 1.40E-41 | 6.91E-01 | 4.90E-01 |
| CDC16 | 13 | 115000362 | 115038198 | 0.0393 | rs7999630 | 1.830 | rs7994556 | 0.032 | -4.970 | 0.988 | 84 | 11 | enet | 0.050 | 7.60E-06 | -6.90E-01 | 4.90E-01 |
| UCHL3 | 13 | 76123619 | 76180085 | 0.2077 | rs2147006 | -1.500 | rs7982613 | 0.221 | 9.390 | -0.897 | 112 | 18 | enet | 0.220 | 1.80E-22 | -6.90E-01 | 4.91E-01 |
| MON1A | 3 | 49946302 | 49967606 | 0.0201 | rs6446298 | -2.380 | rs17657664 | 0.000 | -3.400 | 0.604 | 66 | 6 | enet | 0.000 | 2.90E-01 | -6.87E-01 | 4.92E-01 |
| SH3BGRL3 | 1 | 26605667 | 26608007 | 0.0539 | rs6660744 | 1.272 | rs4659419 | 0.001 | 3.740 | -0.063 | 124 | 18 | enet | 0.024 | 1.50E-03 | -6.85E-01 | 4.93E-01 |
| IAH1 | 2 | 9613787 | 9636672 | 0.5822 | rs11690126 | -2.446 | rs10593 | 0.061 | -5.740 | -1.384 | 61 | 25 | enet | 0.240 | 2.20E-24 | -6.85E-01 | 4.93E-01 |
| MRI1 | 19 | 13875346 | 13885096 | 0.3984 | rs8106403 | 1.340 | rs371671 | 0.310 | -10.740 | -0.699 | 54 | 5 | enet | 0.300 | 8.70E-31 | 6.84E-01 | 4.94E-01 |
| DLGAP2 | 8 | 1449532 | 1656642 | 0.0753 | rs13280292 | 2.880 | rs2301963 | 0.086 | -5.860 | -0.967 | 225 | 7 | enet | 0.065 | 3.40E-07 | 6.84E-01 | 4.94E-01 |
| MIF | 22 | 24236191 | 24237414 | 0.1146 | rs5760060 | 2.190 | rs5751759 | 0.083 | -5.650 | 0.844 | 112 | 7 | enet | 0.082 | 1.80E-08 | -6.82E-01 | 4.95E-01 |
| ARL3 | 10 | 104433488 | 104474164 | 0.0965 | rs284859 | -2.520 | rs2298278 | 0.057 | -5.220 | 0.390 | 100 | 15 | enet | 0.047 | 1.30E-05 | -6.81E-01 | 4.96E-01 |
| H1F0 | 22 | 38201114 | 38203442 | 0.0320 | rs2285179 | -2.320 | rs5756825 | 0.001 | 3.220 | -1.517 | 58 | 18 | enet | -0.001 | 4.90E-01 | -6.80E-01 | 4.96E-01 |
| TBC1D1 | 4 | 37892708 | 38140796 | 0.2164 | rs7671645 | 3.410 | rs12511756 | 0.094 | 6.810 | -0.061 | 289 | 23 | enet | 0.140 | 1.90E-14 | -6.78E-01 | 4.98E-01 |
| FOCAD | 9 | 20658308 | 20995954 | 0.0641 | rs17685984 | -2.140 | rs7041474 | 0.012 | -4.050 | 0.427 | 266 | 9 | enet | 0.013 | 1.50E-02 | -6.73E-01 | 5.01E-01 |
| GRID1 | 10 | 87359312 | 88126250 | 0.2079 | rs1112487 | 2.730 | rs11528413 | 0.140 | -7.950 | 0.166 | 459 | 28 | enet | 0.200 | 1.10E-19 | -6.71E-01 | 5.02E-01 |
| NUDT2 | 9 | 34329504 | 34343709 | 0.3506 | rs4256660 | 3.530 | rs4879775 | 0.201 | 8.760 | -1.034 | 76 | 23 | enet | 0.350 | 8.00E-37 | 6.67E-01 | 5.05E-01 |
| MTMR7 | 8 | 17155539 | 17271037 | 0.0558 | rs2285284 | 2.840 | rs2239867 | 0.004 | -3.840 | 1.017 | 234 | 5 | enet | 0.001 | 2.40E-01 | -6.67E-01 | 5.05E-01 |
| TRPT1 | 11 | 63991271 | 63993726 | 0.1001 | rs2510066 | -4.020 | rs1544538 | 0.079 | 4.640 | 0.422 | 68 | 9 | enet | 0.070 | 1.40E-05 | 6.65E-01 | 5.06E-01 |
| TNS3 | 7 | 47314752 | 47622156 | 0.0696 | rs2462626 | -2.190 | rs6959554 | 0.063 | 5.160 | 0.622 | 255 | 8 | enet | 0.033 | 2.80E-04 | 6.65E-01 | 5.06E-01 |
| PIH1D1 | 19 | 49949555 | 49956754 | 0.0752 | rs4802597 | 6.470 | rs1578944 | 0.034 | -4.910 | -1.530 | 78 | 17 | enet | 0.042 | 6.50E-05 | 6.64E-01 | 5.06E-01 |
| SNX6 | 14 | 35030300 | 35099389 | 0.0708 | rs4304943 | 2.250 | rs7161203 | 0.067 | -5.820 | -0.314 | 95 | 16 | enet | 0.083 | 7.80E-09 | 6.64E-01 | 5.07E-01 |
| PALM2 | 9 | 112403068 | 112713755 | 0.0854 | rs913359 | -2.750 | rs17202329 | 0.053 | 4.940 | -1.046 | 321 | 31 | enet | 0.050 | 6.90E-06 | -6.62E-01 | 5.08E-01 |
| EXOC6B | 2 | 72403113 | 73053170 | 0.0431 | rs12613322 | 2.639 | rs4852883 | 0.002 | 4.090 | -0.654 | 212 | 15 | enet | 0.017 | 6.20E-03 | -6.62E-01 | 5.08E-01 |
| WDR92 | 2 | 68350068 | 68384692 | 0.0312 | rs12465425 | 3.712 | rs13009282 | 0.041 | -4.400 | -0.698 | 75 | 10 | enet | 0.031 | 5.60E-04 | 6.62E-01 | 5.08E-01 |
| C3orf33 | 3 | 155480401 | 155524140 | 0.3405 | rs9850228 | -1.550 | rs358733 | 0.380 | -12.420 | 0.777 | 96 | 31 | enet | 0.410 | 4.70E-44 | -6.62E-01 | 5.08E-01 |
| SLC2A11 | 22 | 24198890 | 24228496 | 0.3469 | rs5760060 | 2.190 | rs875643 | 0.178 | -7.970 | 0.594 | 125 | 30 | enet | 0.250 | 2.80E-23 | 6.59E-01 | 5.10E-01 |
| SULT1A1 | 16 | 28616903 | 28634946 | 0.3611 | rs13336311 | 3.100 | rs1074631 | 0.195 | -8.940 | -0.396 | 45 | 15 | enet | 0.270 | 1.60E-27 | 6.58E-01 | 5.10E-01 |

| | | | | | | | | | | | | | | | | | |
|---|---|---|---|---|---|---|---|---|---|---|---|---|---|---|---|---|---|
| BLOC1S2 | 10 | 102033713 | 102046469 | 0.0429 | rs677603 | 2.080 | rs6584356 | 0.061 | -5.240 | 0.704 | 94 | 6 | enet | 0.041 | 4.60E-05 | -6.57E-01 | 5.11E-01 |
| ACSS3 | 12 | 81331594 | 81650533 | 0.0987 | rs3782165 | -2.190 | rs10778793 | 0.068 | 5.180 | 0.044 | 220 | 12 | enet | 0.098 | 2.90E-10 | 6.56E-01 | 5.12E-01 |
| GSTZ1 | 14 | 77787227 | 77797940 | 0.3783 | rs4899651 | -2.200 | rs2111699 | 0.510 | 13.870 | -0.435 | 128 | 15 | enet | 0.530 | 4.00E-63 | -6.54E-01 | 5.13E-01 |
| SRI | 7 | 87834433 | 87856308 | 0.0489 | rs11760375 | 1.750 | rs17150369 | 0.041 | -4.040 | 1.345 | 65 | 10 | enet | 0.028 | 6.20E-04 | -6.53E-01 | 5.14E-01 |
| NUDT6 | 4 | 123809852 | 123844123 | 0.3775 | rs11944526 | 2.140 | rs12648093 | 0.493 | -12.610 | 0.667 | 90 | 16 | enet | 0.550 | 1.60E-57 | -6.53E-01 | 5.14E-01 |
| PCYOX1L | 5 | 148737570 | 148749216 | 0.1328 | rs353293 | 2.222 | rs13160429 | 0.079 | -6.100 | -0.330 | 126 | 22 | enet | 0.130 | 2.50E-13 | -6.52E-01 | 5.14E-01 |
| CPM | 12 | 69235977 | 69365350 | 0.0790 | rs10784750 | 2.180 | rs17106030 | 0.042 | -4.820 | -0.341 | 157 | 15 | enet | 0.041 | 4.60E-05 | 6.53E-01 | 5.14E-01 |
| SRR | 17 | 2206677 | 2228554 | 0.2110 | rs2005029 | -4.380 | rs12450028 | 0.296 | -10.650 | -0.626 | 112 | 12 | enet | 0.290 | 8.70E-30 | 6.49E-01 | 5.16E-01 |
| CCDC109B | 4 | 110481361 | 110609874 | 0.1824 | rs6533440 | 1.980 | rs6832174 | -0.005 | 3.020 | 1.651 | 111 | 19 | enet | 0.014 | 4.60E-02 | 6.48E-01 | 5.17E-01 |
| HEXDC | 17 | 80376194 | 80400521 | 0.2368 | rs9894993 | 2.800 | rs1141463 | 0.058 | 4.230 | -0.157 | 88 | 20 | enet | 0.048 | 3.20E-04 | -6.47E-01 | 5.17E-01 |
| NIPSNAP1 | 22 | 29950797 | 29977326 | 0.0435 | rs13055076 | 3.360 | rs2857645 | 0.040 | 4.730 | -1.703 | 80 | 15 | enet | 0.038 | 7.60E-05 | -6.46E-01 | 5.18E-01 |
| MFF | 2 | 228189867 | 228222550 | 0.1903 | rs10198887 | -2.339 | rs7560053 | 0.122 | 7.350 | 1.630 | 108 | 20 | enet | 0.120 | 2.80E-12 | 6.43E-01 | 5.20E-01 |
| DCTD | 4 | 183811213 | 183839089 | 0.1058 | rs11132155 | -2.470 | rs2714545 | 0.021 | -4.340 | -0.202 | 128 | 23 | enet | 0.024 | 2.00E-03 | 6.43E-01 | 5.20E-01 |
| TAMM41 | 3 | 11831916 | 11888393 | 0.3420 | rs11923454 | -2.150 | rs402731 | 0.368 | -11.720 | -0.794 | 139 | 20 | enet | 0.440 | 8.10E-48 | 6.40E-01 | 5.22E-01 |
| RAF1 | 3 | 12625100 | 12705725 | 0.0828 | rs1532534 | 2.420 | rs1051208 | 0.042 | 4.350 | 0.414 | 96 | 5 | enet | 0.032 | 3.60E-04 | 6.40E-01 | 5.22E-01 |
| PDCD6IP | 3 | 33839844 | 33911194 | 0.0360 | rs1920052 | 1.860 | rs9843488 | 0.003 | -3.380 | 0.639 | 88 | 6 | enet | -0.003 | 9.20E-01 | -6.39E-01 | 5.23E-01 |
| TMED5 | 1 | 93615299 | 93646285 | 0.0460 | rs575367 | -2.933 | rs1060622 | 0.063 | 4.920 | 0.709 | 74 | 13 | enet | 0.055 | 5.30E-06 | 6.38E-01 | 5.24E-01 |
| TMCC2 | 1 | 205197304 | 205242471 | 0.0620 | rs4950984 | -1.905 | rs12144980 | 0.065 | -5.640 | 0.346 | 123 | 10 | enet | 0.055 | 2.30E-06 | -6.37E-01 | 5.24E-01 |
| NUDCD2 | 5 | 162873532 | 162887146 | 0.0356 | rs10040287 | -2.034 | rs213505 | 0.045 | 5.130 | 0.853 | 103 | 11 | enet | 0.042 | 3.70E-05 | 6.37E-01 | 5.24E-01 |
| SCRG1 | 4 | 174305852 | 174327531 | 0.2308 | rs4696055 | -1.940 | rs1992466 | 0.209 | 9.060 | -0.909 | 58 | 11 | enet | 0.220 | 3.10E-22 | -6.37E-01 | 5.25E-01 |
| TBCD | 17 | 80709940 | 80900724 | 0.3042 | rs2292968 | -2.080 | rs729123 | 0.110 | 8.600 | -0.105 | 187 | 26 | enet | 0.330 | 1.50E-34 | 6.36E-01 | 5.25E-01 |
| HSD17B7 | 1 | 162760492 | 162782607 | 0.0795 | rs16844730 | 2.184 | rs1704745 | 0.050 | 3.930 | -0.495 | 142 | 34 | enet | 0.018 | 1.30E-02 | -6.33E-01 | 5.26E-01 |
| SLC17A6 | 11 | 22359643 | 22401049 | 0.0655 | rs11026477 | -2.390 | rs11026523 | 0.045 | -4.840 | 0.633 | 96 | 14 | enet | 0.029 | 5.70E-04 | -6.33E-01 | 5.27E-01 |
| CHL1 | 3 | 238279 | 451090 | 0.2407 | rs331853 | 2.120 | rs3773410 | 0.074 | 6.560 | -0.808 | 249 | 19 | enet | 0.170 | 4.20E-17 | -6.31E-01 | 5.28E-01 |
| PTGES | 9 | 132500610 | 132515326 | 0.1241 | rs7857431 | 2.990 | rs3844048 | -0.002 | 3.540 | -1.499 | 83 | 13 | enet | -0.002 | 5.40E-01 | -6.30E-01 | 5.29E-01 |
| TRPM4 | 19 | 49660998 | 49715093 | 0.1219 | rs7249040 | 5.760 | rs10410857 | -0.004 | -2.840 | -0.976 | 94 | 28 | enet | 0.009 | 8.50E-02 | -6.30E-01 | 5.29E-01 |
| PSMG1 | 21 | 40546695 | 40555777 | 0.0322 | rs2836884 | -4.660 | rs2142113 | 0.011 | 3.920 | -0.473 | 114 | 8 | enet | 0.024 | 2.40E-03 | 6.30E-01 | 5.29E-01 |
| TBC1D9B | 5 | 179289066 | 179334859 | 0.4331 | rs3797769 | -2.331 | rs30386 | 0.113 | -7.270 | -0.922 | 98 | 35 | enet | 0.300 | 1.10E-30 | 6.30E-01 | 5.29E-01 |
| CAMK2D | 4 | 114372188 | 114683083 | 0.0440 | rs17446851 | -2.230 | rs757175 | 0.012 | 4.280 | -0.699 | 229 | 6 | enet | 0.023 | 1.70E-03 | -6.27E-01 | 5.31E-01 |
| ALDH9A1 | 1 | 165631453 | 165668100 | 0.3739 | rs10918271 | 2.307 | rs12408101 | 0.204 | -8.840 | -0.315 | 126 | 26 | enet | 0.200 | 7.80E-20 | 6.26E-01 | 5.31E-01 |
| AKR7A3 | 1 | 19609052 | 19615744 | 0.1030 | rs12130051 | 1.608 | rs1738023 | 0.073 | -6.070 | -0.152 | 117 | 17 | enet | 0.092 | 1.70E-09 | -6.27E-01 | 5.31E-01 |
| PDIA3 | 15 | 44038590 | 44065477 | 0.0368 | rs11070410 | -1.140 | rs678084 | 0.025 | -3.770 | -0.676 | 49 | 5 | enet | 0.026 | 9.40E-04 | 6.26E-01 | 5.31E-01 |
| PAM | 5 | 102089685 | 102366809 | 0.0480 | rs17155075 | 2.134 | rs10515341 | 0.019 | 3.290 | -0.625 | 144 | 1 | enet | 0.007 | 5.50E-02 | -6.25E-01 | 5.32E-01 |
| MEGF9 | 9 | 123363091 | 123476748 | 0.0341 | rs10984946 | 1.390 | rs10985008 | 0.012 | -3.520 | 0.938 | 76 | 9 | enet | 0.002 | 2.10E-01 | -6.25E-01 | 5.32E-01 |
| GSTM2 | 1 | 110210644 | 110252171 | 0.4759 | rs11102002 | -2.673 | rs3754446 | 0.300 | -10.670 | 0.411 | 108 | 43 | enet | 0.430 | 1.10E-47 | 6.23E-01 | 5.33E-01 |
| ORC4 | 2 | 148687968 | 148779147 | 0.0332 | rs17218875 | 2.091 | rs12105745 | 0.017 | -4.070 | 0.632 | 62 | 13 | enet | 0.024 | 2.60E-03 | -6.24E-01 | 5.33E-01 |
| MFSD11 | 17 | 74731947 | 74777531 | 0.0558 | rs2410997 | 2.200 | rs563079 | 0.067 | -4.880 | -0.997 | 100 | 6 | enet | 0.033 | 1.20E-03 | 6.24E-01 | 5.33E-01 |
| DPYSL4 | 10 | 134000404 | 134019280 | 0.1209 | rs12247439 | -2.080 | rs7096307 | 0.123 | 6.910 | 0.506 | 125 | 27 | enet | 0.093 | 8.20E-10 | 6.23E-01 | 5.33E-01 |
| AMPH | 7 | 38423305 | 38671167 | 0.1413 | rs6972549 | 2.620 | rs6956760 | 0.016 | 4.850 | 0.485 | 304 | 31 | enet | 0.058 | 1.50E-06 | -6.23E-01 | 5.34E-01 |
| FPGT | 1 | 74663896 | 74699770 | 0.1542 | rs1412827 | 1.970 | rs11806946 | 0.214 | -10.040 | -0.271 | 62 | 23 | enet | 0.260 | 1.30E-26 | 6.21E-01 | 5.35E-01 |
| SNX25 | 4 | 186125391 | 186291339 | 0.0564 | rs3733645 | 2.730 | rs3112911 | 0.058 | 4.800 | -0.232 | 135 | 6 | enet | 0.040 | 6.90E-05 | -6.20E-01 | 5.35E-01 |
| GPRIN3 | 4 | 90157537 | 90229161 | 0.2011 | rs4693997 | 2.870 | rs2005104 | 0.087 | 6.210 | 1.307 | 141 | 34 | enet | 0.099 | 2.50E-10 | 6.20E-01 | 5.35E-01 |
| CACNA2D2 | 3 | 50400233 | 50541675 | 0.1399 | rs11916258 | -2.110 | rs2071803 | 0.047 | -5.150 | -0.103 | 86 | 12 | enet | 0.074 | 5.00E-08 | 6.20E-01 | 5.35E-01 |
| RABEPK | 9 | 127962821 | 127996437 | 0.1324 | rs10760392 | 1.590 | rs393721 | 0.029 | -4.100 | 0.659 | 65 | 14 | enet | 0.072 | 2.20E-07 | 6.20E-01 | 5.36E-01 |
| ZNF622 | 5 | 16451628 | 16465901 | 0.0557 | rs16868760 | -1.654 | rs12188529 | 0.027 | 3.930 | -0.350 | 120 | 20 | enet | 0.010 | 4.10E-02 | 6.18E-01 | 5.36E-01 |
| CTSF | 11 | 66330934 | 66336312 | 0.0394 | rs10791881 | 1.890 | rs3867132 | 0.020 | 3.520 | -1.037 | 69 | 12 | enet | 0.008 | 4.80E-02 | 6.17E-01 | 5.37E-01 |

| | | | | | | | | | | | | | | | | | |
|---|---|---|---|---|---|---|---|---|---|---|---|---|---|---|---|---|---|
| TPPP3 | 16 | 67423712 | 67427438 | 0.0513 | rs9938631 | 1.924 | rs3868143 | 0.044 | -4.830 | -0.669 | 50 | 6 | enet | 0.038 | 8.00E-05 | 6.18E-01 | 5.37E-01 |
| IDH3A | 15 | 78423840 | 78464291 | 0.1551 | rs12916463 | -2.260 | rs3816253 | 0.178 | 9.080 | -0.865 | 136 | 19 | enet | 0.190 | 3.70E-19 | -6.16E-01 | 5.38E-01 |
| DPY19L1 | 7 | 34968488 | 35077883 | 0.0456 | rs10271686 | 1.970 | rs329580 | 0.026 | 4.140 | -0.489 | 84 | 7 | enet | 0.028 | 1.50E-03 | -6.15E-01 | 5.39E-01 |
| FKBP1A | 20 | 1349622 | 1373806 | 0.0810 | rs8117744 | -3.218 | rs1294689 | 0.031 | -4.290 | 0.286 | 163 | 6 | enet | 0.020 | 3.40E-03 | -6.14E-01 | 5.39E-01 |
| DENND4C | 9 | 19230433 | 19374139 | 0.1113 | rs7018718 | 3.170 | rs7873748 | 0.022 | 4.680 | 2.893 | 138 | 21 | enet | 0.035 | 1.60E-04 | 6.14E-01 | 5.39E-01 |
| PLSCR1 | 3 | 146232967 | 146262651 | 0.1385 | rs342938 | 1.870 | rs343320 | 0.086 | -5.430 | 0.978 | 129 | 11 | enet | 0.100 | 3.60E-09 | -6.14E-01 | 5.39E-01 |
| THOP1 | 19 | 2785458 | 2813599 | 0.0239 | rs9304900 | 2.330 | rs12608998 | -0.002 | 3.640 | 0.821 | 101 | 8 | enet | 0.001 | 2.50E-01 | 6.12E-01 | 5.40E-01 |
| PLS1 | 3 | 142315229 | 142432506 | 0.0391 | rs16852464 | -2.840 | rs34392719 | -0.001 | 3.680 | -0.971 | 99 | 22 | enet | -0.001 | 4.40E-01 | -6.11E-01 | 5.41E-01 |
| ANXA3 | 4 | 79472673 | 79531597 | 0.1436 | rs17003321 | -1.440 | rs1472365 | 0.067 | 5.190 | 1.161 | 149 | 11 | enet | 0.085 | 5.30E-09 | 6.11E-01 | 5.41E-01 |
| MICAL1 | 6 | 109765265 | 109787171 | 0.2450 | rs10434873 | 1.494 | rs3757237 | 0.115 | 7.220 | -0.355 | 83 | 14 | enet | 0.160 | 1.20E-15 | -6.09E-01 | 5.43E-01 |
| TESC | 12 | 117476728 | 117537284 | 0.0749 | rs6490100 | -1.860 | rs7979277 | 0.085 | -6.370 | -0.418 | 103 | 12 | enet | 0.100 | 2.10E-10 | 6.08E-01 | 5.43E-01 |
| SIDT1 | 3 | 113251143 | 113348425 | 0.3326 | rs11929640 | 3.940 | rs13074313 | 0.134 | 6.800 | -0.060 | 114 | 15 | enet | 0.220 | 1.00E-19 | 6.07E-01 | 5.44E-01 |
| ANO3 | 11 | 26210829 | 26684835 | 0.0625 | rs10834970 | 2.830 | rs10835006 | -0.001 | 3.660 | -0.048 | 375 | 25 | enet | 0.003 | 1.50E-01 | 6.06E-01 | 5.44E-01 |
| TMEM25 | 11 | 118401756 | 118417995 | 0.0736 | rs11216934 | 3.140 | rs7941217 | 0.059 | -4.790 | 1.154 | 70 | 14 | enet | 0.041 | 7.10E-05 | -6.05E-01 | 5.45E-01 |
| PAK1 | 11 | 77032752 | 77185680 | 0.0391 | rs1793488 | 2.010 | rs1670456 | 0.026 | -4.380 | -0.945 | 101 | 9 | enet | 0.025 | 1.10E-03 | 6.04E-01 | 5.46E-01 |
| CMBL | 5 | 10275987 | 10308138 | 0.0554 | rs3844305 | 1.675 | rs11951923 | 0.020 | 3.800 | -0.596 | 118 | 17 | enet | 0.037 | 1.10E-04 | -6.02E-01 | 5.47E-01 |
| ALDH5A1 | 6 | 24495080 | 24537435 | 0.2030 | rs1321820 | 2.125 | rs7775073 | 0.207 | -9.130 | 0.604 | 185 | 16 | enet | 0.210 | 5.80E-21 | -6.03E-01 | 5.47E-01 |
| TSPO | 22 | 43547520 | 43559248 | 0.0928 | rs998409 | 2.410 | rs1076922 | 0.036 | -4.270 | 0.489 | 152 | 36 | enet | 0.016 | 7.40E-03 | -6.01E-01 | 5.48E-01 |
| FAH | 15 | 80444832 | 80479288 | 0.5810 | rs1370276 | -2.170 | rs11555096 | 0.193 | -10.000 | -2.030 | 112 | 21 | enet | 0.410 | 5.30E-45 | 6.01E-01 | 5.48E-01 |
| GNPDA1 | 5 | 141371314 | 141392606 | 0.2496 | rs12653848 | -4.302 | rs164080 | 0.093 | -6.330 | -0.106 | 125 | 32 | enet | 0.110 | 2.10E-11 | 6.01E-01 | 5.48E-01 |
| RAPGEF3 | 12 | 48128455 | 48164823 | 0.0676 | rs11831883 | -3.870 | rs12422983 | 0.030 | -4.860 | -0.504 | 128 | 8 | enet | 0.030 | 6.20E-04 | 6.01E-01 | 5.48E-01 |
| DUSP3 | 17 | 41843489 | 41856356 | 0.0576 | rs2090019 | 4.330 | rs2074143 | 0.004 | -3.630 | 0.543 | 80 | 11 | enet | -0.002 | 5.30E-01 | -6.01E-01 | 5.48E-01 |
| CCM2 | 7 | 45039074 | 45116068 | 0.1039 | rs6946581 | 2.700 | rs11765574 | 0.000 | 3.370 | 0.630 | 122 | 12 | enet | 0.000 | 3.10E-01 | 6.00E-01 | 5.48E-01 |
| PLCD1 | 3 | 38048987 | 38071253 | 0.0983 | rs11129780 | 2.400 | rs17037286 | 0.033 | 4.690 | 0.156 | 93 | 7 | enet | 0.043 | 2.70E-05 | -6.00E-01 | 5.49E-01 |
| POR | 7 | 75528518 | 75616173 | 0.3236 | rs28737229 | 2.690 | rs10755885 | 0.009 | -4.630 | 0.374 | 103 | 24 | enet | 0.140 | 2.90E-14 | -6.00E-01 | 5.49E-01 |
| PTPLAD1 | 15 | 65822756 | 65870687 | 0.0269 | rs12595213 | 1.610 | rs2456015 | 0.014 | 3.400 | 0.981 | 72 | 9 | enet | 0.019 | 4.30E-03 | 5.98E-01 | 5.50E-01 |
| PGM1 | 1 | 64058947 | 64125916 | 0.2705 | rs10889433 | 2.782 | rs4643 | 0.125 | -7.400 | -1.052 | 139 | 31 | enet | 0.200 | 1.20E-19 | 5.97E-01 | 5.51E-01 |
| PDE1A | 2 | 183004763 | 183387919 | 0.0711 | rs11676337 | 2.037 | rs11884886 | 0.034 | 4.590 | -0.766 | 268 | 22 | enet | 0.019 | 4.40E-03 | -5.97E-01 | 5.51E-01 |
| NUMB | 14 | 73741815 | 73930348 | 0.0461 | rs10138789 | 2.320 | rs177378 | 0.016 | -4.010 | -1.516 | 127 | 17 | enet | 0.011 | 2.30E-02 | 5.96E-01 | 5.51E-01 |
| SLC7A6OS | 16 | 68318406 | 68344849 | 0.0714 | rs7190070 | 2.981 | rs3961283 | 0.055 | -4.120 | -1.964 | 88 | 5 | enet | 0.007 | 8.60E-02 | 5.97E-01 | 5.51E-01 |
| FGGY | 1 | 59762310 | 60233347 | 0.1122 | rs12071725 | 2.711 | rs835425 | 0.015 | 4.110 | 0.825 | 230 | 29 | enet | 0.031 | 1.60E-03 | 5.95E-01 | 5.52E-01 |
| CGREF1 | 2 | 27321757 | 27341995 | 0.0401 | rs934986 | 2.545 | rs1866654 | 0.003 | 3.420 | 0.181 | 75 | 15 | enet | 0.000 | 3.40E-01 | -5.96E-01 | 5.52E-01 |
| DIS3L2 | 2 | 232825955 | 233209060 | 0.1078 | rs11904384 | 2.152 | rs6715778 | 0.008 | 4.530 | 0.854 | 216 | 10 | enet | 0.015 | 1.00E-02 | 5.95E-01 | 5.52E-01 |
| TTC37 | 5 | 94799599 | 94890711 | 0.0691 | rs7731790 | -2.526 | rs3822617 | 0.046 | -4.860 | 0.925 | 122 | 12 | enet | 0.018 | 4.70E-03 | -5.94E-01 | 5.52E-01 |
| SNX1 | 15 | 64386322 | 64438289 | 0.0384 | rs17789612 | 1.990 | rs3887731 | 0.020 | -4.050 | 1.142 | 72 | 22 | enet | 0.013 | 1.40E-02 | -5.94E-01 | 5.52E-01 |
| EVI5L | 19 | 7895119 | 7929862 | 0.0473 | rs1864029 | -2.490 | rs520802 | 0.008 | -3.890 | -1.489 | 104 | 17 | enet | 0.006 | 6.90E-02 | 5.94E-01 | 5.53E-01 |
| ALDH4A1 | 1 | 19197926 | 19229275 | 0.2067 | rs2024725 | 3.224 | rs9426794 | 0.159 | 7.870 | 0.941 | 105 | 28 | enet | 0.160 | 2.40E-16 | 5.93E-01 | 5.53E-01 |
| CTSB | 8 | 11700033 | 11726957 | 0.1229 | rs7817757 | 3.710 | rs3947 | 0.075 | 6.100 | -1.183 | 146 | 15 | enet | 0.088 | 2.50E-09 | -5.93E-01 | 5.53E-01 |
| SLC20A2 | 8 | 42273993 | 42397069 | 0.0732 | rs12335053 | -1.700 | rs2923428 | 0.083 | 5.870 | -0.377 | 96 | 21 | enet | 0.076 | 4.20E-08 | -5.94E-01 | 5.53E-01 |
| PLCB1 | 20 | 8112824 | 8949003 | 0.1010 | rs6039170 | -2.883 | rs3923472 | 0.001 | -3.540 | 0.178 | 653 | 52 | enet | 0.029 | 5.70E-04 | 5.92E-01 | 5.54E-01 |
| NASP | 1 | 46049518 | 46084566 | 0.0314 | rs534534 | 1.601 | rs3014210 | 0.025 | -4.570 | 0.669 | 84 | 13 | enet | 0.024 | 1.60E-03 | -5.91E-01 | 5.55E-01 |
| GNB1L | 22 | 19770747 | 19842462 | 0.1600 | rs756661 | 2.570 | rs2073762 | 0.124 | 7.290 | 0.627 | 153 | 20 | enet | 0.150 | 9.40E-15 | 5.89E-01 | 5.56E-01 |
| CPNE3 | 8 | 87497059 | 87573726 | 0.1474 | rs16916239 | 1.710 | rs6415565 | 0.108 | 6.530 | 0.762 | 110 | 29 | enet | 0.120 | 1.50E-12 | 5.90E-01 | 5.56E-01 |
| COQ6 | 14 | 74416629 | 74430373 | 0.1005 | rs2336547 | 1.870 | rs3764859 | 0.134 | 7.360 | -0.294 | 97 | 20 | enet | 0.120 | 3.60E-12 | -5.89E-01 | 5.56E-01 |
| TAOK3 | 12 | 118587606 | 118810750 | 0.0592 | rs7132650 | 3.090 | rs16948204 | 0.030 | -4.840 | 2.129 | 172 | 18 | enet | 0.029 | 5.50E-04 | -5.86E-01 | 5.58E-01 |
| DHODH | 16 | 72042487 | 72058954 | 0.4720 | rs7197967 | 2.988 | rs7184117 | 0.271 | -10.400 | 0.619 | 126 | 17 | enet | 0.320 | 1.00E-33 | -5.85E-01 | 5.59E-01 |

| | | | | | | | | | | | | | | | | | |
|---|---|---|---|---|---|---|---|---|---|---|---|---|---|---|---|---|---|
| STX17 | 9 | 102668915 | 102732618 | 0.1063 | rs35132689 | 1.870 | rs4428721 | 0.055 | 5.540 | 0.319 | 85 | 25 | enet | 0.097 | 3.60E-10 | 5.81E-01 | 5.61E-01 |
| SUCLG2 | 3 | 67410884 | 67705038 | 0.1309 | rs6779718 | -2.070 | rs17806888 | 0.049 | -5.310 | -0.176 | 258 | 15 | enet | 0.075 | 3.60E-08 | 5.81E-01 | 5.61E-01 |
| SESTD1 | 2 | 179966419 | 180129517 | 0.0301 | rs16866687 | 2.337 | rs17454311 | 0.017 | -3.850 | 0.414 | 133 | 4 | enet | 0.006 | 6.50E-02 | -5.80E-01 | 5.62E-01 |
| OSTM1 | 6 | 108362613 | 108487058 | 0.0281 | rs6919324 | -1.948 | rs9386694 | 0.037 | 4.330 | -0.595 | 117 | 8 | enet | 0.020 | 4.00E-03 | -5.78E-01 | 5.63E-01 |
| RMDN1 | 8 | 87480486 | 87526586 | 0.1740 | rs11775728 | -1.830 | rs6471309 | 0.263 | -10.010 | -0.738 | 87 | 25 | enet | 0.260 | 5.70E-27 | 5.78E-01 | 5.63E-01 |
| PABPC4 | 1 | 40026488 | 40042462 | 0.0517 | rs2275767 | 2.227 | rs783820 | 0.003 | -3.570 | -0.099 | 70 | 13 | enet | 0.006 | 7.20E-02 | -5.78E-01 | 5.64E-01 |
| CDK18 | 1 | 205473723 | 205501921 | 0.2046 | rs11240524 | -2.621 | rs12757179 | 0.039 | -5.300 | 0.406 | 124 | 24 | enet | 0.100 | 1.90E-10 | -5.77E-01 | 5.64E-01 |
| CYSTM1 | 5 | 139554227 | 139661637 | 0.1331 | rs13385 | -2.251 | rs269782 | 0.171 | -7.210 | -0.597 | 84 | 19 | enet | 0.140 | 1.10E-11 | 5.76E-01 | 5.64E-01 |
| UBE3B | 12 | 109915207 | 109974507 | 0.1340 | rs16940177 | -1.970 | rs11067165 | 0.017 | 4.440 | 0.103 | 131 | 13 | enet | 0.041 | 4.70E-05 | 5.76E-01 | 5.65E-01 |
| HLCS | 21 | 38123189 | 38362536 | 0.1036 | rs1012959 | 2.290 | rs1235344 | 0.010 | -4.550 | -0.115 | 211 | 27 | enet | 0.069 | 2.10E-07 | 5.74E-01 | 5.66E-01 |
| CDA | 1 | 20915441 | 20945401 | 0.1599 | rs12756783 | -2.593 | rs1689924 | 0.114 | -5.910 | -0.267 | 175 | 19 | enet | 0.120 | 2.00E-10 | -5.73E-01 | 5.67E-01 |
| ABCB8 | 7 | 150725510 | 150744869 | 0.2667 | rs11771443 | -1.930 | rs11770520 | 0.174 | -8.250 | -0.250 | 101 | 16 | enet | 0.230 | 7.80E-23 | 5.73E-01 | 5.67E-01 |
| ADSSL1 | 14 | 105190523 | 105213662 | 0.0262 | rs10142116 | -1.890 | rs2498804 | -0.001 | -3.510 | 0.903 | 57 | 7 | enet | -0.003 | 9.50E-01 | -5.73E-01 | 5.67E-01 |
| OXSR1 | 3 | 38206580 | 38296979 | 0.1524 | rs2070491 | 2.490 | rs7744 | 0.110 | -6.520 | -0.610 | 92 | 8 | enet | 0.094 | 6.70E-10 | 5.68E-01 | 5.70E-01 |
| DGKG | 3 | 185823457 | 186080026 | 0.1292 | rs6765480 | -2.600 | rs1004589 | -0.001 | 4.170 | -1.818 | 212 | 20 | enet | 0.027 | 8.20E-04 | -5.67E-01 | 5.71E-01 |
| JAM2 | 21 | 27011584 | 27089874 | 0.2314 | rs17407272 | -2.530 | rs974680 | 0.100 | 7.970 | -1.308 | 92 | 21 | enet | 0.180 | 5.60E-18 | -5.66E-01 | 5.71E-01 |
| SUCLG1 | 2 | 84650647 | 84687169 | 0.0370 | rs17705525 | 2.453 | rs10173985 | 0.034 | 3.910 | 0.159 | 63 | 6 | enet | 0.034 | 2.20E-04 | 5.66E-01 | 5.72E-01 |
| CPT1A | 11 | 68522088 | 68611878 | 0.1114 | rs3019596 | 2.370 | rs4460815 | 0.062 | 5.710 | 0.219 | 121 | 35 | enet | 0.094 | 7.20E-10 | 5.65E-01 | 5.72E-01 |
| ANKRD16 | 10 | 5903580 | 5931869 | 0.2113 | rs6602360 | -4.530 | rs3750656 | 0.125 | -7.460 | 0.590 | 159 | 40 | enet | 0.230 | 1.40E-20 | -5.65E-01 | 5.72E-01 |
| TBC1D22A | 22 | 47158518 | 47571336 | 0.1193 | rs2227106 | 3.040 | rs136126 | 0.006 | 4.270 | -0.555 | 368 | 43 | enet | 0.019 | 4.70E-03 | -5.64E-01 | 5.73E-01 |
| SCLY | 2 | 238969530 | 239008054 | 0.4145 | rs1374386 | 2.934 | rs1128552 | 0.444 | 12.960 | -0.570 | 97 | 19 | enet | 0.540 | 8.20E-65 | 5.64E-01 | 5.73E-01 |
| CCDC92 | 12 | 124403207 | 124457378 | 0.0640 | rs4930728 | 1.980 | rs4930726 | 0.103 | -6.660 | 0.890 | 97 | 14 | enet | 0.087 | 3.00E-09 | -5.63E-01 | 5.73E-01 |
| ACOT1 | 14 | 74003818 | 74010498 | 0.4758 | rs10138789 | 2.320 | rs1074501 | 0.275 | 10.730 | -0.301 | 75 | 23 | enet | 0.490 | 4.00E-57 | -5.62E-01 | 5.74E-01 |
| DHRS7B | 17 | 21026677 | 21096578 | 0.0645 | rs10468608 | -1.780 | rs735091 | 0.064 | 5.290 | -0.580 | 87 | 6 | enet | 0.044 | 2.50E-05 | -5.62E-01 | 5.74E-01 |
| RAB8B | 15 | 63481668 | 63559981 | 0.0462 | rs2729786 | 1.910 | rs1444405 | 0.029 | -5.010 | -0.355 | 101 | 6 | enet | 0.038 | 7.40E-05 | 5.61E-01 | 5.75E-01 |
| HOGA1 | 10 | 99344080 | 99372559 | 0.1002 | rs7074482 | -2.490 | rs2297643 | 0.040 | -4.760 | -0.280 | 104 | 7 | enet | 0.067 | 6.00E-07 | 5.61E-01 | 5.75E-01 |
| HSD17B12 | 11 | 43577986 | 43878167 | 0.3250 | rs1115491 | 2.320 | rs1061810 | 0.187 | -9.510 | 1.474 | 227 | 31 | enet | 0.370 | 2.20E-40 | -5.61E-01 | 5.75E-01 |
| PFAS | 17 | 8150936 | 8173809 | 0.2482 | rs8069620 | 2.830 | rs9891699 | 0.270 | 10.460 | 0.290 | 112 | 14 | enet | 0.330 | 2.40E-34 | 5.60E-01 | 5.75E-01 |
| CCDC6 | 10 | 61548521 | 61666414 | 0.0407 | rs7098890 | 2.750 | rs10821636 | 0.003 | 3.890 | 1.039 | 197 | 13 | enet | 0.006 | 7.20E-02 | 5.60E-01 | 5.75E-01 |
| CISD1 | 10 | 60028818 | 60049346 | 0.0598 | rs12247015 | 2.490 | rs1199103 | 0.046 | 5.160 | -0.521 | 88 | 29 | enet | 0.045 | 2.10E-05 | -5.60E-01 | 5.75E-01 |
| FAHD2B | 2 | 97749320 | 97760619 | 0.4035 | rs11164114 | -0.906 | rs10496325 | 0.142 | -7.370 | 0.173 | 18 | 6 | enet | 0.220 | 5.40E-17 | 5.58E-01 | 5.77E-01 |
| PAK4 | 19 | 39616410 | 39673456 | 0.0628 | rs8106486 | 2.330 | rs692191 | 0.021 | 4.330 | -1.509 | 92 | 16 | enet | 0.019 | 4.20E-03 | -5.57E-01 | 5.77E-01 |
| EPHX1 | 1 | 225997794 | 226033260 | 0.0818 | rs1557092 | -2.647 | rs12038486 | 0.080 | -5.600 | 0.361 | 113 | 10 | enet | 0.059 | 1.00E-06 | -5.56E-01 | 5.78E-01 |
| DTNBP1 | 6 | 15523032 | 15663289 | 0.0622 | rs3213207 | -2.815 | rs2237113 | 0.008 | 3.910 | -1.196 | 178 | 18 | enet | 0.001 | 2.40E-01 | -5.55E-01 | 5.79E-01 |
| ACAT2 | 6 | 160181360 | 160200144 | 0.0461 | rs11965350 | 2.148 | rs2295899 | 0.048 | 5.110 | -0.180 | 91 | 21 | enet | 0.041 | 4.70E-05 | -5.55E-01 | 5.79E-01 |
| SFXN5 | 2 | 73169165 | 73302747 | 0.1282 | rs999494 | 2.439 | rs1048704 | 0.106 | 6.670 | -0.538 | 100 | 7 | enet | 0.100 | 1.50E-10 | -5.53E-01 | 5.80E-01 |
| SRPR | 11 | 126132814 | 126139039 | 0.0250 | rs4937122 | -3.430 | rs687033 | -0.003 | -3.380 | -0.946 | 134 | 7 | enet | -0.003 | 9.00E-01 | 5.54E-01 | 5.80E-01 |
| LRPAP1 | 4 | 3508103 | 3534286 | 0.1975 | rs12641989 | 1.820 | rs6789 | 0.132 | 7.610 | 1.130 | 87 | 18 | enet | 0.190 | 3.60E-19 | 5.53E-01 | 5.81E-01 |
| KIAA1239 | 4 | 37245842 | 37451087 | 0.0966 | rs17603438 | 2.520 | rs4478199 | 0.002 | -3.600 | -1.306 | 244 | 18 | enet | 0.007 | 6.10E-02 | 5.52E-01 | 5.81E-01 |
| ADCY9 | 16 | 4003388 | 4166186 | 0.0756 | rs1978300 | -2.470 | rs2230739 | 0.057 | -5.000 | -0.710 | 179 | 15 | enet | 0.034 | 1.90E-04 | -5.51E-01 | 5.81E-01 |
| PVR | 19 | 45147098 | 45166850 | 0.1561 | rs7260482 | 4.770 | rs714948 | 0.090 | -5.900 | -0.334 | 86 | 17 | enet | 0.095 | 9.70E-10 | 5.51E-01 | 5.81E-01 |
| MX1 | 21 | 42792231 | 42831141 | 0.0675 | rs430915 | 2.490 | rs469066 | 0.066 | -5.840 | 0.968 | 164 | 9 | enet | 0.064 | 4.10E-07 | -5.50E-01 | 5.83E-01 |
| PEPD | 19 | 33877856 | 34012700 | 0.3380 | rs2772 | -2.360 | rs3556 | 0.385 | 12.340 | 0.268 | 157 | 17 | enet | 0.500 | 4.00E-59 | -5.50E-01 | 5.83E-01 |
| SLC33A1 | 3 | 155538813 | 155572218 | 0.1164 | rs9850228 | -1.550 | rs11921267 | 0.117 | -6.620 | 0.883 | 73 | 17 | enet | 0.140 | 3.30E-13 | -5.46E-01 | 5.85E-01 |
| C2CD2 | 21 | 43305221 | 43373999 | 0.0979 | rs9983923 | -3.120 | rs2839403 | 0.049 | 5.210 | -1.025 | 232 | 15 | enet | 0.047 | 1.20E-05 | -5.46E-01 | 5.85E-01 |
| HTATIP2 | 11 | 20385231 | 20405329 | 0.1716 | rs7112050 | 2.250 | rs10833312 | 0.074 | -5.570 | -0.606 | 108 | 20 | enet | 0.076 | 2.90E-08 | -5.45E-01 | 5.86E-01 |

| | | | | | | | | | | | | | | | | | |
|---|---|---|---|---|---|---|---|---|---|---|---|---|---|---|---|---|---|
| EIF4E3 | 3 | 71724567 | 71803924 | 0.2093 | rs9828619 | 4.610 | rs2029013 | 0.070 | -5.930 | -0.139 | 139 | 21 | enet | 0.110 | 8.90E-11 | -5.44E-01 | 5.87E-01 |
| CCDC91 | 12 | 28286182 | 28732883 | 0.1735 | rs17529138 | -2.950 | rs12371059 | 0.077 | 6.870 | -1.930 | 272 | 13 | enet | 0.190 | 1.50E-19 | -5.41E-01 | 5.89E-01 |
| NMRK1 | 9 | 77675489 | 77703133 | 0.1255 | rs2273762 | 2.240 | rs3780178 | 0.005 | -4.350 | 0.743 | 111 | 5 | enet | 0.028 | 2.70E-03 | -5.39E-01 | 5.90E-01 |
| MMAA | 4 | 146539415 | 146581187 | 0.3515 | rs1016792 | -2.510 | rs17020467 | 0.290 | -10.980 | -0.693 | 81 | 27 | enet | 0.330 | 1.00E-34 | 5.38E-01 | 5.91E-01 |
| C2orf76 | 2 | 120059801 | 120124404 | 0.1967 | rs2579643 | 3.154 | rs6712455 | 0.201 | 7.570 | 0.554 | 157 | 12 | enet | 0.170 | 5.00E-13 | 5.35E-01 | 5.92E-01 |
| SLC13A5 | 17 | 6588032 | 6616886 | 0.0489 | rs16956162 | 2.050 | rs1983177 | 0.060 | 4.910 | -1.486 | 148 | 6 | enet | 0.052 | 4.60E-06 | -5.35E-01 | 5.92E-01 |
| NDUFAF5 | 20 | 13765596 | 13799067 | 0.0535 | rs11698731 | -2.706 | rs1999112 | 0.044 | -4.320 | -0.438 | 95 | 9 | enet | 0.036 | 1.30E-04 | 5.35E-01 | 5.93E-01 |
| NARS2 | 11 | 78147007 | 78285919 | 0.2738 | rs4945276 | -3.000 | rs4945300 | 0.112 | -6.810 | 0.773 | 96 | 19 | enet | 0.100 | 9.10E-11 | 5.33E-01 | 5.94E-01 |
| GFM1 | 3 | 158362067 | 158410364 | 0.1279 | rs7617304 | 3.030 | rs17699324 | 0.066 | -5.440 | 0.731 | 97 | 27 | enet | 0.081 | 1.10E-08 | 5.33E-01 | 5.94E-01 |
| PGP | 16 | 2261998 | 2264808 | 0.0573 | rs161426 | -2.342 | rs26848 | 0.034 | 4.860 | 0.923 | 56 | 3 | enet | 0.026 | 9.90E-04 | 5.30E-01 | 5.96E-01 |
| ZDHHC20 | 13 | 21950263 | 22033509 | 0.0983 | rs2095476 | -2.770 | rs9506668 | -0.004 | 3.150 | -1.071 | 127 | 16 | enet | 0.001 | 2.60E-01 | -5.29E-01 | 5.97E-01 |
| TATDN1 | 8 | 125500726 | 125551699 | 0.0774 | rs7836478 | 2.960 | rs16899649 | 0.024 | -4.440 | 0.098 | 134 | 15 | enet | 0.030 | 4.30E-04 | 5.29E-01 | 5.97E-01 |
| CES1 | 16 | 55836763 | 55867249 | 0.2016 | rs1870038 | 2.700 | rs11076120 | 0.129 | 7.090 | -0.309 | 118 | 31 | enet | 0.140 | 4.80E-14 | 5.28E-01 | 5.98E-01 |
| PACSIN1 | 6 | 34433916 | 34503006 | 0.0715 | rs10947500 | -2.022 | rs10947510 | -0.002 | 3.390 | -0.052 | 84 | 21 | enet | 0.001 | 2.20E-01 | 5.25E-01 | 6.00E-01 |
| SEC23B | 20 | 18488137 | 18542059 | 0.0625 | rs6132059 | -2.526 | rs2247746 | 0.039 | 4.370 | -0.633 | 109 | 7 | enet | 0.026 | 1.20E-03 | -5.24E-01 | 6.00E-01 |
| PPP2R2A | 8 | 26149007 | 26230196 | 0.0841 | rs11775561 | 2.390 | rs12682345 | 0.047 | -4.580 | 0.559 | 150 | 23 | enet | 0.021 | 2.70E-03 | -5.22E-01 | 6.01E-01 |
| FDPS | 1 | 155278539 | 155290457 | 0.1950 | rs2990245 | -2.112 | rs2297480 | 0.284 | -10.460 | 0.600 | 40 | 9 | enet | 0.270 | 4.50E-28 | -5.21E-01 | 6.03E-01 |
| NUDT14 | 14 | 105639275 | 105647660 | 0.1728 | rs8270 | 3.450 | rs4983590 | 0.091 | -5.980 | -1.531 | 69 | 12 | enet | 0.092 | 2.50E-08 | 5.20E-01 | 6.03E-01 |
| SGTB | 5 | 64961755 | 65018862 | 0.1263 | rs2248213 | -3.117 | rs16894005 | 0.117 | -6.880 | 0.595 | 123 | 8 | enet | 0.110 | 1.60E-11 | -5.19E-01 | 6.04E-01 |
| SLC25A15 | 13 | 41363548 | 41384247 | 0.1665 | rs4943810 | 1.310 | rs7337312 | 0.136 | 6.990 | 0.645 | 46 | 11 | enet | 0.150 | 2.20E-11 | 5.16E-01 | 6.06E-01 |
| SYTL2 | 11 | 85405267 | 85522184 | 0.2304 | rs290180 | -2.680 | rs2276018 | 0.018 | 4.370 | 0.075 | 141 | 28 | enet | 0.070 | 1.30E-07 | 5.16E-01 | 6.06E-01 |
| NECAB2 | 16 | 84002237 | 84036381 | 0.1048 | rs12447570 | 2.211 | rs2292326 | 0.085 | -6.300 | -0.508 | 175 | 16 | enet | 0.083 | 8.00E-09 | 5.16E-01 | 6.06E-01 |
| P2RX7 | 12 | 121570622 | 121623876 | 0.6722 | rs7314454 | 2.480 | rs3751143 | 0.433 | -12.820 | 0.732 | 144 | 42 | enet | 0.630 | 6.00E-84 | -5.14E-01 | 6.07E-01 |
| ARHGAP44 | 17 | 12692856 | 12894960 | 0.0815 | rs16946601 | -2.710 | rs9904572 | 0.027 | 4.420 | -0.339 | 214 | 6 | enet | 0.035 | 1.40E-04 | -5.15E-01 | 6.07E-01 |
| TAX1BP3 | 17 | 3566196 | 3571976 | 0.0650 | rs3744678 | 1.830 | rs161397 | 0.041 | 4.770 | -0.124 | 119 | 5 | enet | 0.033 | 2.20E-04 | 5.14E-01 | 6.07E-01 |
| DNAJB1 | 19 | 14625582 | 14640582 | 0.0763 | rs1035395 | -1.730 | rs755892 | 0.036 | -5.040 | -0.961 | 79 | 16 | enet | 0.066 | 2.50E-07 | 5.14E-01 | 6.07E-01 |
| ABCG1 | 21 | 43619799 | 43717354 | 0.1530 | rs7277003 | -2.910 | rs2839468 | 0.031 | -3.880 | 0.698 | 248 | 28 | enet | 0.024 | 7.80E-03 | 5.14E-01 | 6.07E-01 |
| SERPINB9 | 6 | 2887500 | 2903514 | 0.2550 | rs7743193 | -2.452 | rs318491 | 0.090 | -6.160 | 0.089 | 134 | 22 | enet | 0.150 | 6.60E-15 | -5.11E-01 | 6.09E-01 |
| DCPS | 11 | 126173647 | 126215644 | 0.2515 | rs4937122 | -3.430 | rs11220426 | 0.075 | -6.270 | -1.727 | 145 | 46 | enet | 0.100 | 1.60E-10 | 5.11E-01 | 6.09E-01 |
| SCFD1 | 14 | 31091318 | 31205018 | 0.2027 | rs12897219 | 3.190 | rs12434151 | 0.353 | 11.660 | 0.700 | 138 | 19 | enet | 0.350 | 1.40E-36 | 5.11E-01 | 6.09E-01 |
| SLC30A9 | 4 | 41992489 | 42092474 | 0.1245 | rs13151738 | -1.940 | rs6848386 | 0.044 | 4.690 | 0.058 | 116 | 13 | enet | 0.070 | 1.00E-07 | -5.09E-01 | 6.10E-01 |
| BPHL | 6 | 3118608 | 3153812 | 0.5011 | rs2077681 | 1.895 | rs10458071 | 0.338 | -11.410 | -0.194 | 75 | 31 | enet | 0.350 | 4.20E-37 | 5.09E-01 | 6.11E-01 |
| CD38 | 4 | 15779898 | 15854853 | 0.2217 | rs884473 | -2.290 | rs4698412 | 0.157 | 7.870 | -0.375 | 166 | 18 | enet | 0.190 | 8.90E-19 | -5.08E-01 | 6.11E-01 |
| PEBP1 | 12 | 118573663 | 118583389 | 0.0391 | rs795484 | -2.450 | rs795484 | 0.006 | 3.390 | -2.451 | 88 | 14 | enet | 0.007 | 6.10E-02 | -5.08E-01 | 6.12E-01 |
| MPST | 22 | 37415676 | 37425863 | 0.0470 | rs4821568 | 4.000 | rs2160907 | 0.023 | 3.890 | 0.090 | 146 | 18 | enet | 0.012 | 1.90E-02 | 5.07E-01 | 6.12E-01 |
| FOXRED1 | 11 | 126138950 | 126148026 | 0.0387 | rs4937122 | -3.430 | rs530587 | 0.037 | -4.510 | -1.220 | 139 | 16 | enet | 0.009 | 3.70E-02 | 5.06E-01 | 6.13E-01 |
| VSTM2A | 7 | 54610018 | 54638773 | 0.0556 | rs331827 | 1.800 | rs507047 | 0.001 | 3.520 | -0.462 | 135 | 15 | enet | 0.018 | 4.90E-03 | -5.06E-01 | 6.13E-01 |
| EXOC6 | 10 | 94590935 | 94819250 | 0.0848 | rs2497304 | 5.180 | rs1326329 | 0.088 | 5.990 | -0.312 | 218 | 16 | enet | 0.072 | 7.70E-08 | -5.01E-01 | 6.17E-01 |
| LPIN1 | 2 | 11817721 | 11967535 | 0.0602 | rs4233899 | 2.117 | rs3795974 | 0.004 | -4.440 | -0.610 | 171 | 10 | enet | 0.023 | 2.00E-03 | -5.00E-01 | 6.17E-01 |
| RAB5A | 3 | 19988571 | 20026667 | 0.0559 | rs13081502 | 2.660 | rs13072891 | 0.005 | 3.940 | 0.142 | 106 | 13 | enet | 0.009 | 3.50E-02 | -4.99E-01 | 6.17E-01 |
| TMEM127 | 2 | 96914254 | 96931732 | 0.2877 | rs4435501 | 3.404 | rs17119410 | 0.073 | 4.670 | -0.316 | 44 | 29 | enet | 0.030 | 6.40E-03 | -4.99E-01 | 6.18E-01 |
| INPP1 | 2 | 191208196 | 191236391 | 0.3529 | rs4340549 | -2.903 | rs7592352 | 0.111 | -6.730 | -0.180 | 93 | 9 | enet | 0.130 | 5.10E-13 | 4.98E-01 | 6.18E-01 |
| SNX15 | 11 | 64794880 | 64808044 | 0.1234 | rs1129195 | 2.470 | rs534236 | 0.000 | -4.920 | -0.759 | 96 | 18 | enet | 0.040 | 6.90E-05 | 4.99E-01 | 6.18E-01 |
| CRYZ | 1 | 75171170 | 75199092 | 0.6612 | rs11163464 | 2.818 | rs17552114 | 0.180 | -8.990 | 0.042 | 104 | 50 | enet | 0.620 | 1.30E-80 | 4.97E-01 | 6.19E-01 |
| GREM2 | 1 | 240652873 | 240775449 | 0.1545 | rs1889869 | 2.840 | rs12122491 | 0.050 | -3.990 | -0.643 | 213 | 6 | enet | 0.036 | 2.90E-03 | 4.97E-01 | 6.19E-01 |
| TDRKH | 1 | 151742583 | 151763892 | 0.0441 | rs9645406 | 2.473 | rs12758387 | 0.036 | -4.450 | -0.464 | 95 | 10 | enet | 0.020 | 3.60E-03 | 4.97E-01 | 6.19E-01 |

| | | | | | | | | | | | | | | | | | |
|---|---|---|---|---|---|---|---|---|---|---|---|---|---|---|---|---|---|
| PLIN3 | 19 | 4838353 | 4867780 | 0.1118 | rs11672931 | 2.600 | rs8289 | 0.033 | -4.850 | -0.809 | 96 | 4 | enet | 0.068 | 1.50E-07 | 4.96E-01 | 6.20E-01 |
| AKR1C1 | 10 | 4934796 | 5025475 | 0.1924 | rs11252746 | 2.510 | rs4323780 | 0.030 | -4.510 | 0.016 | 191 | 12 | enet | 0.039 | 6.20E-05 | 4.96E-01 | 6.20E-01 |
| NAV1 | 1 | 201592411 | 201796102 | 0.0594 | rs950114 | -2.346 | rs481530 | 0.029 | 3.790 | 0.239 | 192 | 16 | enet | 0.005 | 9.20E-02 | 4.95E-01 | 6.20E-01 |
| MXRA7 | 17 | 74668633 | 74707098 | 0.0708 | rs2410997 | 2.200 | rs7211963 | 0.031 | 4.820 | 0.180 | 98 | 11 | enet | 0.041 | 4.10E-05 | 4.96E-01 | 6.20E-01 |
| PPP2R5C | 14 | 102228135 | 102394326 | 0.2797 | rs2474675 | 2.720 | rs736449 | 0.171 | -8.390 | 0.253 | 104 | 34 | enet | 0.220 | 5.20E-22 | 4.95E-01 | 6.21E-01 |
| C6orf211 | 6 | 151773422 | 151791236 | 0.0542 | rs7753676 | -2.307 | rs7771156 | 0.054 | -5.180 | -0.335 | 97 | 13 | enet | 0.059 | 1.20E-06 | 4.94E-01 | 6.22E-01 |
| SNX16 | 8 | 82711816 | 82755101 | 0.0969 | rs17633897 | -2.200 | rs1817107 | 0.059 | 4.480 | -0.264 | 102 | 19 | enet | 0.057 | 1.30E-05 | -4.94E-01 | 6.22E-01 |
| ADAL | 15 | 43622872 | 43646096 | 0.0569 | rs542036 | -1.660 | rs6493085 | 0.041 | 3.840 | -0.483 | 78 | 5 | enet | 0.009 | 7.90E-02 | -4.92E-01 | 6.23E-01 |
| HTR1A | 5 | 63256183 | 63258334 | 0.0693 | rs10044063 | -1.611 | rs16892364 | 0.023 | -4.230 | -0.494 | 42 | 5 | enet | 0.010 | 3.10E-02 | 4.91E-01 | 6.23E-01 |
| LIME1 | 20 | 62366815 | 62370456 | 0.0706 | rs6742 | -5.279 | rs6062504 | 0.045 | -4.110 | 0.256 | 82 | 13 | enet | 0.017 | 1.60E-02 | 4.91E-01 | 6.23E-01 |
| RAD23B | 9 | 110045418 | 110094475 | 0.2088 | rs1519039 | 2.650 | rs17196438 | 0.003 | 3.810 | -1.150 | 147 | 31 | enet | 0.049 | 8.40E-06 | 4.89E-01 | 6.25E-01 |
| TST | 22 | 37406900 | 37415681 | 0.1094 | rs4821560 | 4.180 | rs6000522 | 0.007 | 4.390 | 0.541 | 148 | 21 | enet | 0.053 | 4.10E-06 | 4.84E-01 | 6.28E-01 |
| NOS1 | 12 | 117645947 | 117889975 | 0.0684 | rs11068469 | -3.070 | rs10774926 | 0.010 | -4.220 | -0.531 | 269 | 12 | enet | 0.029 | 5.70E-04 | 4.83E-01 | 6.29E-01 |
| LRRC8A | 9 | 131644391 | 131680318 | 0.0523 | rs3750340 | 2.560 | rs10988151 | 0.046 | -4.780 | -0.510 | 73 | 4 | enet | 0.040 | 4.90E-05 | 4.81E-01 | 6.31E-01 |
| AK4 | 1 | 65613232 | 65697828 | 0.1333 | rs547812 | -4.100 | rs6699614 | 0.123 | 6.970 | -0.571 | 126 | 21 | enet | 0.130 | 4.50E-13 | 4.80E-01 | 6.31E-01 |
| PANX1 | 11 | 93862094 | 93915138 | 0.0888 | rs2511410 | 2.390 | rs12419615 | 0.026 | -4.390 | 0.830 | 84 | 12 | enet | 0.050 | 9.10E-06 | -4.80E-01 | 6.31E-01 |
| NDUFAF6 | 8 | 95907995 | 96128683 | 0.0564 | rs2956217 | 2.550 | rs1545480 | 0.050 | -5.080 | -0.372 | 206 | 6 | enet | 0.035 | 1.60E-04 | 4.79E-01 | 6.32E-01 |
| SSBP1 | 7 | 141438121 | 141487722 | 0.0321 | rs12374799 | 2.250 | rs2233998 | 0.054 | 4.870 | -0.409 | 94 | 10 | enet | 0.041 | 4.30E-05 | -4.79E-01 | 6.32E-01 |
| C1QBP | 17 | 5336097 | 5352150 | 0.0511 | rs8079518 | 1.590 | rs3026106 | 0.009 | 3.600 | -0.170 | 116 | 9 | enet | 0.020 | 3.50E-03 | -4.76E-01 | 6.34E-01 |
| GRWD1 | 19 | 48949030 | 48960279 | 0.2823 | rs1799286 | 1.300 | rs2302951 | 0.065 | -6.600 | -0.800 | 62 | 10 | enet | 0.180 | 2.60E-18 | 4.74E-01 | 6.36E-01 |
| GCLM | 1 | 94350761 | 94374966 | 0.0634 | rs743110 | -2.157 | rs11165030 | 0.038 | -4.540 | 0.263 | 103 | 8 | enet | 0.059 | 1.00E-06 | 4.73E-01 | 6.36E-01 |
| SIRT5 | 6 | 13574816 | 13614790 | 0.4380 | rs2841528 | 2.586 | rs2804918 | 0.085 | 7.150 | -0.209 | 93 | 30 | enet | 0.200 | 1.50E-20 | 4.72E-01 | 6.37E-01 |
| PFKL | 21 | 45719934 | 45747259 | 0.2211 | rs9983276 | 3.250 | rs3761392 | 0.126 | -7.350 | 0.394 | 87 | 7 | enet | 0.140 | 6.20E-14 | -4.72E-01 | 6.37E-01 |
| TAGLN3 | 3 | 111717511 | 111732734 | 0.0946 | rs13062225 | 2.470 | rs3749310 | 0.081 | -6.210 | 0.343 | 160 | 17 | enet | 0.100 | 1.40E-10 | -4.71E-01 | 6.38E-01 |
| ENDOG | 9 | 131580753 | 131584956 | 0.0599 | rs10739734 | 2.000 | rs2997922 | 0.056 | -5.140 | -0.237 | 58 | 10 | enet | 0.050 | 1.70E-05 | 4.70E-01 | 6.39E-01 |
| KIAA1324 | 1 | 109656301 | 109749401 | 0.0709 | rs17641881 | -2.069 | rs645128 | 0.059 | -5.690 | 0.076 | 126 | 9 | enet | 0.043 | 2.80E-05 | -4.70E-01 | 6.39E-01 |
| NCOA7 | 6 | 126102307 | 126252266 | 0.1314 | rs6906366 | 1.771 | rs10872303 | 0.042 | 4.840 | -0.171 | 111 | 14 | enet | 0.086 | 3.70E-09 | 4.69E-01 | 6.39E-01 |
| PSMF1 | 20 | 1093906 | 1160596 | 0.1888 | rs6078239 | 2.414 | rs6039915 | 0.117 | -6.900 | 1.023 | 155 | 19 | enet | 0.085 | 4.70E-09 | -4.69E-01 | 6.39E-01 |
| SFXN3 | 10 | 102790991 | 102800998 | 0.4923 | rs946327 | -2.240 | rs7099531 | 0.151 | -7.650 | -0.509 | 101 | 22 | enet | 0.270 | 4.10E-28 | -4.69E-01 | 6.39E-01 |
| ABCB6 | 2 | 220074490 | 220083712 | 0.0399 | rs3210652 | 1.701 | rs6710315 | 0.035 | 4.330 | -0.788 | 87 | 29 | enet | 0.021 | 2.80E-03 | 4.67E-01 | 6.41E-01 |
| PDLIM4 | 5 | 131593364 | 131609147 | 0.1722 | rs2077380 | 4.102 | rs3900945 | 0.261 | 10.120 | 0.558 | 123 | 24 | enet | 0.250 | 1.90E-25 | 4.67E-01 | 6.41E-01 |
| SH3GL2 | 9 | 17579080 | 17797127 | 0.0894 | rs7865066 | 3.520 | rs10756910 | 0.002 | 4.320 | -0.464 | 297 | 21 | enet | 0.005 | 9.70E-02 | -4.66E-01 | 6.41E-01 |
| NIPSNAP3B | 9 | 107526438 | 107539738 | 0.3299 | rs3818688 | 1.920 | rs10820726 | 0.447 | -13.430 | 0.501 | 133 | 11 | enet | 0.470 | 3.60E-53 | -4.64E-01 | 6.43E-01 |
| IDH1 | 2 | 209100951 | 209130798 | 0.0495 | rs999891 | 1.982 | rs6710217 | 0.055 | -4.750 | -0.431 | 93 | 3 | enet | 0.037 | 1.10E-04 | 4.64E-01 | 6.43E-01 |
| ERLIN1 | 10 | 101909851 | 101948091 | 0.3024 | rs11190401 | 1.640 | rs2862954 | 0.289 | -10.660 | -0.634 | 85 | 20 | enet | 0.280 | 6.60E-29 | 4.63E-01 | 6.43E-01 |
| PLXND1 | 3 | 129274018 | 129325661 | 0.2307 | rs10934884 | -2.250 | rs2811423 | 0.017 | -3.720 | -0.695 | 73 | 36 | enet | 0.075 | 3.90E-08 | 4.63E-01 | 6.44E-01 |
| UBA2 | 19 | 34919257 | 34960853 | 0.0423 | rs3787035 | 2.700 | rs7252100 | 0.042 | 4.430 | 0.520 | 89 | 3 | enet | 0.032 | 2.70E-04 | 4.62E-01 | 6.44E-01 |
| KIAA1715 | 2 | 176788620 | 176867567 | 0.1221 | rs35817516 | -2.129 | rs935406 | 0.046 | -4.490 | 2.018 | 127 | 17 | enet | 0.038 | 8.90E-05 | -4.62E-01 | 6.44E-01 |
| MYH14 | 19 | 50691443 | 50813802 | 0.1882 | rs2123852 | 2.310 | rs588973 | 0.044 | 5.220 | -0.305 | 104 | 17 | enet | 0.099 | 2.30E-10 | 4.62E-01 | 6.44E-01 |
| WARS2 | 1 | 119573839 | 119683294 | 0.4200 | rs12756326 | 2.013 | rs12029198 | 0.254 | -10.010 | 0.686 | 124 | 22 | enet | 0.290 | 1.20E-29 | -4.59E-01 | 6.46E-01 |
| PRKCA | 17 | 64298754 | 64806861 | 0.1804 | rs11867563 | 3.390 | rs6504459 | 0.053 | 6.310 | -1.756 | 426 | 32 | enet | 0.120 | 2.20E-12 | -4.59E-01 | 6.46E-01 |
| CSE1L | 20 | 47662849 | 47713489 | 0.1231 | rs6066932 | 0.920 | rs1556876 | 0.078 | -6.700 | -0.708 | 79 | 9 | enet | 0.140 | 2.70E-14 | 4.58E-01 | 6.47E-01 |
| WIPF3 | 7 | 29846102 | 29956682 | 0.1233 | rs10232672 | -1.300 | rs174917 | 0.080 | -6.280 | -0.277 | 135 | 10 | enet | 0.098 | 3.40E-10 | 4.58E-01 | 6.47E-01 |
| MGMT | 10 | 131265448 | 131566271 | 0.6087 | rs10764906 | -2.060 | rs1008982 | 0.225 | -9.270 | -0.857 | 286 | 73 | enet | 0.460 | 2.20E-52 | -4.57E-01 | 6.48E-01 |
| PTPN18 | 2 | 131113580 | 131132982 | 0.1257 | rs949771 | -0.887 | rs6713511 | 0.109 | -5.690 | 0.644 | 35 | 9 | enet | 0.100 | 2.20E-07 | -4.57E-01 | 6.48E-01 |
| TRAPPC12 | 2 | 3383446 | 3488865 | 0.0529 | rs17329506 | -1.989 | rs10205909 | -0.002 | -3.150 | 0.729 | 127 | 11 | enet | 0.007 | 6.10E-02 | -4.56E-01 | 6.48E-01 |

| | | | | | | | | | | | | | | | | | |
|---|---|---|---|---|---|---|---|---|---|---|---|---|---|---|---|---|---|
| SLC6A7 | 5 | 149569520 | 149602351 | 0.0945 | rs3756309 | -2.636 | rs4601032 | 0.054 | 4.840 | -1.035 | 148 | 14 | enet | 0.045 | 2.60E-05 | -4.56E-01 | 6.48E-01 |
| CLPB | 11 | 72003469 | 72145692 | 0.2647 | rs1784257 | 2.810 | rs12807696 | 0.126 | 7.570 | -0.214 | 93 | 3 | enet | 0.062 | 5.80E-07 | -4.57E-01 | 6.48E-01 |
| GCSH | 16 | 81115566 | 81130008 | 0.0404 | rs8060549 | 2.677 | rs4454990 | 0.048 | -5.270 | -0.630 | 174 | 10 | enet | 0.049 | 8.00E-06 | 4.56E-01 | 6.48E-01 |
| GRHPR | 9 | 37422663 | 37436987 | 0.0672 | rs744985 | -3.690 | rs7872991 | 0.030 | 4.470 | -0.129 | 116 | 17 | enet | 0.054 | 3.30E-06 | -4.55E-01 | 6.49E-01 |
| MRPL1 | 4 | 78783674 | 78873944 | 0.1463 | rs11097938 | 2.380 | rs7656101 | 0.130 | -7.510 | 0.510 | 109 | 17 | enet | 0.120 | 4.00E-12 | -4.52E-01 | 6.51E-01 |
| IDH2 | 15 | 90626277 | 90645736 | 0.0661 | rs950117 | 2.150 | rs2137891 | 0.036 | 4.580 | 0.350 | 85 | 8 | enet | 0.032 | 2.70E-04 | 4.51E-01 | 6.52E-01 |
| IQSEC3 | 12 | 175931 | 287626 | 0.0807 | rs9651889 | -2.620 | rs881194 | -0.001 | 3.540 | 0.450 | 121 | 27 | enet | 0.009 | 3.90E-02 | 4.51E-01 | 6.52E-01 |
| HBG2 | 11 | 5274420 | 5667019 | 0.2228 | rs2291845 | -3.100 | rs11036474 | 0.180 | 7.780 | 0.560 | 587 | 12 | enet | 0.180 | 1.10E-15 | 4.49E-01 | 6.54E-01 |
| DCXR | 17 | 79993012 | 79995608 | 0.0618 | rs11659000 | -1.670 | rs12936536 | 0.003 | 3.530 | -1.389 | 61 | 18 | enet | 0.012 | 1.90E-02 | -4.48E-01 | 6.54E-01 |
| FAM185A | 7 | 102389418 | 102449672 | 0.2220 | rs11981219 | -2.130 | rs7778418 | 0.069 | -6.160 | -1.223 | 62 | 21 | enet | 0.160 | 9.60E-16 | -4.47E-01 | 6.55E-01 |
| TMEM175 | 4 | 926175 | 952444 | 0.0652 | rs11946340 | 3.840 | rs873785 | 0.010 | 3.700 | -1.968 | 106 | 44 | enet | 0.005 | 1.30E-01 | -4.46E-01 | 6.56E-01 |
| GSTM5 | 1 | 110254864 | 110318050 | 0.4892 | rs11102002 | -2.673 | rs3754446 | 0.405 | -12.370 | 0.411 | 116 | 30 | enet | 0.510 | 1.30E-59 | 4.44E-01 | 6.57E-01 |
| EXOG | 3 | 38537618 | 38583652 | 0.1433 | rs7373102 | 2.080 | rs2186457 | 0.071 | 5.790 | -0.424 | 132 | 26 | enet | 0.087 | 3.10E-09 | 4.43E-01 | 6.58E-01 |
| PRKCD | 3 | 53190025 | 53226733 | 0.3681 | rs2253675 | -3.120 | rs750527 | 0.127 | -7.110 | -1.808 | 108 | 11 | enet | 0.180 | 1.30E-18 | 4.42E-01 | 6.58E-01 |
| TECPR2 | 14 | 102829300 | 102968818 | 0.0635 | rs17100811 | 2.770 | rs1190554 | 0.065 | 5.230 | 0.821 | 108 | 9 | enet | 0.038 | 8.70E-05 | 4.41E-01 | 6.59E-01 |
| PPP2R4 | 9 | 131873229 | 131911225 | 0.2432 | rs10819457 | -1.560 | rs3118634 | 0.153 | -7.730 | 0.344 | 88 | 43 | enet | 0.110 | 1.70E-11 | -4.41E-01 | 6.59E-01 |
| ALDH1A3 | 15 | 101417919 | 101456831 | 0.4066 | rs7163635 | -2.090 | rs11630508 | 0.175 | -8.300 | 0.968 | 123 | 13 | enet | 0.210 | 1.00E-20 | -4.41E-01 | 6.60E-01 |
| PACSIN2 | 22 | 43231418 | 43411151 | 0.1075 | rs5758941 | 2.820 | rs738535 | 0.043 | 5.870 | -1.081 | 178 | 18 | enet | 0.099 | 2.50E-10 | -4.40E-01 | 6.60E-01 |
| SYN3 | 22 | 32908539 | 33454358 | 0.2640 | rs2239765 | 4.040 | rs10483168 | 0.158 | -7.750 | 0.357 | 516 | 39 | enet | 0.230 | 7.60E-23 | 4.40E-01 | 6.60E-01 |
| RRM2B | 8 | 103216730 | 103251346 | 0.1479 | rs10505018 | 2.690 | rs1052071 | 0.177 | 8.300 | -0.838 | 109 | 15 | enet | 0.190 | 6.80E-19 | -4.39E-01 | 6.60E-01 |
| EFR3A | 8 | 132916335 | 133025889 | 0.0676 | rs7386782 | 3.050 | rs1501061 | 0.058 | -5.010 | 0.406 | 168 | 12 | enet | 0.017 | 6.20E-03 | 4.40E-01 | 6.60E-01 |
| PACS1 | 11 | 65837834 | 66012218 | 0.0523 | rs538954 | -5.260 | rs9326370 | 0.021 | 4.510 | 1.325 | 131 | 8 | enet | 0.037 | 1.00E-04 | 4.40E-01 | 6.60E-01 |
| SPARCL1 | 4 | 88394487 | 88452213 | 0.0529 | rs6532012 | 2.200 | rs10028788 | 0.049 | -4.850 | 0.296 | 122 | 11 | enet | 0.032 | 2.90E-04 | -4.40E-01 | 6.60E-01 |
| NIPA1 | 15 | 23043277 | 23100005 | 0.2176 | rs1991922 | -2.310 | rs6606825 | 0.063 | -5.480 | -0.755 | 83 | 27 | enet | 0.062 | 3.20E-06 | 4.39E-01 | 6.60E-01 |
| PHGDH | 1 | 120202421 | 120286838 | 0.2153 | rs478093 | -5.872 | rs10923897 | 0.068 | -5.690 | -1.001 | 141 | 31 | enet | 0.086 | 3.80E-09 | 4.38E-01 | 6.61E-01 |
| GGCT | 7 | 30536237 | 30591095 | 0.2678 | rs2709778 | -1.400 | rs38416 | 0.257 | 9.980 | -0.471 | 98 | 24 | enet | 0.280 | 6.80E-29 | -4.38E-01 | 6.61E-01 |
| SV2C | 5 | 75378997 | 75649764 | 0.0926 | rs875543 | -3.397 | rs4490566 | 0.074 | 6.150 | 0.147 | 235 | 13 | enet | 0.064 | 3.90E-07 | -4.37E-01 | 6.62E-01 |
| DUS2 | 16 | 68021649 | 68113223 | 0.0546 | rs4359427 | 3.018 | rs7196789 | 0.027 | 3.860 | 0.773 | 55 | 14 | enet | 0.016 | 1.30E-02 | 4.37E-01 | 6.62E-01 |
| EMILIN2 | 18 | 2847028 | 2915991 | 0.0954 | rs11661976 | 2.140 | rs642926 | 0.048 | -4.920 | 0.221 | 116 | 6 | enet | 0.050 | 1.10E-05 | 4.36E-01 | 6.63E-01 |
| MAN2B2 | 4 | 6576902 | 6625089 | 0.3595 | rs7654321 | -3.090 | rs2301795 | 0.214 | 8.730 | -0.687 | 106 | 41 | enet | 0.250 | 1.60E-22 | -4.35E-01 | 6.63E-01 |
| GAMT | 19 | 1397091 | 1401569 | 0.0896 | rs7249187 | -1.600 | rs11668809 | 0.042 | -5.370 | -0.838 | 62 | 5 | enet | 0.042 | 3.90E-05 | 4.35E-01 | 6.64E-01 |
| RBP1 | 3 | 139236276 | 139258671 | 0.1889 | rs9838798 | 2.390 | rs2071387 | 0.081 | 5.800 | 0.991 | 110 | 17 | enet | 0.096 | 4.90E-10 | 4.33E-01 | 6.65E-01 |
| MARC1 | 1 | 220960101 | 220987735 | 0.2623 | rs11118607 | -1.865 | rs2642442 | 0.194 | -8.700 | -0.522 | 136 | 10 | enet | 0.160 | 2.80E-16 | 4.32E-01 | 6.66E-01 |
| ECM1 | 1 | 150480538 | 150486265 | 0.0569 | rs4970966 | 3.762 | rs698916 | 0.083 | -5.710 | -0.028 | 53 | 8 | enet | 0.059 | 2.20E-06 | 4.31E-01 | 6.67E-01 |
| SH3BGRL2 | 6 | 80341000 | 80413372 | 0.1014 | rs12199053 | -3.037 | rs2295015 | 0.081 | 6.130 | -0.489 | 137 | 9 | enet | 0.081 | 1.20E-08 | -4.30E-01 | 6.68E-01 |
| DHTKD1 | 10 | 12110971 | 12165224 | 0.6485 | rs7899260 | 2.070 | rs3740015 | 0.524 | 13.630 | 0.277 | 99 | 30 | enet | 0.550 | 5.20E-63 | 4.29E-01 | 6.68E-01 |
| TRIM25 | 17 | 54965270 | 54991399 | 0.0983 | rs2525997 | 1.770 | rs2525994 | 0.105 | -6.590 | -0.033 | 73 | 8 | enet | 0.092 | 1.10E-09 | 4.26E-01 | 6.70E-01 |
| NUP210 | 3 | 13357737 | 13461809 | 0.0705 | rs12635063 | 1.560 | rs9874760 | 0.048 | 4.500 | 0.705 | 148 | 24 | enet | 0.030 | 4.20E-04 | 4.25E-01 | 6.71E-01 |
| OCLN | 5 | 68788119 | 68853931 | 0.1589 | rs6888874 | 0.582 | rs2583707 | 0.068 | -4.870 | -0.341 | 32 | 16 | enet | 0.043 | 8.70E-05 | 4.23E-01 | 6.72E-01 |
| TEFM | 17 | 29224354 | 29233838 | 0.2439 | rs6505211 | -3.310 | rs999797 | 0.220 | -6.950 | 0.757 | 50 | 28 | enet | 0.230 | 1.40E-13 | 4.24E-01 | 6.72E-01 |
| C15orf57 | 15 | 40820882 | 40857256 | 0.1025 | rs4923878 | 2.130 | rs11070283 | 0.072 | -4.900 | -0.828 | 81 | 13 | enet | 0.030 | 1.30E-03 | 4.22E-01 | 6.73E-01 |
| PCP4L1 | 1 | 161228517 | 161255240 | 0.0359 | rs9651049 | -4.285 | rs2502807 | -0.003 | -3.040 | 0.807 | 121 | 31 | enet | -0.002 | 7.10E-01 | -4.21E-01 | 6.74E-01 |
| TMEM231 | 16 | 75572015 | 75590184 | 0.0749 | rs42471 | 1.198 | rs11645180 | 0.050 | -5.050 | 0.687 | 93 | 10 | enet | 0.059 | 3.50E-05 | -4.20E-01 | 6.74E-01 |
| DISP2 | 15 | 40650436 | 40663257 | 0.0788 | rs936216 | 1.720 | rs16970633 | 0.065 | 4.640 | 0.624 | 99 | 7 | enet | 0.051 | 3.60E-05 | -4.20E-01 | 6.75E-01 |
| GPR180 | 13 | 95254157 | 95286899 | 0.0565 | rs1417735 | -2.000 | rs9524555 | 0.039 | -4.020 | 0.108 | 108 | 6 | enet | 0.024 | 3.00E-03 | -4.20E-01 | 6.75E-01 |
| HRAS | 11 | 532242 | 537287 | 0.0572 | rs7935013 | -2.990 | rs12806062 | 0.039 | 4.730 | -0.611 | 83 | 10 | enet | 0.041 | 4.80E-05 | -4.20E-01 | 6.75E-01 |

| | | | | | | | | | | | | | | | | | |
|---|---|---|---|---|---|---|---|---|---|---|---|---|---|---|---|---|---|
| RIC8A | 11 | 207511 | 215113 | 0.1552 | rs7102856 | -4.200 | rs473151 | 0.048 | -5.180 | 1.876 | 98 | 19 | enet | 0.089 | 2.00E-09 | -4.19E-01 | 6.75E-01 |
| C17orf80 | 17 | 71228372 | 71245091 | 0.0616 | rs17248554 | 2.060 | rs7211297 | 0.034 | 4.180 | -0.979 | 97 | 10 | enet | 0.047 | 1.20E-04 | -4.19E-01 | 6.75E-01 |
| YARS2 | 12 | 32880424 | 32908836 | 0.0592 | rs10844367 | -1.820 | rs11052168 | 0.084 | -6.120 | -0.459 | 117 | 26 | enet | 0.073 | 6.00E-08 | 4.19E-01 | 6.76E-01 |
| RAB23 | 6 | 57053607 | 57087078 | 0.0663 | rs13206250 | 3.196 | rs9296862 | 0.023 | -4.510 | -0.720 | 93 | 9 | enet | 0.056 | 2.20E-06 | -4.16E-01 | 6.77E-01 |
| GLO1 | 6 | 38643701 | 38670917 | 0.0702 | rs7769812 | -1.978 | rs1781735 | 0.084 | -6.200 | -0.746 | 106 | 9 | enet | 0.081 | 1.20E-08 | 4.17E-01 | 6.77E-01 |
| PMM2 | 16 | 8882680 | 8943188 | 0.2134 | rs13337024 | 2.101 | rs11074924 | 0.241 | 9.270 | 0.400 | 152 | 13 | enet | 0.230 | 5.30E-22 | 4.16E-01 | 6.77E-01 |
| MPZ | 1 | 161274525 | 161279762 | 0.4030 | rs9651049 | -4.285 | rs7550839 | 0.397 | 9.680 | 1.620 | 103 | 27 | enet | 0.400 | 2.70E-27 | 4.16E-01 | 6.78E-01 |
| EMB | 5 | 49692026 | 49739082 | 0.1273 | rs16879125 | 1.513 | rs2668024 | 0.083 | 6.240 | 0.277 | 53 | 15 | enet | 0.120 | 2.70E-10 | 4.14E-01 | 6.79E-01 |
| TXNDC5 | 6 | 7881483 | 8026646 | 0.0576 | rs197119 | 2.721 | rs9505293 | 0.033 | 4.360 | -0.543 | 236 | 8 | enet | 0.014 | 1.20E-02 | -4.14E-01 | 6.79E-01 |
| ALDH6A1 | 14 | 74523553 | 74551196 | 0.0456 | rs4903163 | 1.740 | rs2072293 | 0.057 | -4.960 | -0.230 | 101 | 5 | enet | 0.054 | 3.20E-06 | 4.14E-01 | 6.79E-01 |
| WIPI1 | 17 | 66417089 | 66453654 | 0.1436 | rs2302783 | 2.290 | rs918167 | 0.105 | 7.560 | 0.085 | 113 | 16 | enet | 0.200 | 2.70E-18 | -4.13E-01 | 6.79E-01 |
| OXLD1 | 17 | 79632066 | 79633665 | 0.1034 | rs9895741 | 2.390 | rs35646762 | 0.013 | -3.990 | 1.271 | 71 | 26 | enet | 0.025 | 1.30E-03 | -4.14E-01 | 6.79E-01 |
| SRSF9 | 12 | 120899471 | 120907596 | 0.2775 | rs17431717 | 4.040 | rs11065126 | 0.128 | -7.300 | 0.322 | 82 | 6 | enet | 0.120 | 2.90E-12 | -4.13E-01 | 6.80E-01 |
| GNAZ | 22 | 23412540 | 23467224 | 0.0356 | rs5751574 | -1.750 | rs3788337 | 0.005 | 3.700 | -0.404 | 107 | 7 | enet | 0.015 | 9.10E-03 | -4.11E-01 | 6.81E-01 |
| MICAL3 | 22 | 18270415 | 18507325 | 0.0978 | rs5747338 | 3.320 | rs424931 | -0.003 | -3.500 | -1.427 | 270 | 22 | enet | 0.021 | 3.00E-03 | 4.11E-01 | 6.81E-01 |
| EML4 | 2 | 42396490 | 42559688 | 0.1673 | rs963405 | -1.810 | rs1017476 | 0.018 | -4.200 | -0.826 | 176 | 28 | enet | 0.025 | 1.20E-03 | 4.09E-01 | 6.83E-01 |
| ESD | 13 | 47345391 | 47371367 | 0.3170 | rs842383 | -2.390 | rs8192892 | 0.172 | -9.030 | -0.348 | 163 | 16 | enet | 0.280 | 7.50E-29 | 4.08E-01 | 6.83E-01 |
| PLIN4 | 19 | 4502204 | 4517716 | 0.1120 | rs10469324 | 4.380 | rs892161 | 0.054 | 5.410 | 1.096 | 89 | 15 | enet | 0.081 | 1.10E-08 | -4.08E-01 | 6.84E-01 |
| S100A1 | 1 | 153600402 | 153604513 | 0.0348 | rs7549308 | -2.244 | rs11497739 | 0.053 | -5.120 | -0.082 | 64 | 5 | enet | 0.058 | 2.10E-06 | 4.07E-01 | 6.84E-01 |
| DPYSL5 | 2 | 27070615 | 27173219 | 0.1904 | rs714447 | -1.518 | rs41448746 | 0.082 | -6.520 | 0.265 | 87 | 36 | enet | 0.100 | 1.00E-10 | -4.05E-01 | 6.85E-01 |
| C15orf40 | 15 | 83657193 | 83680393 | 0.1727 | rs17295457 | 1.680 | rs4842860 | 0.251 | 9.920 | -0.363 | 69 | 11 | enet | 0.310 | 2.60E-32 | -4.05E-01 | 6.86E-01 |
| LARS | 5 | 145492601 | 145562223 | 0.0576 | rs758037 | -1.587 | rs17796870 | 0.088 | -6.410 | -0.426 | 87 | 7 | enet | 0.087 | 3.30E-09 | 4.04E-01 | 6.86E-01 |
| CD82 | 11 | 44585977 | 44641913 | 0.1666 | rs357924 | 3.030 | rs7935166 | 0.156 | 7.850 | 0.265 | 170 | 6 | enet | 0.140 | 3.70E-14 | 4.05E-01 | 6.86E-01 |
| TMEM245 | 9 | 111777432 | 111882225 | 0.1555 | rs614253 | -2.830 | rs1003346 | 0.147 | -7.650 | 0.599 | 154 | 25 | enet | 0.170 | 5.10E-17 | -4.02E-01 | 6.88E-01 |
| TMEM70 | 8 | 74884672 | 74895018 | 0.1210 | rs11787158 | -1.640 | rs12676883 | 0.082 | 5.750 | -0.629 | 63 | 17 | enet | 0.097 | 1.20E-09 | -4.02E-01 | 6.88E-01 |
| DBR1 | 3 | 137879854 | 137893791 | 0.0461 | rs9842193 | 1.670 | rs774807 | 0.058 | 4.790 | 0.252 | 64 | 6 | enet | 0.043 | 1.80E-04 | 4.01E-01 | 6.89E-01 |
| STX6 | 1 | 180941861 | 180992047 | 0.0562 | rs2297185 | 2.584 | rs6665134 | 0.037 | 4.840 | 0.352 | 116 | 23 | enet | 0.048 | 1.00E-05 | -4.00E-01 | 6.89E-01 |
| THEM4 | 1 | 151846060 | 151882284 | 0.2280 | rs9645406 | 2.473 | rs16833668 | 0.338 | -11.580 | -0.564 | 104 | 16 | enet | 0.370 | 7.70E-39 | 4.01E-01 | 6.89E-01 |
| FLOT2 | 17 | 27206353 | 27224697 | 0.3785 | rs1976165 | -2.920 | rs6505095 | 0.188 | -8.900 | -0.137 | 68 | 7 | enet | 0.220 | 3.50E-22 | 4.01E-01 | 6.89E-01 |
| IDNK | 9 | 86237964 | 86259045 | 0.0568 | rs10780628 | 2.950 | rs2811915 | 0.024 | -3.380 | 0.147 | 95 | 20 | enet | 0.010 | 5.10E-02 | 3.98E-01 | 6.90E-01 |
| C5orf22 | 5 | 31532373 | 31555165 | 0.0989 | rs11748613 | -1.840 | rs4867350 | -0.002 | -2.710 | -1.140 | 111 | 24 | enet | 0.037 | 1.70E-04 | -3.98E-01 | 6.91E-01 |
| C15orf27 | 15 | 76352178 | 76521462 | 0.1035 | rs4886755 | -2.370 | rs3784449 | 0.103 | -6.600 | 0.235 | 129 | 22 | enet | 0.088 | 3.70E-08 | -3.97E-01 | 6.92E-01 |
| ASRGL1 | 11 | 62104920 | 62160882 | 0.1915 | rs7125138 | 2.990 | rs2463833 | 0.285 | 10.660 | 0.319 | 119 | 20 | enet | 0.330 | 1.30E-34 | 3.94E-01 | 6.94E-01 |
| RPAP1 | 15 | 41809374 | 41836467 | 0.0827 | rs11635906 | -2.380 | rs1200348 | 0.035 | 4.310 | -1.985 | 79 | 11 | enet | 0.017 | 6.90E-03 | -3.93E-01 | 6.94E-01 |
| CSDC2 | 22 | 41956767 | 41973745 | 0.1785 | rs9611598 | 2.980 | rs9611613 | 0.221 | 9.000 | 0.326 | 43 | 13 | enet | 0.210 | 1.10E-19 | 3.93E-01 | 6.95E-01 |
| TDRD7 | 9 | 100174232 | 100258407 | 0.1216 | rs10817771 | -3.130 | rs10817606 | 0.141 | -7.150 | -0.695 | 142 | 13 | enet | 0.120 | 1.80E-11 | 3.92E-01 | 6.95E-01 |
| NEK4 | 3 | 52744800 | 52804965 | 0.0875 | rs3821831 | -2.570 | rs13064064 | 0.133 | -6.530 | -0.283 | 112 | 20 | enet | 0.120 | 6.70E-09 | 3.90E-01 | 6.96E-01 |
| PNKD | 2 | 219135115 | 219211516 | 0.0398 | rs17462354 | 1.656 | rs4324314 | 0.027 | 4.300 | 0.604 | 118 | 10 | enet | 0.038 | 8.10E-05 | 3.89E-01 | 6.97E-01 |
| CCDC25 | 8 | 27590835 | 27630170 | 0.0522 | rs12542107 | -1.760 | rs9797 | 0.014 | 4.480 | -0.551 | 150 | 32 | enet | 0.014 | 1.30E-02 | -3.89E-01 | 6.97E-01 |
| VPS4A | 16 | 69345259 | 69358949 | 0.0399 | rs8061222 | 2.803 | rs8058805 | 0.042 | 4.850 | -0.347 | 66 | 6 | enet | 0.031 | 3.20E-04 | -3.87E-01 | 6.99E-01 |
| S100A13 | 1 | 153591263 | 153606568 | 0.3505 | rs7549308 | -2.244 | rs9330298 | 0.534 | 14.510 | 0.929 | 68 | 16 | enet | 0.580 | 3.20E-72 | 3.84E-01 | 7.01E-01 |
| PIP4K2A | 10 | 22823778 | 23003484 | 0.3034 | rs11013010 | -2.690 | rs10828316 | 0.291 | -10.500 | -0.530 | 144 | 10 | enet | 0.270 | 4.30E-28 | 3.84E-01 | 7.01E-01 |
| GPD1L | 3 | 32147181 | 32210205 | 0.2198 | rs7624627 | -2.430 | rs6784980 | 0.191 | 8.690 | -0.251 | 120 | 9 | enet | 0.140 | 2.20E-14 | -3.84E-01 | 7.01E-01 |
| SEPTIN5 | 17 | 75276651 | 75496678 | 0.0763 | rs7221079 | 3.360 | rs312826 | 0.053 | -4.670 | -1.160 | 226 | 25 | enet | 0.024 | 1.60E-03 | 3.82E-01 | 7.02E-01 |
| PCYOX1 | 2 | 70484518 | 70508323 | 0.1710 | rs11900489 | 2.711 | rs2706762 | 0.083 | -6.020 | -0.349 | 86 | 12 | enet | 0.075 | 3.80E-08 | 3.78E-01 | 7.05E-01 |
| RRM1 | 11 | 4115937 | 4160106 | 0.0309 | rs894460 | -2.380 | rs1372804 | 0.014 | -3.820 | -0.087 | 101 | 9 | enet | 0.003 | 1.40E-01 | -3.79E-01 | 7.05E-01 |

| | | | | | | | | | | | | | | | | | |
|---|---|---|---|---|---|---|---|---|---|---|---|---|---|---|---|---|---|
| IVD | 15 | 40697686 | 40728146 | 0.2649 | rs624486 | 1.990 | rs10518693 | 0.233 | 9.410 | 0.320 | 86 | 23 | enet | 0.320 | 9.30E-34 | 3.78E-01 | 7.06E-01 |
| CRYZL1 | 21 | 34961647 | 35016232 | 0.0755 | rs9631188 | 2.650 | rs13050238 | 0.051 | -4.460 | -0.563 | 60 | 10 | enet | 0.053 | 3.80E-06 | 3.78E-01 | 7.06E-01 |
| TAC1 | 7 | 97361220 | 97369784 | 0.0427 | rs10242448 | -2.500 | rs2665466 | 0.009 | 3.960 | 0.118 | 90 | 14 | enet | 0.014 | 1.70E-02 | 3.77E-01 | 7.06E-01 |
| PLCG1 | 20 | 39765600 | 39811629 | 0.1362 | rs3795131 | -4.424 | rs6016505 | 0.087 | 7.150 | 2.136 | 56 | 15 | enet | 0.150 | 1.80E-15 | 3.74E-01 | 7.09E-01 |
| DNAJA4 | 15 | 78556428 | 78574538 | 0.2042 | rs16969711 | -2.620 | rs2037347 | 0.148 | 8.390 | 1.363 | 125 | 22 | enet | 0.250 | 8.80E-26 | -3.72E-01 | 7.10E-01 |
| GCDH | 19 | 13001840 | 13025021 | 0.4124 | rs3745647 | -2.940 | rs8012 | 0.216 | -9.080 | 0.278 | 70 | 12 | enet | 0.250 | 4.80E-25 | 3.71E-01 | 7.11E-01 |
| ATP13A2 | 1 | 17312453 | 17338423 | 0.0444 | rs7550829 | 3.778 | rs3738814 | 0.037 | -4.360 | 0.715 | 113 | 28 | enet | 0.010 | 3.30E-02 | -3.69E-01 | 7.12E-01 |
| PLD2 | 17 | 4710391 | 4726729 | 0.1363 | rs8078776 | -3.090 | rs3867173 | 0.079 | -5.880 | -0.445 | 97 | 16 | enet | 0.120 | 1.10E-11 | 3.70E-01 | 7.12E-01 |
| RBBP9 | 20 | 18467184 | 18477887 | 0.1223 | rs6132059 | -2.526 | rs12481256 | 0.038 | -5.230 | 0.196 | 94 | 21 | enet | 0.076 | 3.30E-08 | 3.69E-01 | 7.12E-01 |
| FRMD5 | 15 | 44162962 | 44487450 | 0.0273 | rs8037395 | 1.800 | rs2918945 | 0.042 | 4.680 | -0.365 | 121 | 3 | enet | 0.041 | 6.60E-05 | -3.69E-01 | 7.12E-01 |
| DCAF6 | 1 | 167905021 | 168045081 | 0.1457 | rs2071922 | 1.811 | rs11558511 | 0.084 | -5.940 | -0.107 | 108 | 10 | enet | 0.099 | 3.30E-10 | 3.67E-01 | 7.13E-01 |
| SMIM12 | 1 | 35178338 | 35325417 | 0.1428 | rs6699355 | 2.903 | rs659619 | 0.051 | -5.160 | -0.932 | 161 | 37 | enet | 0.075 | 3.70E-08 | -3.68E-01 | 7.13E-01 |
| MMAB | 12 | 109991542 | 110011679 | 0.3822 | rs1974712 | 1.700 | rs2302706 | 0.593 | -15.310 | 0.120 | 112 | 42 | enet | 0.640 | 2.10E-86 | -3.67E-01 | 7.14E-01 |
| HSCB | 22 | 29138019 | 29153503 | 0.0537 | rs9620811 | -4.610 | rs714191 | -0.001 | -3.230 | 0.756 | 63 | 24 | enet | 0.004 | 1.20E-01 | -3.66E-01 | 7.14E-01 |
| RLBP1 | 15 | 89753100 | 89764982 | 0.1249 | rs7168941 | 1.970 | rs2283433 | 0.226 | 9.300 | -0.034 | 97 | 8 | enet | 0.190 | 1.40E-19 | -3.66E-01 | 7.15E-01 |
| PIN1 | 19 | 9945933 | 9960358 | 0.0315 | rs11556087 | -1.570 | rs889122 | 0.005 | 3.400 | 0.259 | 45 | 11 | enet | 0.015 | 9.70E-03 | 3.66E-01 | 7.15E-01 |
| ABCA8 | 17 | 66863433 | 66951533 | 0.0619 | rs7215642 | -2.950 | rs1373067 | 0.044 | -4.290 | 0.095 | 140 | 13 | enet | 0.016 | 7.80E-03 | 3.65E-01 | 7.15E-01 |
| MRPL9 | 1 | 151732119 | 151736040 | 0.0625 | rs9645406 | 2.473 | rs8480 | 0.067 | -5.470 | -0.344 | 87 | 6 | enet | 0.061 | 8.70E-07 | 3.64E-01 | 7.16E-01 |
| PPIL1 | 6 | 36822603 | 36842800 | 0.4534 | rs236447 | -1.579 | rs236350 | 0.212 | -9.070 | -0.787 | 153 | 23 | enet | 0.250 | 5.80E-26 | 3.63E-01 | 7.16E-01 |
| CDS1 | 4 | 85504132 | 85572491 | 0.1185 | rs17366515 | -2.240 | rs17009168 | 0.003 | -3.880 | 1.080 | 90 | 12 | enet | 0.021 | 2.70E-03 | -3.63E-01 | 7.16E-01 |
| ALG8 | 11 | 77811982 | 77850706 | 0.1443 | rs11237385 | 3.070 | rs563453 | 0.028 | -4.160 | 0.305 | 114 | 12 | enet | 0.053 | 4.00E-04 | 3.62E-01 | 7.17E-01 |
| NT5DC3 | 12 | 104164231 | 104234975 | 0.3229 | rs2583237 | -3.360 | rs3751204 | 0.166 | -8.120 | 1.479 | 256 | 33 | enet | 0.250 | 1.20E-25 | -3.63E-01 | 7.17E-01 |
| TMEM43 | 3 | 14166440 | 14185179 | 0.1900 | rs12491469 | 2.230 | rs3796308 | 0.074 | -5.370 | -1.022 | 99 | 33 | enet | 0.053 | 5.30E-06 | 3.62E-01 | 7.18E-01 |
| STAMBP | 2 | 74056086 | 74100786 | 0.0899 | rs6724905 | 2.198 | rs17009396 | 0.071 | 5.230 | -0.004 | 114 | 13 | enet | 0.059 | 1.10E-06 | 3.61E-01 | 7.18E-01 |
| OLA1 | 2 | 174937175 | 175113426 | 0.0705 | rs10497424 | 3.033 | rs17239055 | 0.043 | 5.080 | 0.380 | 159 | 20 | enet | 0.056 | 2.20E-06 | 3.62E-01 | 7.18E-01 |
| ADSS | 1 | 244571796 | 244615436 | 0.0464 | rs3127468 | 2.699 | rs3123701 | 0.048 | -4.480 | 0.358 | 125 | 4 | enet | 0.033 | 2.10E-04 | -3.60E-01 | 7.19E-01 |
| FECH | 18 | 55215515 | 55254004 | 0.6413 | rs1968274 | 2.100 | rs2272783 | 0.309 | -10.910 | -0.081 | 160 | 18 | enet | 0.350 | 1.00E-36 | 3.57E-01 | 7.21E-01 |
| PAWR | 12 | 79968759 | 80084877 | 0.0591 | rs4842266 | -1.550 | rs11525598 | 0.062 | 4.580 | 0.020 | 79 | 8 | enet | 0.029 | 2.90E-03 | -3.55E-01 | 7.22E-01 |
| DDX18 | 2 | 118572226 | 118589955 | 0.0807 | rs13035453 | -2.367 | rs2060332 | 0.054 | 5.820 | 0.021 | 115 | 17 | enet | 0.086 | 4.20E-09 | -3.55E-01 | 7.23E-01 |
| LRMP | 12 | 25173936 | 25261268 | 0.0495 | rs7299023 | 2.520 | rs7303889 | 0.006 | 3.930 | 0.056 | 172 | 16 | enet | 0.048 | 2.30E-05 | 3.55E-01 | 7.23E-01 |
| MIPEP | 13 | 24304328 | 24463558 | 0.2054 | rs1886797 | 1.940 | rs11551114 | 0.226 | -10.000 | 0.478 | 232 | 15 | enet | 0.230 | 2.10E-23 | -3.54E-01 | 7.23E-01 |
| HSDL2 | 9 | 115142217 | 115234690 | 0.2276 | rs10817323 | 3.390 | rs7852741 | 0.160 | 7.770 | 0.073 | 102 | 16 | enet | 0.160 | 7.60E-16 | 3.54E-01 | 7.24E-01 |
| ADI1 | 2 | 3501132 | 3523507 | 0.1098 | rs10192909 | -1.545 | rs12052249 | 0.031 | -4.330 | -0.517 | 91 | 10 | enet | 0.050 | 1.50E-05 | -3.52E-01 | 7.25E-01 |
| CCBL2 | 1 | 89401456 | 89458636 | 0.5579 | rs17486391 | -1.854 | rs2765527 | 0.681 | -16.190 | 0.343 | 93 | 11 | enet | 0.710 | 2.60E-103 | -3.50E-01 | 7.26E-01 |
| ITGA1 | 5 | 52083730 | 52255040 | 0.0791 | rs350450 | -2.306 | rs152088 | 0.004 | 3.200 | 0.405 | 278 | 23 | enet | -0.001 | 4.10E-01 | 3.50E-01 | 7.26E-01 |
| PLEKHO2 | 15 | 65134088 | 65160206 | 0.0717 | rs2630502 | 2.870 | rs8027676 | 0.015 | -4.000 | 1.008 | 74 | 10 | enet | 0.025 | 1.30E-03 | -3.48E-01 | 7.28E-01 |
| SPR | 2 | 73114489 | 73119287 | 0.0564 | rs999494 | 2.439 | rs11693376 | 0.041 | 4.990 | 0.045 | 50 | 10 | enet | 0.027 | 7.40E-04 | 3.46E-01 | 7.29E-01 |
| PTPRJ | 11 | 48002113 | 48189670 | 0.0327 | rs7946766 | 2.710 | rs1875697 | 0.024 | -3.640 | 0.499 | 109 | 12 | enet | 0.017 | 6.80E-03 | 3.47E-01 | 7.29E-01 |
| MLEC | 12 | 121124672 | 121139667 | 0.0997 | rs7970406 | -1.960 | rs2948150 | 0.016 | 4.370 | 0.329 | 105 | 19 | enet | 0.060 | 9.50E-07 | -3.44E-01 | 7.31E-01 |
| PTPRA | 20 | 2844830 | 3019722 | 0.0341 | rs11699077 | -1.684 | rs3818605 | 0.004 | -3.920 | 0.276 | 125 | 5 | enet | 0.012 | 1.90E-02 | -3.44E-01 | 7.31E-01 |
| TXNRD3 | 3 | 126290622 | 126373998 | 0.0978 | rs13313873 | 1.790 | rs708908 | -0.004 | 3.630 | 0.738 | 139 | 11 | enet | 0.004 | 1.70E-01 | 3.43E-01 | 7.32E-01 |
| FAHD1 | 16 | 1876968 | 1890208 | 0.0499 | rs1742398 | -1.406 | rs1178436 | 0.041 | -5.180 | 0.185 | 122 | 16 | enet | 0.059 | 1.10E-06 | 3.43E-01 | 7.32E-01 |
| PPOX | 1 | 161136200 | 161147803 | 0.3029 | rs2501875 | -3.851 | rs36013429 | 0.277 | -10.270 | -0.192 | 115 | 11 | enet | 0.300 | 4.60E-31 | 3.39E-01 | 7.34E-01 |
| C1orf27 | 1 | 186344890 | 186390510 | 0.3428 | rs2383477 | -2.356 | rs1547084 | 0.096 | 6.210 | -1.296 | 80 | 31 | enet | 0.150 | 4.80E-15 | -3.39E-01 | 7.35E-01 |
| CHMP3 | 2 | 86730554 | 86948245 | 0.0310 | rs12613610 | 1.779 | rs17027270 | 0.023 | 3.680 | -0.023 | 139 | 12 | enet | 0.009 | 4.10E-02 | -3.35E-01 | 7.38E-01 |
| CPNE8 | 12 | 39040624 | 39301232 | 0.0542 | rs12423647 | 2.020 | rs9325144 | 0.023 | -3.980 | 0.396 | 144 | 19 | enet | 0.038 | 9.00E-05 | 3.34E-01 | 7.38E-01 |

| | | | | | | | | | | | | | | | | | |
|---|---|---|---|---|---|---|---|---|---|---|---|---|---|---|---|---|---|
| TIMM44 | 19 | 7991603 | 8008805 | 0.2861 | rs759817 | 2.500 | rs11542187 | 0.104 | -6.460 | 1.701 | 72 | 17 | enet | 0.140 | 7.50E-14 | -3.34E-01 | 7.38E-01 |
| NUDT15 | 13 | 48611703 | 48621358 | 0.5997 | rs9526450 | -1.990 | rs2094196 | 0.012 | -3.680 | -0.143 | 75 | 48 | enet | 0.029 | 1.60E-03 | -3.33E-01 | 7.40E-01 |
| TPBG | 6 | 83072923 | 83080545 | 0.0702 | rs2983877 | -1.844 | rs9449462 | 0.053 | -5.170 | 1.326 | 58 | 14 | enet | 0.055 | 2.30E-06 | -3.33E-01 | 7.40E-01 |
| MOGS | 2 | 74688184 | 74692537 | 0.0564 | rs2268426 | -1.413 | rs2268417 | 0.039 | 4.280 | -0.293 | 59 | 11 | enet | 0.040 | 5.10E-05 | -3.30E-01 | 7.41E-01 |
| ARHGAP21 | 10 | 24872538 | 25012597 | 0.0586 | rs1572364 | -2.130 | rs1888657 | 0.014 | 4.020 | 0.187 | 154 | 12 | enet | 0.019 | 4.20E-03 | -3.30E-01 | 7.42E-01 |
| DLG1 | 3 | 196769431 | 197026171 | 0.2138 | rs4916453 | -2.790 | rs383443 | 0.047 | -5.980 | 0.228 | 209 | 17 | enet | 0.160 | 2.80E-16 | -3.28E-01 | 7.43E-01 |
| MTHFD1L | 6 | 151186685 | 151423023 | 0.1179 | rs9322300 | -2.934 | rs9397029 | 0.001 | -3.340 | -0.396 | 286 | 18 | enet | 0.027 | 7.70E-04 | 3.28E-01 | 7.43E-01 |
| SLC12A6 | 15 | 34525460 | 34630261 | 0.0826 | rs8039233 | 1.870 | rs2169480 | 0.010 | -3.300 | 0.210 | 107 | 21 | enet | 0.010 | 2.90E-02 | -3.27E-01 | 7.44E-01 |
| SUSD2 | 22 | 24577227 | 24585078 | 0.0663 | rs35676668 | 3.030 | rs35146655 | 0.071 | 4.570 | -0.030 | 65 | 24 | enet | 0.061 | 9.40E-05 | -3.27E-01 | 7.44E-01 |
| LYRM4 | 6 | 5102827 | 5261172 | 0.3359 | rs6926122 | 2.223 | rs2753248 | 0.409 | -12.530 | 0.734 | 218 | 13 | enet | 0.410 | 2.00E-44 | -3.25E-01 | 7.45E-01 |
| SERPINB8 | 18 | 61637159 | 61672278 | 0.2761 | rs4564691 | -1.810 | rs2162352 | 0.107 | 5.910 | 0.295 | 133 | 12 | enet | 0.110 | 3.50E-10 | 3.25E-01 | 7.46E-01 |
| SEPTIN3 | 22 | 42372276 | 42394225 | 0.0849 | rs1052717 | -2.730 | rs133333 | 0.013 | 3.740 | -0.177 | 81 | 20 | enet | 0.029 | 5.60E-04 | 3.23E-01 | 7.47E-01 |
| RANBP6 | 9 | 6011043 | 6015618 | 0.0292 | rs10758732 | 2.700 | rs10815320 | 0.028 | -4.270 | -1.098 | 67 | 6 | enet | 0.028 | 5.90E-04 | 3.22E-01 | 7.47E-01 |
| PLCXD3 | 5 | 41307056 | 41510730 | 0.0527 | rs318060 | 2.863 | rs3844180 | 0.076 | 5.470 | 0.254 | 186 | 5 | enet | 0.051 | 5.70E-06 | 3.21E-01 | 7.48E-01 |
| EML1 | 14 | 100204030 | 100408397 | 0.1165 | rs1191005 | -2.160 | rs732787 | 0.066 | -5.600 | 0.421 | 180 | 21 | enet | 0.053 | 3.50E-06 | -3.21E-01 | 7.48E-01 |
| C2orf69 | 2 | 200775979 | 200820658 | 0.3245 | rs11694369 | -4.043 | rs16832017 | 0.247 | -9.800 | -0.163 | 83 | 22 | enet | 0.230 | 9.00E-23 | 3.21E-01 | 7.49E-01 |
| ATE1 | 10 | 123499939 | 123688316 | 0.1919 | rs10886998 | -1.950 | rs7092052 | 0.189 | -9.040 | -0.118 | 210 | 32 | enet | 0.240 | 6.80E-25 | -3.20E-01 | 7.49E-01 |
| IGLON5 | 19 | 51815102 | 51833608 | 0.1529 | rs11084071 | 2.410 | rs273628 | 0.060 | 5.140 | 0.175 | 113 | 17 | enet | 0.063 | 4.50E-07 | -3.19E-01 | 7.50E-01 |
| UFL1 | 6 | 96969471 | 97003152 | 0.0560 | rs4365934 | 1.903 | rs2983897 | 0.060 | 5.870 | 0.591 | 79 | 22 | enet | 0.040 | 5.80E-05 | 3.19E-01 | 7.50E-01 |
| GDAP2 | 1 | 118406107 | 118472253 | 0.0926 | rs6428716 | -1.939 | rs12031260 | 0.022 | -3.710 | 1.191 | 91 | 58 | enet | 0.029 | 5.50E-04 | 3.17E-01 | 7.51E-01 |
| ZMPSTE24 | 1 | 40723779 | 40759856 | 0.0967 | rs364798 | -1.644 | rs6682701 | 0.137 | -7.250 | -0.220 | 89 | 4 | enet | 0.120 | 3.50E-12 | 3.14E-01 | 7.54E-01 |
| SORBS3 | 8 | 22402499 | 22433301 | 0.0807 | rs2469749 | 2.130 | rs11779261 | 0.003 | 3.780 | -0.368 | 120 | 17 | enet | 0.015 | 1.00E-02 | -3.14E-01 | 7.54E-01 |
| NKIRAS1 | 3 | 23933151 | 23988082 | 0.0552 | rs7611018 | 2.020 | rs8933 | 0.010 | 4.060 | -0.117 | 94 | 7 | enet | 0.034 | 2.00E-04 | -3.10E-01 | 7.56E-01 |
| NTM | 11 | 131240373 | 132206716 | 0.2453 | rs564994 | 2.940 | rs318977 | 0.115 | -6.710 | 0.547 | 803 | 41 | enet | 0.120 | 1.30E-12 | 3.08E-01 | 7.58E-01 |
| EPS8 | 12 | 15773092 | 16035263 | 0.0324 | rs7137185 | 2.430 | rs10459102 | 0.035 | -3.980 | 0.168 | 101 | 10 | enet | 0.028 | 6.60E-04 | -3.08E-01 | 7.58E-01 |
| TGOLN2 | 2 | 85545147 | 85555548 | 0.0719 | rs6547602 | 2.664 | rs4247303 | 0.136 | -7.310 | 0.273 | 112 | 3 | enet | 0.120 | 2.00E-12 | -3.07E-01 | 7.59E-01 |
| SAMD4A | 14 | 55033815 | 55260033 | 0.3867 | rs17127785 | 2.540 | rs17657012 | 0.353 | -9.100 | -0.168 | 219 | 13 | enet | 0.340 | 2.20E-23 | 3.05E-01 | 7.60E-01 |
| KRR1 | 12 | 75890684 | 75905416 | 0.0790 | rs4565938 | -1.100 | rs2070162 | 0.073 | -4.800 | 0.239 | 91 | 17 | enet | 0.034 | 1.20E-03 | -3.04E-01 | 7.61E-01 |
| COX11 | 17 | 53029263 | 53046146 | 0.0896 | rs16955299 | -1.500 | rs17817829 | 0.091 | -5.590 | -0.584 | 106 | 17 | enet | 0.092 | 4.50E-08 | 3.03E-01 | 7.62E-01 |
| CLN5 | 13 | 77564795 | 77576652 | 0.1014 | rs2285388 | 2.130 | rs593502 | -0.002 | -3.620 | 1.036 | 96 | 14 | enet | 0.031 | 2.00E-03 | -3.02E-01 | 7.63E-01 |
| UNC45A | 15 | 91473410 | 91497323 | 0.0637 | rs7171099 | 2.710 | rs12148829 | 0.037 | 4.760 | 0.334 | 125 | 19 | enet | 0.043 | 3.80E-05 | 3.01E-01 | 7.64E-01 |
| PSEN1 | 14 | 73603126 | 73690399 | 0.0955 | rs177369 | -1.980 | rs17781801 | 0.011 | -5.460 | 0.065 | 87 | 12 | enet | 0.015 | 9.30E-03 | -2.99E-01 | 7.65E-01 |
| GFRA1 | 10 | 117816444 | 118032979 | 0.2469 | rs2251545 | 2.450 | rs12570087 | 0.160 | 7.760 | 0.570 | 250 | 33 | enet | 0.230 | 4.80E-21 | -2.98E-01 | 7.66E-01 |
| SH3PXD2B | 5 | 171752185 | 171881527 | 0.2240 | rs41495146 | 2.032 | rs2731688 | 0.094 | 6.870 | 0.915 | 170 | 45 | enet | 0.150 | 4.10E-15 | 2.98E-01 | 7.66E-01 |
| TMCC3 | 12 | 94960900 | 95044338 | 0.0551 | rs1316366 | -3.310 | rs7962496 | -0.002 | 3.370 | -0.277 | 191 | 18 | enet | 0.000 | 3.60E-01 | 2.98E-01 | 7.66E-01 |
| ANKMY2 | 7 | 16639401 | 16685442 | 0.2006 | rs17627827 | -2.640 | rs7797003 | 0.185 | 8.520 | 0.296 | 171 | 7 | enet | 0.160 | 9.00E-16 | 2.96E-01 | 7.67E-01 |
| TTC19 | 17 | 15902694 | 15948329 | 0.0325 | rs12603728 | -2.030 | rs758853 | 0.017 | -3.890 | 0.198 | 79 | 17 | enet | 0.025 | 1.20E-03 | 2.96E-01 | 7.67E-01 |
| MOCS1 | 6 | 39867354 | 39902290 | 0.0834 | rs2475490 | 2.922 | rs1923483 | 0.014 | 4.020 | 1.469 | 144 | 17 | enet | 0.055 | 2.60E-06 | 2.93E-01 | 7.69E-01 |
| CLGN | 4 | 141309609 | 141349122 | 0.2988 | rs1877410 | 2.080 | rs2668585 | 0.175 | -8.070 | 0.664 | 102 | 26 | enet | 0.170 | 9.80E-17 | -2.93E-01 | 7.69E-01 |
| EFTUD1 | 15 | 82422571 | 82555104 | 0.1162 | rs2665086 | -1.890 | rs2654206 | 0.065 | 5.810 | -0.235 | 128 | 28 | enet | 0.056 | 2.10E-06 | -2.92E-01 | 7.70E-01 |
| PLEKHM2 | 1 | 16010827 | 16061264 | 0.0344 | rs2271545 | 3.392 | rs6701500 | 0.027 | -3.840 | -0.247 | 88 | 11 | enet | 0.022 | 2.50E-03 | 2.90E-01 | 7.71E-01 |
| PLEKHF2 | 8 | 96146032 | 96168912 | 0.0943 | rs7839455 | 2.520 | rs879999 | 0.010 | -3.800 | 0.472 | 129 | 16 | enet | 0.024 | 1.60E-03 | 2.91E-01 | 7.71E-01 |
| CERS4 | 19 | 8271620 | 8327305 | 0.1353 | rs1007545 | -2.940 | rs36259 | 0.093 | 5.650 | -1.190 | 97 | 9 | enet | 0.088 | 1.90E-08 | -2.91E-01 | 7.71E-01 |
| FNBP1 | 9 | 132649466 | 132805473 | 0.0614 | rs6478949 | -2.270 | rs4836675 | 0.063 | 5.620 | -0.502 | 158 | 11 | enet | 0.038 | 9.10E-05 | -2.90E-01 | 7.71E-01 |
| PARS2 | 1 | 55222571 | 55230187 | 0.1360 | rs558184 | -2.187 | rs17515141 | 0.094 | -6.070 | 0.076 | 133 | 48 | enet | 0.076 | 3.00E-08 | -2.89E-01 | 7.72E-01 |
| ENTPD2 | 9 | 139942550 | 139948497 | 0.1659 | rs3814499 | -2.480 | rs7388830 | 0.081 | 5.480 | -0.350 | 91 | 2 | enet | 0.069 | 4.20E-07 | -2.89E-01 | 7.72E-01 |

| | | | | | | | | | | | | | | | | | |
|---|---|---|---|---|---|---|---|---|---|---|---|---|---|---|---|---|---|
| NUAK1 | 12 | 106457118 | 106533811 | 0.0787 | rs2544118 | -2.820 | rs10746054 | 0.058 | 5.010 | -0.662 | 185 | 10 | enet | 0.035 | 1.60E-04 | -2.88E-01 | 7.73E-01 |
| ARHGEF37 | 5 | 148931510 | 149014531 | 0.0817 | rs17654288 | -1.834 | rs10037341 | 0.074 | 5.430 | 0.372 | 136 | 14 | enet | 0.076 | 2.00E-07 | 2.86E-01 | 7.75E-01 |
| MAP6 | 11 | 75297963 | 75380165 | 0.1554 | rs1790144 | 4.430 | rs655036 | 0.047 | 5.730 | 1.577 | 143 | 15 | enet | 0.075 | 4.10E-08 | -2.86E-01 | 7.75E-01 |
| ABHD10 | 3 | 111697857 | 111712210 | 0.2040 | rs13062225 | 2.470 | rs17429033 | 0.217 | -9.110 | 0.480 | 157 | 28 | enet | 0.200 | 1.10E-19 | -2.81E-01 | 7.79E-01 |
| FSD1L | 9 | 108210077 | 108314714 | 0.1303 | rs4538947 | 1.880 | rs12555267 | 0.009 | -4.870 | -0.373 | 95 | 21 | enet | 0.090 | 1.60E-09 | 2.79E-01 | 7.80E-01 |
| PNP | 14 | 20937113 | 20945253 | 0.0751 | rs2228036 | 2.800 | rs1049564 | 0.037 | -4.830 | -0.086 | 169 | 5 | enet | 0.016 | 7.90E-03 | -2.78E-01 | 7.81E-01 |
| FARP2 | 2 | 242295658 | 242434256 | 0.1528 | rs16843642 | -2.599 | rs3771555 | 0.074 | 6.360 | -1.442 | 121 | 20 | enet | 0.150 | 1.00E-12 | -2.77E-01 | 7.82E-01 |
| UVRAG | 11 | 75526212 | 75854239 | 0.0921 | rs2447947 | -3.170 | rs10793135 | -0.001 | -3.380 | 0.444 | 194 | 46 | enet | 0.003 | 1.50E-01 | -2.76E-01 | 7.82E-01 |
| TRNT1 | 3 | 3168600 | 3192563 | 0.0995 | rs4322988 | 3.240 | rs3804783 | 0.092 | 6.130 | 0.203 | 187 | 15 | enet | 0.074 | 4.70E-08 | 2.75E-01 | 7.84E-01 |
| MCEE | 2 | 71336814 | 71357369 | 0.5875 | rs4852772 | -3.070 | rs6725113 | 0.464 | -13.680 | -0.453 | 112 | 26 | enet | 0.520 | 2.60E-62 | 2.74E-01 | 7.84E-01 |
| NPM2 | 8 | 21881636 | 21894408 | 0.3242 | rs1552286 | -3.440 | rs900781 | 0.003 | 3.570 | 0.582 | 89 | 17 | enet | 0.076 | 6.00E-08 | 2.71E-01 | 7.87E-01 |
| ERC1 | 12 | 1099675 | 1605099 | 0.0499 | rs2240506 | 1.780 | rs10773931 | 0.016 | -4.330 | 1.727 | 265 | 6 | enet | 0.002 | 1.70E-01 | -2.70E-01 | 7.87E-01 |
| XRCC6 | 22 | 42017123 | 42060044 | 0.0262 | rs17002515 | -1.780 | rs4822049 | 0.026 | -3.720 | 0.425 | 48 | 4 | enet | 0.014 | 1.20E-02 | -2.70E-01 | 7.87E-01 |
| PI16 | 6 | 36922209 | 36932613 | 0.4186 | rs9380633 | 1.907 | rs17624006 | 0.201 | -8.850 | 0.023 | 143 | 37 | enet | 0.260 | 2.40E-26 | 2.66E-01 | 7.90E-01 |
| MSTO1 | 1 | 155579979 | 155718153 | 0.0338 | rs12407219 | -0.538 | rs2175391 | 0.046 | -4.390 | 0.273 | 40 | 4 | enet | 0.034 | 2.10E-04 | -2.66E-01 | 7.91E-01 |
| CCZ1B | 7 | 6833765 | 6866401 | 0.1154 | rs6463577 | 1.760 | rs4724860 | 0.041 | -5.140 | -0.244 | 29 | 17 | enet | 0.011 | 2.40E-02 | 2.63E-01 | 7.93E-01 |
| HAAO | 2 | 42994229 | 43019733 | 0.1090 | rs2304661 | -2.035 | rs6719977 | 0.084 | -4.970 | 0.546 | 125 | 6 | enet | 0.100 | 7.00E-07 | -2.63E-01 | 7.93E-01 |
| RFK | 9 | 79000433 | 79009433 | 0.0936 | rs2803418 | -2.250 | rs10116800 | 0.060 | -5.360 | -0.351 | 122 | 10 | enet | 0.052 | 6.30E-06 | -2.60E-01 | 7.95E-01 |
| QRSL1 | 6 | 107077453 | 107116292 | 0.0980 | rs11968064 | 1.836 | rs898896 | 0.044 | 6.010 | 0.131 | 160 | 13 | enet | 0.093 | 8.40E-10 | -2.60E-01 | 7.95E-01 |
| CAPRIN1 | 11 | 34073230 | 34122703 | 0.0550 | rs4756111 | 1.950 | rs7925202 | -0.003 | 2.850 | -0.187 | 89 | 16 | enet | 0.003 | 1.60E-01 | 2.59E-01 | 7.95E-01 |
| SDSL | 12 | 113860042 | 113876081 | 0.0364 | rs4766687 | 1.980 | rs7295347 | 0.039 | 4.690 | 0.299 | 65 | 10 | enet | 0.022 | 2.10E-03 | 2.60E-01 | 7.95E-01 |
| PAPLN | 14 | 73704205 | 73741348 | 0.0619 | rs177369 | -1.980 | rs177374 | 0.007 | 3.660 | 0.448 | 83 | 17 | enet | 0.025 | 1.40E-03 | 2.59E-01 | 7.95E-01 |
| CHGB | 20 | 5892076 | 5906007 | 0.0849 | rs6085298 | 2.083 | rs2821 | 0.037 | -4.380 | 0.083 | 155 | 11 | enet | 0.012 | 2.00E-02 | 2.59E-01 | 7.96E-01 |
| ARL6IP5 | 3 | 69134095 | 69155217 | 0.0914 | rs9868056 | -3.050 | rs7038 | 0.056 | -4.740 | -1.393 | 74 | 13 | enet | 0.038 | 9.10E-05 | 2.58E-01 | 7.97E-01 |
| LGALS8 | 1 | 236681300 | 236716281 | 0.0886 | rs10802536 | -2.577 | rs12069618 | 0.006 | 4.300 | 0.839 | 167 | 13 | enet | 0.015 | 1.00E-02 | -2.57E-01 | 7.97E-01 |
| PITRM1 | 10 | 3179920 | 3215003 | 0.4635 | rs11251813 | -2.650 | rs4880592 | 0.363 | -11.740 | -0.253 | 202 | 40 | enet | 0.550 | 4.50E-68 | 2.57E-01 | 7.97E-01 |
| FAM98C | 19 | 38893775 | 38899728 | 0.1044 | rs10409638 | 2.360 | rs3745964 | 0.016 | -4.220 | 0.514 | 100 | 29 | enet | 0.079 | 4.00E-07 | -2.55E-01 | 7.98E-01 |
| TMEM109 | 11 | 60681346 | 60690915 | 0.1482 | rs2074230 | 6.930 | rs7940551 | -0.003 | 2.760 | 1.214 | 125 | 23 | enet | 0.025 | 1.40E-03 | 2.55E-01 | 7.99E-01 |
| CPPED1 | 16 | 12756919 | 12897874 | 0.0793 | rs11860844 | 2.761 | rs1541807 | -0.003 | 3.320 | 0.935 | 269 | 17 | enet | -0.003 | 9.70E-01 | -2.55E-01 | 7.99E-01 |
| NUDT19 | 19 | 33182867 | 33204702 | 0.2944 | rs17692896 | 2.750 | rs3892630 | 0.272 | -10.000 | -0.026 | 93 | 18 | enet | 0.270 | 9.70E-26 | 2.54E-01 | 7.99E-01 |
| MPP7 | 10 | 28339922 | 28623415 | 0.0590 | rs1249284 | 2.560 | rs786399 | 0.006 | -4.000 | 0.134 | 262 | 10 | enet | 0.009 | 3.60E-02 | 2.52E-01 | 8.01E-01 |
| C16orf13 | 16 | 684429 | 686358 | 0.3118 | rs4984902 | -2.593 | rs4984684 | 0.034 | 4.270 | -1.844 | 90 | 29 | enet | 0.095 | 5.80E-10 | -2.52E-01 | 8.01E-01 |
| NMRAL1 | 16 | 4511681 | 4545764 | 0.5757 | rs1291695 | -2.138 | rs11557236 | 0.205 | -8.770 | 0.205 | 71 | 9 | enet | 0.260 | 2.90E-26 | -2.51E-01 | 8.01E-01 |
| PNMAL2 | 19 | 46990163 | 46999755 | 0.1010 | rs3745792 | 3.380 | rs6509283 | 0.057 | -5.600 | 0.879 | 89 | 11 | enet | 0.080 | 1.40E-08 | -2.51E-01 | 8.02E-01 |
| RASA1 | 5 | 86563705 | 86687748 | 0.2723 | rs6870700 | 1.449 | rs13157168 | 0.132 | -7.150 | 0.074 | 69 | 8 | enet | 0.130 | 4.80E-13 | 2.51E-01 | 8.02E-01 |
| ASPHD1 | 16 | 29911696 | 29931185 | 0.3004 | rs12716974 | -5.118 | rs4787484 | 0.151 | 7.550 | 1.689 | 77 | 16 | enet | 0.170 | 2.60E-16 | 2.51E-01 | 8.02E-01 |
| GUCY1A3 | 4 | 156587863 | 156653501 | 0.1037 | rs6841458 | -2.690 | rs3796582 | 0.009 | 3.850 | 0.837 | 135 | 22 | enet | 0.027 | 7.80E-04 | -2.50E-01 | 8.03E-01 |
| TIMM21 | 18 | 71815746 | 71826197 | 0.1800 | rs1788580 | 2.390 | rs12956301 | 0.181 | 8.410 | 0.921 | 109 | 12 | enet | 0.180 | 1.20E-17 | 2.50E-01 | 8.03E-01 |
| LYRM2 | 6 | 90277863 | 90348472 | 0.2322 | rs9451243 | 3.721 | rs34012596 | 0.235 | -8.330 | 0.088 | 111 | 4 | enet | 0.210 | 6.70E-17 | 2.48E-01 | 8.04E-01 |
| CST3 | 20 | 23608534 | 23619110 | 0.0662 | rs13037020 | 2.636 | rs7266357 | 0.019 | -3.980 | 0.187 | 103 | 10 | enet | 0.015 | 9.90E-03 | 2.45E-01 | 8.06E-01 |
| EML2 | 19 | 46110252 | 46148887 | 0.1043 | rs2287019 | -3.450 | rs3816046 | 0.081 | -6.660 | 0.387 | 87 | 7 | enet | 0.100 | 1.90E-10 | 2.45E-01 | 8.06E-01 |
| FAM162B | 6 | 117073363 | 117086886 | 0.3098 | rs11153626 | -2.650 | rs654128 | 0.184 | -8.340 | 0.120 | 51 | 14 | enet | 0.190 | 1.10E-17 | -2.44E-01 | 8.07E-01 |
| MRAS | 3 | 138066539 | 138124375 | 0.1036 | rs6807945 | 3.300 | rs7626349 | 0.091 | -6.400 | -0.863 | 81 | 16 | enet | 0.100 | 2.80E-10 | 2.44E-01 | 8.07E-01 |
| ARMC6 | 19 | 19144384 | 19170563 | 0.3556 | rs7257072 | -3.750 | rs7253352 | 0.004 | -2.980 | 2.864 | 70 | 34 | enet | 0.063 | 4.90E-07 | -2.44E-01 | 8.08E-01 |
| DCK | 4 | 71858255 | 71896631 | 0.1600 | rs13116869 | -1.820 | rs6826930 | 0.115 | -7.220 | 0.042 | 63 | 9 | enet | 0.130 | 5.90E-13 | -2.42E-01 | 8.09E-01 |
| ALDH1B1 | 9 | 38392661 | 38398658 | 0.7296 | rs13290293 | -2.410 | rs2228093 | 0.333 | -11.260 | -0.021 | 138 | 42 | enet | 0.480 | 8.30E-56 | 2.41E-01 | 8.09E-01 |

| | | | | | | | | | | | | | | | | | |
|---|---|---|---|---|---|---|---|---|---|---|---|---|---|---|---|---|---|
| CPVL | 7 | 29034847 | 29235067 | 0.3466 | rs2286992 | 2.480 | rs245883 | 0.166 | 7.290 | 0.149 | 283 | 50 | enet | 0.240 | 1.60E-20 | -2.41E-01 | 8.10E-01 |
| CARHSP1 | 16 | 8946799 | 8962866 | 0.1037 | rs13337024 | 2.101 | rs2437704 | 0.072 | 5.500 | 0.035 | 126 | 14 | enet | 0.076 | 2.90E-08 | 2.40E-01 | 8.10E-01 |
| ASS1 | 9 | 133320316 | 133376661 | 0.2895 | rs2082293 | 2.460 | rs877326 | 0.066 | 6.790 | -0.318 | 145 | 43 | enet | 0.200 | 2.20E-20 | -2.40E-01 | 8.10E-01 |
| HINT3 | 6 | 126277927 | 126301387 | 0.0208 | rs6906366 | 1.771 | rs4897166 | 0.027 | -3.660 | -0.200 | 61 | 7 | enet | 0.020 | 3.60E-03 | 2.38E-01 | 8.12E-01 |
| RHBDF1 | 16 | 108058 | 126354 | 0.0494 | rs216605 | -0.896 | rs2541613 | 0.002 | -3.780 | 0.123 | 57 | 14 | enet | 0.019 | 6.10E-03 | 2.36E-01 | 8.13E-01 |
| NFAT5 | 16 | 69598997 | 69738569 | 0.1780 | rs8057189 | -1.995 | rs8055929 | -0.001 | 3.230 | 0.365 | 122 | 14 | enet | 0.008 | 5.30E-02 | -2.37E-01 | 8.13E-01 |
| WDR17 | 4 | 176986985 | 177103978 | 0.0843 | rs4690681 | 2.870 | rs4475089 | 0.055 | 5.730 | 0.502 | 150 | 8 | enet | 0.066 | 2.50E-07 | 2.36E-01 | 8.14E-01 |
| KBTBD3 | 11 | 105921825 | 105948492 | 0.0797 | rs1940761 | 1.280 | rs7942450 | 0.008 | -3.400 | 0.087 | 80 | 22 | enet | 0.037 | 1.10E-04 | 2.35E-01 | 8.14E-01 |
| CRK | 17 | 1323983 | 1366456 | 0.0337 | rs1532976 | 2.840 | rs7208768 | 0.003 | 3.530 | 0.539 | 93 | 10 | enet | 0.005 | 1.00E-01 | -2.34E-01 | 8.15E-01 |
| LAMC1 | 1 | 182992595 | 183114727 | 0.1083 | rs6424877 | 1.632 | rs3118182 | 0.010 | -3.550 | 0.387 | 172 | 27 | enet | 0.017 | 6.60E-03 | -2.33E-01 | 8.16E-01 |
| ARSB | 5 | 78073032 | 78281910 | 0.4586 | rs17823744 | -2.222 | rs12522878 | 0.280 | -10.640 | -0.150 | 228 | 45 | enet | 0.460 | 6.40E-53 | 2.33E-01 | 8.16E-01 |
| TRMT11 | 6 | 126307576 | 126360422 | 0.0879 | rs6906366 | 1.771 | rs9388465 | 0.013 | -3.410 | -0.267 | 69 | 18 | enet | 0.015 | 1.30E-02 | -2.33E-01 | 8.16E-01 |
| SLC16A7 | 12 | 59989848 | 60176395 | 0.2487 | rs17573452 | -1.480 | rs10506399 | 0.260 | -10.060 | 0.112 | 156 | 23 | enet | 0.240 | 1.80E-24 | 2.33E-01 | 8.16E-01 |
| ETFA | 15 | 76507696 | 76603813 | 0.0776 | rs8042654 | 1.800 | rs2456046 | 0.104 | -6.590 | 0.702 | 116 | 15 | enet | 0.100 | 1.10E-10 | -2.31E-01 | 8.17E-01 |
| TP53I3 | 2 | 24300303 | 24308731 | 0.2989 | rs12713161 | 2.929 | rs1134516 | 0.273 | -10.210 | 0.659 | 77 | 19 | enet | 0.280 | 6.70E-28 | -2.27E-01 | 8.20E-01 |
| HEBP2 | 6 | 138724668 | 138743334 | 0.0539 | rs9495051 | -1.504 | rs9495070 | 0.024 | -3.730 | -0.326 | 91 | 14 | enet | 0.039 | 6.30E-05 | 2.26E-01 | 8.21E-01 |
| PCMT1 | 6 | 150070579 | 150132556 | 0.1725 | rs9383583 | 0.631 | rs1112730 | 0.266 | -10.410 | -0.356 | 87 | 23 | enet | 0.270 | 4.70E-28 | 2.26E-01 | 8.21E-01 |
| GAS2L1 | 22 | 29702572 | 29708774 | 0.0402 | rs2518683 | 2.450 | rs13056977 | 0.013 | 3.650 | -0.182 | 89 | 8 | enet | 0.016 | 8.70E-03 | -2.26E-01 | 8.21E-01 |
| GNA12 | 7 | 2767746 | 2883958 | 0.0815 | rs17132719 | 2.860 | rs208358 | 0.080 | -5.790 | -0.764 | 165 | 5 | enet | 0.072 | 7.90E-08 | 2.26E-01 | 8.21E-01 |
| C11orf54 | 11 | 93474757 | 93497915 | 0.3882 | rs12785450 | -1.890 | rs652961 | 0.581 | 14.790 | 0.205 | 65 | 11 | enet | 0.560 | 5.90E-70 | 2.24E-01 | 8.23E-01 |
| ARAP2 | 4 | 35949843 | 36246131 | 0.0634 | rs7664826 | 2.290 | rs2889174 | 0.005 | -3.870 | 0.854 | 273 | 15 | enet | 0.015 | 9.10E-03 | 2.21E-01 | 8.25E-01 |
| ALKBH7 | 19 | 6372444 | 6375042 | 0.1335 | rs348362 | 1.660 | rs7540 | 0.176 | -6.460 | 0.372 | 78 | 8 | enet | 0.120 | 8.80E-08 | -2.20E-01 | 8.26E-01 |
| TMEM33 | 4 | 41937137 | 41962589 | 0.0412 | rs1532937 | -2.310 | rs2660343 | 0.028 | -4.370 | -0.244 | 77 | 9 | enet | 0.020 | 3.50E-03 | 2.18E-01 | 8.28E-01 |
| PPIH | 1 | 43124096 | 43142429 | 0.0554 | rs746387 | 2.448 | rs12145783 | 0.054 | -4.660 | 0.518 | 76 | 16 | enet | 0.040 | 5.00E-05 | 2.16E-01 | 8.29E-01 |
| ME1 | 6 | 83920108 | 84140797 | 0.7240 | rs9449589 | -1.558 | rs6901074 | 0.083 | 6.160 | -0.108 | 134 | 48 | enet | 0.340 | 1.70E-36 | 2.16E-01 | 8.29E-01 |
| ACAA2 | 18 | 47309869 | 47340330 | 0.0980 | rs596778 | -2.120 | rs17714688 | 0.005 | 4.470 | -0.568 | 133 | 25 | enet | 0.019 | 4.60E-03 | 2.16E-01 | 8.29E-01 |
| CACNA2D1 | 7 | 81575760 | 82073114 | 0.0722 | rs38540 | 2.640 | rs6947580 | 0.063 | 5.250 | 0.182 | 367 | 19 | enet | 0.042 | 3.90E-05 | -2.15E-01 | 8.30E-01 |
| CAND2 | 3 | 12837971 | 12913415 | 0.5808 | rs1529755 | -2.300 | rs4642101 | 0.462 | -13.080 | -0.554 | 112 | 41 | enet | 0.510 | 9.00E-60 | 2.14E-01 | 8.31E-01 |
| SLC6A17 | 1 | 110693108 | 110744824 | 0.1723 | rs4112315 | 3.810 | rs7554444 | 0.196 | -9.090 | 0.333 | 133 | 21 | enet | 0.220 | 5.50E-22 | -2.14E-01 | 8.31E-01 |
| TMEM229A | 7 | 123670973 | 123673523 | 0.1280 | rs12706565 | -2.820 | rs10231282 | 0.075 | 4.950 | 0.393 | 64 | 7 | enet | 0.067 | 6.30E-05 | -2.13E-01 | 8.31E-01 |
| PDF | 16 | 69362524 | 69364498 | 0.0917 | rs8061222 | 2.803 | rs877534 | 0.137 | 7.480 | 0.087 | 65 | 4 | enet | 0.120 | 2.00E-12 | 2.09E-01 | 8.34E-01 |
| CARS2 | 13 | 111293759 | 111365950 | 0.4289 | rs12184676 | 2.340 | rs7337089 | 0.209 | -10.230 | 0.119 | 144 | 21 | enet | 0.370 | 3.20E-39 | 2.09E-01 | 8.34E-01 |
| AKR7A2 | 1 | 19630459 | 19638640 | 0.1225 | rs12130051 | 1.608 | rs6694099 | 0.077 | -5.960 | -0.025 | 125 | 19 | enet | 0.068 | 2.00E-07 | 2.09E-01 | 8.35E-01 |
| EOGT | 3 | 69024365 | 69063112 | 0.1082 | rs13072064 | -1.920 | rs2289245 | 0.080 | -5.350 | -0.169 | 91 | 11 | enet | 0.099 | 3.50E-08 | 2.08E-01 | 8.35E-01 |
| RAB3C | 5 | 57878048 | 58155213 | 0.1141 | rs158957 | -2.650 | rs292969 | 0.088 | 6.050 | 0.287 | 236 | 15 | enet | 0.047 | 1.40E-05 | 2.07E-01 | 8.36E-01 |
| GDPD1 | 17 | 57297828 | 57353328 | 0.0480 | rs534242 | 3.310 | rs11079369 | 0.067 | 5.540 | -0.256 | 80 | 8 | enet | 0.056 | 2.10E-06 | -2.07E-01 | 8.36E-01 |
| TBC1D13 | 9 | 131549483 | 131572711 | 0.0869 | rs10739734 | 2.000 | rs2900269 | 0.101 | 6.640 | -0.156 | 52 | 12 | enet | 0.077 | 2.60E-08 | -2.07E-01 | 8.36E-01 |
| TRPV2 | 17 | 16318856 | 16340317 | 0.6273 | rs12453841 | -1.910 | rs3813769 | 0.628 | -15.380 | -0.341 | 83 | 16 | enet | 0.630 | 4.90E-83 | 2.05E-01 | 8.37E-01 |
| NUDT5 | 10 | 12207324 | 12238143 | 0.0613 | rs11257688 | -2.030 | rs11257576 | 0.027 | 4.060 | 0.368 | 92 | 6 | enet | 0.012 | 1.90E-02 | 2.04E-01 | 8.39E-01 |
| POMGNT2 | 3 | 43120724 | 43147568 | 0.1051 | rs17075299 | -2.000 | rs7648947 | 0.099 | -6.470 | 0.339 | 96 | 9 | enet | 0.100 | 3.00E-10 | -2.03E-01 | 8.39E-01 |
| KCTD10 | 12 | 109886461 | 109915349 | 0.0485 | rs16940177 | -1.970 | rs6663 | 0.013 | 3.980 | -0.731 | 120 | 4 | enet | 0.032 | 2.90E-04 | -2.03E-01 | 8.39E-01 |
| LMO7 | 13 | 76194570 | 76434004 | 0.0471 | rs9670668 | -1.870 | rs530855 | 0.037 | 4.490 | -0.189 | 233 | 2 | enet | 0.017 | 6.20E-03 | -2.03E-01 | 8.39E-01 |
| LYPLA2 | 1 | 24117460 | 24122029 | 0.0610 | rs12733278 | -3.824 | rs7514394 | 0.009 | 3.750 | 2.202 | 60 | 17 | enet | 0.030 | 4.70E-04 | 2.01E-01 | 8.41E-01 |
| EFCAB7 | 1 | 63989043 | 64038364 | 0.0706 | rs10889433 | 2.782 | rs3004318 | 0.007 | 3.450 | -0.604 | 93 | 14 | enet | 0.004 | 1.30E-01 | 1.99E-01 | 8.43E-01 |
| TMEM173 | 5 | 138855119 | 138862520 | 0.1652 | rs10214401 | 2.130 | rs7380062 | 0.143 | -5.670 | 0.137 | 24 | 4 | enet | 0.110 | 9.00E-07 | -1.98E-01 | 8.43E-01 |
| FLNB | 3 | 57994127 | 58157982 | 0.0559 | rs4681655 | -3.040 | rs9880603 | 0.017 | -3.730 | -0.470 | 174 | 36 | enet | 0.023 | 1.70E-03 | 1.97E-01 | 8.44E-01 |

| | | | | | | | | | | | | | | | | | |
|---|---|---|---|---|---|---|---|---|---|---|---|---|---|---|---|---|---|
| RMDN3 | 15 | 41028082 | 41048049 | 0.2480 | rs1866170 | 2.040 | rs1142468 | 0.215 | -9.460 | -0.981 | 53 | 12 | enet | 0.260 | 6.20E-27 | 1.96E-01 | 8.45E-01 |
| SNPH | 20 | 1246960 | 1289972 | 0.1049 | rs8117744 | -3.218 | rs11905792 | 0.046 | -5.390 | 0.282 | 144 | 17 | enet | 0.075 | 4.00E-08 | -1.95E-01 | 8.46E-01 |
| EEFSEC | 3 | 127872297 | 128127485 | 0.0621 | rs2713604 | 3.220 | rs7641133 | 0.129 | -7.030 | -0.097 | 166 | 12 | enet | 0.100 | 1.30E-10 | 1.93E-01 | 8.47E-01 |
| THTPA | 14 | 24025216 | 24029480 | 0.0446 | rs222714 | -1.670 | rs34015250 | 0.037 | -4.760 | -0.226 | 91 | 6 | enet | 0.027 | 8.90E-04 | -1.93E-01 | 8.47E-01 |
| CYP4X1 | 1 | 47427036 | 47516423 | 0.0300 | rs9332998 | 1.649 | rs9793716 | 0.029 | -4.820 | -0.301 | 75 | 10 | enet | 0.036 | 1.30E-04 | 1.92E-01 | 8.48E-01 |
| ECI2 | 6 | 4115923 | 4135831 | 0.3286 | rs723796 | 2.362 | rs3177253 | 0.379 | -12.070 | 0.371 | 131 | 25 | enet | 0.470 | 1.70E-53 | -1.92E-01 | 8.48E-01 |
| COLGALT2 | 1 | 183898796 | 184006863 | 0.1131 | rs2986546 | 2.556 | rs1327136 | 0.059 | -5.030 | 0.568 | 183 | 7 | enet | 0.049 | 5.60E-05 | -1.89E-01 | 8.50E-01 |
| MBLAC2 | 5 | 89754020 | 89770585 | 0.0494 | rs3096032 | 2.528 | rs10473934 | 0.030 | 4.880 | -0.546 | 74 | 2 | enet | 0.030 | 4.60E-04 | -1.90E-01 | 8.50E-01 |
| CMPK1 | 1 | 47799469 | 47844511 | 0.6720 | rs3125630 | 2.757 | rs7555040 | 0.139 | -7.450 | -0.425 | 85 | 47 | enet | 0.130 | 5.00E-13 | -1.87E-01 | 8.51E-01 |
| EHD1 | 11 | 64619114 | 64655768 | 0.0604 | rs11231903 | -2.460 | rs4930685 | -0.002 | 3.190 | -0.278 | 79 | 25 | enet | 0.021 | 2.60E-03 | 1.88E-01 | 8.51E-01 |
| RELL1 | 4 | 37592422 | 37687998 | 0.1093 | rs17422624 | 2.500 | rs12710806 | 0.113 | -6.220 | 0.176 | 196 | 17 | enet | 0.093 | 1.10E-08 | 1.86E-01 | 8.52E-01 |
| UBQLN4 | 1 | 156005092 | 156023585 | 0.0396 | rs536857 | 2.051 | rs1111102 | 0.024 | -4.100 | -0.645 | 73 | 5 | enet | 0.013 | 1.40E-02 | 1.86E-01 | 8.53E-01 |
| GNAQ | 9 | 80331003 | 80646374 | 0.0368 | rs1328531 | 1.750 | rs7044515 | 0.033 | -5.100 | -0.178 | 165 | 9 | enet | 0.041 | 4.70E-05 | 1.85E-01 | 8.53E-01 |
| SLC25A19 | 17 | 73269073 | 73285591 | 0.1250 | rs9900586 | 4.860 | rs12603538 | 0.035 | -4.580 | 2.016 | 50 | 20 | enet | 0.060 | 1.50E-06 | -1.83E-01 | 8.55E-01 |
| FAM210B | 20 | 54933971 | 54943719 | 0.1238 | rs6024881 | 1.795 | rs6024786 | 0.087 | -6.580 | 0.589 | 110 | 15 | enet | 0.110 | 1.50E-11 | -1.81E-01 | 8.57E-01 |
| ECHS1 | 10 | 135175984 | 135187193 | 0.1661 | rs2275723 | -1.290 | rs11101707 | 0.087 | 7.040 | 0.143 | 73 | 7 | enet | 0.120 | 3.20E-12 | 1.79E-01 | 8.58E-01 |
| C19orf53 | 19 | 13884982 | 13889276 | 0.0522 | rs8106403 | 1.340 | rs1044733 | 0.023 | -3.830 | -0.024 | 56 | 15 | enet | 0.026 | 1.20E-03 | 1.79E-01 | 8.58E-01 |
| UROD | 1 | 45477819 | 45481247 | 0.0194 | rs452989 | 2.355 | rs998418 | 0.023 | -3.760 | -0.104 | 52 | 7 | enet | 0.026 | 9.70E-04 | -1.79E-01 | 8.58E-01 |
| PGAM2 | 7 | 44102326 | 44105186 | 0.1823 | rs4640970 | 1.400 | rs17546439 | 0.024 | -5.240 | -0.020 | 62 | 10 | enet | 0.071 | 1.10E-07 | -1.78E-01 | 8.59E-01 |
| SBF1 | 22 | 50883429 | 50913454 | 0.1190 | rs140522 | 6.310 | rs5771040 | 0.064 | -5.050 | 1.567 | 99 | 12 | enet | 0.046 | 1.50E-05 | 1.77E-01 | 8.60E-01 |
| TBC1D15 | 12 | 72233487 | 72320629 | 0.0543 | rs17110566 | -3.000 | rs6582065 | -0.003 | 3.100 | 1.345 | 138 | 22 | enet | 0.001 | 2.70E-01 | -1.76E-01 | 8.60E-01 |
| PBLD | 10 | 70042417 | 70092806 | 0.2181 | rs10998066 | 2.550 | rs10762201 | 0.331 | 11.270 | -0.129 | 125 | 15 | enet | 0.330 | 1.70E-34 | 1.76E-01 | 8.60E-01 |
| PGAM5 | 12 | 133287405 | 133299228 | 0.0776 | rs5745066 | 1.900 | rs11147015 | 0.038 | -3.950 | -0.177 | 88 | 13 | enet | 0.041 | 4.70E-05 | -1.75E-01 | 8.61E-01 |
| MYCBP | 1 | 39328636 | 39347289 | 0.0900 | rs1886690 | 2.138 | rs4970563 | 0.100 | 5.840 | 0.207 | 89 | 4 | enet | 0.076 | 4.90E-07 | 1.72E-01 | 8.63E-01 |
| WIPF1 | 2 | 175424300 | 175547644 | 0.0785 | rs12151707 | -2.179 | rs1010027 | 0.007 | -3.730 | 0.939 | 128 | 18 | enet | 0.012 | 3.00E-02 | -1.73E-01 | 8.63E-01 |
| KCTD21 | 11 | 77882295 | 77899868 | 0.0649 | rs11237385 | 3.070 | rs550458 | 0.051 | -4.370 | 0.402 | 99 | 13 | enet | 0.051 | 3.80E-05 | -1.71E-01 | 8.64E-01 |
| LY6H | 8 | 144239331 | 144242128 | 0.1249 | rs7824044 | -2.720 | rs7835528 | 0.083 | -6.790 | -0.627 | 78 | 10 | enet | 0.090 | 1.60E-09 | 1.70E-01 | 8.65E-01 |
| HACL1 | 3 | 15602211 | 15643338 | 0.4861 | rs2124493 | -2.570 | rs924814 | 0.047 | -5.410 | -1.235 | 88 | 30 | enet | 0.220 | 1.40E-21 | 1.70E-01 | 8.65E-01 |
| CYP4F11 | 19 | 16023177 | 16045677 | 0.1976 | rs757546 | -1.150 | rs1060463 | 0.067 | -6.040 | -0.341 | 113 | 21 | enet | 0.120 | 7.90E-11 | 1.69E-01 | 8.66E-01 |
| STON2 | 14 | 81727000 | 81902809 | 0.0682 | rs17111840 | 1.690 | rs6574636 | 0.063 | 5.110 | -0.578 | 131 | 11 | enet | 0.048 | 1.10E-05 | -1.69E-01 | 8.66E-01 |
| PTGR1 | 9 | 114312002 | 114362135 | 0.4510 | rs7854485 | 2.890 | rs3739704 | 0.328 | 11.160 | -0.798 | 115 | 49 | enet | 0.390 | 1.90E-42 | -1.68E-01 | 8.66E-01 |
| ACBD5 | 10 | 27484146 | 27531059 | 0.2174 | rs651134 | -1.870 | rs7918793 | 0.088 | -7.030 | 1.277 | 98 | 67 | enet | 0.045 | 2.00E-05 | -1.68E-01 | 8.67E-01 |
| TSPAN2 | 1 | 115590632 | 115632121 | 0.1131 | rs4839413 | -1.988 | rs17479252 | 0.082 | -4.640 | 0.966 | 139 | 4 | enet | 0.058 | 2.80E-04 | -1.68E-01 | 8.67E-01 |
| PGM2L1 | 11 | 74041363 | 74109518 | 0.1865 | rs601419 | 2.460 | rs11236101 | 0.014 | -4.290 | -1.018 | 132 | 23 | enet | 0.074 | 4.70E-08 | -1.68E-01 | 8.67E-01 |
| CAMKK2 | 12 | 121675497 | 121736111 | 0.1759 | rs7314454 | 2.480 | rs3794207 | 0.049 | 5.480 | -0.416 | 125 | 27 | enet | 0.120 | 2.40E-12 | 1.68E-01 | 8.67E-01 |
| L3HYPDH | 14 | 59927081 | 59951148 | 0.2744 | rs1253149 | -2.210 | rs2145042 | 0.129 | -8.330 | 0.245 | 111 | 20 | enet | 0.260 | 6.80E-26 | 1.67E-01 | 8.68E-01 |
| GPX4 | 19 | 1103936 | 1106787 | 0.2960 | rs4147932 | 3.200 | rs4807542 | 0.200 | 8.730 | 0.388 | 82 | 33 | enet | 0.250 | 5.10E-25 | 1.66E-01 | 8.68E-01 |
| HEXB | 5 | 73935848 | 74018472 | 0.0836 | rs17559939 | -2.296 | rs820880 | 0.059 | 5.360 | -0.356 | 140 | 12 | enet | 0.049 | 8.70E-06 | 1.65E-01 | 8.69E-01 |
| ARHGEF10 | 8 | 1772142 | 1906807 | 0.0979 | rs2294041 | -2.390 | rs3779698 | 0.009 | -5.000 | 0.438 | 264 | 14 | enet | 0.022 | 3.30E-03 | -1.64E-01 | 8.69E-01 |
| MRVI1 | 11 | 10594638 | 10715535 | 0.1904 | rs2242359 | -1.910 | rs11042916 | 0.116 | -7.540 | 0.259 | 211 | 23 | enet | 0.160 | 1.30E-16 | 1.65E-01 | 8.69E-01 |
| TANGO2 | 22 | 20004537 | 20053449 | 0.1936 | rs756661 | 2.570 | rs2008591 | 0.120 | 7.270 | -0.182 | 180 | 27 | enet | 0.110 | 1.30E-11 | -1.65E-01 | 8.69E-01 |
| MARC2 | 1 | 220921567 | 220958150 | 0.0485 | rs11118607 | -1.865 | rs6697965 | 0.067 | -5.520 | 0.175 | 132 | 9 | enet | 0.058 | 1.30E-06 | -1.63E-01 | 8.70E-01 |
| LMOD1 | 1 | 201865580 | 201915715 | 0.0490 | rs7529387 | 2.405 | rs2172935 | 0.016 | -3.900 | -0.266 | 105 | 11 | enet | 0.010 | 4.50E-02 | -1.64E-01 | 8.70E-01 |
| MDP1 | 14 | 24683143 | 24685276 | 0.3544 | rs2748531 | 2.560 | rs2295319 | 0.072 | 5.340 | -0.391 | 97 | 20 | enet | 0.130 | 7.40E-13 | 1.64E-01 | 8.70E-01 |
| NTPCR | 1 | 233086351 | 233119628 | 0.7271 | rs11586537 | -2.067 | rs12410594 | 0.676 | -15.950 | 0.076 | 128 | 22 | enet | 0.680 | 7.30E-94 | -1.62E-01 | 8.71E-01 |
| COQ3 | 6 | 99817276 | 99842080 | 0.2090 | rs1135676 | -1.548 | rs1009031 | 0.201 | -9.410 | 0.753 | 108 | 27 | enet | 0.260 | 7.90E-27 | 1.62E-01 | 8.71E-01 |

| | | | | | | | | | | | | | | | | | |
|---|---|---|---|---|---|---|---|---|---|---|---|---|---|---|---|---|---|
| PISD | 22 | 32014477 | 32058418 | 0.7361 | rs11703079 | 3.470 | rs2295251 | 0.084 | 6.370 | -1.429 | 89 | 30 | enet | 0.320 | 4.60E-33 | -1.57E-01 | 8.76E-01 |
| ABCG2 | 4 | 89011416 | 89152474 | 0.1433 | rs4693944 | 2.660 | rs2231142 | 0.150 | -7.440 | 0.167 | 162 | 6 | enet | 0.120 | 6.80E-11 | -1.54E-01 | 8.78E-01 |
| FBXO22 | 15 | 76196200 | 76227609 | 0.0414 | rs28695645 | -2.370 | rs2593275 | 0.043 | -4.330 | 0.634 | 56 | 20 | enet | 0.036 | 1.20E-04 | 1.52E-01 | 8.79E-01 |
| WDSUB1 | 2 | 160092304 | 160143310 | 0.4069 | rs10489999 | 3.035 | rs7564250 | 0.508 | -13.770 | 0.487 | 132 | 35 | enet | 0.570 | 1.80E-69 | -1.51E-01 | 8.80E-01 |
| ACE | 17 | 61554422 | 61599205 | 0.0975 | rs4968777 | 2.070 | rs4459609 | 0.059 | 5.970 | 0.337 | 71 | 5 | enet | 0.069 | 1.40E-07 | -1.51E-01 | 8.80E-01 |
| ILVBL | 19 | 15225795 | 15236596 | 0.0816 | rs10414659 | 1.880 | rs718100 | 0.013 | -4.200 | 0.142 | 103 | 10 | enet | 0.025 | 1.30E-03 | 1.50E-01 | 8.80E-01 |
| HARS | 5 | 140052758 | 140071609 | 0.1084 | rs34282994 | -2.786 | rs2262572 | -0.002 | 3.380 | -0.064 | 76 | 3 | enet | -0.001 | 4.00E-01 | -1.50E-01 | 8.81E-01 |
| AP3D1 | 19 | 2100988 | 2164464 | 0.0564 | rs4405674 | 2.840 | rs2072304 | 0.035 | -4.290 | -0.086 | 72 | 6 | enet | 0.015 | 9.50E-03 | 1.47E-01 | 8.83E-01 |
| ANKRD29 | 18 | 21178890 | 21242849 | 0.0535 | rs12605274 | 1.530 | rs11662113 | 0.025 | -4.130 | -0.613 | 94 | 5 | enet | 0.019 | 4.60E-03 | 1.47E-01 | 8.84E-01 |
| CLCC1 | 1 | 109472130 | 109506111 | 0.0390 | rs12028832 | -2.280 | rs1769740 | 0.023 | 3.980 | -0.522 | 107 | 11 | enet | 0.009 | 3.60E-02 | -1.46E-01 | 8.84E-01 |
| COA1 | 7 | 43648055 | 43769316 | 0.0842 | rs849165 | 2.010 | rs2286297 | 0.027 | -4.520 | 0.434 | 127 | 20 | enet | 0.058 | 1.50E-06 | -1.43E-01 | 8.86E-01 |
| APMAP | 20 | 24943561 | 24973615 | 0.0378 | rs3746332 | 2.366 | rs6114995 | 0.015 | -4.330 | -0.125 | 120 | 8 | enet | 0.004 | 1.10E-01 | 1.43E-01 | 8.87E-01 |
| TPP1 | 11 | 6634000 | 6640692 | 0.0684 | rs4758124 | 2.400 | rs1800752 | 0.031 | 4.490 | 0.248 | 147 | 21 | enet | 0.028 | 6.20E-04 | -1.41E-01 | 8.88E-01 |
| NAP1L4 | 11 | 2965667 | 3013607 | 0.1061 | rs2411767 | -2.780 | rs16928841 | 0.014 | 3.870 | 0.265 | 126 | 37 | enet | 0.062 | 5.80E-07 | 1.40E-01 | 8.88E-01 |
| DNAJC6 | 1 | 65713902 | 65881552 | 0.0583 | rs9436301 | 2.227 | rs1336474 | 0.004 | 3.680 | -0.733 | 166 | 14 | enet | 0.001 | 2.50E-01 | -1.40E-01 | 8.89E-01 |
| FASTKD5 | 20 | 3127165 | 3140543 | 0.3218 | rs2422863 | -2.076 | rs16988305 | 0.246 | -9.640 | -0.657 | 84 | 17 | enet | 0.230 | 4.20E-22 | -1.38E-01 | 8.90E-01 |
| AAMDC | 11 | 77532155 | 77629478 | 0.4115 | rs650636 | -1.640 | rs3819211 | 0.592 | -14.950 | -0.007 | 102 | 38 | enet | 0.600 | 6.10E-76 | 1.37E-01 | 8.91E-01 |
| RETSAT | 2 | 85569211 | 85581743 | 0.1216 | rs6547602 | 2.664 | rs11678606 | 0.076 | -6.530 | -0.435 | 116 | 23 | enet | 0.085 | 4.60E-09 | 1.36E-01 | 8.92E-01 |
| DNAJC10 | 2 | 183580999 | 183659191 | 0.0824 | rs7592998 | 2.538 | rs288299 | 0.050 | -4.740 | 0.438 | 113 | 25 | enet | 0.040 | 5.80E-05 | 1.36E-01 | 8.92E-01 |
| PAM16 | 16 | 4381550 | 4405608 | 0.1853 | rs2283479 | -3.080 | rs11989 | 0.008 | 3.500 | -0.052 | 71 | 14 | enet | 0.059 | 9.90E-07 | -1.36E-01 | 8.92E-01 |
| STXBP5L | 3 | 120626919 | 121143608 | 0.0896 | rs12491742 | -3.320 | rs1850293 | 0.007 | 3.550 | 0.107 | 205 | 16 | enet | 0.019 | 4.50E-03 | -1.34E-01 | 8.93E-01 |
| PTGFRN | 1 | 117452679 | 117532980 | 0.0442 | rs11583252 | -2.598 | rs4233450 | -0.002 | -3.410 | 0.450 | 148 | 23 | enet | -0.002 | 5.70E-01 | -1.34E-01 | 8.94E-01 |
| GAA | 17 | 78075355 | 78093678 | 0.2080 | rs4889998 | -3.020 | rs12952612 | 0.170 | 8.130 | 0.032 | 98 | 20 | enet | 0.220 | 1.70E-22 | -1.32E-01 | 8.95E-01 |
| SHPK | 17 | 3511556 | 3539616 | 0.4645 | rs3744678 | 1.830 | rs17707155 | 0.313 | 10.890 | 0.235 | 149 | 28 | enet | 0.450 | 1.50E-50 | 1.32E-01 | 8.95E-01 |
| NMNAT3 | 3 | 139279022 | 139396859 | 0.3752 | rs9838798 | 2.390 | rs16849220 | 0.088 | 7.460 | 0.228 | 163 | 33 | enet | 0.350 | 1.30E-37 | -1.31E-01 | 8.95E-01 |
| CPNE1 | 20 | 34213953 | 34252878 | 0.4209 | rs6060540 | 0.834 | rs11696527 | 0.393 | -12.400 | 0.087 | 84 | 22 | enet | 0.400 | 1.20E-43 | -1.31E-01 | 8.96E-01 |
| NUDCD3 | 7 | 44418720 | 44530479 | 0.0926 | rs1055971 | 1.860 | rs1043084 | 0.130 | 7.090 | -0.402 | 106 | 12 | enet | 0.120 | 6.20E-12 | -1.31E-01 | 8.96E-01 |
| CA14 | 1 | 150229554 | 150237478 | 0.2657 | rs12403795 | -1.978 | rs17646887 | 0.059 | -4.870 | -1.608 | 59 | 16 | enet | 0.025 | 1.10E-03 | 1.28E-01 | 8.98E-01 |
| SCAMP5 | 15 | 75249560 | 75313837 | 0.0374 | rs6495126 | 3.230 | rs2304903 | 0.021 | 4.230 | -1.097 | 80 | 12 | enet | 0.029 | 5.50E-04 | -1.28E-01 | 8.99E-01 |
| ALPL | 1 | 21835858 | 21904905 | 0.0660 | rs1780316 | 2.660 | rs1780329 | 0.063 | -5.220 | 0.059 | 146 | 4 | enet | 0.048 | 1.10E-05 | 1.27E-01 | 8.99E-01 |
| GFER | 16 | 2034208 | 2037750 | 0.0609 | rs12929367 | -2.203 | rs1058474 | 0.055 | 5.170 | -0.117 | 86 | 9 | enet | 0.033 | 2.90E-04 | -1.27E-01 | 8.99E-01 |
| PRDX1 | 1 | 45976708 | 45988719 | 0.3045 | rs534534 | 1.601 | rs6657284 | 0.030 | 3.900 | -1.208 | 78 | 11 | enet | 0.060 | 8.50E-07 | 1.26E-01 | 9.00E-01 |
| SRA1 | 5 | 139916925 | 139937895 | 0.0688 | rs753280 | -2.108 | rs6871703 | 0.032 | -4.510 | -0.489 | 58 | 13 | enet | 0.032 | 3.00E-04 | -1.25E-01 | 9.01E-01 |
| ACTR1B | 2 | 98272431 | 98280570 | 0.5135 | rs895437 | 2.181 | rs11692435 | 0.259 | -10.010 | -0.254 | 42 | 15 | enet | 0.290 | 5.90E-30 | -1.23E-01 | 9.02E-01 |
| CBR4 | 4 | 169784921 | 169931426 | 0.1193 | rs10518032 | 2.280 | rs1548315 | 0.073 | -6.720 | -0.628 | 165 | 20 | enet | 0.110 | 2.50E-11 | 1.23E-01 | 9.02E-01 |
| COPS7B | 2 | 232646381 | 232673963 | 0.0283 | rs17199677 | 1.373 | rs736326 | 0.013 | 3.980 | -0.266 | 64 | 6 | enet | 0.012 | 1.80E-02 | -1.22E-01 | 9.03E-01 |
| NQO1 | 16 | 69740899 | 69760854 | 0.5361 | rs17231474 | -1.210 | rs12232410 | 0.496 | -13.710 | 0.282 | 74 | 21 | enet | 0.570 | 6.90E-71 | 1.21E-01 | 9.03E-01 |
| TTLL12 | 22 | 43562628 | 43583139 | 0.3972 | rs998409 | 2.410 | rs6973 | 0.150 | -8.650 | 0.163 | 164 | 34 | enet | 0.350 | 3.20E-37 | 1.21E-01 | 9.04E-01 |
| ISOC2 | 19 | 55964352 | 55973710 | 0.0722 | rs542186 | 1.860 | rs751152 | 0.059 | -4.740 | -0.321 | 67 | 11 | enet | 0.086 | 2.10E-08 | -1.20E-01 | 9.05E-01 |
| SCO2 | 22 | 50961997 | 50964868 | 0.3083 | rs140522 | 6.310 | rs5770871 | 0.127 | -7.670 | 2.648 | 109 | 32 | enet | 0.230 | 2.10E-23 | -1.19E-01 | 9.05E-01 |
| MTHFS | 15 | 80125927 | 80189721 | 0.0771 | rs2733102 | -2.000 | rs16971429 | 0.017 | -4.210 | 0.381 | 144 | 10 | enet | -0.005 | 8.30E-01 | 1.18E-01 | 9.06E-01 |
| PNPLA7 | 9 | 140354404 | 140444986 | 0.2550 | rs11137131 | 1.860 | rs2259657 | 0.084 | -5.920 | 0.400 | 59 | 5 | enet | 0.096 | 6.70E-10 | -1.18E-01 | 9.06E-01 |
| VPS13C | 15 | 62144588 | 62352672 | 0.1844 | rs10519159 | -2.540 | rs12442569 | 0.050 | -6.510 | -0.587 | 205 | 21 | enet | 0.140 | 2.10E-14 | 1.17E-01 | 9.07E-01 |
| SARDH | 9 | 136528682 | 136605077 | 0.1119 | rs12552884 | 2.770 | rs886016 | 0.092 | 6.080 | -0.743 | 181 | 13 | enet | 0.070 | 1.30E-07 | -1.16E-01 | 9.08E-01 |
| HTT | 4 | 3076408 | 3245676 | 0.0484 | rs3856973 | -2.770 | rs2857839 | -0.002 | 3.730 | -1.401 | 138 | 16 | enet | 0.008 | 5.00E-02 | 1.16E-01 | 9.08E-01 |
| GSTT2B | 22 | 24299601 | 24303373 | 0.5268 | rs17550765 | -1.660 | rs2739338 | 0.492 | 13.660 | -0.098 | 75 | 24 | enet | 0.630 | 2.50E-82 | 1.16E-01 | 9.08E-01 |

| | | | | | | | | | | | | | | | | | |
|---|---|---|---|---|---|---|---|---|---|---|---|---|---|---|---|---|---|
| KHK | 2 | 27309615 | 27323640 | 0.3860 | rs934986 | 2.545 | rs2304681 | 0.564 | -13.690 | -0.212 | 61 | 9 | enet | 0.540 | 7.60E-58 | 1.14E-01 | 9.09E-01 |
| AP2A2 | 11 | 924894 | 1012239 | 0.1432 | rs7942850 | -1.790 | rs11246340 | -0.001 | 4.000 | -0.370 | 133 | 17 | enet | 0.043 | 2.90E-05 | 1.14E-01 | 9.09E-01 |
| AARS2 | 6 | 44267391 | 44281063 | 0.3442 | rs2396243 | 2.009 | rs2233434 | 0.190 | -8.650 | -0.116 | 107 | 22 | enet | 0.220 | 4.00E-22 | -1.13E-01 | 9.10E-01 |
| ACP6 | 1 | 147101453 | 147142618 | 0.4812 | rs1027424 | -2.808 | rs12119079 | 0.447 | -10.760 | 0.156 | 129 | 30 | enet | 0.480 | 1.40E-36 | 1.11E-01 | 9.12E-01 |
| GSS | 20 | 33516236 | 33543620 | 0.0612 | rs6088659 | -1.275 | rs2236271 | 0.059 | -5.540 | -0.093 | 82 | 6 | enet | 0.051 | 6.20E-06 | 1.09E-01 | 9.13E-01 |
| ADSL | 22 | 40742507 | 40786467 | 0.1277 | rs139063 | -2.450 | rs8135371 | 0.118 | 6.770 | 0.759 | 74 | 5 | enet | 0.110 | 2.60E-11 | 1.09E-01 | 9.14E-01 |
| NENF | 1 | 212606229 | 212619714 | 0.0886 | rs701907 | -1.878 | rs4804 | 0.106 | -6.560 | -0.135 | 104 | 5 | enet | 0.100 | 2.60E-10 | 1.08E-01 | 9.14E-01 |
| SLC5A6 | 2 | 27422455 | 27435826 | 0.1230 | rs11682713 | 2.713 | rs1395 | 0.051 | -4.890 | 1.433 | 79 | 14 | enet | 0.120 | 8.20E-10 | 1.08E-01 | 9.14E-01 |
| RPA3 | 7 | 7676149 | 7758238 | 0.0534 | rs17466949 | -2.340 | rs12702648 | 0.003 | 3.590 | 1.113 | 209 | 10 | enet | -0.002 | 6.60E-01 | -1.07E-01 | 9.15E-01 |
| TSPAN9 | 12 | 3186521 | 3395730 | 0.1558 | rs11062486 | 2.520 | rs7963488 | 0.009 | -4.380 | -0.050 | 282 | 23 | enet | 0.066 | 1.70E-06 | -1.06E-01 | 9.15E-01 |
| AMZ2 | 17 | 66243715 | 66253297 | 0.4742 | rs3760269 | -2.670 | rs3213690 | 0.453 | -12.870 | -0.052 | 83 | 13 | enet | 0.440 | 7.20E-48 | -1.06E-01 | 9.16E-01 |
| CTSH | 15 | 79213400 | 79241916 | 0.4259 | rs16970344 | -4.650 | rs12148472 | 0.333 | -11.220 | 0.011 | 97 | 25 | enet | 0.320 | 3.50E-33 | 1.03E-01 | 9.18E-01 |
| DNAL1 | 14 | 74111578 | 74170435 | 0.0666 | rs17129410 | 1.990 | rs10162371 | 0.037 | -4.160 | -0.154 | 98 | 8 | enet | 0.022 | 2.10E-03 | -1.03E-01 | 9.18E-01 |
| RTFDC1 | 20 | 55043647 | 55093943 | 0.1823 | rs6014763 | 2.036 | rs6024911 | 0.235 | -9.220 | -0.030 | 116 | 5 | enet | 0.230 | 8.10E-22 | -1.01E-01 | 9.19E-01 |
| MAP1LC3B | 16 | 87417601 | 87438385 | 0.0889 | rs9903 | 3.727 | rs4843593 | -0.002 | -3.830 | 0.392 | 95 | 5 | enet | -0.002 | 7.20E-01 | 1.01E-01 | 9.20E-01 |
| COMTD1 | 10 | 76993727 | 76995788 | 0.3747 | rs1259578 | 1.740 | rs7073279 | 0.146 | -7.580 | -0.757 | 65 | 21 | enet | 0.140 | 1.70E-13 | 9.92E-02 | 9.21E-01 |
| APOA1BP | 1 | 156561554 | 156564091 | 0.2428 | rs7544205 | 2.986 | rs12567958 | 0.327 | -11.210 | 0.218 | 102 | 31 | enet | 0.380 | 1.20E-40 | 9.90E-02 | 9.21E-01 |
| UBXN2B | 8 | 59323823 | 59364060 | 0.0692 | rs17260430 | 2.430 | rs13277646 | 0.055 | -5.240 | 0.025 | 120 | 9 | enet | 0.058 | 3.60E-06 | 9.91E-02 | 9.21E-01 |
| CDS2 | 20 | 5107432 | 5178533 | 0.0393 | rs16990786 | -1.928 | rs1040758 | 0.041 | -5.030 | 0.372 | 115 | 3 | enet | 0.041 | 4.30E-05 | -9.97E-02 | 9.21E-01 |
| SSTR2 | 17 | 71161151 | 71167185 | 0.1005 | rs17248554 | 2.060 | rs8081059 | 0.004 | 3.710 | 0.664 | 104 | 20 | enet | 0.040 | 5.10E-05 | 9.69E-02 | 9.23E-01 |
| MDH2 | 7 | 75677369 | 75696826 | 0.1029 | rs2302434 | -2.260 | rs4732511 | 0.028 | 4.250 | -0.359 | 79 | 8 | enet | 0.032 | 3.00E-04 | 9.49E-02 | 9.24E-01 |
| CPOX | 3 | 98239976 | 98312567 | 0.4873 | rs1461161 | 2.770 | rs1131857 | 0.346 | -11.510 | -0.210 | 113 | 23 | enet | 0.370 | 1.20E-39 | 9.44E-02 | 9.25E-01 |
| SLK | 10 | 105726959 | 105788991 | 0.0791 | rs2902638 | -2.880 | rs4917407 | 0.000 | -3.340 | -1.017 | 113 | 13 | enet | 0.008 | 5.10E-02 | -9.21E-02 | 9.27E-01 |
| IST1 | 16 | 71879899 | 71962913 | 0.2535 | rs11859903 | 3.034 | rs12445641 | 0.029 | -5.150 | -0.636 | 115 | 22 | enet | 0.110 | 2.40E-11 | -9.21E-02 | 9.27E-01 |
| MON1B | 16 | 77224732 | 77236302 | 0.0669 | rs1594050 | -2.106 | rs2081254 | 0.013 | -4.230 | 0.220 | 140 | 9 | enet | 0.007 | 6.30E-02 | -8.98E-02 | 9.28E-01 |
| FN3KRP | 17 | 80674559 | 80688204 | 0.6054 | rs12600791 | 1.500 | rs3859206 | 0.419 | 12.600 | 0.184 | 74 | 20 | enet | 0.500 | 1.60E-58 | 9.05E-02 | 9.28E-01 |
| ART3 | 4 | 76932337 | 77033955 | 0.1987 | rs6532111 | -2.740 | rs9995300 | 0.038 | 5.120 | 1.576 | 173 | 30 | enet | 0.072 | 1.50E-07 | 8.99E-02 | 9.28E-01 |
| LOH12CR1 | 12 | 12510013 | 12619840 | 0.1061 | rs4763800 | 1.970 | rs10505767 | 0.092 | -6.150 | -0.078 | 158 | 21 | enet | 0.084 | 5.40E-09 | 8.89E-02 | 9.29E-01 |
| GPT | 8 | 145728356 | 145732557 | 0.0441 | rs6989368 | 1.150 | rs13254911 | 0.069 | -5.390 | 0.084 | 52 | 2 | enet | 0.052 | 4.50E-06 | -8.67E-02 | 9.31E-01 |
| ANKLE2 | 12 | 133302254 | 133338474 | 0.0397 | rs5745066 | 1.900 | rs1132375 | 0.026 | -4.210 | -0.660 | 105 | 7 | enet | 0.009 | 3.90E-02 | 8.62E-02 | 9.31E-01 |
| KIAA1549 | 7 | 138516126 | 138666064 | 0.0906 | rs10271373 | 4.310 | rs7809049 | 0.029 | 5.170 | 0.058 | 181 | 16 | enet | 0.039 | 7.40E-05 | -8.59E-02 | 9.32E-01 |
| AKR1A1 | 1 | 46016215 | 46035721 | 0.8267 | rs534534 | 1.601 | rs2229540 | 0.486 | -13.550 | 0.075 | 80 | 11 | enet | 0.450 | 1.70E-50 | 8.43E-02 | 9.33E-01 |
| ACOT4 | 14 | 74058410 | 74063200 | 0.2645 | rs10138789 | 2.320 | rs11625677 | 0.179 | -6.870 | 0.285 | 69 | 17 | enet | 0.210 | 8.70E-14 | -8.45E-02 | 9.33E-01 |
| CNTNAP3 | 9 | 39072764 | 39288312 | 0.2400 | rs4961901 | -0.490 | rs4961901 | -0.002 | 1.590 | -0.490 | 2 | 2 | enet | 0.062 | 4.50E-05 | -8.38E-02 | 9.33E-01 |
| LSS | 21 | 47608055 | 47648738 | 0.3507 | rs17296821 | 2.990 | rs4819214 | 0.361 | -11.690 | 0.165 | 126 | 21 | enet | 0.400 | 2.80E-43 | 8.26E-02 | 9.34E-01 |
| GOLGA3 | 12 | 133345495 | 133405444 | 0.2053 | rs13273 | -2.190 | rs12303977 | 0.166 | 8.170 | -0.694 | 101 | 20 | enet | 0.150 | 7.40E-15 | -8.14E-02 | 9.35E-01 |
| HRSP12 | 8 | 99114572 | 99129469 | 0.1475 | rs2514344 | -2.920 | rs2514334 | 0.155 | -7.880 | -0.307 | 121 | 5 | enet | 0.160 | 4.10E-16 | -8.00E-02 | 9.36E-01 |
| NLRX1 | 11 | 119037277 | 119054725 | 0.1486 | rs549893 | 2.800 | rs4938637 | 0.042 | 5.190 | -0.284 | 87 | 18 | enet | 0.075 | 3.80E-08 | -8.00E-02 | 9.36E-01 |
| RABEP1 | 17 | 5185558 | 5289129 | 0.1068 | rs2585284 | -1.890 | rs11078559 | 0.128 | -7.560 | 0.126 | 117 | 9 | enet | 0.110 | 2.10E-11 | -8.08E-02 | 9.36E-01 |
| PPA1 | 10 | 71962586 | 71993667 | 0.0333 | rs11813308 | 2.580 | rs4746965 | 0.000 | 3.270 | 0.831 | 148 | 14 | enet | 0.015 | 1.10E-02 | 7.98E-02 | 9.36E-01 |
| NID2 | 14 | 52471521 | 52535712 | 0.0843 | rs4898725 | 3.090 | rs2516585 | 0.029 | -4.840 | 1.023 | 182 | 23 | enet | 0.045 | 2.00E-05 | 7.93E-02 | 9.37E-01 |
| DGUOK | 2 | 74153953 | 74186088 | 0.4973 | rs6724905 | 2.198 | rs7570375 | 0.084 | -5.960 | -0.345 | 126 | 35 | enet | 0.180 | 4.20E-18 | -7.77E-02 | 9.38E-01 |
| GUF1 | 4 | 44680444 | 44702943 | 0.1726 | rs4695023 | 1.690 | rs1547525 | 0.275 | -10.590 | -0.165 | 87 | 15 | enet | 0.330 | 2.30E-34 | 7.71E-02 | 9.39E-01 |
| SAA2 | 11 | 18260770 | 18270190 | 0.2701 | rs1122938 | 2.070 | rs7950019 | 0.011 | 4.440 | 0.043 | 113 | 53 | enet | 0.110 | 2.80E-09 | 7.51E-02 | 9.40E-01 |
| DCAKD | 17 | 43100708 | 43138473 | 0.0671 | rs4793165 | -2.870 | rs6503410 | 0.017 | 4.040 | -0.472 | 116 | 13 | enet | 0.035 | 1.40E-04 | 7.48E-02 | 9.40E-01 |
| KIAA0391 | 14 | 35591052 | 35743271 | 0.0632 | rs7147346 | -2.490 | rs11156878 | 0.041 | -3.770 | 0.685 | 130 | 9 | enet | 0.044 | 1.50E-04 | -7.45E-02 | 9.41E-01 |

| | | | | | | | | | | | | | | | | | |
|---|---|---|---|---|---|---|---|---|---|---|---|---|---|---|---|---|---|
| ROBO1 | 3 | 78646390 | 79816965 | 0.0866 | rs3851995 | 2.610 | rs7612183 | 0.041 | 5.330 | 0.304 | 386 | 11 | enet | 0.047 | 1.30E-05 | 7.40E-02 | 9.41E-01 |
| FARP1 | 13 | 98794816 | 99102027 | 0.1042 | rs1571524 | 2.830 | rs17568378 | 0.004 | -4.020 | 0.612 | 353 | 37 | enet | 0.019 | 5.00E-03 | 7.31E-02 | 9.42E-01 |
| DPH1 | 17 | 1933404 | 1946724 | 0.1368 | rs7213049 | 3.000 | rs11653030 | 0.109 | -5.620 | -0.149 | 95 | 6 | enet | 0.090 | 1.70E-07 | 7.31E-02 | 9.42E-01 |
| OMG | 17 | 29599031 | 29624557 | 0.2354 | rs2525570 | 1.180 | rs11080149 | 0.042 | -4.480 | -0.133 | 71 | 4 | enet | 0.030 | 4.10E-04 | 7.29E-02 | 9.42E-01 |
| PSPH | 7 | 56078744 | 56119297 | 0.0681 | rs2242508 | -5.130 | rs816229 | 0.067 | -5.220 | -0.624 | 83 | 4 | enet | 0.045 | 1.90E-05 | -7.24E-02 | 9.42E-01 |
| DUSP23 | 1 | 159750722 | 159752333 | 0.6683 | rs4131568 | -3.036 | rs1129923 | 0.556 | -14.550 | -0.017 | 104 | 8 | enet | 0.550 | 1.30E-66 | -7.15E-02 | 9.43E-01 |
| BNIP1 | 5 | 172571445 | 172591390 | 0.2520 | rs17075700 | 2.274 | rs35387209 | 0.264 | -10.100 | -0.058 | 93 | 11 | enet | 0.260 | 3.50E-26 | 6.97E-02 | 9.44E-01 |
| DARS2 | 1 | 173793641 | 173827684 | 0.3148 | rs941987 | 1.629 | rs2273366 | 0.330 | -11.290 | 0.479 | 59 | 29 | enet | 0.320 | 1.40E-33 | 6.71E-02 | 9.46E-01 |
| ATP5O | 21 | 35275757 | 35288284 | 0.2340 | rs2268251 | -1.930 | rs12482697 | 0.239 | -9.560 | 0.141 | 131 | 9 | enet | 0.220 | 2.30E-22 | -6.76E-02 | 9.46E-01 |
| ATP1B3 | 3 | 141594966 | 141645356 | 0.3007 | rs4683645 | -1.480 | rs10935443 | 0.302 | 10.700 | -0.102 | 95 | 17 | enet | 0.320 | 5.90E-33 | 6.75E-02 | 9.46E-01 |
| TBC1D5 | 3 | 17198654 | 18486309 | 0.1525 | rs7635386 | -2.570 | rs4685439 | 0.016 | 4.050 | 0.353 | 421 | 58 | enet | 0.031 | 3.30E-04 | 6.70E-02 | 9.47E-01 |
| PPP1R3G | 6 | 5085720 | 5087455 | 0.0780 | rs2764142 | 2.016 | rs591481 | 0.034 | -4.220 | -0.276 | 164 | 32 | enet | 0.015 | 1.60E-02 | -6.65E-02 | 9.47E-01 |
| DECR1 | 8 | 91013633 | 91064320 | 0.1614 | rs13312986 | -1.310 | rs1805890 | 0.059 | -6.460 | 0.248 | 99 | 13 | enet | 0.130 | 2.20E-13 | 6.60E-02 | 9.47E-01 |
| DIP2A | 21 | 47878812 | 47989926 | 0.0950 | rs743347 | 2.940 | rs11702704 | 0.057 | -4.840 | -0.383 | 161 | 17 | enet | 0.030 | 3.90E-04 | 6.54E-02 | 9.48E-01 |
| RANGAP1 | 22 | 41641615 | 41682255 | 0.0361 | rs12170305 | -4.090 | rs9611528 | 0.024 | 3.720 | 2.291 | 73 | 10 | enet | 0.029 | 5.30E-04 | 6.53E-02 | 9.48E-01 |
| ISYNA1 | 19 | 18545198 | 18549111 | 0.0189 | rs6413435 | 2.040 | rs34053595 | 0.023 | 3.620 | 0.106 | 71 | 6 | enet | 0.006 | 6.50E-02 | 6.50E-02 | 9.48E-01 |
| FSCN1 | 7 | 5632439 | 5646286 | 0.0441 | rs7805164 | 1.520 | rs4560713 | 0.015 | 3.560 | -0.239 | 76 | 8 | enet | 0.003 | 1.40E-01 | 6.49E-02 | 9.48E-01 |
| CRAT | 9 | 131857089 | 131873468 | 0.0313 | rs3750340 | 2.560 | rs7849270 | 0.042 | -4.300 | 0.108 | 65 | 9 | enet | 0.027 | 7.40E-04 | -6.43E-02 | 9.49E-01 |
| PRPSAP1 | 17 | 74305567 | 74380602 | 0.0977 | rs7207108 | 3.230 | rs8072202 | 0.005 | -3.600 | -0.922 | 122 | 29 | enet | 0.010 | 2.70E-02 | 6.10E-02 | 9.51E-01 |
| CHMP4A | 14 | 24678789 | 24683075 | 0.0716 | rs2748531 | 2.560 | rs6573607 | 0.044 | 5.190 | -0.134 | 96 | 20 | enet | 0.057 | 1.70E-06 | 5.98E-02 | 9.52E-01 |
| SLC39A12 | 10 | 18240768 | 18332221 | 0.1473 | rs2488155 | -2.360 | rs2165899 | 0.038 | -4.730 | 0.470 | 141 | 33 | enet | 0.066 | 3.50E-07 | 5.81E-02 | 9.54E-01 |
| PYCRL | 8 | 144686083 | 144691943 | 0.2781 | rs7829491 | -1.760 | rs11549789 | 0.130 | -7.040 | 1.443 | 87 | 12 | enet | 0.180 | 6.40E-18 | -5.71E-02 | 9.54E-01 |
| RAB27B | 18 | 52385091 | 52562747 | 0.1618 | rs6566847 | 2.410 | rs2871673 | 0.192 | -8.630 | 0.757 | 132 | 35 | enet | 0.180 | 1.70E-18 | -5.67E-02 | 9.55E-01 |
| MTRF1L | 6 | 153308497 | 153323820 | 0.1418 | rs7453812 | 3.168 | rs766127 | 0.172 | -7.620 | 0.131 | 146 | 11 | enet | 0.150 | 1.30E-12 | 5.61E-02 | 9.55E-01 |
| PPP1R14C | 6 | 150464212 | 150571493 | 0.0935 | rs9384388 | 2.448 | rs9397821 | 0.002 | -3.850 | -0.496 | 210 | 25 | enet | 0.010 | 3.10E-02 | 5.62E-02 | 9.55E-01 |
| CDH13 | 16 | 82660408 | 83830204 | 0.2529 | rs7190148 | 3.322 | rs9933518 | 0.017 | 5.560 | -1.729 | 1385 | 70 | enet | 0.088 | 2.40E-09 | 5.60E-02 | 9.55E-01 |
| VPS36 | 13 | 52986737 | 53024763 | 0.2654 | rs4286007 | -1.590 | rs12430144 | 0.105 | -6.430 | 0.925 | 49 | 14 | enet | 0.120 | 7.50E-12 | 5.55E-02 | 9.56E-01 |
| HYI | 1 | 43916824 | 43919660 | 0.3343 | rs12039931 | -2.033 | rs6954 | 0.225 | 9.260 | 0.489 | 77 | 15 | enet | 0.260 | 1.20E-26 | -5.45E-02 | 9.56E-01 |
| TPMT | 6 | 18128542 | 18155305 | 0.5865 | rs9383343 | 2.247 | rs1142345 | 0.489 | -14.330 | -0.012 | 109 | 20 | enet | 0.540 | 1.20E-65 | 5.48E-02 | 9.56E-01 |
| DNM1 | 9 | 130965658 | 131017527 | 0.0329 | rs1004464 | 2.170 | rs2502731 | 0.022 | 4.090 | -0.403 | 81 | 7 | enet | 0.025 | 1.20E-03 | 5.49E-02 | 9.56E-01 |
| CLYBL | 13 | 100258919 | 100549387 | 0.7437 | rs9513627 | -3.480 | rs3783185 | 0.489 | 13.590 | 0.202 | 278 | 53 | enet | 0.550 | 2.90E-67 | 5.46E-02 | 9.57E-01 |
| CRYM | 16 | 21250195 | 21314404 | 0.0848 | rs7194021 | 1.944 | rs741720 | 0.034 | -4.300 | -0.523 | 81 | 13 | enet | 0.052 | 4.30E-06 | -5.40E-02 | 9.57E-01 |
| GLRX3 | 10 | 131934663 | 131982785 | 0.0975 | rs1183343 | -2.490 | rs11017104 | 0.095 | -6.090 | 0.337 | 154 | 15 | enet | 0.072 | 7.80E-08 | -5.05E-02 | 9.60E-01 |
| SARS | 1 | 109756540 | 109780791 | 0.0882 | rs17641881 | -2.069 | rs14000 | 0.010 | -3.870 | -0.679 | 116 | 9 | enet | 0.007 | 5.80E-02 | 4.84E-02 | 9.61E-01 |
| ABCC8 | 11 | 17414432 | 17498449 | 0.1081 | rs10832796 | -2.010 | rs4757512 | 0.098 | -6.450 | -0.462 | 221 | 8 | enet | 0.110 | 3.00E-10 | -4.88E-02 | 9.61E-01 |
| OSBP2 | 22 | 31089769 | 31303811 | 0.0896 | rs136233 | 3.240 | rs3804085 | 0.052 | -4.880 | 0.385 | 160 | 18 | enet | 0.029 | 5.50E-04 | 4.79E-02 | 9.62E-01 |
| ISOC1 | 5 | 128430444 | 128449721 | 0.0839 | rs7709280 | 1.983 | rs3756747 | 0.039 | 4.630 | -0.539 | 89 | 12 | enet | 0.047 | 1.30E-05 | -4.59E-02 | 9.63E-01 |
| GSTM1 | 1 | 110230436 | 110251661 | 0.5124 | rs11102002 | -2.673 | rs3754446 | 0.141 | -7.400 | 0.411 | 101 | 30 | enet | 0.350 | 2.70E-37 | 4.53E-02 | 9.64E-01 |
| PLCB3 | 11 | 64018995 | 64036622 | 0.0481 | rs479777 | -4.140 | rs11600990 | 0.024 | 4.330 | 0.067 | 81 | 13 | enet | 0.015 | 1.00E-02 | 4.46E-02 | 9.64E-01 |
| ZADH2 | 18 | 72907063 | 72921303 | 0.2186 | rs7237556 | -2.110 | rs7230037 | 0.196 | -8.960 | -0.038 | 136 | 18 | enet | 0.180 | 3.30E-18 | 4.50E-02 | 9.64E-01 |
| HADH | 4 | 108910870 | 108956331 | 0.2220 | rs9992744 | 5.410 | rs6831530 | 0.022 | 4.380 | 1.427 | 94 | 13 | enet | 0.071 | 8.50E-08 | 4.46E-02 | 9.64E-01 |
| CXADR | 21 | 18884700 | 18965897 | 0.0658 | rs9808661 | 2.840 | rs2824350 | 0.015 | 4.240 | 0.011 | 138 | 9 | enet | -0.002 | 5.50E-01 | 4.43E-02 | 9.65E-01 |
| ACADSB | 10 | 124768495 | 124817827 | 0.1016 | rs1106056 | 2.410 | rs7894710 | 0.040 | -4.870 | -0.251 | 131 | 19 | enet | 0.073 | 5.70E-08 | -4.41E-02 | 9.65E-01 |
| MAP1S | 19 | 17830051 | 17845325 | 0.1678 | rs6512209 | 2.500 | rs12979056 | 0.150 | -8.460 | -0.064 | 91 | 13 | enet | 0.220 | 2.50E-22 | 4.39E-02 | 9.65E-01 |
| TXNDC17 | 17 | 6544078 | 6547861 | 0.0775 | rs356053 | 2.280 | rs2165844 | 0.045 | 5.490 | -0.402 | 122 | 16 | enet | 0.049 | 7.90E-06 | -4.31E-02 | 9.66E-01 |
| ACP1 | 2 | 264140 | 278283 | 0.3809 | rs6719944 | 1.638 | rs2290911 | 0.606 | -15.140 | 0.042 | 96 | 18 | enet | 0.610 | 1.50E-78 | -4.12E-02 | 9.67E-01 |

| | | | | | | | | | | | | | | | | | |
|---|---|---|---|---|---|---|---|---|---|---|---|---|---|---|---|---|---|
| KDELC2 | 11 | 108342832 | 108369159 | 0.0490 | rs11212672 | -2.710 | rs3741060 | 0.033 | -3.620 | -0.736 | 68 | 21 | enet | 0.050 | 8.20E-05 | 4.16E-02 | 9.67E-01 |
| HAGH | 16 | 1845621 | 1877195 | 0.3143 | rs1742398 | -1.406 | rs10500323 | 0.308 | 10.820 | -0.009 | 134 | 12 | enet | 0.340 | 4.00E-36 | 4.15E-02 | 9.67E-01 |
| DPCD | 10 | 103330317 | 103369425 | 0.1143 | rs4151060 | -2.070 | rs7006 | 0.054 | 4.490 | 0.337 | 68 | 12 | enet | 0.036 | 2.70E-04 | -4.07E-02 | 9.68E-01 |
| CLPP | 19 | 6361463 | 6368919 | 0.1475 | rs10405167 | -1.660 | rs17625356 | 0.092 | 6.350 | -0.132 | 82 | 14 | enet | 0.110 | 8.90E-11 | -4.02E-02 | 9.68E-01 |
| SPATA20 | 17 | 48620419 | 48633213 | 0.6707 | rs4794175 | -1.900 | rs878619 | 0.400 | -12.610 | -0.372 | 102 | 35 | enet | 0.640 | 2.40E-85 | -4.04E-02 | 9.68E-01 |
| NDUFA6 | 22 | 42481529 | 42486959 | 0.0944 | rs5758563 | -2.310 | rs5751204 | 0.104 | -6.530 | -0.511 | 61 | 11 | enet | 0.110 | 3.50E-11 | 4.01E-02 | 9.68E-01 |
| AKR1B1 | 7 | 134127102 | 134144036 | 0.1465 | rs17773014 | 2.680 | rs918825 | 0.041 | 5.460 | 0.613 | 128 | 16 | enet | 0.099 | 2.30E-10 | -3.92E-02 | 9.69E-01 |
| INPP4B | 4 | 142944313 | 143768585 | 0.0582 | rs3756125 | 2.630 | rs331961 | 0.038 | -4.440 | -0.114 | 370 | 15 | enet | 0.012 | 2.20E-02 | 3.72E-02 | 9.70E-01 |
| SERAC1 | 6 | 158530536 | 158589312 | 0.1523 | rs9295289 | -2.537 | rs13202019 | 0.051 | -4.820 | 1.302 | 141 | 34 | enet | 0.057 | 1.50E-06 | 3.69E-02 | 9.71E-01 |
| LSM6 | 4 | 147096837 | 147121152 | 0.0513 | rs11100914 | 1.720 | rs4835286 | 0.057 | 5.430 | -0.164 | 104 | 7 | enet | 0.039 | 6.50E-05 | 3.58E-02 | 9.72E-01 |
| PDXK | 21 | 45138975 | 45182188 | 0.2006 | rs4818858 | 3.020 | rs1107204 | 0.010 | -3.650 | 0.356 | 117 | 15 | enet | 0.041 | 4.60E-05 | -3.56E-02 | 9.72E-01 |
| TSPAN15 | 10 | 71211229 | 71267425 | 0.0513 | rs990394 | 2.530 | rs12776158 | 0.000 | 3.570 | -0.453 | 165 | 17 | enet | -0.001 | 4.00E-01 | -3.47E-02 | 9.72E-01 |
| TPP2 | 13 | 103249353 | 103331521 | 0.0500 | rs17507827 | -2.300 | rs660207 | 0.045 | -5.360 | 0.029 | 192 | 7 | enet | 0.032 | 3.10E-04 | -3.42E-02 | 9.73E-01 |
| STAU1 | 20 | 47729878 | 47804904 | 0.0379 | rs1567865 | -1.247 | rs6066968 | 0.035 | 4.170 | 0.069 | 92 | 8 | enet | 0.014 | 1.30E-02 | 3.37E-02 | 9.73E-01 |
| CALCOCO1 | 12 | 54104903 | 54121529 | 0.1133 | rs10876496 | 2.750 | rs3741659 | 0.103 | 6.480 | -0.182 | 102 | 10 | enet | 0.080 | 1.20E-08 | -3.25E-02 | 9.74E-01 |
| ASAH1 | 8 | 17913934 | 17942494 | 0.0768 | rs1234372 | -2.190 | rs4377998 | 0.097 | -6.160 | 0.229 | 180 | 16 | enet | 0.088 | 3.20E-09 | 3.08E-02 | 9.75E-01 |
| GANAB | 11 | 62392298 | 62414104 | 0.2275 | rs35587183 | -2.140 | rs1058678 | 0.086 | -5.810 | 0.136 | 56 | 15 | enet | 0.070 | 1.20E-07 | 3.03E-02 | 9.76E-01 |
| BCAT1 | 12 | 24964295 | 25102393 | 0.2449 | rs3736212 | -2.650 | rs1872644 | 0.087 | -6.020 | 0.830 | 204 | 44 | enet | 0.099 | 2.50E-10 | 2.95E-02 | 9.76E-01 |
| GDE1 | 16 | 19513015 | 19533467 | 0.0452 | rs13338968 | 1.932 | rs2187659 | 0.052 | -4.680 | 0.516 | 117 | 6 | enet | 0.034 | 1.70E-04 | 3.02E-02 | 9.76E-01 |
| DYSF | 2 | 71680852 | 71913898 | 0.0800 | rs7598653 | 2.874 | rs1459266 | 0.009 | -3.800 | 0.191 | 218 | 23 | enet | 0.016 | 7.50E-03 | 2.87E-02 | 9.77E-01 |
| LPA | 6 | 160952515 | 161087407 | 0.1105 | rs6919346 | 2.536 | rs783147 | 0.007 | -3.200 | 0.676 | 128 | 22 | enet | 0.017 | 3.00E-02 | 2.71E-02 | 9.78E-01 |
| CARS | 11 | 3022152 | 3078843 | 0.0511 | rs2411767 | -2.780 | rs4758506 | 0.016 | 3.920 | 0.857 | 142 | 19 | enet | 0.007 | 5.40E-02 | -2.79E-02 | 9.78E-01 |
| SLC24A4 | 14 | 92788925 | 92962596 | 0.1062 | rs7142428 | 2.850 | rs8017062 | -0.002 | -2.930 | 0.770 | 221 | 23 | enet | -0.001 | 4.60E-01 | 2.75E-02 | 9.78E-01 |
| C6orf57 | 6 | 71276620 | 71299272 | 0.0364 | rs1524738 | -1.839 | rs6455371 | 0.019 | -4.100 | 0.306 | 76 | 5 | enet | 0.007 | 5.30E-02 | 2.61E-02 | 9.79E-01 |
| FABP1 | 2 | 88422510 | 88427635 | 0.2692 | rs4972169 | -2.361 | rs17350803 | 0.204 | 7.710 | 0.159 | 120 | 18 | enet | 0.210 | 2.90E-16 | -2.56E-02 | 9.80E-01 |
| BPNT1 | 1 | 220230824 | 220263804 | 0.0522 | rs2808027 | -2.190 | rs17563262 | 0.059 | 5.070 | -0.113 | 93 | 6 | enet | 0.054 | 2.80E-06 | 2.32E-02 | 9.81E-01 |
| PFKM | 12 | 48498922 | 48540187 | 0.2282 | rs10747531 | -3.470 | rs886588 | 0.198 | -9.390 | -0.208 | 132 | 17 | enet | 0.220 | 2.00E-22 | 2.42E-02 | 9.81E-01 |
| HNMT | 2 | 138721590 | 138773930 | 0.2178 | rs17646809 | -2.301 | rs3100720 | 0.286 | -10.590 | -0.187 | 125 | 30 | enet | 0.300 | 1.20E-31 | -2.32E-02 | 9.82E-01 |
| MOXD1 | 6 | 132617194 | 132722684 | 0.1266 | rs6900501 | 2.810 | rs11154669 | 0.003 | 3.800 | -1.280 | 133 | 39 | enet | 0.031 | 3.70E-04 | -2.32E-02 | 9.82E-01 |
| TAB1 | 22 | 39795746 | 39833065 | 0.1476 | rs3827380 | 4.160 | rs35515473 | 0.058 | -4.820 | -0.894 | 99 | 11 | enet | 0.044 | 2.60E-05 | -2.25E-02 | 9.82E-01 |
| ARMC9 | 2 | 232063260 | 232239548 | 0.0836 | rs3806546 | 2.261 | rs4621162 | 0.025 | -3.930 | -0.832 | 142 | 34 | enet | 0.012 | 1.70E-02 | -2.10E-02 | 9.83E-01 |
| ACOT11 | 1 | 55007930 | 55104865 | 0.3431 | rs17398958 | -2.339 | rs1368883 | 0.314 | 11.100 | -0.003 | 159 | 12 | enet | 0.340 | 3.80E-34 | 2.02E-02 | 9.84E-01 |
| RWDD1 | 6 | 116892530 | 116918838 | 0.0647 | rs10457308 | 2.834 | rs11961321 | 0.067 | -6.310 | -0.066 | 74 | 9 | enet | 0.067 | 2.30E-07 | 1.96E-02 | 9.84E-01 |
| TARSL2 | 15 | 102193801 | 102264807 | 0.1073 | rs4965898 | -2.600 | rs7495447 | 0.142 | 7.990 | 0.237 | 104 | 15 | enet | 0.130 | 2.70E-13 | 1.94E-02 | 9.85E-01 |
| CTNNAL1 | 9 | 111704851 | 111775809 | 0.0597 | rs7034770 | 3.460 | rs3750457 | 0.033 | 4.160 | -0.035 | 164 | 10 | enet | 0.012 | 3.20E-02 | -1.84E-02 | 9.85E-01 |
| SLC41A3 | 3 | 125725198 | 125820404 | 0.0481 | rs10934751 | 3.710 | rs2003334 | 0.016 | -4.620 | -1.885 | 149 | 17 | enet | 0.021 | 3.30E-03 | 1.79E-02 | 9.86E-01 |
| PAAF1 | 11 | 73587744 | 73638790 | 0.5655 | rs1792174 | -2.230 | rs643064 | 0.000 | -3.520 | -0.126 | 98 | 57 | enet | 0.180 | 5.40E-18 | 1.73E-02 | 9.86E-01 |
| SNX19 | 11 | 130745331 | 130786404 | 0.0946 | rs11222312 | 2.100 | rs6590535 | 0.092 | -5.800 | -0.815 | 145 | 21 | enet | 0.075 | 2.70E-07 | -1.44E-02 | 9.89E-01 |
| DNAJC11 | 1 | 6694228 | 6761984 | 0.2359 | rs11122122 | 2.558 | rs200448 | 0.094 | 6.780 | 1.170 | 109 | 20 | enet | 0.200 | 5.60E-20 | 1.12E-02 | 9.91E-01 |
| UCKL1 | 20 | 62571186 | 62587769 | 0.1036 | rs11905801 | -3.959 | rs6062599 | 0.017 | 4.480 | 0.140 | 85 | 6 | enet | 0.049 | 1.10E-05 | -1.01E-02 | 9.92E-01 |
| PCNT | 21 | 47744036 | 47865682 | 0.0773 | rs17296821 | 2.990 | rs2839313 | 0.021 | 3.860 | -0.904 | 152 | 12 | enet | 0.036 | 1.20E-04 | 9.01E-03 | 9.93E-01 |
| ATG7 | 3 | 11313995 | 11599139 | 0.1779 | rs4684787 | 2.250 | rs367745 | 0.161 | -8.050 | -0.223 | 200 | 22 | enet | 0.140 | 3.10E-14 | -8.31E-03 | 9.93E-01 |
| RASAL1 | 12 | 113536624 | 113574044 | 0.0820 | rs2701623 | -2.040 | rs1674093 | 0.002 | 3.240 | 0.324 | 91 | 23 | enet | 0.033 | 2.40E-04 | 7.98E-03 | 9.94E-01 |
| PRMT3 | 11 | 20409076 | 20530840 | 0.0401 | rs7112050 | 2.250 | rs16906047 | 0.002 | 3.600 | -0.458 | 169 | 15 | enet | -0.001 | 4.20E-01 | 6.54E-03 | 9.95E-01 |
| SPRYD4 | 12 | 56862301 | 56864763 | 0.1770 | rs2638319 | -3.110 | rs1043011 | 0.151 | 7.710 | 0.851 | 64 | 11 | enet | 0.130 | 2.70E-13 | 5.66E-03 | 9.95E-01 |
| KIAA1161 | 9 | 34366668 | 34376851 | 0.2713 | rs4256660 | 3.530 | rs2381117 | 0.264 | -9.540 | -0.738 | 74 | 9 | enet | 0.280 | 2.40E-24 | -6.16E-03 | 9.95E-01 |

| | | | | | | | | | | | | | | | | | |
|---|---|---|---|---|---|---|---|---|---|---|---|---|---|---|---|---|---|
| EIF2A | 3 | 150264465 | 150302029 | 0.0671 | rs13081536 | -1.970 | rs17280260 | 0.016 | 4.010 | 0.964 | 93 | 18 | enet | 0.040 | 5.70E-05 | 5.51E-03 | 9.96E-01 |
| TIMM9 | 14 | 58875212 | 58894332 | 0.0654 | rs8014077 | 2.700 | rs10147224 | 0.061 | 5.160 | 0.372 | 37 | 11 | enet | 0.055 | 2.30E-06 | 2.47E-03 | 9.98E-01 |
| ATP5S | 14 | 50779044 | 50802276 | 0.5619 | rs4901030 | 2.060 | rs2356243 | 0.205 | -9.070 | -0.688 | 93 | 34 | enet | 0.280 | 1.30E-28 | 1.61E-03 | 9.99E-01 |
| FUK | 16 | 70488324 | 70514177 | 0.2283 | rs8059245 | -1.588 | rs17882961 | 0.022 | 3.780 | -0.453 | 43 | 17 | enet | 0.009 | 3.60E-02 | 9.51E-04 | 9.99E-01 |
| CHRM3 | 1 | 239549865 | 240078750 | 0.0760 | NA | NA | NA | NA | NA | NA | 0 | 0 | enet | 0.002 | 1.70E-01 | NA | NA |
| DTYMK | 2 | 242615157 | 242626406 | 0.1050 | NA | NA | NA | NA | NA | NA | 33 | 0 | enet | 0.044 | 2.30E-05 | NA | NA |
| ATG4B | 2 | 242576628 | 242613272 | 0.1264 | NA | NA | NA | NA | NA | NA | 46 | 0 | enet | 0.088 | 2.70E-09 | NA | NA |
| RAPH1 | 2 | 204259068 | 204400133 | 0.0346 | NA | NA | NA | NA | NA | NA | 0 | 0 | enet | -0.001 | 4.00E-01 | NA | NA |
| D2HGDH | 2 | 242673994 | 242708231 | 0.3675 | NA | NA | NA | NA | NA | NA | 22 | 0 | enet | 0.370 | 3.50E-39 | NA | NA |
| CISD2 | 4 | 103790135 | 103810399 | 0.0830 | NA | NA | NA | NA | NA | NA | 43 | 0 | enet | 0.110 | 4.10E-11 | NA | NA |
| SYNPO2 | 4 | 119809996 | 119982402 | 0.0680 | NA | NA | NA | NA | NA | NA | 0 | 0 | enet | 0.003 | 1.30E-01 | NA | NA |
| HSP90AB1 | 6 | 44214824 | 44221620 | 0.0729 | NA | NA | NA | NA | NA | NA | 0 | 0 | enet | -0.001 | 4.80E-01 | NA | NA |
| MLLT4 | 6 | 168227602 | 168372703 | 0.0417 | NA | NA | NA | NA | NA | NA | 0 | 0 | enet | -0.002 | 6.90E-01 | NA | NA |
| SEMA4D | 9 | 91975702 | 92113045 | 0.0580 | NA | NA | NA | NA | NA | NA | 0 | 0 | enet | 0.016 | 7.30E-03 | NA | NA |
| AQP11 | 11 | 77300436 | 77321400 | 0.0493 | NA | NA | NA | NA | NA | NA | 0 | 0 | enet | 0.008 | 1.00E-01 | NA | NA |
| IGHA2 | 14 | 106053226 | 106054732 | 0.0915 | NA | NA | NA | NA | NA | NA | 13 | 0 | enet | 0.031 | 1.10E-03 | NA | NA |
| ADAM10 | 15 | 58887403 | 59042177 | 0.0724 | NA | NA | NA | NA | NA | NA | 0 | 0 | enet | -0.003 | 9.70E-01 | NA | NA |
| NXN | 17 | 702553 | 883010 | 0.0765 | NA | NA | NA | NA | NA | NA | 0 | 0 | enet | -0.001 | 4.60E-01 | NA | NA |

PWAS: proteome-wide association study; pQTL: protein quantitative trait loci; Chr: chromosome; P0: the start position of the gene; P1: the end position of the gene; HSQ: heritability of the gene; BEST.GWAS.ID: rsID of the most significant GWAS SNP in locus; BEST.GWAS.Z: Z-score of the most significant GWAS SNP in locus; PQTL.ID: rsID of the best pQTL in the locus; PQTL.R2: cross-validation $R^2$ of the best pQTL in the locus; PQTL.Z: Z-score of the best pQTL in the locus; PQTL.GWAS.Z: GWAS Z-score for this pQTL; MODELCV.R2: cross-validation $R^2$ of the best performing model; MODELCV.PV: cross-validation P-value of the best performing model; PWAS.Z: PWAS Z-score; PWAS.P: PWAS P-value.

**Table S3. SMR analysis identifying plasma proteins causally associated with multiple sclerosis, based on candidate proteins determined by PWAS**

| ProbeID | Probe chromosome | Gene | Protein | UniProt | Probe position | TopSNP | TopSNP_chromosome | TopSNP_position | A1 | A2 | Freq | Beta_GWAS | SE_GWAS | P_GWAS | Beta_pQTL | SE_pQTL | P_pQTL | Beta_SMR | SE_SMR | OR_SMR | 95%CI_SMR | P_SMR | FDR_adjusted_P_SMR | P_HEIDI | $N_{SNP}$_HEIDI |
|---|---|---|---|---|---|---|---|---|---|---|---|---|---|---|---|---|---|---|---|---|---|---|---|---|---|
| SeqId_3186_2 | 6 | C2 | C2 | P06681 | 32299822 | rs9268149 | 6 | 32263099 | T | C | 0.1605 | 0.9362 | 0.0253 | 1.00E-299 | -0.3388 | 0.0216 | 1.58E-55 | -2.763 | 0.191 | 0.063 | (0.043-0.092) | 2.47E-47 | 2.47E-45 | 5.91E-21 | 20 |
| SeqId_7757_5 | 6 | HLA-DQA2 | DQA2 | P01906 | 32631029 | rs9272014 | 6 | 32598262 | A | G | 0.2832 | -0.5699 | 0.0380 | 8.95E-51 | -0.6166 | 0.0180 | 4.97E-257 | 0.924 | 0.067 | 2.520 | (2.208-2.875) | 6.74E-43 | 2.47E-45 | 8.21E-173 | 20 |
| SeqId_11387_3 | 6 | ATF6B | ATF6B | Q99941 | 32299822 | rs6449 | 6 | 32006655 | T | C | 0.3027 | -1.0137 | 0.0362 | 1.85E-172 | 0.2722 | 0.0181 | 5.67E-51 | -3.725 | 0.281 | 0.024 | (0.014-0.042) | 5.63E-40 | 3.37E-41 | 5.57E-16 | 20 |
| SeqId_4125_52 | 6 | AGER | sRAGE | Q15109 | 32631029 | rs2070600 | 6 | 32151443 | T | C | 0.0521 | -0.6471 | 0.0479 | 1.40E-41 | -0.7418 | 0.0388 | 2.80E-81 | 0.872 | 0.079 | 2.393 | (2.049-2.794) | 2.81E-28 | 1.88E-38 | 2.25E-17 | 20 |
| SeqId_2730_58 | 6 | MICA | MICA | Q29983 | 31132414 | rs7775759 | 6 | 31352446 | A | G | 0.3712 | -0.1509 | 0.0181 | 8.35E-17 | -0.6490 | 0.0173 | 1.10E-301 | 0.233 | 0.029 | 1.262 | (1.193-1.335) | 4.34E-16 | 7.02E-27 | 9.39E-10 | 20 |
| SeqId_3003_29 | 6 | NCR3 | NKp30 | O14931 | 31132414 | rs986475 | 6 | 31556709 | G | A | 0.0879 | -0.2825 | 0.0335 | 3.63E-17 | -0.6205 | 0.0280 | 6.64E-109 | 0.455 | 0.058 | 1.577 | (1.408-1.766) | 3.41E-15 | 8.67E-15 | 4.08E-16 | 20 |
| SeqId_10346_5 | 17 | STAT3 | STAT3 | P40763 | 40727427 | rs4796791 | 17 | 40530763 | T | C | 0.3701 | 0.1295 | 0.0175 | 1.18E-13 | -0.4289 | 0.0171 | 2.72E-138 | -0.302 | 0.042 | 0.739 | (0.68-0.803) | 1.13E-12 | 5.68E-14 | 2.35E-02 | 20 |
| SeqId_2654_19 | 12 | TNFRSF1A | TNF sR-I | P19438 | 6601743 | rs1800693 | 12 | 6440009 | C | T | 0.4274 | 0.1269 | 0.0171 | 1.02E-13 | -0.1919 | 0.0167 | 2.10E-30 | -0.661 | 0.106 | 0.516 | (0.419-0.635) | 4.39E-10 | 1.61E-11 | 9.21E-02 | 20 |
| SeqId_4440_15 | 1 | FCRL3 | FCRL3 | Q96P31 | 157255396 | rs6427397 | 1 | 157705725 | T | C | 0.4499 | -0.0963 | 0.0170 | 1.37E-08 | 0.6073 | 0.0162 | 1.10E-301 | -0.159 | 0.028 | 0.853 | (0.807-0.902) | 1.98E-08 | 5.49E-09 | 1.63E-01 | 20 |
| SeqId_7875_86 | 2 | PLEK | PLEK | P08567 | 68333616 | rs1867312 | 2 | 68619981 | C | A | 0.4254 | -0.0937 | 0.0164 | 1.20E-08 | -0.4435 | 0.0165 | 3.92E-159 | 0.211 | 0.038 | 1.235 | (1.147-1.33) | 2.47E-08 | 2.20E-07 | 4.28E-03 | 20 |
| SeqId_12378_71 | 6 | TAPBP | TPSN | O15533 | 32852448 | rs469064 | 6 | 33250476 | C | A | 0.4949 | 0.0932 | 0.0164 | 1.40E-08 | 0.4512 | 0.0161 | 9.14E-173 | 0.207 | 0.037 | 1.229 | (1.143-1.322) | 2.69E-08 | 2.44E-07 | 8.21E-09 | 20 |
| SeqId_11278_4 | 6 | COL11A2 | COBA2 | P13942 | 32852448 | rs3129205 | 6 | 33125859 | T | G | 0.3497 | 0.0981 | 0.0174 | 1.72E-08 | 0.3817 | 0.0171 | 7.99E-111 | 0.257 | 0.047 | 1.293 | (1.179-1.418) | 4.58E-08 | 2.44E-07 | 1.76E-21 | 20 |
| SeqId_11361_73 | 22 | TYMP | TP | P19971 | 51138753 | rs131805 | 22 | 50964153 | T | C | 0.2249 | 0.1100 | 0.0198 | 2.61E-08 | -0.3626 | 0.0198 | 2.87E-75 | -0.304 | 0.057 | 0.738 | (0.66-0.825) | 1.00E-07 | 3.81E-07 | 1.28E-01 | 20 |
| SeqId_4129_72 | 6 | CFB | Factor B | P00751 | 32299822 | rs541862 | 6 | 31916951 | C | T | 0.0879 | -0.1408 | 0.0299 | 2.56E-06 | 0.8163 | 0.0277 | 1.23E-190 | -0.172 | 0.037 | 0.842 | (0.782-0.905) | 3.41E-06 | 7.71E-07 | 4.51E-07 | 20 |
| SeqId_8973_23 | 1 | FCRL4 | FCRL4 | Q96PJ5 | 157255396 | rs9427292 | 1 | 157565223 | G | T | 0.2485 | 0.0968 | 0.0220 | 1.11E-05 | -0.6808 | 0.0181 | 1.10E-301 | -0.142 | 0.033 | 0.867 | (0.814-0.925) | 1.28E-05 | 2.44E-05 | 2.39E-01 | 20 |
| SeqId_3151_6 | 10 | IL2RA | IL-2 sRa | P01589 | 5685209 | rs12722497 | 10 | 6095928 | A | C | 0.1452 | 0.1228 | 0.0264 | 3.20E-06 | 0.2282 | 0.0285 | 1.09E-15 | 0.538 | 0.134 | 1.713 | (1.318-2.226) | 5.64E-05 | 8.51E-05 | 1.44E-01 | 20 |
| SeqId_2855_49 | 16 | MAPK3 | ERK-1 | P27361 | 29927702 | rs9932466 | 16 | 30141847 | T | C | 0.3405 | -0.0767 | 0.0193 | 6.93E-05 | 0.6286 | 0.0168 | 5.17E-301 | -0.122 | 0.031 | 0.885 | (0.833-0.94) | 7.61E-05 | 3.53E-04 | 4.42E-02 | 20 |
| SeqId_19556_12 | 1 | CR1 | Complement receptor type 1 | P17927 | 208129798 | rs679515 | 1 | 207750568 | T | C | 0.1718 | 0.0806 | 0.0215 | 1.81E-04 | 0.6182 | 0.0208 | 3.18E-193 | 0.130 | 0.035 | 1.139 | (1.063-1.22) | 2.03E-04 | 4.48E-04 | 1.67E-01 | 20 |
| SeqId_9870_17 | 14 | WARS | SYWC | P23381 | 101178330 | rs4905957 | 14 | 100837230 | C | T | 0.2137 | -0.0707 | 0.0191 | 2.21E-04 | -0.6326 | 0.0186 | 5.77E-254 | 0.112 | 0.030 | 1.118 | (1.054-1.187) | 2.40E-04 | 1.13E-03 | 3.37E-01 | 20 |
| SeqId_10938_13 | 1 | CD58 | sLFA-3 | P19256 | 117555012 | rs60612523 | 1 | 117059709 | G | A | 0.0204 | -0.2805 | 0.0722 | 1.03E-04 | 0.7048 | 0.0649 | 1.95E-27 | -0.398 | 0.109 | 0.672 | (0.543-0.831) | 2.57E-04 | 1.26E-03 | 8.40E-01 | 20 |
| SeqId_3329_14 | 19 | PGLYRP1 | PGRP-S | O75594 | 46728857 | rs2072563 | 19 | 46526648 | A | G | 0.2740 | 0.0700 | 0.0183 | 1.34E-04 | -0.2137 | 0.0179 | 6.66E-33 | -0.327 | 0.090 | 0.721 | (0.604-0.86) | 2.75E-04 | 1.28E-03 | 3.96E-01 | 20 |
| SeqId_5728_60 | 1 | FCRL1 | FCRL1 | Q96LA6 | 157672304 | rs4971154 | 1 | 157771880 | C | T | 0.5061 | -0.0601 | 0.0165 | 2.61E-04 | 0.4075 | 0.0163 | 1.16E-138 | -0.147 | 0.041 | 0.863 | (0.797-0.935) | 3.02E-04 | 1.31E-03 | 2.66E-06 | 20 |
| SeqId_3169_70 | 4 | IDUA | IDUA | P35475 | 1357325 | rs3796622 | 4 | 983060 | T | C | 0.3558 | 0.0635 | 0.0177 | 3.38E-04 | -0.6237 | 0.0166 | 2.99E-301 | -0.102 | 0.029 | 0.903 | (0.854-0.955) | 3.59E-04 | 1.37E-03 | 7.56E-01 | 20 |
| SeqId_11514_196 | 11 | CD59 | CD59 | P13987 | 33814061 | rs831630 | 11 | 33743019 | T | C | 0.2945 | 0.0621 | 0.0175 | 3.77E-04 | -0.3943 | 0.0175 | 4.17E-112 | -0.157 | 0.045 | 0.854 | (0.782-0.933) | 4.44E-04 | 1.56E-03 | 1.66E-01 | 20 |
| SeqId_2579_17 | 20 | MMP9 | MMP-9 | P14780 | 45092921 | rs8113877 | 20 | 44635045 | G | T | 0.4264 | -0.0554 | 0.0167 | 9.28E-04 | 0.2769 | 0.0168 | 1.11E-60 | -0.200 | 0.062 | 0.819 | (0.725-0.924) | 1.17E-03 | 1.85E-03 | 1.20E-02 | 20 |
| SeqId_11955_1 | 11 | ARHGAP1 | RHG01 | Q07960 | 46849360 | rs11039024 | 11 | 46923168 | T | C | 0.1288 | 0.0890 | 0.0230 | 1.05E-04 | -0.1386 | 0.0241 | 8.28E-09 | -0.643 | 0.200 | 0.526 | (0.356-0.778) | 1.29E-03 | 4.68E-03 | 6.10E-01 | 20 |
| SeqId_12558_3 | 11 | UBASH3B | UBS3B | Q8TF42 | 122588149 | rs3937027 | 11 | 122525773 | A | C | 0.2566 | 0.0628 | 0.0193 | 1.12E-03 | -0.3676 | 0.0191 | 1.67E-82 | -0.171 | 0.053 | 0.843 | (0.759-0.936) | 1.31E-03 | 4.86E-03 | 2.36E-02 | 20 |
| SeqId_6388_21 | 7 | CCDC126 | CC126 | Q96EE4 | 24084202 | rs35121828 | 7 | 23634985 | A | G | 0.2301 | -0.0641 | 0.0199 | 1.27E-03 | 0.4975 | 0.0186 | 2.40E-157 | -0.129 | 0.040 | 0.879 | (0.812-0.951) | 1.38E-03 | 4.86E-03 | 5.31E-01 | 20 |
| SeqId_4124_24 | 6 | HSPA1A | HSP 70 | P0DMV8 | 32153409 | rs3869145 | 6 | 31851354 | A | G | 0.0307 | -0.2038 | 0.0577 | 4.11E-04 | -0.2869 | 0.0413 | 3.84E-12 | 0.710 | 0.226 | 2.035 | (1.308-3.166) | 1.64E-03 | 4.92E-03 | 7.81E-06 | 20 |
| SeqId_4925_54 | 11 | MMP13 | MMP-13 | P45452 | 102701021 | rs655316 | 11 | 102832364 | T | C | 0.2955 | 0.0594 | 0.0173 | 5.74E-04 | 0.1327 | 0.0177 | 7.41E-14 | 0.448 | 0.143 | 1.565 | (1.182-2.072) | 1.76E-03 | 5.65E-03 | 9.45E-01 | 20 |
| SeqId_13488_3 | 4 | ARFIP1 | ARFP1 | P53367 | 154055176 | rs4619875 | 4 | 153701130 | T | C | 0.4121 | -0.0523 | 0.0169 | 1.95E-03 | 0.4250 | 0.0166 | 5.61E-145 | -0.123 | 0.040 | 0.884 | (0.817-0.956) | 2.11E-03 | 5.86E-03 | 1.10E-01 | 20 |
| SeqId_3593_72 | 4 | CASP3 | Caspase-3 | P42574 | 185412635 | rs72689255 | 4 | 185590141 | C | T | 0.1145 | -0.0731 | 0.0246 | 2.95E-03 | -0.7283 | 0.0245 | 2.13E-193 | 0.100 | 0.034 | 1.106 | (1.034-1.182) | 3.09E-03 | 6.80E-03 | 4.60E-01 | 20 |
| SeqId_3581_53 | 3 | AHSG | a2-HS-Glycoprotein | P02765 | 186006350 | rs2518134 | 3 | 186332182 | C | T | 0.3292 | 0.0492 | 0.0172 | 4.19E-03 | -0.5178 | 0.0169 | 2.61E-206 | -0.095 | 0.033 | 0.909 | (0.852-0.971) | 4.36E-03 | 9.66E-03 | 1.57E-02 | 20 |
| SeqId_3054_3 | 16 | HP | HPT | P00738 | 72053141 | rs217184 | 16 | 72105965 | C | T | 0.2157 | 0.0598 | 0.0214 | 5.28E-03 | 0.6744 | 0.0204 | 2.84E-239 | 0.089 | 0.032 | 1.093 | (1.026-1.163) | 5.44E-03 | 1.32E-02 | 9.66E-02 | 20 |
| SeqId_17384_110 | 12 | PFKM | K6PF | P08237 | 48591352 | rs3742074 | 12 | 48457385 | T | C | 0.1616 | -0.0670 | 0.0220 | 2.36E-03 | 0.1431 | 0.0225 | 1.82E-10 | -0.468 | 0.171 | 0.626 | (0.448-0.875) | 6.06E-03 | 1.60E-02 | 9.80E-01 | 20 |
| SeqId_15583_18 | 1 | FCRLB | FCRLB | Q6BAA4 | 161811555 | rs61801180 | 1 | 161688764 | C | G | 0.0501 | 0.0946 | 0.0343 | 5.81E-03 | 0.6975 | 0.0366 | 3.88E-81 | 0.136 | 0.050 | 1.145 | (1.039-1.263) | 6.34E-03 | 1.73E-02 | 3.44E-02 | 20 |
| SeqId_18218_48 | 2 | CNRIP1 | CB032 | Q96F85 | 68333616 | rs7604489 | 2 | 68579911 | T | C | 0.1309 | -0.0627 | 0.0231 | 6.74E-03 | -0.8157 | 0.0221 | 1.09E-298 | 0.077 | 0.028 | 1.080 | (1.021-1.142) | 6.89E-03 | 1.76E-02 | 1.37E-03 | 20 |
| SeqId_16825_20 | 14 | ATXN3 | ATX3 | P54252 | 92353962 | rs1133441 | 14 | 92435065 | A | T | 0.3180 | -0.0465 | 0.0173 | 7.12E-03 | -0.5942 | 0.0170 | 9.14E-269 | 0.078 | 0.029 | 1.081 | (1.021-1.145) | 7.29E-03 | 1.86E-02 | 7.21E-02 | 20 |
| SeqId_19365_11 | 19 | BCAT2 | BCAT2 | O15382 | 49768463 | rs62125920 | 19 | 49306276 | G | A | 0.2168 | -0.0562 | 0.0209 | 7.10E-03 | -0.5821 | 0.0202 | 3.89E-182 | 0.097 | 0.036 | 1.101 | (1.026-1.182) | 7.36E-03 | 1.89E-02 | 1.26E-01 | 20 |
| SeqId_5060_62 | 9 | CD274 | B7-H1 | Q9NZQ7 | 5350828 | rs822342 | 9 | 5453973 | T | C | 0.2873 | 0.0501 | 0.0189 | 7.88E-03 | -0.3654 | 0.0189 | 3.28E-83 | -0.137 | 0.052 | 0.872 | (0.787-0.966) | 8.48E-03 | 1.89E-02 | 2.21E-01 | 20 |
| SeqId_6404_20 | 17 | C1QL1 | C1QRF | O75973 | 43413451 | rs7225162 | 17 | 43036260 | C | T | 0.5245 | -0.0433 | 0.0166 | 9.00E-03 | 0.3279 | 0.0163 | 1.64E-90 | -0.132 | 0.051 | 0.876 | (0.793-0.968) | 9.59E-03 | 2.12E-02 | 1.50E-01 | 20 |
| SeqId_5939_42 | 17 | TNFSF12 | TWEAK | O43508 | 7403792 | rs62059804 | 17 | 7453505 | A | C | 0.2536 | -0.0496 | 0.0187 | 8.00E-03 | 0.1809 | 0.0190 | 2.14E-21 | -0.274 | 0.107 | 0.760 | (0.616-0.938) | 1.06E-02 | 2.34E-02 | 5.20E-01 | 20 |
| SeqId_5069_9 | 1 | CD55 | DAF | P08174 | 207830383 | rs7526831 | 1 | 207438606 | C | G | 0.2454 | 0.0477 | 0.0189 | 1.15E-02 | -0.7963 | 0.0215 | 1.04E-300 | -0.060 | 0.024 | 0.942 | (0.899-0.987) | 1.17E-02 | 2.53E-02 | 3.00E-02 | 20 |
| SeqId_6947_4 | 3 | ST3GAL6 | SIA10 | Q9Y274 | 98342907 | rs9851650 | 3 | 98746055 | A | G | 0.0440 | 0.0952 | 0.0386 | 1.37E-02 | -1.3535 | 0.0360 | 1.10E-301 | -0.070 | 0.029 | 0.932 | (0.881-0.986) | 1.39E-02 | 2.72E-02 | 2.18E-01 | 20 |
| SeqId_4559_64 | 2 | KYNU | KYNU | Q16719 | 143747623 | rs73961713 | 2 | 143801205 | A | C | 0.1871 | -0.0534 | 0.0218 | 1.43E-02 | -0.8777 | 0.0237 | 1.04E-300 | 0.061 | 0.025 | 1.063 | (1.012-1.116) | 1.45E-02 | 3.16E-02 | 5.35E-01 | 20 |
| SeqId_6392_7 | 20 | WISP2 | WISP-2 | O76076 | 43715188 | rs6065746 | 20 | 43322002 | G | C | 0.4274 | -0.0412 | 0.0168 | 1.42E-02 | -0.3486 | 0.0166 | 3.82E-98 | 0.118 | 0.049 | 1.126 | (1.023-1.238) | 1.48E-02 | 3.23E-02 | 1.35E-01 | 20 |
| SeqId_19614_8 | 11 | TCN1 | Holo-TC I | P20061 | 60098597 | rs34324219 | 11 | 59623378 | A | C | 0.1155 | 0.1253 | 0.0509 | 1.38E-02 | -0.5714 | 0.0266 | 4.71E-102 | -0.219 | 0.090 | 0.803 | (0.674-0.957) | 1.44E-02 | 3.06E-02 | 2.37E-01 | 20 |
| SeqId_8479_4 | 11 | MMP10 | MMP-10 | P09238 | 102701021 | rs17860955 | 11 | 102649482 | C | T | 0.0184 | 0.1662 | 0.0688 | 1.57E-02 | -1.0766 | 0.0631 | 2.56E-65 | -0.154 | 0.065 | 0.857 | (0.755-0.973) | 1.68E-02 | 3.06E-02 | 2.50E-01 | 20 |
| SeqId_3516_60 | 10 | CXCL12 | SDF-1 | P48061 | 45371777 | rs1023264 | 10 | 44893756 | C | T | 0.3333 | 0.0431 | 0.0179 | 1.58E-02 | 0.1762 | 0.0179 | 8.26E-23 | 0.245 | 0.104 | 1.277 | (1.041-1.567) | 1.91E-02 | 3.50E-02 | 9.89E-02 | 20 |
| SeqId_3038_9 | 4 | CXCL11 | I-TAC | O14625 | 76939335 | rs6827617 | 4 | 76916146 | G | A | 0.4243 | -0.0395 | 0.0167 | 1.81E-02 | 0.1929 | 0.0170 | 8.32E-30 | -0.205 | 0.088 | 0.815 | (0.685-0.969) | 2.06E-02 | 3.90E-02 | 8.82E-01 | 20 |
| SeqId_2998_53 | 11 | JAM3 | JAM-C | Q9BX67 | 133901772 | rs655627 | 11 | 134021859 | G | A | 0.4417 | 0.0401 | 0.0171 | 1.92E-02 | -0.2157 | 0.0167 | 5.24E-38 | -0.186 | 0.081 | 0.830 | (0.709-0.973) | 2.12E-02 | 4.12E-02 | 9.01E-01 | 20 |
| SeqId_6364_7 | 12 | TAPBPL | TPSNR | Q9BX59 | 6601743 | rs2534724 | 12 | 6583387 | G | A | 0.3088 | -0.0423 | 0.0182 | 1.99E-02 | -0.6732 | 0.0179 | 1.10E-301 | 0.063 | 0.027 | 1.065 | (1.01-1.123) | 2.01E-02 | 3.87E-02 | 1.55E-01 | 20 |
| SeqId_5631_83 | 6 | MLN | MOTI | P12872 | 33834626 | rs9394169 | 6 | 33778964 | A | G | 0.4530 | -0.0381 | 0.0164 | 1.98E-02 | 0.3832 | 0.0163 | 5.55E-122 | -0.099 | 0.043 | 0.905 | (0.832-0.985) | 2.04E-02 | 3.85E-02 | 2.90E-02 | 20 |
| SeqId_3293_2 | 1 | CD5L | CD5L | O43866 | 157672304 | rs2765501 | 1 | 157804648 | A | G | 0.4018 | 0.0416 | 0.0180 | 2.12E-02 | 0.3770 | 0.0167 | 6.98E-113 | 0.110 | 0.048 | 1.117 | (1.016-1.227) | 2.19E-02 | 3.85E-02 | 1.34E-07 | 20 |
| SeqId_4771_10 | 6 | SMPDL3A | ASM3A | Q92484 | 122688494 | rs28385609 | 6 | 123122464 | T | C | 0.1391 | 0.0554 | 0.0237 | 1.95E-02 | -0.4827 | 0.0232 | 1.64E-96 | -0.115 | 0.049 | 0.892 | (0.809-0.982) | 2.03E-02 | 3.69E-02 | 2.52E-01 | 20 |
| SeqId_14091_42 | 21 | CBR3 | Carbonyl reductase 3 | O75828 | 37317418 | rs879894 | 21 | 37508643 | T | G | 0.3548 | 0.0383 | 0.0170 | 2.39E-02 | -0.8455 | 0.0228 | 1.04E-300 | -0.045 | 0.020 | 0.956 | (0.919-0.994) | 2.42E-02 | 3.69E-02 | 9.31E-01 | 20 |
| SeqId_16324_38 | 4 | TLR1 | TLR1 | Q15399 | 38924330 | rs5743604 | 4 | 38801285 | G | A | 0.2740 | 0.0452 | 0.0205 | 2.72E-02 | 0.2943 | 0.0194 | 5.94E-52 | 0.154 | 0.070 | 1.166 | (1.016-1.338) | 2.89E-02 | 4.31E-02 | 3.55E-01 | 20 |
| SeqId_3499_77 | 5 | IL17B | IL-17B | Q9UHF5 | 149074772 | rs6862301 | 5 | 148783690 | A | C | 0.3160 | -0.0406 | 0.0185 | 2.83E-02 | 0.1893 | 0.0182 | 2.04E-25 | -0.215 | 0.100 | 0.807 | (0.663-0.982) | 3.19E-02 | 5.06E-02 | 3.54E-01 | 20 |
| SeqId_9241_40 | 20 | SIRPG | SIRPG | Q9P1W8 | 1271886 | rs6043409 | 20 | 1616206 | A | G | 0.3384 | 0.0362 | 0.0173 | 3.60E-02 | -0.4930 | 0.0171 | 2.61E-183 | -0.074 | 0.035 | 0.929 | (0.867-0.995) | 3.65E-02 | 5.49E-02 | 6.38E-01 | 20 |

| | | | | | | | | | | | | | | | | | | | | | | | | |
|---|---|---|---|---|---|---|---|---|---|---|---|---|---|---|---|---|---|---|---|---|---|---|---|---|
| SeqId_15475_4 | 20 | PLTP | PLTP | P55058 | 44264887 | rs6065906 | 20 | 44554015 | C | T | 0.2055 | -0.0450 | 0.0215 | 3.63E-02 | -0.7378 | 0.0206 | 9.22E-281 | 0.061 | 0.029 | 1.063 | (1.004-1.125) | 3.66E-02 | 6.10E-02 | 2.34E-02 | 20 |
| SeqId_3352_80 | 1 | CA6 | Carbonic anhydrase 6 | P23280 | 8640831 | rs3765963 | 1 | 9034598 | G | A | 0.4039 | -0.0359 | 0.0177 | 4.21E-02 | 0.6486 | 0.0175 | 1.04E-300 | -0.055 | 0.027 | 0.946 | (0.897-0.998) | 4.24E-02 | 6.10E-02 | 9.80E-01 | 20 |
| SeqId_10391_1 | 1 | ANGPTL3 | ANGL3 | Q9Y5C1 | 63432716 | rs10889333 | 1 | 62957030 | A | G | 0.2996 | 0.0354 | 0.0175 | 4.33E-02 | -0.3361 | 0.0173 | 2.70E-84 | -0.105 | 0.052 | 0.900 | (0.812-0.997) | 4.44E-02 | 6.95E-02 | 7.54E-01 | 20 |
| SeqId_14079_14 | 2 | IL18R1 | IL-18 Ra | Q13478 | 102514224 | rs990171 | 2 | 103086770 | A | C | 0.2178 | -0.0386 | 0.0194 | 4.67E-02 | 0.8499 | 0.0229 | 1.04E-300 | -0.045 | 0.023 | 0.956 | (0.914-0.999) | 4.70E-02 | 7.16E-02 | 4.80E-01 | 20 |
| SeqId_2609_59 | 20 | CST3 | Cystatin C | P01034 | 23529910 | rs2405367 | 20 | 23622880 | A | G | 0.2249 | 0.0409 | 0.0207 | 4.76E-02 | -0.6126 | 0.0201 | 3.03E-204 | -0.067 | 0.034 | 0.935 | (0.875-0.999) | 4.81E-02 | 7.46E-02 | 7.79E-01 | 20 |
| SeqId_13983_27 | 1 | CRYZ | QOR | Q08257 | 75571679 | rs9804168 | 1 | 75249616 | T | C | 0.1258 | 0.0485 | 0.0250 | 5.26E-02 | 1.0264 | 0.0277 | 1.04E-300 | 0.047 | 0.024 | 1.048 | (0.999-1.1) | 5.29E-02 | 7.52E-02 | 6.87E-01 | 20 |
| SeqId_16616_137 | 17 | ENO3 | ENOB | P13929 | 5283252 | rs238238 | 17 | 4856376 | A | G | 0.3170 | -0.0339 | 0.0178 | 5.70E-02 | 0.3599 | 0.0180 | 2.33E-89 | -0.094 | 0.050 | 0.910 | (0.826-1.003) | 5.82E-02 | 8.14E-02 | 2.45E-01 | 20 |
| SeqId_8346_9 | 9 | DPP7 | DPP2 | Q9UHL4 | 140449582 | rs4880198 | 9 | 139979437 | T | C | 0.2301 | -0.0398 | 0.0210 | 5.81E-02 | -0.5236 | 0.0196 | 1.46E-157 | 0.076 | 0.040 | 1.079 | (0.997-1.167) | 5.87E-02 | 8.77E-02 | 7.32E-01 | 20 |
| SeqId_19277_4 | 1 | TSTD1 | KAT | Q8NFU3 | 160617028 | rs11580071 | 1 | 160951832 | A | G | 0.1329 | 0.0464 | 0.0247 | 6.10E-02 | 0.8957 | 0.0239 | 4.45E-301 | 0.052 | 0.028 | 1.053 | (0.998-1.112) | 6.13E-02 | 8.77E-02 | 5.86E-01 | 20 |
| SeqId_6462_12 | 3 | TIMP4 | TIMP-4 | Q99727 | 11811589 | rs184262 | 3 | 12134740 | A | G | 0.1513 | 0.0405 | 0.0216 | 6.09E-02 | -0.5602 | 0.0218 | 1.21E-145 | -0.072 | 0.039 | 0.930 | (0.862-1.004) | 6.15E-02 | 8.92E-02 | 1.49E-02 | 20 |
| SeqId_8480_29 | 2 | EFEMP1 | FBLN3 | Q12805 | 56498664 | rs3791679 | 2 | 56096892 | G | A | 0.2311 | -0.0353 | 0.0190 | 6.31E-02 | 0.4274 | 0.0193 | 3.78E-109 | -0.083 | 0.045 | 0.921 | (0.844-1.005) | 6.40E-02 | 8.92E-02 | 9.74E-01 | 20 |
| SeqId_16900_29 | 6 | MDGA1 | MDGA1 | Q8NFP4 | 37315967 | rs9394450 | 6 | 37648812 | A | C | 0.3538 | -0.0319 | 0.0175 | 6.78E-02 | 0.6270 | 0.0167 | 1.10E-301 | -0.051 | 0.028 | 0.950 | (0.9-1.004) | 6.81E-02 | 9.14E-02 | 6.89E-01 | 20 |
| SeqId_3449_58 | 14 | SERPINA4 | Kallistatin | P29622 | 94943634 | rs10140765 | 14 | 95023389 | T | C | 0.2536 | 0.0335 | 0.0186 | 7.22E-02 | 0.7169 | 0.0191 | 1.10E-301 | 0.047 | 0.026 | 1.048 | (0.996-1.103) | 7.25E-02 | 9.59E-02 | 4.73E-01 | 20 |
| SeqId_5353_89 | 2 | IL1RN | IL-1Ra | P18510 | 113785842 | rs55709272 | 2 | 113867288 | C | T | 0.4387 | 0.0290 | 0.0166 | 7.97E-02 | -0.3779 | 0.0166 | 8.23E-115 | -0.077 | 0.044 | 0.926 | (0.85-1.009) | 8.05E-02 | 1.01E-01 | 3.35E-01 | 20 |
| SeqId_12675_14 | 2 | DARS | SYDC | P14868 | 136553639 | rs2304371 | 2 | 136561557 | G | A | 0.2546 | 0.0502 | 0.0287 | 8.02E-02 | 0.4181 | 0.0214 | 1.06E-84 | 0.120 | 0.069 | 1.128 | (0.985-1.291) | 8.14E-02 | 1.10E-01 | 8.91E-02 | 20 |
| SeqId_3421_54 | 9 | TNFSF8 | CD30 Ligand | P32971 | 117908733 | rs3181348 | 9 | 117694184 | A | G | 0.4274 | 0.0282 | 0.0164 | 8.56E-02 | -0.2444 | 0.0167 | 1.57E-48 | -0.115 | 0.068 | 0.891 | (0.781-1.017) | 8.77E-02 | 1.10E-01 | 6.53E-01 | 20 |
| SeqId_5102_55 | 6 | MICB | MICB | Q29980 | 31132414 | rs3094011 | 6 | 31451836 | C | T | 0.0879 | 0.0413 | 0.0254 | 1.04E-01 | -0.8968 | 0.0249 | 2.64E-283 | -0.046 | 0.028 | 0.955 | (0.903-1.01) | 1.05E-01 | 1.17E-01 | 1.43E-13 | 20 |
| SeqId_9185_15 | 21 | TFF1 | TFF1 | P04155 | 43880975 | rs3761376 | 21 | 43787038 | A | G | 0.2352 | 0.0317 | 0.0202 | 1.16E-01 | -0.3651 | 0.0196 | 1.10E-77 | -0.087 | 0.055 | 0.917 | (0.822-1.022) | 1.17E-01 | 1.38E-01 | 6.03E-01 | 20 |
| SeqId_5128_53 | 1 | SLAMF6 | SLAF6 | Q96DU3 | 160617028 | rs1041067 | 1 | 160457127 | T | C | 0.1943 | 0.0322 | 0.0205 | 1.16E-01 | -0.3886 | 0.0206 | 1.45E-79 | -0.083 | 0.053 | 0.921 | (0.83-1.021) | 1.17E-01 | 1.51E-01 | 2.32E-03 | 20 |
| SeqId_15602_43 | 1 | IL6R | IL-6 sRa | P08887 | 154468135 | rs7549250 | 1 | 154404336 | C | T | 0.4571 | 0.0244 | 0.0165 | 1.39E-01 | -0.8594 | 0.0232 | 1.04E-300 | -0.028 | 0.019 | 0.972 | (0.936-1.009) | 1.39E-01 | 1.51E-01 | 6.60E-01 | 20 |
| SeqId_9416_77 | 12 | CPM | CBPM | P14384 | 69378261 | rs8181716 | 12 | 69418274 | T | G | 0.1892 | 0.0317 | 0.0219 | 1.48E-01 | -0.2219 | 0.0217 | 1.27E-24 | -0.143 | 0.100 | 0.867 | (0.713-1.054) | 1.52E-01 | 1.76E-01 | 5.89E-01 | 20 |
| SeqId_9018_38 | 4 | PCDH10 | PCD10 | Q9P2E7 | 134318904 | rs4864200 | 4 | 134467929 | T | G | 0.3517 | -0.0231 | 0.0170 | 1.75E-01 | 0.2359 | 0.0169 | 1.87E-44 | -0.098 | 0.072 | 0.907 | (0.787-1.045) | 1.77E-01 | 1.91E-01 | 6.98E-01 | 20 |
| SeqId_2849_49 | 6 | AIF1 | AIF1 | P55008 | 31132414 | rs3130623 | 6 | 31597700 | T | C | 0.1503 | -0.0259 | 0.0213 | 2.24E-01 | 0.2009 | 0.0214 | 5.49E-21 | -0.129 | 0.107 | 0.879 | (0.713-1.084) | 2.27E-01 | 2.18E-01 | 1.13E-11 | 20 |
| SeqId_19124_9 | 5 | UBLCP1 | UBCP1 | Q8WVY7 | 158327749 | rs13154564 | 5 | 158539430 | C | T | 0.0184 | 0.5384 | 0.4407 | 2.22E-01 | -0.3856 | 0.0610 | 2.66E-10 | -1.396 | 1.164 | 0.248 | (0.025-2.423) | 2.30E-01 | 2.77E-01 | 2.46E-01 | 20 |
| SeqId_14131_37 | 13 | EFNB2 | EFNB2 | P52799 | 107233192 | rs59166663 | 13 | 107299665 | C | A | 0.1339 | 0.0274 | 0.0230 | 2.34E-01 | 0.2788 | 0.0236 | 3.71E-32 | 0.098 | 0.083 | 1.103 | (0.938-1.298) | 2.36E-01 | 2.77E-01 | 3.61E-01 | 20 |
| SeqId_15635_4 | 6 | SMOC2 | SMOC2 | Q9H3U7 | 169167153 | rs56296467 | 6 | 168863807 | C | T | 0.2536 | -0.0217 | 0.0183 | 2.36E-01 | -0.4716 | 0.0183 | 9.25E-147 | 0.046 | 0.039 | 1.047 | (0.97-1.13) | 2.36E-01 | 2.78E-01 | 3.61E-01 | 20 |
| SeqId_19392_6 | 1 | DDAH1 | DDAH1 | O94760 | 85879524 | rs233071 | 1 | 85806005 | T | C | 0.3630 | -0.0195 | 0.0170 | 2.50E-01 | -0.1242 | 0.0173 | 7.18E-13 | 0.157 | 0.138 | 1.170 | (0.892-1.534) | 2.56E-01 | 2.78E-01 | 7.57E-04 | 20 |
| SeqId_5349_69 | 6 | DLL1 | DLL1 | O00548 | 170353402 | rs4710790 | 6 | 170588232 | C | A | 0.4448 | -0.0194 | 0.0182 | 2.85E-01 | 0.1754 | 0.0167 | 9.68E-26 | -0.111 | 0.104 | 0.895 | (0.73-1.098) | 2.87E-01 | 2.98E-01 | 3.00E-02 | 20 |
| SeqId_7813_6 | 2 | ALPP | Alkaline phosphatase-placental | P05187 | 232820171 | rs35458538 | 2 | 233304352 | A | G | 0.2280 | -0.0185 | 0.0205 | 3.65E-01 | 0.3932 | 0.0193 | 1.95E-92 | -0.047 | 0.052 | 0.954 | (0.861-1.057) | 3.66E-01 | 3.30E-01 | 9.94E-01 | 20 |
| SeqId_6715_63 | 2 | ALPPL2 | PPBN | P10696 | 232820171 | rs35458538 | 2 | 233304352 | A | G | 0.2280 | -0.0185 | 0.0205 | 3.65E-01 | 0.3251 | 0.0194 | 3.18E-63 | -0.057 | 0.063 | 0.945 | (0.835-1.069) | 3.66E-01 | 4.11E-01 | 9.98E-01 | 20 |
| SeqId_16288_17 | 2 | EPHA4 | EPHA4 | P54764 | 222135380 | rs16862777 | 2 | 222355473 | C | G | 0.2536 | 0.0164 | 0.0184 | 3.71E-01 | -0.2916 | 0.0185 | 8.95E-56 | -0.056 | 0.063 | 0.945 | (0.835-1.07) | 3.71E-01 | 4.11E-01 | 3.20E-01 | 20 |
| SeqId_6393_63 | 12 | HSP90B1 | Endoplasmin | P14625 | 104303246 | rs7973401 | 12 | 104252509 | A | G | 0.1626 | 0.0181 | 0.0225 | 4.21E-01 | 0.8374 | 0.0223 | 1.34E-301 | 0.022 | 0.027 | 1.022 | (0.969-1.077) | 4.21E-01 | 4.13E-01 | 8.20E-01 | 20 |
| SeqId_5742_14 | 1 | ACP6 | PPA6 | Q9NPH0 | 147166377 | rs10900387 | 1 | 147114303 | G | A | 0.4622 | -0.0128 | 0.0164 | 4.33E-01 | -0.6088 | 0.0162 | 1.10E-301 | 0.021 | 0.027 | 1.021 | (0.969-1.076) | 4.33E-01 | 4.62E-01 | 9.45E-01 | 20 |
| SeqId_2647_66 | 10 | GDI2 | Rab GDP dissociation inhibitor beta | P50395 | 5685209 | rs55913768 | 10 | 5749449 | A | G | 0.2955 | -0.0122 | 0.0173 | 4.79E-01 | -0.4715 | 0.0172 | 1.38E-165 | 0.026 | 0.037 | 1.026 | (0.955-1.103) | 4.79E-01 | 4.71E-01 | 5.40E-02 | 20 |
| SeqId_8960_3 | 5 | ERAP2 | LRAP | Q6P179 | 96533260 | rs7719293 | 5 | 96343766 | C | T | 0.0552 | 0.0149 | 0.0354 | 6.74E-01 | -1.2813 | 0.0341 | 1.10E-301 | -0.012 | 0.028 | 0.988 | (0.936-1.043) | 6.74E-01 | 5.15E-01 | 4.08E-01 | 20 |
| SeqId_6467_65 | 3 | POGLUT1 | KTEL1 | Q8NBL1 | 119113820 | rs17203139 | 3 | 119205217 | G | T | 0.0726 | 0.0066 | 0.0330 | 8.41E-01 | 1.1990 | 0.0336 | 5.87E-279 | 0.006 | 0.028 | 1.006 | (0.953-1.061) | 8.41E-01 | 7.17E-01 | 7.37E-04 | 20 |
| SeqId_10445_20 | 6 | APOM | ApoM | O95445 | 31132414 | rs805272 | 6 | 31641386 | T | G | 0.0317 | 0.0082 | 0.0489 | 8.66E-01 | -0.9520 | 0.0522 | 1.99E-74 | -0.009 | 0.051 | 0.991 | (0.896-1.096) | 8.66E-01 | 8.85E-01 | 4.65E-05 | 20 |
| SeqId_5698_60 | 6 | TNXB | Tenascin-X | P22105 | 32299822 | rs45451301 | 6 | 31935392 | C | T | 0.0245 | 0.0049 | 0.0939 | 9.58E-01 | -1.5551 | 0.0509 | 3.70E-205 | -0.003 | 0.060 | 0.997 | (0.886-1.122) | 9.58E-01 | 9.03E-01 | 3.86E-09 | 20 |
| SeqId_10761_5 | 12 | TMED2 | TMED2 | Q15363 | 124105839 | rs9919 | NA# | NA# | NA# | NA# | NA# | NA# | NA# | NA# | NA# | NA# | NA# | NA# | NA# | NA# | NA# | NA# | NA# | NA# | 20 |
| SeqId_15579_26 | 4 | ENPP6 | ENPP6 | Q6UWR7 | 185140823 | rs28673999 | NA# | NA# | NA# | NA# | NA# | NA# | NA# | NA# | NA# | NA# | NA# | NA# | NA# | NA# | NA# | NA# | NA# | NA# | 20 |
| SeqId_4141_79 | 4 | CXCL10 | IP-10 | P02778 | 77076287 | rs151000561 | NA* | NA* | NA* | NA* | NA* | NA* | NA* | NA* | NA* | NA* | NA* | NA* | NA* | NA* | NA* | NA* | NA* | NA* | 20 |

SMR: summary-data-based Mendelian randomization; PWAS: proteome-wide association study; pQTL: protein quantitative trait loci; SNP: single nucleotide polymorphism; A1: the effect allele; A2: the other allele; Freq: frequency of the effect allele; SE: standard error; OR: odds ratio; CI: confidence interval; HEIDI: heterogeneity in dependent instruments test; Benjamini-Hochberg method was used in FDR adjustment; * no signal in MS GWAS；#Pexposure>5e-8.

**Table S4. Colocalization analysis investigating whether the same causal variant is responsible for both plasma protein expression and multiple sclerosis**

| Gene | Protein | UniProt | $N_{snp}$ | PP.H0.abf | PP.H1.abf | PP.H2.abf | PP.H3.abf | PP.H4.abf |
|---|---|---|---|---|---|---|---|---|
| TNFRSF1A | TNF sR-I | P19438 | 2187 | 4.67E-34 | 3.46E-27 | 1.39E-10 | 2.88E-05 | 1.000 |
| STAT3 | STAT3 | P40763 | 1121 | 2.16E-146 | 2.61E-138 | 1.77E-10 | 2.03E-02 | 0.980 |
| FCRL3 | FCRL3 | Q96P31 | 2525 | 0.00E+00 | 0.00E+00 | 9.85E-06 | 5.86E-02 | 0.941 |
| ARHGAP1 | RHG01 | Q07960 | 849 | 3.23E-05 | 8.68E-05 | 2.33E-02 | 6.17E-02 | 0.915 |
| TYMP | TP | P19971 | 1862 | 2.79E-74 | 1.82E-70 | 1.95E-05 | 1.27E-01 | 0.873 |
| CR1 | Complement receptor type 1 | P17927 | 1748 | 2.32E-196 | 2.76E-196 | 7.61E-02 | 8.96E-02 | 0.834 |
| CD59 | CD59 | P13987 | 1763 | 3.07E-109 | 2.74E-110 | 1.69E-01 | 1.43E-02 | 0.817 |
| IDUA | IDUA | P35475 | 2211 | 0.00E+00 | 0.00E+00 | 1.55E-01 | 2.87E-02 | 0.816 |
| AHSG | a2-HS-Glycoprotein | P02765 | 2365 | 2.90E-212 | 3.96E-212 | 8.02E-02 | 1.09E-01 | 0.811 |
| PGLYRP1 | PGRP-S | O75594 | 2272 | 2.42E-28 | 2.79E-28 | 9.27E-02 | 1.06E-01 | 0.801 |
| WARS | SYWC | P23381 | 1795 | 7.01E-266 | 1.02E-265 | 9.36E-02 | 1.36E-01 | 0.770 |
| PLEK | PLEK | P08567 | 2397 | 6.26E-164 | 1.78E-159 | 8.97E-06 | 2.54E-01 | 0.746 |
| MMP13 | MMP-13 | P45452 | 3236 | 5.73E-09 | 1.72E-09 | 3.35E-01 | 1.00E-01 | 0.565 |
| ST3GAL6 | SIA10 | Q9Y274 | 2660 | 0.00E+00 | 0.00E+00 | 4.26E-01 | 8.82E-02 | 0.486 |
| PFKM | K6PF | P08237 | 2365 | 1.68E-06 | 7.08E-07 | 3.75E-01 | 1.57E-01 | 0.468 |
| ARFIP1 | ARFP1 | P53367 | 1662 | 8.11E-144 | 1.44E-144 | 4.63E-01 | 8.18E-02 | 0.456 |
| CCDC126 | CC126 | Q96EE4 | 2169 | 2.57E-157 | 4.03E-157 | 2.41E-01 | 3.79E-01 | 0.380 |
| CASP3 | Caspase-3 | P42574 | 2954 | 3.40E-195 | 1.89E-195 | 4.01E-01 | 2.22E-01 | 0.377 |
| UBASH3B | UBS3B | Q8TF42 | 2111 | 2.16E-78 | 5.76E-78 | 1.81E-01 | 4.83E-01 | 0.336 |
| HP | HPT | P00738 | 1617 | 1.73E-247 | 1.77E-248 | 6.24E-01 | 6.37E-02 | 0.312 |
| TCN1 | Holo-TC I | P20061 | 1697 | 3.29E-96 | 2.22E-97 | 6.70E-01 | 4.50E-02 | 0.285 |
| MAPK3 | ERK-1 | P27361 | 696 | 0.00E+00 | 0.00E+00 | 4.99E-03 | 7.33E-01 | 0.262 |
| FCRLB | FCRLB | Q6BAA4 | 2193 | 2.48E-73 | 3.11E-73 | 3.41E-01 | 4.28E-01 | 0.231 |
| TNFSF12 | TWEAK | O43508 | 1914 | 1.05E-15 | 6.63E-17 | 7.36E-01 | 4.62E-02 | 0.218 |
| ATXN3 | ATX3 | P54252 | 2591 | 4.39E-283 | 1.77E-283 | 5.85E-01 | 2.36E-01 | 0.179 |
| KYNU | KYNU | Q16719 | 1668 | 0.00E+00 | 0.00E+00 | 7.60E-01 | 6.57E-02 | 0.174 |
| CXCL11 | I-TAC | O14625 | 2551 | 1.30E-24 | 1.69E-25 | 7.65E-01 | 9.91E-02 | 0.136 |
| WISP2 | WISP-2 | O76076 | 1753 | 5.78E-94 | 5.66E-95 | 7.99E-01 | 7.82E-02 | 0.122 |
| SMPDL3A | ASM3A | Q92484 | 2234 | 3.08E-91 | 1.16E-91 | 6.45E-01 | 2.44E-01 | 0.111 |
| JAM3 | JAM-C | Q9BX67 | 2254 | 6.79E-32 | 4.75E-33 | 8.34E-01 | 5.81E-02 | 0.108 |
| CXCL12 | SDF-1 | P48061 | 2901 | 7.97E-17 | 1.81E-17 | 7.29E-01 | 1.65E-01 | 0.106 |
| CBR3 | Carbonyl reductase 3 | O75828 | 2489 | 0.00E+00 | 0.00E+00 | 8.52E-01 | 5.92E-02 | 0.089 |
| CD55 | DAF | P08174 | 1617 | 0.00E+00 | 0.00E+00 | 4.18E-01 | 4.95E-01 | 0.087 |
| IL17B | IL-17B | Q9UHF5 | 2206 | 2.82E-19 | 1.52E-20 | 8.67E-01 | 4.66E-02 | 0.087 |
| HSP90B1 | Endoplasmin | P14625 | 2598 | 0.00E+00 | 0.00E+00 | 7.91E-01 | 1.25E-01 | 0.084 |
| IL6R | IL-6 sRa | P08887 | 1632 | 0.00E+00 | 0.00E+00 | 8.83E-01 | 4.46E-02 | 0.073 |
| SERPINA4 | Kallistatin | P29622 | 3212 | 0.00E+00 | 0.00E+00 | 8.47E-01 | 8.20E-02 | 0.071 |
| SIRPG | SIRPG | Q9P1W8 | 2564 | 1.73E-185 | 2.17E-186 | 8.35E-01 | 1.05E-01 | 0.061 |
| ANGPTL3 | ANGL3 | Q9Y5C1 | 2168 | 3.52E-80 | 2.85E-81 | 8.71E-01 | 7.04E-02 | 0.059 |
| CST3 | Cystatin C | P01034 | 2869 | 5.13E-208 | 6.36E-209 | 8.39E-01 | 1.04E-01 | 0.057 |
| ERAP2 | LRAP | Q6P179 | 2594 | 0.00E+00 | 0.00E+00 | 8.58E-01 | 8.61E-02 | 0.056 |
| CD274 | B7-H1 | Q9NZQ7 | 2118 | 4.19E-79 | 1.66E-78 | 1.90E-01 | 7.54E-01 | 0.056 |
| TIMP4 | TIMP-4 | Q99727 | 2171 | 1.72E-143 | 2.82E-144 | 8.13E-01 | 1.33E-01 | 0.055 |
| DPP7 | DPP2 | Q9UHL4 | 996 | 1.51E-156 | 1.23E-157 | 8.75E-01 | 7.12E-02 | 0.054 |

| | | | | | | | | |
|---|---|---|---|---|---|---|---|---|
| CA6 | Carbonic anhydrase 6 | P23280 | 1968 | 0.00E+00 | 0.00E+00 | 7.80E-01 | 1.66E-01 | 0.053 |
| IL18R1 | IL-18 Ra | Q13478 | 2451 | 0.00E+00 | 0.00E+00 | 8.30E-01 | 1.20E-01 | 0.050 |
| CXCL10 | IP-10 | P02778 | 2520 | 1.90E-02 | 2.46E-03 | 8.23E-01 | 1.06E-01 | 0.049 |
| ENO3 | ENOB | P13929 | 2161 | 2.52E-84 | 2.01E-85 | 8.82E-01 | 7.03E-02 | 0.047 |
| EFEMP1 | FBLN3 | Q12805 | 2475 | 5.53E-105 | 4.32E-106 | 8.85E-01 | 6.91E-02 | 0.046 |
| MDGA1 | MDGA1 | Q8NFP4 | 2141 | 0.00E+00 | 0.00E+00 | 8.53E-01 | 1.09E-01 | 0.038 |
| DARS | SYDC | P14868 | 1346 | 2.24E-79 | 1.74E-79 | 5.43E-01 | 4.23E-01 | 0.035 |
| IL1RN | IL-1Ra | P18510 | 2002 | 3.01E-111 | 4.56E-112 | 8.41E-01 | 1.28E-01 | 0.032 |
| TNFSF8 | CD30 Ligand | P32971 | 2517 | 8.74E-43 | 1.13E-43 | 8.58E-01 | 1.11E-01 | 0.032 |
| TFF1 | TFF1 | P04155 | 3053 | 4.37E-72 | 6.26E-73 | 8.50E-01 | 1.22E-01 | 0.029 |
| CPM | CBPM | P14384 | 2013 | 1.09E-18 | 1.24E-19 | 8.74E-01 | 9.88E-02 | 0.027 |
| TSTD1 | KAT | Q8NFU3 | 2107 | 0.00E+00 | 0.00E+00 | 4.05E-02 | 9.35E-01 | 0.024 |
| ENPP6 | ENPP6 | Q6UWR7 | 2543 | 2.02E-02 | 1.77E-03 | 8.77E-01 | 7.68E-02 | 0.024 |
| SMOC2 | SMOC2 | Q9H3U7 | 3487 | 9.18E-146 | 2.24E-146 | 7.92E-01 | 1.93E-01 | 0.014 |
| ALPP | Alkaline phosphatase-placental | P05187 | 2112 | 1.34E-87 | 8.91E-89 | 9.24E-01 | 6.15E-02 | 0.014 |
| ALPPL2 | PPBN | P10696 | 2131 | 1.93E-57 | 1.31E-58 | 9.23E-01 | 6.26E-02 | 0.014 |
| MMP10 | MMP-10 | P09238 | 2470 | 6.72E-58 | 1.88E-58 | 7.72E-01 | 2.15E-01 | 0.013 |
| EFNB2 | EFNB2 | P52799 | 2149 | 3.97E-27 | 3.57E-27 | 5.20E-01 | 4.68E-01 | 0.012 |
| EPHA4 | EPHA4 | P54764 | 1951 | 3.07E-50 | 2.04E-51 | 9.26E-01 | 6.16E-02 | 0.012 |
| TLR1 | TLR1 | Q15399 | 2518 | 3.59E-01 | 3.16E-01 | 1.68E-01 | 1.48E-01 | 0.010 |
| ACP6 | PPA6 | Q9NPH0 | 1854 | 0.00E+00 | 0.00E+00 | 9.28E-01 | 6.34E-02 | 0.008 |
| CRYZ | QOR | Q08257 | 1888 | 5.43E-01 | 3.92E-01 | 3.34E-02 | 2.41E-02 | 0.008 |
| DLL1 | DLL1 | O00548 | 1791 | 1.29E-20 | 3.68E-20 | 2.58E-01 | 7.37E-01 | 0.004 |
| C1QL1 | C1QRF | O75973 | 1587 | 1.60E-87 | 8.75E-86 | 1.79E-02 | 9.79E-01 | 0.003 |
| PCDH10 | PCD10 | Q9P2E7 | 2340 | 8.38E-01 | 7.40E-02 | 7.82E-02 | 6.91E-03 | 0.003 |
| MICA | MICA | Q29983 | 2278 | 8.72E-01 | 5.18E-02 | 6.98E-02 | 4.14E-03 | 0.003 |
| NCR3 | NKp30 | O14931 | 2127 | 8.80E-01 | 4.85E-02 | 6.55E-02 | 3.60E-03 | 0.002 |
| AIF1 | AIF1 | P55008 | 1249 | 9.00E-01 | 5.47E-02 | 4.14E-02 | 2.52E-03 | 0.002 |
| FCRL1 | FCRL1 | Q96LA6 | 2493 | 1.28E-140 | 7.73E-137 | 1.65E-04 | 9.99E-01 | 0.001 |
| TMED2 | TMED2 | Q15363 | 1735 | 2.70E-05 | 6.27E-02 | 4.03E-04 | 9.36E-01 | 0.001 |
| SLAMF6 | SLAF6 | Q96DU3 | 2040 | 5.74E-76 | 2.69E-74 | 2.09E-02 | 9.78E-01 | 0.001 |
| BCAT2 | BCAT2 | O15382 | 1766 | 1.04E-186 | 8.53E-184 | 1.21E-03 | 9.98E-01 | 0.000 |
| FCRL4 | FCRL4 | Q96PJ5 | 2536 | 0.00E+00 | 0.00E+00 | 1.65E-04 | 1.00E+00 | 0.000 |
| UBLCP1 | UBCP1 | Q8WVY7 | 2047 | 1.22E-09 | 9.27E-04 | 1.31E-06 | 9.99E-01 | 0.000 |
| CD5L | CD5L | O43866 | 2429 | 6.78E-113 | 4.10E-109 | 1.65E-04 | 1.00E+00 | 0.000 |
| CNRIP1 | CB032 | Q96F85 | 2412 | 0.00E+00 | 0.00E+00 | 3.52E-05 | 1.00E+00 | 0.000 |
| IL2RA | IL-2 sRa | P01589 | 2917 | 2.16E-29 | 1.58E-09 | 1.37E-20 | 1.00E+00 | 0.000 |
| POGLUT1 | KTEL1 | Q8NBL1 | 2111 | 4.00E-290 | 1.13E-285 | 3.53E-05 | 1.00E+00 | 0.000 |
| TAPBP | TPSN | O15533 | 2045 | 0.00E+00 | 0.00E+00 | 1.35E-07 | 1.00E+00 | 0.000 |
| TAPBPL | TPSNR | Q9BX59 | 2045 | 0.00E+00 | 0.00E+00 | 1.35E-07 | 1.00E+00 | 0.000 |
| DDAH1 | DDAH1 | O94760 | 2292 | 3.27E-13 | 2.45E-08 | 1.33E-05 | 1.00E+00 | 0.000 |
| HSPA1A | HSP 70 | P0DMV8 | 4684 | 5.94E-305 | 4.95E-06 | 1.20E-299 | 1.00E+00 | 0.000 |
| MMP9 | MMP-9 | P14780 | 2256 | 9.62E-63 | 2.48E-55 | 3.87E-08 | 1.00E+00 | 0.000 |
| C2 | C2 | P06681 | 4018 | 0.00E+00 | 2.62E-49 | 1.20E-299 | 1.00E+00 | 0.000 |
| PLTP | PLTP | P55058 | 2328 | 7.71E-303 | 1.99E-295 | 3.87E-08 | 1.00E+00 | 0.000 |
| CD58 | sLFA-3 | P19256 | 1429 | 2.03E-28 | 2.47E-18 | 8.21E-11 | 1.00E+00 | 0.000 |
| MLN | MOTI | P12872 | 2685 | 9.99E-130 | 4.19E-119 | 2.38E-11 | 1.00E+00 | 0.000 |

| | | | | | | | | |
|---|---|---|---|---|---|---|---|---|
| GDI2 | Rab GDP dissociation inhibitor beta | P50395 | 3079 | 6.29E-186 | 4.58E-166 | 1.37E-20 | 1.00E+00 | 0.000 |
| ATF6B | ATF6B | Q99941 | 3979 | 0.00E+00 | 7.62E-45 | 1.18E-299 | 1.00E+00 | 0.000 |
| APOM | ApoM | O95445 | 5935 | 0.00E+00 | 2.88E-63 | 3.73E-290 | 1.00E+00 | 0.000 |
| AGER | sRAGE | Q15109 | 4266 | 0.00E+00 | 2.21E-72 | 1.18E-299 | 1.00E+00 | 0.000 |
| COL11A2 | COBA2 | P13942 | 5813 | 0 | 6.8166E-107 | 7.3693E-291 | 1 | 0.000 |
| HLA-DQA2 | DQA2 | P01906 | 7922 | 0.00E+00 | 7.07E-269 | 6.03E-298 | 1.00E+00 | 0.000 |
| TNXB | Tenascin-X | P22105 | 3707 | 0.00E+00 | 9.72E-192 | 1.18E-299 | 1.00E+00 | 0.000 |
| CFB | Factor B | P00751 | 4024 | 0.00E+00 | 2.03E-190 | 1.20E-299 | 1.00E+00 | 0.000 |
| MICB | MICB | Q29980 | 7172 | 0.00E+00 | 2.53E-296 | 3.71E-260 | 1.00E+00 | 0.000 |

pQTL: protein quantitative trait loci; PP.H0.abf: posterior probability of hypothesis 0 (neither trait of protein quantitative nor multiple sclerosis has a genetic association in the region); PP.H1.abf: posterior probability of hypothesis 1 ( only protein

**Table S5. SMR analysis identifying brain proteins causally associated with multiple sclerosis, based on candidate proteins determined by PWAS**

| ProbeID | Probe chromosome | Gene | Probe position | TopSNP | TopSNP_chromosome | TopSNP_position | A1 | A2 | Freq | Beta_GWAS | SE_GWAS | P_GWAS | Beta_pQTL | SE_pQTL | P_pQTL | Beta_SMR | SE_SMR | OR_SMR | 95%CI_SMR | P_SMR | FDR_adjusted_P_SMR | P_HEIDI | $N_{SNP}$_HEIDI |
|---|---|---|---|---|---|---|---|---|---|---|---|---|---|---|---|---|---|---|---|---|---|---|---|
| ENSG00000234745 | 6 | HLA-B | 31327660 | rs2523589 | 6 | 31327334 | T | G | 0.562 | -0.278 | 0.017 | 4.52E-62 | -0.156 | 0.026 | 3.65E-09 | 1.782 | 0.320 | 5.939 | (5.92-5.96) | 2.70E-08 | 2.30E-06 | 6.84E-02 | 9 |
| ENSG00000123297 | 12 | TSFM | 58104104 | rs11172335 | 12 | 58175201 | T | C | 0.317 | -0.113 | 0.018 | 1.57E-10 | -0.092 | 0.009 | 1.31E-24 | 1.238 | 0.228 | 3.447 | (3.44-3.46) | 5.75E-08 | 2.30E-06 | 1.25E-01 | 20 |
| ENSG00000213722 | 6 | DDAH2 | 31710020 | rs1144708 | 6 | 31710020 | T | C | 0.366 | -0.108 | 0.017 | 4.10E-10 | -0.084 | 0.009 | 1.04E-22 | 1.282 | 0.243 | 3.602 | (3.59-3.61) | 1.36E-07 | 2.44E-06 | 1.85E-06 | 8 |
| ENSG00000054983 | 14 | GALC | 88211507 | rs366615 | 14 | 88411837 | A | G | 0.489 | -0.087 | 0.016 | 7.20E-08 | -0.242 | 0.016 | 4.33E-51 | 0.361 | 0.071 | 1.435 | (1.43-1.44) | 3.96E-07 | 3.85E-06 | 2.69E-05 | 20 |
| ENSG00000104427 | 8 | ZC2HC1A | 79586852 | rs1545228 | 8 | 79644437 | G | A | 0.284 | 0.102 | 0.018 | 1.53E-08 | 0.037 | 0.006 | 4.74E-10 | 2.742 | 0.655 | 15.514 | (15.41-15.62) | 2.82E-05 | 8.42E-06 | 7.45E-01 | 10 |
| ENSG00000176974 | 17 | SHMT1 | 18221010 | rs2461838 | 17 | 18265264 | A | G | 0.291 | 0.096 | 0.022 | 1.20E-05 | 0.172 | 0.013 | 5.69E-42 | 0.558 | 0.134 | 1.748 | (1.75-1.75) | 3.10E-05 | 4.39E-04 | 3.30E-02 | 10 |
| ENSG00000150967 | 12 | ABCB9 | 123377821 | rs7305511 | 12 | 123498253 | G | T | 0.311 | 0.095 | 0.018 | 9.73E-08 | 0.067 | 0.011 | 3.60E-10 | 1.421 | 0.350 | 4.143 | (4.13-4.16) | 4.87E-05 | 4.39E-04 | 1.28E-03 | 4 |
| ENSG00000131323 | 14 | TRAF3 | 103404159 | rs3803286 | 14 | 103246470 | A | G | 0.319 | 0.095 | 0.017 | 2.93E-08 | 0.034 | 0.006 | 2.29E-08 | 2.796 | 0.710 | 16.373 | (16.25-16.49) | 8.27E-05 | 5.91E-04 | 1.76E-01 | 10 |
| ENSG00000163389 | 3 | POGLUT1 | 119113820 | rs2282171 | 3 | 119182598 | T | C | 0.395 | -0.081 | 0.017 | 1.00E-06 | -0.051 | 0.009 | 1.55E-08 | 1.587 | 0.429 | 4.889 | (4.87-4.91) | 2.16E-04 | 8.79E-04 | 1.78E-02 | 12 |
| ENSG00000163596 | 2 | ICA1L | 203700897 | rs1541853 | 2 | 203835177 | C | A | 0.129 | -0.098 | 0.025 | 7.35E-05 | 0.077 | 0.009 | 9.04E-18 | -1.271 | 0.353 | 0.281 | (0.28-0.28) | 3.19E-04 | 2.04E-03 | 6.64E-01 | 5 |
| ENSG00000177000 | 1 | MTHFR | 11928830 | rs1801133 | 1 | 11856378 | A | G | 0.364 | -0.079 | 0.022 | 2.72E-04 | -0.130 | 0.012 | 4.15E-28 | 0.608 | 0.176 | 1.838 | (1.83-1.84) | 5.48E-04 | 2.71E-03 | 5.62E-01 | 10 |
| ENSG00000149927 | 16 | DOC2A | 29927702 | rs11642612 | 16 | 30030195 | C | A | 0.383 | 0.068 | 0.017 | 5.20E-05 | 0.042 | 0.007 | 4.19E-10 | 1.628 | 0.479 | 5.093 | (5.07-5.12) | 6.83E-04 | 4.24E-03 | 1.22E-01 | 8 |
| ENSG00000117305 | 1 | HMGCL | 24133612 | rs11591202 | 1 | 24102080 | C | A | 0.328 | 0.064 | 0.017 | 2.04E-04 | 0.050 | 0.007 | 1.16E-13 | 1.272 | 0.383 | 3.567 | (3.55-3.58) | 8.97E-04 | 4.84E-03 | 2.78E-01 | 7 |
| ENSG00000146729 | 7 | GBAS | 56064262 | rs7793921 | 7 | 56120881 | G | A | 0.350 | -0.065 | 0.018 | 2.63E-04 | 0.051 | 0.006 | 1.75E-15 | -1.265 | 0.381 | 0.282 | (0.28-0.28) | 9.09E-04 | 5.52E-03 | 1.46E-03 | 11 |
| ENSG00000112584 | 6 | FAM120B | 170716259 | rs9366216 | 6 | 170676326 | A | C | 0.123 | -0.094 | 0.024 | 8.53E-05 | -0.071 | 0.012 | 2.20E-09 | 1.317 | 0.401 | 3.734 | (3.72-3.75) | 1.02E-03 | 5.52E-03 | 4.94E-01 | 14 |
| ENSG00000110514 | 11 | MADD | 47294626 | rs11570115 | 11 | 47354905 | C | T | 0.100 | -0.131 | 0.038 | 5.07E-04 | -0.085 | 0.010 | 6.72E-18 | 1.549 | 0.480 | 4.707 | (4.68-4.73) | 1.26E-03 | 5.80E-03 | 6.65E-01 | 8 |
| ENSG00000133574 | 7 | GIMAP4 | 150313416 | rs6951484 | 7 | 150262436 | C | T | 0.380 | 0.056 | 0.017 | 6.68E-04 | 0.101 | 0.011 | 2.39E-19 | 0.559 | 0.176 | 1.749 | (1.75-1.75) | 1.46E-03 | 6.71E-03 | 5.97E-01 | 14 |
| ENSG00000148090 | 9 | AUH | 93936592 | rs4743820 | 9 | 93928416 | C | T | 0.284 | -0.063 | 0.018 | 5.97E-04 | 0.055 | 0.007 | 1.30E-16 | -1.139 | 0.359 | 0.320 | (0.32-0.32) | 1.52E-03 | 7.18E-03 | 8.69E-01 | 20 |
| ENSG00000103485 | 16 | QPRT | 29652488 | rs9933310 | 16 | 29690904 | G | A | 0.392 | -0.056 | 0.017 | 1.29E-03 | -0.183 | 0.014 | 1.97E-39 | 0.304 | 0.097 | 1.356 | (1.35-1.36) | 1.78E-03 | 7.18E-03 | 1.02E-01 | 9 |
| ENSG00000214309 | 7 | MBLAC1 | 99746201 | rs4134927 | 7 | 99705320 | C | A | 0.070 | 0.121 | 0.039 | 1.88E-03 | -0.315 | 0.021 | 3.21E-50 | -0.386 | 0.127 | 0.680 | (0.68-0.68) | 2.35E-03 | 7.95E-03 | 6.97E-01 | 4 |
| ENSG00000023191 | 11 | RNH1 | 494662 | rs7121599 | 11 | 464667 | C | T | 0.304 | -0.054 | 0.018 | 2.92E-03 | -0.099 | 0.009 | 1.73E-28 | 0.546 | 0.190 | 1.727 | (1.72-1.73) | 4.05E-03 | 9.97E-03 | 3.53E-01 | 20 |
| ENSG00000163818 | 3 | LZTFL1 | 45780780 | rs1129183 | 3 | 45869972 | T | C | 0.075 | 0.091 | 0.030 | 2.13E-03 | -0.090 | 0.012 | 2.38E-14 | -1.012 | 0.355 | 0.364 | (0.36-0.36) | 4.39E-03 | 1.64E-02 | 3.44E-01 | 4 |
| ENSG00000125779 | 20 | PANK2 | 3784593 | rs16989000 | 20 | 3848416 | C | A | 0.405 | 0.050 | 0.017 | 2.88E-03 | 0.043 | 0.005 | 2.28E-17 | 1.159 | 0.412 | 3.187 | (3.17-3.20) | 4.92E-03 | 1.70E-02 | 5.45E-01 | 19 |
| ENSG00000153904 | 1 | DDAH1 | 85939476 | rs648310 | 1 | 85915134 | C | T | 0.424 | 0.052 | 0.016 | 1.38E-03 | -0.025 | 0.004 | 1.24E-08 | -2.116 | 0.758 | 0.121 | (0.12-0.12) | 5.28E-03 | 1.82E-02 | 1.30E-02 | 20 |
| ENSG00000097021 | 1 | ACOT7 | 6542330 | rs3789498 | 1 | 6358604 | C | T | 0.137 | 0.070 | 0.024 | 2.84E-03 | -0.058 | 0.008 | 1.64E-12 | -1.215 | 0.442 | 0.297 | (0.30-0.30) | 5.97E-03 | 1.87E-02 | 4.06E-01 | 20 |
| ENSG00000213689 | 3 | TREX1 | 48449956 | rs11797 | 3 | 48508585 | T | C | 0.442 | 0.049 | 0.017 | 3.56E-03 | -0.107 | 0.014 | 1.33E-14 | -0.460 | 0.169 | 0.631 | (0.63-0.63) | 6.40E-03 | 2.03E-02 | 6.21E-01 | 10 |
| ENSG00000136810 | 9 | TXN | 112928492 | rs1049927 | 9 | 113018755 | C | T | 0.122 | -0.069 | 0.024 | 4.61E-03 | -0.085 | 0.009 | 1.01E-22 | 0.811 | 0.298 | 2.251 | (2.24-2.26) | 6.49E-03 | 2.04E-02 | 6.47E-01 | 4 |
| ENSG00000141569 | 17 | TRIM65 | 73785718 | rs1551619 | 17 | 73885805 | T | C | 0.247 | 0.056 | 0.020 | 3.82E-03 | 0.137 | 0.019 | 3.95E-13 | 0.411 | 0.153 | 1.508 | (1.51-1.51) | 7.21E-03 | 2.04E-02 | 2.32E-01 | 10 |
| ENSG00000086205 | 11 | FOLH1 | 49135301 | rs10839239 | 11 | 49243246 | G | A | 0.275 | 0.052 | 0.018 | 4.24E-03 | 0.148 | 0.023 | 3.03E-10 | 0.349 | 0.134 | 1.418 | (1.42-1.42) | 9.21E-03 | 2.19E-02 | 2.68E-01 | 8 |
| ENSG00000163933 | 3 | RFT1 | 53140603 | rs2581786 | 3 | 53126143 | G | A | 0.402 | -0.044 | 0.016 | 7.30E-03 | 0.097 | 0.010 | 3.91E-22 | -0.450 | 0.174 | 0.638 | (0.64-0.64) | 9.73E-03 | 2.70E-02 | 2.93E-01 | 11 |
| ENSG00000167434 | 17 | CA4 | 58140953 | rs345189 | 17 | 58230148 | C | T | 0.061 | 0.106 | 0.039 | 6.25E-03 | -0.274 | 0.036 | 1.96E-14 | -0.386 | 0.150 | 0.680 | (0.68-0.68) | 1.00E-02 | 2.75E-02 | 5.55E-01 | 12 |
| ENSG00000113391 | 5 | FAM172A | 93074804 | rs9314093 | 5 | 93333347 | A | G | 0.139 | 0.067 | 0.025 | 6.60E-03 | 0.061 | 0.009 | 6.73E-11 | 1.087 | 0.434 | 2.966 | (2.95-2.98) | 1.22E-02 | 2.75E-02 | 8.43E-01 | 18 |
| ENSG00000205808 | 9 | PPAPDC2 | 4734014 | rs445684 | 9 | 4670315 | A | G | 0.091 | -0.104 | 0.039 | 7.38E-03 | -0.118 | 0.018 | 7.54E-11 | 0.883 | 0.356 | 2.418 | (2.41-2.43) | 1.32E-02 | 3.23E-02 | 9.27E-02 | 3 |
| ENSG00000126602 | 16 | TRAP1 | 3620927 | rs2791 | 16 | 3708170 | T | C | 0.039 | -0.123 | 0.048 | 1.04E-02 | -0.230 | 0.025 | 6.97E-20 | 0.534 | 0.216 | 1.706 | (1.70-1.71) | 1.36E-02 | 3.41E-02 | 5.95E-01 | 13 |
| ENSG00000157881 | 1 | PANK4 | 2376733 | rs10910082 | 1 | 2418935 | C | T | 0.387 | -0.047 | 0.018 | 8.55E-03 | 0.031 | 0.005 | 3.11E-10 | -1.508 | 0.622 | 0.221 | (0.22-0.22) | 1.53E-02 | 3.41E-02 | 4.29E-02 | 14 |
| ENSG00000110880 | 12 | CORO1C | 108949739 | rs1861642 | 12 | 109032489 | T | C | 0.459 | 0.048 | 0.019 | 1.14E-02 | -0.041 | 0.005 | 7.50E-17 | -1.161 | 0.479 | 0.313 | (0.31-0.31) | 1.55E-02 | 3.65E-02 | 5.43E-01 | 12 |
| ENSG00000152778 | 10 | IFIT5 | 91103420 | rs303218 | 10 | 91152593 | A | G | 0.211 | 0.051 | 0.021 | 1.37E-02 | -0.079 | 0.008 | 1.80E-21 | -0.641 | 0.268 | 0.527 | (0.53-0.53) | 1.70E-02 | 3.65E-02 | 6.34E-01 | 12 |
| ENSG00000170266 | 3 | GLB1 | 33102464 | rs7637099 | 3 | 33138549 | G | A | 0.357 | -0.043 | 0.017 | 1.14E-02 | -0.055 | 0.008 | 4.73E-12 | 0.779 | 0.328 | 2.179 | (2.17-2.19) | 1.75E-02 | 3.91E-02 | 3.72E-02 | 20 |
| ENSG00000136319 | 14 | TTC5 | 20639015 | rs2318864 | 14 | 20767618 | G | A | 0.259 | -0.046 | 0.019 | 1.33E-02 | -0.060 | 0.007 | 3.38E-17 | 0.767 | 0.323 | 2.153 | (2.15-2.16) | 1.76E-02 | 3.83E-02 | 7.21E-01 | 20 |
| ENSG00000164308 | 5 | ERAP2 | 96320495 | rs7716222 | 5 | 96293006 | A | C | 0.413 | -0.039 | 0.016 | 1.67E-02 | 0.445 | 0.038 | 2.53E-32 | -0.088 | 0.038 | 0.915 | (0.92-0.92) | 1.90E-02 | 3.83E-02 | 8.15E-01 | 20 |
| ENSG00000088280 | 1 | ASAP3 | 23884661 | rs1555024 | 1 | 23786203 | C | T | 0.308 | -0.045 | 0.018 | 1.16E-02 | -0.056 | 0.010 | 1.13E-08 | 0.803 | 0.348 | 2.233 | (2.22-2.24) | 2.10E-02 | 4.04E-02 | 3.41E-01 | 7 |

| | | | | | | | | | | | | | | | | | | | | | | | |
|---|---|---|---|---|---|---|---|---|---|---|---|---|---|---|---|---|---|---|---|---|---|---|---|
| ENSG00000170017 | 3 | ALCAM | 105238615 | rs11710166 | 3 | 105168098 | A | C | 0.156 | 0.055 | 0.022 | 1.33E-02 | -0.055 | 0.009 | 2.37E-10 | -1.000 | 0.434 | 0.368 | (0.37-0.37) | 2.11E-02 | 4.27E-02 | 5.87E-01 | 15 |
| ENSG00000183605 | 10 | SFXN4 | 120907828 | rs10749291 | 10 | 120920588 | C | T | 0.436 | -0.039 | 0.017 | 1.89E-02 | -0.077 | 0.007 | 1.06E-25 | 0.508 | 0.222 | 1.662 | (1.66-1.67) | 2.19E-02 | 4.27E-02 | 8.22E-01 | 17 |
| ENSG00000140474 | 15 | ULK3 | 75137252 | rs936227 | 15 | 75131959 | A | G | 0.420 | 0.038 | 0.017 | 2.09E-02 | 0.107 | 0.007 | 3.37E-49 | 0.358 | 0.157 | 1.431 | (1.43-1.43) | 2.25E-02 | 4.34E-02 | 2.39E-01 | 16 |
| ENSG00000197043 | 5 | ANXA6 | 150458146 | rs6859236 | 5 | 150535644 | A | G | 0.510 | -0.040 | 0.016 | 1.60E-02 | 0.045 | 0.007 | 3.57E-11 | -0.880 | 0.389 | 0.415 | (0.41-0.42) | 2.36E-02 | 4.34E-02 | 7.32E-01 | 10 |
| ENSG00000140105 | 14 | WARS | 100820980 | rs12897338 | 14 | 100844368 | C | T | 0.522 | -0.039 | 0.016 | 1.85E-02 | -0.061 | 0.009 | 3.71E-12 | 0.630 | 0.282 | 1.877 | (1.87-1.88) | 2.57E-02 | 4.46E-02 | 1.46E-01 | 19 |
| ENSG00000001561 | 6 | ENPP4 | 46128745 | rs7754915 | 6 | 46097569 | G | A | 0.323 | 0.041 | 0.017 | 1.75E-02 | 0.057 | 0.009 | 1.83E-10 | 0.715 | 0.321 | 2.044 | (2.04-2.05) | 2.60E-02 | 4.71E-02 | 4.37E-01 | 13 |
| ENSG00000159231 | 21 | CBR3 | 37443658 | rs1056892 | 21 | 37518706 | A | G | 0.354 | 0.038 | 0.017 | 2.62E-02 | -0.188 | 0.010 | 1.18E-74 | -0.200 | 0.091 | 0.818 | (0.82-0.82) | 2.73E-02 | 4.71E-02 | 9.16E-01 | 20 |
| ENSG00000088682 | 16 | COQ9 | 57575397 | rs223877 | 16 | 57509164 | A | G | 0.254 | 0.043 | 0.019 | 2.14E-02 | 0.053 | 0.008 | 3.82E-10 | 0.806 | 0.373 | 2.239 | (2.23-2.25) | 3.08E-02 | 5.00E-02 | 9.71E-01 | 18 |
| ENSG00000203705 | 1 | TATDN3 | 212951364 | rs12753524 | 1 | 212887790 | A | C | 0.319 | 0.040 | 0.018 | 2.24E-02 | 0.055 | 0.009 | 1.17E-09 | 0.726 | 0.340 | 2.068 | (2.06-2.08) | 3.26E-02 | 5.33E-02 | 3.25E-02 | 14 |
| ENSG00000183208 | 15 | GDPGP1 | 90825433 | rs12904427 | 15 | 90789670 | T | C | 0.181 | 0.046 | 0.022 | 3.43E-02 | -0.258 | 0.019 | 9.07E-43 | -0.179 | 0.086 | 0.836 | (0.84-0.84) | 3.65E-02 | 5.54E-02 | 6.18E-02 | 12 |
| ENSG00000170448 | 4 | NFXL1 | 47958679 | rs1822030 | 4 | 47907524 | A | G | 0.332 | -0.038 | 0.017 | 2.90E-02 | -0.054 | 0.008 | 1.78E-11 | 0.700 | 0.337 | 2.015 | (2.01-2.02) | 3.78E-02 | 6.08E-02 | 2.97E-01 | 8 |
| ENSG00000133835 | 5 | HSD17B4 | 118859546 | rs2678070 | 5 | 118812180 | G | A | 0.484 | -0.034 | 0.016 | 3.41E-02 | -0.060 | 0.006 | 2.69E-24 | 0.570 | 0.275 | 1.769 | (1.76-1.77) | 3.80E-02 | 6.10E-02 | 3.77E-03 | 20 |
| ENSG00000133606 | 7 | MKRN1 | 140129626 | rs12155423 | 7 | 140145753 | T | C | 0.310 | -0.041 | 0.019 | 3.03E-02 | -0.058 | 0.009 | 9.32E-12 | 0.698 | 0.338 | 2.010 | (2.00-2.02) | 3.90E-02 | 6.10E-02 | 7.02E-01 | 7 |
| ENSG00000178802 | 15 | MPI | 75137252 | rs1130741 | 15 | 75189930 | A | G | 0.491 | 0.034 | 0.016 | 3.69E-02 | -0.083 | 0.007 | 2.01E-29 | -0.411 | 0.200 | 0.663 | (0.66-0.66) | 4.02E-02 | 6.14E-02 | 2.13E-01 | 17 |
| ENSG00000008283 | 17 | CYB561 | 61459067 | rs4968765 | 17 | 61432774 | A | G | 0.509 | -0.035 | 0.016 | 3.23E-02 | 0.095 | 0.013 | 1.00E-12 | -0.366 | 0.179 | 0.693 | (0.69-0.69) | 4.03E-02 | 6.12E-02 | 3.44E-01 | 7 |
| ENSG00000159131 | 21 | GART | 34821570 | rs2834234 | 21 | 34894623 | T | C | 0.222 | -0.040 | 0.019 | 3.98E-02 | -0.173 | 0.011 | 2.11E-54 | 0.230 | 0.113 | 1.259 | (1.26-1.26) | 4.16E-02 | 6.12E-02 | 3.60E-01 | 14 |
| ENSG00000162728 | 1 | KCNJ9 | 160086968 | rs11265318 | 1 | 160060272 | A | G | 0.371 | 0.035 | 0.017 | 4.34E-02 | -0.134 | 0.015 | 3.33E-19 | -0.260 | 0.132 | 0.771 | (0.77-0.77) | 4.88E-02 | 6.20E-02 | 1.49E-01 | 19 |
| ENSG00000108064 | 10 | TFAM | 60230128 | rs9971104 | 10 | 60142800 | T | G | 0.180 | 0.044 | 0.021 | 3.90E-02 | -0.053 | 0.008 | 1.09E-10 | -0.832 | 0.424 | 0.435 | (0.43-0.44) | 4.93E-02 | 7.11E-02 | 3.94E-01 | 6 |
| ENSG00000137133 | 9 | HINT2 | 35833448 | rs4879929 | 9 | 35815356 | C | T | 0.116 | -0.055 | 0.027 | 4.60E-02 | -0.060 | 0.010 | 1.03E-09 | 0.909 | 0.479 | 2.482 | (2.47-2.49) | 5.78E-02 | 7.11E-02 | 4.26E-02 | 7 |
| ENSG00000162999 | 2 | DUSP19 | 183957860 | rs3748881 | 2 | 184023151 | A | G | 0.054 | 0.073 | 0.037 | 4.91E-02 | -0.210 | 0.032 | 3.66E-11 | -0.349 | 0.185 | 0.706 | (0.70-0.71) | 5.93E-02 | 8.19E-02 | 7.35E-01 | 4 |
| ENSG00000155792 | 8 | DEPTOR | 120924030 | rs4871827 | 8 | 121061879 | A | G | 0.337 | -0.034 | 0.017 | 4.94E-02 | 0.086 | 0.013 | 2.91E-11 | -0.394 | 0.209 | 0.674 | (0.67-0.68) | 5.94E-02 | 8.15E-02 | 2.51E-01 | 20 |
| ENSG00000159784 | 7 | FAM131B | 143149253 | rs4236482 | 7 | 143054309 | A | G | 0.212 | -0.041 | 0.021 | 4.95E-02 | -0.049 | 0.007 | 2.20E-11 | 0.827 | 0.439 | 2.287 | (2.28-2.30) | 5.95E-02 | 8.03E-02 | 9.25E-01 | 3 |
| ENSG00000240771 | 12 | ARHGEF25 | 58104104 | rs1806652 | 12 | 57999101 | T | C | 0.239 | 0.039 | 0.020 | 4.83E-02 | 0.057 | 0.009 | 4.47E-10 | 0.676 | 0.359 | 1.966 | (1.96-1.97) | 5.98E-02 | 7.94E-02 | 2.61E-02 | 5 |
| ENSG00000106665 | 7 | CLIP2 | 73800711 | rs10949838 | 7 | 73810096 | A | G | 0.496 | -0.032 | 0.017 | 5.42E-02 | -0.024 | 0.004 | 2.19E-09 | 1.328 | 0.725 | 3.773 | (3.75-3.80) | 6.69E-02 | 7.94E-02 | 6.04E-01 | 3 |
| ENSG00000146085 | 6 | MUT | 49523873 | rs2501979 | 6 | 49376924 | C | T | 0.339 | -0.030 | 0.017 | 7.34E-02 | -0.088 | 0.012 | 3.71E-13 | 0.344 | 0.198 | 1.411 | (1.41-1.41) | 8.21E-02 | 8.74E-02 | 8.19E-01 | 12 |
| ENSG00000079691 | 6 | LRRC16A | 25659172 | rs1543603 | 6 | 25413922 | A | G | 0.242 | 0.034 | 0.020 | 7.94E-02 | 0.037 | 0.006 | 2.92E-09 | 0.925 | 0.550 | 2.521 | (2.51-2.54) | 9.25E-02 | 1.06E-01 | 5.91E-01 | 12 |
| ENSG00000164933 | 8 | SLC25A32 | 104373506 | rs3134269 | 8 | 104421902 | T | C | 0.061 | 0.053 | 0.030 | 8.28E-02 | -0.090 | 0.016 | 3.42E-08 | -0.587 | 0.354 | 0.556 | (0.55-0.56) | 9.79E-02 | 1.17E-01 | 7.45E-01 | 5 |
| ENSG00000115840 | 2 | SLC25A12 | 172820315 | rs7608403 | 2 | 172646765 | C | A | 0.281 | 0.031 | 0.018 | 9.11E-02 | 0.049 | 0.007 | 2.41E-11 | 0.627 | 0.383 | 1.872 | (1.86-1.88) | 1.01E-01 | 1.22E-01 | 3.92E-02 | 16 |
| ENSG00000143156 | 1 | NME7 | 169279707 | rs10442644 | 1 | 169257750 | A | C | 0.344 | -0.029 | 0.017 | 9.00E-02 | -0.050 | 0.008 | 1.22E-09 | 0.591 | 0.362 | 1.806 | (1.80-1.81) | 1.02E-01 | 1.24E-01 | 4.42E-01 | 20 |
| ENSG00000149089 | 11 | APIP | 35010469 | rs1571134 | 11 | 34910077 | G | A | 0.314 | 0.028 | 0.017 | 1.05E-01 | 0.356 | 0.024 | 1.98E-51 | 0.079 | 0.049 | 1.083 | (1.08-1.08) | 1.07E-01 | 1.24E-01 | 6.71E-01 | 20 |
| ENSG00000110801 | 12 | PSMD9 | 122427338 | rs1169081 | 12 | 122405912 | T | G | 0.310 | -0.028 | 0.018 | 1.16E-01 | -0.044 | 0.006 | 7.50E-15 | 0.637 | 0.414 | 1.890 | (1.88-1.90) | 1.24E-01 | 1.28E-01 | 1.13E-01 | 10 |
| ENSG00000196839 | 20 | ADA | 43337933 | rs2007720 | 20 | 43276688 | G | A | 0.242 | -0.031 | 0.020 | 1.26E-01 | 0.123 | 0.014 | 1.97E-19 | -0.251 | 0.166 | 0.778 | (0.78-0.78) | 1.31E-01 | 1.46E-01 | 3.95E-01 | 12 |
| ENSG00000161618 | 19 | ALDH16A1 | 49974386 | rs11083974 | 19 | 49946493 | G | A | 0.247 | -0.030 | 0.019 | 1.28E-01 | 0.150 | 0.014 | 4.26E-28 | -0.198 | 0.131 | 0.820 | (0.82-0.82) | 1.31E-01 | 1.51E-01 | 4.83E-01 | 8 |
| ENSG00000143416 | 1 | SELENBP1 | 151288172 | rs17564294 | 1 | 151347700 | T | G | 0.364 | 0.027 | 0.018 | 1.37E-01 | -0.100 | 0.012 | 1.78E-17 | -0.267 | 0.182 | 0.766 | (0.76-0.77) | 1.43E-01 | 1.51E-01 | 2.09E-01 | 7 |
| ENSG00000100994 | 20 | PYGB | 25190598 | rs11699316 | 20 | 25199992 | A | G | 0.149 | 0.036 | 0.024 | 1.32E-01 | -0.079 | 0.013 | 2.07E-09 | -0.457 | 0.313 | 0.633 | (0.63-0.63) | 1.44E-01 | 1.61E-01 | 8.83E-01 | 3 |
| ENSG00000161904 | 6 | LEMD2 | 33834626 | rs2281918 | 6 | 33663055 | A | G | 0.183 | -0.030 | 0.021 | 1.47E-01 | -0.050 | 0.009 | 3.81E-09 | 0.601 | 0.427 | 1.823 | (1.82-1.83) | 1.59E-01 | 1.61E-01 | 5.07E-02 | 20 |
| ENSG00000078070 | 3 | MCCC1 | 182655858 | rs10513790 | 3 | 182815556 | T | C | 0.366 | 0.024 | 0.017 | 1.60E-01 | -0.080 | 0.007 | 3.84E-30 | -0.304 | 0.218 | 0.738 | (0.74-0.74) | 1.63E-01 | 1.76E-01 | 9.74E-01 | 20 |
| ENSG00000155903 | 3 | RASA2 | 141107612 | rs2640017 | 3 | 141335121 | G | A | 0.062 | 0.048 | 0.034 | 1.57E-01 | 0.081 | 0.013 | 1.14E-09 | 0.591 | 0.429 | 1.805 | (1.80-1.81) | 1.68E-01 | 1.78E-01 | 9.71E-01 | 7 |
| ENSG00000151693 | 2 | ASAP2 | 9437151 | rs4392227 | 2 | 9475337 | G | A | 0.260 | 0.029 | 0.021 | 1.58E-01 | -0.037 | 0.007 | 3.76E-08 | -0.788 | 0.576 | 0.455 | (0.45-0.46) | 1.71E-01 | 1.81E-01 | 7.14E-01 | 12 |
| ENSG00000167103 | 9 | PIP5KL1 | 130638467 | rs7865476 | 9 | 130735937 | G | T | 0.049 | -0.058 | 0.043 | 1.76E-01 | -0.215 | 0.022 | 1.77E-22 | 0.268 | 0.200 | 1.308 | (1.30-1.31) | 1.80E-01 | 1.82E-01 | 6.66E-02 | 8 |
| ENSG00000173267 | 10 | SNCG | 88659047 | rs1240390 | 10 | 88725984 | A | G | 0.229 | 0.023 | 0.019 | 2.19E-01 | -0.072 | 0.011 | 5.44E-12 | -0.324 | 0.268 | 0.724 | (0.72-0.73) | 2.27E-01 | 1.89E-01 | 4.01E-01 | 10 |
| ENSG00000160993 | 7 | ALKBH4 | 102044690 | rs4989124 | 7 | 102101510 | A | G | 0.229 | -0.021 | 0.020 | 2.88E-01 | -0.073 | 0.013 | 2.64E-08 | 0.284 | 0.272 | 1.329 | (1.32-1.33) | 2.97E-01 | 2.35E-01 | 8.41E-02 | 7 |
| ENSG00000143740 | 1 | SNAP47 | 227831498 | rs9786978 | 1 | 227968033 | T | C | 0.448 | -0.015 | 0.017 | 3.67E-01 | 0.041 | 0.007 | 4.35E-10 | -0.367 | 0.411 | 0.693 | (0.69-0.70) | 3.72E-01 | 3.04E-01 | 9.27E-01 | 13 |
| ENSG00000112763 | 6 | BTN2A1 | 26404958 | rs10456045 | 6 | 26404958 | A | G | 0.315 | 0.004 | 0.018 | 8.41E-01 | -0.190 | 0.015 | 2.54E-36 | -0.019 | 0.094 | 0.981 | (0.98-0.98) | 8.41E-01 | 3.76E-01 | 2.46E-02 | 20 |

| | | | | | | | | | | | | | | | | | | | | | | | |
|---|---|---|---|---|---|---|---|---|---|---|---|---|---|---|---|---|---|---|---|---|---|---|---|
| NA# | NA# | AAK1 | NA# | NA# | NA# | NA# | NA# | NA# | NA# | NA# | NA# | NA# | NA# | NA# | NA# | NA# | NA# | NA# | NA# | NA# | NA# | NA# | NA# |
| NA# | NA# | ACLY | NA# | NA# | NA# | NA# | NA# | NA# | NA# | NA# | NA# | NA# | NA# | NA# | NA# | NA# | NA# | NA# | NA# | NA# | NA# | NA# | NA# |
| NA# | NA# | AGPS | NA# | NA# | NA# | NA# | NA# | NA# | NA# | NA# | NA# | NA# | NA# | NA# | NA# | NA# | NA# | NA# | NA# | NA# | NA# | NA# | NA# |
| NA# | NA# | APPL2 | NA# | NA# | NA# | NA# | NA# | NA# | NA# | NA# | NA# | NA# | NA# | NA# | NA# | NA# | NA# | NA# | NA# | NA# | NA# | NA# | NA# |
| NA# | NA# | ARHGAP12 | NA# | NA# | NA# | NA# | NA# | NA# | NA# | NA# | NA# | NA# | NA# | NA# | NA# | NA# | NA# | NA# | NA# | NA# | NA# | NA# | NA# |
| NA# | NA# | ARNT2 | NA# | NA# | NA# | NA# | NA# | NA# | NA# | NA# | NA# | NA# | NA# | NA# | NA# | NA# | NA# | NA# | NA# | NA# | NA# | NA# | NA# |
| NA# | NA# | C12orf73 | NA# | NA# | NA# | NA# | NA# | NA# | NA# | NA# | NA# | NA# | NA# | NA# | NA# | NA# | NA# | NA# | NA# | NA# | NA# | NA# | NA# |
| NA# | NA# | CAMK2G | NA# | NA# | NA# | NA# | NA# | NA# | NA# | NA# | NA# | NA# | NA# | NA# | NA# | NA# | NA# | NA# | NA# | NA# | NA# | NA# | NA# |
| NA# | NA# | CARM1 | NA# | NA# | NA# | NA# | NA# | NA# | NA# | NA# | NA# | NA# | NA# | NA# | NA# | NA# | NA# | NA# | NA# | NA# | NA# | NA# | NA# |
| NA# | NA# | CCDC132 | NA# | NA# | NA# | NA# | NA# | NA# | NA# | NA# | NA# | NA# | NA# | NA# | NA# | NA# | NA# | NA# | NA# | NA# | NA# | NA# | NA# |
| NA# | NA# | CCS | NA# | NA# | NA# | NA# | NA# | NA# | NA# | NA# | NA# | NA# | NA# | NA# | NA# | NA# | NA# | NA# | NA# | NA# | NA# | NA# | NA# |
| NA# | NA# | CD53 | NA# | NA# | NA# | NA# | NA# | NA# | NA# | NA# | NA# | NA# | NA# | NA# | NA# | NA# | NA# | NA# | NA# | NA# | NA# | NA# | NA# |
| NA# | NA# | CDC37L1 | NA# | NA# | NA# | NA# | NA# | NA# | NA# | NA# | NA# | NA# | NA# | NA# | NA# | NA# | NA# | NA# | NA# | NA# | NA# | NA# | NA# |
| NA# | NA# | CDC42BPB | NA# | NA# | NA# | NA# | NA# | NA# | NA# | NA# | NA# | NA# | NA# | NA# | NA# | NA# | NA# | NA# | NA# | NA# | NA# | NA# | NA# |
| NA# | NA# | CENPV | NA# | NA# | NA# | NA# | NA# | NA# | NA# | NA# | NA# | NA# | NA# | NA# | NA# | NA# | NA# | NA# | NA# | NA# | NA# | NA# | NA# |
| NA# | NA# | CHI3L1 | NA# | NA# | NA# | NA# | NA# | NA# | NA# | NA# | NA# | NA# | NA# | NA# | NA# | NA# | NA# | NA# | NA# | NA# | NA# | NA# | NA# |
| NA# | NA# | CNPY4 | NA# | NA# | NA# | NA# | NA# | NA# | NA# | NA# | NA# | NA# | NA# | NA# | NA# | NA# | NA# | NA# | NA# | NA# | NA# | NA# | NA# |
| NA# | NA# | CNTN2 | NA# | NA# | NA# | NA# | NA# | NA# | NA# | NA# | NA# | NA# | NA# | NA# | NA# | NA# | NA# | NA# | NA# | NA# | NA# | NA# | NA# |
| NA# | NA# | COG6 | NA# | NA# | NA# | NA# | NA# | NA# | NA# | NA# | NA# | NA# | NA# | NA# | NA# | NA# | NA# | NA# | NA# | NA# | NA# | NA# | NA# |
| NA# | NA# | COX15 | NA# | NA# | NA# | NA# | NA# | NA# | NA# | NA# | NA# | NA# | NA# | NA# | NA# | NA# | NA# | NA# | NA# | NA# | NA# | NA# | NA# |
| NA# | NA# | CPNE4 | NA# | NA# | NA# | NA# | NA# | NA# | NA# | NA# | NA# | NA# | NA# | NA# | NA# | NA# | NA# | NA# | NA# | NA# | NA# | NA# | NA# |
| NA# | NA# | CYB5R1 | NA# | NA# | NA# | NA# | NA# | NA# | NA# | NA# | NA# | NA# | NA# | NA# | NA# | NA# | NA# | NA# | NA# | NA# | NA# | NA# | NA# |
| NA# | NA# | DEPDC5 | NA# | NA# | NA# | NA# | NA# | NA# | NA# | NA# | NA# | NA# | NA# | NA# | NA# | NA# | NA# | NA# | NA# | NA# | NA# | NA# | NA# |
| NA# | NA# | DGKA | NA# | NA# | NA# | NA# | NA# | NA# | NA# | NA# | NA# | NA# | NA# | NA# | NA# | NA# | NA# | NA# | NA# | NA# | NA# | NA# | NA# |
| NA# | NA# | DLD | NA# | NA# | NA# | NA# | NA# | NA# | NA# | NA# | NA# | NA# | NA# | NA# | NA# | NA# | NA# | NA# | NA# | NA# | NA# | NA# | NA# |
| NA# | NA# | ECHDC1 | NA# | NA# | NA# | NA# | NA# | NA# | NA# | NA# | NA# | NA# | NA# | NA# | NA# | NA# | NA# | NA# | NA# | NA# | NA# | NA# | NA# |
| NA# | NA# | EIF2B3 | NA# | NA# | NA# | NA# | NA# | NA# | NA# | NA# | NA# | NA# | NA# | NA# | NA# | NA# | NA# | NA# | NA# | NA# | NA# | NA# | NA# |
| NA# | NA# | EIF2B5 | NA# | NA# | NA# | NA# | NA# | NA# | NA# | NA# | NA# | NA# | NA# | NA# | NA# | NA# | NA# | NA# | NA# | NA# | NA# | NA# | NA# |
| NA# | NA# | EPG5 | NA# | NA# | NA# | NA# | NA# | NA# | NA# | NA# | NA# | NA# | NA# | NA# | NA# | NA# | NA# | NA# | NA# | NA# | NA# | NA# | NA# |
| NA# | NA# | EPM2A | NA# | NA# | NA# | NA# | NA# | NA# | NA# | NA# | NA# | NA# | NA# | NA# | NA# | NA# | NA# | NA# | NA# | NA# | NA# | NA# | NA# |
| NA# | NA# | EVI5 | NA# | NA# | NA# | NA# | NA# | NA# | NA# | NA# | NA# | NA# | NA# | NA# | NA# | NA# | NA# | NA# | NA# | NA# | NA# | NA# | NA# |
| NA# | NA# | FAIM2 | NA# | NA# | NA# | NA# | NA# | NA# | NA# | NA# | NA# | NA# | NA# | NA# | NA# | NA# | NA# | NA# | NA# | NA# | NA# | NA# | NA# |
| NA# | NA# | FAM118B | NA# | NA# | NA# | NA# | NA# | NA# | NA# | NA# | NA# | NA# | NA# | NA# | NA# | NA# | NA# | NA# | NA# | NA# | NA# | NA# | NA# |
| NA# | NA# | FAM177A1 | NA# | NA# | NA# | NA# | NA# | NA# | NA# | NA# | NA# | NA# | NA# | NA# | NA# | NA# | NA# | NA# | NA# | NA# | NA# | NA# | NA# |
| NA# | NA# | FLAD1 | NA# | NA# | NA# | NA# | NA# | NA# | NA# | NA# | NA# | NA# | NA# | NA# | NA# | NA# | NA# | NA# | NA# | NA# | NA# | NA# | NA# |
| NA# | NA# | FLCN | NA# | NA# | NA# | NA# | NA# | NA# | NA# | NA# | NA# | NA# | NA# | NA# | NA# | NA# | NA# | NA# | NA# | NA# | NA# | NA# | NA# |
| NA# | NA# | FUCA1 | NA# | NA# | NA# | NA# | NA# | NA# | NA# | NA# | NA# | NA# | NA# | NA# | NA# | NA# | NA# | NA# | NA# | NA# | NA# | NA# | NA# |
| NA# | NA# | FYCO1 | NA# | NA# | NA# | NA# | NA# | NA# | NA# | NA# | NA# | NA# | NA# | NA# | NA# | NA# | NA# | NA# | NA# | NA# | NA# | NA# | NA# |
| NA# | NA# | GALT | NA# | NA# | NA# | NA# | NA# | NA# | NA# | NA# | NA# | NA# | NA# | NA# | NA# | NA# | NA# | NA# | NA# | NA# | NA# | NA# | NA# |
| NA# | NA# | GLRA3 | NA# | NA# | NA# | NA# | NA# | NA# | NA# | NA# | NA# | NA# | NA# | NA# | NA# | NA# | NA# | NA# | NA# | NA# | NA# | NA# | NA# |
| NA# | NA# | GNB1 | NA# | NA# | NA# | NA# | NA# | NA# | NA# | NA# | NA# | NA# | NA# | NA# | NA# | NA# | NA# | NA# | NA# | NA# | NA# | NA# | NA# |
| NA# | NA# | GOSR2 | NA# | NA# | NA# | NA# | NA# | NA# | NA# | NA# | NA# | NA# | NA# | NA# | NA# | NA# | NA# | NA# | NA# | NA# | NA# | NA# | NA# |
| NA# | NA# | GPC5 | NA# | NA# | NA# | NA# | NA# | NA# | NA# | NA# | NA# | NA# | NA# | NA# | NA# | NA# | NA# | NA# | NA# | NA# | NA# | NA# | NA# |
| NA# | NA# | GTF3C3 | NA# | NA# | NA# | NA# | NA# | NA# | NA# | NA# | NA# | NA# | NA# | NA# | NA# | NA# | NA# | NA# | NA# | NA# | NA# | NA# | NA# |

| | | | | | | | | | | | | | | | | | | | | | | | |
|---|---|---|---|---|---|---|---|---|---|---|---|---|---|---|---|---|---|---|---|---|---|---|---|
| NA# | NA# | HCLS1 | NA# | NA# | NA# | NA# | NA# | NA# | NA# | NA# | NA# | NA# | NA# | NA# | NA# | NA# | NA# | NA# | NA# | NA# | NA# | NA# | NA# |
| NA# | NA# | HOOK1 | NA# | NA# | NA# | NA# | NA# | NA# | NA# | NA# | NA# | NA# | NA# | NA# | NA# | NA# | NA# | NA# | NA# | NA# | NA# | NA# | NA# |
| NA# | NA# | IGSF21 | NA# | NA# | NA# | NA# | NA# | NA# | NA# | NA# | NA# | NA# | NA# | NA# | NA# | NA# | NA# | NA# | NA# | NA# | NA# | NA# | NA# |
| NA# | NA# | IKBKAP | NA# | NA# | NA# | NA# | NA# | NA# | NA# | NA# | NA# | NA# | NA# | NA# | NA# | NA# | NA# | NA# | NA# | NA# | NA# | NA# | NA# |
| NA# | NA# | INPP4A | NA# | NA# | NA# | NA# | NA# | NA# | NA# | NA# | NA# | NA# | NA# | NA# | NA# | NA# | NA# | NA# | NA# | NA# | NA# | NA# | NA# |
| NA# | NA# | IQGAP1 | NA# | NA# | NA# | NA# | NA# | NA# | NA# | NA# | NA# | NA# | NA# | NA# | NA# | NA# | NA# | NA# | NA# | NA# | NA# | NA# | NA# |
| NA# | NA# | KIAA0195 | NA# | NA# | NA# | NA# | NA# | NA# | NA# | NA# | NA# | NA# | NA# | NA# | NA# | NA# | NA# | NA# | NA# | NA# | NA# | NA# | NA# |
| NA# | NA# | KIAA1468 | NA# | NA# | NA# | NA# | NA# | NA# | NA# | NA# | NA# | NA# | NA# | NA# | NA# | NA# | NA# | NA# | NA# | NA# | NA# | NA# | NA# |
| NA# | NA# | KLHL3 | NA# | NA# | NA# | NA# | NA# | NA# | NA# | NA# | NA# | NA# | NA# | NA# | NA# | NA# | NA# | NA# | NA# | NA# | NA# | NA# | NA# |
| NA# | NA# | LANCL1 | NA# | NA# | NA# | NA# | NA# | NA# | NA# | NA# | NA# | NA# | NA# | NA# | NA# | NA# | NA# | NA# | NA# | NA# | NA# | NA# | NA# |
| NA# | NA# | LLGL1 | NA# | NA# | NA# | NA# | NA# | NA# | NA# | NA# | NA# | NA# | NA# | NA# | NA# | NA# | NA# | NA# | NA# | NA# | NA# | NA# | NA# |
| NA# | NA# | LMAN2 | NA# | NA# | NA# | NA# | NA# | NA# | NA# | NA# | NA# | NA# | NA# | NA# | NA# | NA# | NA# | NA# | NA# | NA# | NA# | NA# | NA# |
| NA# | NA# | LRIG1 | NA# | NA# | NA# | NA# | NA# | NA# | NA# | NA# | NA# | NA# | NA# | NA# | NA# | NA# | NA# | NA# | NA# | NA# | NA# | NA# | NA# |
| NA# | NA# | LRP4 | NA# | NA# | NA# | NA# | NA# | NA# | NA# | NA# | NA# | NA# | NA# | NA# | NA# | NA# | NA# | NA# | NA# | NA# | NA# | NA# | NA# |
| NA# | NA# | MAP1LC3A | NA# | NA# | NA# | NA# | NA# | NA# | NA# | NA# | NA# | NA# | NA# | NA# | NA# | NA# | NA# | NA# | NA# | NA# | NA# | NA# | NA# |
| NA# | NA# | MAPRE1 | NA# | NA# | NA# | NA# | NA# | NA# | NA# | NA# | NA# | NA# | NA# | NA# | NA# | NA# | NA# | NA# | NA# | NA# | NA# | NA# | NA# |
| NA# | NA# | MERTK | NA# | NA# | NA# | NA# | NA# | NA# | NA# | NA# | NA# | NA# | NA# | NA# | NA# | NA# | NA# | NA# | NA# | NA# | NA# | NA# | NA# |
| NA# | NA# | MPZL1 | NA# | NA# | NA# | NA# | NA# | NA# | NA# | NA# | NA# | NA# | NA# | NA# | NA# | NA# | NA# | NA# | NA# | NA# | NA# | NA# | NA# |
| NA# | NA# | MYO18A | NA# | NA# | NA# | NA# | NA# | NA# | NA# | NA# | NA# | NA# | NA# | NA# | NA# | NA# | NA# | NA# | NA# | NA# | NA# | NA# | NA# |
| NA# | NA# | NADK2 | NA# | NA# | NA# | NA# | NA# | NA# | NA# | NA# | NA# | NA# | NA# | NA# | NA# | NA# | NA# | NA# | NA# | NA# | NA# | NA# | NA# |
| NA# | NA# | NAGK | NA# | NA# | NA# | NA# | NA# | NA# | NA# | NA# | NA# | NA# | NA# | NA# | NA# | NA# | NA# | NA# | NA# | NA# | NA# | NA# | NA# |
| NA# | NA# | NFU1 | NA# | NA# | NA# | NA# | NA# | NA# | NA# | NA# | NA# | NA# | NA# | NA# | NA# | NA# | NA# | NA# | NA# | NA# | NA# | NA# | NA# |
| NA# | NA# | NRP1 | NA# | NA# | NA# | NA# | NA# | NA# | NA# | NA# | NA# | NA# | NA# | NA# | NA# | NA# | NA# | NA# | NA# | NA# | NA# | NA# | NA# |
| NA# | NA# | NSUN2 | NA# | NA# | NA# | NA# | NA# | NA# | NA# | NA# | NA# | NA# | NA# | NA# | NA# | NA# | NA# | NA# | NA# | NA# | NA# | NA# | NA# |
| NA# | NA# | NT5C | NA# | NA# | NA# | NA# | NA# | NA# | NA# | NA# | NA# | NA# | NA# | NA# | NA# | NA# | NA# | NA# | NA# | NA# | NA# | NA# | NA# |
| NA# | NA# | NUBPL | NA# | NA# | NA# | NA# | NA# | NA# | NA# | NA# | NA# | NA# | NA# | NA# | NA# | NA# | NA# | NA# | NA# | NA# | NA# | NA# | NA# |
| NA# | NA# | OPALIN | NA# | NA# | NA# | NA# | NA# | NA# | NA# | NA# | NA# | NA# | NA# | NA# | NA# | NA# | NA# | NA# | NA# | NA# | NA# | NA# | NA# |
| NA# | NA# | ORAI2 | NA# | NA# | NA# | NA# | NA# | NA# | NA# | NA# | NA# | NA# | NA# | NA# | NA# | NA# | NA# | NA# | NA# | NA# | NA# | NA# | NA# |
| NA# | NA# | OSBPL11 | NA# | NA# | NA# | NA# | NA# | NA# | NA# | NA# | NA# | NA# | NA# | NA# | NA# | NA# | NA# | NA# | NA# | NA# | NA# | NA# | NA# |
| NA# | NA# | PAICS | NA# | NA# | NA# | NA# | NA# | NA# | NA# | NA# | NA# | NA# | NA# | NA# | NA# | NA# | NA# | NA# | NA# | NA# | NA# | NA# | NA# |
| NA# | NA# | PARP10 | NA# | NA# | NA# | NA# | NA# | NA# | NA# | NA# | NA# | NA# | NA# | NA# | NA# | NA# | NA# | NA# | NA# | NA# | NA# | NA# | NA# |
| NA# | NA# | PDE2A | NA# | NA# | NA# | NA# | NA# | NA# | NA# | NA# | NA# | NA# | NA# | NA# | NA# | NA# | NA# | NA# | NA# | NA# | NA# | NA# | NA# |
| NA# | NA# | PDK1 | NA# | NA# | NA# | NA# | NA# | NA# | NA# | NA# | NA# | NA# | NA# | NA# | NA# | NA# | NA# | NA# | NA# | NA# | NA# | NA# | NA# |
| NA# | NA# | PDXDC1 | NA# | NA# | NA# | NA# | NA# | NA# | NA# | NA# | NA# | NA# | NA# | NA# | NA# | NA# | NA# | NA# | NA# | NA# | NA# | NA# | NA# |
| NA# | NA# | PIK3IP1 | NA# | NA# | NA# | NA# | NA# | NA# | NA# | NA# | NA# | NA# | NA# | NA# | NA# | NA# | NA# | NA# | NA# | NA# | NA# | NA# | NA# |
| NA# | NA# | PLCD3 | NA# | NA# | NA# | NA# | NA# | NA# | NA# | NA# | NA# | NA# | NA# | NA# | NA# | NA# | NA# | NA# | NA# | NA# | NA# | NA# | NA# |
| NA# | NA# | PMPCB | NA# | NA# | NA# | NA# | NA# | NA# | NA# | NA# | NA# | NA# | NA# | NA# | NA# | NA# | NA# | NA# | NA# | NA# | NA# | NA# | NA# |
| NA# | NA# | PPM1F | NA# | NA# | NA# | NA# | NA# | NA# | NA# | NA# | NA# | NA# | NA# | NA# | NA# | NA# | NA# | NA# | NA# | NA# | NA# | NA# | NA# |
| NA# | NA# | PPTC7 | NA# | NA# | NA# | NA# | NA# | NA# | NA# | NA# | NA# | NA# | NA# | NA# | NA# | NA# | NA# | NA# | NA# | NA# | NA# | NA# | NA# |
| NA# | NA# | PREX1 | NA# | NA# | NA# | NA# | NA# | NA# | NA# | NA# | NA# | NA# | NA# | NA# | NA# | NA# | NA# | NA# | NA# | NA# | NA# | NA# | NA# |
| NA# | NA# | PSMB9 | NA# | NA# | NA# | NA# | NA# | NA# | NA# | NA# | NA# | NA# | NA# | NA# | NA# | NA# | NA# | NA# | NA# | NA# | NA# | NA# | NA# |
| NA# | NA# | PTK2B | NA# | NA# | NA# | NA# | NA# | NA# | NA# | NA# | NA# | NA# | NA# | NA# | NA# | NA# | NA# | NA# | NA# | NA# | NA# | NA# | NA# |
| NA# | NA# | PTPMT1 | NA# | NA# | NA# | NA# | NA# | NA# | NA# | NA# | NA# | NA# | NA# | NA# | NA# | NA# | NA# | NA# | NA# | NA# | NA# | NA# | NA# |
| NA# | NA# | PTRH1 | NA# | NA# | NA# | NA# | NA# | NA# | NA# | NA# | NA# | NA# | NA# | NA# | NA# | NA# | NA# | NA# | NA# | NA# | NA# | NA# | NA# |

| | | | | | | | | | | | | | | | | | | | | | | | |
|---|---|---|---|---|---|---|---|---|---|---|---|---|---|---|---|---|---|---|---|---|---|---|---|
| NA# | NA# | RAB24 | NA# | NA# | NA# | NA# | NA# | NA# | NA# | NA# | NA# | NA# | NA# | NA# | NA# | NA# | NA# | NA# | NA# | NA# | NA# | NA# | NA# |
| NA# | NA# | RAB40C | NA# | NA# | NA# | NA# | NA# | NA# | NA# | NA# | NA# | NA# | NA# | NA# | NA# | NA# | NA# | NA# | NA# | NA# | NA# | NA# | NA# |
| NA# | NA# | RALB | NA# | NA# | NA# | NA# | NA# | NA# | NA# | NA# | NA# | NA# | NA# | NA# | NA# | NA# | NA# | NA# | NA# | NA# | NA# | NA# | NA# |
| NA# | NA# | RDH11 | NA# | NA# | NA# | NA# | NA# | NA# | NA# | NA# | NA# | NA# | NA# | NA# | NA# | NA# | NA# | NA# | NA# | NA# | NA# | NA# | NA# |
| NA# | NA# | RHOQ | NA# | NA# | NA# | NA# | NA# | NA# | NA# | NA# | NA# | NA# | NA# | NA# | NA# | NA# | NA# | NA# | NA# | NA# | NA# | NA# | NA# |
| NA# | NA# | RRAS2 | NA# | NA# | NA# | NA# | NA# | NA# | NA# | NA# | NA# | NA# | NA# | NA# | NA# | NA# | NA# | NA# | NA# | NA# | NA# | NA# | NA# |
| NA# | NA# | RRP12 | NA# | NA# | NA# | NA# | NA# | NA# | NA# | NA# | NA# | NA# | NA# | NA# | NA# | NA# | NA# | NA# | NA# | NA# | NA# | NA# | NA# |
| NA# | NA# | RSU1 | NA# | NA# | NA# | NA# | NA# | NA# | NA# | NA# | NA# | NA# | NA# | NA# | NA# | NA# | NA# | NA# | NA# | NA# | NA# | NA# | NA# |
| NA# | NA# | SBF2 | NA# | NA# | NA# | NA# | NA# | NA# | NA# | NA# | NA# | NA# | NA# | NA# | NA# | NA# | NA# | NA# | NA# | NA# | NA# | NA# | NA# |
| NA# | NA# | SCGN | NA# | NA# | NA# | NA# | NA# | NA# | NA# | NA# | NA# | NA# | NA# | NA# | NA# | NA# | NA# | NA# | NA# | NA# | NA# | NA# | NA# |
| NA# | NA# | SDF4 | NA# | NA# | NA# | NA# | NA# | NA# | NA# | NA# | NA# | NA# | NA# | NA# | NA# | NA# | NA# | NA# | NA# | NA# | NA# | NA# | NA# |
| NA# | NA# | SEPTIN4 | NA# | NA# | NA# | NA# | NA# | NA# | NA# | NA# | NA# | NA# | NA# | NA# | NA# | NA# | NA# | NA# | NA# | NA# | NA# | NA# | NA# |
| NA# | NA# | SKIV2L | NA# | NA# | NA# | NA# | NA# | NA# | NA# | NA# | NA# | NA# | NA# | NA# | NA# | NA# | NA# | NA# | NA# | NA# | NA# | NA# | NA# |
| NA# | NA# | SLC44A2 | NA# | NA# | NA# | NA# | NA# | NA# | NA# | NA# | NA# | NA# | NA# | NA# | NA# | NA# | NA# | NA# | NA# | NA# | NA# | NA# | NA# |
| NA# | NA# | SLC4A8 | NA# | NA# | NA# | NA# | NA# | NA# | NA# | NA# | NA# | NA# | NA# | NA# | NA# | NA# | NA# | NA# | NA# | NA# | NA# | NA# | NA# |
| NA# | NA# | SLC7A6 | NA# | NA# | NA# | NA# | NA# | NA# | NA# | NA# | NA# | NA# | NA# | NA# | NA# | NA# | NA# | NA# | NA# | NA# | NA# | NA# | NA# |
| NA# | NA# | SMCR8 | NA# | NA# | NA# | NA# | NA# | NA# | NA# | NA# | NA# | NA# | NA# | NA# | NA# | NA# | NA# | NA# | NA# | NA# | NA# | NA# | NA# |
| NA# | NA# | SSH1 | NA# | NA# | NA# | NA# | NA# | NA# | NA# | NA# | NA# | NA# | NA# | NA# | NA# | NA# | NA# | NA# | NA# | NA# | NA# | NA# | NA# |
| NA# | NA# | STEAP2 | NA# | NA# | NA# | NA# | NA# | NA# | NA# | NA# | NA# | NA# | NA# | NA# | NA# | NA# | NA# | NA# | NA# | NA# | NA# | NA# | NA# |
| NA# | NA# | STX1A | NA# | NA# | NA# | NA# | NA# | NA# | NA# | NA# | NA# | NA# | NA# | NA# | NA# | NA# | NA# | NA# | NA# | NA# | NA# | NA# | NA# |
| NA# | NA# | SUMF2 | NA# | NA# | NA# | NA# | NA# | NA# | NA# | NA# | NA# | NA# | NA# | NA# | NA# | NA# | NA# | NA# | NA# | NA# | NA# | NA# | NA# |
| NA# | NA# | TAPT1 | NA# | NA# | NA# | NA# | NA# | NA# | NA# | NA# | NA# | NA# | NA# | NA# | NA# | NA# | NA# | NA# | NA# | NA# | NA# | NA# | NA# |
| NA# | NA# | TM7SF2 | NA# | NA# | NA# | NA# | NA# | NA# | NA# | NA# | NA# | NA# | NA# | NA# | NA# | NA# | NA# | NA# | NA# | NA# | NA# | NA# | NA# |
| NA# | NA# | TMA7 | NA# | NA# | NA# | NA# | NA# | NA# | NA# | NA# | NA# | NA# | NA# | NA# | NA# | NA# | NA# | NA# | NA# | NA# | NA# | NA# | NA# |
| NA# | NA# | TMEM132A | NA# | NA# | NA# | NA# | NA# | NA# | NA# | NA# | NA# | NA# | NA# | NA# | NA# | NA# | NA# | NA# | NA# | NA# | NA# | NA# | NA# |
| NA# | NA# | TMEM150C | NA# | NA# | NA# | NA# | NA# | NA# | NA# | NA# | NA# | NA# | NA# | NA# | NA# | NA# | NA# | NA# | NA# | NA# | NA# | NA# | NA# |
| NA# | NA# | TMEM179B | NA# | NA# | NA# | NA# | NA# | NA# | NA# | NA# | NA# | NA# | NA# | NA# | NA# | NA# | NA# | NA# | NA# | NA# | NA# | NA# | NA# |
| NA# | NA# | TMTC4 | NA# | NA# | NA# | NA# | NA# | NA# | NA# | NA# | NA# | NA# | NA# | NA# | NA# | NA# | NA# | NA# | NA# | NA# | NA# | NA# | NA# |
| NA# | NA# | TPD52L2 | NA# | NA# | NA# | NA# | NA# | NA# | NA# | NA# | NA# | NA# | NA# | NA# | NA# | NA# | NA# | NA# | NA# | NA# | NA# | NA# | NA# |
| NA# | NA# | TYW5 | NA# | NA# | NA# | NA# | NA# | NA# | NA# | NA# | NA# | NA# | NA# | NA# | NA# | NA# | NA# | NA# | NA# | NA# | NA# | NA# | NA# |
| NA# | NA# | UFSP2 | NA# | NA# | NA# | NA# | NA# | NA# | NA# | NA# | NA# | NA# | NA# | NA# | NA# | NA# | NA# | NA# | NA# | NA# | NA# | NA# | NA# |
| NA# | NA# | VAMP1 | NA# | NA# | NA# | NA# | NA# | NA# | NA# | NA# | NA# | NA# | NA# | NA# | NA# | NA# | NA# | NA# | NA# | NA# | NA# | NA# | NA# |
| NA# | NA# | VAT1L | NA# | NA# | NA# | NA# | NA# | NA# | NA# | NA# | NA# | NA# | NA# | NA# | NA# | NA# | NA# | NA# | NA# | NA# | NA# | NA# | NA# |
| NA# | NA# | VKORC1 | NA# | NA# | NA# | NA# | NA# | NA# | NA# | NA# | NA# | NA# | NA# | NA# | NA# | NA# | NA# | NA# | NA# | NA# | NA# | NA# | NA# |
| NA# | NA# | VWA7 | NA# | NA# | NA# | NA# | NA# | NA# | NA# | NA# | NA# | NA# | NA# | NA# | NA# | NA# | NA# | NA# | NA# | NA# | NA# | NA# | NA# |
| NA# | NA# | WBP2 | NA# | NA# | NA# | NA# | NA# | NA# | NA# | NA# | NA# | NA# | NA# | NA# | NA# | NA# | NA# | NA# | NA# | NA# | NA# | NA# | NA# |
| NA# | NA# | WDR48 | NA# | NA# | NA# | NA# | NA# | NA# | NA# | NA# | NA# | NA# | NA# | NA# | NA# | NA# | NA# | NA# | NA# | NA# | NA# | NA# | NA# |
| NA# | NA# | XRCC6BP1 | NA# | NA# | NA# | NA# | NA# | NA# | NA# | NA# | NA# | NA# | NA# | NA# | NA# | NA# | NA# | NA# | NA# | NA# | NA# | NA# | NA# |
| NA# | NA# | HLA-DRB1 | NA# | NA# | NA# | NA# | NA# | NA# | NA# | NA# | NA# | NA# | NA# | NA# | NA# | NA# | NA# | NA# | NA# | NA# | NA# | NA# | NA# |

SMR: summary-data-based Mendelian randomization; PWAS: proteome-wide association study; pQTL: protein quantitative trait loci; SNP: single nucleotide polymorphism; A1: the effect allele; A2: the other allele; Freq: frequency of the effect allele; SE: standard error; OR: odds ratio; CI: confidence interval; HEIDI: heterogeneity in dependent instruments test; Benjamini-Hochberg method was used in FDR adjustment; #Pexposure>5e-8.

**Table S6. Colocalization analysis investigating whether the same causal variant is responsible for both brain protein expression and multiple sclerosis**

| Gene | $N_{snp}$ | PP.H0.abf | PP.H1.abf | PP.H2.abf | PP.H3.abf | PP.H4.abf |
|---|---|---|---|---|---|---|
| ZC2HC1A | 81 | 1.82E-08 | 1.57E-05 | 1.52E-05 | 1.22E-02 | 0.988 |
| TRAF3 | 102 | 1.06E-06 | 1.14E-03 | 1.55E-05 | 1.57E-02 | 0.983 |
| SHMT1 | 56 | 3.81E-30 | 7.28E-30 | 6.97E-03 | 1.23E-02 | 0.981 |
| HLA-B | 19 | 4.19E-117 | 3.03E-04 | 2.97E-115 | 2.05E-02 | 0.979 |
| ABCB9 | 51 | 1.13E-07 | 2.74E-05 | 1.10E-04 | 2.57E-02 | 0.974 |
| TSFM | 83 | 2.67E-22 | 7.95E-17 | 1.38E-07 | 4.01E-02 | 0.960 |
| ICA1L | 81 | 8.83E-10 | 1.84E-10 | 3.59E-02 | 6.51E-03 | 0.958 |
| FAM120B | 156 | 3.52E-04 | 1.60E-04 | 2.98E-02 | 1.26E-02 | 0.957 |
| MTHFR | 144 | 2.38E-18 | 1.51E-19 | 1.16E-01 | 6.48E-03 | 0.877 |
| HMGCL | 54 | 3.34E-07 | 3.44E-08 | 1.27E-01 | 1.22E-02 | 0.860 |
| PREX1 | 226 | 3.62E-03 | 1.17E-01 | 1.86E-03 | 5.96E-02 | 0.817 |
| LLGL1 | 99 | 7.70E-02 | 1.27E-01 | 1.05E-02 | 1.65E-02 | 0.769 |
| MERTK | 146 | 7.69E-04 | 1.73E-01 | 2.80E-04 | 6.21E-02 | 0.764 |
| AUH | 127 | 1.11E-08 | 3.81E-10 | 2.40E-01 | 7.51E-03 | 0.753 |
| PARP10 | 67 | 1.18E-01 | 3.36E-02 | 8.80E-02 | 2.42E-02 | 0.736 |
| GIMAP4 | 113 | 1.14E-11 | 2.20E-12 | 2.42E-01 | 4.60E-02 | 0.712 |
| WARS | 91 | 1.37E-05 | 4.92E-06 | 2.49E-01 | 8.86E-02 | 0.662 |
| RAB24 | 73 | 3.34E-03 | 3.30E-02 | 2.77E-02 | 2.74E-01 | 0.662 |
| LRP4 | 65 | 2.00E-01 | 6.00E-02 | 7.33E-02 | 2.13E-02 | 0.645 |
| MBLAC1 | 70 | 1.55E-23 | 1.63E-25 | 3.52E-01 | 3.07E-03 | 0.645 |
| POGLUT1 | 143 | 9.94E-07 | 9.88E-03 | 3.89E-05 | 3.87E-01 | 0.604 |
| CA4 | 45 | 3.22E-03 | 2.04E-05 | 3.97E-01 | 1.92E-03 | 0.598 |
| QPRT | 28 | 1.70E-27 | 1.96E-29 | 4.04E-01 | 4.05E-03 | 0.592 |
| LZTFL1 | 143 | 3.99E-04 | 8.03E-06 | 4.65E-01 | 8.83E-03 | 0.526 |
| TMEM132A | 120 | 1.04E-06 | 4.57E-01 | 6.07E-08 | 2.61E-02 | 0.517 |
| GBAS | 87 | 1.85E-08 | 9.53E-08 | 7.88E-02 | 4.05E-01 | 0.516 |
| EVI5 | 116 | 2.59E-09 | 4.63E-01 | 1.77E-10 | 3.10E-02 | 0.506 |
| ACOT7 | 115 | 1.94E-04 | 4.02E-05 | 4.25E-01 | 8.76E-02 | 0.487 |
| DOC2A | 75 | 5.68E-06 | 1.97E-04 | 1.45E-02 | 5.02E-01 | 0.483 |
| CARM1 | 70 | 4.33E-02 | 5.17E-03 | 4.23E-01 | 5.00E-02 | 0.478 |
| RRP12 | 90 | 3.39E-01 | 1.71E-01 | 3.38E-02 | 1.66E-02 | 0.440 |
| MADD | 99 | 5.71E-08 | 4.23E-07 | 6.79E-02 | 5.03E-01 | 0.429 |
| TRIM65 | 61 | 9.07E-06 | 6.25E-07 | 5.42E-01 | 3.69E-02 | 0.421 |
| RNH1 | 81 | 3.24E-18 | 3.08E-20 | 5.76E-01 | 5.04E-03 | 0.419 |
| PANK2 | 103 | 2.80E-09 | 1.03E-11 | 5.91E-01 | 1.77E-03 | 0.408 |

| | | | | | | |
|---|---|---|---|---|---|---|
| IQGAP1 | 141 | 7.67E-03 | 5.51E-01 | 5.35E-04 | 3.80E-02 | 0.403 |
| VAMP1 | 104 | 9.84E-02 | 1.16E-02 | 4.48E-01 | 5.22E-02 | 0.389 |
| TXN | 141 | 3.95E-11 | 1.90E-13 | 6.22E-01 | 2.61E-03 | 0.375 |
| TREX1 | 62 | 9.63E-08 | 2.32E-09 | 6.15E-01 | 1.44E-02 | 0.371 |
| FAM172A | 160 | 1.02E-03 | 4.04E-05 | 6.23E-01 | 2.42E-02 | 0.352 |
| PPAPDC2 | 135 | 1.97E-02 | 1.20E-04 | 6.48E-01 | 3.64E-03 | 0.329 |
| ACLY | 101 | 5.40E-01 | 1.19E-01 | 1.54E-02 | 3.07E-03 | 0.323 |
| FOLH1 | 49 | 9.78E-04 | 1.74E-05 | 6.72E-01 | 1.17E-02 | 0.315 |
| WBP2 | 60 | 2.19E-01 | 1.77E-02 | 4.36E-01 | 3.49E-02 | 0.293 |
| RSU1 | 245 | 4.55E-02 | 7.50E-04 | 6.52E-01 | 1.04E-02 | 0.291 |
| XRCC6BP1 | 71 | 5.72E-03 | 2.29E-01 | 1.22E-02 | 4.87E-01 | 0.266 |
| CAMK2G | 78 | 4.87E-01 | 1.96E-01 | 4.24E-02 | 1.69E-02 | 0.258 |
| COX15 | 81 | 1.57E-01 | 3.32E-02 | 4.58E-01 | 9.65E-02 | 0.255 |
| HCLS1 | 92 | 1.81E-01 | 1.60E-01 | 2.22E-01 | 1.96E-01 | 0.241 |
| GLB1 | 120 | 1.76E-05 | 4.52E-06 | 6.04E-01 | 1.55E-01 | 0.240 |
| RFT1 | 86 | 6.29E-14 | 6.18E-16 | 7.53E-01 | 7.16E-03 | 0.240 |
| CDC42BPB | 99 | 4.40E-01 | 5.60E-02 | 2.43E-01 | 3.07E-02 | 0.230 |
| CORO1C | 128 | 3.97E-09 | 1.25E-10 | 7.49E-01 | 2.33E-02 | 0.228 |
| STX1A | 74 | 6.33E-01 | 1.98E-02 | 1.16E-01 | 3.40E-03 | 0.227 |
| PTPMT1 | 38 | 6.59E-01 | 1.33E-02 | 1.15E-01 | 2.11E-03 | 0.211 |
| FLCN | 92 | 6.66E-01 | 5.81E-02 | 6.88E-02 | 5.79E-03 | 0.201 |
| HSD17B4 | 149 | 2.68E-15 | 4.52E-16 | 6.87E-01 | 1.16E-01 | 0.197 |
| RRAS2 | 100 | 4.80E-01 | 3.00E-01 | 1.71E-02 | 1.05E-02 | 0.192 |
| EIF2B3 | 102 | 3.81E-01 | 9.64E-03 | 4.10E-01 | 1.02E-02 | 0.189 |
| PIK3IP1 | 62 | 6.44E-01 | 9.20E-02 | 6.61E-02 | 9.26E-03 | 0.189 |
| ALCAM | 169 | 2.76E-03 | 5.12E-05 | 7.94E-01 | 1.45E-02 | 0.189 |
| ASAP3 | 87 | 3.47E-02 | 2.16E-04 | 7.73E-01 | 4.63E-03 | 0.188 |
| RASA2 | 137 | 6.35E-02 | 2.39E-03 | 7.23E-01 | 2.70E-02 | 0.185 |
| IFIT5 | 97 | 6.97E-12 | 3.29E-14 | 8.22E-01 | 3.71E-03 | 0.174 |
| EPG5 | 122 | 4.91E-01 | 2.61E-02 | 2.99E-01 | 1.57E-02 | 0.169 |
| DLD | 156 | 2.89E-02 | 2.88E-04 | 7.98E-01 | 7.78E-03 | 0.165 |
| SDF4 | 50 | 5.69E-01 | 1.21E-02 | 2.48E-01 | 5.10E-03 | 0.165 |
| NFU1 | 92 | 6.74E-03 | 7.43E-05 | 8.22E-01 | 8.90E-03 | 0.162 |
| LMAN2 | 75 | 7.61E-02 | 7.48E-01 | 1.79E-03 | 1.74E-02 | 0.157 |
| TYW5 | 75 | 5.49E-01 | 1.59E-01 | 1.07E-01 | 3.08E-02 | 0.154 |
| TTC5 | 171 | 6.39E-09 | 7.55E-11 | 8.41E-01 | 9.78E-03 | 0.150 |
| SCGN | 167 | 5.72E-01 | 1.95E-01 | 6.45E-02 | 2.18E-02 | 0.146 |
| HLA-DRB1 | 10 | 9.49E-286 | 7.93E-01 | 7.94E-287 | 6.61E-02 | 0.141 |
| SUMF2 | 75 | 1.11E-01 | 7.18E-01 | 4.75E-03 | 3.07E-02 | 0.136 |

| | | | | | | |
|---|---|---|---|---|---|---|
| CDC37L1 | 138 | 6.29E-01 | 3.81E-03 | 2.34E-01 | 1.28E-03 | 0.132 |
| MPI | 78 | 3.90E-19 | 6.86E-21 | 8.59E-01 | 1.50E-02 | 0.126 |
| TRAP1 | 69 | 1.44E-05 | 3.89E-08 | 8.72E-01 | 2.23E-03 | 0.126 |
| PPTC7 | 49 | 2.73E-01 | 1.47E-03 | 5.96E-01 | 3.08E-03 | 0.126 |
| ENPP4 | 109 | 1.70E-04 | 1.14E-06 | 8.73E-01 | 5.76E-03 | 0.121 |
| ERAP2 | 132 | 1.51E-22 | 9.70E-25 | 8.74E-01 | 5.52E-03 | 0.120 |
| ALKBH4 | 40 | 3.55E-02 | 1.63E-04 | 8.41E-01 | 3.74E-03 | 0.120 |
| SFXN4 | 82 | 4.21E-16 | 1.43E-18 | 8.86E-01 | 2.90E-03 | 0.111 |
| GNB1 | 73 | 7.89E-01 | 3.17E-02 | 6.59E-02 | 2.53E-03 | 0.111 |
| KIAA0195 | 103 | 1.82E-01 | 6.71E-01 | 7.76E-03 | 2.85E-02 | 0.110 |
| ANXA6 | 210 | 2.29E-04 | 1.65E-06 | 8.88E-01 | 6.29E-03 | 0.106 |
| COQ9 | 104 | 6.05E-04 | 2.30E-06 | 8.92E-01 | 3.28E-03 | 0.104 |
| ULK3 | 86 | 1.08E-34 | 2.32E-36 | 8.79E-01 | 1.88E-02 | 0.103 |
| TMA7 | 67 | 7.02E-01 | 1.65E-02 | 1.75E-01 | 4.01E-03 | 0.102 |
| DUSP19 | 76 | 5.23E-02 | 2.91E-04 | 8.42E-01 | 4.59E-03 | 0.101 |
| TFAM | 85 | 2.30E-03 | 7.75E-06 | 8.99E-01 | 2.93E-03 | 0.095 |
| MKRN1 | 94 | 2.26E-04 | 6.23E-07 | 9.02E-01 | 2.39E-03 | 0.095 |
| CBR3 | 122 | 1.18E-57 | 4.69E-60 | 9.02E-01 | 3.49E-03 | 0.095 |
| C12orf73 | 161 | 2.84E-02 | 8.42E-04 | 8.57E-01 | 2.53E-02 | 0.088 |
| KIAA1468 | 149 | 1.78E-01 | 5.78E-03 | 7.06E-01 | 2.28E-02 | 0.087 |
| PDE2A | 145 | 7.95E-01 | 5.34E-02 | 6.25E-02 | 4.12E-03 | 0.085 |
| COG6 | 152 | 6.75E-01 | 2.05E-01 | 2.79E-02 | 8.39E-03 | 0.084 |
| NFXL1 | 78 | 4.36E-05 | 4.99E-07 | 9.07E-01 | 1.03E-02 | 0.083 |
| HINT2 | 85 | 8.46E-02 | 3.67E-03 | 7.96E-01 | 3.44E-02 | 0.082 |
| VKORC1 | 51 | 7.40E-01 | 9.44E-03 | 1.68E-01 | 2.07E-03 | 0.080 |
| WDR48 | 96 | 7.41E-01 | 8.59E-02 | 8.67E-02 | 9.97E-03 | 0.076 |
| TATDN3 | 94 | 2.46E-03 | 7.81E-05 | 8.93E-01 | 2.83E-02 | 0.076 |
| FUCA1 | 47 | 7.64E-01 | 7.77E-02 | 7.76E-02 | 7.81E-03 | 0.073 |
| GART | 73 | 1.45E-37 | 1.88E-39 | 9.17E-01 | 1.18E-02 | 0.071 |
| CYB561 | 67 | 8.45E-06 | 1.14E-08 | 9.32E-01 | 1.19E-03 | 0.067 |
| CCDC132 | 113 | 7.70E-01 | 4.05E-03 | 1.59E-01 | 7.69E-04 | 0.066 |
| KCNJ9 | 139 | 8.53E-11 | 8.32E-13 | 9.26E-01 | 8.97E-03 | 0.065 |
| CNTN2 | 158 | 9.80E-02 | 5.30E-04 | 8.33E-01 | 4.44E-03 | 0.065 |
| FAM131B | 94 | 1.22E-03 | 2.60E-06 | 9.33E-01 | 1.92E-03 | 0.064 |
| ARNT2 | 199 | 7.65E-02 | 7.03E-04 | 8.51E-01 | 7.76E-03 | 0.064 |
| SLC44A2 | 95 | 6.84E-02 | 5.56E-01 | 3.44E-02 | 2.80E-01 | 0.061 |
| CNPY4 | 71 | 8.60E-01 | 9.07E-03 | 7.49E-02 | 7.35E-04 | 0.055 |
| DEPTOR | 178 | 6.26E-04 | 9.51E-06 | 9.31E-01 | 1.41E-02 | 0.055 |
| SBF2 | 285 | 8.16E-01 | 3.25E-02 | 9.37E-02 | 3.68E-03 | 0.054 |

| | | | | | | |
|---|---|---|---|---|---|---|
| CPNE4 | 529 | 2.75E-01 | 6.10E-03 | 6.51E-01 | 1.44E-02 | 0.054 |
| LANCL1 | 91 | 4.64E-01 | 2.45E-03 | 4.77E-01 | 2.46E-03 | 0.054 |
| NME7 | 200 | 8.01E-04 | 1.27E-05 | 9.33E-01 | 1.47E-02 | 0.052 |
| SKIV2L | 69 | 1.00E-250 | 7.96E-01 | 1.92E-251 | 1.53E-01 | 0.052 |
| CLIP2 | 77 | 6.11E-03 | 1.81E-05 | 9.39E-01 | 2.73E-03 | 0.052 |
| SMCR8 | 61 | 3.12E-01 | 5.96E-01 | 1.51E-02 | 2.88E-02 | 0.048 |
| PIP5KL1 | 72 | 2.45E-05 | 1.87E-07 | 9.45E-01 | 7.17E-03 | 0.048 |
| GALC | 187 | 5.56E-44 | 1.66E-38 | 3.19E-06 | 9.53E-01 | 0.047 |
| MAPRE1 | 109 | 8.01E-01 | 4.41E-03 | 1.48E-01 | 7.67E-04 | 0.046 |
| TPD52L2 | 79 | 3.73E-01 | 3.38E-01 | 1.28E-01 | 1.16E-01 | 0.044 |
| MUT | 84 | 2.66E-06 | 5.38E-09 | 9.55E-01 | 1.89E-03 | 0.043 |
| PPM1F | 115 | 2.20E-06 | 6.39E-01 | 1.10E-06 | 3.20E-01 | 0.042 |
| CD53 | 98 | 1.65E-01 | 4.75E-04 | 7.91E-01 | 2.24E-03 | 0.041 |
| FYCO1 | 129 | 8.63E-01 | 2.03E-02 | 7.57E-02 | 1.74E-03 | 0.039 |
| ORAI2 | 40 | 7.48E-01 | 3.43E-03 | 2.08E-01 | 9.17E-04 | 0.039 |
| SLC25A12 | 139 | 7.34E-05 | 1.80E-06 | 9.39E-01 | 2.30E-02 | 0.038 |
| GTF3C3 | 62 | 8.61E-01 | 1.02E-02 | 9.16E-02 | 1.05E-03 | 0.037 |
| PSMB9 | 143 | 3.46E-178 | 4.44E-01 | 4.06E-178 | 5.20E-01 | 0.036 |
| SELENBP1 | 58 | 2.86E-09 | 6.93E-12 | 9.62E-01 | 2.30E-03 | 0.035 |
| LRRC16A | 330 | 1.52E-02 | 7.39E-03 | 6.35E-01 | 3.08E-01 | 0.034 |
| SLC25A32 | 94 | 4.52E-01 | 7.68E-04 | 5.15E-01 | 8.44E-04 | 0.031 |
| APIP | 161 | 1.81E-36 | 1.63E-38 | 9.60E-01 | 8.63E-03 | 0.031 |
| IKBKAP | 186 | 1.32E-01 | 4.37E-03 | 8.06E-01 | 2.67E-02 | 0.031 |
| ADA | 90 | 3.91E-10 | 2.35E-12 | 9.63E-01 | 5.75E-03 | 0.031 |
| PYGB | 112 | 1.04E-01 | 1.83E-04 | 8.63E-01 | 1.49E-03 | 0.031 |
| INPP4A | 108 | 1.24E-01 | 4.56E-04 | 8.42E-01 | 3.07E-03 | 0.031 |
| RALB | 69 | 5.72E-01 | 1.49E-03 | 3.97E-01 | 1.00E-03 | 0.029 |
| PDK1 | 115 | 6.98E-01 | 5.79E-03 | 2.66E-01 | 2.18E-03 | 0.028 |
| SLC4A8 | 138 | 5.62E-01 | 4.88E-03 | 4.02E-01 | 3.47E-03 | 0.027 |
| CENPV | 72 | 2.64E-02 | 4.63E-05 | 9.45E-01 | 1.63E-03 | 0.027 |
| MAP1LC3A | 72 | 5.70E-01 | 2.06E-03 | 4.00E-01 | 1.42E-03 | 0.027 |
| PSMD9 | 89 | 1.93E-07 | 6.49E-10 | 9.71E-01 | 3.24E-03 | 0.026 |
| RHOQ | 138 | 8.73E-01 | 1.72E-02 | 8.36E-02 | 1.62E-03 | 0.025 |
| GPC5 | 713 | 7.71E-02 | 1.26E-03 | 8.84E-01 | 1.45E-02 | 0.023 |
| DEPDC5 | 113 | 8.50E-01 | 2.18E-02 | 1.02E-01 | 2.61E-03 | 0.023 |
| DGKA | 44 | 5.57E-01 | 7.95E-04 | 4.19E-01 | 5.75E-04 | 0.023 |
| MCCC1 | 112 | 7.56E-20 | 1.77E-22 | 9.76E-01 | 2.27E-03 | 0.022 |
| SNCG | 54 | 1.84E-04 | 3.62E-07 | 9.77E-01 | 1.91E-03 | 0.020 |
| ARHGAP12 | 119 | 6.13E-01 | 3.38E-03 | 3.61E-01 | 1.97E-03 | 0.020 |

| | | | | | | |
|---|---|---|---|---|---|---|
| NSUN2 | 176 | 5.56E-01 | 1.77E-01 | 1.88E-01 | 5.98E-02 | 0.020 |
| SSH1 | 110 | 8.61E-01 | 1.89E-02 | 9.88E-02 | 2.15E-03 | 0.019 |
| FAIM2 | 104 | 4.62E-01 | 3.00E-03 | 5.12E-01 | 3.30E-03 | 0.019 |
| ASAP2 | 159 | 1.37E-02 | 6.47E-05 | 9.63E-01 | 4.53E-03 | 0.019 |
| UFSP2 | 98 | 8.92E-01 | 4.67E-03 | 8.49E-02 | 4.27E-04 | 0.018 |
| EPM2A | 120 | 7.93E-01 | 1.18E-02 | 1.75E-01 | 2.60E-03 | 0.017 |
| VWA7 | 56 | 1.15E-242 | 2.80E-01 | 2.90E-242 | 7.04E-01 | 0.017 |
| TMTC4 | 123 | 2.38E-01 | 5.84E-04 | 7.43E-01 | 1.80E-03 | 0.017 |
| GOSR2 | 112 | 8.12E-01 | 1.14E-02 | 1.58E-01 | 2.20E-03 | 0.017 |
| OPALIN | 125 | 8.84E-01 | 2.03E-02 | 7.86E-02 | 1.79E-03 | 0.016 |
| AAK1 | 130 | 6.48E-01 | 4.78E-03 | 3.30E-01 | 2.42E-03 | 0.016 |
| GALT | 90 | 7.56E-01 | 8.68E-03 | 2.18E-01 | 2.48E-03 | 0.016 |
| NUBPL | 200 | 5.77E-01 | 2.23E-03 | 4.04E-01 | 1.55E-03 | 0.015 |
| AGPS | 135 | 7.72E-01 | 2.70E-03 | 2.11E-01 | 7.25E-04 | 0.014 |
| FAM118B | 173 | 6.19E-01 | 6.62E-03 | 3.57E-01 | 3.80E-03 | 0.014 |
| NRP1 | 261 | 6.39E-01 | 5.52E-03 | 3.40E-01 | 2.92E-03 | 0.013 |
| TAPT1 | 143 | 9.32E-01 | 9.13E-03 | 4.61E-02 | 4.40E-04 | 0.012 |
| SNAP47 | 95 | 2.83E-04 | 6.14E-07 | 9.87E-01 | 2.13E-03 | 0.011 |
| RDH11 | 96 | 5.34E-01 | 9.30E-04 | 4.54E-01 | 7.80E-04 | 0.011 |
| VAT1L | 349 | 6.93E-01 | 7.92E-03 | 2.86E-01 | 3.25E-03 | 0.010 |
| KLHL3 | 106 | 9.12E-01 | 4.76E-03 | 7.31E-02 | 3.72E-04 | 0.010 |
| RAB40C | 102 | 8.39E-01 | 3.69E-03 | 1.47E-01 | 6.36E-04 | 0.010 |
| PMPCB | 34 | 6.77E-01 | 4.84E-04 | 3.13E-01 | 2.15E-04 | 0.010 |
| OSBPL11 | 110 | 8.99E-01 | 8.53E-03 | 8.20E-02 | 7.69E-04 | 0.010 |
| LRIG1 | 117 | 8.63E-01 | 5.75E-03 | 1.21E-01 | 7.99E-04 | 0.009 |
| MPZL1 | 163 | 8.19E-01 | 4.42E-03 | 1.66E-01 | 8.90E-04 | 0.009 |
| EIF2B5 | 365 | 6.20E-01 | 8.35E-03 | 3.57E-01 | 4.80E-03 | 0.009 |
| TM7SF2 | 87 | 8.78E-01 | 2.54E-03 | 1.11E-01 | 3.12E-04 | 0.008 |
| PAICS | 81 | 5.12E-01 | 1.19E-03 | 4.78E-01 | 1.11E-03 | 0.008 |
| CCS | 64 | 5.33E-01 | 6.44E-04 | 4.58E-01 | 5.46E-04 | 0.008 |
| FAM177A1 | 102 | 9.29E-01 | 2.21E-02 | 4.00E-02 | 9.44E-04 | 0.007 |
| NAGK | 104 | 6.16E-01 | 1.90E-03 | 3.74E-01 | 1.14E-03 | 0.007 |
| IGSF21 | 343 | 8.84E-01 | 7.01E-03 | 1.01E-01 | 7.94E-04 | 0.007 |
| APPL2 | 135 | 9.39E-01 | 2.17E-02 | 3.23E-02 | 7.41E-04 | 0.006 |
| SLC7A6 | 87 | 9.23E-01 | 1.16E-02 | 5.82E-02 | 7.28E-04 | 0.006 |
| NADK2 | 79 | 8.88E-01 | 4.15E-03 | 1.02E-01 | 4.70E-04 | 0.006 |
| STEAP2 | 104 | 9.16E-01 | 4.74E-03 | 7.37E-02 | 3.75E-04 | 0.006 |
| CHI3L1 | 130 | 7.93E-01 | 8.53E-03 | 1.91E-01 | 2.05E-03 | 0.005 |
| PLCD3 | 95 | 8.18E-01 | 9.70E-03 | 1.64E-01 | 1.94E-03 | 0.005 |

| | | | | | | |
|---|---|---|---|---|---|---|
| ECHDC1 | 64 | 9.14E-01 | 2.16E-03 | 7.87E-02 | 1.81E-04 | 0.005 |
| BTN2A1 | 179 | 2.46E-24 | 2.63E-24 | 4.81E-01 | 5.15E-01 | 0.004 |
| NT5C | 63 | 9.13E-01 | 3.63E-03 | 7.96E-02 | 3.13E-04 | 0.004 |
| CYB5R1 | 102 | 9.49E-01 | 2.55E-03 | 4.51E-02 | 1.18E-04 | 0.003 |
| MYO18A | 111 | 9.64E-01 | 9.86E-03 | 2.33E-02 | 2.36E-04 | 0.003 |
| GLRA3 | 201 | 9.07E-01 | 9.12E-03 | 8.07E-02 | 8.09E-04 | 0.003 |
| GDPGP1 | 95 | 1.24E-27 | 4.67E-26 | 2.59E-02 | 9.72E-01 | 0.002 |
| PTK2B | 218 | 9.45E-01 | 1.49E-02 | 3.75E-02 | 5.88E-04 | 0.002 |
| PTRH1 | 79 | 9.22E-01 | 2.14E-03 | 7.32E-02 | 1.68E-04 | 0.002 |
| FLAD1 | 74 | 9.74E-01 | 3.56E-03 | 2.03E-02 | 7.22E-05 | 0.002 |
| LEMD2 | 211 | 3.63E-04 | 1.36E-02 | 2.56E-02 | 9.58E-01 | 0.002 |
| PDXDC1 | 30 | 9.46E-01 | 1.01E-03 | 5.13E-02 | 5.30E-05 | 0.002 |
| TMEM179B | 58 | 9.75E-01 | 2.08E-03 | 2.16E-02 | 4.50E-05 | 0.001 |
| DDAH1 | 254 | 1.11E-06 | 5.79E-03 | 1.91E-04 | 9.93E-01 | 0.001 |
| TMEM150C | 133 | 9.63E-01 | 2.65E-03 | 3.35E-02 | 9.16E-05 | 0.001 |
| HOOK1 | 112 | 9.63E-01 | 2.37E-03 | 3.38E-02 | 8.25E-05 | 0.001 |
| ARHGEF25 | 83 | 3.64E-08 | 2.61E-03 | 1.39E-05 | 9.97E-01 | 0.000 |
| PANK4 | 95 | 8.02E-14 | 9.16E-04 | 8.75E-11 | 9.99E-01 | 0.000 |
| ALDH16A1 | 77 | 3.13E-21 | 3.28E-17 | 9.53E-05 | 1.00E+00 | 0.000 |
| DDAH2 | 40 | 8.90E-180 | 4.18E-13 | 2.13E-167 | 1.00E+00 | 0.000 |
| SEPTIN4 | NA* | | | | | |

pQTL: protein quantitative trait loci; PP.H0.abf: posterior probability of hypothesis 0 (neither trait of protein quantitative nor

**Table S7. Evaluating posterior probability in different colocalization approaches**

| Gene | PP.H4 via Coloc | PP via HyPrColoc |
|---|---|---|
| **Plasma** | | |
| CD59 | 0.817 | 0.807 |
| FCRL3 | 0.941 | 0.967 |
| CR1 | 0.834 | 0.858 |
| IDUA | 0.816 | 0.811 |
| PGLYRP1 | 0.801 | 0.827 |
| ARHGAP1 | 0.915 | 0.938 |
| TNFRSF1A | 1.000 | 1.000 |
| WARS | 0.770 | 0.804 |
| TYMP | 0.873 | 0.926 |
| **Brain** | | |
| HLA-B | 0.979 | 0.998 |
| ICA1L | 0.958 | 0.957 |
| HMGCL | 0.860 | 0.867 |
| FAM120B | 0.957 | 0.961 |
| AUH | 0.753 | **0.737** |
| TSFM | 0.960 | 0.977 |
| ZC2HC1A | 0.988 | 0.993 |
| TRAF3 | 0.983 | 0.989 |
| MTHFR | 0.877 | 0.869 |

PP.H4: posterior probability of hypothesis 4 in coloc analysis (both traits of protein quantitative and multiple sclerosis are associated and share a single causal variant). PP: posterior probability in HyPrColoc analysis with hypothesis of both traits of protein quantitative and multiple sclerosis are associated and share a single causal variant.

**Table S8. SMR analysis identifying gene expression of potential causal proteins in plasma, through integrating eQTL and MS GWAS**

| ProbeID | ProbeChr | Gene | Probe_bp | TopSNP | TopSNP_chr | TopSNP_bp | A1 | A2 | Freq | Beta_GWAS | SE_GWAS | P_GWAS | Beta_eQTL | SE_eQTL | P_eQTL | Beta_SMR | SE_SMR | OR_SMR | 95%CI_SMR | P_SMR | FDR_adjusted_P_SMR | P_HEIDI | $N_{snp}$_HEIDI |
|---|---|---|---|---|---|---|---|---|---|---|---|---|---|---|---|---|---|---|---|---|---|---|---|
| ILMN_1797428 | 1 | FCRL3 | 157648068 | rs3761959 | 1 | 157669278 | T | C | 0.473 | -0.094 | 0.016 | 1.03E-08 | 0.798 | 0.028 | 1.58E-179 | -0.118 | 0.021 | 0.889 | (0.85-0.93) | 1.98E-08 | 2.57E-07 | 7.10E-02 | 20 |
| ILMN_1699599 | 1 | FCRL3 | 157648482 | rs3761959 | 1 | 157669278 | T | C | 0.473 | -0.094 | 0.016 | 1.03E-08 | 0.664 | 0.028 | 1.37E-122 | -0.142 | 0.026 | 0.868 | (0.83-0.91) | 2.65E-08 | 1.72E-07 | 6.83E-02 | 20 |
| ILMN_1704870 | 19 | PGLYRP1 | 46522541 | rs2072563 | 19 | 46526648 | A | G | 0.274 | 0.070 | 0.018 | 1.34E-04 | -0.769 | 0.030 | 3.21E-142 | -0.091 | 0.024 | 0.913 | (0.87-0.96) | 1.59E-04 | 1.72E-07 | 3.88E-01 | 20 |
| ILMN_1727271 | 14 | WARS | 100800398 | rs1570305 | 14 | 100808155 | G | A | 0.189 | -0.068 | 0.020 | 5.67E-04 | 1.037 | 0.032 | 1.08E-226 | -0.066 | 0.019 | 0.936 | (0.9-0.97) | 6.09E-04 | 6.89E-04 | 7.85E-02 | 20 |
| ILMN_2337655 | 14 | WARS | 100800252 | rs1570305 | 14 | 100808155 | G | A | 0.189 | -0.068 | 0.020 | 5.67E-04 | 1.016 | 0.032 | 4.62E-218 | -0.067 | 0.020 | 0.935 | (0.9-0.97) | 6.11E-04 | 1.59E-03 | 1.24E-01 | 20 |
| ILMN_1685005 | 12 | TNFRSF1A | 6438212 | rs1800692 | 12 | 6442346 | A | G | 0.368 | -0.061 | 0.018 | 5.72E-04 | 0.279 | 0.028 | 6.03E-23 | -0.220 | 0.068 | 0.803 | (0.7-0.92) | 1.15E-03 | 1.59E-03 | 7.98E-05 | 9 |
| ILMN_1703041 | 4 | IDUA | 997408 | rs13101828 | 4 | 965720 | G | A | 0.447 | -0.050 | 0.017 | 2.78E-03 | 0.458 | 0.028 | 1.40E-60 | -0.110 | 0.037 | 0.896 | (0.83-0.96) | 3.25E-03 | 2.48E-03 | 1.67E-01 | 20 |
| ILMN_2388112 | 1 | CR1 | 207812977 | rs41274768 | 1 | 207782769 | A | G | 0.032 | -0.115 | 0.050 | 2.07E-02 | -0.466 | 0.082 | 1.55E-08 | 0.247 | 0.115 | 1.280 | (1.02-1.6) | 3.22E-02 | 6.04E-03 | 1.27E-01 | 3 |
| ILMN_1701621 | 22 | TYMP | 50962152 | rs73172252 | 22 | 50973779 | A | G | 0.140 | 0.049 | 0.024 | 4.15E-02 | -0.530 | 0.038 | 4.05E-45 | -0.092 | 0.045 | 0.912 | (0.83-1) | 4.37E-02 | 5.24E-02 | 5.19E-01 | 3 |
| ILMN_1690939 | 22 | TYMP | 50965032 | rs73172252 | 22 | 50973779 | A | G | 0.140 | 0.049 | 0.024 | 4.15E-02 | -0.347 | 0.038 | 2.13E-20 | -0.140 | 0.070 | 0.870 | (0.76-1) | 4.65E-02 | 6.05E-02 | NA | NA |
| ILMN_1742601 | 1 | CR1 | 207793387 | rs11581832 | 1 | 207804238 | C | A | 0.032 | -0.105 | 0.050 | 3.44E-02 | -0.462 | 0.083 | 2.21E-08 | 0.227 | 0.115 | 1.255 | (1-1.57) | 4.78E-02 | 5.65E-02 | 2.25E-03 | 9 |
| ILMN_2109708 | 22 | TYMP | 50964207 | rs73172252 | 22 | 50973779 | A | G | 0.140 | 0.049 | 0.024 | 4.15E-02 | -0.312 | 0.038 | 1.55E-16 | -0.156 | 0.079 | 0.856 | (0.73-1) | 4.78E-02 | 5.18E-02 | NA | NA |
| ILMN_1711611 | 1 | CR1 | 207679280 | rs34860402 | 1 | 207856009 | G | A | 0.108 | 0.010 | 0.026 | 6.90E-01 | -0.579 | 0.044 | 1.07E-39 | -0.018 | 0.045 | 0.982 | (0.9-1.07) | 6.90E-01 | 5.18E-02 | 2.18E-02 | 20 |
| NA | NA | CD59 | NA | NA | NA | NA | NA | NA | NA | NA | NA | NA | NA | NA | NA | NA | NA | NA | NA | NA | NA | NA | NA |
| NA | NA | ARHGAP1 | NA | NA | NA | NA | NA | NA | NA | NA | NA | NA | NA | NA | NA | NA | NA | NA | NA | NA | NA | NA | NA |

eQTL: expression quantitative trait loci; GWAS: genome-wide association study; SMR: summary-data-based Mendelian randomization; Chr: chromosome; bp: base pair; SNP: single nucleotide polymorphism; A1: the effect allele; A2: the other allele; Freq: frequency of the effect allele; SE: standard error; HEIDI: heterogeneity in dependen

**Table S9. SMR analysis examining if both gene expression and protein expression in plasma are causally affected by a single variant, through integrating eQTL and plasma pQTL**

| Exposure_ID | Exposure_chromosome | Exposure_Gene | Exposure_position | Outcome_ID | Outcome_chromosome | Outcome_Gene | Outcome_position | TopSNP | TopSNP_chromosome | TopSNP_position | A1 | A2 | Freq | Beta_Outcome | SE_Outcome | P_Outcome | Beta_Exposure | SE_Exposure | P_Exposure | Beta_SMR | SE_SMR | OR_SMR | 95%CI_SMR | P_SMR | FDR_adjusted_P_SMR | P_HEIDI | $N_{snp}$_HEIDI |
|---|---|---|---|---|---|---|---|---|---|---|---|---|---|---|---|---|---|---|---|---|---|---|---|---|---|---|---|
| ILMN_1797428 | 1 | FCRL3 | 157648068 | SeqId_4440_15 | 1 | FCRL3 | 157255396 | rs3761959 | 1 | 157669278 | T | C | 0.473 | 0.712 | 0.019 | 1.04E-300 | 0.798 | 0.028 | 1.58E-179 | 0.891 | 0.039 | 2.438 | (2.26-2.63) | 2.30E-113 | 1.61E-112 | 2.91E-04 | 20 |
| ILMN_1699599 | 1 | FCRL3 | 157648482 | SeqId_4440_15 | 1 | FCRL3 | 157255396 | rs3761959 | 1 | 157669278 | T | C | 0.473 | 0.712 | 0.019 | 1.04E-300 | 0.664 | 0.028 | 1.37E-122 | 1.072 | 0.054 | 2.921 | (2.63-3.25) | 6.72E-88 | 1.61E-112 | 2.37E-03 | 20 |
| ILMN_1703041 | 4 | IDUA | 997408 | SeqId_3169_70 | 4 | IDUA | 1357325 | rs13101828 | 4 | 965720 | G | A | 0.447 | 0.497 | 0.016 | 2.24E-206 | 0.458 | 0.028 | 1.40E-60 | 1.085 | 0.075 | 2.960 | (2.56-3.43) | 1.78E-47 | 2.35E-87 | 1.59E-05 | 20 |
| ILMN_1704870 | 19 | PGLYRP1 | 46522541 | SeqId_3329_14 | 19 | PGLYRP1 | 46728857 | rs2072563 | 19 | 46526648 | A | G | 0.274 | -0.214 | 0.018 | 6.66E-33 | -0.769 | 0.030 | 3.21E-142 | 0.278 | 0.026 | 1.321 | (1.26-1.39) | 3.06E-27 | 4.15E-47 | 5.19E-04 | 20 |
| ILMN_2388112 | 1 | CR1 | 207812977 | SeqId_19556_12 | 1 | CR1 | 208129798 | rs115126815 | 1 | 207828971 | T | C | 0.0143149 | -0.580 | 0.058 | 0.000 | 0.630 | 0.099 | 1.98E-10 | -0.920 | 0.171 | 0.398 | (0.28-0.56) | 7.62E-08 | 5.36E-27 | 4.12E-02 | 5 |
| ILMN_1742601 | 1 | CR1 | 207793387 | SeqId_19556_12 | 1 | CR1 | 208129798 | rs11581832 | 1 | 207804238 | C | A | 0.0316973 | -0.342 | 0.048 | 0.000 | -0.462 | 0.083 | 2.21E-08 | 0.740 | 0.168 | 2.095 | (1.51-2.91) | 1.01E-05 | 1.07E-07 | 1.69E-07 | 12 |
| ILMN_1701621 | 22 | TYMP | 50962152 | SeqId_11361_73 | 22 | TYMP | 51138753 | rs73172252 | 22 | 50973779 | A | G | 0.140082 | 0.005 | 0.023 | 0.843 | -0.530 | 0.038 | 4.05E-45 | -0.009 | 0.043 | 0.991 | (0.91-1.08) | 8.43E-01 | 1.18E-05 | 9.92E-01 | 3 |

eQTL: expression quantitative trait loci; pQTL:protein quantitative trait loci; SMR: summary-data-based Mendelian randomization; Chr: chromosome; SNP: single nucleotide polymorphism; A1: the effect allele; A2: the other allele; Freq: frequency of the effect allele; SE: standard error; HEIDI: heterogeneity in dependent instruments test; Benjamini-Hochberg method was used in FDR

**Table S10. SMR analysis identifying gene expression of potential causal proteins in brain, through integrating eQTL and MS GWAS**

| ProbeID | Probe Chr | Gene | Probe_bp | TopSNP | TopSNP_chr | TopSNP_bp | A1 | A2 | Freq | Beta_GWAS | SE_GWAS | P_GWAS | Beta_eQTL | SE_eQTL | P_eQTL | Beta_SMR | SE_SMR | OR_SMR | 95%CI_SMR | P_SMR | FDR_adjusted_P_SMR | P_HEIDI | $N_{snp}$_HEIDI |
|---|---|---|---|---|---|---|---|---|---|---|---|---|---|---|---|---|---|---|---|---|---|---|---|
| ENSG00000123297.20 | 12 | TSFM | 58189199 | rs10877012 | 12 | 58162085 | T | G | 0.316 | -0.115 | 0.018 | 7.60E-11 | -0.899 | 0.033 | 1.94E-168 | 0.128 | 0.020 | 1.136 | (1.09-1.18) | 2.37E-10 | 1.89E-09 | 2.73E-02 | 20 |
| ENSG00000234745.13 | 6 | HLA-B | 31323307 | rs12196740 | 6 | 31321407 | T | C | 0.094 | -0.423 | 0.051 | 1.79E-16 | 0.594 | 0.069 | 7.33E-18 | -0.713 | 0.120 | 0.490 | (0.39-0.62) | 2.66E-09 | 1.89E-09 | 3.29E-02 | 20 |
| ENSG00000131323.16 | 14 | TRAF3 | 103310812 | rs7145882 | 14 | 103255461 | T | C | 0.325 | 0.096 | 0.017 | 2.15E-08 | 0.393 | 0.035 | 6.47E-29 | 0.245 | 0.049 | 1.277 | (1.16-1.41) | 5.60E-07 | 1.06E-08 | 5.46E-02 | 20 |
| ENSG00000163596.17 | 2 | ICA1L | 203687181 | rs72932772 | 2 | 203682304 | G | C | 0.130 | -0.098 | 0.025 | 7.97E-05 | -0.726 | 0.044 | 5.26E-60 | 0.135 | 0.035 | 1.144 | (1.07-1.23) | 1.26E-04 | 1.49E-06 | 1.18E-01 | 20 |
| ENSG00000117305.15 | 1 | HMGCL | 24146742 | rs2076343 | 1 | 24129126 | G | A | 0.329 | 0.065 | 0.017 | 1.64E-04 | 0.639 | 0.031 | 1.22E-91 | 0.102 | 0.027 | 1.107 | (1.05-1.17) | 2.11E-04 | 2.51E-04 | 2.47E-01 | 20 |
| ENSG00000148090.12 | 9 | AUH | 94050148 | rs2482338 | 9 | 94037873 | C | T | 0.370 | -0.048 | 0.017 | 0.005398 | 0.393 | 0.030 | 4.11E-38 | -0.121 | 0.044 | 0.886 | (0.81-0.97) | 6.53E-03 | 3.37E-04 | 2.97E-01 | 20 |
| ENSG00000112584.14 | 6 | FAM120B | 170657973 | rs9459991 | 6 | 170641014 | A | G | 0.131 | 0.036 | 0.026 | 0.162 | 0.577 | 0.045 | 6.98E-37 | 0.063 | 0.045 | 1.065 | (0.97-1.16) | 1.65E-01 | 8.71E-03 | 3.44E-01 | 20 |
| ENSG00000177000.13 | 1 | MTHFR | 11856146 | rs1476413 | 1 | 11852300 | T | C | 0.277 | -0.009 | 0.018 | 0.6186 | 0.595 | 0.031 | 6.21E-83 | -0.015 | 0.031 | 0.985 | (0.93-1.05) | 6.19E-01 | 1.88E-01 | 1.79E-02 | 20 |
| NA | 8 | ZC2HC1A | NA | NA | NA | NA | NA | NA | NA | NA | NA | NA | NA | NA | NA | NA | NA | NA | NA | NA | NA | NA | NA |

eQTL: expression quantitative trait loci; GWAS: genome-wide association study; SMR: summary-data-based Mendelian randomization; Chr: chromosome; bp: base pair; SNP: single nucleotide polymorphism; A1: the effect allele; A2: the other allele; Freq: frequency of the effect allele; SE: standard error; HEIDI:

**Table S11. SMR analysis examining if both gene expression and protein expression in brain are causally affected by a single variant, through Integrating eQTL and brain pQTL**

| Exposure_ID | Exposure_chromosome | Exposure_Gene | Exposure_position | Outcome_ID | Outcome_chromosome | Outcome_Gene | Outcome_position | TopSNP | TopSNP_chromosome | TopSNP_position | A1 | A2 | Freq | Beta_Outcome | SE_Outcome | P_Outcome | Beta_Exposure | SE_Exposure | P_Exposure | Beta_SMR | SE_SMR | OR_SMR | 95%CI_SMR | P_SMR | FDR_adjusted_P_SMR | P_HEIDI | $N_{snp}$_HEIDI |
|---|---|---|---|---|---|---|---|---|---|---|---|---|---|---|---|---|---|---|---|---|---|---|---|---|---|---|---|
| ENSG00000117305.15 | 1 | HMGCL | 24146742 | ENSG00000117305 | 1 | HMGCL | 24133612 | rs2076343 | 1 | 24129126 | G | A | 0.329 | 0.051 | 6.86E-03 | 0.000 | 0.639 | 3.15E-02 | 1.22E-91 | 0.079 | 1.14E-02 | 1.082 | (1.06-1.11) | 4.30E-12 | 1.29E-11 | 5.54E-01 | 13 |
| ENSG00000148090.12 | 9 | AUH | 94050148 | ENSG00000148090 | 9 | AUH | 93936592 | rs2482338 | 9 | 94037873 | C | T | 0.370 | 0.039 | 6.21E-03 | 0.000 | 0.393 | 3.05E-02 | 4.11E-38 | 0.099 | 1.75E-02 | 1.104 | (1.07-1.14) | 1.93E-08 | 2.89E-08 | 1.77E-03 | 20 |
| ENSG00000131323.16 | 14 | TRAF3 | 103310812 | ENSG00000131323 | 14 | TRAF3 | 103404159 | rs7145882 | 14 | 103255461 | T | C | 0.325 | 0.030 | 6.03E-03 | 0.000 | 0.393 | 3.52E-02 | 6.47E-29 | 0.077 | 1.68E-02 | 1.080 | (1.05-1.12) | 4.76E-06 | 4.76E-06 | 9.62E-01 | 19 |

eQTL: expression quantitative trait loci; pQTL:protein quantitative trait loci; SMR: summary-data-based Mendelian randomization; Chr: chromosome; SNP: single nucleotide polymorphism; A1: the effect allele; A2: the other allele; Freq: frequency of the effect allele; SE: standard error; HEIDI: heterogeneity in dependent instruments test; Benjamini-Hochberg method was used in FDR

**Table S12. Single cell-specific differential gene expression of prioritized proteins**

| Cell type | Enesmbl ID | Gene symbols | Average log2 fold change | P value | FDR-adjusted P value |
|---|---|---|---|---|---|
| **Blood*** | | | | | |
| CD4 T cells | ENSG00000175220 | ARHGAP1 | -0.11 | 2.73E-216 | 1.50E-10 |
| CD4 T cells | ENSG00000067182 | TNFRSF1A | -0.35 | 2.88E-183 | 2.66E-02 |
| Granulocytes | ENSG00000067182 | TNFRSF1A | -0.65 | 7.41E-33 | 2.04E-05 |
| CD8a T cells | ENSG00000067182 | TNFRSF1A | -0.36 | 1.49E-97 | 1.08E-09 |
| **Brain** | | | | | |
| Oligodendrocyte | ENSG00000148090 | AUH | -0.99 | 2.64E-10 | 3.65E-10 |
| Oligodendrocyte precursor cell | ENSG00000148090 | AUH | -3.83 | 1.95E-06 | 2.58E-05 |
| Oligodendrocyte precursor cell | ENSG00000163596 | ICA1L | -1.1 | 2.03E-03 | 5.69E-03 |
| Astrocyte | ENSG00000148090 | AUH | -1.49 | 2.10E-05 | 5.70E-05 |
| Endothelial cell | ENSG00000148090 | AUH | -1.35 | 1.00E-03 | 2.20E-03 |
| Neuron | ENSG00000148090 | AUH | -1.66 | 9.64E-14 | 2.12E-13 |
| Neuron | ENSG00000163596 | ICA1L | -0.43 | 6.14E-06 | 7.59E-06 |

* The differential expression analysis of blood was performed by a published study (PMID: 31937773), P value was

**Table S13. For all detected potential causal proteins in plasma: pathway annotation, protein-targeted drugs, and evidence in previous studies**

| Gene | Ensembl_ID | Protein | UniProt | KEGG ID | Central dogma | Known MS drug target | Link with known MS drug traget | Pathway | Protein-targeted drug | General function of the identified drug | Protein (gene)-MS relations suggested by previous studies | DOI |
|---|---|---|---|---|---|---|---|---|---|---|---|---|
| ARHGAP1 | ENSG00000175220 | RHG01 | Q07960 | hsa:392 | no | no | no | - | - | - | - | - |
| PGLYRP1 | ENSG00000008438 | PGRP1 | O75594 | hsa:8993 | no | no | no | - | - | - | PGLYRP1 is suggested to serve as a proinflammatory molecule in myeloid cells during autoimmune conditions. These findings shed light on the potential of PGLYRP1 as a target for immunotherapy. Targeting PGLYRP1 could trigger a robust antitumor immune response while also offering protection against certain types of tissue inflammation and autoimmune reactions.<br>The PGLYRP1 protein shows expression in cerebellar and spinal cord tissues of patients with MS. Immunofluorescence analysis revealed elevated levels of PGLYRP1 protein in both the white and gray matter of the cerebellum and spinal cord in patients with MS. Immunoblot analysis of spinal cord tissue from patients with MS confirmed the upregulation of PGLYRP1 protein, consistent with the findings from immunofluorescence analysis. Conversely, no significant immunoreactivity of PGLYRP1 protein was observed in the controls. | https://doi.org/10.1038/s41590-023-01645-4; https://doi.org/10.1016/j.celrep.2024.113813 |
| CR1 | ENSG00000203710 | Complement receptor type 1 | P17927 | hsa:1378 | no | no | yes | Complement and coagulation cascades; Neutrophil extracellular trap formation; Hematopoietic cell lineage; Legionellosis; Leishmaniasis; Malaria; Tuberculosis | - | - | CR1-positive immunolabeled cells were significantly increased in white matter lesions tissue compared with normal appearing white matter. | https://doi.org/10.1111/bpa.12546 |
| WARS | ENSG00000140105 | SYWC | P23381 | hsa:7453 | no | no | no | Aminoacyl-tRNA biosynthesis | Tryptophan (DB00150), Tryptophanyl-5'amp (DB01831), Tryptophanamide (DB04537) | Tryptophan is an amino acid commonly found as a component of total parenteral nutrition. | - | - |
| IDUA | ENSG00000127415 | IDUA | P35475 | hsa:3425 | no | no | no | Glycosaminoglycan degradation; Metabolic pathways; Lysosome | Laronidase (DB00090) | Laronidase is a form of recombinant human alpha-L-iduronidase used to treat Hurler and Hurler-Scheie forms of mucopolysaccharidosis, a genetic deficiency of alpha-L-iduronidase. | IDUA protein associated with MS progression. | https://doi.org/10.1101/2022.07.29.22278211 |
| CD59 | ENSG00000085063 | CD59 | P13987 | hsa:966 | no | no | yes | Complement and coagulation cascades; Hematopoietic cell lineage | - | - | The CSF levels of CD59 were upregulated in NMO and MS patients. The CSF CD59 levels decreased after treatment. Elevated CSF CD59 levels may reflect complement activation within CNS. https://doi.org/10.1016/j.cca.2015.12.013 The deficiency of CD59 is one of factor that exacerbates MS disease, as demonstrated by MS experimental animals with CD55 and CD59 gene deficiencies, which resulted in more severe rates of MS disease. | https://doi.org/10.1016/j.cca.2015.12.013; https://doi.org/10.3389/fimmu.2022.970486 |
| TNFRSF1A | ENSG00000067182 | TNR1A | P19438 | hsa:7132 | no | no | yes | MAPK signaling pathway; Cytokine-cytokine receptor interaction; Viral protein interaction with cytokine and cytokine receptor; NF-kappa B signaling pathway; Sphingolipid signaling pathway; mTOR signaling pathway; Apoptosis; Apoptosis - multiple species; Necroptosis; Osteoclast differentiation; TNF signaling pathway; Adipocytokine signaling pathway; Insulin resistance; Non-alcoholic fatty liver disease; Alcoholic liver disease; Alzheimer disease; Amyotrophic lateral sclerosis; Pathways of neurodegeneration - multiple diseases; Pathogenic Escherichia coli infection; Shigellosis; Salmonella infection; Chagas disease; Toxoplasmosis; Tuberculosis; Hepatitis C; Human cytomegalovirus infection; Influenza A; Human papillomavirus infection; Human T-cell leukemia virus 1 infection; Kaposi sarcoma-associated herpesvirus infection; Herpes simplex virus 1 infection; Human immunodeficiency virus 1 infection; Coronavirus disease - COVID-19; Lipid and atherosclerosis; Fluid shear stress and atherosclerosis | 6-[3-(4-Morpholinyl)Propyl]-2-(3-Nitrophenyl)-5-Thioxo-5,6,-Dihydro-7h-Thienol[2',3':4,5]Pyrrolo[1,2-C]Imidazol-7-One (DB03507), Tasonermin (DB11626) | Tasonermin is a tumor necrosis factor alpha used along with surgery to remove soft tissue sarcomas of the limbs. Tasonermin is a tumor necrosis factor alpha used along with surgery to remove soft tissue sarcomas of the limbs. | A polymorphism associated with an elevated risk of developing MS has been identified in the TNFRSF1A gene, marking TNFRSF1A as a novel susceptibility locus for multiple sclerosis. | https://doi.org/10.1212/01.wnl.0000436612.66328.8a; https://doi.org/10.1212/WNL.0b013e318294b2d6 |
| FCRL3 | ENSG00000160856 | FCRL3 | Q96P31 | hsa:115352 | no | no | yes | - | - | - | FCRL3 can activate the SHP-1 and p38 MAPK pathways and then promote the secretion of IL-10 in B cells, thus inhibiting the secretion of inflammatory factors. Therefore, FCRL3 may play an immunoprotective role in MS, and it will be an effective target for the diagnosis and treatment of MS. TLR9 activation impacts various aspects of B cell function, including proliferation, apoptosis, antibody production, and IL-10 secretion, through the upregulation of FCRL3 expression. Consequently, FCRL3 emerges as a significant target for both diagnosis and treatment of B cell-related diseases. Studies found that FCRL3 variants were associated with MS | https://doi.org/10.1002/cbin.11373；https://doi.org/10.1007/s11626-022-00720-8 |
| TYMP | ENSG00000025708 | TP | P19971 | hsa:1890 | no | no | yes | Pyrimidine metabolism; Drug metabolism - other enzymes; Metabolic pathways; Nucleotide metabolism; Bladder cancer | Tipiracil (DB09343), Capecitabine (DB01101), Floxuridine (DB00322), Fluorouracil (DB00544), Trifluridine (DB00432), Cidofovir (DB00369), Tezacitabine (DB06433) | Tipiracil is a thymidine phosphorylase inhibitor used as an adjunct treatment of adult patients with certain types of gastric or colorectal malignancies. Floxuridine is an antimetabolite used as palliative management for liver metastases of gastrointestinal malignancy.<br>Fluorouracil is a pyrimidine analog used to treat basal cell carcinomas, and as an injection in palliative cancer treatment. Trifluridine is a nucleoside metabolic inhibitor used to treat keratoconjunctivitis and epithelial keratitis caused by simplex virus, and as a part of chemotherapy for certain types of metastatic gastrointestinal cancers. Cidofovir is an antiviral agent used to treat Cytomegalovirus (CMV) retinitis in patients with AIDS. | The gene TYMP encoding for the protein tymidine phosphorylase was found downregulated from MS. TYMP was reported as the second key astrocyte-derived permeability factor that, via Vascular Endothelial Growth Factor A (VEGFA) interaction, plays an essential role in the blood-brain-barrier disruption observed in Experimental Autoimmune Encephalomyelitis lesions, the animal model of MS<br>Chapouly C, Tadesse Argaw A, Horng S, Castro K, Zhang J, Asp L, et al. Astrocytic TYMP and VEGFA drive blood-brain barrier opening in inflammatory central nervous system lesions. Brain. 2015; 138: 1548–1567. | https://doi.org/10.31083/j.jin2201004; https://doi.org/10.1093/brain/awv077 |

**Table S14. Protein-protein interaction analysis of all detected potential causal proteins in plasma with targets of current multiple sclerosis drugs (minimum combined score of 0**

| MS drug | MS drug target | armacological action of MS | Potential target | ood_on_ch | gene_fusion | netic_coocc | homology | coexpressio | y_determin | base_annot | nated_textn | combined_score |
|---|---|---|---|---|---|---|---|---|---|---|---|---|
| Alemtuzumab | FCGR2A | Unknown | CR1 | 0 | 0 | 0 | 0 | 0.261 | 0 | 0 | 0.729 | 0.791 |
| Alemtuzumab | FCGR2B | Unknown | CR1 | 0 | 0 | 0 | 0 | 0.218 | 0 | 0 | 0.711 | 0.765 |
| Alemtuzumab | FCGR3A | Unknown | CR1 | 0 | 0 | 0 | 0 | 0.123 | 0 | 0 | 0.720 | 0.744 |
| Alemtuzumab | FCGR3B | Unknown | CR1 | 0 | 0 | 0 | 0 | 0.142 | 0 | 0 | 0.723 | 0.752 |
| Cladribine | PNP | Inhibitor | TYMP | 0.120 | 0 | 0 | 0 | 0.042 | 0 | 0.900 | 0.642 | 0.965 |
| Cladribine | RRM2B | Inhibitor | TYMP | 0 | 0 | 0 | 0 | 0.042 | 0 | 0 | 0.798 | 0.798 |
| Daclizumab | FCGR2A | Unknown | CR1 | 0 | 0 | 0 | 0 | 0.261 | 0 | 0 | 0.729 | 0.791 |
| Daclizumab | FCGR2B | Unknown | CR1 | 0 | 0 | 0 | 0 | 0.218 | 0 | 0 | 0.711 | 0.765 |
| Daclizumab | FCGR3A | Unknown | CR1 | 0 | 0 | 0 | 0 | 0.123 | 0 | 0 | 0.720 | 0.744 |
| Daclizumab | FCGR3B | Unknown | CR1 | 0 | 0 | 0 | 0 | 0.142 | 0 | 0 | 0.723 | 0.752 |
| Daclizumab | IL2RA | Antibody | TNFRSF1A | 0 | 0 | 0 | 0 | 0 | 0 | 0 | 0.763 | 0.763 |
| Dimethyl fumarate | RELA | Unknown | TNFRSF1A | 0 | 0 | 0 | 0 | 0.115 | 0 | 0 | 0.811 | 0.826 |
| Natalizumab | FCGR3B | Unknown | CR1 | 0 | 0 | 0 | 0 | 0.142 | 0 | 0 | 0.723 | 0.752 |
| Natalizumab | ICAM1 | Unknown | TNFRSF1A | 0 | 0 | 0 | 0 | 0.066 | 0 | 0 | 0.736 | 0.742 |
| Ocrelizumab | MS4A1 | Antibody | FCRL3 | 0 | 0 | 0 | 0 | 0.511 | 0.173 | 0 | 0.317 | 0.700 |
| Ofatumumab | MS4A1 | Antibody | FCRL3 | 0 | 0 | 0 | 0 | 0.511 | 0.173 | 0 | 0.317 | 0.700 |
| Teriflunomide | DHODH | Inhibitor | TYMP | 0.06 | 0 | 0 | 0 | 0 | 0 | 0.792 | 0.363 | 0.864 |
| Ublituximab | MS4A1 | Binder; Antibody | FCRL3 | 0 | 0 | 0 | 0 | 0.511 | 0.173 | 0 | 0.317 | 0.700 |

**Table S15. For all detected potential causal proteins in brain: pathway annotation, protein-targeted drugs, and evidence in previous studies**

| Gene | Ensembl_ID | Protein | UniProt | KEGG ID | Central dogma | Known MS drug target | Link with known MS drug traget | Pathway | Protein-targeted drug | General function of the identified drug | Protein (gene)-MS relations suggested by previous studies | DOI |
|---|---|---|---|---|---|---|---|---|---|---|---|---|
| HLA-B | ENSG00000234745 | HLA class I histocompatibility antigen, B alpha chain | Q31612 | hsa:3106 | no | no | yes | Endocytosis; Phagosome; Cellular senescence; Cell adhesion molecules; Antigen processing and presentation; Natural killer cell mediated cytotoxicity; Type I diabetes mellitus; Human cytomegalovirus infection; Human papillomavirus infection; Human T-cell leukemia virus 1 infection; Kaposi sarcoma-associated herpesvirus infection; Herpes simplex virus 1 infection; Epstein-Barr virus infection; Human immunodeficiency virus 1 infection; Viral carcinogenesis; Autoimmune thyroid disease; Allograft rejection; Graft-versus-host disease; Viral myocarditis | - | - | The HLA-B*44:02 allele, known to confer protective effects against multiple sclerosis (MS) susceptibility, was associated with improved MRI outcomes, including better brain parenchymal fraction and reduced T2 hyperintense lesion volume. Additionally, this allele (with frequencies of 4.4% in women with MS and 6.9% in control women) was nominally linked to the preservation of subcortical gray matter fraction. | https://doi.org/10.1212/WNL.0b013e3181ed9c9c; https://doi.org/10.1001/jamaneurol.2016.0980 |
| TSFM | ENSG00000123297 | Elongation factor Ts, mitochondrial | P43897 | hsa:10102 | no | no | no | - | - | - | The probe set corresponding to exon 4 of TSFM showed significantly higher levels in healthy controls compared to patients with MS. | https://doi.org/10.3389/fimmu.2022.931831 |
| ZC2HC1A | ENSG00000104427 | Zinc finger C2HC domain-containing | Q96GY0 | hsa:51101 | no | no | no | - | - | - | the eQTL SMR tests identified that ZC2HC1A was significantly associated with MS | https://doi.org/10.1007/s00415-019-09476-w |
| TRAF3 | ENSG00000131323 | TNF receptor-associated factor 3 | Q13114 | hsa:7187 | yes | no | yes | NF-kappa B signaling pathway; Toll-like receptor signaling pathway; NOD-like receptor signaling pathway; RIG-I-like receptor signaling pathway; IL-17 signaling pathway; TNF signaling pathway; Alcoholic liver disease; Hepatitis C; Hepatitis B; Measles; Influenza A; Human papillomavirus infection; Kaposi sarcoma-associated herpesvirus infection; Herpes simplex virus 1 infection; Epstein-Barr virus infection; Coronavirus disease - COVID-19; Pathways in cancer; Viral carcinogenesis; Small cell lung cancer; Lipid and atherosclerosis | - | - | Peli1 promotes microglia-mediated CNS inflammation by regulating Traf3 degradation. TRAF3 haploinsufficiency syndrome exhibited B cell hyperactivity leading to hypergammaglobulinemia and autoimmunity | https://doi.org/10.1038/nm.3111; https://doi.org/10.1126/sciimmunol.abn3800 |
| ICA1L | ENSG00000163596 | Islet cell autoantigen 1-like protein | Q8NDH6 | hsa:130026 | no | no | no | - | - | - | - | - |
| MTHFR | ENSG00000177000 | Methylenetetrahydrofolate reductase (NADPH) | P42898 | hsa:4524 | no | no | no | One carbon pool by folate; Metabolic pathways; Antifolate resistance | Cyanocobalamin(Vitamin B12, DB00115), Tetrahydrofolic acid (DB00116) | Vitamin B12 is a vitamin available in many formulations to correct vitamin B12 deficiency. Tetrahydrofolic acid is for nutritional supplementation, also for treating dietary shortage or imbalance. | This study suggested a possible gene dose-dependent association between MTHFR mutrant alleles and the risk of MS development. | PMID: 26261797 |
| FAM120B | ENSG00000112584 | Constitutive coactivator of peroxisome proliferator-activated receptor gamma | Q96EK7 | hsa:84498 | no | no | no | - | - | - | - | - |
| HMGCL | ENSG00000117305 | Hydroxymethylglutaryl-CoA lyase, mitochondrial | P35914 | hsa:3155 | yes | no | no | Valine, leucine and isoleucine degradation; Butanoate metabolism; Metabolic pathways; Peroxisome | 3-hydroxyglutaric acid (DB04594) | - | - | - |
| AUH | ENSG00000148090 | Methylglutaconyl-CoA hydratase, mitochondrial | Q13825 | hsa:549 | no | no | no | Valine, leucine and isoleucine degradation; Metabolic pathways | - | - | - | - |

**Table S16. Protein-protein interaction analysis of all detected potential causal proteins in brain with targets of current multiple sclerosis drugs**

| MS drug | MS drug target | Known pharmacological action of MS drug target | Potential target | neighborhood_on_chromosome | gene_fusion | phylogenetic_cooccurrence | homology | coexpression | experimentally_determined_interaction | database_annotated | automated_textmining | combined_score |
|---|---|---|---|---|---|---|---|---|---|---|---|---|
| Dimethyl fumarate | RELA | Unknown | TRAF3 | 0 | 0 | 0 | 0 | 0.159 | 0.045 | 0 | 0.596 | 0.647 |
| Alemtuzumab | FCGR3A | Unknown | HLA-B | 0 | 0 | 0 | 0 | 0.183 | 0 | 0 | 0.450 | 0.532 |
| Alemtuzumab | FCGR3B | Unknown | HLA-B | 0 | 0 | 0 | 0 | 0.123 | 0 | 0 | 0.448 | 0.495 |
| Glatiramer | HLA-DRB1 | Unknown | HLA-B | 0 | 0 | 0 | 0.619 | 0.168 | 0 | 0.900 | 0.602 | 0.963 |
| Daclizumab | C1R | Unknown | HLA-B | 0 | 0 | 0 | 0 | 0.161 | 0 | 0 | 0.435 | 0.505 |
| Interferon beta | IFNAR1 | Binder | TRAF3 | 0 | 0 | 0 | 0 | 0.049 | 0.071 | 0 | 0.518 | 0.537 |

MS: multiple sclerosis; Daclizumab is not anymore a licensed drug for MS, due to severe liver toxicity

**Table S17. Protein-protein interaction analysis among all detected potential causal proteins (minimum combined**

| Node1 | Node2 | ıood_on_ch | gene_fusion | netic_coocc | homology | coexpressio | y_determin | base_annot | nated_textn | mbined_score |
|---|---|---|---|---|---|---|---|---|---|---|
| **Interactions of proteins in plasma** | | | | | | | | | | |
| CD59 | CR1 | 0 | 0 | 0 | 0 | 0 | 0 | 0 | 0.824 | 0.824 |
| PGLYRP1 | TNFRSF1A | 0 | 0 | 0 | 0 | 0.060 | 0 | 0 | 0.579 | 0.587 |
| TYMP | WARS | 0.119 | 0 | 0 | 0 | 0.302 | 0 | 0 | 0.108 | 0.403 |
| **Interactions of proteins in brain** | | | | | | | | | | |
| AUH | HMGCL | 0.059 | 0.082 | 0 | 0 | 0.211 | 0 | 0.941 | 0.627 | 0.982 |
| HMGCL | MTHFR | 0.111 | 0 | 0 | 0 | 0.105 | 0 | 0 | 0.393 | 0.475 |
| **Interactions of proteins between plasma and brain (node1: plasma protein; node2: brain protein)** | | | | | | | | | | |
| WARS | TSFM | 0 | 0 | 0 | 0 | 0.114 | 0 | 0 | 0.408 | 0.454 |
| TNFRSF1A | TRAF3 | 0 | 0 | 0 | 0 | 0 | 0.311 | 0 | 0.969 | 0.977 |

**Table S18. Associations between genetically predicted plasma protein levels and MS risk: a Mendelian randomization study**

| Protein | Method | genome-wide pQTL | | | | cis-pQTL | | | |
|---|---|---|---|---|---|---|---|---|---|
| | | IV | OR (95%CI) | *P*-value | *P*-value MR-Egger intercept | IV | OR (95%CI) | *P*-value | *P*-value MR-Egger intercept |
| ARHGAP1 | IVW | 7 | 0.93 (0.76-1.13) | 0.46 | 0.74 | 0 | - | - | - |
| | WM | | 0.93 (0.71-1.20) | 0.56 | | | - | - | |
| CD59 | IVW | 10 | 0.87 (0.66-1.14) | 0.32 | 0.48 | 6 | **0.76 (0.66-0.88)** | **$3.8\times10^{-5}$** | 0.85 |
| | WM | | 0.81 (0.66-0.99) | 0.04 | | | **0.72 (0.59-0.88)** | **$1.0\times10^{-3}$** | |
| CR1 | IVW | 171 | 1.03 (0.98-1.08) | 0.29 | 0.43 | 13 | **1.08 (0.97-1.21)** | **0.01** | 0.94 |
| | WM | | 1.00 (0.94-1.07) | 0.93 | | | **1.22 (1.05-1.41)** | **$9.5\times10^{-3}$** | |
| FCRL3 | IVW | 166 | **0.92 (0.89-0.96)** | **$4.4\times10^{-6}$** | 0.34 | 52 | **0.90 (0.86-0.94)** | **$1.2\times10^{-9}$** | 0.7 |
| | WM | | **0.92 (0.87-0.97)** | **$1.3\times10^{-3}$** | | | **0.91 (0.87-0.97)** | **$1.4\times10^{-3}$** | |
| IDUA | IVW | 125 | **0.92 (0.88-0.97)** | **$1.2\times10^{-3}$** | 0.82 | 28 | **0.91 (0.87-0.96)** | **$3.1\times10^{-5}$** | 0.9 |
| | WM | | **0.92 (0.87-0.97)** | **$4.4\times10^{-3}$** | | | **0.88 (0.83-0.94)** | **$4.7\times10^{-5}$** | |
| PGLYRP1 | IVW | 49 | 3.47 (1.29-9.35) | 0.01 | 0.66 | 4 | **0.71 (0.59-0.85)** | **$3.0\times10^{-4}$** | 0.38 |
| | WM | | 0.83 (0.72-0.96) | 0.01 | | | **0.70 (0.57-0.85)** | **$3.4\times10^{-4}$** | |
| TNFRSF1A | IVW | 17 | 1.09 (0.77-1.54) | 0.64 | 0.73 | 0 | - | - | - |
| | WM | | 1.25 (0.99-1.58) | 0.07 | | | - | - | |
| TYMP | IVW | 7 | **0.63 (0.36-1.10)** | **0.11** | 0.71 | 0 | - | - | - |
| | WM | | **0.72 (0.54-0.96)** | **0.03** | | | - | - | |
| WARS | IVW | 30 | **1.22 (1.13-1.33)** | **$2.1\times10^{-6}$** | 0.65 | 3 | 1.47 (0.84-2.56) | 0.18 | 0.92 |
| | WM | | **1.22 (1.09-1.35)** | **$4.4\times10^{-4}$** | | | 1.50 (0.81-2.77) | 0.2 | |

IVW: inverse-variance weighted method; WM: weighted median method; IV: instrument variable; OR: odds ratio; CI: confidence interval

**Table S19. Associations between genetically predicted brain protein levels and multiple sclerosis risk: a Mendelian randomization study**

| Protein | Method | cis-pQTL | | |
|---|---|---|---|---|
| | | **IV** | **OR (95%CI)** | ***P*-value** |
| HLA-B | WR | 0 | - | - |
| | RAPS | | - | - |
| ICA1L | WR | 0 | - | - |
| | RAPS | | - | - |
| HMGCL | WR | 1 | **0.25 (0.12-0.52)** | **$2.0\times10^{-4}$** |
| | RAPS | | **0.25 (0.11-0.58)** | **$1.3\times10^{-3}$** |
| FAM120B | WR | 0 | - | - |
| | RAPS | | - | - |
| AUH | WR | 0 | - | - |
| | RAPS | | - | - |
| TSFM | WR | 1 | **0.20 (0.12-0.32)** | **$6.4\times10^{-11}$** |
| | RAPS | | **0.20 (0.10-0.38)** | **$1.5\times10^{-6}$** |
| ZC2HC1A | WR | 0 | - | - |
| | RAPS | | - | - |
| TRAF3 | WR | 0 | - | - |
| | RAPS | | - | - |
| MTHFR | WR | 1 | **0.48 (0.31-0.75)** | **$1.4\times10^{-3}$** |
| | RAPS | | **0.48 (0.28-0.81)** | **$5.8\times10^{-3}$** |

WR: Wald ratio method; RAPS: Robust adjusted profile score method; IV: instrument variable; OR: odds

**Table S20. For all relaxed-criteria-based potential causal proteins in plasma: pathway annotation, protein-targeted drugs, evidence in previous studies**

| Gene | Ensembl_ID | Protein | UniProt | KEGG ID | Central dogma | Stringent criteria | Known MS drug target | Link with known MS drug traget | Pathway | Protein-targeted drug | General function of the identified drug | Protein (gene)-MS relations suggested by previous studies | DOI |
|---|---|---|---|---|---|---|---|---|---|---|---|---|---|
| ARHGAP1 | ENSG00000175220 | RHG01 | Q07960 | hsa:392 | no | yes | no | no | - | - | - | - | - |
| PGLYRP1 | ENSG00000008438 | PGRP1 | O75594 | hsa:8993 | no | yes | no | no | - | - | - | PGLYRP1 is suggested to serve as a proinflammatory molecule in myeloid cells during autoimmune conditions. These findings shed light on the potential of PGLYRP1 as a target for immunotherapy. Targeting PGLYRP1 could trigger a robust antitumor immune response while also offering protection against certain types of tissue inflammation and autoimmune reactions. The PGLYRP1 protein shows expression in cerebellar and spinal cord tissues of patients with MS. Immunofluorescence analysis revealed elevated levels of PGLYRP1 protein in both the white and gray matter of the cerebellum and spinal cord in patients with MS. Immunoblot analysis of spinal cord tissue from patients with MS confirmed the upregulation of PGLYRP1 protein, consistent with the findings from immunofluorescence analysis. Conversely, no significant immunoreactivity of PGLYRP1 protein was observed in the controls | https://doi.org/10.1038/s41590-023-01645-4; https://doi.org/10.1016/j.celrep.2024.113813 |
| CR1 | ENSG00000203710 | Complement receptor type 1 | P17927 | hsa:1378 | no | yes | no | yes | Complement and coagulation cascades; Neutrophil extracellular trap formation; Hematopoietic cell lineage; Legionellosis; Leishmaniasis; Malaria; Tuberculosis | - | - | CR1-positive immunolabeled cells were significantly increased in white matter lesions tissue compared with normal appearing white matter. | https://doi.org/10.1111/bpa.12546 |
| WARS | ENSG00000140105 | SYWC | P23381 | hsa:7453 | no | yes | no | no | Aminoacyl-tRNA biosynthesis | Tryptophan (DB00150), Tryptophanyl-5'amp (DB01831), Tryptophanamide (DB04537) | Tryptophan is an amino acid commonly found as a component of total parenteral nutrition. | - | - |
| IDUA | ENSG00000127415 | IDUA | P35475 | hsa:3425 | no | yes | no | no | Glycosaminoglycan degradation; Metabolic pathways; Lysosome | Laronidase (DB00090) | Laronidase is a form of recombinant human alpha-L-iduronidase used to treat Hurler and Hurler-Scheie forms of mucopolysaccharidosis, a genetic deficiency of alpha-L-iduronidase. | IDUA protein associated with MS progression. | https://doi.org/10.1101/2022.07.29.22278211 |
| CD59 | ENSG00000085063 | CD59 | P13987 | hsa:966 | no | yes | no | yes | Complement and coagulation cascades; Hematopoietic cell lineage | - | - | The CSF levels of CD59 were upregulated in NMO and MS patients. The CSF CD59 levels decreased after treatment. Elevated CSF CD59 levels may reflect complement activation within CNS. https://doi.org/10.1016/j.cca.2015.12.013 The deficiency of CD59 is one of factor that exacerbates MS disease, as demonstrated by MS experimental animals with CD55 and CD59 gene deficiencies, which resulted in more severe rates of MS disease. | https://doi.org/10.1016/j.cca.2015.12.013; https://doi.org/10.3389/fimmu.2022.970486 |

| | | | | | | | | | | | | |
|---|---|---|---|---|---|---|---|---|---|---|---|---|
| TNFRSF1A | ENSG00000067182 | TNR1A | P19438 | hsa:7132 | no | yes | no | yes | MAPK signaling pathway; Cytokine-cytokine receptor interaction; Viral protein interaction with cytokine and cytokine receptor; NF-kappa B signaling pathway; Sphingolipid signaling pathway; mTOR signaling pathway; Apoptosis; Apoptosis - multiple species; Necroptosis; Osteoclast differentiation; TNF signaling pathway; Adipocytokine signaling pathway; Insulin resistance; Non-alcoholic fatty liver disease; Alcoholic liver disease; Alzheimer disease; Amyotrophic lateral sclerosis; Pathways of neurodegeneration - multiple diseases; Pathogenic Escherichia coli infection; Shigellosis; Salmonella infection; Chagas disease; Toxoplasmosis; Tuberculosis; Hepatitis C; Human cytomegalovirus infection; Influenza A; Human papillomavirus infection; Human T-cell leukemia virus 1 infection; Kaposi sarcoma-associated herpesvirus infection; Herpes simplex virus 1 infection; Human immunodeficiency virus 1 infection; Coronavirus disease - COVID-19; Lipid and atherosclerosis; Fluid shear stress and atherosclerosis | 6-[3-(4-Morpholinyl)Propyl]-2-(3-Nitrophenyl)-5-Thioxo-5,6,-Dihydro-7h-Thienol[2',3':4,5]Pyrrolo[1,2-C]Imidazol-7-One (DB03507), Tasonermin (DB11626) | Tasonermin is a tumor necrosis factor alpha used along with surgery to remove soft tissue sarcomas of the limbs. Tasonermin is a tumor necrosis factor alpha used along with surgery to remove soft tissue sarcomas of the limbs. | A polymorphism associated with an elevated risk of developing MS has been identified in the TNFRSF1A gene, marking TNFRSF1A as a novel susceptibility locus for multiple sclerosis. | https://doi.org/10.1212/01.wnl.0000436612.66328.8a; https://doi.org/10.1212/WNL.0b013e318294b2d6 |
| FCRL3 | ENSG00000160856 | FCRL3 | Q96P31 | hsa:115352 | no | yes | no | yes | - | - | - | FCRL3 can activate the SHP-1 and p38 MAPK pathways and then promote the secretion of IL-10 in B cells, thus inhibiting the secretion of inflammatory factors. Therefore, FCRL3 may play an immunoprotective role in MS, and it will be an effective target for the diagnosis and treatment of MS. TLR9 activation impacts various aspects of B cell function, including proliferation, apoptosis, antibody production, and IL-10 secretion, through the upregulation of FCRL3 expression. Consequently, FCRL3 emerges as a significant target for both diagnosis and treatment of B cell-related diseases. Studies found that FCRL3 variants were associated with MS | https://doi.org/10.1002/cbin.11373；https://doi.org/10.1007/s11626-022-00720-8 |

| | | | | | | | | | | | | |
|---|---|---|---|---|---|---|---|---|---|---|---|---|
| TYMP | ENSG00000025708 | TP | P19971 | hsa:1890 | no | yes | no | yes | Pyrimidine metabolism; Drug metabolism - other enzymes; Metabolic pathways; Nucleotide metabolism; Bladder cancer | Tipiracil (DB09343), Capecitabine (DB01101), Floxuridine (DB00322), Fluorouracil (DB00544), Trifluridine (DB00432), Cidofovir (DB00369), Tezacitabine (DB06433) | Tipiracil is a thymidine phosphorylase inhibitor used as an adjunct treatment of adult patients with certain types of gastric or colorectal malignancies. Floxuridine is an antimetabolite used as palliative management for liver metastases of gastrointestinal malignancy. Fluorouracil is a pyrimidine analog used to treat basal cell carcinomas, and as an injection in palliative cancer treatment. Trifluridine is a nucleoside metabolic inhibitor used to treat keratoconjunctivitis and epithelial keratitis caused by simplex virus, and as a part of chemotherapy for certain types of metastatic gastrointestinal cancers. Cidofovir is an antiviral agent used to treat Cytomegalovirus (CMV) retinitis in patients with AIDS. | The gene TYMP encoding for the protein tymidine phosphorylase was found downregulated from MS. TYMP was reported as the second key astrocyte-derived permeability factor that, via Vascular Endothelial Growth Factor A (VEGFA) interaction, plays an essential role in the blood-brain-barrier disruption observed in Experimental Autoimmune Encephalomyelitis lesions, the animal model of MS Chapouly C, Tadesse Argaw A, Horng S, Castro K, Zhang J, Asp L, et al. Astrocytic TYMP and VEGFA drive blood-brain barrier opening in inflammatory central nervous system lesions. Brain. 2015; 138: 1548–1567. | https://doi.org/10.31083/j.jin2201004; https://doi.org/10.1093/brain/awv077 |
| CD58 | ENSG00000116815 | sLFA-3 | P19256 | hsa:965 | no | no | no | yes | Cell adhesion molecules; Epstein-Barr virus infection | Alefacept (DB00092) | Alefacept is a genetically engineered immunosuppressive drug used for plaque psoriasis treatment | Allelic variants within the CD58 gene region, which encodes the costimulatory molecule LFA-3, have been linked to an increased susceptibility to MS. Functional investigations suggest a potential mechanism that protective allele raises CD58 expression, and then up-regulates the expression of transcription factor FoxP3 through engagement of the CD58 receptor, CD2, leading to the enhanced function of CD4+CD25high regulatory T cells that are defective in subjects with MS. Variants in the CD58 gene locus point to a role of microRNA-548ac in the pathogenesis of multiple sclerosis. | https://doi.org/10.1073/pnas.0813310106; https://doi.org/10.1016/j.mrrev.2014.10.002 |
| BCAT2 | ENSG00000105552 | BCAT2 | O15382 | hsa:587 | no | no | no | yes | Cysteine and methionine metabolism; Valine, leucine and isoleucine degradation; Valine, leucine and isoleucine biosynthesis; Pantothenate and CoA biosynthesis; Metabolic pathways; 2-Oxocarboxylic acid metabolism; Biosynthesis of amino acids; Biosynthesis of cofactors | Pyridoxal phosphate (DB00114), Glutamic acid (DB00142), Leucine (DB00149), Isoleucine (DB00167), Pyridoxamine-5'-Phosphate (DB02142), N-[O-Phosphono-Pyridoxyl]-Isoleucine (DB02635), alpha-Ketoisovalerate (DB04074) | Pyridoxal phosphate is a vitamin available in many formulations to correct vitamin B6 deficiency. Glutamic acid is an amino acid commonly found as a component in total parenteral nutrition. Leucine is an amino acid commonly found as a component of total parenteral nutrition. Isoleucine is an amino acid commonly found as a component of total parenteral nutrition. | - | - |
| ST3GAL6 | ENSG00000064225 | SIA10 | Q9Y274 | hsa:10402 | no | no | no | no | Glycosphingolipid biosynthesis - lacto and neolacto series; Metabolic pathways | - | - | - | - |

| | | | | | | | | | | | | | |
|---|---|---|---|---|---|---|---|---|---|---|---|---|---|
| TCN1 | ENSG00000 134827 | Holo-TC I | P20061 | hsa:6947 | no | no | no | no | - | Hydroxocobalamin (DB00200) | Hydroxocobalamin is a synthetic form of vitamin B12 used to treat vitamin B12 associated disorders and cyanide poisoning. | Patients with clinically isolated syndrome exhibit a statistically elevated level of serum Tcn1 compared to other groups. Analysis of an additional dataset comprising 19 participants confirms a notably higher concentration of Tcn1 in female patients but not in male patients. These findings lend support to the notion that transcobalamin 1 may play a role in the pathophysiology of multiple sclerosis in females, particularly during the early stages of the disease. | https://doi.org/10.1371/ journal.pone.0090482 |
| MMP10 | ENSG00000 166670 | MMP-10 | P09238 | hsa:4319 | no | no | no | no | - | Marimastat (DB00786), N-ISOBUTYL-N-[4-METHOXYPHENYLSULFONYL]GLYCYL HYDROXAMIC ACID (DB08271) | Used in the treatment of cancer, Marmiastat is an angiogenesis and metastasis inhibitor. | MMP-10 concentrations were higher in patients with CSF-restricted IgG oligoclonal bands. Matrix metalloproteinases (MMPs) are implicated in multiple sclerosis Of interest is the enrichment of a majority of MMP members in monocytes: MMP-1, MMP-3, MMP-9, MMP-10, MMP-14, MMP-19 and MMP-25. | https://doi.org/10.3389/ fneur.2018.01080; https://doi.org/10.1093/ brain/awg285 |
| SMPDL3A | ENSG00000 172594 | ASM3A | Q92484 | hsa:1092 4 | no | no | no | no | - | - | - | In vitro studies demonstrate that SMPDL3A selectively hydrolyzes 2′3′-cGAMP without affecting other cyclic dinucleotides. Moreover, SMPDL3A inhibits STING–IFN-induced responses in cell-based assays. These findings collectively suggest that LXR signaling triggers the expression of SMPDL3A, an enzyme dedicated to degrading 2′3′-cGAMP, thereby dampening inflammatory signaling mediated by STING. | https://doi.org/10.1038/ s41577-023-00962-x |
| CBR3 | ENSG00000 159231 | CBR3 | O75828 | hsa:874 | no | no | no | no | Arachidonic acid metabolism; Metabolism of xenobiotics by cytochrome P450; Metabolic pathways | - | - | - | - |
| AHSG | ENSG00000 145192 | a2-HS-Glycoprotei n | P02765 | hsa:197 | no | no | no | no | - | Zinc (DB01593), Copper(DB09130), Zinc acetate(DB14487), Zinc chloride (DB14533) | Zinc is an essential element commonly used for the treatment of patients with documented zinc deficiency. Copper is a transition metal found in a variety of supplements and vitamins, including intravenous solutions for total parenteral nutrition (TPN). Zinc acetate is a medication used to treat zinc deficiency. Zinc chloride is a medication used to treat zinc deficiencies and associated symptoms and also in total parenteral nutrition. | In MS, changes in cerebrospinal fluid levels of fetuin-A (also known as alpha-2-HS-glycoprotein or AHSG) are linked to the early transition to relapsing–remitting MS. Additionally, increased levels of fetuin-A are observed in secondary progressive MS, whereas they are not typically elevated in primary progressive MS. | https://doi.org/10.1016/ j.nbd.2010.06.007; https://doi.org/10.1177/ 1352458507076406; https://doi.org/10.1016/ j.jprot.2010.01.004; https://doi.org/10.1016/ j.neulet.2009.01.057 |

| | | | | | | | | | | | | | |
|---|---|---|---|---|---|---|---|---|---|---|---|---|---|
| STAT3 | ENSG00000168610 | STAT3 | P40763 | hsa:6774 | no | no | no | yes | EGFR tyrosine kinase inhibitor resistance; Chemokine signaling pathway; HIF-1 signaling pathway; FoxO signaling pathway; Necroptosis; Signaling pathways regulating pluripotency of stem cells; JAK-STAT signaling pathway; Th17 cell differentiation; Prolactin signaling pathway; Adipocytokine signaling pathway; Insulin resistance; AGE-RAGE signaling pathway in diabetic complications; Growth hormone synthesis, secretion and action; Toxoplasmosis; Hepatitis C; Hepatitis B; Measles; Human cytomegalovirus infection; Kaposi sarcoma-associated herpesvirus infection; Epstein-Barr virus infection; Coronavirus disease - COVID-19; Pathways in cancer; Viral carcinogenesis; Proteoglycans in cancer; MicroRNAs in cancer; Chemical carcinogenesis - receptor activation; Pancreatic cancer; Acute myeloid leukemia; Non-small cell lung cancer; PD-L1 expression and PD-1 checkpoint pathway in cancer; Inflammatory bowel disease; Lipid and atherosclerosis | ENMD-1198 (DB05959), Napabucasin (DB12155) | Napabucasin has been investigated for the treatment of Colorectal Carcinoma. | The level of STAT3 methylation significantly decreased in relapsing–remitting MS patients compared to control groups. STAT3 gene expression significantly increased in the patient group compared with healthy controls. Therefore, DNA hypermethylation of STAT3 affects the gene expression. Additionally, a strong and significant negative correlation exists between STAT3 methylation status and mRNA levels. STAT3 genes could be downregulated by M2000 in patients with secondary progressive MS. | https://doi.org/10.1186/s13104-020-05427-1; https://doi.org/10.1002/jcph.2008 |
| HP | ENSG00000257017 | HPT | P00738 | | no | no | no | yes | - | Zinc chloride(DB14533), Zinc sulfate, unspecified form(DB14548) | Zinc chloride is a medication used to treat zinc deficiencies and associated symptoms and also in total parenteral nutrition.<br>Zinc sulfate, unspecified form is a zinc supplement indicated in parenteral nutrition. | - | - |
| KYNU | ENSG00000115919 | KYNU | Q16719 | hsa:8942 | no | no | no | no | Tryptophan metabolism; Metabolic pathways; Biosynthesis of cofactors | Pyridoxal phosphate (DB00114), Alanine (DB00160), m-Hydroxyhippuric acid (DB07069) | Pyridoxal phosphate is a vitamin available in many formulations to correct vitamin B6 deficiency. Alanine is an amino acid commonly found as a component of total parenteral nutrition. | - | - |
| FCRL4 | ENSG00000163518 | FCRL4 | Q96PJ5 | | no | no | no | no | - | - | | - | - |

| | | | | | | | | | | | | | |
|---|---|---|---|---|---|---|---|---|---|---|---|---|---|
| IL2RA | ENSG00000 134460 | IL-2 sRa | P01589 | hsa:3559 | no | no | yes | yes | Cytokine-cytokine receptor interaction; Viral protein interaction with cytokine and cytokine receptor; Endocytosis; PI3K-Akt signaling pathway; JAK-STAT signaling pathway; Hematopoietic cell lineage; Th1 and Th2 cell differentiation; Th17 cell differentiation; Measles; Human T-cell leukemia virus 1 infection; Pathways in cancer | Denileukin diftitox (DB00004); Basiliximab (DB00074); Daclizumab (DB00111); Aldesleukin (DB00041); Inolimomab (DB15628); Camidanlumab tesirine (DB16411) | Denileukin diftitox is a recombinant cytotoxic protein based on a combination of diphtheria toxin fragments and interleukin-2 used to treat cutaneous T-cell lymphoma by targeting the interleukin-2 receptor. Basiliximab is a monoclonal anti-C25 antibody (interleukin-2 receptor alpha subunit) used as immunosuppressive therapy in kidney transplant patients. Daclizumab is a monoclonal anti-CD25 antibody that blocks the interleukin-2 receptor which is used to treat relapsing forms of multiple sclerosis. Aldesleukin is a recombinant analog of interleukin-2 used to induce an adaptive immune response in the treatment of renal cell carcinoma. Inolimomab is under investigation as an agent to treat steroid-resistant graft vs host disease. Camidanlumab tesirine is under investigation. | - | - |
| CCDC126 | ENSG00000 169193 | CC126 | Q96EE4 | hsa:3559 | no | no | no | no | - | - | - | - | - |

| | | | | | | | | | | | | |
|---|---|---|---|---|---|---|---|---|---|---|---|---|
| MMP13 | ENSG00000137745 | MMP-13 | P45452 | hsa:4322 | no | no | no | yes | IL-17 signaling pathway; Relaxin signaling pathway; Parathyroid hormone synthesis, secretion and action | 3-Methylpyridine(DB01996); WAY-151693(DB02071); Hydroxyaminovaline (DB02697); 4-methoxybenzenesulfinate (DB03033); PYRIMIDINE-4,6-DICARBOXYLIC ACID BIS-(3-METHYL-BENZYLAMIDE) (DB04759); PYRIMIDINE-4,6-DICARBOXYLIC ACID BIS-(4-FLUORO-3-METHYL-BENZYLAMIDE) (DB04760); PYRIMIDINE-4,6-DICARBOXYLIC ACID BIS-[(PYRIDIN-3-YLMETHYL)-AMIDE] (DB04761); Endostatin (DB06423); Marimastat (DB00786); TERT-BUTYL 4-({[4-(BUT-2-YN-1-YLAMINO)PHENYL] [illegible] | All drugs are under experiment or investigation | MMP13 expression is increased following mutant α-synuclein exposure and promotes inflammatory responses in microglia | https://doi.org/10.3389/fnins.2020.585544 |
| ARFIP1 | ENSG00000164144 | ARFP1 | P53367 | sasa:100380283 | no | no | no | no | - | - | - | - | - |

| | | | | | | | | | | | | | |
|---|---|---|---|---|---|---|---|---|---|---|---|---|---|
| CASP3 | ENSG00000164305 | Caspase-3 | P42574 | hsa:836 | no | no | no | yes | Platinum drug resistance; MAPK signaling pathway; p53 signaling pathway; Efferocytosis; Apoptosis; Apoptosis - multiple species; Cytosolic DNA-sensing pathway; Natural killer cell mediated cytotoxicity; IL-17 signaling pathway; TNF signaling pathway; Serotonergic synapse; Non-alcoholic fatty liver disease; AGE-RAGE signaling pathway in diabetic complications; Alcoholic liver disease; Alzheimer disease; Parkinson disease; Amyotrophic lateral sclerosis; Huntington disease; Prion disease; Pathways of neurodegeneration - multiple diseases; Epithelial cell signaling in Helicobacter pylori infection; Pathogenic Escherichia coli infection; Salmonella infection; Pertussis; Legionellosis; Toxoplasmosis; Amoebiasis; Tuberculosis; Hepatitis C; Hepatitis B; Measles; Human cytomegalovirus infection; Influenza A; Human papillomavirus infection; Kaposi sarcoma-associated herpesvirus infection; Herpes simplex virus 1 infection; Epstein-Barr virus infection; Human immunodeficiency virus 1 infection; Pathways in cancer; Viral carcinogenesis; Proteoglycans in cancer; MicroRNAs in cancer; Colorectal cancer; Small cell lung cancer; Viral myocarditis<br>Lipid and atherosclerosis | Minocycline (DB01017); 5-[4-(1-Carboxymethyl-2-Oxo-Propylcarbamoyl)-Benzylsulfamoyl]-2-Hydroxy-Benzoic Acid (DB03124); Emricasan (DB05408); 2-HYDROXY-5-(2-MERCAPTO-ETHYLSULFAMOYL)-BENZOIC ACID (DB06862); methyl (3S)-3-[(tert-butoxycarbonyl)amino]-4-oxopentanoate (DB07696); 1-METHYL-5-(2-PHENOXYMETHYL-PYRROLIDINE-1-SULFONYL)-1H-INDOLE-2,3-DIONE (DB08213); [N-(3-dibenzylcarbamoyl-oxiranecarbonyl)-hydrazino]-acetic acid (DB08229); 4-[5-(2-CARBOXY-1-FORMYL-ETHYLCARBAMOYL)-PYRIDIN-3-YL]-BENZOIC ACID | Glycyrrhizic acid has been developed in Japan and China as a hepatoprotective drug in cases of chronic hepatitis. PAC-1 has been used in trials studying the treatment of Lymphoma, Melanoma, Solid Tumors, Breast Cancer, and Thoracic Cancers, among others. Minocycline is a tetracycline analog used to treat a wide variety of infections in the body. The other drugs are under experiment or investigation | Activation of the executioner caspases-3 and-7 promotes microglial pyroptosis in models of multiple sclerosis | https://doi.org/10.1186/s12974-020-01902-5 |
| PFKM | ENSG00000152556 | K6PF | P08237 | hsa:5213 | no | no | no | no | Glycolysis / Gluconeogenesis; Pentose phosphate pathway; Fructose and mannose metabolism; Galactose metabolism; Metabolic pathways; Carbon metabolism; Biosynthesis of amino acids; RNA degradation; HIF-1 signaling pathway; AMPK signaling pathway; Thyroid hormone signaling pathway; Glucagon signaling pathway; Central carbon metabolism in cancer | - | - | - | - |
| ATXN3 | ENSG00000066427 | ATX3 | P54252 | hsa:4287 | no | no | no | no | Protein processing in endoplasmic reticulum; Spinocerebellar ataxia; Pathways of neurodegeneration - multiple diseases | - | - | ATXN3 is ubiquitously expressed throughout all cell types in the central nervous system and was first described to function as a deubiquitinase with roles in protein quality control pathways. | https://doi.org/10.3390/cells11162615 |

| | | | | | | | | | | | | | |
|---|---|---|---|---|---|---|---|---|---|---|---|---|---|
| CD274 | ENSG00000120217 | B7-H1 | Q9NZQ7 | hsa:29126 | no | no | no | yes | Cell adhesion molecules; PD-L1 expression and PD-1 checkpoint pathway in cancer | Nivolumab (DB09035); Pembrolizumab (DB09037); Valproic acid (DB00313); Sildenafil (DB00203); Atezolizumab (DB11595); Avelumab (DB11945); Durvalumab (DB11714); CA-170 (DB15772); anti-OX40 antibody BMS 986178 (DB15773); Envafolimab (DB15769); Cosibelimab (DB15770); AUNP-12 (DB15771); Camrelizumab (DB14776) | Nivolumab is a PD-1 blocking antibody used to treat melanoma, non small-cell lung cancer, renal cell cancer, head and neck cancer, and Hodgkin lymphoma. Pembrolizumab is a PD-1 blocking antibody used to treat various types of cancer, including metastatic melanoma, non small-cell lung cancer, cervical cancer, head and neck cancer, and Hodgkin's lymphoma. Valproic acid is an anticonvulsant used to control complex partial seizures and both simple and complex absence seizures. Sildenafil is a phosphodiesterase inhibitor used for the treatment of erectile dysfunction. Atezolizumab is a monoclonal antibody used to treat advanced or metastatic urothelial carcinoma with disease progression during or up to 12 months after platinum-containing chemotherapy. Avelumab is an anti-PD-L1 monoclonal antibody used to treat metastatic merkel cell carcinoma, metastatic urothelial carcinoma, or renal cell carcinoma. Durvalumab is an antineoplastic monoclonal [illegible] | CD274 was upregulated in pregnant MS patients compared to the untreated RRMS patients. | https://doi.org/10.3389/fimmu.2020.552101 |
| C1QL1 | ENSG00000131094 | C1QRF | O75973 | hsa:10882 | no | no | no | yes | - | - | - | C1ql3 knockout affects microglia activation, neuronal integrity, and spontaneous behavior in Wistar rats | https://doi.org/10.1002/ame2.12383 |
| TNFSF12 | ENSG00000239697 | TWEAK | O43508 | hsa:407977 | no | no | no | no | - | - | - | MS is characterized by communication disturbances between the central nervous system and the immune system, often involving cytokines. One such cytokine is TWEAK (TNFSF12), a transmembrane protein belonging to the tumor necrosis facto ligand family. TWEAK transcripts are detected in various tissues, including the brain, and can be released as a soluble cytokine. Monocytes/macrophages, including microglia, are primary sources of TWEAK protein. In experimental mouse models of MS, TWEAK exhibits pro-inflammatory effects during central nervous system inflammation. | https://doi.org/10.1186/s12967-019-1789-3; https://doi.org/10.1189/jlb.0608347 |
| WISP2 | ENSG00000064205 | WISP-2 | O76076 | hsa:8839 | no | no | no | no | - | - | - | CCN5 (previously identified as WISP-2) is a member of the connective tissue growth factor/cysteine-rich 61/nephroblastoma overexpressed (CCN) family, known for its crucial roles in cell growth, adhesion, and migration. Studies have shown a decrease in CCN5 levels in the CSF of patients with clinically definite multiple sclerosis . In an experimental autoimmune encephalomyelitis (EAE) model of MS, CCN5 mRNA expression was found to be significantly upregulated in spinal cord tissue. | https://doi.org/10.3389/fimmu.2021.811351; https://doi.org/10.1371/journal.pone.0157754 |
| CXCL11 | ENSG00000169248 | I-TAC | O14625 | hsa:6373 | no | no | no | yes | Cytokine-cytokine receptor interaction; Viral protein interaction with cytokine and cytokine receptor; Chemokine signaling pathway; Toll-like receptor signaling pathway | - | - | Serum levels of CXCL11 were found to be significantly higher in patients with relapsing-remitting multiple sclerosis compared to controls | https://doi.org/10.1159/000107945 |

| | | | | | | | | | | | | |
|---|---|---|---|---|---|---|---|---|---|---|---|---|
| CXCL12 | ENSG00000107562 | SDF-1 | P48061 | hsa:6387 | no | no | no | yes | Cytokine-cytokine receptor interaction; Viral protein interaction with cytokine and cytokine receptor; Chemokine signaling pathway; NF-kappa B signaling pathway; Axon guidance; Leukocyte transendothelial migration; Intestinal immune network for IgA production; Regulation of actin cytoskeleton; Human cytomegalovirus infection; Pathways in cancer; Rheumatoid arthritis | Tinzaparin (DB06822); CTCE-0214 (DB05934) | Tinzaparin is a low molecular weight heparin used for the treatment of acute symptomatic deep vein thrombosis with or without pulmonary embolism when administered in conjunction with warfarin. CTCE-0214 is under investigation for use/treatment in adverse effects (chemotherapy), blood (blood-forming organ disorders, unspecified), cancer/tumors (unspecified), neutropenics, and vascular diseases. | Immunohistochemically, CXCL12 was constitutively expressed in the central nervous system parenchyma on blood vessel walls. In both active and chronically inactive lesions of multiple sclerosis, CXCL12 protein levels were elevated and detected on astrocytes and blood vessels. In relapsing-remitting multiple sclerosis patients, CXCL12 was elevated. The elevated levels of CXCL12 in active multiple sclerosis lesions, and also in inactive lesions, suggest potential consequences. CXCL12's presence on blood vessels suggests a possible role in leukocyte extravasation, and it may contribute to plasma cell persistence, given that its receptor CXCR4 is retained during plasma cell differentiation. Furthermore, CXCL12 may contribute to axonal damage, as it can act as a neurotoxic mediator of cleavage by metalloproteases found in multiple sclerosis lesions. | https://doi.org/10.1093/brain/awh680 |
| JAM3 | ENSG00000166086 | JAM-C | Q9BX67 | hsa:83700 | no | no | no | yes | Cell adhesion molecules; Tight junction; Leukocyte transendothelial migration; Epithelial cell signaling in Helicobacter pylori infection | - | - | The downregulation of Jam3 could potentially contribute to the loss of myelin integrity observed in cuprizone-induced demyelination. Notably, Jam3 was also identified as a common differentially expressed gene between human multiple sclerosis samples and contrals. After glucocorticoid treatment of MS patients in relapse, RNA levels of JAM3 decreased, and claudin 5 protein levels in Peripheral Blood Leukocytes (PBLs) decreased as well. | https://doi.org/10.3390/ijms232314868; https://doi.org/10.1111/j.1582-4934.2011.01380.x |
| TAPBPL | ENSG00000139192 | TPSNR | Q9BX59 | hsa:55080 | no | no | no | | - | - | - | The in vivo administration of hTAPBPL-Ig protein attenuates experimental autoimmune encephalomyelitis (EAE) in mice. | https://doi.org/10.15252/emmm.202013404 |
| TLR1 | ENSG00000174125 | TLR1 | Q15399 | hsa:7096 | no | no | no | yes | Toll-like receptor signaling pathway; Tuberculosis | - | - | TLR1 is down regulated in peripheral blood mononuclear cells (PBMC) of MS patients and up regulated in patients treated with INFβ | https://doi.org/10.4049/jimmunol.178.8.5076. https://doi.org/10.1016/j.jns.2007.02.034. |

**Table S21. For all relaxed-criteria-based potential causal proteins in brain: pathway annotation, targeted existing drugs, and evidence in previous studies**

| Gene | Ensembl_ID | Protein | UniProt | KEGG ID | Central dogma | Stringent criteria | Known MS drug target | Link with known MS drug traget | Pathway | Protein-targeted drug | General function of the identified drug | Protein (gene)-MS relations suggested by previous studies | DOI |
|---|---|---|---|---|---|---|---|---|---|---|---|---|---|
| HLA-B | ENSG00000234745 | HLA class I histocompatibility antigen, B alpha chain | Q31612 | hsa:3106 | no | yes | no | yes | Endocytosis; Phagosome; Cellular senescence; Cell adhesion molecules; Antigen processing and presentation; Natural killer cell mediated cytotoxicity; Type I diabetes mellitus; Human cytomegalovirus infection; Human papillomavirus infection; Human T-cell leukemia virus 1 infection; Kaposi sarcoma-associated herpesvirus infection; Herpes simplex virus 1 infection; Epstein-Barr virus infection; Human immunodeficiency virus 1 infection; Viral carcinogenesis; Autoimmune thyroid disease; Allograft rejection; Graft-versus-host disease; Viral myocarditis | - | - | The HLA-B*44:02 allele, known to confer protective effects against multiple sclerosis (MS) susceptibility, was associated with improved MRI outcomes, including better brain parenchymal fraction and reduced T2 hyperintense lesion volume. Additionally, this allele (with frequencies of 4.4% in women with MS and 6.9% in control women) was nominally linked to the preservation of subcortical gray matter fraction. | https://doi.org/10.1212/WNL.0b013e3181ed9c9c; https://doi.org/10.1001/jamaneurol.2016.0980 |
| TSFM | ENSG00000123297 | Elongation factor Ts, mitochondrial | P43897 | hsa:10102 | no | yes | no | no | - | - | - | The probe set corresponding to exon 4 of TSFM showed significantly higher levels in healthy controls compared to patients with MS. | https://doi.org/10.3389/fimmu.2022.931831 |
| ZC2HC1A | ENSG00000104427 | Zinc finger C2HC domain-containing | Q96GY0 | hsa:51101 | no | yes | no | no | - | - | - | the eQTL SMR tests identified that ZC2HC1A was significantly associated with MS | https://doi.org/10.1007/s00415-019-09476-w |
| TRAF3 | ENSG00000131323 | TNF receptor-associated factor 3 | Q13114 | hsa:7187 | yes | yes | no | yes | NF-kappa B signaling pathway; Toll-like receptor signaling pathway; NOD-like receptor signaling pathway; RIG-I-like receptor signaling pathway; IL-17 signaling pathway; TNF signaling pathway; Alcoholic liver disease; Hepatitis C; Hepatitis B; Measles; Influenza A; Human papillomavirus infection; Kaposi sarcoma-associated herpesvirus infection; Herpes simplex virus 1 infection; Epstein-Barr virus infection; Coronavirus disease - COVID-19; Pathways in cancer; Viral carcinogenesis; Small cell lung cancer; Lipid and atherosclerosis | - | - | Peli1 promotes microglia-mediated CNS inflammation by regulating Traf3 degradation. TRAF3 haploinsufficiency syndrome exhibited B cell hyperactivity leading to hypergammaglobulinemia and autoimmunity | https://doi.org/10.1038/nm.3111; https://doi.org/10.1126/sciimmunol.abn3800 |
| ICA1L | ENSG00000163596 | Islet cell autoantigen 1-like protein | Q8NDH6 | hsa:130026 | no | yes | no | no | - | - | - | - | - |
| MTHFR | ENSG00000177000 | Methylenetetrahydrofolate reductase (NADPH) | P42898 | hsa:4524 | no | yes | no | no | One carbon pool by folate; Metabolic pathways; Antifolate resistance | Cyanocobalamin(Vitamin B12, DB00115), Tetrahydrofolic acid (DB00116) | Vitamin B12 is a vitamin available in many formulations to correct vitamin B12 deficiency. Tetrahydrofolic acid is for nutritional supplementation, also for treating dietary shortage or imbalance. | This study suggested a possible gene dose-dependent association between MTHFR mutrant alleles and the risk of MS development. | PMID: 26261797 |
| FAM120B | ENSG00000112584 | Constitutive coactivator of peroxisome proliferator-activated receptor gamma | Q96EK7 | hsa:84498 | no | yes | no | no | - | - | - | - | - |

| | | | | | | | | | | | | | |
|---|---|---|---|---|---|---|---|---|---|---|---|---|---|
| HMGCL | ENSG00000117305 | Hydroxymethylglutaryl-CoA lyase, mitochondrial | P35914 | hsa:3155 | yes | yes | no | no | Valine, leucine and isoleucine degradation; Butanoate metabolism; Metabolic pathways; Peroxisome | 3-hydroxyglutaric acid (DB04594) | - | - | - |
| AUH | ENSG00000148090 | Methylglutaconyl-CoA hydratase, mitochondrial | Q13825 | hsa:549 | no | yes | no | no | Valine, leucine and isoleucine degradation; Metabolic pathways | - | - | - | - |
| CBR3 | ENSG00000159231 | Carbonyl reductase [NADPH] 3 | O75828 | hsa:874 | no | no | no | no | Arachidonic acid metabolism; Metabolism of xenobiotics by cytochrome P450; Metabolic pathways | - | - | - | - |
| SHMT1 | ENSG00000176974 | Serine hydroxymethyltransferase, cytosolic | P34896 | hsa:6470 | no | no | no | yes | Glycine, serine and threonine metabolism; Glyoxylate and dicarboxylate metabolism; One carbon pool by folate; Metabolic pathways; Carbon metabolism; Biosynthesis of amino acids; Biosynthesis of cofactors; Antifolate resistance | Pyridoxal phosphate (DB00114), Tetrahydro folic acid (DB00116), Glycine (DB00145), Mimosine (DB01055), Triglu-5-formyl-tetrahydrofolate (DB02067), 5-hydroxymethyl-5,6-dihydrofolic acid (DB02800), N-Pyridoxyl-Glycine-5-Monophosphate (DB02824) | Pyridoxal phosphate is a vitamin available in many formulations to correct vitamin B6 deficiency. Tetrahydrofolic acid is for nutritional supplementation, also for treating dietary shortage or imbalance. Glycine is an amino acid commonly used as a component of total parenteral nutrition and is also used as irrigation during surgery. Mimosine is an antineoplastic alanine-substituted pyridine derivative isolated from Leucena glauca. | In vivo, inhibiting SHMT1 and SHMT2 with the inhibitor SHIN2 decreases SAM levels and increases the expression of some EBV genes. | https://doi.org/10.1016/j.cmet.2022.08.009 |
| ABCB9 | ENSG00000150967 | ABC-type oligopeptide transporter | Q9NP78 | hsa:23457 | no | no | no | no | ABC transporters; Lysosome | - | - | - | - |
| DOC2A | ENSG00000149927 | Double C2-like domain-containing protein alpha | Q14183 | hsa:8448 | no | no | no | no | - | - | - | - | - |
| MADD | ENSG00000110514 | MAP kinase-activating death domain protein | Q8WXG6 | hsa:8567 | no | no | no | no | - | - | - | - | - |
| GIMAP4 | ENSG00000133574 | GTPase IMAP family member 4 | Q9NUV9 | hsa:55303 | no | no | no | yes | - | - | - | - | - |
| QPRT | ENSG00000103485 | Nicotinate-nucleotide pyrophosphorylase [carboxylating] | Q15274 | hsa:23475 | no | no | no | yes | Nicotinate and nicotinamide metabolism; Metabolic pathways; Biosynthesis of cofactors | Niacin (DB00627) | Niacin is a B vitamin used to treat hypertriglyceridemia and pellagra. | UIN serves as the substrate for QPRT. The expression of QPRT, correlating with the catabolic rather than anabolic activity of QUIN, further reinforces the neuroprotective role of oligodendrocytes. | https://doi.org/10.1186/s12974-014-0204-5 |
| MBLAC1 | ENSG00000214309 | Metallo-beta-lactamase domain-containing protein 1 | A4D2B0 | hsa:255374 | no | no | no | no | - | - | - | - | - |
| RNH1 | ENSG00000023191 | Ribonuclease inhibitor | P13489 | hsa:6050 | yes | no | no | no | - | - | - | - | - |

| | | | | | | | | | | | | | |
|---|---|---|---|---|---|---|---|---|---|---|---|---|---|
| LZTFL1 | ENSG00000163818 | Leucine zipper transcription factor-like protein 1 | Q9NQ48 | hsa:54585 | no | no | no | no | - | - | - | - | - |
| PANK2 | ENSG00000125779 | Pantothenate kinase 2, mitochondrial | Q9BZ23 | hsa:80025 | no | no | no | no | Pantothenate and CoA biosynthesis; Metabolic pathways; Biosynthesis of cofactors | - | - | - | - |
| ACOT7 | ENSG00000097021 | Cytosolic acyl coenzyme A thioester hydrolase | O00154 | hsa:11332 | no | no | no | no | Fatty acid elongation; Biosynthesis of unsaturated fatty acids | - | - | - | - |
| TXN | ENSG00000136810 | Thioredoxin | P10599 | hsa:7295 | no | no | no | yes | NOD-like receptor signaling pathway; Parkinson disease; Salmonella infection; Fluid shear stress and atherosclerosis | Phenethyl Isothiocyanate (DB12695) | Phenethyl Isothiocyanate has been used in trials studying the prevention and treatment of Leukemia, Lung Cancer, Tobacco Use Disorder, and Lymphoproliferative Disorders. | The TXN system was reported to be related to the immunogenic cell death of cancer cells, suggesting promising roles of TXN and TXNRD1 for immune modulation | https://doi.org/10.3390/molecules28010107 |
| TREX1 | ENSG00000213689 | Three-prime repair exonuclease 1 | Q9NSU2 | hsa:11277 | no | no | no | yes | Cytosolic DNA-sensing pathway | - | - | The 3’ repair exonuclease 1 (TREX1) gene is the major DNA-specific 3’–5 ’exonuclease of mammalian cells which reduces single- and double-stranded DNA (ssDNA and dsDNA) to prevent undue immune activation mediated by the nucleic acid. TREX1 is also a crucial suppressor of selfrecognition that protects the host from inappropriate autoimmune activations. It has been revealed that TREX1 function is necessary to prevent host DNA accumulating after cell death which could actuate an autoimmune response. | https://doi.org/10.2174/1381612825666190902113218 |
| TRIM65 | ENSG00000141569 | E3 ubiquitin-protein ligase TRIM65 | Q6PJ69 | hsa:201292 | no | no | no | no | - | - | - | - | - |

| | | | | | | | | | | | | | |
|---|---|---|---|---|---|---|---|---|---|---|---|---|---|
| FOLH1 | ENSG00000086205 | Glutamate carboxypeptidase 2 | Q04609 | hsa:2346 | no | no | no | no | Alanine, aspartate and glutamate metabolism; Metabolic pathways; Vitamin digestion and absorption | Capromab pendetide (DB00089, D03372), Glutamic acid (DB00142), (2S)-2-{[HYDROXY(4-IODOBENZYL)PHOSPHORYL]METHYL}PENTANEDIOIC ACID (DB06928), DCFBC (DB07754), Spaglumic acid (DB08835), Acapatamab (D12121), Flotufolastat F18 gallium (D12606<US>), Gallium (68Ga) gozetotide (D12257<US>,DB16019), Capromab pendetide (D04525, DB00089), Iofolastat I 123 (D10070,DB12514), Lutetium (177Lu) vipivotide tetraxetan (D12335<US>,DB16778), Nezastomig (D12566), Piflufolastat (18F) (D12132<US>,DB14805), Technetium Tc99m trofolastat chloride | Capromab pendetide is a monoclonal anti-prostate specific membrane antigen antibody used in imaging kits to target radioactive agents to malignant prostate tissue. Glutamic acid is an amino acid commonly found as a component in total parenteral nutrition. Spaglumic acid is used in allergic eye conditions due to its ability to stabilize mast cells. Ga 68 PSMA-11 is a radiopharmaceutical agent used in the diagnosis of prostate-specific membrane antigen (PSMA) positive lesions in male patients during positron emission tomography. Lutetium Lu-177 vipivotide tetraxetan is a radioligand therapeutic agent used to treat prostate-specific membrane antigen (PSMA)-positive metastatic castration-resistant prostate cancer in adults. Piflufolastat F 18 is | A missense mutation in FOLH1 that leads to greater expression of GCPII. Elevated GCPII signaling also appears to contribute to cognitive deficits in multiple sclerosis, where hippocampal NAAG levels correlate with cognitive abilities | https://doi.org/10.1176/appi.ajp.2020.20101458 |

| | | | | | | | | | | | | | |
|---|---|---|---|---|---|---|---|---|---|---|---|---|---|
| CA4 | ENSG00000167434 | Carbonic anhydrase 4 | P22748 | hsa:762 | no | no | no | no | Nitrogen metabolism; Metabolic pathways; Proximal tubule bicarbonate reclamation | Methyclothiazide (DB00232, D00655), Topiramate (D00537, DB00273), Ethoxzolamide (DB00311, D02441), Bendroflumethiazide (DB00436), Benzthiazide (DB00562), Cyclothiazide (DB00606), Methazolamide (DB00703), Hydroflumethiazide (DB00774), Acetazolamide (DB00819,DG01134), Dorzolamide (DB00869, DG01135), Zonisamide (DB00909), Trichlormethiazide (DB01021), Diclofenamide (DB01144,D00518<US>, D00653<JP/US> D07871), Brinzolamide (DB01194), Ellagic acid (DB08846), Sodium [illegible] | Methyclothiazide is a diuretic drug used to treat hypertension and edema caused by heart failure, renal conditions, treatment with corticosteroids, and estrogen therapy. Topiramate is an anticonvulsant drug used in the control of epilepsy and in the prophylaxis and treatment of migraines. Ethoxzolamide is a sulfonamide used as diuretic and in glaucoma. Bendroflumethiazide is a diuretic used to suppress lactation and to treat hypertension and edema. Benzthiazide is used to treat hypertension and edema. Cyclothiazide is a diuretic. Methazolamide is a carbonic anhydrase inhibitor used to treat open angle glaucoma and acute angle closure glaucoma. Hydroflumethiazide is a thiazide diuretic used to treat hypertension as well as edema due to congestive heart failure [illegible] | - | - |
| RFT1 | ENSG00000163933 | Protein RFT1 homolog | Q96AA3 | hsa:91869 | yes | no | no | no | - | - | - | - | - |
| FAM172A | ENSG00000113391 | Cotranscriptional regulator FAM172A | Q8WUF8 | hsa:83989 | no | no | no | no | - | - | - | - | - |
| TRAP1 | ENSG00000126602 | Heat shock protein 75 kDa, mitochondrial | Q12931 | hsa:10131 | no | no | no | no | Parkinson disease; Pathways of neurodegeneration - multiple diseases | - | - | TRAP1 inhibition in astrocytes significantly reduced TNF-α, CXCL10, and MCP-1 production, indicating an IL-17-mediated TRAP1 upregulation linked to pro-inflammatory cytokine production in astrocytes. TRAP1 knockdown in mice markedly slowed EAE progression, reducing TRAP1, Smad4, p-Smad2/3, GFAP levels, and pro-inflammatory cytokines. Additionally, reduced inflammatory cell infiltration and demyelination in the spinal cord were observed. | https://doi.org/10.3390/ijms25042255 |
| PPAPDC2 | ENSG00000205808 | Polyisoprenoid diphosphate/phosphate phosphohydrolase PLPP6 | Q8IY26 | hsa:403313 | no | no | no | no | - | - | - | - | - |
| CORO1C | ENSG00000110880 | Coronin-1C | Q9ULV4 | hsa:23603 | yes | no | no | no | - | - | - | - | - |
| TTC5 | ENSG00000136319 | Tetratricopeptide repeat protein 5 | Q8N0Z6 | hsa:91875 | yes | no | no | yes | - | - | - | - | - |

| | | | | | | | | | | | | | |
|---|---|---|---|---|---|---|---|---|---|---|---|---|---|
| IFIT5 | ENSG00000152778 | Interferon-induced protein with tetratricopeptide repeats 5 | Q13325 | hsa:24138 | no | no | no | yes | - | - | - | - | - |
| ERAP2 | ENSG00000164308 | Endoplasmic reticulum aminopeptidase 2 | Q6P179 | hsa:64167 | yes | no | no | no | - | - | - | - | - |
| ALCAM | ENSG00000170017 | CD166 antigen | Q13740 | hsa:214 | no | no | no | yes | Cell adhesion molecules | Praluzatamab (D11959, DB16090) | - | Human oligodendrocytes express ALCAM is modulated by inflammation and T cell contact. ALCAM is a ligand for Th17-polarized cells, contributing to their capacity to adhere and induce damage to human oligodendrocytes, and therefore could represent a relevant target for neuroprotection in multiple sclerosis. | https://doi.org/10.1093/brain/awad286 |
| SFXN4 | ENSG00000183605 | Sideroflexin-4 | Q6P4A7 | hsa:119559 | no | no | no | no | - | - | - | A potential role for SFXN3 in the regulation of neurodegeneration pathways. | https://doi.org/10.1111/febs.16377 |
| ULK3 | ENSG00000140474 | Serine/threonine-protein kinase ULK3 | Q6PHR2 | hsa:25989 | no | no | no | no | - | Fostamatinib (DB12010) | Fostamatinib is a spleen tyrosine kinase inhibitor used to treat chronic immune thrombocytopenia after attempting one other treatment. | - | - |
| ASAP3 | ENSG00000088280 | Arf-GAP with SH3 domain, ANK repeat and PH domain-containing protein 3 | Q8TDY4 | hsa:55616 | yes | no | no | no | Endocytosis; FcγRs-mediated phagocytosis | - | - | - | - |
| ANXA6 | ENSG00000197043 | Annexin A6 | P08133 | hsa:309 | yes | no | no | yes | - | - | - | The levels of expression of almost all members of the Annexin A (ANXA) family were induced in EAE mice (ANXA1, 1.91-fold; ANXA2, 1.50-fold; ANXA3, 1.20-fold; ANXA4, 1.30-fold; ANXA6, 1.22-fold; ANXA7, 1.22-fold). Among the ANXA family, ANXA5, ANXA2, and ANXA6 proteins were abundantly expressed in brain capillaries isolated from EAE mice, and the expression levels of these proteins were 71.2-, 67.2-, and 57.3-fold greater than that of Claudin-5, a BBB-specific marker, respectively. | https://doi.org/10.1111/jnc.15578 |
| ENPP4 | ENSG00000001561 | Bis(5'-adenosyl)-triphosphatase ENPP4 | Q9Y6X5 | hsa:22875 | yes | no | no | no | Purine metabolism; Metabolic pathways | - | - | - | - |
| WARS | ENSG00000140105 | Tryptophan--tRNA ligase, cytoplasmic | P23381 | hsa:7453 | no | no | no | no | Aminoacyl-tRNA biosynthesis | Tryptophan (DB00150), Tryptophanyl-5'amp (DB01831), Tryptophan amide (DB04537) | Tryptophan is an amino acid commonly found as a component of total parenteral nutrition. | - | - |

**Table S22. SMR analysis identifying gene expression of relaxed-criteria-based plasma proteins, through integrating eQTL and MS GWAS**

| ProbeID | Probe Chr | Gene | Probe_bp | TopSNP | TopSNP_chr | TopSNP_bp | A1 | A2 | Freq | Beta_GWAS | SE_GWAS | P_GWAS | Beta_eQTL | SE_eQTL | P_eQTL | Beta_SMR | SE_SMR | OR_SMR | 95%CI_SMR | P_SMR | FDR_adjusted_P_SMR | P_HEIDI | $N_{snp}$_HEIDI |
|---|---|---|---|---|---|---|---|---|---|---|---|---|---|---|---|---|---|---|---|---|---|---|---|
| ILMN_1797428 | 1 | FCRL3 | 157648068 | rs3761959 | 1 | 157669278 | T | C | 0.473 | -0.094 | 0.016 | 1.03E-08 | 0.798 | 0.028 | 1.58E-179 | -0.118 | 0.021 | 0.889 | (0.85-0.93) | 1.98E-08 | 6.53E-07 | 7.10E-02 | 20 |
| ILMN_1699599 | 1 | FCRL3 | 157648482 | rs3761959 | 1 | 157669278 | T | C | 0.473 | -0.094 | 0.016 | 1.03E-08 | 0.664 | 0.028 | 1.37E-122 | -0.142 | 0.026 | 0.868 | (0.83-0.91) | 2.65E-08 | 4.37E-07 | 6.83E-02 | 20 |
| ILMN_1704870 | 19 | PGLYRP1 | 46522541 | rs2072563 | 19 | 46526648 | A | G | 0.274 | 0.070 | 0.018 | 1.34E-04 | -0.769 | 0.030 | 3.21E-142 | -0.091 | 0.024 | 0.913 | (0.87-0.96) | 1.59E-04 | 4.37E-07 | 3.88E-01 | 20 |
| ILMN_1727271 | 14 | WARS | 100800398 | rs1570305 | 14 | 100808155 | G | A | 0.189 | -0.068 | 0.020 | 5.67E-04 | 1.037 | 0.032 | 1.08E-226 | -0.066 | 0.019 | 0.936 | (0.9-0.97) | 6.09E-04 | 1.75E-03 | 7.85E-02 | 20 |
| ILMN_2337655 | 14 | WARS | 100800252 | rs1570305 | 14 | 100808155 | G | A | 0.189 | -0.068 | 0.020 | 5.67E-04 | 1.016 | 0.032 | 4.62E-218 | -0.067 | 0.020 | 0.935 | (0.9-0.97) | 6.11E-04 | 4.03E-03 | 1.24E-01 | 20 |
| ILMN_1805449 | 12 | TAPBPL | 6571371 | rs2534709 | 12 | 6568572 | C | T | 0.309 | 0.060 | 0.018 | 6.83E-04 | 0.973 | 0.031 | 2.09E-221 | 0.062 | 0.018 | 1.064 | (1.03-1.1) | 7.33E-04 | 4.03E-03 | 3.10E-01 | 20 |
| ILMN_1685005 | 12 | TNFRSF1A | 6438212 | rs1800692 | 12 | 6442346 | A | G | 0.368 | -0.061 | 0.018 | 5.72E-04 | 0.279 | 0.028 | 6.03E-23 | -0.220 | 0.068 | 0.803 | (0.7-0.92) | 1.15E-03 | 4.03E-03 | 7.98E-05 | 9 |
| ILMN_1751264 | 7 | CCDC126 | 23683651 | rs13238708 | 7 | 23630951 | C | T | 0.233 | -0.062 | 0.019 | 1.51E-03 | 0.436 | 0.032 | 9.41E-42 | -0.142 | 0.046 | 0.868 | (0.79-0.95) | 2.00E-03 | 5.40E-03 | 1.38E-01 | 20 |
| ILMN_1731639 | 4 | CASP3 | 185569645 | rs2696051 | 4 | 185604780 | G | T | 0.228 | -0.064 | 0.020 | 1.11E-03 | -0.308 | 0.033 | 4.85E-21 | 0.207 | 0.067 | 1.230 | (1.08-1.4) | 2.06E-03 | 7.56E-03 | 8.15E-01 | 20 |
| ILMN_2403906 | 4 | ARFIP1 | 153832568 | rs4276265 | 4 | 153695779 | T | C | 0.399 | -0.052 | 0.017 | 1.96E-03 | 0.731 | 0.028 | 2.27E-151 | -0.071 | 0.023 | 0.932 | (0.89-0.97) | 2.11E-03 | 6.95E-03 | 1.63E-01 | 20 |
| ILMN_1703041 | 4 | IDUA | 997408 | rs13101828 | 4 | 965720 | G | A | 0.447 | -0.050 | 0.017 | 2.78E-03 | 0.458 | 0.028 | 1.40E-60 | -0.110 | 0.037 | 0.896 | (0.83-0.96) | 3.25E-03 | 6.95E-03 | 1.67E-01 | 20 |
| ILMN_2388155 | 4 | CASP3 | 185549071 | rs72689263 | 4 | 185590961 | T | C | 0.117 | -0.073 | 0.025 | 2.95E-03 | -0.583 | 0.042 | 2.02E-43 | 0.125 | 0.043 | 1.133 | (1.04-1.23) | 3.66E-03 | 9.75E-03 | 7.23E-02 | 20 |
| ILMN_1768399 | 4 | ARFIP1 | 153791934 | rs11733742 | 4 | 153675656 | T | C | 0.415 | -0.044 | 0.017 | 8.40E-03 | 0.226 | 0.027 | 9.72E-17 | -0.193 | 0.077 | 0.825 | (0.71-0.96) | 1.20E-02 | 1.01E-02 | 1.26E-01 | 10 |
| ILMN_1746517 | 2 | KYNU | 143798222 | rs3845642 | 2 | 143799003 | G | A | 0.268 | -0.043 | 0.019 | 2.07E-02 | 0.539 | 0.031 | 8.73E-68 | -0.080 | 0.035 | 0.923 | (0.86-0.99) | 2.18E-02 | 3.05E-02 | 5.42E-01 | 20 |
| ILMN_2388112 | 1 | CR1 | 207812977 | rs41274768 | 1 | 207782769 | A | G | 0.032 | -0.115 | 0.050 | 2.07E-02 | -0.466 | 0.082 | 1.55E-08 | 0.247 | 0.115 | 1.280 | (1.02-1.6) | 3.22E-02 | 5.15E-02 | 1.27E-01 | 3 |
| ILMN_1812433 | 16 | HP | 72094525 | rs8062041 | 16 | 72088964 | T | C | 0.357 | -0.036 | 0.017 | 3.53E-02 | -0.791 | 0.028 | 8.36E-172 | 0.045 | 0.021 | 1.046 | (1-1.09) | 3.59E-02 | 7.09E-02 | 2.07E-01 | 20 |
| ILMN_1701621 | 22 | TYMP | 50962152 | rs73172252 | 22 | 50973779 | A | G | 0.140 | 0.049 | 0.024 | 4.15E-02 | -0.530 | 0.038 | 4.05E-45 | -0.092 | 0.045 | 0.912 | (0.83-1) | 4.37E-02 | 7.39E-02 | 5.19E-01 | 3 |
| ILMN_1690939 | 22 | TYMP | 50965032 | rs73172252 | 22 | 50973779 | A | G | 0.140 | 0.049 | 0.024 | 4.15E-02 | -0.347 | 0.038 | 2.13E-20 | -0.140 | 0.070 | 0.870 | (0.76-1) | 4.65E-02 | 8.47E-02 | NA | NA |
| ILMN_1742601 | 1 | CR1 | 207793387 | rs11581832 | 1 | 207804238 | C | A | 0.032 | -0.105 | 0.050 | 3.44E-02 | -0.462 | 0.083 | 2.21E-08 | 0.227 | 0.115 | 1.255 | (1-1.57) | 4.78E-02 | 8.30E-02 | 2.25E-03 | 9 |
| ILMN_2109708 | 22 | TYMP | 50964207 | rs73172252 | 22 | 50973779 | A | G | 0.140 | 0.049 | 0.024 | 4.15E-02 | -0.312 | 0.038 | 1.55E-16 | -0.156 | 0.079 | 0.856 | (0.73-1) | 4.78E-02 | 7.89E-02 | NA | NA |
| ILMN_1768469 | 11 | TCN1 | 59620689 | rs12277676 | 11 | 59659006 | T | G | 0.344 | 0.036 | 0.021 | 8.49E-02 | -0.298 | 0.029 | 5.11E-25 | -0.121 | 0.071 | 0.886 | (0.77-1.02) | 8.92E-02 | 7.89E-02 | 5.11E-01 | 18 |
| ILMN_1652237 | 21 | CBR3 | 37518652 | rs11088337 | 21 | 37484426 | G | A | 0.406 | 0.024 | 0.017 | 1.57E-01 | -0.531 | 0.029 | 4.56E-77 | -0.046 | 0.032 | 0.955 | (0.9-1.02) | 1.59E-01 | 1.40E-01 | 4.34E-01 | 20 |
| ILMN_2401978 | 17 | STAT3 | 40465716 | rs2883233 | 17 | 40286827 | C | G | 0.212 | -0.044 | 0.033 | 1.81E-01 | 0.344 | 0.036 | 2.10E-21 | -0.127 | 0.096 | 0.881 | (0.73-1.06) | 1.85E-01 | 2.38E-01 | 5.71E-04 | 20 |
| ILMN_2410986 | 17 | STAT3 | 40466067 | rs2883233 | 17 | 40286827 | C | G | 0.212 | -0.044 | 0.033 | 1.81E-01 | 0.321 | 0.036 | 3.48E-19 | -0.136 | 0.103 | 0.873 | (0.71-1.07) | 1.86E-01 | 2.56E-01 | 6.96E-04 | 20 |
| ILMN_1695110 | 19 | BCAT2 | 49298626 | rs545587 | 19 | 49319664 | A | C | 0.493 | -0.023 | 0.018 | 1.86E-01 | -0.272 | 0.027 | 2.11E-23 | 0.085 | 0.065 | 1.089 | (0.96-1.24) | 1.90E-01 | 2.50E-01 | 1.01E-02 | 20 |
| ILMN_1654560 | 4 | TLR1 | 38798623 | rs73236618 | 4 | 38794041 | C | T | 0.212 | 0.029 | 0.023 | 2.01E-01 | -0.236 | 0.037 | 1.74E-10 | -0.122 | 0.097 | 0.885 | (0.73-1.07) | 2.10E-01 | 2.50E-01 | 2.17E-01 | 11 |
| ILMN_2393497 | 14 | ATXN3 | 92537300 | rs8019051 | 14 | 92519504 | A | G | 0.412 | 0.021 | 0.017 | 2.23E-01 | -0.522 | 0.029 | 8.52E-73 | -0.040 | 0.033 | 0.961 | (0.9-1.02) | 2.24E-01 | 2.66E-01 | 5.00E-03 | 20 |
| ILMN_1731048 | 4 | TLR1 | 38797967 | rs56289835 | 4 | 38811845 | C | G | 0.216 | 0.027 | 0.022 | 2.27E-01 | -0.307 | 0.037 | 8.83E-17 | -0.088 | 0.073 | 0.916 | (0.79-1.06) | 2.32E-01 | 2.74E-01 | 1.72E-02 | 20 |
| ILMN_1796349 | 6 | SMPDL3A | 123130323 | rs9482268 | 6 | 123023776 | A | G | 0.230 | -0.021 | 0.020 | 2.98E-01 | -0.546 | 0.033 | 4.16E-61 | 0.038 | 0.037 | 1.039 | (0.97-1.12) | 2.98E-01 | 2.74E-01 | 6.84E-01 | 20 |
| ILMN_1663618 | 17 | STAT3 | 40465880 | rs1557744 | 17 | 40261284 | A | G | 0.210 | -0.028 | 0.031 | 3.54E-01 | 0.291 | 0.036 | 4.52E-16 | -0.098 | 0.106 | 0.907 | (0.74-1.12) | 3.57E-01 | 3.40E-01 | 4.32E-04 | 20 |
| ILMN_1737514 | 2 | KYNU | 143743551 | rs2278587 | 2 | 143638662 | A | G | 0.062 | -0.032 | 0.038 | 3.95E-01 | 0.909 | 0.062 | 4.52E-49 | -0.035 | 0.042 | 0.965 | (0.89-1.05) | 3.96E-01 | 3.93E-01 | 5.32E-01 | 20 |
| ILMN_1711611 | 1 | CR1 | 207679280 | rs34860402 | 1 | 207856009 | G | A | 0.108 | 0.010 | 0.026 | 6.90E-01 | -0.579 | 0.044 | 1.07E-39 | -0.018 | 0.045 | 0.982 | (0.9-1.07) | 6.90E-01 | 4.22E-01 | 2.18E-02 | 20 |
| ILMN_1769575 | 11 | JAM3 | 134021526 | rs605930 | 11 | 134032102 | A | G | 0.251 | -0.005 | 0.019 | 8.08E-01 | 0.206 | 0.033 | 4.88E-10 | -0.023 | 0.094 | 0.977 | (0.81-1.18) | 8.08E-01 | 7.11E-01 | 6.80E-02 | 5 |
| NA | NA | CD58 | NA | NA | NA | NA | NA | NA | NA | NA | NA | NA | NA | NA | NA | NA | NA | NA | NA | NA | NA | NA | NA |
| NA | NA | ST3GAL6 | NA | NA | NA | NA | NA | NA | NA | NA | NA | NA | NA | NA | NA | NA | NA | NA | NA | NA | NA | NA | NA |
| NA | NA | MMP10 | NA | NA | NA | NA | NA | NA | NA | NA | NA | NA | NA | NA | NA | NA | NA | NA | NA | NA | NA | NA | NA |
| NA | NA | CD59 | NA | NA | NA | NA | NA | NA | NA | NA | NA | NA | NA | NA | NA | NA | NA | NA | NA | NA | NA | NA | NA |
| NA | NA | AHSG | NA | NA | NA | NA | NA | NA | NA | NA | NA | NA | NA | NA | NA | NA | NA | NA | NA | NA | NA | NA | NA |
| NA | NA | FCRL4 | NA | NA | NA | NA | NA | NA | NA | NA | NA | NA | NA | NA | NA | NA | NA | NA | NA | NA | NA | NA | NA |
| NA | NA | IL2RA | NA | NA | NA | NA | NA | NA | NA | NA | NA | NA | NA | NA | NA | NA | NA | NA | NA | NA | NA | NA | NA |
| NA | NA | MMP13 | NA | NA | NA | NA | NA | NA | NA | NA | NA | NA | NA | NA | NA | NA | NA | NA | NA | NA | NA | NA | NA |
| NA | NA | PFKM | NA | NA | NA | NA | NA | NA | NA | NA | NA | NA | NA | NA | NA | NA | NA | NA | NA | NA | NA | NA | NA |
| NA | NA | CD274 | NA | NA | NA | NA | NA | NA | NA | NA | NA | NA | NA | NA | NA | NA | NA | NA | NA | NA | NA | NA | NA |
| NA | NA | C1QL1 | NA | NA | NA | NA | NA | NA | NA | NA | NA | NA | NA | NA | NA | NA | NA | NA | NA | NA | NA | NA | NA |
| NA | NA | TNFSF12 | NA | NA | NA | NA | NA | NA | NA | NA | NA | NA | NA | NA | NA | NA | NA | NA | NA | NA | NA | NA | NA |
| NA | NA | WISP2 | NA | NA | NA | NA | NA | NA | NA | NA | NA | NA | NA | NA | NA | NA | NA | NA | NA | NA | NA | NA | NA |
| NA | NA | CXCL11 | NA | NA | NA | NA | NA | NA | NA | NA | NA | NA | NA | NA | NA | NA | NA | NA | NA | NA | NA | NA | NA |
| NA | NA | CXCL12 | NA | NA | NA | NA | NA | NA | NA | NA | NA | NA | NA | NA | NA | NA | NA | NA | NA | NA | NA | NA | NA |
| NA | NA | ARHGAP1 | NA | NA | NA | NA | NA | NA | NA | NA | NA | NA | NA | NA | NA | NA | NA | NA | NA | NA | NA | NA | NA |

eQTL: expression quantitative trait loci; GWAS: genome-wide association study; SMR: summary-data-based Mendelian randomization; Chr: chromosome; bp: base pair; SNP: single nucleotide polymorphism; A1: the effect allele; A2: the other allele; Freq: frequency of the effect allele; SE: standard error;

**Table S23. SMR analysis examining if both gene expression and relaxed-criteria-based plasma protein expression are causally affected by a single variant, through integrating eQTL and plasma pQTL**

| Exposure_ID | Exposure_chromosome | Exposure_Gene | Exposure_position | Outcome_ID | Outcome_chromosome | Outcome_Gene | Outcome_position | TopSNP | TopSNP_chromosome | TopSNP_position | A1 | A2 | Freq | Beta_Outcome | SE_Outcome | P_Outcome | Beta_Exposure | SE_Exposure | P_Exposure | Beta_SMR | SE_SMR | OR_SMR | 95%CI_SMR | P_SMR | FDR_adjusted_P_SMR | P_HEIDI | $N_{snp}$_HEIDI |
|---|---|---|---|---|---|---|---|---|---|---|---|---|---|---|---|---|---|---|---|---|---|---|---|---|---|---|---|
| ILMN_1805449 | 12 | TAPBPL | 6571371 | SeqId_6364_7 | 12 | TAPBPL | 6601743 | rs2534709 | 12 | 6568572 | C | T | 0.309 | 1.075 | 0.029 | 1.04E-300 | 0.973 | 0.031 | 2.09E-221 | 1.105 | 0.046 | 3.018 | (2.76-3.3) | 1.60E-128 | 2.56E-127 | 8.58E-06 | 20 |
| ILMN_1797428 | 1 | FCRL3 | 157648068 | SeqId_4440_15 | 1 | FCRL3 | 157255396 | rs3761959 | 1 | 157669278 | T | C | 0.473 | 0.712 | 0.019 | 1.04E-300 | 0.798 | 0.028 | 1.58E-179 | 0.891 | 0.039 | 2.438 | (2.26-2.63) | 2.30E-113 | 2.56E-127 | 2.91E-04 | 20 |
| ILMN_1699599 | 1 | FCRL3 | 157648482 | SeqId_4440_15 | 1 | FCRL3 | 157255396 | rs3761959 | 1 | 157669278 | T | C | 0.473 | 0.712 | 0.019 | 1.04E-300 | 0.664 | 0.028 | 1.37E-122 | 1.072 | 0.054 | 2.921 | (2.63-3.25) | 6.72E-88 | 1.84E-112 | 2.37E-03 | 20 |
| ILMN_2403906 | 4 | ARFIP1 | 153832568 | SeqId_13488_3 | 4 | ARFIP1 | 154055176 | rs4276265 | 4 | 153695779 | T | C | 0.399 | 0.402 | 0.017 | 1.02E-127 | 0.731 | 0.028 | 2.27E-151 | 0.550 | 0.031 | 1.733 | (1.63-1.84) | 3.17E-70 | 3.58E-87 | 1.09E-03 | 20 |
| ILMN_1703041 | 4 | IDUA | 997408 | SeqId_3169_70 | 4 | IDUA | 1357325 | rs13101828 | 4 | 965720 | G | A | 0.447 | 0.497 | 0.016 | 2.24E-206 | 0.458 | 0.028 | 1.40E-60 | 1.085 | 0.075 | 2.960 | (2.56-3.43) | 1.78E-47 | 1.27E-69 | 1.59E-05 | 20 |
| ILMN_2388155 | 4 | CASP3 | 185549071 | SeqId_3593_72 | 4 | CASP3 | 185412635 | rs72689263 | 4 | 185590961 | T | C | 0.117 | -0.725 | 0.025 | 2.13E-192 | -0.583 | 0.042 | 2.02E-43 | 1.244 | 0.099 | 3.468 | (2.85-4.21) | 5.87E-36 | 5.68E-47 | 4.95E-04 | 20 |
| ILMN_2388155 | 4 | CASP3 | 185549071 | SeqId_3593_72 | 4 | CASP3 | 185412635 | rs72689263 | 4 | 185590961 | T | C | 0.117 | -0.725 | 0.025 | 2.13E-192 | -0.583 | 0.042 | 2.02E-43 | 1.244 | 0.099 | 3.468 | (2.85-4.21) | 5.87E-36 | 1.34E-35 | 4.95E-04 | 20 |
| ILMN_1751264 | 7 | CCDC126 | 23683651 | SeqId_6388_21 | 7 | CCDC126 | 24084202 | rs13238708 | 7 | 23630951 | C | T | 0.233 | 0.497 | 0.019 | 4.34E-157 | 0.436 | 0.032 | 9.41E-42 | 1.138 | 0.094 | 3.120 | (2.59-3.75) | 1.44E-33 | 1.34E-35 | 4.20E-02 | 20 |
| ILMN_1704870 | 19 | PGLYRP1 | 46522541 | SeqId_3329_14 | 19 | PGLYRP1 | 46728857 | rs2072563 | 19 | 46526648 | A | G | 0.274 | -0.214 | 0.018 | 6.66E-33 | -0.769 | 0.030 | 3.21E-142 | 0.278 | 0.026 | 1.321 | (1.26-1.39) | 3.06E-27 | 2.89E-33 | 5.19E-04 | 20 |
| ILMN_1731639 | 4 | CASP3 | 185569645 | SeqId_3593_72 | 4 | CASP3 | 185412635 | rs2696051 | 4 | 185604780 | G | T | 0.228 | -0.501 | 0.020 | 1.97E-141 | -0.308 | 0.033 | 4.85E-21 | 1.624 | 0.184 | 5.072 | (3.54-7.28) | 1.12E-18 | 5.44E-27 | 3.99E-02 | 20 |
| ILMN_1731639 | 4 | CASP3 | 185569645 | SeqId_3593_72 | 4 | CASP3 | 185412635 | rs2696051 | 4 | 185604780 | G | T | 0.228 | -0.501 | 0.020 | 1.97E-141 | -0.308 | 0.033 | 4.85E-21 | 1.624 | 0.184 | 5.072 | (3.54-7.28) | 1.12E-18 | 1.63E-18 | 3.99E-02 | 20 |
| ILMN_1768399 | 4 | ARFIP1 | 153791934 | SeqId_13488_3 | 4 | ARFIP1 | 154055176 | rs11733742 | 4 | 153675656 | T | C | 0.415 | 0.382 | 0.017 | 1.14E-116 | 0.226 | 0.027 | 9.72E-17 | 1.690 | 0.216 | 5.418 | (3.55-8.28) | 5.61E-15 | 1.63E-18 | 2.59E-01 | 11 |
| ILMN_2388112 | 1 | CR1 | 207812977 | SeqId_19556_12 | 1 | CR1 | 208129798 | rs115126815 | 1 | 207828971 | T | C | 0.0143 | -0.580 | 0.058 | 9.09E-24 | 0.630 | 0.099 | 1.98E-10 | -0.920 | 0.171 | 0.398 | (0.28-0.56) | 7.62E-08 | 7.48E-15 | 4.12E-02 | 5 |
| ILMN_1742601 | 1 | CR1 | 207793387 | SeqId_19556_12 | 1 | CR1 | 208129798 | rs11581832 | 1 | 207804238 | C | A | 0.032 | -0.342 | 0.048 | 6.67E-13 | -0.462 | 0.083 | 2.21E-08 | 0.740 | 0.168 | 2.095 | (1.51-2.91) | 1.01E-05 | 9.38E-08 | 1.69E-07 | 12 |
| ILMN_1812433 | 16 | HP | 72094525 | SeqId_3054_3 | 16 | HP | 72053141 | rs8062041 | 16 | 72088964 | T | C | 0.357 | -0.047 | 0.017 | 6.14E-03 | -0.791 | 0.028 | 8.36E-172 | 0.060 | 0.022 | 1.062 | (1.02-1.11) | 6.39E-03 | 1.16E-05 | 3.40E-88 | 20 |
| ILMN_1701621 | 22 | TYMP | 50962152 | SeqId_11361_73 | 22 | TYMP | 51138753 | rs73172252 | 22 | 50973779 | A | G | 0.140 | 0.005 | 0.023 | 8.43E-01 | -0.530 | 0.038 | 4.05E-45 | -0.009 | 0.043 | 0.991 | (0.91-1.08) | 8.43E-01 | 6.81E-03 | 9.92E-01 | 3 |

eQTL: expression quantitative trait loci; pQTL:protein quantitative trait loci; SMR: summary-data-based Mendelian randomization; Chr: chromosome; SNP: single nucleotide polymorphism; A1: the effect allele; A2: the other allele; Freq: frequency of the effect allele; SE: standard error; HEIDI: heterogeneity in dependent instruments test; Benjamini-Hochberg method was used in FDR

**Table S24. SMR analysis identifying gene expression of relaxed-criteria-based brain proteins, through integrating eQTL and MS GWAS**

| ProbeID | ProbeChr | Gene | Probe_bp | TopSNP | TopSNP_chr | TopSNP_bp | A1 | A2 | Freq | Beta_GWAS | SE_GWAS | P_GWAS | Beta_eQTL | SE_eQTL | P_eQTL | Beta_SMR | SE_SMR | OR_SMR | 95%CI_SMR | P_SMR | FDR_adjusted_P_SMR | P_HEIDI | $N_{snp}$_HEIDI |
|---|---|---|---|---|---|---|---|---|---|---|---|---|---|---|---|---|---|---|---|---|---|---|---|
| ENSG00000123297.20 | 12 | TSFM | 58189199 | rs10877012 | 12 | 58162085 | T | G | 0.316 | -0.115 | 0.018 | 7.60E-11 | -0.899 | 0.033 | 1.94E-168 | 0.128 | 0.020 | 1.136 | (1.09-1.18) | 2.37E-10 | 8.05E-09 | 2.73E-02 | 20 |
| ENSG00000234745.13 | 6 | HLA-B | 31323307 | rs12196740 | 6 | 31321407 | T | C | 0.094 | -0.423 | 0.051 | 1.79E-16 | 0.594 | 0.069 | 7.33E-18 | -0.713 | 0.120 | 0.490 | (0.39-0.62) | 2.66E-09 | 8.05E-09 | 3.29E-02 | 20 |
| ENSG00000131323.16 | 14 | TRAF3 | 103310812 | rs7145882 | 14 | 103255461 | T | C | 0.325 | 0.096 | 0.017 | 2.15E-08 | 0.393 | 0.035 | 6.47E-29 | 0.245 | 0.049 | 1.277 | (1.16-1.41) | 5.60E-07 | 4.52E-08 | 5.46E-02 | 20 |
| ENSG00000163596.17 | 2 | ICA1L | 203687181 | rs72932772 | 2 | 203682304 | G | C | 0.130 | -0.098 | 0.025 | 7.97E-05 | -0.726 | 0.044 | 5.26E-60 | 0.135 | 0.035 | 1.144 | (1.07-1.23) | 1.26E-04 | 6.35E-06 | 1.18E-01 | 20 |
| ENSG00000117305.15 | 1 | HMGCL | 24146742 | rs2076343 | 1 | 24129126 | G | A | 0.329 | 0.065 | 0.017 | 1.64E-04 | 0.639 | 0.031 | 1.22E-91 | 0.102 | 0.027 | 1.107 | (1.05-1.17) | 2.11E-04 | 1.07E-03 | 2.47E-01 | 20 |
| ENSG00000149927.18 | 16 | DOC2A | 30025713 | rs3935873 | 16 | 30018500 | T | C | 0.440 | 0.062 | 0.016 | 1.56E-04 | -0.214 | 0.033 | 4.52E-11 | -0.288 | 0.088 | 0.750 | (0.63-0.89) | 1.04E-03 | 1.43E-03 | 2.20E-02 | 14 |
| ENSG00000141569.12 | 17 | TRIM65 | 73884724 | rs3760128 | 17 | 73886888 | G | A | 0.352 | 0.056 | 0.017 | 0.001171 | 0.748 | 0.031 | 3.36E-125 | 0.075 | 0.023 | 1.078 | (1.03-1.13) | 1.30E-03 | 5.91E-03 | 1.79E-01 | 20 |
| ENSG00000214309.5 | 7 | MBLAC1 | 99725263 | rs4134911 | 7 | 99708388 | C | T | 0.070 | 0.121 | 0.039 | 0.00194 | 1.209 | 0.070 | 1.68E-66 | 0.100 | 0.033 | 1.105 | (1.04-1.18) | 2.29E-03 | 6.31E-03 | 1.48E-01 | 20 |
| ENSG00000163933.10 | 3 | RFT1 | 53143484 | rs2581800 | 3 | 53109352 | A | G | 0.400 | -0.046 | 0.016 | 0.004793 | 0.766 | 0.034 | 6.90E-115 | -0.060 | 0.022 | 0.941 | (0.9-0.98) | 5.12E-03 | 9.72E-03 | 1.82E-01 | 20 |
| ENSG00000148090.12 | 9 | AUH | 94050148 | rs2482338 | 9 | 94037873 | C | T | 0.370 | -0.048 | 0.017 | 0.005398 | 0.393 | 0.030 | 4.11E-38 | -0.121 | 0.044 | 0.886 | (0.81-0.97) | 6.53E-03 | 1.94E-02 | 2.97E-01 | 20 |
| ENSG00000023191.17 | 11 | RNH1 | 500906 | rs4963191 | 11 | 496626 | G | A | 0.311 | -0.050 | 0.018 | 0.005558 | -0.426 | 0.034 | 1.09E-35 | 0.118 | 0.044 | 1.126 | (1.03-1.23) | 6.80E-03 | 2.10E-02 | 1.77E-01 | 20 |
| ENSG00000110514.19 | 11 | MADD | 47321147 | rs35233100 | 11 | 47306630 | T | C | 0.049 | -0.357 | 0.141 | 0.0115 | -1.749 | 0.055 | 1.56E-224 | 0.204 | 0.081 | 1.227 | (1.05-1.44) | 1.18E-02 | 2.10E-02 | 1.91E-02 | 20 |
| ENSG00000001561.7 | 6 | ENPP4 | 46106076 | rs9472703 | 6 | 46117964 | A | G | 0.333 | 0.043 | 0.017 | 0.01258 | 0.671 | 0.030 | 3.43E-112 | 0.064 | 0.026 | 1.066 | (1.01-1.12) | 1.31E-02 | 3.33E-02 | 7.28E-01 | 20 |
| ENSG00000136319.12 | 14 | TTC5 | 20749418 | rs2318864 | 14 | 20767618 | G | A | 0.259 | -0.046 | 0.019 | 0.01334 | -0.475 | 0.032 | 4.96E-50 | 0.097 | 0.040 | 1.101 | (1.02-1.19) | 1.46E-02 | 3.43E-02 | 6.47E-01 | 20 |
| ENSG00000110880.11 | 12 | CORO1C | 109082094 | rs11114042 | 12 | 109124714 | G | A | 0.389 | 0.043 | 0.017 | 0.01288 | -0.343 | 0.028 | 1.17E-34 | -0.125 | 0.051 | 0.882 | (0.8-0.98) | 1.48E-02 | 3.35E-02 | 8.77E-02 | 20 |
| ENSG00000197043.14 | 5 | ANXA6 | 150508806 | rs6859236 | 5 | 150535644 | A | G | 0.510 | -0.040 | 0.016 | 0.01601 | 0.478 | 0.032 | 5.31E-52 | -0.083 | 0.035 | 0.921 | (0.86-0.99) | 1.74E-02 | 3.35E-02 | 6.72E-01 | 20 |
| ENSG00000183605.17 | 10 | SFXN4 | 120912752 | rs10749291 | 10 | 120920588 | T | C | 0.564 | 0.039 | 0.017 | 0.01887 | -0.686 | 0.026 | 9.31E-153 | -0.057 | 0.024 | 0.945 | (0.90-0.99) | 1.93E-02 | 3.69E-02 | 7.01E-01 | 20 |
| ENSG00000140105.18 | 14 | WARS | 100821634 | rs2273803 | 14 | 100842778 | C | T | 0.479 | 0.039 | 0.016 | 0.01763 | 0.255 | 0.028 | 2.61E-19 | 0.152 | 0.066 | 1.164 | (1.02-1.33) | 2.18E-02 | 3.87E-02 | 1.01E-01 | 20 |
| ENSG00000159231.6 | 21 | CBR3 | 37513118 | rs4816521 | 21 | 37510738 | G | A | 0.355 | 0.038 | 0.017 | 0.02408 | -0.614 | 0.032 | 6.50E-84 | -0.062 | 0.028 | 0.939 | (0.89-0.99) | 2.50E-02 | 4.11E-02 | 9.53E-01 | 20 |
| ENSG00000088280.19 | 1 | ASAP3 | 23783058 | rs61778877 | 1 | 23774016 | T | C | 0.144 | -0.054 | 0.025 | 0.03166 | -0.797 | 0.047 | 1.00E-65 | 0.067 | 0.032 | 1.070 | (1.01-1.14) | 3.30E-02 | 4.48E-02 | 5.95E-02 | 20 |
| ENSG00000164308.17 | 5 | ERAP2 | 96233548 | rs2910686 | 5 | 96252589 | C | T | 0.424 | -0.034 | 0.016 | 0.04058 | 1.235 | 0.018 | 0 | -0.027 | 0.013 | 0.973 | (0.95-1.00) | 4.07E-02 | 5.61E-02 | 6.94E-01 | 20 |
| ENSG00000167434.10 | 17 | CA4 | 58237782 | rs9675236 | 17 | 58226800 | T | C | 0.050 | 0.076 | 0.039 | 0.053 | -0.909 | 0.077 | 2.49E-32 | -0.084 | 0.044 | 0.920 | (0.84-1.00) | 5.62E-02 | 6.59E-02 | 6.21E-01 | 20 |
| ENSG00000152778.10 | 10 | IFIT5 | 91177844 | rs303208 | 10 | 91165833 | T | C | 0.278 | 0.034 | 0.019 | 0.07677 | -0.910 | 0.034 | 1.25E-161 | -0.037 | 0.021 | 0.964 | (0.92-1.00) | 7.74E-02 | 8.68E-02 | 2.32E-01 | 20 |
| ENSG00000150967.18 | 12 | ABCB9 | 123435847 | rs7953894 | 12 | 123404966 | A | C | 0.507 | -0.025 | 0.017 | 0.1341 | -0.180 | 0.032 | 2.65E-08 | 0.141 | 0.097 | 1.151 | (0.95-1.39) | 1.48E-01 | 1.14E-01 | 8.19E-03 | 20 |
| ENSG00000112584.14 | 6 | FAM120B | 170657973 | rs9459991 | 6 | 170641014 | A | G | 0.131 | 0.036 | 0.026 | 0.162 | 0.577 | 0.045 | 6.98E-37 | 0.063 | 0.045 | 1.065 | (0.97-1.16) | 1.65E-01 | 2.10E-01 | 3.44E-01 | 20 |
| ENSG00000176974.20 | 17 | SHMT1 | 18249026 | rs638416 | 17 | 18266975 | G | C | 0.388 | -0.023 | 0.018 | 0.2052 | -0.522 | 0.034 | 2.67E-54 | 0.044 | 0.035 | 1.045 | (0.98-1.12) | 2.07E-01 | 2.24E-01 | 1.65E-03 | 20 |
| ENSG00000133574.10 | 7 | GIMAP4 | 150267749 | rs2204129 | 7 | 150251439 | A | G | 0.391 | -0.016 | 0.017 | 0.3481 | 0.237 | 0.030 | 2.70E-15 | -0.066 | 0.071 | 0.936 | (0.81-1.08) | 3.51E-01 | 2.70E-01 | 9.61E-05 | 20 |
| ENSG00000136810.13 | 9 | TXN | 113012438 | rs4135220 | 9 | 113007289 | T | G | 0.521 | -0.015 | 0.017 | 0.3564 | -0.475 | 0.028 | 4.42E-63 | 0.032 | 0.035 | 1.033 | (0.96-1.11) | 3.57E-01 | 4.34E-01 | 1.51E-01 | 20 |
| ENSG00000170017.12 | 3 | ALCAM | 105190748 | rs6797043 | 3 | 105108867 | C | T | 0.228 | 0.017 | 0.019 | 0.375 | -0.302 | 0.042 | 5.84E-13 | -0.056 | 0.064 | 0.945 | (0.83-1.07) | 3.79E-01 | 4.34E-01 | 1.02E-02 | 20 |
| ENSG00000125779.23 | 20 | PANK2 | 3890010 | rs241594 | 20 | 3899640 | A | G | 0.298 | 0.011 | 0.018 | 0.5431 | 0.519 | 0.030 | 6.37E-68 | 0.021 | 0.035 | 1.022 | (0.95-1.09) | 5.43E-01 | 4.44E-01 | 1.94E-01 | 20 |
| ENSG00000177000.13 | 1 | MTHFR | 11856146 | rs1476413 | 1 | 11852300 | T | C | 0.277 | -0.009 | 0.018 | 0.6186 | 0.595 | 0.031 | 6.21E-83 | -0.015 | 0.031 | 0.985 | (0.93-1.05) | 6.19E-01 | 6.16E-01 | 1.79E-02 | 20 |
| ENSG00000103485.19 | 16 | QPRT | 29692310 | rs12596308 | 16 | 29691196 | T | C | 0.244 | 0.005 | 0.019 | 0.7924 | 0.923 | 0.036 | 1.38E-144 | 0.006 | 0.021 | 1.006 | (0.96-1.05) | 7.92E-01 | 6.79E-01 | 1.53E-02 | 20 |
| ENSG00000126602.11 | 16 | TRAP1 | 3734597 | rs4786429 | 16 | 3728517 | C | T | 0.284 | 0.005 | 0.020 | 0.8091 | -0.806 | 0.034 | 2.65E-127 | -0.006 | 0.024 | 0.994 | (0.95-1.04) | 8.09E-01 | 8.34E-01 | 4.64E-01 | 20 |
| ENSG00000086205.18 | 11 | FOLH1 | 49198417 | rs71481219 | 11 | 49931536 | A | G | 0.062 | 0.008 | 0.061 | 0.8988 | 21.280 | 0.734 | 9.45E-185 | 0.000 | 0.003 | 1.000 | (0.99-1.01) | 8.99E-01 | 8.34E-01 | 1.39E-02 | 14 |
| NA | 8 | ZC2HC1A | NA | NA | NA | NA | NA | NA | NA | NA | NA | NA | NA | NA | NA | NA | NA | NA | NA | NA | NA | NA | NA |
| NA | 3 | LZTFL1 | NA | NA | NA | NA | NA | NA | NA | NA | NA | NA | NA | NA | NA | NA | NA | NA | NA | NA | NA | NA | NA |
| NA | 1 | ACOT7 | NA | NA | NA | NA | NA | NA | NA | NA | NA | NA | NA | NA | NA | NA | NA | NA | NA | NA | NA | NA | NA |
| NA | 3 | TREX1 | NA | NA | NA | NA | NA | NA | NA | NA | NA | NA | NA | NA | NA | NA | NA | NA | NA | NA | NA | NA | NA |
| NA | 5 | FAM172A | NA | NA | NA | NA | NA | NA | NA | NA | NA | NA | NA | NA | NA | NA | NA | NA | NA | NA | NA | NA | NA |
| NA | 9 | PPAPDC2 | NA | NA | NA | NA | NA | NA | NA | NA | NA | NA | NA | NA | NA | NA | NA | NA | NA | NA | NA | NA | NA |
| NA | 15 | ULK3 | NA | NA | NA | NA | NA | NA | NA | NA | NA | NA | NA | NA | NA | NA | NA | NA | NA | NA | NA | NA | NA |

eQTL: expression quantitative trait loci; GWAS: genome-wide association study; SMR: summary-data-based Mendelian randomization; Chr: chromosome; bp: base pair; SNP: single nucleotide polymorphism; A1: the effect allele; A2: the other allele; Freq: frequency of the effect allele; SE: standard error; HEIDI: heterogeneity in dependent instruments test; Benjamini-Hochberg method was used in FDR adjustment.

**Table S25. SMR analysis examining if both gene expression and relaxed-criteria-based brain protein expression are causally affected by a single variant, through integrating eQTL and plasma pQTL**

| Exposure_ID | Exposure_chromosome | Exposure_Gene | Exposure_position | Outcome_ID | Outcome_chromosome | Outcome_Gene | Outcome_position | TopSNP | TopSNP_chromosome | TopSNP_position | A1 | A2 | Freq | Beta_Outcome | SE_Outcome | P_Outcome | Beta_Exposure | SE_Exposure | P_Exposure | Beta_SMR | SE_SMR | OR_SMR | 95%CI_SMR | P_SMR | FDR_adjusted_P_SMR | P_HEIDI | $N_{snp}$_HEIDI |
|---|---|---|---|---|---|---|---|---|---|---|---|---|---|---|---|---|---|---|---|---|---|---|---|---|---|---|---|
| ENSG00000159231.6 | 21 | CBR3 | 37513118 | ENSG00000159231 | 21 | CBR3 | 37443658 | rs879894 | 21 | 37508643 | T | G | 0.355 | -0.188 | 1.03E-02 | 1.18E-74 | -0.614 | 3.16E-02 | 6.50E-84 | 0.307 | 2.30E-02 | 1.359 | (1.30-1.42) | 2.10E-40 | 2.94E-39 | 7.95E-01 | 20 |
| ENSG00000164308.17 | 5 | ERAP2 | 96233548 | ENSG00000164308 | 5 | ERAP2 | 96320495 | rs38034 | 5 | 96322419 | T | C | 0.414 | 0.443 | 3.75E-02 | 2.96E-32 | 1.230 | 1.90E-02 | 0.00E+00 | 0.360 | 3.10E-02 | 1.434 | (1.35-1.52) | 2.87E-31 | 2.94E-39 | 1.75E-01 | 20 |
| ENSG00000163933.10 | 3 | RFT1 | 53143484 | ENSG00000163933 | 3 | RFT1 | 53140603 | rs891368 | 3 | 53123273 | A | G | 0.402 | 0.097 | 1.01E-02 | 9.21E-22 | 0.762 | 3.35E-02 | 2.10E-114 | 0.127 | 1.44E-02 | 1.136 | (1.10-1.17) | 1.02E-18 | 2.01E-30 | 4.86E-01 | 14 |
| ENSG00000023191.17 | 11 | RNH1 | 500906 | ENSG00000023191 | 11 | RNH1 | 494662 | rs7930593 | 11 | 502013 | A | G | 0.345 | -0.090 | 9.04E-03 | 2.70E-23 | -0.417 | 3.36E-02 | 3.18E-35 | 0.216 | 2.78E-02 | 1.241 | (1.17-1.31) | 8.95E-15 | 4.78E-18 | 1.49E-01 | 20 |
| ENSG00000136319.12 | 14 | TTC5 | 20749418 | ENSG00000136319 | 14 | TTC5 | 20639015 | rs2318864 | 14 | 20767618 | G | A | 0.259 | -0.060 | 7.10E-03 | 3.38E-17 | -0.475 | 3.20E-02 | 4.96E-50 | 0.126 | 1.72E-02 | 1.134 | (1.10-1.17) | 2.21E-13 | 3.13E-14 | 6.61E-01 | 20 |
| ENSG00000117305.15 | 1 | HMGCL | 24146742 | ENSG00000117305 | 1 | HMGCL | 24133612 | rs2076343 | 1 | 24129126 | G | A | 0.329 | 0.051 | 6.86E-03 | 1.72E-13 | 0.639 | 3.15E-02 | 1.22E-91 | 0.079 | 1.14E-02 | 1.082 | (1.06-1.11) | 4.30E-12 | 6.18E-13 | 5.54E-01 | 13 |
| ENSG00000110880.11 | 12 | CORO1C | 109082094 | ENSG00000110880 | 12 | CORO1C | 108949739 | rs7300215 | 12 | 109089394 | A | G | 0.348 | -0.043 | 5.18E-03 | 9.64E-17 | -0.345 | 2.91E-02 | 2.29E-32 | 0.125 | 1.84E-02 | 1.133 | (1.09-1.17) | 1.03E-11 | 1.00E-11 | 8.27E-02 | 11 |
| ENSG00000197043.14 | 5 | ANXA6 | 150508806 | ENSG00000197043 | 5 | ANXA6 | 150458146 | rs6859236 | 5 | 150535644 | A | G | 0.510 | 0.045 | 6.78E-03 | 3.57E-11 | 0.478 | 3.15E-02 | 5.31E-52 | 0.094 | 1.55E-02 | 1.098 | (1.07-1.13) | 1.29E-09 | 2.06E-11 | 9.62E-01 | 12 |
| ENSG00000001561.7 | 6 | ENPP4 | 46106076 | ENSG00000001561 | 6 | ENPP4 | 46128745 | rs9472701 | 6 | 46116449 | G | A | 0.332 | 0.055 | 8.95E-03 | 6.73E-10 | 0.670 | 2.98E-02 | 1.10E-111 | 0.082 | 1.39E-02 | 1.086 | (1.06-1.12) | 2.66E-09 | 2.26E-09 | 8.28E-02 | 17 |
| ENSG00000148090.12 | 9 | AUH | 94050148 | ENSG00000148090 | 9 | AUH | 93936592 | rs2482338 | 9 | 94037873 | C | T | 0.370 | 0.039 | 6.21E-03 | 4.36E-10 | 0.393 | 3.05E-02 | 4.11E-38 | 0.099 | 1.75E-02 | 1.104 | (1.07-1.14) | 1.93E-08 | 4.13E-09 | 1.77E-03 | 20 |
| ENSG00000140105.18 | 14 | WARS | 100821634 | ENSG00000140105 | 14 | WARS | 100820980 | rs12897338 | 14 | 100844368 | T | C | 0.478 | 0.061 | 8.81E-03 | 3.71E-12 | 0.254 | 2.85E-02 | 3.75E-19 | 0.240 | 4.38E-02 | 1.272 | (1.17-1.39) | 4.09E-08 | 2.70E-08 | 8.73E-01 | 17 |
| ENSG00000088280.19 | 1 | ASAP3 | 23783058 | ENSG00000088280 | 1 | ASAP3 | 23884661 | rs1555024 | 1 | 23786203 | C | T | 0.308 | -0.056 | 9.88E-03 | 1.13E-08 | -0.509 | 3.63E-02 | 1.11E-44 | 0.111 | 2.10E-02 | 1.117 | (1.07-1.16) | 1.23E-07 | 5.21E-08 | 4.56E-01 | 14 |
| ENSG00000131323.16 | 14 | TRAF3 | 103310812 | ENSG00000131323 | 14 | TRAF3 | 103404159 | rs7145882 | 14 | 103255461 | T | C | 0.325 | 0.030 | 6.03E-03 | 5.27E-07 | 0.393 | 3.52E-02 | 6.47E-29 | 0.077 | 1.68E-02 | 1.080 | (1.05-1.12) | 4.76E-06 | 1.44E-07 | 9.62E-01 | 19 |
| ENSG00000141569.12 | 17 | TRIM65 | 73884724 | ENSG00000141569 | 17 | TRIM65 | 73785718 | rs3760128 | 17 | 73886888 | G | A | 0.352 | 0.065 | 1.83E-02 | 3.83E-04 | 0.748 | 3.14E-02 | 3.36E-125 | 0.087 | 2.47E-02 | 1.091 | (1.04-1.14) | 4.44E-04 | 5.12E-06 | 2.79E-06 | 20 |

eQTL: expression quantitative trait loci; pQTL:protein quantitative trait loci; SMR: summary-data-based Mendelian randomization; Chr: chromosome; SNP: single nucleotide polymorphism; A1: the effect allele; A2: the other allele; Freq: frequency of the effect allele; SE: standard error; HEIDI: heterogeneity in dependent instruments test; Benjamini-Hochberg method was used in FDR

**Table S26. Protein-protein interaction analysis of relaxed-criteria-based plasma proteins with multiple sclerosis drug targets (minimum combined score of 0.4)**

| MS drug | MS drug target | armacological action of MS | Potential target | ıood_on_ch | gene_fusion | netic_coocc | homology | coexpressio | y_determin | base_annot | nated_textm | combined_score |
|---|---|---|---|---|---|---|---|---|---|---|---|---|
| Alemtuzumab | CD52 | Antibody | CD59 | 0 | 0 | 0 | 0 | 0 | 0 | 0 | 0.431 | 0.431 |
| Alemtuzumab | CD52 | Antibody | IL2RA | 0 | 0 | 0 | 0 | 0.088 | 0 | 0 | 0.623 | 0.641 |
| Alemtuzumab | CD52 | Antibody | CD274 | 0 | 0 | 0 | 0 | 0.060 | 0 | 0 | 0.434 | 0.445 |
| Alemtuzumab | FCGR1A | Unknown | CR1 | 0 | 0 | 0 | 0 | 0.085 | 0 | 0 | 0.571 | 0.590 |
| Alemtuzumab | FCGR1A | Unknown | STAT3 | 0 | 0 | 0 | 0 | 0.061 | 0 | 0 | 0.404 | 0.417 |
| Alemtuzumab | FCGR1A | Unknown | TNFRSF1A | 0 | 0 | 0 | 0 | 0.060 | 0 | 0 | 0.575 | 0.583 |
| Alemtuzumab | FCGR1A | Unknown | CD274 | 0 | 0 | 0 | 0 | 0.126 | 0.060 | 0 | 0.678 | 0.712 |
| Alemtuzumab | FCGR1A | Unknown | TLR1 | 0 | 0 | 0 | 0 | 0.192 | 0.095 | 0 | 0.32 | 0.459 |
| Alemtuzumab | FCGR2A | Unknown | CD58 | 0 | 0 | 0 | 0 | 0.144 | 0 | 0 | 0.366 | 0.434 |
| Alemtuzumab | FCGR2A | Unknown | CR1 | 0 | 0 | 0 | 0 | 0.261 | 0 | 0 | 0.729 | 0.791 |
| Alemtuzumab | FCGR2A | Unknown | STAT3 | 0 | 0 | 0 | 0 | 0.111 | 0 | 0 | 0.37 | 0.416 |
| Alemtuzumab | FCGR2A | Unknown | TNFRSF1A | 0 | 0 | 0 | 0 | 0.068 | 0 | 0 | 0.666 | 0.675 |
| Alemtuzumab | FCGR2A | Unknown | CASP3 | 0 | 0 | 0 | 0 | 0.078 | 0 | 0 | 0.568 | 0.584 |
| Alemtuzumab | FCGR2A | Unknown | CD274 | 0 | 0 | 0 | 0 | 0.088 | 0 | 0 | 0.525 | 0.549 |
| Alemtuzumab | FCGR2A | Unknown | TLR1 | 0 | 0 | 0 | 0 | 0.35 | 0 | 0 | 0.37 | 0.573 |
| Alemtuzumab | FCGR2B | Unknown | TNFRSF1A | 0 | 0 | 0 | 0 | 0.049 | 0 | 0 | 0.486 | 0.490 |
| Alemtuzumab | FCGR2B | Unknown | CR1 | 0 | 0 | 0 | 0 | 0.218 | 0 | 0 | 0.711 | 0.765 |
| Alemtuzumab | FCGR2B | Unknown | CASP3 | 0 | 0 | 0 | 0 | 0.078 | 0 | 0 | 0.551 | 0.568 |
| Alemtuzumab | FCGR2B | Unknown | CD274 | 0 | 0 | 0 | 0 | 0.099 | 0 | 0 | 0.692 | 0.711 |
| Alemtuzumab | FCGR2B | Unknown | TLR1 | 0 | 0 | 0 | 0 | 0.256 | 0 | 0 | 0.301 | 0.459 |
| Alemtuzumab | FCGR3A | Unknown | CD58 | 0 | 0 | 0 | 0 | 0.086 | 0 | 0 | 0.596 | 0.614 |
| Alemtuzumab | FCGR3A | Unknown | CD59 | 0 | 0 | 0 | 0 | 0 | 0 | 0 | 0.474 | 0.474 |
| Alemtuzumab | FCGR3A | Unknown | CR1 | 0 | 0 | 0 | 0 | 0.123 | 0 | 0 | 0.720 | 0.744 |
| Alemtuzumab | FCGR3A | Unknown | FCRL3 | 0 | 0 | 0 | 0.613 | 0.097 | 0.176 | 0 | 0.262 | 0.403 |
| Alemtuzumab | FCGR3A | Unknown | STAT3 | 0 | 0 | 0 | 0 | 0.077 | 0 | 0 | 0.664 | 0.676 |
| Alemtuzumab | FCGR3A | Unknown | TNFRSF1A | 0 | 0 | 0 | 0 | 0.062 | 0 | 0 | 0.64 | 0.647 |
| Alemtuzumab | FCGR3A | Unknown | IL2RA | 0 | 0 | 0 | 0 | 0.083 | 0 | 0 | 0.554 | 0.573 |
| Alemtuzumab | FCGR3A | Unknown | CASP3 | 0 | 0 | 0 | 0 | 0.068 | 0 | 0 | 0.439 | 0.455 |
| Alemtuzumab | FCGR3A | Unknown | CD274 | 0 | 0 | 0 | 0 | 0.127 | 0 | 0 | 0.866 | 0.878 |
| Alemtuzumab | FCGR3A | Unknown | CXCL11 | 0 | 0 | 0 | 0 | 0.138 | 0 | 0 | 0.419 | 0.478 |
| Alemtuzumab | FCGR3A | Unknown | CXCL12 | 0 | 0 | 0 | 0 | 0.110 | 0 | 0 | 0.512 | 0.547 |
| Alemtuzumab | FCGR3A | Unknown | TLR1 | 0 | 0 | 0 | 0 | 0.256 | 0 | 0 | 0.425 | 0.554 |
| Alemtuzumab | FCGR3B | Unknown | CD58 | 0 | 0 | 0 | 0 | 0.106 | 0 | 0 | 0.596 | 0.623 |
| Alemtuzumab | FCGR3B | Unknown | CD59 | 0 | 0 | 0 | 0 | 0 | 0 | 0 | 0.448 | 0.448 |
| Alemtuzumab | FCGR3B | Unknown | CR1 | 0 | 0 | 0 | 0 | 0.142 | 0 | 0 | 0.723 | 0.752 |
| Alemtuzumab | FCGR3B | Unknown | STAT3 | 0 | 0 | 0 | 0 | 0.086 | 0 | 0 | 0.660 | 0.676 |
| Alemtuzumab | FCGR3B | Unknown | TNFRSF1A | 0 | 0 | 0 | 0 | 0.064 | 0 | 0 | 0.419 | 0.432 |
| Alemtuzumab | FCGR3B | Unknown | IL2RA | 0 | 0 | 0 | 0 | 0.062 | 0 | 0 | 0.541 | 0.551 |
| Alemtuzumab | FCGR3B | Unknown | CASP3 | 0 | 0 | 0 | 0 | 0.068 | 0 | 0 | 0.439 | 0.455 |
| Alemtuzumab | FCGR3B | Unknown | CD274 | 0 | 0 | 0 | 0 | 0.127 | 0 | 0 | 0.867 | 0.879 |
| Alemtuzumab | FCGR3B | Unknown | CXCL11 | 0 | 0 | 0 | 0 | 0.134 | 0 | 0 | 0.419 | 0.475 |
| Alemtuzumab | FCGR3B | Unknown | CXCL12 | 0 | 0 | 0 | 0 | 0.098 | 0 | 0 | 0.512 | 0.541 |

| | | | | | | | | | | | | |
|---|---|---|---|---|---|---|---|---|---|---|---|---|
| Alemtuzumab | FCGR3B | Unknown | TLR1 | 0 | 0 | 0 | 0 | 0.329 | 0 | 0 | 0.426 | 0.598 |
| Cladribine | PNP | Inhibitor | TYMP | 0.120 | 0 | 0 | 0 | 0.042 | 0 | 0.900 | 0.642 | 0.965 |
| Cladribine | RRM2B | Inhibitor | TYMP | 0 | 0 | 0 | 0 | 0.042 | 0 | 0 | 0.798 | 0.798 |
| Daclizumab | C1QA | Unknown | CD59 | 0 | 0 | 0 | 0 | 0.049 | 0 | 0 | 0.409 | 0.414 |
| Daclizumab | C1QB | Unknown | CR1 | 0 | 0 | 0 | 0 | 0.094 | 0.407 | 0 | 0.125 | 0.489 |
| Daclizumab | C1R | Unknown | CR1 | 0 | 0 | 0 | 0 | 0.060 | 0.046 | 0 | 0.511 | 0.523 |
| Daclizumab | C1R | Unknown | CD59 | 0 | 0 | 0 | 0 | 0.079 | 0 | 0 | 0.640 | 0.654 |
| Daclizumab | C1R | Unknown | C1QL1 | 0 | 0 | 0 | 0 | 0.042 | 0.051 | 0 | 0.427 | 0.434 |
| Daclizumab | C1R | Unknown | CXCL12 | 0 | 0 | 0 | 0 | 0.245 | 0 | 0 | 0.444 | 0.562 |
| Daclizumab | FCGR1A | Unknown | TNFRSF1A | 0 | 0 | 0 | 0 | 0.060 | 0 | 0 | 0.575 | 0.583 |
| Daclizumab | FCGR1A | Unknown | STAT3 | 0 | 0 | 0 | 0 | 0.061 | 0 | 0 | 0.404 | 0.417 |
| Daclizumab | FCGR1A | Unknown | CR1 | 0 | 0 | 0 | 0 | 0.085 | 0 | 0 | 0.571 | 0.590 |
| Daclizumab | FCGR1A | Unknown | CD274 | 0 | 0 | 0 | 0 | 0.126 | 0.06 | 0 | 0.678 | 0.712 |
| Daclizumab | FCGR1A | Unknown | TLR1 | 0 | 0 | 0 | 0 | 0.192 | 0.095 | 0 | 0.32 | 0.459 |
| Daclizumab | FCGR2A | Unknown | CASP3 | 0 | 0 | 0 | 0 | 0.078 | 0 | 0 | 0.568 | 0.584 |
| Daclizumab | FCGR2A | Unknown | CD274 | 0 | 0 | 0 | 0 | 0.088 | 0 | 0 | 0.525 | 0.549 |
| Daclizumab | FCGR2A | Unknown | TNFRSF1A | 0 | 0 | 0 | 0 | 0.068 | 0 | 0 | 0.666 | 0.675 |
| Daclizumab | FCGR2A | Unknown | STAT3 | 0 | 0 | 0 | 0 | 0.111 | 0 | 0 | 0.370 | 0.416 |
| Daclizumab | FCGR2A | Unknown | CD58 | 0 | 0 | 0 | 0 | 0.144 | 0 | 0 | 0.366 | 0.434 |
| Daclizumab | FCGR2A | Unknown | CR1 | 0 | 0 | 0 | 0 | 0.261 | 0 | 0 | 0.729 | 0.791 |
| Daclizumab | FCGR2A | Unknown | TLR1 | 0 | 0 | 0 | 0 | 0.350 | 0 | 0 | 0.370 | 0.573 |
| Daclizumab | FCGR2B | Unknown | TNFRSF1A | 0 | 0 | 0 | 0 | 0.049 | 0 | 0 | 0.486 | 0.490 |
| Daclizumab | FCGR2B | Unknown | CR1 | 0 | 0 | 0 | 0 | 0.218 | 0 | 0 | 0.711 | 0.765 |
| Daclizumab | FCGR2B | Unknown | CASP3 | 0 | 0 | 0 | 0 | 0.078 | 0 | 0 | 0.551 | 0.568 |
| Daclizumab | FCGR2B | Unknown | CD274 | 0 | 0 | 0 | 0 | 0.099 | 0 | 0 | 0.692 | 0.711 |
| Daclizumab | FCGR2B | Unknown | TLR1 | 0 | 0 | 0 | 0 | 0.256 | 0 | 0 | 0.301 | 0.459 |
| Daclizumab | FCGR3A | Unknown | STAT3 | 0 | 0 | 0 | 0 | 0.077 | 0 | 0 | 0.664 | 0.676 |
| Daclizumab | FCGR3A | Unknown | CR1 | 0 | 0 | 0 | 0 | 0.123 | 0 | 0 | 0.720 | 0.744 |
| Daclizumab | FCGR3A | Unknown | FCRL3 | 0 | 0 | 0 | 0.613 | 0.097 | 0.176 | 0 | 0.262 | 0.403 |
| Daclizumab | FCGR3A | Unknown | CD59 | 0 | 0 | 0 | 0 | 0 | 0 | 0 | 0.474 | 0.474 |
| Daclizumab | FCGR3A | Unknown | CD58 | 0 | 0 | 0 | 0 | 0.086 | 0 | 0 | 0.596 | 0.614 |
| Daclizumab | FCGR3A | Unknown | IL2RA | 0 | 0 | 0 | 0 | 0.083 | 0 | 0 | 0.554 | 0.573 |
| Daclizumab | FCGR3A | Unknown | CASP3 | 0 | 0 | 0 | 0 | 0.068 | 0 | 0 | 0.439 | 0.455 |
| Daclizumab | FCGR3A | Unknown | CD274 | 0 | 0 | 0 | 0 | 0.127 | 0 | 0 | 0.866 | 0.878 |
| Daclizumab | FCGR3A | Unknown | CXCL11 | 0 | 0 | 0 | 0 | 0.138 | 0 | 0 | 0.419 | 0.478 |
| Daclizumab | FCGR3A | Unknown | CXCL12 | 0 | 0 | 0 | 0 | 0.110 | 0 | 0 | 0.512 | 0.547 |
| Daclizumab | FCGR3A | Unknown | TLR1 | 0 | 0 | 0 | 0 | 0.256 | 0 | 0 | 0.425 | 0.554 |
| Daclizumab | FCGR3B | Unknown | TNFRSF1A | 0 | 0 | 0 | 0 | 0.064 | 0 | 0 | 0.419 | 0.432 |
| Daclizumab | FCGR3B | Unknown | STAT3 | 0 | 0 | 0 | 0 | 0.086 | 0 | 0 | 0.660 | 0.676 |
| Daclizumab | FCGR3B | Unknown | CR1 | 0 | 0 | 0 | 0 | 0.142 | 0 | 0 | 0.723 | 0.752 |
| Daclizumab | FCGR3B | Unknown | CD58 | 0 | 0 | 0 | 0 | 0.106 | 0 | 0 | 0.596 | 0.623 |
| Daclizumab | FCGR3B | Unknown | CD59 | 0 | 0 | 0 | 0 | 0 | 0 | 0 | 0.448 | 0.448 |
| Daclizumab | FCGR3B | Unknown | IL2RA | 0 | 0 | 0 | 0 | 0.062 | 0 | 0 | 0.541 | 0.551 |
| Daclizumab | FCGR3B | Unknown | CASP3 | 0 | 0 | 0 | 0 | 0.068 | 0 | 0 | 0.439 | 0.455 |

| | | | | | | | | | | | | |
|---|---|---|---|---|---|---|---|---|---|---|---|---|
| Daclizumab | FCGR3B | Unknown | CD274 | 0 | 0 | 0 | 0 | 0.127 | 0 | 0 | 0.867 | 0.879 |
| Daclizumab | FCGR3B | Unknown | CXCL11 | 0 | 0 | 0 | 0 | 0.134 | 0 | 0 | 0.419 | 0.475 |
| Daclizumab | FCGR3B | Unknown | CXCL12 | 0 | 0 | 0 | 0 | 0.098 | 0 | 0 | 0.512 | 0.541 |
| Daclizumab | FCGR3B | Unknown | TLR1 | 0 | 0 | 0 | 0 | 0.329 | 0 | 0 | 0.426 | 0.598 |
| Daclizumab | IL2RA | Antibody | TNFRSF1A | 0 | 0 | 0 | 0 | 0 | 0 | 0 | 0.763 | 0.763 |
| Daclizumab | IL2RA | Antibody | STAT3 | 0 | 0 | 0 | 0 | 0.070 | 0 | 0 | 0.649 | 0.659 |
| Daclizumab | IL2RA | Antibody | FCRL3 | 0 | 0 | 0 | 0 | 0.122 | 0.125 | 0 | 0.370 | 0.473 |
| Daclizumab | IL2RA | Antibody | CD58 | 0 | 0 | 0 | 0 | 0.050 | 0 | 0 | 0.467 | 0.472 |
| Daclizumab | IL2RB | Antibody | FCRL3 | 0 | 0 | 0 | 0 | 0.281 | 0 | 0 | 0.219 | 0.414 |
| Daclizumab | IL2RB | Antibody | STAT3 | 0 | 0 | 0 | 0 | 0.047 | 0.292 | 0.500 | 0.663 | 0.871 |
| Daclizumab | IL2RB | Antibody | IL2RA | 0 | 0 | 0 | 0 | 0.148 | 0.851 | 0.900 | 0.995 | 0.999 |
| Daclizumab | IL2RB | Antibody | CD274 | 0 | 0 | 0 | 0 | 0.127 | 0 | 0 | 0.439 | 0.49 |
| Dimethyl fumarate | KEAP1 | Binder | STAT3 | 0 | 0 | 0 | 0 | 0.079 | 0 | 0 | 0.645 | 0.659 |
| Dimethyl fumarate | KEAP1 | Binder | CASP3 | 0 | 0 | 0 | 0 | 0.071 | 0 | 0 | 0.638 | 0.65 |
| Dimethyl fumarate | KEAP1 | Binder | CD274 | 0 | 0 | 0 | 0 | 0 | 0 | 0 | 0.432 | 0.432 |
| Dimethyl fumarate | RELA | Unknown | STAT3 | 0 | 0 | 0 | 0 | 0.171 | 0.793 | 0 | 0.959 | 0.992 |
| Dimethyl fumarate | RELA | Unknown | TNFRSF1A | 0 | 0 | 0 | 0 | 0.115 | 0 | 0 | 0.811 | 0.826 |
| Dimethyl fumarate | RELA | Unknown | IL2RA | 0 | 0 | 0 | 0 | 0 | 0.292 | 0 | 0.258 | 0.452 |
| Dimethyl fumarate | RELA | Unknown | CASP3 | 0 | 0 | 0 | 0 | 0.068 | 0.061 | 0 | 0.587 | 0.607 |
| Dimethyl fumarate | RELA | Unknown | CD274 | 0 | 0 | 0 | 0 | 0.059 | 0 | 0 | 0.57 | 0.578 |
| Dimethyl fumarate | RELA | Unknown | TLR1 | 0 | 0 | 0 | 0 | 0.074 | 0.125 | 0 | 0.373 | 0.448 |
| Fingolimod | HDAC1 | Inhibitor | STAT3 | 0 | 0 | 0 | 0 | 0.060 | 0.744 | 0.400 | 0.903 | 0.984 |
| Fingolimod | HDAC1 | Inhibitor | CASP3 | 0 | 0 | 0 | 0 | 0.074 | 0 | 0 | 0.728 | 0.738 |
| Fingolimod | S1PR1 | Modulator | STAT3 | 0 | 0 | 0 | 0 | 0.072 | 0 | 0.500 | 0.640 | 0.818 |
| Fingolimod | S1PR1 | Modulator | CXCL12 | 0 | 0 | 0 | 0 | 0.067 | 0.046 | 0 | 0.611 | 0.624 |
| Glatiramer | HLA-DRB1 | Unknown | FCRL3 | 0 | 0 | 0 | 0 | 0.080 | 0.087 | 0 | 0.455 | 0.502 |
| Glatiramer | HLA-DRB1 | Unknown | CD58 | 0 | 0 | 0 | 0 | 0.066 | 0.094 | 0 | 0.543 | 0.580 |
| Glatiramer | HLA-DRB1 | Unknown | IL2RA | 0 | 0 | 0 | 0 | 0.092 | 0 | 0 | 0.514 | 0.54 |
| Glatiramer | HLA-DRB1 | Unknown | CD274 | 0 | 0 | 0 | 0 | 0.073 | 0 | 0.5 | 0.370 | 0.682 |
| Interferon beta | IFNAR1 | Binder | STAT3 | 0 | 0 | 0 | 0 | 0.110 | 0.294 | 0 | 0.862 | 0.906 |
| Interferon beta | IFNAR1 | Binder | TNFRSF1A | 0 | 0 | 0 | 0 | 0.132 | 0 | 0 | 0.593 | 0.631 |
| Human interferon beta | IFNAR1 | Binder | CASP3 | 0 | 0 | 0 | 0 | 0.107 | 0 | 0 | 0.373 | 0.417 |
| Human interferon beta | IFNAR1 | Binder | CD274 | 0 | 0 | 0 | 0 | 0.059 | 0 | 0 | 0.478 | 0.489 |
| Human interferon beta | IFNAR1 | Binder | CXCL11 | 0 | 0 | 0 | 0 | 0.087 | 0 | 0 | 0.593 | 0.612 |
| Human interferon beta | IFNAR1 | Binder | TLR1 | 0 | 0 | 0 | 0 | 0.107 | 0 | 0 | 0.376 | 0.42 |
| Human interferon beta | IFNAR1 | Binder | CASP3 | 0 | 0 | 0 | 0 | 0.107 | 0 | 0 | 0.373 | 0.417 |
| Human interferon beta | IFNAR1 | Binder | CD274 | 0 | 0 | 0 | 0 | 0.059 | 0 | 0 | 0.478 | 0.489 |
| Human interferon beta | IFNAR1 | Binder | CXCL11 | 0 | 0 | 0 | 0 | 0.087 | 0 | 0 | 0.593 | 0.612 |
| Human interferon beta | IFNAR1 | Binder | TLR1 | 0 | 0 | 0 | 0 | 0.107 | 0 | 0 | 0.376 | 0.42 |
| Natalizuma | ITGA4 | Antibody | CD58 | 0 | 0 | 0 | 0 | 0.105 | 0 | 0 | 0.447 | 0.484 |
| Natalizuma | ITGA4 | Antibody | CXCL12 | 0 | 0 | 0 | 0 | 0.072 | 0 | 0 | 0.518 | 0.533 |
| Natalizuma | ITGA4 | Antibody | JAM3 | 0 | 0 | 0 | 0 | 0.047 | 0 | 0.500 | 0.679 | 0.833 |
| Natalizumab | FCGR1A | Unknown | CR1 | 0 | 0 | 0 | 0 | 0.085 | 0 | 0 | 0.571 | 0.590 |
| Natalizumab | FCGR1A | Unknown | STAT3 | 0 | 0 | 0 | 0 | 0.061 | 0 | 0 | 0.404 | 0.417 |

| | | | | | | | | | | | | |
|---|---|---|---|---|---|---|---|---|---|---|---|---|
| Natalizumab | FCGR1A | Unknown | TNFRSF1A | 0 | 0 | 0 | 0 | 0.060 | 0 | 0 | 0.575 | 0.583 |
| Natalizumab | FCGR1A | Unknown | CD274 | 0 | 0 | 0 | 0 | 0.126 | 0.06 | 0 | 0.678 | 0.712 |
| Natalizumab | FCGR1A | Unknown | TLR1 | 0 | 0 | 0 | 0 | 0.192 | 0.095 | 0 | 0.32 | 0.459 |
| Natalizumab | FCGR3B | Unknown | CD58 | 0 | 0 | 0 | 0 | 0.106 | 0 | 0 | 0.596 | 0.623 |
| Natalizumab | FCGR3B | Unknown | CD59 | 0 | 0 | 0 | 0 | 0 | 0 | 0 | 0.448 | 0.448 |
| Natalizumab | FCGR3B | Unknown | CR1 | 0 | 0 | 0 | 0 | 0.142 | 0 | 0 | 0.723 | 0.752 |
| Natalizumab | FCGR3B | Unknown | STAT3 | 0 | 0 | 0 | 0 | 0.086 | 0 | 0 | 0.660 | 0.676 |
| Natalizumab | FCGR3B | Unknown | TNFRSF1A | 0 | 0 | 0 | 0 | 0.064 | 0 | 0 | 0.419 | 0.432 |
| Natalizumab | FCGR3B | Unknown | IL2RA | 0 | 0 | 0 | 0 | 0.062 | 0 | 0 | 0.541 | 0.551 |
| Natalizumab | FCGR3B | Unknown | CASP3 | 0 | 0 | 0 | 0 | 0.068 | 0 | 0 | 0.439 | 0.455 |
| Natalizumab | FCGR3B | Unknown | CD274 | 0 | 0 | 0 | 0 | 0.127 | 0 | 0 | 0.867 | 0.879 |
| Natalizumab | FCGR3B | Unknown | CXCL11 | 0 | 0 | 0 | 0 | 0.134 | 0 | 0 | 0.419 | 0.475 |
| Natalizumab | FCGR3B | Unknown | CXCL12 | 0 | 0 | 0 | 0 | 0.098 | 0 | 0 | 0.512 | 0.541 |
| Natalizumab | FCGR3B | Unknown | TLR1 | 0 | 0 | 0 | 0 | 0.329 | 0 | 0 | 0.426 | 0.598 |
| Natalizumab | ICAM1 | Unknown | CD58 | 0 | 0 | 0 | 0 | 0.101 | 0 | 0 | 0.996 | 0.996 |
| Natalizumab | ICAM1 | Unknown | CD59 | 0 | 0 | 0 | 0 | 0.056 | 0 | 0 | 0.431 | 0.439 |
| Natalizumab | ICAM1 | Unknown | CR1 | 0 | 0 | 0 | 0 | 0.058 | 0 | 0 | 0.397 | 0.409 |
| Natalizumab | ICAM1 | Unknown | STAT3 | 0 | 0 | 0 | 0 | 0.122 | 0 | 0.500 | 0.649 | 0.832 |
| Natalizumab | ICAM1 | Unknown | TNFRSF1A | 0 | 0 | 0 | 0 | 0.066 | 0 | 0 | 0.736 | 0.742 |
| Natalizumab | ICAM1 | Unknown | HP | 0 | 0 | 0 | 0 | 0.088 | 0 | 0 | 0.398 | 0.427 |
| Natalizumab | ICAM1 | Unknown | IL2RA | 0 | 0 | 0 | 0 | 0.066 | 0.292 | 0 | 0.666 | 0.759 |
| Natalizumab | ICAM1 | Unknown | MMP13 | 0 | 0 | 0 | 0 | 0 | 0 | 0 | 0.564 | 0.564 |
| Natalizumab | ICAM1 | Unknown | CASP3 | 0 | 0 | 0 | 0 | 0.057 | 0 | 0 | 0.629 | 0.635 |
| Natalizumab | ICAM1 | Unknown | CD274 | 0 | 0 | 0 | 0 | 0.188 | 0 | 0 | 0.713 | 0.757 |
| Natalizumab | ICAM1 | Unknown | CXCL11 | 0 | 0 | 0 | 0 | 0.168 | 0 | 0 | 0.541 | 0.602 |
| Natalizumab | ICAM1 | Unknown | CXCL12 | 0 | 0 | 0 | 0 | 0 | 0 | 0 | 0.67 | 0.67 |
| Natalizumab | ICAM1 | Unknown | JAM3 | 0 | 0 | 0 | 0 | 0 | 0 | 0 | 0.625 | 0.625 |
| Natalizumab | ICAM1 | Unknown | TLR1 | 0 | 0 | 0 | 0 | 0.167 | 0 | 0 | 0.434 | 0.508 |
| Natalizumab | ITGA4 | Antibody | CXCL12 | 0 | 0 | 0 | 0 | 0.072 | 0 | 0 | 0.518 | 0.533 |
| Natalizumab | ITGA4 | Antibody | JAM3 | 0 | 0 | 0 | 0 | 0.047 | 0 | 0.5 | 0.679 | 0.833 |
| Ocrelizumab | MS4A1 | Antibody | FCRL3 | 0 | 0 | 0 | 0 | 0.511 | 0.173 | 0 | 0.317 | 0.700 |
| Ocrelizumab | MS4A1 | Antibody | IL2RA | 0 | 0 | 0 | 0 | 0.084 | 0 | 0 | 0.390 | 0.417 |
| Ofatumumab | MS4A1 | Antibody | FCRL3 | 0 | 0 | 0 | 0 | 0.511 | 0.173 | 0 | 0.317 | 0.700 |
| Ofatumumab | MS4A1 | Antibody | IL2RA | 0 | 0 | 0 | 0 | 0.084 | 0 | 0 | 0.39 | 0.417 |
| Ozanimod | S1PR1 | Agonist | STAT3 | 0 | 0 | 0 | 0 | 0.072 | 0 | 0.500 | 0.640 | 0.818 |
| Ozanimod | S1PR1 | Agonist | CXCL12 | 0 | 0 | 0 | 0 | 0.067 | 0.046 | 0 | 0.611 | 0.624 |
| Siponimod | S1PR1 | Unknown | STAT3 | 0 | 0 | 0 | 0 | 0.072 | 0 | 0.500 | 0.640 | 0.818 |
| Siponimod | S1PR1 | Unknown | STAT3 | 0 | 0 | 0 | 0 | 0.072 | 0 | 0.500 | 0.640 | 0.818 |
| Siponimod | S1PR1 | Unknown | CXCL12 | 0 | 0 | 0 | 0 | 0.067 | 0.046 | 0 | 0.611 | 0.624 |
| Teriflunomide | DHODH | Inhibitor | BCAT2 | 0.108 | 0 | 0 | 0 | 0.110 | 0 | 0 | 0.376 | 0.462 |
| Teriflunomide | DHODH | Inhibitor | TYMP | 0.06 | 0 | 0 | 0 | 0 | 0 | 0.792 | 0.363 | 0.864 |
| Ublituximab | MS4A1 | Binder; Antibody | IL2RA | 0 | 0 | 0 | 0 | 0.084 | 0 | 0 | 0.390 | 0.417 |
| Ublituximab | MS4A1 | Binder; Antibody | FCRL3 | 0 | 0 | 0 | 0 | 0.511 | 0.173 | 0 | 0.317 | 0.700 |

MS: multiple sclerosis; Daclizumab is not anymore a licensed drug for MS, due to severe liver toxicity

**Table S27. Protein-protein interaction analysis of relaxed-criteria-based brain proteins with multiple sclerosis drug targets (minimum combined score of 0.4)**

| MS drug | MS drug target | cological action of | Potential target | rhood_on_chro | gene_fusion | netic_coocc | homology | coexpressio | y_determin | base_annot | nated_textm | combined_score |
|---|---|---|---|---|---|---|---|---|---|---|---|---|
| Natalizumab | ICAM1 | Unknown | ALCAM | 0 | 0 | 0 | 0 | 0.062 | 0 | 0 | 0.750 | 0.755 |
| Natalizumab | ITGA4 | Antibody | ALCAM | 0 | 0 | 0 | 0 | 0 | 0 | 0 | 0.435 | 0.435 |
| Dimethyl fumarate | RELA | Unknown | ANXA6 | 0 | 0 | 0 | 0 | 0.071 | 0 | 0 | 0.772 | 0.779 |
| Dimethyl fumarate | RELA | Unknown | TRAF3 | 0 | 0 | 0 | 0 | 0.159 | 0.045 | 0 | 0.596 | 0.647 |
| Teriflunomide | DHODH | Inhibitor | SHMT1 | 0 | 0 | 0 | 0 | 0.133 | 0 | 0 | 0.339 | 0.403 |
| Teriflunomide | DHODH | Inhibitor | TTC5 | 0.091 | 0 | 0 | 0 | 0.208 | 0.071 | 0.152 | 0.266 | 0.507 |
| Alemtuzumab | FCGR3A | Unknown | GIMAP4 | 0 | 0 | 0 | 0 | 0.384 | 0 | 0 | 0.068 | 0.401 |
| Alemtuzumab | FCGR3A | Unknown | GIMAP4 | 0 | 0 | 0 | 0 | 0.384 | 0 | 0 | 0.068 | 0.401 |
| Alemtuzumab | FCGR3A | Unknown | HLA-B | 0 | 0 | 0 | 0 | 0.183 | 0 | 0 | 0.450 | 0.532 |
| Alemtuzumab | FCGR3B | Unknown | HLA-B | 0 | 0 | 0 | 0 | 0.123 | 0 | 0 | 0.448 | 0.495 |
| Glatiramer | HLA-DRB1 | Unknown | HLA-B | 0 | 0 | 0 | 0.619 | 0.168 | 0 | 0.900 | 0.602 | 0.963 |
| Daclizumab | C1R | Unknown | HLA-B | 0 | 0 | 0 | 0 | 0.161 | 0 | 0 | 0.435 | 0.505 |
| Daclizumab | C1QB | Unknown | GIMAP4 | 0 | 0 | 0 | 0 | 0.332 | 0.095 | 0 | 0.105 | 0.411 |
| Daclizumab | C1QA | Unknown | GIMAP4 | 0 | 0 | 0 | 0 | 0.335 | 0.095 | 0 | 0.117 | 0.422 |
| Interferon beta | IFNAR1 | Binder | IFIT5 | 0 | 0 | 0 | 0 | 0.081 | 0 | 0 | 0.375 | 0.401 |
| Interferon beta | IFNAR1 | Binder | TRAF3 | 0 | 0 | 0 | 0 | 0.049 | 0.071 | 0 | 0.518 | 0.537 |
| Interferon beta | IFNAR1 | Binder | TREX1 | 0 | 0 | 0 | 0 | 0.065 | 0 | 0 | 0.668 | 0.676 |
| Cladribine | PNP | Inducer | QPRT | 0 | 0 | 0 | 0 | 0 | 0 | 0 | 0.420 | 0.420 |
| Cladribine | RRM1 | Inhibitor | SHMT1 | 0.057 | 0 | 0 | 0 | 0.072 | 0 | 0 | 0.413 | 0.441 |
| Cladribine | RRM1 | Inhibitor | TXN | 0.057 | 0 | 0 | 0 | 0.223 | 0 | 0.750 | 0.306 | 0.856 |
| Cladribine | RRM2 | Inhibitor | TXN | 0 | 0 | 0 | 0 | 0.102 | 0 | 0.750 | 0.529 | 0.885 |
| Cladribine | RRM2B | Inhibitor | TXN | 0 | 0 | 0 | 0 | 0.078 | 0 | 0.750 | 0.248 | 0.811 |

MS: multiple sclerosis; Daclizumab is not anymore a licensed drug for MS, due to severe liver toxicity

**Table S28. Protein-protein interaction analysis among all relaxed-criteria-based potential causal proteins (minimum combined**

| Node1 | Node2 | rhood_on_chro | gene_fusion | netic_coocc | homology | coexpression | y_determin | base_annot | nated_textm | combined_score |
|---|---|---|---|---|---|---|---|---|---|---|
| **Interactions of proteins in plasma** | | | | | | | | | | |
| CD58 | CD59 | 0 | 0 | 0 | 0 | 0.062 | 0 | 0 | 0.649 | 0.656 |
| CD59 | CR1 | 0 | 0 | 0 | 0 | 0 | 0 | 0 | 0.824 | 0.824 |
| MMP10 | TCN1 | 0 | 0 | 0 | 0 | 0.080 | 0 | 0 | 0.506 | 0.526 |
| PGLYRP1 | TCN1 | 0 | 0 | 0 | 0 | 0.182 | 0 | 0 | 0.336 | 0.433 |
| PGLYRP1 | TNFRSF1A | 0 | 0 | 0 | 0 | 0.060 | 0 | 0 | 0.579 | 0.587 |
| STAT3 | TNFRSF1A | 0 | 0 | 0 | 0 | 0.266 | 0 | 0.500 | 0.571 | 0.829 |
| CASP3 | TNFRSF1A | 0 | 0 | 0 | 0 | 0.052 | 0 | 0 | 0.852 | 0.853 |
| CASP3 | STAT3 | 0 | 0 | 0 | 0 | 0.055 | 0 | 0 | 0.801 | 0.804 |
| CASP3 | CD274 | 0 | 0 | 0 | 0 | 0.076 | 0 | 0 | 0.506 | 0.524 |
| CASP3 | CXCL12 | 0 | 0 | 0 | 0 | 0.086 | 0 | 0 | 0.52 | 0.543 |
| CD274 | TNFRSF1A | 0 | 0 | 0 | 0 | 0.071 | 0 | 0 | 0.447 | 0.464 |
| CD274 | STAT3 | 0 | 0 | 0 | 0 | 0.124 | 0 | 0.75 | 0.967 | 0.992 |
| CD274 | CXCL11 | 0 | 0 | 0 | 0 | 0.224 | 0 | 0 | 0.543 | 0.63 |
| CD274 | CD58 | 0 | 0 | 0 | 0 | 0.08 | 0 | 0 | 0.594 | 0.61 |
| CD274 | CXCL12 | 0 | 0 | 0 | 0 | 0.077 | 0 | 0 | 0.747 | 0.757 |
| CXCL11 | STAT3 | 0 | 0 | 0 | 0 | 0.101 | 0 | 0 | 0.447 | 0.482 |
| CXCL11 | CBR3 | 0 | 0 | 0 | 0 | 0.067 | 0 | 0 | 0.401 | 0.417 |
| CXCL11 | CXCL12 | 0 | 0 | 0 | 0 | 0.118 | 0.994 | 0.5 | 0.862 | 0.999 |
| CXCL12 | TNFRSF1A | 0 | 0 | 0 | 0 | 0.048 | 0 | 0 | 0.399 | 0.403 |
| CXCL12 | STAT3 | 0 | 0 | 0 | 0 | 0.103 | 0 | 0 | 0.661 | 0.684 |
| CXCL12 | AHSG | 0 | 0 | 0 | 0 | 0.042 | 0 | 0 | 0.438 | 0.438 |
| CXCL12 | TLR1 | 0 | 0 | 0 | 0 | 0.08 | 0 | 0 | 0.471 | 0.493 |
| IL2RA | TNFRSF1A | 0 | 0 | 0 | 0 | 0 | 0 | 0 | 0.763 | 0.763 |
| IL2RA | STAT3 | 0 | 0 | 0 | 0 | 0.07 | 0 | 0 | 0.649 | 0.659 |
| IL2RA | CXCL11 | 0 | 0 | 0 | 0 | 0.163 | 0 | 0 | 0.352 | 0.434 |
| IL2RA | FCRL3 | 0 | 0 | 0 | 0 | 0.122 | 0.125 | 0 | 0.37 | 0.473 |
| IL2RA | CD58 | 0 | 0 | 0 | 0 | 0.05 | 0 | 0 | 0.467 | 0.472 |
| IL2RA | CXCL12 | 0 | 0 | 0 | 0 | 0.044 | 0 | 0 | 0.399 | 0.401 |
| IL2RA | CD274 | 0 | 0 | 0.302 | 0 | 0.111 | 0 | 0 | 0.497 | 0.661 |
| MMP13 | TCN1 | 0 | 0 | 0 | 0 | 0.05 | 0 | 0 | 0.423 | 0.428 |
| MMP13 | CXCL12 | 0 | 0 | 0 | 0 | 0.082 | 0 | 0 | 0.476 | 0.499 |
| MMP13 | CASP3 | 0 | 0 | 0 | 0 | 0.088 | 0 | 0 | 0.57 | 0.592 |
| MMP13 | STAT3 | 0 | 0 | 0 | 0 | 0.055 | 0 | 0 | 0.65 | 0.655 |
| MMP13 | MMP10 | 0 | 0 | 0.07 | 0.94 | 0.268 | 0 | 0.5 | 0.119 | 0.66 |
| TLR1 | TNFRSF1A | 0 | 0 | 0 | 0 | 0.082 | 0 | 0 | 0.374 | 0.401 |
| TLR1 | STAT3 | 0 | 0 | 0 | 0 | 0.06 | 0.045 | 0 | 0.414 | 0.429 |

| | | | | | | | | | | |
|---|---|---|---|---|---|---|---|---|---|---|
| TNFSF12 | TNFRSF1A | 0 | 0 | 0 | 0 | 0.06 | 0 | 0 | 0.847 | 0.85 |
| HP | AHSG | 0 | 0 | 0 | 0 | 0.220 | 0.107 | 0 | 0.845 | 0.882 |
| HP | CD59 | 0 | 0 | 0 | 0 | 0.054 | 0 | 0 | 0.417 | 0.424 |
| TYMP | WARS | 0.119 | 0 | 0 | 0 | 0.302 | 0 | 0 | 0.108 | 0.403 |
| **Interactions of proteins in brain** | | | | | | | | | | |
| AUH | HMGCL | 0.059 | 0.082 | 0 | 0 | 0.211 | 0 | 0.941 | 0.627 | 0.982 |
| DOC2A | QPRT | 0 | 0 | 0 | 0 | 0 | 0 | 0 | 0.542 | 0.542 |
| ERAP2 | HLA-B | 0 | 0 | 0 | 0 | 0.087 | 0 | 0 | 0.597 | 0.616 |
| FOLH1 | SHMT1 | 0 | 0 | 0 | 0 | 0.073 | 0 | 0 | 0.404 | 0.424 |
| FOLH1 | MTHFR | 0 | 0 | 0 | 0 | 0 | 0 | 0 | 0.546 | 0.546 |
| HMGCL | MTHFR | 0.111 | 0 | 0 | 0 | 0.105 | 0 | 0 | 0.393 | 0.475 |
| MTHFR | SHMT1 | 0.113 | 0 | 0 | 0 | 0.290 | 0 | 0.978 | 0.913 | 0.998 |
| QPRT | TXN | 0 | 0 | 0 | 0 | 0.047 | 0 | 0 | 0.397 | 0.400 |
| SFXN4 | TSFM | 0 | 0 | 0 | 0 | 0.201 | 0 | 0 | 0.369 | 0.474 |
| TSFM | TTC5 | 0.102 | 0 | 0 | 0 | 0.243 | 0 | 0 | 0.24 | 0.438 |
| WARS | TSFM | 0 | 0 | 0 | 0 | 0.114 | 0 | 0 | 0.408 | 0.454 |
| **Interactions of proteins between plasma and brain (node1: plasma protein; node2: brain protein)** | | | | | | | | | | |
| AHSG | ANXA6 | 0 | 0 | 0 | 0 | 0.075 | 0 | 0 | 0.598 | 0.612 |
| HP | ANXA6 | 0 | 0 | 0 | 0 | 0.042 | 0 | 0 | 0.449 | 0.449 |
| CD58 | ALCAM | 0 | 0 | 0 | 0 | 0.049 | 0.042 | 0 | 0.397 | 0.402 |
| CD59 | ALCAM | 0 | 0 | 0 | 0 | 0.062 | 0 | 0 | 0.444 | 0.456 |
| BCAT2 | SHMT1 | 0.111 | 0 | 0 | 0 | 0.106 | 0 | 0 | 0.436 | 0.513 |
| BCAT2 | HMGCL | 0.111 | 0 | 0 | 0 | 0.684 | 0.077 | 0 | 0.467 | 0.843 |
| TNFRSF1A | ERAP2 | 0 | 0 | 0 | 0 | 0 | 0 | 0 | 0.429 | 0.429 |
| KYNU | QPRT | 0 | 0 | 0 | 0 | 0.120 | 0.087 | 0 | 0.846 | 0.866 |
| TNFRSF1A | MADD | 0 | 0 | 0 | 0 | 0 | 0.292 | 0.700 | 0.491 | 0.882 |
| TCN1 | MTHFR | 0 | 0 | 0 | 0 | 0.055 | 0 | 0 | 0.399 | 0.407 |
| STAT3 | TRAF3 | 0 | 0 | 0 | 0 | 0.06 | 0 | 0 | 0.423 | 0.435 |
| STAT3 | TXN | 0 | 0 | 0 | 0 | 0.044 | 0.292 | 0 | 0.431 | 0.581 |
| ARFIP1 | ICA1L | 0 | 0 | 0 | 0 | 0 | 0 | 0 | 0.401 | 0.401 |
| ATXN3 | TXN | 0 | 0 | 0 | 0 | 0 | 0.15 | 0 | 0.429 | 0.493 |
| CASP3 | QPRT | 0 | 0 | 0 | 0 | 0 | 0 | 0 | 0.467 | 0.467 |
| CASP3 | TRAF3 | 0 | 0 | 0 | 0 | 0.103 | 0.51 | 0 | 0.394 | 0.71 |
| CASP3 | TXN | 0 | 0 | 0 | 0 | 0.056 | 0.441 | 0 | 0.751 | 0.857 |
| CD274 | FOLH1 | 0 | 0 | 0 | 0 | 0 | 0 | 0 | 0.52 | 0.52 |
| CD274 | HLA-B | 0 | 0 | 0 | 0 | 0.123 | 0 | 0 | 0.507 | 0.549 |
| CBR3 | IFIT5 | 0 | 0 | 0 | 0 | 0.24 | 0 | 0 | 0.25 | 0.406 |
| CXCL12 | ALCAM | 0 | 0 | 0 | 0 | 0 | 0 | 0 | 0.445 | 0.445 |
| CXCL12 | TXN | 0 | 0 | 0 | 0 | 0 | 0 | 0 | 0.472 | 0.472 |

| | | | | | | | | | | |
|---|---|---|---|---|---|---|---|---|---|---|
| JAM3 | ALCAM | 0 | 0 | 0 | 0 | 0 | 0.075 | 0 | 0.413 | 0.433 |
| PFKM | TRAP1 | 0 | 0 | 0 | 0 | 0.105 | 0.292 | 0 | 0.168 | 0.427 |
| PFKM | SHMT1 | 0 | 0 | 0 | 0 | 0.11 | 0 | 0 | 0.409 | 0.452 |
| TAPBPL | ERAP2 | 0 | 0 | 0 | 0 | 0.095 | 0 | 0 | 0.511 | 0.538 |
| TAPBPL | HLA-B | 0 | 0 | 0 | 0 | 0.13 | 0.322 | 0 | 0.431 | 0.635 |
| TLR1 | TRAF3 | 0 | 0 | 0 | 0 | 0.074 | 0.073 | 0 | 0.465 | 0.501 |
| WARS | TSFM | 0 | 0 | 0 | 0 | 0.114 | 0 | 0 | 0.408 | 0.454 |
| TNFSF12 | SHMT1 | 0 | 0 | 0 | 0 | 0 | 0 | 0 | 0.652 | 0.652 |
| TNFSF12 | TRAF3 | 0 | 0 | 0 | 0 | 0.045 | 0 | 0.5 | 0.669 | 0.828 |
| TNFRSF1A | TRAP1 | 0 | 0 | 0 | 0 | 0 | 0.422 | 0 | 0.393 | 0.634 |
| TNFRSF1A | TRAF3 | 0 | 0 | 0 | 0 | 0 | 0.311 | 0 | 0.969 | 0.977 |

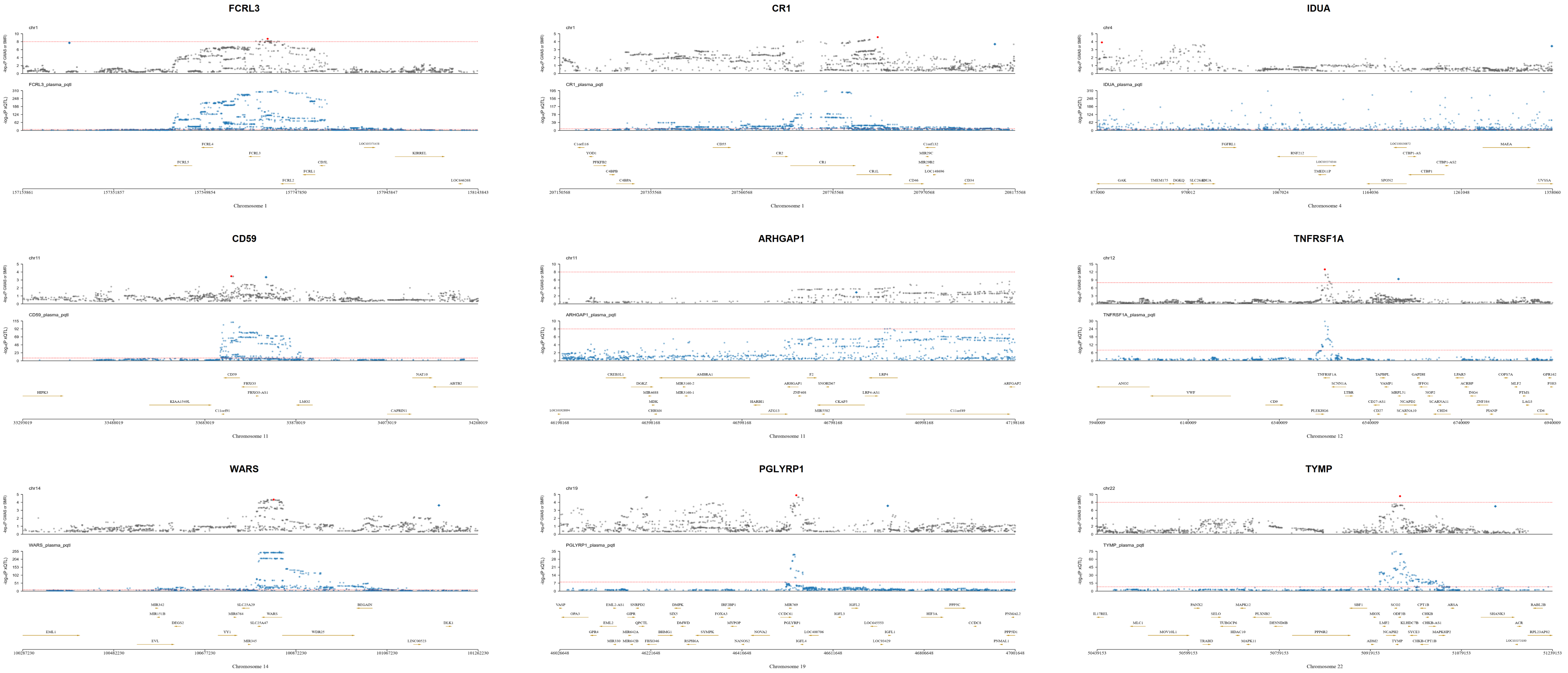
FCRL3
CR1
IDUA
Chromosome 1
Chromosome 1
Chromosome 4
CD59
ARHGAP1
TNFRSF1A
Chromosome 11
Chromosome 11
Chromosome 12
WARS
PGLYRP1
TYMP
Chromosome 14
Chromosome 19
Chromosome 22

**Supplementary Figure S1.** Regional visualization of pQTL, GWAS signals, and annotated neighboring genes for prioritized plasma proteins linked to multiple sclerosis.

This figure displays the regional plot of pQTL, GWAS signals, and annotated neighboring genes of prioritized plasma proteins potentially causal impact multiple sclerosis. The top panel shows $-\log_{10}$(*P*-value) of GWAS or SMR signals in the given region. A red dashed line indicates the genome-wide significance threshold. The red point indicates the top associated variant in each region, and the blue diamond indicates a variant exhibiting both a strong pQTL signal and a GWAS association, used in the SMR analysis. The middle panel presents the $-\log_{10}$(*P*-value) of pQTL, with a significance threshold marked in red. The bottom panel depicts the genomic region, including the gene encoding the prioritized protein and its neighboring genes. The x-axis represents the chromosomal position (hg19), while the y-axes denote the strength of association.

pQTL: protein quantitative trait loci; GWAS: genome-wide association study; SMR: Summary-based Mendelian Randomization

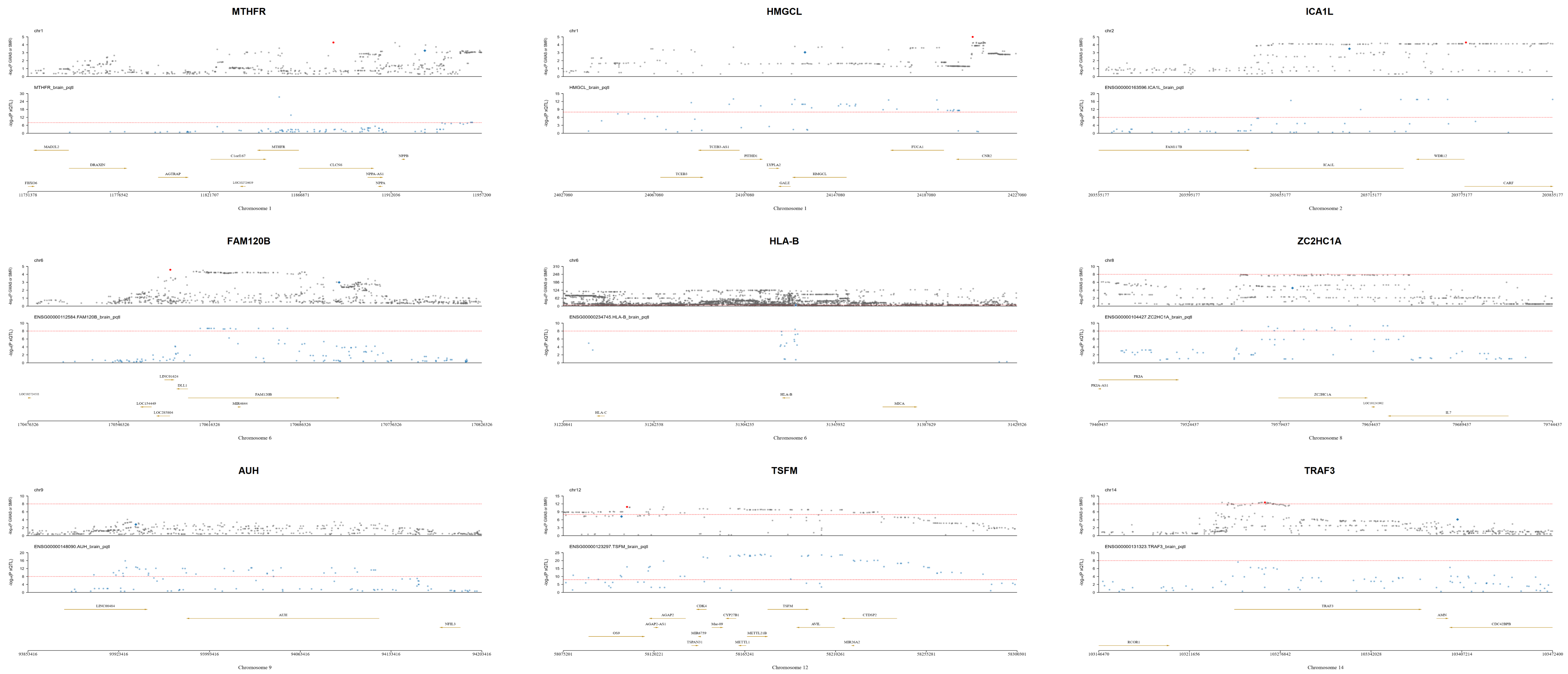
MTHFR
HMGCL
ICA1L
Chromosome 1
Chromosome 1
Chromosome 2
FAM120B
HLA-B
ZC2HC1A
Chromosome 6
Chromosome 6
Chromosome 8
AUH
TSFM
TRAF3
Chromosome 9
Chromosome 12
Chromosome 14

**Supplementary Figure S2.** Regional visualization of pQTL, GWAS signals, and annotated neighboring genes for prioritized brain proteins linked to multiple sclerosis.

This figure displays the regional plot of pQTL, GWAS signals, and annotated neighboring genes of prioritized brain proteins potentially causal impact on multiple sclerosis. The top panel shows $-\log_{10}(P$-value) of GWAS or SMR signals in the given region. A red dashed line indicates the genome-wide significance threshold. The red point indicates the top associated variant in this given region, and the blue diamond indicates a variant exhibiting both a strong pQTL signal and a GWAS association, used in the SMR analysis. The middle panel presents the $-\log_{10}(P$-value) of pQTL, with a significance threshold marked in red. The bottom panel depicts the genomic region, including the gene encoding the prioritized protein and its neighboring genes. The x-axis represents the chromosomal position (hg19), while the y-axes denote the strength of association.

pQTL: protein quantitative trait loci; GWAS: genome-wide association study; SMR: Summary-based Mendelian Randomization

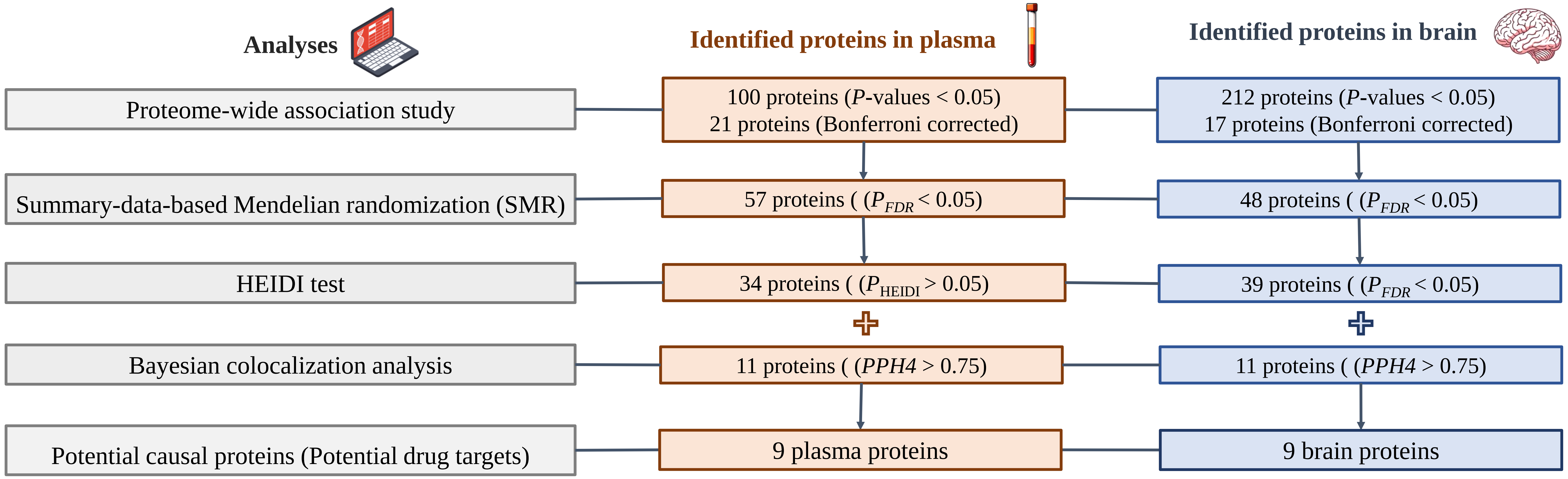
Analyses
Identified proteins in plasma
Identified proteins in brain
Proteome-wide association study
100 proteins (P-values < 0.05)
21 proteins (Bonferroni corrected)
212 proteins (P-values < 0.05)
17 proteins (Bonferroni corrected)
Summary-data-based Mendelian randomization (SMR)
57 proteins ( (P_FDR < 0.05)
48 proteins ( (P_FDR < 0.05)
HEIDI test
34 proteins ( (P_HEIDI > 0.05)
39 proteins ( (P_FDR < 0.05)
Bayesian colocalization analysis
11 proteins ( (PPH4 > 0.75)
11 proteins ( (PPH4 > 0.75)
Potential causal proteins (Potential drug targets)
9 plasma proteins
9 brain proteins

**Supplementary Figure S3. Number of proteins implicated in MS susceptibility at each step of identification analysis**

The number of proteins implicated in MS susceptibility identified through PWAS, SMR/HEIDI, and Coloc are present. The first column lists the names of the analyses performed at each step. The second and third columns show the corresponding number of plasma proteins and brain proteins identified in each analysis, respectively.

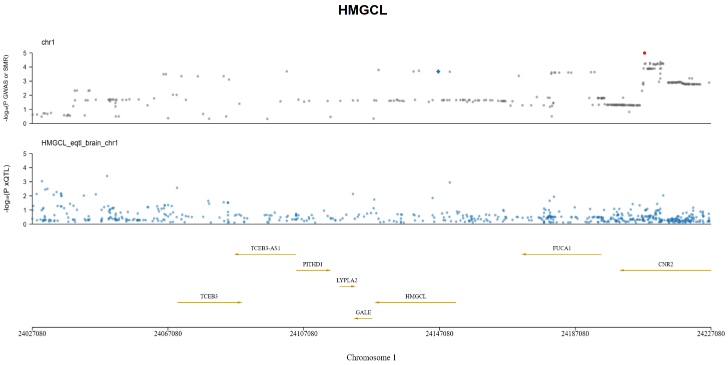
HMGCL
chr1
-log10(P GWAS or SMR)
HMGCL_eqtl_brain_chr1
-log10(P xQTL)
TCEB3-AS1
FUCA1
PITHD1
CNR2
LYPLA2
TCEB3
HMGCL
GALE
24027080
24067080
24107080
24147080
24187080
24227080
Chromosome 1

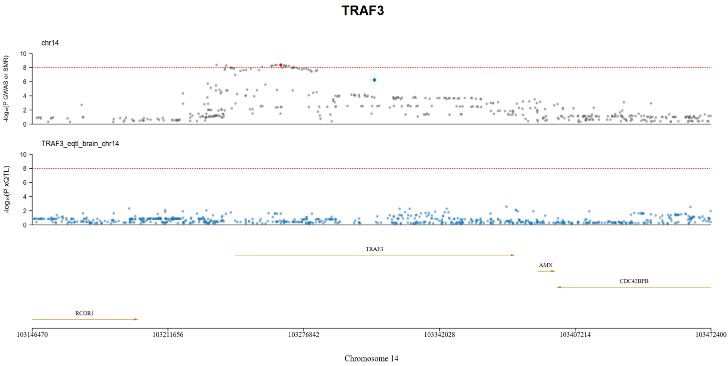
TRAF3
chr14
-log10(P GWAS or SMR)
TRAF3_eqtl_brain_chr14
-log10(P xQTL)
TRAF3
AMN
CDC42BPB
RCOR1
103146470
103211656
103276842
103342028
103407214
103472400
Chromosome 14

**Supplementary Figure S4.** Regional visualization of eQTL, GWAS signals, and annotated neighboring genes for prioritized brain proteins linked to multiple sclerosis.

This figure displays the regional plot of eQTL, GWAS signals, and annotated neighboring genes of prioritized brain proteins potentially causal impact on multiple sclerosis. The top panel shows $-\log_{10}$(*P*-value) of GWAS or SMR signals in the given region. A red dashed line indicates the genome-wide significance threshold. The red point indicates the top associated variant in this given region, and the blue diamond indicates a variant exhibiting both a strong eQTL signal and a GWAS association, used in the SMR analysis. The middle panel presents the $-\log_{10}$(*P*-value) of eQTL, with a significance threshold marked in red. The bottom panel depicts the genomic region, including the gene encoding the prioritized protein and its neighboring genes. The x-axis represents the chromosomal position (hg19), while the y-axes denote the strength of association.

eQTL: expression quantitative trait loci; GWAS: genome-wide association study; SMR: Summary-based Mendelian Randomization

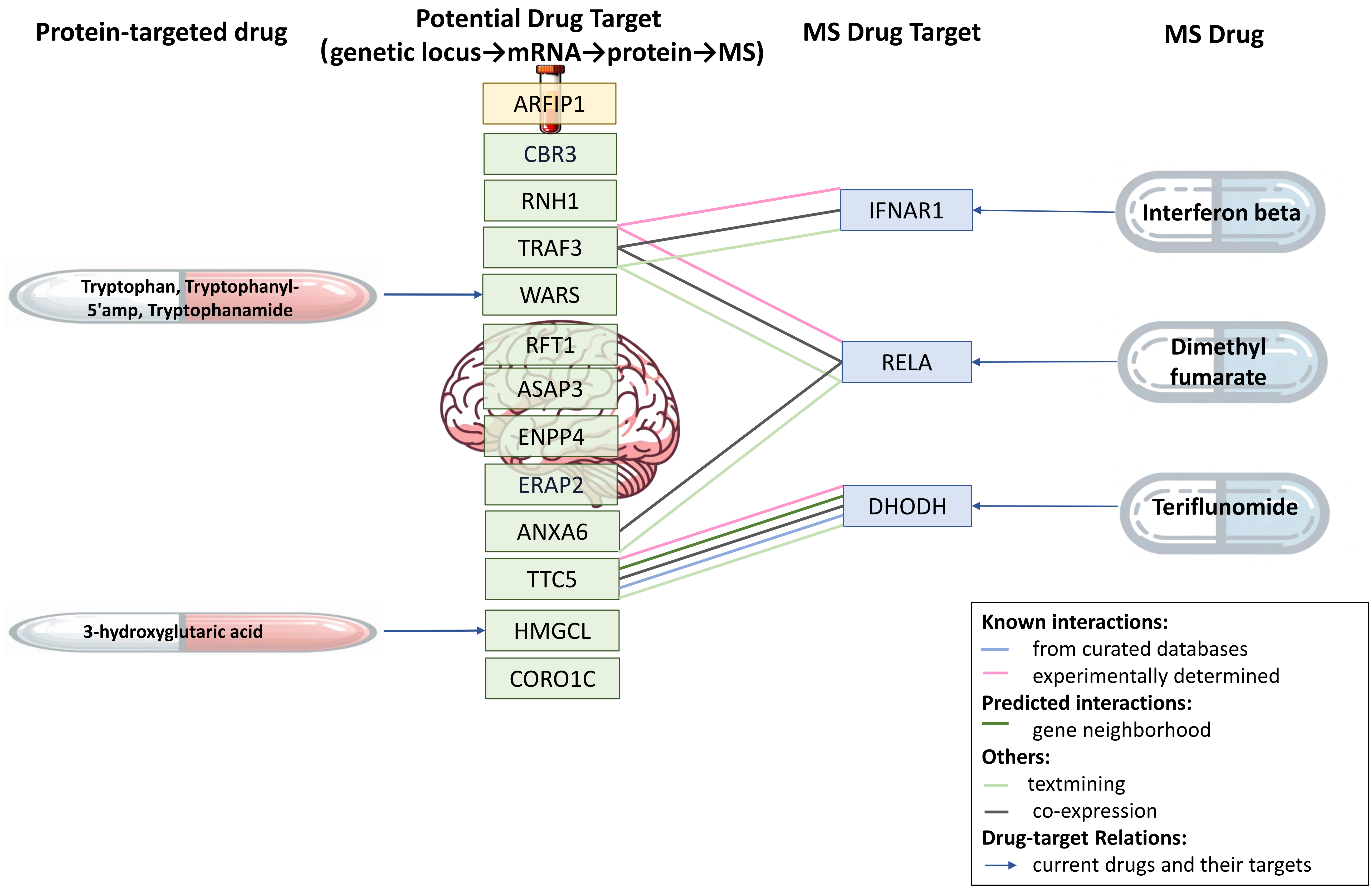

Protein-targeted drug
Potential Drug Target
(genetic locus→mRNA→protein→MS)
MS Drug Target
MS Drug
ARFIP1
CBR3
RNH1
TRAF3
WARS
RFT1
ASAP3
ENPP4
ERAP2
ANXA6
TTC5
HMGCL
CORO1C
Tryptophan, Tryptophanyl-5'amp, Tryptophanamide
3-hydroxyglutaric acid
IFNAR1
RELA
DHODH
Interferon beta
Dimethyl fumarate
Teriflunomide
Known interactions:
from curated databases
experimentally determined
Predicted interactions:
gene neighborhood
Others:
textmining
co-expression
Drug-target Relations:
current drugs and their targets

**Supplementary Figure S5. Potential drug targets, protein-targeted drugs, and protein-protein interaction networks of proteins that adhere to the central dogma of molecular biology**

The differential mRNA expression of potential causal proteins (genetic locus → protein translation → MS ) has also been proved. Therefore, these proteins adhere to the central dogma of molecular biology (genetic locus → mRNA transcription → protein translation → MS). The corresponding encoding genes for these potential causal proteins are presented. Gene names in red indicate that their corresponding proteins adhere to the central dogma of molecular biology. Potential drug targets highlighted in yellow boxes are identified in plasma, and those in green boxes are identified in brain. Drug relations refer to the existing drugs targeting the identified potential drug targets. MS drug targets refer to proteins targeted by existing MS drugs. The protein-protein interaction network is presented between potential drug targets and current MS drug targets, with lines of different colors representing various types of interaction.

MS: multiple sclerosis; HEIDI: heterogeneity in dependent instruments test.
